# Supplementary material for: Identification of rare alternative splicing events in MS/MS data reveals a significant fraction of alternative translation initiation sites
Source: PeerJ. 2014 Nov 13;2:e673. doi: 10.7717/peerj.673 (PMC4232841; doi:10.7717/peerj.673)
Supplement: File S2 — Peptides in yellow are specific to the Splooce database and do not exist in Uniprot as tryptic peptides. [file peerj-02-673-s002.zip › Supplementary_S2.html]

```
# Supplementary Material (S2)

List and sequence alignment of all identified events characterizing TIS changes.
  Peptides in yellow are specific to the Splooce database and do not exist in Uniprot as tryptic peptides.

----------------------------------------------------------------------------------------------------


P35637 (Uniprot)	versus
NM_004960#(-s-s-s-s-s-:16_F9638643950132) (Splooce)

For more details about the Alternative Splicing Event -> Link to Splooce page

Peptides that support the ASE (Splooce-specific):
LNLYTDR (PEAKS)
INLYTDR (MAXQUANT)

Alignment:
Uniprot       MASNDYTQQATQSYGAYPTQPGQGYSQQSSQPYGQQSYSGYSQSTDTSGYGQSSYSSYGQSQNTGYGTQSTPQGYGSTGGYGSSQSSQSSYGQQSSYPGY
Splooce       ----------------------------------------------------------------------------------------------------

Uniprot       GQQPAPSSTSGSYGSSSQSSSYGQPQSGSYSQQPSYGGQQQSYGQQQSYNPPQGYGQQNQYNSSSGGGGGGGGGGNYGQDQSSMSSGGGSGGGYGNQDQS
Splooce       ----------------------------------------------------------------------------------------------------

Uniprot       GGGGSGGYGQQDRGGRGRGGSGGGGGGGGGGYNRSSGGYEPRGRGGGRGGRGGMGGSDRGGFNKFGGPRDQGSRHDSEQDNSDNNTIFVQGLGENVTIES
Splooce       ----------------------------------------------------------------------------------------------------

Uniprot       VADYFKQIGIIKTNKKTGQPMINLYTDRETGKLKGEATVSFDDPPSAKAAIDWFDGKEFSGNPIKVSFATRRADFNRGGGNGRGGRGRGGPMGRGGYGGG
Splooce       --------------------MINLYTDRETGKLKGEATVSFDDPPSAKAAIDWFDGKEFSGNPIKVSFATRRADFNRGGGNGRGGRGRGGPMGRGGYGGG

Uniprot       GSGGGGRGGFPSGGGGGGGQQRAGDWKCPNPTCENMNFSWRNECNQCKAPKPDGPGGGPGGSHMGGNYGDDRRGGRGGYDRGGYRGRGGDRGGFRGGRGG
Splooce       GSGGGGRGGFPSGGGGGGGQQRAGDWKCPNPTCENMNFSWRNECNQCKAPKPDGPGGGPGGSHMGGNYGDDRRGGRGGYDRGGYRGRGGDRGGFRGGRGG

Uniprot       GDRGGFGPGKMDSRGEHRQDRRERPY
Splooce       GDRGGFGPGKMDSRGEHRQDRRERPY

----------------------------------------------------------------------------------------------------

Q16186 (Uniprot)	versus
NM_175573#(-s-s-s-:20_A3920652179332) (Splooce)

For more details about the Alternative Splicing Event -> Link to Splooce page

Peptides that support the ASE (Splooce-specific):
NVPAGPAGGQQVDLASVLTPEIMAPILANADVQER (MAXQUANT)
MNVPAGPAGGQQVDLASVLTPEIMAPILANADVQER (MAXQUANT)

Alignment:
Uniprot       MTTSGALFPSLVPGSRGASNKYLVEFRAGKMSLKGTTVTPDKRKGLVYIQQTDDSLIHFCWKDRTSGNVEDDLIIFPDDCEFKRVPQCPSGRVYVLKFKA
Splooce       ----------------------------------------------------------------------------------------------------

Uniprot       GSKRLFFWMQEPKTDQDEEHCRKVNEYLNNPPMPGALGASGSSGHELSALGGEGGLQSLLGNMSHSQLMQLIGPAGLGGLGGLGALTGPGLASLLGSSGP
Splooce       ----------------------------------------------------------------------------------------------------

Uniprot       PGSSSSSSSRSQSAAVTPSSTTSSTRATPAPSAPAAASATSPSPAPSSGNGASTAASPTQPIQLSDLQSILATMNVPAGPAGGQQVDLASVLTPEIMAPI
Splooce       -------------------------------------------------------------------------MNVPAGPAGGQQVDLASVLTPEIMAPI

Uniprot       LANADVQERLLPYLPSGESLPQTADEIQNTLTSPQFQQALGMFSAALASGQLGPLMCQFGLPAEAVEAANKGDVEAFAKAMQNNAKPEQKEGDTKDKKDE
Splooce       LANADVQERLLPYLPSGESLPQTADEIQNTLTSPQFQQALGMFSAALASGQLGPLMCQFGLPAEAVEAANKGDVEAFAKAMQNNAKPEQKEGDTKDKKDE

Uniprot       EEDMSLD
Splooce       EEDMSLD

----------------------------------------------------------------------------------------------------

Q96G23 (Uniprot)	versus
NM_022075#(-s-s-s-s-s-:1_L1121495190350) (Splooce)

For more details about the Alternative Splicing Event -> Link to Splooce page

Peptides that support the ASE (Splooce-specific):
ALHDSSDYLLESAK (MAXQUANT + PEAKS)

Alignment:
Uniprot       MLQTLYDYFWWERLWLPVNLTWADLEDRDGRVYAKASDLYITLPLALLFLIVRYFFELYVATPLAALLNIKEKTRLRAPPNATLEHFYLTSGKQPKQVEV
Splooce       ----------------------------------------------------------------------------------------------------

Uniprot       ELLSRQSGLSGRQVERWFRRRRNQDRPSLLKKFREASWRFTFYLIAFIAGMAVIVDKPWFYDMKKVWEGYPIQSTIPSQYWYYMIELSFYWSLLFSIASD
Splooce       ----------------------------------------------------------------------------------------------------

Uniprot       VKRKDFKEQIIHHVATIILISFSWFANYIRAGTLIMALHDSSDYLLESAKMFNYAGWKNTCNNIFIVFAIVFIITRLVILPFWILHCTLVYPLELYPAFF
Splooce       -----------------------------------MALHDSSDYLLESAKMFNYAGWKNTCNNIFIVFAIVFIITRLVILPFWILHCTLVYPLELYPAFF

Uniprot       GYYFFNSMMGVLQLLHIFWAYLILRMAHKFITGKLVEDERSDREETESSEGEEAAAGGGAKSRPLANGHPILNNNHRKND
Splooce       GYYFFNSMMGVLQLLHIFWAYLILRMAHKFITGKLVEDERSDREETESSEGEEAAAGGGAKSRPLANGHPILNNNHRKND

----------------------------------------------------------------------------------------------------

P62333 (Uniprot)	versus
NM_002806#(-t:14_P3122860265360) (Splooce)

For more details about the Alternative Splicing Event -> Link to Splooce page

Peptides that support the ASE (Splooce-specific):
SHEDPGNVSYSEIGGLSEQIR (MAXQUANT)
MSHEDPGNVSYSEIGGLSEQIR (MAXQUANT)

Alignment:
Uniprot       MAIPGIPYERRLLIMADPRDKALQDYRKKLLEHKEIDGRLKELREQLKELTKQYEKSENDLKALQSVGQIVGEVLKQLTEEKFIVKATNGPRYVVGCRRQ
Splooce       ----------------------------------------------------------------------------------------------------

Uniprot       LDKSKLKPGTRVALDMTTLTIMRYLPREVDPLVYNMSHEDPGNVSYSEIGGLSEQIRELREVIELPLTNPELFQRVGIIPPKGCLLYGPPGTGKTLLARA
Splooce       -----------------------------------MSHEDPGNVSYSEIGGLSEQIRELREVIELPLTNPELFQRVGIIPPKGCLLYGPPGTGKTLLARA

Uniprot       VASQLDCNFLKVVSSSIVDKYIGESARLIREMFNYARDHQPCIIFMDEIDAIGGRRFSEGTSADREIQRTLMELLNQMDGFDTLHRVKMIMATNRPDTLD
Splooce       VASQLDCNFLKVVSSSIVDKYIGESARLIREMFNYARDHQPCIIFMDEIDAIGGRRFSEGTSADREIQRTLMELLNQMDGFDTLHRVKMIMATNRPDTLD

Uniprot       PALLRPGRLDRKIHIDLPNEQARLDILKIHAGPITKHGEIDYEAIVKLSDGFNGADLRNVCTEAGMFAIRADHDFVVQEDFMKAVRKVADSKKLESKLDY
Splooce       PALLRPGRLDRKIHIDLPNEQARLDILKIHAGPITKHGEIDYEAIVKLSDGFNGADLRNVCTEAGMFAIRADHDFVVQEDFMKAVRKVADSKKLESKLDY

Uniprot       KPV
Splooce       KPV

----------------------------------------------------------------------------------------------------

Q9Y617 (Uniprot)	versus
NM_058179#(-s-s-:9_P8388548550609) (Splooce)

For more details about the Alternative Splicing Event -> Link to Splooce page

Peptides that support the ASE (Splooce-specific):
GLVLEWIK (MAXQUANT)

Alignment:
Uniprot       MDAPRQVVNFGPGPAKLPHSVLLEIQKELLDYKGVGISVLEMSHRSSDFAKIINNTENLVRELLAVPDNYKVIFLQGGGCGQFSAVPLNLIGLKAGRCAD
Splooce       ----------------------------------------------------------------------------------------------------

Uniprot       YVVTGAWSAKAAEEAKKFGTINIVHPKLGSYTKIPDPSTWNLNPDASYVYYCANETVHGVEFDFIPDVKGAVLVCDMSSNFLSKPVDVSKFGVIFAGAQK
Splooce       ----------------------------------------------------------------------------------------------------

Uniprot       NVGSAGVTVVIVRDDLLGFALRECPSVLEYKVQAGNSSLYNTPPCFSIYVMGLVLEWIKNNGGAAAMEKLSSIKSQTIYEIIDNSQGFYVCPVEPQNRSK
Splooce       --------------------------------------------------MGLVLEWIKNNGGAAAMEKLSSIKSQTIYEIIDNSQGFYVCPVEPQNRSK

Uniprot       MNIPFRIGNAKGDDALEKRFLDKALELNMLSLKGHRSVGGIRASLYNAVTIEDVQKLAAFMKKFLEMHQL
Splooce       MNIPFRIGNAKGDDALEKRFLDKALELNMLSLKGHRSVGGIRASLYNAVTIEDVQKLAAFMKKFLEMHQL

----------------------------------------------------------------------------------------------------

Q969X5 (Uniprot)	versus
NM_001031711#(f-:5_E5938528725142) (Splooce)

For more details about the Alternative Splicing Event -> Link to Splooce page

Peptides that support the ASE (Splooce-specific):
MPFEFDIYR (MAXQUANT)

Alignment:
Uniprot       MPFDFRRFDIYRKVPKDLTQPTYTGAIISICCCLFILFLFLSELTGFITTEVVNELYVDDPDKDSGGKIDVSLNISLPNLHCELVGLDIQDEMGRHEVGH
Splooce       MPFEF---DIYRKVPKDLTQPTYTGAIISICCCLFILFLFLSELTGFITTEVVNELYVDDPDKDSGGKIDVSLNISLPNLHCELVGLDIQDEMGRHEVGH

Uniprot       IDNSMKIPLNNGAGCRFEGQFSINKVPGNFHVSTHSATAQPQNPDMTHVIHKLSFGDTLQVQNIHGAFNALGGADRLTSNPLASHDYILKIVPTVYEDKS
Splooce       IDNSMKIPLNNGAGCRFEGQFSINKVPGNFHVSTHSATAQPQNPDMTHVIHKLSFGDTLQVQNIHGAFNALGGADRLTSNPLASHDYILKIVPTVYEDKS

Uniprot       GKQRYSYQYTVANKEYVAYSHTGRIIPAIWFRYDLSPITVKYTERRQPLYRFITTICAIIGGTFTVAGILDSCIFTASEAWKKIQLGKMH
Splooce       GKQRYSYQYTVANKEYVAYSHTGRIIPAIWFRYDLSPITVKYTERRQPLYRFITTICAIIGGTFTVAGILDSCIFTASEAWKKIQLGKMH

----------------------------------------------------------------------------------------------------

P49321 (Uniprot)	versus
NM_002482#(-s-:1_N3709693878410) (Splooce)

For more details about the Alternative Splicing Event -> Link to Splooce page

Peptides that support the ASE (Splooce-specific):
GDIPAAVNAFQEAASLLGK (MAXQUANT)

Alignment:
Uniprot       MAMESTATAAVAAELVSADKIEDVPAPSTSADKVESLDVDSEAKKLLGLGQKHLVMGDIPAAVNAFQEAASLLGKKYGETANECGEAFFFYGKSLLELAR
Splooce       -------------------------------------------------------MGDIPAAVNAFQEAASLLGKKYGETANECGEAFFFYGKSLLELAR

Uniprot       MENGVLGNALEGVHVEEEEGEKTEDESLVENNDNIDEEAREELREQVYDAMGEKEEAKKTEDKSLAKPETDKEQDSEMEKGGREDMDISKSAEEPQEKVD
Splooce       MENGVLGNALEGVHVEEEEGEKTEDESLVENNDNIDEEAREELREQVYDAMGEKEEAKKTEDKSLAKPETDKEQDSEMEKGGREDMDISKSAEEPQEKVD

Uniprot       LTLDWLTETSEEAKGGAAPEGPNEAEVTSGKPEQEVPDAEEEKSVSGTDVQEECREKGGQEKQGEVIVSIEEKPKEVSEEQPVVTLEKQGTAVEVEAESL
Splooce       LTLDWLTETSEEAKGGAAPEGPNEAEVTSGKPEQEVPDAEEEKSVSGTDVQEECREKGGQEKQGEVIVSIEEKPKEVSEEQPVVTLEKQGTAVEVEAESL

Uniprot       DPTVKPVDVGGDEPEEKVVTSENEAGKAVLEQLVGQEVPPAEESPEVTTEAAEASAVEAGSEVSEKPGQEAPVLPKDGAVNGPSVVGDQTPIEPQTSIER
Splooce       DPTVKPVDVGGDEPEEKVVTSENEAGKAVLEQLVGQEVPPAEESPEVTTEAAEASAVEAGSEVSEKPGQEAPVLPKDGAVNGPSVVGDQTPIEPQTSIER

Uniprot       LTETKDGSGLEEKVRAKLVPSQEETKLSVEESEAAGDGVDTKVAQGATEKSPEDKVQIAANEETQEREEQMKEGEETEGSEEDDKENDKTEEMPNDSVLE
Splooce       LTETKDGSGLEEKVRAKLVPSQEETKLSVEESEAAGDGVDTKVAQGATEKSPEDKVQIAANEETQEREEQMKEGEETEGSEEDDKENDKTEEMPNDSVLE

Uniprot       NKSLQENEEEEIGNLELAWDMLDLAKIIFKRQETKEAQLYAAQAHLKLGEVSVESENYVQAVEEFQSCLNLQEQYLEAHDRLLAETHYQLGLAYGYNSQY
Splooce       NKSLQENEEEEIGNLELAWDMLDLAKIIFKRQETKEAQLYAAQAHLKLGEVSVESENYVQAVEEFQSCLNLQEQYLEAHDRLLAETHYQLGLAYGYNSQY

Uniprot       DEAVAQFSKSIEVIENRMAVLNEQVKEAEGSSAEYKKEIEELKELLPEIREKIEDAKESQRSGNVAELALKATLVESSTSGFTPGGGGSSVSMIASRKPT
Splooce       DEAVAQFSKSIEVIENRMAVLNEQVKEAEGSSAEYKKEIEELKELLPEIREKIEDAKESQRSGNVAELALKATLVESSTSGFTPGGGGSSVSMIASRKPT

Uniprot       DGASSSNCVTDISHLVRKKRKPEEESPRKDDAKKAKQEPEVNGGSGDAVPSGNEVSENMEEEAENQAESRAAVEGTVEAGATVESTAC
Splooce       DGASSSNCVTDISHLVRKKRKPEEESPRKDDAKKAKQEPEVNGGSGDAVPSGNEVSENMEEEAENQAESRAAVEGTVEAGATVESTAC

----------------------------------------------------------------------------------------------------

Q8WTP8 (Uniprot)	versus
NM_022767#(f-:15_A9088319600969) (Splooce)

For more details about the Alternative Splicing Event -> Link to Splooce page

Peptides that support the ASE (Splooce-specific):
MWELPAFR (MAXQUANT)

Alignment:
Uniprot       ----------------------------------------------------------------------------------------------------
Splooce       MWELPAFRTPRADRKRHAGLQVRSRTLHRGGGVGRAEAACPGSQVPARCFRPGLRRGRLNWADPALPARRKAGRAPPVSSPRSARGSRARGGGAGRWDSA

Uniprot       ------------------------------------MVPREAPESAQCLCPSLTIPNAKDVLRKRHKRRSRQHQRFMARKALLQEQGLLSMPPEPGSSPL
Splooce       QLPRPASDQRPSPSRLLPHWKITPQASLAPSSELTGMVPREAPESAQCLCPSLTIPNAKDVLRKRHKRRSRQHQRFMARKALLQEQGLLSMPPEPGSSPL

Uniprot       PTPFGAATATEAASSGKQCLRAGSGSAPCSRRPAPGKASGPLPSKCVAIDCEMVGTGPRGRVSELARCSIVSYHGNVLYDKYIRPEMPIADYRTRWSGIT
Splooce       PTPFGAATATEAASSGKQCLRAGSGSAPCSRRPAPGKASGPLPSKCVAIDCEMVGTGPRGRVSELARCSIVSYHGNVLYDKYIRPEMPIADYRTRWSGIT

Uniprot       RQHMRKAVPFQVAQKEILKLLKGKVVVGHALHNDFQALKYVHPRSQTRDTTYVPNFLSEPGLHTRARVSLKDLALQLLHKKIQVGQHGHSSVEDATTAME
Splooce       RQHMRKAVPFQVAQKEILKLLKGKVVVGHALHNDFQALKYVHPRSQTRDTTYVPNFLSEPGLHTRARVSLKDLALQLLHKKIQVGQHGHSSVEDATTAME

Uniprot       LYRLVEVQWEQQEARSLWTCPEDREPDSSTDMEQYMEDQYWPDDLAHGSRGGAREAQDRRN
Splooce       LYRLVEVQWEQQEARSLWTCPEDREPDSSTDMEQYMEDQYWPDDLAHGSRGGAREAQDRRN

----------------------------------------------------------------------------------------------------

F8W904 (Uniprot)	versus
NM_001316#(-t:20_C390301552880) (Splooce)

For more details about the Alternative Splicing Event -> Link to Splooce page

Peptides that support the ASE (Splooce-specific):
LSSPEQIQK (MAXQUANT)
MLSSPEQIQK (MAXQUANT)
MLSSPEQIQKQLSDAISIIGR (MAXQUANT)
LSSPEQLQK (PEAKS)

Alignment:
Uniprot       MELSDANLQTLTEYLKKTLDPDPAIRRPAEKFLESVEGNQNYPLLLLTLLEKSQDNVIKVCASVTFKNYIKRNWRIVEDEPNKICEADRVAIKANIVHLM
Splooce       ---------------------------------------------------------------------------------------------------M

Uniprot       LSSPEQIQKQLSDAISIIGREDFPQKWPDLLTEMVNRFQSGDFHVINGVLRTAHSLFKRYRHEFKSNELWTEIKLVLDAFALPLTNLFKATIELCSTHAN
Splooce       LSSPEQIQKQLSDAISIIGREDFPQKWPDLLTEMVNRFQSGDFHVINGVLRTAHSLFKRYRHEFKSNELWTEIKLVLDAFALPLTNLFKATIELCSTHAN

Uniprot       DASALRILFSSLILISKLFYSLNFQDLPEFFEDNMETWMNNFHTLLTLDNKLLQTDDEEEAGLLELLKSQICDNAALYAQKYDEEFQRYLPRFVTAIWNL
Splooce       DASALRILFSSLILISKLFYSLNFQDLPEFFEDNMETWMNNFHTLLTLDNKLLQTDDEEEAGLLELLKSQICDNAALYAQKYDEEFQRYLPRFVTAIWNL

Uniprot       LVTTGQEVKYDLLVSNAIQFLASVCERPHYKNLFEDQNTLTSICEKVIVPNMEFRAADEEAFEDNSEEYIRRDLEGSDIDTRRRAACDLVRGLCKFFEGP
Splooce       LVTTGQEVKYDLLVSNAIQFLASVCERPHYKNLFEDQNTLTSICEKVIVPNMEFRAADEEAFEDNSEEYIRRDLEGSDIDTRRRAACDLVRGLCKFFEGP

Uniprot       VTGIFSGYVNSMLQEYAKNPSVNWKHKDAAIYLVTSLASKAQTQKHGITQANELVNLTEFFVNHILPDLKSANVNEFPVLKADGIKYIMIFRNQVPKEHL
Splooce       VTGIFSGYVNSMLQEYAKNPSVNWKHKDAAIYLVTSLASKAQTQKHGITQANELVNLTEFFVNHILPDLKSANVNEFPVLKADGIKYIMIFRNQVPKEHL

Uniprot       LVSIPLLINHLQAESIVVHTYAAHALERLFTMRGPNNATLFTAAEIAPFVEILLTNLFKALTLPGSSENEYIMKAIMRSFSLLQEAIIPYIPTLITQLTQ
Splooce       LVSIPLLINHLQAESIVVHTYAAHALERLFTMRGPNNATLFTAAEIAPFVEILLTNLFKALTLPGSSENEYIMKAIMRSFSLLQEAIIPYIPTLITQLTQ

Uniprot       KLLAVSKNPSKPHFNHYMFEAICLSIRITCKANPAAVVNFEEALFLVFTEILQNDVQEFIPYVFQVMSLLLETHKNDIPSSYMALFPHLLQPVLWERTGN
Splooce       KLLAVSKNPSKPHFNHYMFEAICLSIRITCKANPAAVVNFEEALFLVFTEILQNDVQEFIPYVFQVMSLLLETHKNDIPSSYMALFPHLLQPVLWERTGN

Uniprot       IPALVRLLQAFLERGSNTIASAAADKIPGLLGVFQKLIASKANDHQGFYLLNSIIEHMPPESVDQYRKQIFILLFQRLQNSKTTKFIKSFLVFINLYCIK
Splooce       IPALVRLLQAFLERGSNTIASAAADKIPGLLGVFQKLIASKANDHQGFYLLNSIIEHMPPESVDQYRKQIFILLFQRLQNSKTTKFIKSFLVFINLYCIK

Uniprot       YGALALQEIFDGIQPKMFGMVLEKIIIPEIQKVSGNVEKKICAVGITKLLTECPPMMDTEYTKLWTPLLQSLIGLFELPEDDTIPDEEHFIDIEDTPGYQ
Splooce       YGALALQEIFDGIQPKMFGMVLEKIIIPEIQKVSGNVEKKICAVGITKLLTECPPMMDTEYTKLWTPLLQSLIGLFELPEDDTIPDEEHFIDIEDTPGYQ

Uniprot       TAFSQLAFAGKKEHDPVGQMVNNPKIHLAQSLHKLSTACPGRVPSMVSTSLNAEALQYLQGYLQAASVTLL
Splooce       TAFSQLAFAGKKEHDPVGQMVNNPKIHLAQSLHKLSTACPGRVPSMVSTSLNAEALQYLQGYLQAASVTLL

----------------------------------------------------------------------------------------------------

P55160 (Uniprot)	versus
NM_005337#(-s-s-s-:12_N5537146585896) (Splooce)

For more details about the Alternative Splicing Event -> Link to Splooce page

Peptides that support the ASE (Splooce-specific):
LLCSVTSFSFHLR (MAXQUANT)

Alignment:
Uniprot       MSLTSAYQHKLAEKLTILNDRGQGVLIRMYNIKKTCSDPKSKPPFLLEKSMEPSLKYINKKFPNIDVRNSTQHLGPVHREKAEIIRFLTNYYQSFVDVME
Splooce       ----------------------------------------------------------------------------------------------------

Uniprot       FRDHVYELLNTIDACQCHFDINLNFDFTRSYLDLIVTYTSVILLLSRIEDRRILIGMYNCAHEMLHGHGDPSFARLGQMVLEYDHPLKKLTEEFGPHTKA
Splooce       ----------------------------------------------------------------------------------------------------

Uniprot       VSGALLSLHFLFVRRNQGAEQWRSAQLLSLISNPPAMINPANSDTMACEYLSVEVMERWIIIGFLLCHGCLNSNSQCQKLWKLCLQGSLYITLIREDVLQ
Splooce       ----------------------------------------------------------------------------------------------------

Uniprot       VHKVTEDLFSSLKGYGKRVADIKESKEHVIANSGQFHCQRRQFLRMAVKELETVLADEPGLLGPKALFAFMALSFIRDEVTWLVRHTENVTKTKTPEDYA
Splooce       ----------------------------------------------------------------------------------------------------

Uniprot       DSSIAELLFLLEGIRSLVRRHIKVIQQYHLQYLARFDALVLSDIIQNLSVCPEEESIIMSSFVSILSSLNLKQVDNGEKFEFSGLRLDWFRLQAYTSVAK
Splooce       ----------------------------------------------------------------------------------------------------

Uniprot       APLHLHENPDLAKVMNLIVFHSRMLDSVEKLLVETSDLSTFCFHLRIFEKMFAMTLEESAMLRYAIAFPLICAHFVHCTHEMCPEEYPHLKNHGLHHCNS
Splooce       --------------------------------MLLCSVTSFSFHLRIFEKMFAMTLEESAMLRYAIAFPLICAHFVHCTHEMCPEEYPHLKNHGLHHCNS

Uniprot       FLEELAKQTSNCVLEICAEQRNLSEQLLPKHCATTISKAKNKKTRKQRQTPRKGEPERDKPGAESHRKNRSIVTNMDKLHLNLTELALTMNHVYSFSVFE
Splooce       FLEELAKQTSNCVLEICAEQRNLSEQLLPKHCATTISKAKNKKTRKQRQTPRKGEPERDKPGAESHRKNRSIVTNMDKLHLNLTELALTMNHVYSFSVFE

Uniprot       HTIFPSEYLSSHLEARLNRAIVWLAGYNATTQEIVRPSELLAGVKAYIGFIQSLAQFLGADASRVIRNALLQQTQPLDSCGEQTITTLYTNWYLESLLRQ
Splooce       HTIFPSEYLSSHLEARLNRAIVWLAGYNATTQEIVRPSELLAGVKAYIGFIQSLAQFLGADASRVIRNALLQQTQPLDSCGEQTITTLYTNWYLESLLRQ

Uniprot       ASSGTIILSPAMQAFVSLPREGEQNFSAEEFSDISEMRALAELLGPYGMKFLSENLMWHVTSQIVELKKLVVENMDILVQIRSNFSKPDLMASLLPQLTG
Splooce       ASSGTIILSPAMQAFVSLPREGEQNFSAEEFSDISEMRALAELLGPYGMKFLSENLMWHVTSQIVELKKLVVENMDILVQIRSNFSKPDLMASLLPQLTG

Uniprot       AENVLKRMTIIGVILSFRAMAQEGLREVFSSHCPFLMGPIECLKEFVTPDTDIKVTLSIFELASAAGVGCDIDPALVAAIANLKADTSSPEEEYKVACLL
Splooce       AENVLKRMTIIGVILSFRAMAQEGLREVFSSHCPFLMGPIECLKEFVTPDTDIKVTLSIFELASAAGVGCDIDPALVAAIANLKADTSSPEEEYKVACLL

Uniprot       LIFLAVSLPLLATDPSSFYSIEKDGYNNNIHCLTKAIIQVSAALFTLYNKNIETHLKEFLVVASVSLLQLGQETDKLKTRNRESISLLMRLVVEESSFLT
Splooce       LIFLAVSLPLLATDPSSFYSIEKDGYNNNIHCLTKAIIQVSAALFTLYNKNIETHLKEFLVVASVSLLQLGQETDKLKTRNRESISLLMRLVVEESSFLT

Uniprot       LDMLESCFPYVLLRNAYREVSRAFHLN
Splooce       LDMLESCFPYVLLRNAYREVSRAFHLN

----------------------------------------------------------------------------------------------------

O14787 (Uniprot)	versus
NM_013433#(-s-s-s-s-s-s-s-s-s-s-:19_T3882692944185) (Splooce)

For more details about the Alternative Splicing Event -> Link to Splooce page

Peptides that support the ASE (Splooce-specific):
MYTQHPEQYEAPDK (MAXQUANT)

Alignment:
Uniprot       MDWQPDEQGLQQVLQLLKDSQSPNTATQRIVQDKLKQLNQFPDFNNYLIFVLTRLKSEDEPTRSLSGLILKNNVKAHYQSFPPPVADFIKQECLNNIGDA
Splooce       ----------------------------------------------------------------------------------------------------

Uniprot       SSLIRATIGILITTIASKGELQMWPELLPQLCNLLNSEDYNTCEGAFGALQKICEDSSELLDSDALNRPLNIMIPKFLQFFKHCSPKIRSHAIACVNQFI
Splooce       ----------------------------------------------------------------------------------------------------

Uniprot       MDRAQALMDNIDTFIEHLFALAVDDDPEVRKNVCRALVMLLEVRIDRLIPHMHSIIQYMLQRTQDHDENVALEACEFWLTLAEQPICKEVLASHLVQLIP
Splooce       ----------------------------------------------------------------------------------------------------

Uniprot       ILVNGMKYSEIDIILLKGDVEEDEAVPDSEQDIKPRFHKSRTVTLPHEAERPDGSEDAEDDDDDDALSDWNLRKCSAAALDVLANVFREELLPHLLPLLK
Splooce       ----------------------------------------------------------------------------------------------------

Uniprot       GLLFHPEWVVKESGILVLGAIAEGCMQGMVPYLPELIPHLIQCLSDKKALVRSIACWTLSRYAHWVVSQPPDMHLKPLMTELLKRILDGNKRVQEAACSA
Splooce       ----------------------------------------------------------------------------------------------------

Uniprot       FATLEEEACTELVPYLSYILDTLVFAFGKYQHKNLLILYDAIGTLADSVGHHLNQPEYIQKLMPPLIQKWNELKDEDKDLFPLLECLSSVATALQSGFLP
Splooce       ----------------------------------------------------------------------------------------------------

Uniprot       YCEPVYQRCVTLVQKTLAQAMMYTQHPEQYEAPDKDFMIVALDLLSGLAEGLGGHVEQLVARSNIMTLLFQCMQDSMPEVRQSSFALLGDLTKACFIHVK
Splooce       ---------------------MYTQHPEQYEAPDKDFMIVALDLLSGLAEGLGGHVEQLVARSNIMTLLFQCMQDSMPEVRQSSFALLGDLTKACFIHVK

Uniprot       PCIAEFMPILGTNLNPEFISVCNNATWAIGEICMQMGAEMQPYVQMVLNNLVEIINRPNTPKTLLENTAITIGRLGYVCPQEVAPMLQQFIRPWCTSLRN
Splooce       PCIAEFMPILGTNLNPEFISVCNNATWAIGEICMQMGAEMQPYVQMVLNNLVEIINRPNTPKTLLENTAITIGRLGYVCPQEVAPMLQQFIRPWCTSLRN

Uniprot       IRDNEEKDSAFRGICMMIGVNPGGVVQDFIFFCDAVASWVSPKDDLRDMFYKILHGFKDQVGEDNWQQFSEQFPPLLKERLAAFYGV
Splooce       IRDNEEKDSAFRGICMMIGVNPGGVVQDFIFFCDAVASWVSPKDDLRDMFYKILHGFKDQVGEDNWQQFSEQFPPLLKERLAAFYGV

----------------------------------------------------------------------------------------------------

P40616 (Uniprot)	versus
NM_001177#(-s-:12_A2530858888324) (Splooce)

For more details about the Alternative Splicing Event -> Link to Splooce page

Peptides that support the ASE (Splooce-specific):
LEEEELR (MAXQUANT)
LEEEELRK (MAXQUANT)
MLEEEELR (MAXQUANT)

Alignment:
Uniprot       MGGFFSSIFSSLFGTREMRILILGLDGAGKTTILYRLQVGEVVTTIPTIGFNVETVTYKNLKFQVWDLGGQTSIRPYWRCYYSNTDAVIYVVDSCDRDRI
Splooce       ----------------------------------------------------------------------------------------------------

Uniprot       GISKSELVAMLEEEELRKAILVVFANKQDMEQAMTSSEMANSLGLPALKDRKWQIFKTSATKGTGLDEAMEWLVETLKSRQ
Splooce       ---------MLEEEELRKAILVVFANKQDMEQAMTSSEMANSLGLPALKDRKWQIFKTSATKGTGLDEAMEWLVETLKSRQ

----------------------------------------------------------------------------------------------------

Q99436 (Uniprot)	versus
NM_002799#(f-:9_P8383605527344) (Splooce)

For more details about the Alternative Splicing Event -> Link to Splooce page

Peptides that support the ASE (Splooce-specific):
TTQLISSNLELHSLSTGR (MAXQUANT)
MTTQLISSNLELHSLSTGR (MAXQUANT)

Alignment:
Uniprot       MAAVSVYAPPVGGFSFDNCRRNAVLEADFAKRGYKLPKVRKTGTTIAGVVYKDGIVLGADTRATEGMVVADKNCSKIHFISPNIYCCGAGTAADTDMTTQ
Splooce       ------------------------------------------------------------------------------------------------MTTQ

Uniprot       LISSNLELHSLSTGRLPRVVTANRMLKQMLFRYQGYIGAALVLGGVDVTGPHLYSIYPHGSTDKLPYVTMGSGSLAAMAVFEDKFRPDMEEEEAKNLVSE
Splooce       LISSNLELHSLSTGRLPRVVTANRMLKQMLFRYQGYIGAALVLGGVDVTGPHLYSIYPHGSTDKLPYVTMGSGSLAAMAVFEDKFRPDMEEEEAKNLVSE

Uniprot       AIAAGIFNDLGSGSNIDLCVISKNKLDFLRPYTVPNKKGTRLGRYRCEKGTTAVLTEKITPLEIEVLEETVQTMDTS
Splooce       AIAAGIFNDLGSGSNIDLCVISKNKLDFLRPYTVPNKKGTRLGRYRCEKGTTAVLTEKITPLEIEVLEETVQTMDTS

----------------------------------------------------------------------------------------------------

O95372 (Uniprot)	versus
NM_007260#(r:1_L6970224377555) (Splooce)

For more details about the Alternative Splicing Event -> Link to Splooce page

Peptides that support the ASE (Splooce-specific):
GLSPDAPEDEAGIK (MAXQUANT)

Alignment:
Uniprot       MCGNTMSVPLLTDAATVSGAERETAAVIFLHGLGDTGHSWADALSTIRLPHVKYICPHAPRIPVTLNMKMVMPSWFDLMGLSPDAPEDEAGIKKAAENIK
Splooce       ------------------------------------------------------------------------------MGLSPDAPEDEAGIKKAAENIK

Uniprot       ALIEHEMKNGIPANRIVLGGFSQGGALSLYTALTCPHPLAGIVALSCWLPLHRAFPQAANGSAKDLAILQCHGELDPMVPVRFGALTAEKLRSVVTPARV
Splooce       ALIEHEMKNGIPANRIVLGGFSQGGALSLYTALTCPHPLAGIVALSCWLPLHRAFPQAANGSAKDLAILQCHGELDPMVPVRFGALTAEKLRSVVTPARV

Uniprot       QFKTYPGVMHSSCPQEMAAVKEFLEKLLPPV
Splooce       QFKTYPGVMHSSCPQEMAAVKEFLEKLLPPV

----------------------------------------------------------------------------------------------------

P07602 (Uniprot)	versus
NM_002778#(f-:10_P8707747282317) (Splooce)

For more details about the Alternative Splicing Event -> Link to Splooce page

Peptides that support the ASE (Splooce-specific):
MVTDIQTAVR (MAXQUANT)

Alignment:
Uniprot       MYALFLLASLLGAALAGPVLGLKECTRGSAVWCQNVKTASDCGAVKHCLQTVWNKPTVKSLPCDICKDVVTAAGDMLKDNATEEEILVYLEKTCDWLPKP
Splooce       ----------------------------------------------------------------------------------------------------

Uniprot       NMSASCKEIVDSYLPVILDIIKGEMSRPGEVCSALNLCESLQKHLAELNHQKQLESNKIPELDMTEVVAPFMANIPLLLYPQDGPRSKPQPKDNGDVCQD
Splooce       ----------------------------------------------------------------------------------------------------

Uniprot       CIQMVTDIQTAVRTNSTFVQALVEHVKEECDRLGPGMADICKNYISQYSEIAIQMMMHMQPKEICALVGFCDEVKEMPMQTLVPAKVASKNVIPALELVE
Splooce       ---MVTDIQTAVRTNSTFVQALVEHVKEECDRLGPGMADICKNYISQYSEIAIQMMMHMQPKEICALVGFCDEVKEMPMQTLVPAKVASKNVIPALELVE

Uniprot       PIKKHEVPAKSDVYCEVCEFLVKEVTKLIDNNKTEKEILDAFDKMCSKLPKSLSEECQEVVDTYGSSILSILLEEVSPELVCSMLHLCSGTRLPALTVHV
Splooce       PIKKHEVPAKSDVYCEVCEFLVKEVTKLIDNNKTEKEILDAFDKMCSKLPKSLSEECQEVVDTYGSSILSILLEEVSPELVCSMLHLCSGTRLPALTVHV

Uniprot       TQPKDGGFCEVCKKLVGYLDRNLEKNSTKQEILAALEKGCSFLPDPYQKQCDQFVAEYEPVLIEILVEVMDPSFVCLKIGACPSAHKPLLGTEKCIWGPS
Splooce       TQPKDGGFCEVCKKLVGYLDRNLEKNSTKQEILAALEKGCSFLPDPYQKQCDQFVAEYEPVLIEILVEVMDPSFVCLKIGACPSAHKPLLGTEKCIWGPS

Uniprot       YWCQNTETAAQCNAVEHCKRHVWN
Splooce       YWCQNTETAAQCNAVEHCKRHVWN

----------------------------------------------------------------------------------------------------

P28288 (Uniprot)	versus
NM_002858#(-s-s-s-s-s-s-s-s-s-:1_A6841548938277) (Splooce)

For more details about the Alternative Splicing Event -> Link to Splooce page

Peptides that support the ASE (Splooce-specific):
MTLGTLR (MAXQUANT + PEAKS)

Alignment:
Uniprot       MAAFSKYLTARNSSLAGAAFLLLCLLHKRRRALGLHGKKSGKPPLQNNEKEGKKERAVVDKVFFSRLIQILKIMVPRTFCKETGYLVLIAVMLVSRTYCD
Splooce       ----------------------------------------------------------------------------------------------------

Uniprot       VWMIQNGTLIESGIIGRSRKDFKRYLLNFIAAMPLISLVNNFLKYGLNELKLCFRVRLTKYLYEEYLQAFTYYKMGNLDNRIANPDQLLTQDVEKFCNSV
Splooce       ----------------------------------------------------------------------------------------------------

Uniprot       VDLYSNLSKPFLDIVLYIFKLTSAIGAQGPASMMAYLVVSGLFLTRLRRPIGKMTITEQKYEGEYRYVNSRLITNSEEIAFYNGNKREKQTVHSVFRKLV
Splooce       ----------------------------------------------------------------------------------------------------

Uniprot       EHLHNFILFRFSMGFIDSIIAKYLATVVGYLVVSRPFLDLSHPRHLKSTHSELLEDYYQSGRMLLRMSQALGRIVLAGREMTRLAGFTARITELMQVLKD
Splooce       ----------------------------------------------------------------------------------------------------

Uniprot       LNHGKYERTMVSQQEKGIEGVQVIPLIPGAGEIIIADNIIKFDHVPLATPNGDVLIRDLNFEVRSGANVLICGPNGCGKSSLFRVLGELWPLFGGRLTKP
Splooce       ----------------------------------------------------------------------------------------------------

Uniprot       ERGKLFYVPQRPYMTLGTLRDQVIYPDGREDQKRKGISDLVLKEYLDNVQLGHILEREGGWDSVQDWMDVLSGGEKQRMAMARLFYHKPQFAILDECTSA
Splooce       -------------MTLGTLRDQVIYPDGREDQKRKGISDLVLKEYLDNVQLGHILEREGGWDSVQDWMDVLSGGEKQRMAMARLFYHKPQFAILDECTSA

Uniprot       VSVDVEGYIYSHCRKVGITLFTVSHRKSLWKHHEYYLHMDGRGNYEFKQITEDTVEFGS
Splooce       VSVDVEGYIYSHCRKVGITLFTVSHRKSLWKHHEYYLHMDGRGNYEFKQITEDTVEFGS

----------------------------------------------------------------------------------------------------

Q9UI10 (Uniprot)	versus
NM_172195#(-s-:2_E9418095079851) (Splooce)

For more details about the Alternative Splicing Event -> Link to Splooce page

Peptides that support the ASE (Splooce-specific):
MSIPSSVIHPAMVR (MAXQUANT)

Alignment:
Uniprot       MPTQQPAAPSTRAPKPSRSLSGSLCALFSDADSGSGMKAELPPGPGAVGREMTKEEKLQLRKEKKQQKKKRKEEKGAEPETGSAVSAAQCQGPTRELPES
Splooce       ----------------------------------------------------------------------------------------------------

Uniprot       GIQLGTPREKVPAGRSKAELRAERRAKQEAERALKQARKGEQGGPPPKASPSTAGETPSGVKRLPEYPQVDDLLLRRLVKKPERQQVPTRKDYGSKVSLF
Splooce       ----------------------------------------------------------------------------------------------------

Uniprot       SHLPQYSRQNSLTQFMSIPSSVIHPAMVRLGLQYSQGLVSGSNARCIALLRALQQVIQDYTTPPNEELSRDLVNKLKPYMSFLTQCRPLSASMHNAIKFL
Splooce       ---------------MSIPSSVIHPAMVRLGLQYSQGLVSGSNARCIALLRALQQVIQDYTTPPNEELSRDLVNKLKPYMSFLTQCRPLSASMHNAIKFL

Uniprot       NKEITSVGSSKREEEAKSELRAAIDRYVQEKIVLAAQAISRFAYQKISNGDVILVYGCSSLVSRILQEAWTEGRRFRVVVVDSRPWLEGRHTLRSLVHAG
Splooce       NKEITSVGSSKREEEAKSELRAAIDRYVQEKIVLAAQAISRFAYQKISNGDVILVYGCSSLVSRILQEAWTEGRRFRVVVVDSRPWLEGRHTLRSLVHAG

Uniprot       VPASYLLIPAASYVLPEVSKVLLGAHALLANGSVMSRVGTAQLALVARAHNVPVLVCCETYKFCERVQTDAFVSNELDDPDDLQCKRGEHVALANWQNHA
Splooce       VPASYLLIPAASYVLPEVSKVLLGAHALLANGSVMSRVGTAQLALVARAHNVPVLVCCETYKFCERVQTDAFVSNELDDPDDLQCKRGEHVALANWQNHA

Uniprot       SLRLLNLVYDVTPPELVDLVITELGMIPCSSVPVVLRVKSSDQ
Splooce       SLRLLNLVYDVTPPELVDLVITELGMIPCSSVPVVLRVKSSDQ

----------------------------------------------------------------------------------------------------

P35579 (Uniprot)	versus
NM_002473#(-s-s-s-s-s-s-s-s-s-s-:22_M6769030376743) (Splooce)

For more details about the Alternative Splicing Event -> Link to Splooce page

Peptides that support the ASE (Splooce-specific):
SETALPGAFK (MAXQUANT)

Alignment:
Uniprot       MAQQAADKYLYVDKNFINNPLAQADWAAKKLVWVPSDKSGFEPASLKEEVGEEAIVELVENGKKVKVNKDDIQKMNPPKFSKVEDMAELTCLNEASVLHN
Splooce       ----------------------------------------------------------------------------------------------------

Uniprot       LKERYYSGLIYTYSGLFCVVINPYKNLPIYSEEIVEMYKGKKRHEMPPHIYAITDTAYRSMMQDREDQSILCTGESGAGKTENTKKVIQYLAYVASSHKS
Splooce       ----------------------------------------------------------------------------------------------------

Uniprot       KKDQGELERQLLQANPILEAFGNAKTVKNDNSSRFGKFIRINFDVNGYIVGANIETYLLEKSRAIRQAKEERTFHIFYYLLSGAGEHLKTDLLLEPYNKY
Splooce       ----------------------------------------------------------------------------------------------------

Uniprot       RFLSNGHVTIPGQQDKDMFQETMEAMRIMGIPEEEQMGLLRVISGVLQLGNIVFKKERNTDQASMPDNTAAQKVSHLLGINVTDFTRGILTPRIKVGRDY
Splooce       ----------------------------------------------------------------------------------------------------

Uniprot       VQKAQTKEQADFAIEALAKATYERMFRWLVLRINKALDKTKRQGASFIGILDIAGFEIFDLNSFEQLCINYTNEKLQQLFNHTMFILEQEEYQREGIEWN
Splooce       ----------------------------------------------------------------------------------------------------

Uniprot       FIDFGLDLQPCIDLIEKPAGPPGILALLDEECWFPKATDKSFVEKVMQEQGTHPKFQKPKQLKDKADFCIIHYAGKVDYKADEWLMKNMDPLNDNIATLL
Splooce       ----------------------------------------------------------------------------------------------------

Uniprot       HQSSDKFVSELWKDVDRIIGLDQVAGMSETALPGAFKTRKGMFRTVGQLYKEQLAKLMATLRNTNPNFVRCIIPNHEKKAGKLDPHLVLDQLRCNGVLEG
Splooce       --------------------------MSETALPGAFKTRKGMFRTVGQLYKEQLAKLMATLRNTNPNFVRCIIPNHEKKAGKLDPHLVLDQLRCNGVLEG

Uniprot       IRICRQGFPNRVVFQEFRQRYEILTPNSIPKGFMDGKQACVLMIKALELDSNLYRIGQSKVFFRAGVLAHLEEERDLKITDVIIGFQACCRGYLARKAFA
Splooce       IRICRQGFPNRVVFQEFRQRYEILTPNSIPKGFMDGKQACVLMIKALELDSNLYRIGQSKVFFRAGVLAHLEEERDLKITDVIIGFQACCRGYLARKAFA

Uniprot       KRQQQLTAMKVLQRNCAAYLKLRNWQWWRLFTKVKPLLQVSRQEEEMMAKEEELVKVREKQLAAENRLTEMETLQSQLMAEKLQLQEQLQAETELCAEAE
Splooce       KRQQQLTAMKVLQRNCAAYLKLRNWQWWRLFTKVKPLLQVSRQEEEMMAKEEELVKVREKQLAAENRLTEMETLQSQLMAEKLQLQEQLQAETELCAEAE

Uniprot       ELRARLTAKKQELEEICHDLEARVEEEEERCQHLQAEKKKMQQNIQELEEQLEEEESARQKLQLEKVTTEAKLKKLEEEQIILEDQNCKLAKEKKLLEDR
Splooce       ELRARLTAKKQELEEICHDLEARVEEEEERCQHLQAEKKKMQQNIQELEEQLEEEESARQKLQLEKVTTEAKLKKLEEEQIILEDQNCKLAKEKKLLEDR

Uniprot       IAEFTTNLTEEEEKSKSLAKLKNKHEAMITDLEERLRREEKQRQELEKTRRKLEGDSTDLSDQIAELQAQIAELKMQLAKKEEELQAALARVEEEAAQKN
Splooce       IAEFTTNLTEEEEKSKSLAKLKNKHEAMITDLEERLRREEKQRQELEKTRRKLEGDSTDLSDQIAELQAQIAELKMQLAKKEEELQAALARVEEEAAQKN

Uniprot       MALKKIRELESQISELQEDLESERASRNKAEKQKRDLGEELEALKTELEDTLDSTAAQQELRSKREQEVNILKKTLEEEAKTHEAQIQEMRQKHSQAVEE
Splooce       MALKKIRELESQISELQEDLESERASRNKAEKQKRDLGEELEALKTELEDTLDSTAAQQELRSKREQEVNILKKTLEEEAKTHEAQIQEMRQKHSQAVEE

Uniprot       LAEQLEQTKRVKANLEKAKQTLENERGELANEVKVLLQGKGDSEHKRKKVEAQLQELQVKFNEGERVRTELADKVTKLQVELDNVTGLLSQSDSKSSKLT
Splooce       LAEQLEQTKRVKANLEKAKQTLENERGELANEVKVLLQGKGDSEHKRKKVEAQLQELQVKFNEGERVRTELADKVTKLQVELDNVTGLLSQSDSKSSKLT

Uniprot       KDFSALESQLQDTQELLQEENRQKLSLSTKLKQVEDEKNSFREQLEEEEEAKHNLEKQIATLHAQVADMKKKMEDSVGCLETAEEVKRKLQKDLEGLSQR
Splooce       KDFSALESQLQDTQELLQEENRQKLSLSTKLKQVEDEKNSFREQLEEEEEAKHNLEKQIATLHAQVADMKKKMEDSVGCLETAEEVKRKLQKDLEGLSQR

Uniprot       HEEKVAAYDKLEKTKTRLQQELDDLLVDLDHQRQSACNLEKKQKKFDQLLAEEKTISAKYAEERDRAEAEAREKETKALSLARALEEAMEQKAELERLNK
Splooce       HEEKVAAYDKLEKTKTRLQQELDDLLVDLDHQRQSACNLEKKQKKFDQLLAEEKTISAKYAEERDRAEAEAREKETKALSLARALEEAMEQKAELERLNK

Uniprot       QFRTEMEDLMSSKDDVGKSVHELEKSKRALEQQVEEMKTQLEELEDELQATEDAKLRLEVNLQAMKAQFERDLQGRDEQSEEKKKQLVRQVREMEAELED
Splooce       QFRTEMEDLMSSKDDVGKSVHELEKSKRALEQQVEEMKTQLEELEDELQATEDAKLRLEVNLQAMKAQFERDLQGRDEQSEEKKKQLVRQVREMEAELED

Uniprot       ERKQRSMAVAARKKLEMDLKDLEAHIDSANKNRDEAIKQLRKLQAQMKDCMRELDDTRASREEILAQAKENEKKLKSMEAEMIQLQEELAAAERAKRQAQ
Splooce       ERKQRSMAVAARKKLEMDLKDLEAHIDSANKNRDEAIKQLRKLQAQMKDCMRELDDTRASREEILAQAKENEKKLKSMEAEMIQLQEELAAAERAKRQAQ

Uniprot       QERDELADEIANSSGKGALALEEKRRLEARIAQLEEELEEEQGNTELINDRLKKANLQIDQINTDLNLERSHAQKNENARQQLERQNKELKVKLQEMEGT
Splooce       QERDELADEIANSSGKGALALEEKRRLEARIAQLEEELEEEQGNTELINDRLKKANLQIDQINTDLNLERSHAQKNENARQQLERQNKELKVKLQEMEGT

Uniprot       VKSKYKASITALEAKIAQLEEQLDNETKERQAACKQVRRTEKKLKDVLLQVDDERRNAEQYKDQADKASTRLKQLKRQLEEAEEEAQRANASRRKLQREL
Splooce       VKSKYKASITALEAKIAQLEEQLDNETKERQAACKQVRRTEKKLKDVLLQVDDERRNAEQYKDQADKASTRLKQLKRQLEEAEEEAQRANASRRKLQREL

Uniprot       EDATETADAMNREVSSLKNKLRRGDLPFVVPRRMARKGAGDGSDEEVDGKADGAEAKPAE
Splooce       EDATETADAMNREVSSLKNKLRRGDLPFVVPRRMARKGAGDGSDEEVDGKADGAEAKPAE

----------------------------------------------------------------------------------------------------

P30046 (Uniprot)	versus
NM_001355#(r:22_D8038632565639) (Splooce)

For more details about the Alternative Splicing Event -> Link to Splooce page

Peptides that support the ASE (Splooce-specific):
ALSGSTEPCAQLSISSIGVVGTAEDNR (MAXQUANT)

Alignment:
Uniprot       MPFLELDTNLPANRVPAGLEKRLCAAAASILGKPADRVNVTVRPGLAMALSGSTEPCAQLSISSIGVVGTAEDNRSHSAHFFEFLTKELALGQDRILIRF
Splooce       -----------------------------------------------MALSGSTEPCAQLSISSIGVVGTAEDNRSHSAHFFEFLTKELALGQDRILIRF

Uniprot       FPLESWQIGKIGTVMTFL
Splooce       FPLESWQIGKIGTVMTFL

----------------------------------------------------------------------------------------------------

Q13838 (Uniprot)	versus
NM_004640#(r:6_B5172618510246) (Splooce)

For more details about the Alternative Splicing Event -> Link to Splooce page

Peptides that support the ASE (Splooce-specific):
DVLCQAK (MAXQUANT + PEAKS)

Alignment:
Uniprot       MAENDVDNELLDYEDDEVETAAGGDGAEAPAKKDVKGSYVSIHSSGFRDFLLKPELLRAIVDCGFEHPSEVQHECIPQAILGMDVLCQAKSGMGKTAVFV
Splooce       ----------------------------------------------------------------------------------MDVLCQAKSGMGKTAVFV

Uniprot       LATLQQLEPVTGQVSVLVMCHTRELAFQISKEYERFSKYMPNVKVAVFFGGLSIKKDEEVLKKNCPHIVVGTPGRILALARNKSLNLKHIKHFILDECDK
Splooce       LATLQQLEPVTGQVSVLVMCHTRELAFQISKEYERFSKYMPNVKVAVFFGGLSIKKDEEVLKKNCPHIVVGTPGRILALARNKSLNLKHIKHFILDECDK

Uniprot       MLEQLDMRRDVQEIFRMTPHEKQVMMFSATLSKEIRPVCRKFMQDPMEIFVDDETKLTLHGLQQYYVKLKDNEKNRKLFDLLDVLEFNQVVIFVKSVQRC
Splooce       MLEQLDMRRDVQEIFRMTPHEKQVMMFSATLSKEIRPVCRKFMQDPMEIFVDDETKLTLHGLQQYYVKLKDNEKNRKLFDLLDVLEFNQVVIFVKSVQRC

Uniprot       IALAQLLVEQNFPAIAIHRGMPQEERLSRYQQFKDFQRRILVATNLFGRGMDIERVNIAFNYDMPEDSDTYLHRVARAGRFGTKGLAITFVSDENDAKIL
Splooce       IALAQLLVEQNFPAIAIHRGMPQEERLSRYQQFKDFQRRILVATNLFGRGMDIERVNIAFNYDMPEDSDTYLHRVARAGRFGTKGLAITFVSDENDAKIL

Uniprot       NDVQDRFEVNISELPDEIDISSYIEQTR
Splooce       NDVQDRFEVNISELPDEIDISSYIEQTR

----------------------------------------------------------------------------------------------------

O76080 (Uniprot)	versus
NM_001102420#(r:9_Z1354725704799) (Splooce)

For more details about the Alternative Splicing Event -> Link to Splooce page

Peptides that support the ASE (Splooce-specific):
TEMSISREDK (MAXQUANT)

Alignment:
Uniprot       MAQETNQTPGPMLCSTGCGFYGNPRTNGMCSVCYKEHLQRQQNSGRMSPMGTASGSNSPTSDSASVQRADTSLNNCEGAAGSTSEKSRNVPVAALPVTQQ
Splooce       ----------------------------------------------------------------------------------------------------

Uniprot       MTEMSISREDKITTPKTEVSEPVVTQPSPSVSQPSTSQSEEKAPELPKPKKNRCFMCRKKVGLTGFDCRCGNLFCGLHRYSDKHNCPYDYKAEAAAKIRK
Splooce       MTEMSISREDKITTPKTEVSEPVVTQPSPSVSQPSTSQSEEKAPELPKPKKNRCFMCRKKVGLTGFDCRCGNLFCGLHRYSDKHNCPYDYKAEAAAKIRK

Uniprot       ENPVVVAEKIQRI
Splooce       ENPVVVAEKIQRI

----------------------------------------------------------------------------------------------------

Q9Y4P8 (Uniprot)	versus
NM_015610#(-s-:7_W5478775767955) (Splooce)

For more details about the Alternative Splicing Event -> Link to Splooce page

Peptides that support the ASE (Splooce-specific):
MIPAHDSPLAALAFDASGTK (MAXQUANT)

Alignment:
Uniprot       MNLASQSGEAGAGQLLFANFNQDNTEVKGASRAAGLGRRAVVWSLAVGSKSGYKFFSLSSVDKLEQIYECTDTEDVCIVERLFSSSLVAIVSLKAPRKLK
Splooce       ----------------------------------------------------------------------------------------------------

Uniprot       VCHFKKGTEICNYSYSNTILAVKLNRQRLIVCLEESLYIHNIRDMKVLHTIRETPPNPAGLCALSINNDNCYLAYPGSATIGEVQVFDTINLRAANMIPA
Splooce       ------------------------------------------------------------------------------------------------MIPA

Uniprot       HDSPLAALAFDASGTKLATASEKGTVIRVFSIPEGQKLFEFRRGVKRCVSICSLAFSMDGMFLSASSNTETVHIFKLETVKEKPPEEPTTWTGYFGKVLM
Splooce       HDSPLAALAFDASGTKLATASEKGTVIRVFSIPEGQKLFEFRRGVKRCVSICSLAFSMDGMFLSASSNTETVHIFKLETVKEKPPEEPTTWTGYFGKVLM

Uniprot       ASTSYLPSQVTEMFNQGRAFATVRLPFCGHKNICSLATIQKIPRLLVGAADGYLYMYNLDPQEGGECALMKQHRLDGSLETTNEILDSASHDCPLVTQTY
Splooce       ASTSYLPSQVTEMFNQGRAFATVRLPFCGHKNICSLATIQKIPRLLVGAADGYLYMYNLDPQEGGECALMKQHRLDGSLETTNEILDSASHDCPLVTQTY

Uniprot       GAAAGKGTYVPSSPTRLAYTDDLGAVGGACLEDEASALRLDEDSEHPPMILRTD
Splooce       GAAAGKGTYVPSSPTRLAYTDDLGAVGGACLEDEASALRLDEDSEHPPMILRTD

----------------------------------------------------------------------------------------------------

Q92930 (Uniprot)	versus
NM_016530#(-s-:15_R555170445510) (Splooce)

For more details about the Alternative Splicing Event -> Link to Splooce page

Peptides that support the ASE (Splooce-specific):
GLMLVYDLTNEK (PEAKS)
GIMLVYDITNEK (MAXQUANT)

Alignment:
Uniprot       MAKTYDYLFKLLLIGDSGVGKTCLLFRFSEDAFNTTFISTIGIDFKIRTIELDGKKIKLQIWDTAGQERFRTITTAYYRGAMGIMLVYDITNEKSFDNIK
Splooce       ---------------------------------------------------------------------------------MGIMLVYDITNEKSFDNIK

Uniprot       NWIRNIEEHASSDVERMILGNKCDMNDKRQVSKERGEKLAIDYGIKFLETSAKSSANVEEAFFTLARDIMTKLNRKMNDSNSAGAGGPVKITENRSKKTS
Splooce       NWIRNIEEHASSDVERMILGNKCDMNDKRQVSKERGEKLAIDYGIKFLETSAKSSANVEEAFFTLARDIMTKLNRKMNDSNSAGAGGPVKITENRSKKTS

Uniprot       FFRCSLL
Splooce       FFRCSLL

----------------------------------------------------------------------------------------------------

P35579 (Uniprot)	versus
NM_002473#(-s-s-s-s-s-s-s-s-s-s-:22_M842589043945) (Splooce)

For more details about the Alternative Splicing Event -> Link to Splooce page

Peptides that support the ASE (Splooce-specific):
MQEQGTHPK (MAXQUANT)

Alignment:
Uniprot       MAQQAADKYLYVDKNFINNPLAQADWAAKKLVWVPSDKSGFEPASLKEEVGEEAIVELVENGKKVKVNKDDIQKMNPPKFSKVEDMAELTCLNEASVLHN
Splooce       ----------------------------------------------------------------------------------------------------

Uniprot       LKERYYSGLIYTYSGLFCVVINPYKNLPIYSEEIVEMYKGKKRHEMPPHIYAITDTAYRSMMQDREDQSILCTGESGAGKTENTKKVIQYLAYVASSHKS
Splooce       ----------------------------------------------------------------------------------------------------

Uniprot       KKDQGELERQLLQANPILEAFGNAKTVKNDNSSRFGKFIRINFDVNGYIVGANIETYLLEKSRAIRQAKEERTFHIFYYLLSGAGEHLKTDLLLEPYNKY
Splooce       ----------------------------------------------------------------------------------------------------

Uniprot       RFLSNGHVTIPGQQDKDMFQETMEAMRIMGIPEEEQMGLLRVISGVLQLGNIVFKKERNTDQASMPDNTAAQKVSHLLGINVTDFTRGILTPRIKVGRDY
Splooce       ----------------------------------------------------------------------------------------------------

Uniprot       VQKAQTKEQADFAIEALAKATYERMFRWLVLRINKALDKTKRQGASFIGILDIAGFEIFDLNSFEQLCINYTNEKLQQLFNHTMFILEQEEYQREGIEWN
Splooce       ----------------------------------------------------------------------------------------------------

Uniprot       FIDFGLDLQPCIDLIEKPAGPPGILALLDEECWFPKATDKSFVEKVMQEQGTHPKFQKPKQLKDKADFCIIHYAGKVDYKADEWLMKNMDPLNDNIATLL
Splooce       ----------------------------------------------MQEQGTHPKFQKPKQLKDKADFCIIHYAGKVDYKADEWLMKNMDPLNDNIATLL

Uniprot       HQSSDKFVSELWKDVDRIIGLDQVAGMSETALPGAFKTRKGMFRTVGQLYKEQLAKLMATLRNTNPNFVRCIIPNHEKKAGKLDPHLVLDQLRCNGVLEG
Splooce       HQSSDKFVSELWKDVDRIIGLDQVAGMSETALPGAFKTRKGMFRTVGQLYKEQLAKLMATLRNTNPNFVRCIIPNHEKKAGKLDPHLVLDQLRCNGVLEG

Uniprot       IRICRQGFPNRVVFQEFRQRYEILTPNSIPKGFMDGKQACVLMIKALELDSNLYRIGQSKVFFRAGVLAHLEEERDLKITDVIIGFQACCRGYLARKAFA
Splooce       IRICRQGFPNRVVFQEFRQRYEILTPNSIPKGFMDGKQACVLMIKALELDSNLYRIGQSKVFFRAGVLAHLEEERDLKITDVIIGFQACCRGYLARKAFA

Uniprot       KRQQQLTAMKVLQRNCAAYLKLRNWQWWRLFTKVKPLLQVSRQEEEMMAKEEELVKVREKQLAAENRLTEMETLQSQLMAEKLQLQEQLQAETELCAEAE
Splooce       KRQQQLTAMKVLQRNCAAYLKLRNWQWWRLFTKVKPLLQVSRQEEEMMAKEEELVKVREKQLAAENRLTEMETLQSQLMAEKLQLQEQLQAETELCAEAE

Uniprot       ELRARLTAKKQELEEICHDLEARVEEEEERCQHLQAEKKKMQQNIQELEEQLEEEESARQKLQLEKVTTEAKLKKLEEEQIILEDQNCKLAKEKKLLEDR
Splooce       ELRARLTAKKQELEEICHDLEARVEEEEERCQHLQAEKKKMQQNIQELEEQLEEEESARQKLQLEKVTTEAKLKKLEEEQIILEDQNCKLAKEKKLLEDR

Uniprot       IAEFTTNLTEEEEKSKSLAKLKNKHEAMITDLEERLRREEKQRQELEKTRRKLEGDSTDLSDQIAELQAQIAELKMQLAKKEEELQAALARVEEEAAQKN
Splooce       IAEFTTNLTEEEEKSKSLAKLKNKHEAMITDLEERLRREEKQRQELEKTRRKLEGDSTDLSDQIAELQAQIAELKMQLAKKEEELQAALARVEEEAAQKN

Uniprot       MALKKIRELESQISELQEDLESERASRNKAEKQKRDLGEELEALKTELEDTLDSTAAQQELRSKREQEVNILKKTLEEEAKTHEAQIQEMRQKHSQAVEE
Splooce       MALKKIRELESQISELQEDLESERASRNKAEKQKRDLGEELEALKTELEDTLDSTAAQQELRSKREQEVNILKKTLEEEAKTHEAQIQEMRQKHSQAVEE

Uniprot       LAEQLEQTKRVKANLEKAKQTLENERGELANEVKVLLQGKGDSEHKRKKVEAQLQELQVKFNEGERVRTELADKVTKLQVELDNVTGLLSQSDSKSSKLT
Splooce       LAEQLEQTKRVKANLEKAKQTLENERGELANEVKVLLQGKGDSEHKRKKVEAQLQELQVKFNEGERVRTELADKVTKLQVELDNVTGLLSQSDSKSSKLT

Uniprot       KDFSALESQLQDTQELLQEENRQKLSLSTKLKQVEDEKNSFREQLEEEEEAKHNLEKQIATLHAQVADMKKKMEDSVGCLETAEEVKRKLQKDLEGLSQR
Splooce       KDFSALESQLQDTQELLQEENRQKLSLSTKLKQVEDEKNSFREQLEEEEEAKHNLEKQIATLHAQVADMKKKMEDSVGCLETAEEVKRKLQKDLEGLSQR

Uniprot       HEEKVAAYDKLEKTKTRLQQELDDLLVDLDHQRQSACNLEKKQKKFDQLLAEEKTISAKYAEERDRAEAEAREKETKALSLARALEEAMEQKAELERLNK
Splooce       HEEKVAAYDKLEKTKTRLQQELDDLLVDLDHQRQSACNLEKKQKKFDQLLAEEKTISAKYAEERDRAEAEAREKETKALSLARALEEAMEQKAELERLNK

Uniprot       QFRTEMEDLMSSKDDVGKSVHELEKSKRALEQQVEEMKTQLEELEDELQATEDAKLRLEVNLQAMKAQFERDLQGRDEQSEEKKKQLVRQVREMEAELED
Splooce       QFRTEMEDLMSSKDDVGKSVHELEKSKRALEQQVEEMKTQLEELEDELQATEDAKLRLEVNLQAMKAQFERDLQGRDEQSEEKKKQLVRQVREMEAELED

Uniprot       ERKQRSMAVAARKKLEMDLKDLEAHIDSANKNRDEAIKQLRKLQAQMKDCMRELDDTRASREEILAQAKENEKKLKSMEAEMIQLQEELAAAERAKRQAQ
Splooce       ERKQRSMAVAARKKLEMDLKDLEAHIDSANKNRDEAIKQLRKLQAQMKDCMRELDDTRASREEILAQAKENEKKLKSMEAEMIQLQEELAAAERAKRQAQ

Uniprot       QERDELADEIANSSGKGALALEEKRRLEARIAQLEEELEEEQGNTELINDRLKKANLQIDQINTDLNLERSHAQKNENARQQLERQNKELKVKLQEMEGT
Splooce       QERDELADEIANSSGKGALALEEKRRLEARIAQLEEELEEEQGNTELINDRLKKANLQIDQINTDLNLERSHAQKNENARQQLERQNKELKVKLQEMEGT

Uniprot       VKSKYKASITALEAKIAQLEEQLDNETKERQAACKQVRRTEKKLKDVLLQVDDERRNAEQYKDQADKASTRLKQLKRQLEEAEEEAQRANASRRKLQREL
Splooce       VKSKYKASITALEAKIAQLEEQLDNETKERQAACKQVRRTEKKLKDVLLQVDDERRNAEQYKDQADKASTRLKQLKRQLEEAEEEAQRANASRRKLQREL

Uniprot       EDATETADAMNREVSSLKNKLRRGDLPFVVPRRMARKGAGDGSDEEVDGKADGAEAKPAE
Splooce       EDATETADAMNREVSSLKNKLRRGDLPFVVPRRMARKGAGDGSDEEVDGKADGAEAKPAE

----------------------------------------------------------------------------------------------------

Q9NR45 (Uniprot)	versus
NM_018946#(-s-:9_N1494164318268) (Splooce)

For more details about the Alternative Splicing Event -> Link to Splooce page

Peptides that support the ASE (Splooce-specific):
AVEFLHELNVPFFK (MAXQUANT)

Alignment:
Uniprot       MPLELELCPGRWVGGQHPCFIIAEIGQNHQGDLDVAKRMIRMAKECGADCAKFQKSELEFKFNRKALERPYTSKHSWGKTYGEHKRHLEFSHDQYRELQR
Splooce       ----------------------------------------------------------------------------------------------------

Uniprot       YAEEVGIFFTASGMDEMAVEFLHELNVPFFKVGSGDTNNFPYLEKTAKKGRPMVISSGMQSMDTMKQVYQIVKPLNPNFCFLQCTSAYPLQPEDVNLRVI
Splooce       ----------------MAVEFLHELNVPFFKVGSGDTNNFPYLEKTAKKGRPMVISSGMQSMDTMKQVYQIVKPLNPNFCFLQCTSAYPLQPEDVNLRVI

Uniprot       SEYQKLFPDIPIGYSGHETGIAISVAAVALGAKVLERHITLDKTWKGSDHSASLEPGELAELVRSVRLVERALGSPTKQLLPCEMACNEKLGKSVVAKVK
Splooce       SEYQKLFPDIPIGYSGHETGIAISVAAVALGAKVLERHITLDKTWKGSDHSASLEPGELAELVRSVRLVERALGSPTKQLLPCEMACNEKLGKSVVAKVK

Uniprot       IPEGTILTMDMLTVKVGEPKGYPPEDIFNLVGKKVLVTVEEDDTIMEELVDNHGKKIKS
Splooce       IPEGTILTMDMLTVKVGEPKGYPPEDIFNLVGKKVLVTVEEDDTIMEELVDNHGKKIKS

----------------------------------------------------------------------------------------------------

P07910 (Uniprot)	versus
NM_031314#(-s-s-:14_H4687440387186) (Splooce)

For more details about the Alternative Splicing Event -> Link to Splooce page

Peptides that support the ASE (Splooce-specific):
YGSVTEHPSPSPLLSSSFDLDYDFQR (MAXQUANT)

Alignment:
Uniprot       MASNVTNKTDPRSMNSRVFIGNLNTLVVKKSDVEAIFSKYGKIVGCSVHKGFAFVQYVNERNARAAVAGEDGRMIAGQVLDINLAAEPKVNRGKAGVKRS
Splooce       ----------------------------------------------------------------------------------------------------

Uniprot       AAEMYGSVTEHPSPSPLLSSSFDLDYDFQRDYYDRMYSYPARVPPPPPIARAVVPSKRQRVSGNTSRRGKSGFNSKSGQRGSSKSGKLKGDDLQAIKKEL
Splooce       ---MYGSVTEHPSPSPLLSSSFDLDYDFQRDYYDRMYSYPARVPPPPPIARAVVPSKRQRVSGNTSRRGKSGFNSKSGQRGSSKSGKLKGDDLQAIKKEL

Uniprot       TQIKQKVDSLLENLEKIEKEQSKQAVEMKNDKSEEEQSSSSVKKDETNVKMESEGGADDSAEEGDLLDDDDNEDRGDDQLELIKDDEKEAEEGEDDRDSA
Splooce       TQIKQKVDSLLENLEKIEKEQSKQAVEMKNDKSEEEQSSSSVKKDETNVKMESEGGADDSAEEGDLLDDDDNEDRGDDQLELIKDDEKEAEEGEDDRDSA

Uniprot       NGEDDS
Splooce       NGEDDS

----------------------------------------------------------------------------------------------------

Q9NR31 (Uniprot)	versus
NM_020150#(-s-s-:10_S6718750603471) (Splooce)

For more details about the Alternative Splicing Event -> Link to Splooce page

Peptides that support the ASE (Splooce-specific):
TFTTFDLGGHEQAR (MAXQUANT)

Alignment:
Uniprot       MSFIFEWIYNGFSSVLQFLGLYKKSGKLVFLGLDNAGKTTLLHMLKDDRLGQHVPTLHPTSEELTIAGMTFTTFDLGGHEQARRVWKNYLPAINGIVFLV
Splooce       --------------------------------------------------------------------MTFTTFDLGGHEQARRVWKNYLPAINGIVFLV

Uniprot       DCADHSRLVESKVELNALMTDETISNVPILILGNKIDRTDAISEEKLREIFGLYGQTTGKGNVTLKELNARPMEVFMCSVLKRQGYGEGFRWLSQYID
Splooce       DCADHSRLVESKVELNALMTDETISNVPILILGNKIDRTDAISEEKLREIFGLYGQTTGKGNVTLKELNARPMEVFMCSVLKRQGYGEGFRWLSQYID

----------------------------------------------------------------------------------------------------

P13010 (Uniprot)	versus
NM_021141#(-s-:2_X5408191843275) (Splooce)

For more details about the Alternative Splicing Event -> Link to Splooce page

Peptides that support the ASE (Splooce-specific):
DVIQHETIGK (MAXQUANT)
DVLQHETLGK (PEAKS)

Alignment:
Uniprot       MVRSGNKAAVVLCMDVGFTMSNSIPGIESPFEQAKKVITMFVQRQVFAENKDEIALVLFGTDGTDNPLSGGDQYQNITVHRHLMLPDFDLLEDIESKIQP
Splooce       ----------------------------------------------------------------------------------------------------

Uniprot       GSQQADFLDALIVSMDVIQHETIGKKFEKRHIEIFTDLSSRFSKSQLDIIIHSLKKCDISLQFFLPFSLGKEDGSGDRGDGPFRLGGHGPSFPLKGITEQ
Splooce       --------------MDVIQHETIGKKFEKRHIEIFTDLSSRFSKSQLDIIIHSLKKCDISLQFFLPFSLGKEDGSGDRGDGPFRLGGHGPSFPLKGITEQ

Uniprot       QKEGLEIVKMVMISLEGEDGLDEIYSFSESLRKLCVFKKIERHSIHWPCRLTIGSNLSIRIAAYKSILQERVKKTWTVVDAKTLKKEDIQKETVYCLNDD
Splooce       QKEGLEIVKMVMISLEGEDGLDEIYSFSESLRKLCVFKKIERHSIHWPCRLTIGSNLSIRIAAYKSILQERVKKTWTVVDAKTLKKEDIQKETVYCLNDD

Uniprot       DETEVLKEDIIQGFRYGSDIVPFSKVDEEQMKYKSEGKCFSVLGFCKSSQVQRRFFMGNQVLKVFAARDDEAAAVALSSLIHALDDLDMVAIVRYAYDKR
Splooce       DETEVLKEDIIQGFRYGSDIVPFSKVDEEQMKYKSEGKCFSVLGFCKSSQVQRRFFMGNQVLKVFAARDDEAAAVALSSLIHALDDLDMVAIVRYAYDKR

Uniprot       ANPQVGVAFPHIKHNYECLVYVQLPFMEDLRQYMFSSLKNSKKYAPTEAQLNAVDALIDSMSLAKKDEKTDTLEDLFPTTKIPNPRFQRLFQCLLHRALH
Splooce       ANPQVGVAFPHIKHNYECLVYVQLPFMEDLRQYMFSSLKNSKKYAPTEAQLNAVDALIDSMSLAKKDEKTDTLEDLFPTTKIPNPRFQRLFQCLLHRALH

Uniprot       PREPLPPIQQHIWNMLNPPAEVTTKSQIPLSKIKTLFPLIEAKKKDQVTAQEIFQDNHEDGPTAKKLKTEQGGAHFSVSSLAEGSVTSVGSVNPAENFRV
Splooce       PREPLPPIQQHIWNMLNPPAEVTTKSQIPLSKIKTLFPLIEAKKKDQVTAQEIFQDNHEDGPTAKKLKTEQGGAHFSVSSLAEGSVTSVGSVNPAENFRV

Uniprot       LVKQKKASFEEASNQLINHIEQFLDTNETPYFMKSIDCIRAFREEAIKFSEEQRFNNFLKALQEKVEIKQLNHFWEIVVQDGITLITKEEASGSSVTAEE
Splooce       LVKQKKASFEEASNQLINHIEQFLDTNETPYFMKSIDCIRAFREEAIKFSEEQRFNNFLKALQEKVEIKQLNHFWEIVVQDGITLITKEEASGSSVTAEE

Uniprot       AKKFLAPKDKPSGDTAAVFEEGGDVDDLLDMI
Splooce       AKKFLAPKDKPSGDTAAVFEEGGDVDDLLDMI

----------------------------------------------------------------------------------------------------

P27824 (Uniprot)	versus
NM_001746#(-s-s-s-s-:5_C5003394282904) (Splooce)

For more details about the Alternative Splicing Event -> Link to Splooce page

Peptides that support the ASE (Splooce-specific):
MDGEWEAPQIANPR (MAXQUANT)

Alignment:
Uniprot       MEGKWLLCMLLVLGTAIVEAHDGHDDDVIDIEDDLDDVIEEVEDSKPDTTAPPSSPKVTYKAPVPTGEVYFADSFDRGTLSGWILSKAKKDDTDDEIAKY
Splooce       ----------------------------------------------------------------------------------------------------

Uniprot       DGKWEVEEMKESKLPGDKGLVLMSRAKHHAISAKLNKPFLFDTKPLIVQYEVNFQNGIECGGAYVKLLSKTPELNLDQFHDKTPYTIMFGPDKCGEDYKL
Splooce       ----------------------------------------------------------------------------------------------------

Uniprot       HFIFRHKNPKTGIYEEKHAKRPDADLKTYFTDKKTHLYTLILNPDNSFEILVDQSVVNSGNLLNDMTPPVNPSREIEDPEDRKPEDWDERPKIPDPEAVK
Splooce       ----------------------------------------------------------------------------------------------------

Uniprot       PDDWDEDAPAKIPDEEATKPEGWLDDEPEYVPDPDAEKPEDWDEDMDGEWEAPQIANPRCESAPGCGVWQRPVIDNPNYKGKWKPPMIDNPSYQGIWKPR
Splooce       ---------------------------------------------MDGEWEAPQIANPRCESAPGCGVWQRPVIDNPNYKGKWKPPMIDNPSYQGIWKPR

Uniprot       KIPNPDFFEDLEPFRMTPFSAIGLELWSMTSDIFFDNFIICADRRIVDDWANDGWGLKKAADGAAEPGVVGQMIEAAEERPWLWVVYILTVALPVFLVIL
Splooce       KIPNPDFFEDLEPFRMTPFSAIGLELWSMTSDIFFDNFIICADRRIVDDWANDGWGLKKAADGAAEPGVVGQMIEAAEERPWLWVVYILTVALPVFLVIL

Uniprot       FCCSGKKQTSGMEYKKTDAPQPDVKEEEEEKEEEKDKGDEEEEGEEKLEEKQKSDAEEDGGTVSQEEEDRKPKAEEDEILNRSPRNRKPRRE
Splooce       FCCSGKKQTSGMEYKKTDAPQPDVKEEEEEKEEEKDKGDEEEEGEEKLEEKQKSDAEEDGGTVSQEEEDRKPKAEEDEILNRSPRNRKPRRE

----------------------------------------------------------------------------------------------------

Q9NVN8 (Uniprot)	versus
NM_019067#(-s-s-s-:X_G48692256681) (Splooce)

For more details about the Alternative Splicing Event -> Link to Splooce page

Peptides that support the ASE (Splooce-specific):
QEVYLDK (MAXQUANT)

Alignment:
Uniprot       MMKLRHKNKKPGEGSKGHKKISWPYPQPAKQNGKKATSKVPSAPHFVHPNDHANREAELKKKWVEEMREKQQAAREQERQKRRTIESYCQDVLRRQEEFE
Splooce       ----------------------------------------------------------------------------------------------------

Uniprot       HKEEVLQELNMFPQLDDEATRKAYYKEFRKVVEYSDVILEVLDARDPLGCRCFQMEEAVLRAQGNKKLVLVLNKIDLVPKEVVEKWLDYLRNELPTVAFK
Splooce       ----------------------------------------------------------------------------------------------------

Uniprot       ASTQHQVKNLNRCSVPVDQASESLLKSKACFGAENLMRVLGNYCRLGEVRTHIRVGVVGLPNVGKSSLINSLKRSRACSVGAVPGITKFMQEVYLDKFIR
Splooce       -----------------------------------------------------------------------------------------MQEVYLDKFIR

Uniprot       LLDAPGIVPGPNSEVGTILRNCVHVQKLADPVTPVETILQRCNLEEISNYYGVSGFQTTEHFLTAVAHRLGKKKKGGLYSQEQAAKAVLADWVSGKISFY
Splooce       LLDAPGIVPGPNSEVGTILRNCVHVQKLADPVTPVETILQRCNLEEISNYYGVSGFQTTEHFLTAVAHRLGKKKKGGLYSQEQAAKAVLADWVSGKISFY

Uniprot       IPPPATHTLPTHLSAEIVKEMTEVFDIEDTEQANEDTMECLATGESDELLGDTDPLEMEIKLLHSPMTKIADAIENKTTVYKIGDLTGYCTNPNRHQMGW
Splooce       IPPPATHTLPTHLSAEIVKEMTEVFDIEDTEQANEDTMECLATGESDELLGDTDPLEMEIKLLHSPMTKIADAIENKTTVYKIGDLTGYCTNPNRHQMGW

Uniprot       AKRNVDHRPKSNSMVDVCSVDRRSVLQRIMETDPLQQGQALASALKNKKKMQKRADKIASKLSDSMMSALDLSGNADDGVGD
Splooce       AKRNVDHRPKSNSMVDVCSVDRRSVLQRIMETDPLQQGQALASALKNKKKMQKRADKIASKLSDSMMSALDLSGNADDGVGD

----------------------------------------------------------------------------------------------------

E7EUT4 (Uniprot)	versus
NM_002046#(-s-s-:12_G9320657958537) (Splooce)

For more details about the Alternative Splicing Event -> Link to Splooce page

Peptides that support the ASE (Splooce-specific):
MFQYDSTHGKFHGTVK (MAXQUANT)
FQYDSTHGK (MAXQUANT + PEAKS)
MFQYDSTHGK (MAXQUANT + PEAKS)

Alignment:
Uniprot       MGKVKVGVNGFGRIGRLVTRAAFNSGKVDIVAINDPFIDLNYMVYMFQYDSTHGKFHGTVKAENGKLVINGNPITIFQERDPSKIKWGDAGAEYVVESTG
Splooce       ---------------------------------------------MFQYDSTHGKFHGTVKAENGKLVINGNPITIFQERDPSKIKWGDAGAEYVVESTG

Uniprot       VFTTMEKAGAHLQGGAKRVIISAPSADAPMFVMGVNHEKYDNSLKIISNASCTTNCLAPLAKVIHDNFGIVEGLMTTVHAITATQKTVDGPSGKLWRDGR
Splooce       VFTTMEKAGAHLQGGAKRVIISAPSADAPMFVMGVNHEKYDNSLKIISNASCTTNCLAPLAKVIHDNFGIVEGLMTTVHAITATQKTVDGPSGKLWRDGR

Uniprot       GALQNIIPASTGAAKAVGKVIPELNGKLTGMAFRVPTANVSVVDLTCRLEKPAKYDDIKKVVKQASEGPLKGILGYTEHQVVSSDFNSDTHSSTFDAGAG
Splooce       GALQNIIPASTGAAKAVGKVIPELNGKLTGMAFRVPTANVSVVDLTCRLEKPAKYDDIKKVVKQASEGPLKGILGYTEHQVVSSDFNSDTHSSTFDAGAG

Uniprot       IALNDHFVKLISWYDNEFGYSNRVVDLMAHMASKE
Splooce       IALNDHFVKLISWYDNEFGYSNRVVDLMAHMASKE

----------------------------------------------------------------------------------------------------

P31483 (Uniprot)	versus
NM_022173#(-s-s-s-:2_T4152205289438) (Splooce)

For more details about the Alternative Splicing Event -> Link to Splooce page

Peptides that support the ASE (Splooce-specific):
MGGQWLGGR (MAXQUANT)

Alignment:
Uniprot       MEDEMPKTLYVGNLSRDVTEALILQLFSQIGPCKNCKMIMDTAGNDPYCFVEFHEHRHAAAALAAMNGRKIMGKEVKVNWATTPSSQKKDTSSSTVVSTQ
Splooce       ----------------------------------------------------------------------------------------------------

Uniprot       RSQDHFHVFVGDLSPEITTEDIKAAFAPFGRISDARVVKDMATGKSKGYGFVSFFNKWDAENAIQQMGGQWLGGRQIRTNWATRKPPAPKSTYESNTKQL
Splooce       ------------------------------------------------------------------MGGQWLGGRQIRTNWATRKPPAPKSTYESNTKQL

Uniprot       SYDEVVNQSSPSNCTVYCGGVTSGLTEQLMRQTFSPFGQIMEIRVFPDKGYSFVRFNSHESAAHAIVSVNGTTIEGHVVKCYWGKETLDMINPVQQQNQI
Splooce       SYDEVVNQSSPSNCTVYCGGVTSGLTEQLMRQTFSPFGQIMEIRVFPDKGYSFVRFNSHESAAHAIVSVNGTTIEGHVVKCYWGKETLDMINPVQQQNQI

Uniprot       GYPQPYGQWGQWYGNAQQIGQYMPNGWQVPAYGMYGQAWNQQGFNQTQSSAPWMGPNYGVQPPQGQNGSMLPNQPSGYRVAGYETQ
Splooce       GYPQPYGQWGQWYGNAQQIGQYMPNGWQVPAYGMYGQAWNQQGFNQTQSSAPWMGPNYGVQPPQGQNGSMLPNQPSGYRVAGYETQ

----------------------------------------------------------------------------------------------------

P43686 (Uniprot)	versus
NM_153001#(-s-:19_P8806635891713) (Splooce)

For more details about the Alternative Splicing Event -> Link to Splooce page

Peptides that support the ASE (Splooce-specific):
MLTSDQKPDVMYADIGGMDIQK (MAXQUANT)

Alignment:
Uniprot       MEEIGILVEKAQDEIPALSVSRPQTGLSFLGPEPEDLEDLYSRYKEEVKRIQSIPLVIGQFLEAVDQNTAIVGSTTGSNYYVRILSTIDRELLKPNASVA
Splooce       ----------------------------------------------------------------------------------------------------

Uniprot       LHKHSNALVDVLPPEADSSIMMLTSDQKPDVMYADIGGMDIQKQEVREAVELPLTHFELYKQIGIDPPRGVLMYGPPGCGKTMLAKAVAHHTTAAFIRVV
Splooce       --------------------MMLTSDQKPDVMYADIGGMDIQKQEVREAVELPLTHFELYKQIGIDPPRGVLMYGPPGCGKTMLAKAVAHHTTAAFIRVV

Uniprot       GSEFVQKYLGEGPRMVRDVFRLAKENAPAIIFIDEIDAIATKRFDAQTGADREVQRILLELLNQMDGFDQNVNVKVIMATNRADTLDPALLRPGRLDRKI
Splooce       GSEFVQKYLGEGPRMVRDVFRLAKENAPAIIFIDEIDAIATKRFDAQTGADREVQRILLELLNQMDGFDQNVNVKVIMATNRADTLDPALLRPGRLDRKI

Uniprot       EFPLPDRRQKRLIFSTITSKMNLSEEVDLEDYVARPDKISGADINSICQESGMLAVRENRYIVLAKDFEKAYKTVIKKDEQEHEFYK
Splooce       EFPLPDRRQKRLIFSTITSKMNLSEEVDLEDYVARPDKISGADINSICQESGMLAVRENRYIVLAKDFEKAYKTVIKKDEQEHEFYK

----------------------------------------------------------------------------------------------------

P68104 (Uniprot)	versus
NM_001402#(-s-s-s-s-s-:6_E3893517591451) (Splooce)

For more details about the Alternative Splicing Event -> Link to Splooce page

Peptides that support the ASE (Splooce-specific):
VPGKPMCVESFSDYPPLGR (MAXQUANT)
MVPGKPMCVESFSDYPPLGR (MAXQUANT)

Alignment:
Uniprot       MGKEKTHINIVVIGHVDSGKSTTTGHLIYKCGGIDKRTIEKFEKEAAEMGKGSFKYAWVLDKLKAERERGITIDISLWKFETSKYYVTIIDAPGHRDFIK
Splooce       ----------------------------------------------------------------------------------------------------

Uniprot       NMITGTSQADCAVLIVAAGVGEFEAGISKNGQTREHALLAYTLGVKQLIVGVNKMDSTEPPYSQKRYEEIVKEVSTYIKKIGYNPDTVAFVPISGWNGDN
Splooce       ----------------------------------------------------------------------------------------------------

Uniprot       MLEPSANMPWFKGWKVTRKDGNASGTTLLEALDCILPPTRPTDKPLRLPLQDVYKIGGIGTVPVGRVETGVLKPGMVVTFAPVNVTTEVKSVEMHHEALS
Splooce       ----------------------------------------------------------------------------------------------------

Uniprot       EALPGDNVGFNVKNVSVKDVRRGNVAGDSKNDPPMEAAGFTAQVIILNHPGQISAGYAPVLDCHTAHIACKFAELKEKIDRRSGKKLEDGPKFLKSGDAA
Splooce       ----------------------------------------------------------------------------------------------------

Uniprot       IVDMVPGKPMCVESFSDYPPLGRFAVRDMRQTVAVGVIKAVDKKAAGAGKVTKSAQKAQKAK
Splooce       ---MVPGKPMCVESFSDYPPLGRFAVRDMRQTVAVGVIKAVDKKAAGAGKVTKSAQKAQKAK

----------------------------------------------------------------------------------------------------

B7Z840 (Uniprot)	versus
NM_005051#(f-:3_Q2985235380043) (Splooce)

For more details about the Alternative Splicing Event -> Link to Splooce page

Peptides that support the ASE (Splooce-specific):
MGLLMGEAR (MAXQUANT)

Alignment:
Uniprot       MAALDSLSLFTSLGLSEQKARETLKNSALSAQLREAATQAQQTLGSTIDKATGILLYGLASRLRDTRRLSFLVSYIASKKIHTEPQLSAALEYVRSHPLD
Splooce       ----------------------------------------------------------------------------------------------------

Uniprot       PIDTVDFERECGVGVIVTPEQIEEAVEAAINRHRPQLLVERYHFNMGLLMGEARAVLKWADGKMIKNEVDMQVLHLLGPKLEADLEKKFKVAKARLEETD
Splooce       ---------------------------------------------MGLLMGEARAVLKWADGKMIKNEVDMQVLHLLGPKLEADLEKKFKVAKARLEETD

Uniprot       RRTAKDVVENGETADQTLSLMEQLRGEALKFHKPGENYKTPGYVVTPHTMNLLKQHLEITGGQVRTRFPPEPNGILHIGHAKAINFNFGYAKANNGICFL
Splooce       RRTAKDVVENGETADQTLSLMEQLRGEALKFHKPGENYKTPGYVVTPHTMNLLKQHLEITGGQVRTRFPPEPNGILHIGHAKAINFNFGYAKANNGICFL

Uniprot       RFDDTNPEKEEAKFFTAICDMVAWLGYTPYKVTYASDYFDQLYAWAVELIRRGLAYVCHQRGEELKGHNTLPSPWRDRPMEESLLLFEAMRKGKFSEGEA
Splooce       RFDDTNPEKEEAKFFTAICDMVAWLGYTPYKVTYASDYFDQLYAWAVELIRRGLAYVCHQRGEELKGHNTLPSPWRDRPMEESLLLFEAMRKGKFSEGEA

Uniprot       TLRMKLVMEDGKMDPVAYRVKYTPHHRTGDKWCIYPTYDYTHCLCDSIEHITHSLCTKEFQARRSSYFWLCNALDVYCPVQWEYGRLNLHYAVVSKRKIL
Splooce       TLRMKLVMEDGKMDPVAYRVKYTPHHRTGDKWCIYPTYDYTHCLCDSIEHITHSLCTKEFQARRSSYFWLCNALDVYCPVQWEYGRLNLHYAVVSKRKIL

Uniprot       QLVATGAVRDWDDPRLFTLTALRRRGFPPEAINNFCARVGVTVAQTTMEPHLLEACVRDVLNDTAPRAMAVLESLRVIITNFPAAKSLDIQVPNFPADET
Splooce       QLVATGAVRDWDDPRLFTLTALRRRGFPPEAINNFCARVGVTVAQTTMEPHLLEACVRDVLNDTAPRAMAVLESLRVIITNFPAAKSLDIQVPNFPADET

Uniprot       KGFHQVPFAPIVFIERTDFKEEPEPGFKRLAWGQPVGLRHTGYVIELQHVVKGPSGCVESLEVTCRRADAGEKPKAFIHWVSQPLMCEVRLYERLFQHKN
Splooce       KGFHQVPFAPIVFIERTDFKEEPEPGFKRLAWGQPVGLRHTGYVIELQHVVKGPSGCVESLEVTCRRADAGEKPKAFIHWVSQPLMCEVRLYERLFQHKN

Uniprot       PEDPTEVPGGFLSDLNLASLHVVDAALVDCSVALAKPFDKFQFERLGYFSVDPDSHQGKLVFNRTVTLKEDPGKV
Splooce       PEDPTEVPGGFLSDLNLASLHVVDAALVDCSVALAKPFDKFQFERLGYFSVDPDSHQGKLVFNRTVTLKEDPGKV

----------------------------------------------------------------------------------------------------

Q13505 (Uniprot)	versus
NM_002455#(r:1_M5056571408618) (Splooce)

For more details about the Alternative Splicing Event -> Link to Splooce page

Peptides that support the ASE (Splooce-specific):
MSLLEEK (MAXQUANT + PEAKS)

Alignment:
Uniprot       MLLGGPPRSPRSGTSPKGPWSSTGHVQFGKSPQTWPRRTRPRSPEPAAPSGVRGSTWTRRRDTPRRAGPTALSRYVGHLWMGRRPPSPEARGPVPRSSAA
Splooce       ----------------------------------------------------------------------------------------------------

Uniprot       SRARRSLASPGISPGPLTATIGGAVAGGGPRQGRAEAHKEVFPGQRVGKMAAPMELFCWSGGWGLPSVDLDSLAVLTYARFTGAPLKVHKISNPWQSPSG
Splooce       ----------------------------------------------------------------------------------------------------

Uniprot       TLPALRTSHGEVISVPHKIITHLRKEKYNADYDLSARQGADTLAFMSLLEEKLLPVLVHTFWIDTKNYVEVTRKWYAEAMPFPLNFFLPGRMQRQYMERL
Splooce       ---------------------------------------------MSLLEEKLLPVLVHTFWIDTKNYVEVTRKWYAEAMPFPLNFFLPGRMQRQYMERL

Uniprot       QLLTGEHRPEDEEELEKELYREARECLTLLSQRLGSQKFFFGDAPASLDAFVFSYLALLLQAKLPSGKLQVHLRGLHNLCAYCTHILSLYFPWDGAEVPP
Splooce       QLLTGEHRPEDEEELEKELYREARECLTLLSQRLGSQKFFFGDAPASLDAFVFSYLALLLQAKLPSGKLQVHLRGLHNLCAYCTHILSLYFPWDGAEVPP

Uniprot       QRQTPAGPETEEEPYRRRNQILSVLAGLAAMVGYALLSGIVSIQRATPARAPGTRTLGMAEEDEEE
Splooce       QRQTPAGPETEEEPYRRRNQILSVLAGLAAMVGYALLSGIVSIQRATPARAPGTRTLGMAEEDEEE

----------------------------------------------------------------------------------------------------

Q8IWZ8 (Uniprot)	versus
NM_172231#(-t:19_S8499991317703) (Splooce)

For more details about the Alternative Splicing Event -> Link to Splooce page

Peptides that support the ASE (Splooce-specific):
LQEELIAQK (MAXQUANT)

Alignment:
Uniprot       MSLKMDNRDVAGKANRWFGVAPPKSGKMNMNILHQEELIAQKKREIEAKMEQKAKQNQVASPQPPHPGEITNAHNSSCISNKFANDGSFLQQFLKLQKAQ
Splooce       --------------------------------MLQEELIAQKKREIEAKMEQKAKQNQVASPQPPHPGEITNAHNSSCISNKFANDGSFLQQFLKLQKAQ

Uniprot       TSTDAPTSAPSAPPSTPTPSAGKRSLLISRRTGLGLASLPGPVKSYSHAKQLPVAHRPSVFQSPDEDEEEDYEQWLEIKVSPPEGAETRKVIEKLARFVA
Splooce       TSTDAPTSAPSAPPSTPTPSAGKRSLLISRRTGLGLASLPGPVKSYSHAKQLPVAHRPSVFQSPDEDEEEDYEQWLEIKVSPPEGAETRKVIEKLARFVA

Uniprot       EGGPELEKVAMEDYKDNPAFAFLHDKNSREFLYYRKKVAEIRKEAQKSQAASQKVSPPEDEEVKNLAEKLARFIADGGPEVETIALQNNRENQAFSFLYE
Splooce       EGGPELEKVAMEDYKDNPAFAFLHDKNSREFLYYRKKVAEIRKEAQKSQAASQKVSPPEDEEVKNLAEKLARFIADGGPEVETIALQNNRENQAFSFLYE

Uniprot       PNSQGYKYYRQKLEEFRKAKASSTGSFTAPDPGLKRKSPPEALSGSLPPATTCPASSTPAPTIIPAPAAPGKPASAATVKRKRKSRWGPEEDKVELPPAE
Splooce       PNSQGYKYYRQKLEEFRKAKASSTGSFTAPDPGLKRKSPPEALSGSLPPATTCPASSTPAPTIIPAPAAPGKPASAATVKRKRKSRWGPEEDKVELPPAE

Uniprot       LVQRDVDASPSPLSVQDLKGLGYEKGKPVGLVGVTELSDAQKKQLKEQQEMQQMYDMIMQHKRAMQDMQLLWEKAVQQHQHGYDSDEEVDSELGTWEHQL
Splooce       LVQRDVDASPSPLSVQDLKGLGYEKGKPVGLVGVTELSDAQKKQLKEQQEMQQMYDMIMQHKRAMQDMQLLWEKAVQQHQHGYDSDEEVDSELGTWEHQL

Uniprot       RRMEMDKTREWAEQLTKMGRGKHFIGDFLPPDELEKFMETFKALKEGREPDYSEYKEFKLTVENIGYQMLMKMGWKEGEGLGSEGQGIKNPVNKGTTTVD
Splooce       RRMEMDKTREWAEQLTKMGRGKHFIGDFLPPDELEKFMETFKALKEGREPDYSEYKEFKLTVENIGYQMLMKMGWKEGEGLGSEGQGIKNPVNKGTTTVD

Uniprot       GAGFGIDRPAELSKEDDEYEAFRKRMMLAYRFRPNPLNNPRRPYY
Splooce       GAGFGIDRPAELSKEDDEYEAFRKRMMLAYRFRPNPLNNPRRPYY

----------------------------------------------------------------------------------------------------

Q9UBQ7 (Uniprot)	versus
NM_012203#(-s-:9_G5864951506829) (Splooce)

For more details about the Alternative Splicing Event -> Link to Splooce page

Peptides that support the ASE (Splooce-specific):
SVGIDHLALDEIKK (MAXQUANT)
SVGIDHLALDEIK (MAXQUANT)
SVGLDHLALDELK (PEAKS)

Alignment:
Uniprot       MRPVRLMKVFVTRRIPAEGRVALARAADCEVEQWDSDEPIPAKELERGVAGAHGLLCLLSDHVDKRILDAAGANLKVISTMSVGIDHLALDEIKKRGIRV
Splooce       --------------------------------------------------------------------------------MSVGIDHLALDEIKKRGIRV

Uniprot       GYTPDVLTDTTAELAVSLLLTTCRRLPEAIEEVKNGGWTSWKPLWLCGYGLTQSTVGIIGLGRIGQAIARRLKPFGVQRFLYTGRQPRPEEAAEFQAEFV
Splooce       GYTPDVLTDTTAELAVSLLLTTCRRLPEAIEEVKNGGWTSWKPLWLCGYGLTQSTVGIIGLGRIGQAIARRLKPFGVQRFLYTGRQPRPEEAAEFQAEFV

Uniprot       STPELAAQSDFIVVACSLTPATEGLCNKDFFQKMKETAVFINISRGDVVNQDDLYQALASGKIAAAGLDVTSPEPLPTNHPLLTLKNCVILPHIGSATHR
Splooce       STPELAAQSDFIVVACSLTPATEGLCNKDFFQKMKETAVFINISRGDVVNQDDLYQALASGKIAAAGLDVTSPEPLPTNHPLLTLKNCVILPHIGSATHR

Uniprot       TRNTMSLLAANNLLAGLRGEPMPSELKL
Splooce       TRNTMSLLAANNLLAGLRGEPMPSELKL

----------------------------------------------------------------------------------------------------

P11413 (Uniprot)	versus
NM_001042351#(-s-:X_G8989155101404) (Splooce)

For more details about the Alternative Splicing Event -> Link to Splooce page

Peptides that support the ASE (Splooce-specific):
MNALHLGSQANR (MAXQUANT + PEAKS)

Alignment:
Uniprot       MAEQVALSRTQVCGILREELFQGDAFHQSDTHIFIIMGASGDLAKKKIYPTIWWLFRDGLLPENTFIVGYARSRLTVADIRKQSEPFFKATPEEKLKLED
Splooce       ----------------------------------------------------------------------------------------------------

Uniprot       FFARNSYVAGQYDDAASYQRLNSHMNALHLGSQANRLFYLALPPTVYEAVTKNIHESCMSQIGWNRIIVEKPFGRDLQSSDRLSNHISSLFREDQIYRID
Splooce       ------------------------MNALHLGSQANRLFYLALPPTVYEAVTKNIHESCMSQIGWNRIIVEKPFGRDLQSSDRLSNHISSLFREDQIYRID

Uniprot       HYLGKEMVQNLMVLRFANRIFGPIWNRDNIACVILTFKEPFGTEGRGGYFDEFGIIRDVMQNHLLQMLCLVAMEKPASTNSDDVRDEKVKVLKCISEVQA
Splooce       HYLGKEMVQNLMVLRFANRIFGPIWNRDNIACVILTFKEPFGTEGRGGYFDEFGIIRDVMQNHLLQMLCLVAMEKPASTNSDDVRDEKVKVLKCISEVQA

Uniprot       NNVVLGQYVGNPDGEGEATKGYLDDPTVPRGSTTATFAAVVLYVENERWDGVPFILRCGKALNERKAEVRLQFHDVAGDIFHQQCKRNELVIRVQPNEAV
Splooce       NNVVLGQYVGNPDGEGEATKGYLDDPTVPRGSTTATFAAVVLYVENERWDGVPFILRCGKALNERKAEVRLQFHDVAGDIFHQQCKRNELVIRVQPNEAV

Uniprot       YTKMMTKKPGMFFNPEESELDLTYGNRYKNVKLPDAYERLILDVFCGSQMHFVRSDELREAWRIFTPLLHQIELEKPKPIPYIYGSRGPTEADELMKRVG
Splooce       YTKMMTKKPGMFFNPEESELDLTYGNRYKNVKLPDAYERLILDVFCGSQMHFVRSDELREAWRIFTPLLHQIELEKPKPIPYIYGSRGPTEADELMKRVG

Uniprot       FQYEGTYKWVNPHKL
Splooce       FQYEGTYKWVNPHKL

----------------------------------------------------------------------------------------------------

P00558 (Uniprot)	versus
NM_000291#(f-T:X_P6336186253668) (Splooce)

For more details about the Alternative Splicing Event -> Link to Splooce page

Peptides that support the ASE (Splooce-specific):
VGVNLPQK (MAXQUANT + PEAKS)

Alignment:
Uniprot       MSLSNKLTLDKLDVKGKRVVMRVDFNVPMKNNQITNNQRIKAAVPSIKFCLDNGAKSVVLMSHLGRPDGVPMPDKYSLEPVAVELKSLLGKDVLFLKDCV
Splooce       ----------------------------------------------------------------------------------------------------

Uniprot       GPEVEKACANPAAGSVILLENLRFHVEEEGKGKDASGNKVKAEPAKIEAFRASLSKLGDVYVNDAFGTAHRAHSSMVGVNLPQKAGGFLMKKELNYFAKA
Splooce       ---------------------------------------------------------------------------MVGVNLPQKAGGFLMKKELNYFAKA

Uniprot       LESPERPFLAILGGAKVADKIQLINNMLDKVNEMIIGGGMAFTFLKVLNNMEIGTSLFDEEGAKIVKDLMSKAEKNGVKITLPVDFVTADKFDENAKTGQ
Splooce       LESPERPFLAILGGAKVADKIQLINNMLDKVNEMIIGGGMAFTFLKVLNNMEIGTSLFDEEGAKIVKDLMSKAEKNGVKITLPVDFVTADKFDENAKTGQ

Uniprot       ATVASGIPAGWMGLDCGPESSKKYAEAVTRAKQIVWNGPVGVFEWEAFARGTKALMDEVVKATSRGCITIIGGGDTATCCAKWNTEDKVSHVSTGGGASL
Splooce       ATVASGIPAGWMGLDCGPESSKKYAEAVTRAKQIVWNGPVGVFEWEAFARGTKALMDEVVKATSRGCITIIGGGDTATCCAKWNTEDKVSHVSTGGGASL

Uniprot       ELLEGKVLPGVDALSNI
Splooce       ELLEGKVLPGVDALSNI

----------------------------------------------------------------------------------------------------

Q92504 (Uniprot)	versus
NM_001077516#(-t:6_S9093373123999) (Splooce)

For more details about the Alternative Splicing Event -> Link to Splooce page

Peptides that support the ASE (Splooce-specific):
TVLLHEVPHEVGDFAILVQSGCSK (MAXQUANT)

Alignment:
Uniprot       MARGLGAPHWVAVGLLTWATLGLLVAGLGGHDDLHDDLQEDFHGHSHRHSHEDFHHGHSHAHGHGHTHESIWHGHTHDHDHGHSHEDLHHGHSHGYSHES
Splooce       ----------------------------------------------------------------------------------------------------

Uniprot       LYHRGHGHDHEHSHGGYGESGAPGIKQDLDAVTLWAYALGATVLISAAPFFVLFLIPVESNSPRHRSLLQILLSFASGGLLGDAFLHLIPHALEPHSHHT
Splooce       ----------------------------------------------------------------------------------------------------

Uniprot       LEQPGHGHSHSGQGPILSVGLWVLSGIVAFLVVEKFVRHVKGGHGHSHGHGHAHSHTRGSHGHGRQERSTKEKQSSEEEEKETRGVQKRRGGSTVPKDGP
Splooce       ----------------------------------------------------------------------------------------------------

Uniprot       VRPQNAEEEKRGLDLRVSGYLNLAADLAHNFTDGLAIGASFRGGRGLGILTTMTVLLHEVPHEVGDFAILVQSGCSKKQAMRLQLLTAVGALAGTACALL
Splooce       ----------------------------------------------------MTVLLHEVPHEVGDFAILVQSGCSKKQAMRLQLLTAVGALAGTACALL

Uniprot       TEGGAVGSEIAGGAGPGWVLPFTAGGFIYVATVSVLPELLREASPLQSLLEVLGLLGGVIMMVLIAHLE
Splooce       TEGGAVGSEIAGGAGPGWVLPFTAGGFIYVATVSVLPELLREASPLQSLLEVLGLLGGVIMMVLIAHLE

----------------------------------------------------------------------------------------------------

P11388 (Uniprot)	versus
NM_001067#(-s-s-s-s-s-s-:17_T5292746341757) (Splooce)

For more details about the Alternative Splicing Event -> Link to Splooce page

Peptides that support the ASE (Splooce-specific):
VLINGAEGIGTGWSCK (MAXQUANT)
VLLNGAEGLGTGWSCK (PEAKS)

Alignment:
Uniprot       MEVSPLQPVNENMQVNKIKKNEDAKKRLSVERIYQKKTQLEHILLRPDTYIGSVELVTQQMWVYDEDVGINYREVTFVPGLYKIFDEILVNAADNKQRDP
Splooce       ----------------------------------------------------------------------------------------------------

Uniprot       KMSCIRVTIDPENNLISIWNNGKGIPVVEHKVEKMYVPALIFGQLLTSSNYDDDEKKVTGGRNGYGAKLCNIFSTKFTVETASREYKKMFKQTWMDNMGR
Splooce       ----------------------------------------------------------------------------------------------------

Uniprot       AGEMELKPFNGEDYTCITFQPDLSKFKMQSLDKDIVALMVRRAYDIAGSTKDVKVFLNGNKLPVKGFRSYVDMYLKDKLDETGNSLKVIHEQVNHRWEVC
Splooce       ----------------------------------------------------------------------------------------------------

Uniprot       LTMSEKGFQQISFVNSIATSKGGRHVDYVADQIVTKLVDVVKKKNKGGVAVKAHQVKNHMWIFVNALIENPTFDSQTKENMTLQPKSFGSTCQLSEKFIK
Splooce       ----------------------------------------------------------------------------------------------------

Uniprot       AAIGCGIVESILNWVKFKAQVQLNKKCSAVKHNRIKGIPKLDDANDAGGRNSTECTLILTEGDSAKTLAVSGLGVVGRDKYGVFPLRGKILNVREASHKQ
Splooce       ----------------------------------------------------------------------------------------------------

Uniprot       IMENAEINNIIKIVGLQYKKNYEDEDSLKTLRYGKIMIMTDQDQDGSHIKGLLINFIHHNWPSLLRHRFLEEFITPIVKVSKNKQEMAFYSLPEFEEWKS
Splooce       ----------------------------------------------------------------------------------------------------

Uniprot       STPNHKKWKVKYYKGLGTSTSKEAKEYFADMKRHRIQFKYSGPEDDAAISLAFSKKQIDDRKEWLTNFMEDRRQRKLLGLPEDYLYGQTTTYLTYNDFIN
Splooce       ----------------------------------------------------------------------------------------------------

Uniprot       KELILFSNSDNERSIPSMVDGLKPGQRKVLFTCFKRNDKREVKVAQLAGSVAEMSSYHHGEMSLMMTIINLAQNFVGSNNLNLLQPIGQFGTRLHGGKDS
Splooce       ----------------------------------------------------------------------------------------------------

Uniprot       ASPRYIFTMLSSLARLLFPPKDDHTLKFLYDDNQRVEPEWYIPIIPMVLINGAEGIGTGWSCKIPNFDVREIVNNIRRLMDGEEPLPMLPSYKNFKGTIE
Splooce       ----------------------------------------------MVLINGAEGIGTGWSCKIPNFDVREIVNNIRRLMDGEEPLPMLPSYKNFKGTIE

Uniprot       ELAPNQYVISGEVAILNSTTIEISELPVRTWTQTYKEQVLEPMLNGTEKTPPLITDYREYHTDTTVKFVVKMTEEKLAEAERVGLHKVFKLQTSLTCNSM
Splooce       ELAPNQYVISGEVAILNSTTIEISELPVRTWTQTYKEQVLEPMLNGTEKTPPLITDYREYHTDTTVKFVVKMTEEKLAEAERVGLHKVFKLQTSLTCNSM

Uniprot       VLFDHVGCLKKYDTVLDILRDFFELRLKYYGLRKEWLLGMLGAESAKLNNQARFILEKIDGKIIIENKPKKELIKVLIQRGYDSDPVKAWKEAQQKVPDE
Splooce       VLFDHVGCLKKYDTVLDILRDFFELRLKYYGLRKEWLLGMLGAESAKLNNQARFILEKIDGKIIIENKPKKELIKVLIQRGYDSDPVKAWKEAQQKVPDE

Uniprot       EENEESDNEKETEKSDSVTDSGPTFNYLLDMPLWYLTKEKKDELCRLRNEKEQELDTLKRKSPSDLWKEDLATFIEELEAVEAKEKQDEQVGLPGKGGKA
Splooce       EENEESDNEKETEKSDSVTDSGPTFNYLLDMPLWYLTKEKKDELCRLRNEKEQELDTLKRKSPSDLWKEDLATFIEELEAVEAKEKQDEQVGLPGKGGKA

Uniprot       KGKKTQMAEVLPSPRGQRVIPRITIEMKAEAEKKNKKKIKNENTEGSPQEDGVELEGLKQRLEKKQKREPGTKTKKQTTLAFKPIKKGKKRNPWSDSESD
Splooce       KGKKTQMAEVLPSPRGQRVIPRITIEMKAEAEKKNKKKIKNENTEGSPQEDGVELEGLKQRLEKKQKREPGTKTKKQTTLAFKPIKKGKKRNPWSDSESD

Uniprot       RSSDESNFDVPPRETEPRRAATKTKFTMDLDSDEDFSDFDEKTDDEDFVPSDASPPKTKTSPKLSNKELKPQKSVVSDLEADDVKGSVPLSSSPPATHFP
Splooce       RSSDESNFDVPPRETEPRRAATKTKFTMDLDSDEDFSDFDEKTDDEDFVPSDASPPKTKTSPKLSNKELKPQKSVVSDLEADDVKGSVPLSSSPPATHFP

Uniprot       DETEITNPVPKKNVTVKKTAAKSQSSTSTTGAKKRAAPKGTKRDPALNSGVSQKPDPAKTKNRRKRKPSTSDDSDSNFEKIVSKAVTSKKSKGESDDFHM
Splooce       DETEITNPVPKKNVTVKKTAAKSQSSTSTTGAKKRAAPKGTKRDPALNSGVSQKPDPAKTKNRRKRKPSTSDDSDSNFEKIVSKAVTSKKSKGESDDFHM

Uniprot       DFDSAVAPRAKSVRAKKPIKYLEESDEDDLF
Splooce       DFDSAVAPRAKSVRAKKPIKYLEESDEDDLF

----------------------------------------------------------------------------------------------------

P21980 (Uniprot)	versus
NM_004613#(f-:20_T5375357547045) (Splooce)

For more details about the Alternative Splicing Event -> Link to Splooce page

Peptides that support the ASE (Splooce-specific):
SWIGSVDILR (MAXQUANT)

Alignment:
Uniprot       MAEELVLERCDLELETNGRDHHTADLCREKLVVRRGQPFWLTLHFEGRNYEASVDSLTFSVVTGPAPSQEAGTKARFPLRDAVEEGDWTATVVDQQDCTL
Splooce       ----------------------------------------------------------------------------------------------------

Uniprot       SLQLTTPANAPIGLYRLSLEASTGYQGSSFVLGHFILLFNAWCPADAVYLDSEEERQEYVLTQQGFIYQGSAKFIKNIPWNFGQFEDGILDICLILLDVN
Splooce       ----------------------------------------------------------------------------------------------------

Uniprot       PKFLKNAGRDCSRRSSPVYVGRVVSGMVNCNDDQGVLLGRWDNNYGDGVSPMSWIGSVDILRRWKNHGCQRVKYGQCWVFAAVACTVLRCLGIPTRVVTN
Splooce       ---------------------------------------------------MSWIGSVDILRRWKNHGCQRVKYGQCWVFAAVACTVLRCLGIPTRVVTN

Uniprot       YNSAHDQNSNLLIEYFRNEFGEIQGDKSEMIWNFHCWVESWMTRPDLQPGYEGWQALDPTPQEKSEGTYCCGPVPVRAIKEGDLSTKYDAPFVFAEVNAD
Splooce       YNSAHDQNSNLLIEYFRNEFGEIQGDKSEMIWNFHCWVESWMTRPDLQPGYEGWQALDPTPQEKSEGTYCCGPVPVRAIKEGDLSTKYDAPFVFAEVNAD

Uniprot       VVDWIQQDDGSVHKSINRSLIVGLKISTKSVGRDEREDITHTYKYPEGSSEEREAFTRANHLNKLAEKEETGMAMRIRVGQSMNMGSDFDVFAHITNNTA
Splooce       VVDWIQQDDGSVHKSINRSLIVGLKISTKSVGRDEREDITHTYKYPEGSSEEREAFTRANHLNKLAEKEETGMAMRIRVGQSMNMGSDFDVFAHITNNTA

Uniprot       EEYVCRLLLCARTVSYNGILGPECGTKYLLNLNLEPFSEKSVPLCILYEKYRDCLTESNLIKVRALLVEPVINSYLLAERDLYLENPEIKIRILGEPKQK
Splooce       EEYVCRLLLCARTVSYNGILGPECGTKYLLNLNLEPFSEKSVPLCILYEKYRDCLTESNLIKVRALLVEPVINSYLLAERDLYLENPEIKIRILGEPKQK

Uniprot       RKLVAEVSLQNPLPVALEGCTFTVEGAGLTEEQKTVEIPDPVEAGEEVKVRMDLLPLHMGLHKLVVNFESDKLKAVKGFRNVIIGPA
Splooce       RKLVAEVSLQNPLPVALEGCTFTVEGAGLTEEQKTVEIPDPVEAGEEVKVRMDLLPLHMGLHKLVVNFESDKLKAVKGFRNVIIGPA

----------------------------------------------------------------------------------------------------

UNIPROT? (Uniprot)	versus
NM_033667#(-s-:10_I8890540060622) (Splooce)

For more details about the Alternative Splicing Event -> Link to Splooce page

Peptides that support the ASE (Splooce-specific):
MYTMSHYYDYPSIAHLVQK (MAXQUANT)

Alignment:
Uniprot       -------
Splooce       ----------------------------------------------------------------------------------------------------

Uniprot       -------
Splooce       ----------------------------------------------------------------------------------------------------

Uniprot       -------
Splooce       ----------------------------------------------------------------------------------------------------

Uniprot       -------
Splooce       ------MYTMSHYYDYPSIAHLVQKLSENNIQTIFAVTEEFQPVYKELKNLIPKSAVGTLSANSSNVIQLIIDAYNSLSSEVILENGKLSEGVTISYKSY

Uniprot       -------
Splooce       CKNGVNGTGENGRKCSNISIGDEVQFEISITSNKCPKKDSDSFKIRPLGFTEEVEVILQYICECECQSEGIPESPKCHEGNGTFECGACRCNEGRVGRHC

Uniprot       -------
Splooce       ECSTDEVNSEDMDAYCRKENSSEICSNNGECVCGQCVCRKRDNTNEIYSGKFCECDNFNCDRSNGLICGGNGVCKCRVCECNPNYTGSACDCSLDTSTCE

Uniprot       -------
Splooce       ASNGQICNGRGICECGVCKCTDPKFQGQTCEMCQTCLGVCAEHKECVQCRAFNKGEKKDTCTQECSYFNITKVESRDKLPQPVQPDPVSHCKEKDVDDCW

Uniprot       -------
Splooce       FYFTYSVNGNNEVMVHVVENPECPTGPDIIPIVAGVVAGIVLIGLALLLIWKLLMIIHDRREFAKFEKEKMNAKWDTSLSVAQPGVQWCDISSLQPLTSR

Uniprot       -------
Splooce       FQQFSCLSLPSTWDYRVKILFIRVP

----------------------------------------------------------------------------------------------------

O75083 (Uniprot)	versus
NM_005112#(-t:4_W7727852654191) (Splooce)

For more details about the Alternative Splicing Event -> Link to Splooce page

Peptides that support the ASE (Splooce-specific):
GSTVLDQQLGCLWQK (MAXQUANT)

Alignment:
Uniprot       MPYEIKKVFASLPQVERGVSKIIGGDPKGNNFLYTNGKCVILRNIDDHSRFVNCVRFSPDGNRFATASADGQIYIYDGKTGEKVCALGGSKAHDGGIYAI
Splooce       ----------------------------------------------------------------------------------------------------

Uniprot       SWSPDSTHLLSASGDKTSKIWDVSVNSVVSTFPMGSTVLDQQLGCLWQKDHLLSVSLSGYINYLDRNNPSKPLHVIKGHSKSIQCLTVHKNGGKSYIYSG
Splooce       ---------------------------------MGSTVLDQQLGCLWQKDHLLSVSLSGYINYLDRNNPSKPLHVIKGHSKSIQCLTVHKNGGKSYIYSG

Uniprot       SHDGHINYWDSETGENDSFAGKGHTNQVSRMTVDESGQLISCSMDDTVRYTSLMLRDYSGQGVVKLDVQPKCVAVGPGGYAVVVCIGQIVLLKDQRKCFS
Splooce       SHDGHINYWDSETGENDSFAGKGHTNQVSRMTVDESGQLISCSMDDTVRYTSLMLRDYSGQGVVKLDVQPKCVAVGPGGYAVVVCIGQIVLLKDQRKCFS

Uniprot       IDNPGYEPEVVAVHPGGDTVAIGGVDGNVRLYSILGTTLKDEGKLLEAKGPVTDVAYSHDGAFLAVCDASKVVTVFSVADGYSENNVFYGHHAKIVCLAW
Splooce       IDNPGYEPEVVAVHPGGDTVAIGGVDGNVRLYSILGTTLKDEGKLLEAKGPVTDVAYSHDGAFLAVCDASKVVTVFSVADGYSENNVFYGHHAKIVCLAW

Uniprot       SPDNEHFASGGMDMMVYVWTLSDPETRVKIQDAHRLHHVSSLAWLDEHTLVTTSHDASVKEWTITY
Splooce       SPDNEHFASGGMDMMVYVWTLSDPETRVKIQDAHRLHHVSSLAWLDEHTLVTTSHDASVKEWTITY

----------------------------------------------------------------------------------------------------

Q3YEC7 (Uniprot)	versus
NM_024718#(-s-:9_C1368148613046) (Splooce)

For more details about the Alternative Splicing Event -> Link to Splooce page

Peptides that support the ASE (Splooce-specific):
VAGFQDDVDLEDQPR (MAXQUANT)

Alignment:
Uniprot       MFSALKKLVGSDQAPGRDKNIPAGLQSMNQALQRRFAKGVQYNMKIVIRGDRNTGKTALWHRLQGRPFVEEYIPTQEIQVTSIHWSYKTTDDIVKVEVWD
Splooce       ----------------------------------------------------------------------------------------------------

Uniprot       VVDKGKCKKRGDGLKMENDPQEAESEMALDAEFLDVYKNCNGVVMMFDITKQWTFNYILRELPKVPTHVPVCVLGNYRDMGEHRVILPDDVRDFIDNLDR
Splooce       ----------------------------------------------------------------------------------------------------

Uniprot       PPGSSYFRYAESSMKNSFGLKYLHKFFNIPFLQLQRETLLRQLETNQLDMDATLEELSVQQETEDQNYGIFLEMMEARSRGHASPLAANGQSPSPGSQSP
Splooce       ----------------------------------------------------------------------------------------------------

Uniprot       VVPAGAVSTGSSSPGTPQPAPQLPLNAAPPSSVPPVPPSEALPPPACPSAPAPRRSIISRLFGTSPATEAAPPPPEPVPAAEGPATVQSVEDFVPDDRLD
Splooce       ----------------------------------------------------------------------------------------------------

Uniprot       RSFLEDTTPARDEKKVGAKAAQQDSDSDGEALGGNPMVAGFQDDVDLEDQPRGSPPLPAGPVPSQDITLSSEEEAEVAAPTKGPAPAPQQCSEPETKWSS
Splooce       ------------------------------------MVAGFQDDVDLEDQPRGSPPLPAGPVPSQDITLSSEEEAEVAAPTKGPAPAPQQCSEPETKWSS

Uniprot       IPASKPRRGTAPTRTAAPPWPGGVSVRTGPEKRSSTRPPAEMEPGKGEQASSSESDPEGPIAAQMLSFVMDDPDFESEGSDTQRRADDFPVRDDPSDVTD
Splooce       IPASKPRRGTAPTRTAAPPWPGGVSVRTGPEKRSSTRPPAEMEPGKGEQASSSESDPEGPIAAQMLSFVMDDPDFESEGSDTQRRADDFPVRDDPSDVTD

Uniprot       EDEGPAEPPPPPKLPLPAFRLKNDSDLFGLGLEEAGPKESSEEGKEGKTPSKEKKKKKKKGKEEEEKAAKKKSKHKKSKDKEEGKEERRRRQQRPPRSRE
Splooce       EDEGPAEPPPPPKLPLPAFRLKNDSDLFGLGLEEAGPKESSEEGKEGKTPSKEKKKKKKKGKEEEEKAAKKKSKHKKSKDKEEGKEERRRRQQRPPRSRE

Uniprot       RTAADELEAFLGGGAPGGRHPGGGDYEEL
Splooce       RTAADELEAFLGGGAPGGRHPGGGDYEEL

----------------------------------------------------------------------------------------------------

P23284 (Uniprot)	versus
NM_000942#(f-:15_P9457867302463) (Splooce)

For more details about the Alternative Splicing Event -> Link to Splooce page

Peptides that support the ASE (Splooce-specific):
IQGGDFTR (MAXQUANT)

Alignment:
Uniprot       MLRLSERNMKVLLAAALIAGSVFFLLLPGPSAADEKKKGPKVTVKVYFDLRIGDEDVGRVIFGLFGKTVPKTVDNFVALATGEKGFGYKNSKFHRVIKDF
Splooce       ----------------------------------------------------------------------------------------------------

Uniprot       MIQGGDFTRGDGTGGKSIYGERFPDENFKLKHYGPGWVSMANAGKDTNGSQFFITTVKTAWLDGKHVVFGKVLEGMEVVRKVESTKTDSRDKPLKDVIIA
Splooce       MIQGGDFTRGDGTGGKSIYGERFPDENFKLKHYGPGWVSMANAGKDTNGSQFFITTVKTAWLDGKHVVFGKVLEGMEVVRKVESTKTDSRDKPLKDVIIA

Uniprot       DCGKIEVEKPFAIAKE
Splooce       DCGKIEVEKPFAIAKE

----------------------------------------------------------------------------------------------------

P42345 (Uniprot)	versus
NM_004958#(-s-s-s-s-:1_F3139024117155) (Splooce)

For more details about the Alternative Splicing Event -> Link to Splooce page

Peptides that support the ASE (Splooce-specific):
TILNLLPR (MAXQUANT)

Alignment:
Uniprot       MLGTGPAAATTAATTSSNVSVLQQFASGLKSRNEETRAKAAKELQHYVTMELREMSQEESTRFYDQLNHHIFELVSSSDANERKGGILAIASLIGVEGGN
Splooce       ----------------------------------------------------------------------------------------------------

Uniprot       ATRIGRFANYLRNLLPSNDPVVMEMASKAIGRLAMAGDTFTAEYVEFEVKRALEWLGADRNEGRRHAAVLVLRELAISVPTFFFQQVQPFFDNIFVAVWD
Splooce       ----------------------------------------------------------------------------------------------------

Uniprot       PKQAIREGAVAALRACLILTTQREPKEMQKPQWYRHTFEEAEKGFDETLAKEKGMNRDDRIHGALLILNELVRISSMEGERLREEMEEITQQQLVHDKYC
Splooce       ----------------------------------------------------------------------------------------------------

Uniprot       KDLMGFGTKPRHITPFTSFQAVQPQQSNALVGLLGYSSHQGLMGFGTSPSPAKSTLVESRCCRDLMEEKFDQVCQWVLKCRNSKNSLIQMTILNLLPRLA
Splooce       -----------------------------------------------------------------------------------------MTILNLLPRLA

Uniprot       AFRPSAFTDTQYLQDTMNHVLSCVKKEKERTAAFQALGLLSVAVRSEFKVYLPRVLDIIRAALPPKDFAHKRQKAMQVDATVFTCISMLARAMGPGIQQD
Splooce       AFRPSAFTDTQYLQDTMNHVLSCVKKEKERTAAFQALGLLSVAVRSEFKVYLPRVLDIIRAALPPKDFAHKRQKAMQVDATVFTCISMLARAMGPGIQQD

Uniprot       IKELLEPMLAVGLSPALTAVLYDLSRQIPQLKKDIQDGLLKMLSLVLMHKPLRHPGMPKGLAHQLASPGLTTLPEASDVGSITLALRTLGSFEFEGHSLT
Splooce       IKELLEPMLAVGLSPALTAVLYDLSRQIPQLKKDIQDGLLKMLSLVLMHKPLRHPGMPKGLAHQLASPGLTTLPEASDVGSITLALRTLGSFEFEGHSLT

Uniprot       QFVRHCADHFLNSEHKEIRMEAARTCSRLLTPSIHLISGHAHVVSQTAVQVVADVLSKLLVVGITDPDPDIRYCVLASLDERFDAHLAQAENLQALFVAL
Splooce       QFVRHCADHFLNSEHKEIRMEAARTCSRLLTPSIHLISGHAHVVSQTAVQVVADVLSKLLVVGITDPDPDIRYCVLASLDERFDAHLAQAENLQALFVAL

Uniprot       NDQVFEIRELAICTVGRLSSMNPAFVMPFLRKMLIQILTELEHSGIGRIKEQSARMLGHLVSNAPRLIRPYMEPILKALILKLKDPDPDPNPGVINNVLA
Splooce       NDQVFEIRELAICTVGRLSSMNPAFVMPFLRKMLIQILTELEHSGIGRIKEQSARMLGHLVSNAPRLIRPYMEPILKALILKLKDPDPDPNPGVINNVLA

Uniprot       TIGELAQVSGLEMRKWVDELFIIIMDMLQDSSLLAKRQVALWTLGQLVASTGYVVEPYRKYPTLLEVLLNFLKTEQNQGTRREAIRVLGLLGALDPYKHK
Splooce       TIGELAQVSGLEMRKWVDELFIIIMDMLQDSSLLAKRQVALWTLGQLVASTGYVVEPYRKYPTLLEVLLNFLKTEQNQGTRREAIRVLGLLGALDPYKHK

Uniprot       VNIGMIDQSRDASAVSLSESKSSQDSSDYSTSEMLVNMGNLPLDEFYPAVSMVALMRIFRDQSLSHHHTMVVQAITFIFKSLGLKCVQFLPQVMPTFLNV
Splooce       VNIGMIDQSRDASAVSLSESKSSQDSSDYSTSEMLVNMGNLPLDEFYPAVSMVALMRIFRDQSLSHHHTMVVQAITFIFKSLGLKCVQFLPQVMPTFLNV

Uniprot       IRVCDGAIREFLFQQLGMLVSFVKSHIRPYMDEIVTLMREFWVMNTSIQSTIILLIEQIVVALGGEFKLYLPQLIPHMLRVFMHDNSPGRIVSIKLLAAI
Splooce       IRVCDGAIREFLFQQLGMLVSFVKSHIRPYMDEIVTLMREFWVMNTSIQSTIILLIEQIVVALGGEFKLYLPQLIPHMLRVFMHDNSPGRIVSIKLLAAI

Uniprot       QLFGANLDDYLHLLLPPIVKLFDAPEAPLPSRKAALETVDRLTESLDFTDYASRIIHPIVRTLDQSPELRSTAMDTLSSLVFQLGKKYQIFIPMVNKVLV
Splooce       QLFGANLDDYLHLLLPPIVKLFDAPEAPLPSRKAALETVDRLTESLDFTDYASRIIHPIVRTLDQSPELRSTAMDTLSSLVFQLGKKYQIFIPMVNKVLV

Uniprot       RHRINHQRYDVLICRIVKGYTLADEEEDPLIYQHRMLRSGQGDALASGPVETGPMKKLHVSTINLQKAWGAARRVSKDDWLEWLRRLSLELLKDSSSPSL
Splooce       RHRINHQRYDVLICRIVKGYTLADEEEDPLIYQHRMLRSGQGDALASGPVETGPMKKLHVSTINLQKAWGAARRVSKDDWLEWLRRLSLELLKDSSSPSL

Uniprot       RSCWALAQAYNPMARDLFNAAFVSCWSELNEDQQDELIRSIELALTSQDIAEVTQTLLNLAEFMEHSDKGPLPLRDDNGIVLLGERAAKCRAYAKALHYK
Splooce       RSCWALAQAYNPMARDLFNAAFVSCWSELNEDQQDELIRSIELALTSQDIAEVTQTLLNLAEFMEHSDKGPLPLRDDNGIVLLGERAAKCRAYAKALHYK

Uniprot       ELEFQKGPTPAILESLISINNKLQQPEAAAGVLEYAMKHFGELEIQATWYEKLHEWEDALVAYDKKMDTNKDDPELMLGRMRCLEALGEWGQLHQQCCEK
Splooce       ELEFQKGPTPAILESLISINNKLQQPEAAAGVLEYAMKHFGELEIQATWYEKLHEWEDALVAYDKKMDTNKDDPELMLGRMRCLEALGEWGQLHQQCCEK

Uniprot       WTLVNDETQAKMARMAAAAAWGLGQWDSMEEYTCMIPRDTHDGAFYRAVLALHQDLFSLAQQCIDKARDLLDAELTAMAGESYSRAYGAMVSCHMLSELE
Splooce       WTLVNDETQAKMARMAAAAAWGLGQWDSMEEYTCMIPRDTHDGAFYRAVLALHQDLFSLAQQCIDKARDLLDAELTAMAGESYSRAYGAMVSCHMLSELE

Uniprot       EVIQYKLVPERREIIRQIWWERLQGCQRIVEDWQKILMVRSLVVSPHEDMRTWLKYASLCGKSGRLALAHKTLVLLLGVDPSRQLDHPLPTVHPQVTYAY
Splooce       EVIQYKLVPERREIIRQIWWERLQGCQRIVEDWQKILMVRSLVVSPHEDMRTWLKYASLCGKSGRLALAHKTLVLLLGVDPSRQLDHPLPTVHPQVTYAY

Uniprot       MKNMWKSARKIDAFQHMQHFVQTMQQQAQHAIATEDQQHKQELHKLMARCFLKLGEWQLNLQGINESTIPKVLQYYSAATEHDRSWYKAWHAWAVMNFEA
Splooce       MKNMWKSARKIDAFQHMQHFVQTMQQQAQHAIATEDQQHKQELHKLMARCFLKLGEWQLNLQGINESTIPKVLQYYSAATEHDRSWYKAWHAWAVMNFEA

Uniprot       VLHYKHQNQARDEKKKLRHASGANITNATTAATTAATATTTASTEGSNSESEAESTENSPTPSPLQKKVTEDLSKTLLMYTVPAVQGFFRSISLSRGNNL
Splooce       VLHYKHQNQARDEKKKLRHASGANITNATTAATTAATATTTASTEGSNSESEAESTENSPTPSPLQKKVTEDLSKTLLMYTVPAVQGFFRSISLSRGNNL

Uniprot       QDTLRVLTLWFDYGHWPDVNEALVEGVKAIQIDTWLQVIPQLIARIDTPRPLVGRLIHQLLTDIGRYHPQALIYPLTVASKSTTTARHNAANKILKNMCE
Splooce       QDTLRVLTLWFDYGHWPDVNEALVEGVKAIQIDTWLQVIPQLIARIDTPRPLVGRLIHQLLTDIGRYHPQALIYPLTVASKSTTTARHNAANKILKNMCE

Uniprot       HSNTLVQQAMMVSEELIRVAILWHEMWHEGLEEASRLYFGERNVKGMFEVLEPLHAMMERGPQTLKETSFNQAYGRDLMEAQEWCRKYMKSGNVKDLTQA
Splooce       HSNTLVQQAMMVSEELIRVAILWHEMWHEGLEEASRLYFGERNVKGMFEVLEPLHAMMERGPQTLKETSFNQAYGRDLMEAQEWCRKYMKSGNVKDLTQA

Uniprot       WDLYYHVFRRISKQLPQLTSLELQYVSPKLLMCRDLELAVPGTYDPNQPIIRIQSIAPSLQVITSKQRPRKLTLMGSNGHEFVFLLKGHEDLRQDERVMQ
Splooce       WDLYYHVFRRISKQLPQLTSLELQYVSPKLLMCRDLELAVPGTYDPNQPIIRIQSIAPSLQVITSKQRPRKLTLMGSNGHEFVFLLKGHEDLRQDERVMQ

Uniprot       LFGLVNTLLANDPTSLRKNLSIQRYAVIPLSTNSGLIGWVPHCDTLHALIRDYREKKKILLNIEHRIMLRMAPDYDHLTLMQKVEVFEHAVNNTAGDDLA
Splooce       LFGLVNTLLANDPTSLRKNLSIQRYAVIPLSTNSGLIGWVPHCDTLHALIRDYREKKKILLNIEHRIMLRMAPDYDHLTLMQKVEVFEHAVNNTAGDDLA

Uniprot       KLLWLKSPSSEVWFDRRTNYTRSLAVMSMVGYILGLGDRHPSNLMLDRLSGKILHIDFGDCFEVAMTREKFPEKIPFRLTRMLTNAMEVTGLDGNYRITC
Splooce       KLLWLKSPSSEVWFDRRTNYTRSLAVMSMVGYILGLGDRHPSNLMLDRLSGKILHIDFGDCFEVAMTREKFPEKIPFRLTRMLTNAMEVTGLDGNYRITC

Uniprot       HTVMEVLREHKDSVMAVLEAFVYDPLLNWRLMDTNTKGNKRSRTRTDSYSAGQSVEILDGVELGEPAHKKTGTTVPESIHSFIGDGLVKPEALNKKAIQI
Splooce       HTVMEVLREHKDSVMAVLEAFVYDPLLNWRLMDTNTKGNKRSRTRTDSYSAGQSVEILDGVELGEPAHKKTGTTVPESIHSFIGDGLVKPEALNKKAIQI

Uniprot       INRVRDKLTGRDFSHDDTLDVPTQVELLIKQATSHENLCQCYIGWCPFW
Splooce       INRVRDKLTGRDFSHDDTLDVPTQVELLIKQATSHENLCQCYIGWCPFW

----------------------------------------------------------------------------------------------------

O14777 (Uniprot)	versus
NM_006101#(-s-s-:18_N4918013285586) (Splooce)

For more details about the Alternative Splicing Event -> Link to Splooce page

Peptides that support the ASE (Splooce-specific):
MSGADSFDEMNAELQSK (MAXQUANT + PEAKS)

Alignment:
Uniprot       MKRSSVSSGGAGRLSMQELRSQDVNKQGLYTPQTKEKPTFGKLSINKPTSERKVSLFGKRTSGHGSRNSQLGIFSSSEKIKDPRPLNDKAFIQQCIRQLC
Splooce       ----------------------------------------------------------------------------------------------------

Uniprot       EFLTENGYAHNVSMKSLQAPSVKDFLKIFTFLYGFLCPSYELPDTKFEEEVPRIFKDLGYPFALSKSSMYTVGAPHTWPHIVAALVWLIDCIKIHTAMKE
Splooce       ----------------------------------------------------------------------------------------------------

Uniprot       SSPLFDDGQPWGEETEDGIMHNKLFLDYTIKCYESFMSGADSFDEMNAELQSKLKDLFNVDAFKLESLEAKNRALNEQIARLEQEREKEPNRLESLRKLK
Splooce       ------------------------------------MSGADSFDEMNAELQSKLKDLFNVDAFKLESLEAKNRALNEQIARLEQEREKEPNRLESLRKLK

Uniprot       ASLQGDVQKYQAYMSNLESHSAILDQKLNGLNEEIARVELECETIKQENTRLQNIIDNQKYSVADIERINHERNELQQTINKLTKDLEAEQQKLWNEELK
Splooce       ASLQGDVQKYQAYMSNLESHSAILDQKLNGLNEEIARVELECETIKQENTRLQNIIDNQKYSVADIERINHERNELQQTINKLTKDLEAEQQKLWNEELK

Uniprot       YARGKEAIETQLAEYHKLARKLKLIPKGAENSKGYDFEIKFNPEAGANCLVKYRAQVYVPLKELLNETEEEINKALNKKMGLEDTLEQLNAMITESKRSV
Splooce       YARGKEAIETQLAEYHKLARKLKLIPKGAENSKGYDFEIKFNPEAGANCLVKYRAQVYVPLKELLNETEEEINKALNKKMGLEDTLEQLNAMITESKRSV

Uniprot       RTLKEEVQKLDDLYQQKIKEAEEEDEKCASELESLEKHKHLLESTVNQGLSEAMNELDAVQREYQLVVQTTTEERRKVGNNLQRLLEMVATHVGSVEKHL
Splooce       RTLKEEVQKLDDLYQQKIKEAEEEDEKCASELESLEKHKHLLESTVNQGLSEAMNELDAVQREYQLVVQTTTEERRKVGNNLQRLLEMVATHVGSVEKHL

Uniprot       EEQIAKVDREYEECMSEDLSENIKEIRDKYEKKATLIKSSEE
Splooce       EEQIAKVDREYEECMSEDLSENIKEIRDKYEKKATLIKSSEE

----------------------------------------------------------------------------------------------------

Q13330 (Uniprot)	versus
NM_004689#(r:14_M8880356997087) (Splooce)

For more details about the Alternative Splicing Event -> Link to Splooce page

Peptides that support the ASE (Splooce-specific):
MSAAAASR (PEAKS)

Alignment:
Uniprot       MAANMYRVGDYVYFENSSSNPYLIRRIEELNKTANGNVEAKVVCFYRRRDISSTLIALADKHATLSVCYKAGPGADNGEEGEIEEEMENPEMVDLPEKLK
Splooce       ----------------------------------------------------------------------------------------------------

Uniprot       HQLRHRELFLSRQLESLPATHIRGKCSVTLLNETESLKSYLEREDFFFYSLVYDPQQKTLLADKGEIRVGNRYQADITDLLKEGEEDGRDQSRLETQVWE
Splooce       ----------------------------------------------------------------------------------------------------

Uniprot       AHNPLTDKQIDQFLVVARSVGTFARALDCSSSVRQPSLHMSAAAASRDITLFHAMDTLHKNIYDISKAISALVPQGGPVLCRDEMEEWSASEANLFEEAL
Splooce       ---------------------------------------MSAAAASRDITLFHAMDTLHKNIYDISKAISALVPQGGPVLCRDEMEEWSASEANLFEEAL

Uniprot       EKYGKDFTDIQQDFLPWKSLTSIIEYYYMWKTTDRYVQQKRLKAAEAESKLKQVYIPNYNKPNPNQISVNNVKAGVVNGTGAPGQSPGAGRACESCYTTQ
Splooce       EKYGKDFTDIQQDFLPWKSLTSIIEYYYMWKTTDRYVQQKRLKAAEAESKLKQVYIPNYNKPNPNQISVNNVKAGVVNGTGAPGQSPGAGRACESCYTTQ

Uniprot       SYQWYSWGPPNMQCRLCASCWTYWKKYGGLKMPTRLDGERPGPNRSNMSPHGLPARSSGSPKFAMKTRQAFYLHTTKLTRIARRLCREILRPWHAARHPY
Splooce       SYQWYSWGPPNMQCRLCASCWTYWKKYGGLKMPTRLDGERPGPNRSNMSPHGLPARSSGSPKFAMKTRQAFYLHTTKLTRIARRLCREILRPWHAARHPY

Uniprot       LPINSAAIKAECTARLPEASQSPLVLKQAVRKPLEAVLRYLETHPRPPKPDPVKSVSSVLSSLTPAKVAPVINNGSPTILGKRSYEQHNGVDGNMKKRLL
Splooce       LPINSAAIKAECTARLPEASQSPLVLKQAVRKPLEAVLRYLETHPRPPKPDPVKSVSSVLSSLTPAKVAPVINNGSPTILGKRSYEQHNGVDGNMKKRLL

Uniprot       MPSRGLANHGQARHMGPSRNLLLNGKSYPTKVRLIRGGSLPPVKRRRMNWIDAPDDVFYMATEETRKIRKLLSSSETKRAARRPYKPIALRQSQALPPRP
Splooce       MPSRGLANHGQARHMGPSRNLLLNGKSYPTKVRLIRGGSLPPVKRRRMNWIDAPDDVFYMATEETRKIRKLLSSSETKRAARRPYKPIALRQSQALPPRP

Uniprot       PPPAPVNDEPIVIED
Splooce       PPPAPVNDEPIVIED

----------------------------------------------------------------------------------------------------

P28066 (Uniprot)	versus
NM_002790#(f-:1_P8191478648082) (Splooce)

For more details about the Alternative Splicing Event -> Link to Splooce page

Peptides that support the ASE (Splooce-specific):
SGLLADAK (PEAKS)

Alignment:
Uniprot       MFLTRSEYDRGVNTFSPEGRLFQVEYAIEAIKLGSTAIGIQTSEGVCLAVEKRITSPLMEPSSIEKIVEIDAHIGCAMSGLIADAKTLIDKARVETQNHW
Splooce       -----------------------------------------------------------------------------MSGLIADAKTLIDKARVETQNHW

Uniprot       FTYNETMTVESVTQAVSNLALQFGEEDADPGAMSRPFGVALLFGGVDEKGPQLFHMDPSGTFVQCDARAIGSASEGAQSSLQEVYHKSMTLKEAIKSSLI
Splooce       FTYNETMTVESVTQAVSNLALQFGEEDADPGAMSRPFGVALLFGGVDEKGPQLFHMDPSGTFVQCDARAIGSASEGAQSSLQEVYHKSMTLKEAIKSSLI

Uniprot       ILKQVMEEKLNATNIELATVQPGQNFHMFTKEELEEVIKDI
Splooce       ILKQVMEEKLNATNIELATVQPGQNFHMFTKEELEEVIKDI

----------------------------------------------------------------------------------------------------

Q14UF6 (Uniprot)	versus
NM_000574#(f-:1_C2635390046555) (Splooce)

For more details about the Alternative Splicing Event -> Link to Splooce page

Peptides that support the ASE (Splooce-specific):
IGEHSIYCTVNNDEGEWSGPPPECR (MAXQUANT)

Alignment:
Uniprot       MTVARPSVPAALPLLGELPRLLLLVLLCLPAVWGDCGLPPDVPNAQPALEGRTSFPEDTVITYKCEESFVKIPGEKDSVICLKGSQWSDIEEFCNRSCEV
Splooce       ----------------------------------------------------------------------------------------------------

Uniprot       PTRLNSASLKQPYITQNYFPVGTVVEYECRPGYRREPSLSPKLTCLQNLKWSTAVEFCKKKSCPNPGEIRNGQIDVPGGILFGATISFSCNTGYKLFGST
Splooce       ----------------------------------------------------------------------------------------------------

Uniprot       SSFCLISGSSVQWSDPLPECREIYCPAPPQIDNGIIQGERDHYGYRQSVTYACNKGFTMIGEHSIYCTVNNDEGEWSGPPPECRGKSLTSKVPPTVQKPT
Splooce       ----------------------------------------------------------MIGEHSIYCTVNNDEGEWSGPPPECRGKSLTSKVPPTVQKPT

Uniprot       TVNVPTTEVSPTSQKTTTKTTTPNAQATRSTPVSRTTKHFHETTPNKGSGTTSGTTRLLSGHTCFTLTGLLGTLVTMGLLT
Splooce       TVNVPTTEVSPTSQKTTTKTTTPNAQATRSTPVSRTTKHFHETTPNKGSGTTSGTTRLLSGHTCFTLTGLLGTLVTMGLLT

----------------------------------------------------------------------------------------------------

Q9BQG0 (Uniprot)	versus
NM_014520#(-s-:17_M1063830032291) (Splooce)

For more details about the Alternative Splicing Event -> Link to Splooce page

Peptides that support the ASE (Splooce-specific):
EEALTEQVAR (MAXQUANT + PEAKS)

Alignment:
Uniprot       MESRDPAQPMSPGEATQSGARPADRYGLLKHSREFLDFFWDIAKPEQETRLAATEKLLEYLRGRPKGSEMKYALKRLITGLGVGRETARPCYSLALAQLL
Splooce       ----------------------------------------------------------------------------------------------------

Uniprot       QSFEDLPLCSILQQIQEKYDLHQVKKAMLRPALFANLFGVLALFQSGRLVKDQEALMKSVKLLQALAQYQNHLQEQPRKALVDILSEVSKATLQEILPEV
Splooce       ----------------------------------------------------------------------------------------------------

Uniprot       LKADLNIILSSPEQLELFLLAQQKVPSKLKKLVGSVNLFSDENVPRLVNVLKMAASSVKKDRKLPAIALDLLRLALKEDKFPRFWKEVVEQGLLKMQFWP
Splooce       ----------------------------------------------------------------------------------------------------

Uniprot       ASYLCFRLLGAALPLLTKEQLHLVMQGDVIRHYGEHVCTAKLPKQFKFAPEMDDYVGTFLEGCQDDPERQLAVLVAFSSVTNQGLPVTPTFWRVVRFLSP
Splooce       ----------------------------------------------------------------------------------------------------

Uniprot       PALQGYVAWLRAMFLQPDLDSLVDFSTNNQKKAQDSSLHMPERAVFRLRKWIIFRLVSIVDSLHLEMEEALTEQVARFCLFHSFFVTKKPTSQIPETKHP
Splooce       ------------------------------------------------------------------MEEALTEQVARFCLFHSFFVTKKPTSQIPETKHP

Uniprot       FSFPLENQAREAVSSAFFSLLQTLSTQFKQAPGQTQGGQPWTYHLVQFADLLLNHSHNVTTVTPFTAQQRQAWDRMLQTLKELEAHSAEARAAAFQHLLL
Splooce       FSFPLENQAREAVSSAFFSLLQTLSTQFKQAPGQTQGGQPWTYHLVQFADLLLNHSHNVTTVTPFTAQQRQAWDRMLQTLKELEAHSAEARAAAFQHLLL

Uniprot       LVGIHLLKSPAESCDLLGDIQTCIRKSLGEKPRRSRTKTIDPQEPPWVEVLVEILLALLAQPSHLMRQVARSVFGHICSHLTPRALQLILDVLNPETSED
Splooce       LVGIHLLKSPAESCDLLGDIQTCIRKSLGEKPRRSRTKTIDPQEPPWVEVLVEILLALLAQPSHLMRQVARSVFGHICSHLTPRALQLILDVLNPETSED

Uniprot       ENDRVVVTDDSDERRLKGAEDKSEEGEDNRSSESEEESEGEESEEEERDGDVDQGFREQLMTVLQAGKALGGEDSENEEELGDEAMMALDQSLASLFAEQ
Splooce       ENDRVVVTDDSDERRLKGAEDKSEEGEDNRSSESEEESEGEESEEEERDGDVDQGFREQLMTVLQAGKALGGEDSENEEELGDEAMMALDQSLASLFAEQ

Uniprot       KLRIQARRDEKNKLQKEKALRRDFQIRVLDLVEVLVTKQPENALVLELLEPLLSIIRRSLRSSSSKQEQDLLHKTARIFTHHLCRARRYCHDLGERAGAL
Splooce       KLRIQARRDEKNKLQKEKALRRDFQIRVLDLVEVLVTKQPENALVLELLEPLLSIIRRSLRSSSSKQEQDLLHKTARIFTHHLCRARRYCHDLGERAGAL

Uniprot       HAQVERLVQQAGRQPDSPTALYHFNASLYLLRVLKGNTAEGCVHETQEKQKAGTDPSHMPTGPQAASCLDLNLVTRVYSTALSSFLTKRNSPLTVPMFLS
Splooce       HAQVERLVQQAGRQPDSPTALYHFNASLYLLRVLKGNTAEGCVHETQEKQKAGTDPSHMPTGPQAASCLDLNLVTRVYSTALSSFLTKRNSPLTVPMFLS

Uniprot       LFSRHPVLCQSLLPILVQHITGPVRPRHQACLLLQKTLSMREVRSCFEDPEWKQLMGQVLAKVTENLRVLGEAQTKAQHQQALSSLELLNVLFRTCKHEK
Splooce       LFSRHPVLCQSLLPILVQHITGPVRPRHQACLLLQKTLSMREVRSCFEDPEWKQLMGQVLAKVTENLRVLGEAQTKAQHQQALSSLELLNVLFRTCKHEK

Uniprot       LTLDLTVLLGVLQGQQQSLQQGAHSTGSSRLHDLYWQAMKTLGVQRPKLEKKDAKEIPSATQSPISKKRKKKGFLPETKKRKKRKSEDGTPAEDGTPAAT
Splooce       LTLDLTVLLGVLQGQQQSLQQGAHSTGSSRLHDLYWQAMKTLGVQRPKLEKKDAKEIPSATQSPISKKRKKKGFLPETKKRKKRKSEDGTPAEDGTPAAT

Uniprot       GGSQPPSMGRKKRNRTKAKVPAQANGTPTTKSPAPGAPTRSPSTPAKSPKLQKKNQKPSQVNGAPGSPTEPAGQKQHQKALPKKGVLGKSPLSALARKKA
Splooce       GGSQPPSMGRKKRNRTKAKVPAQANGTPTTKSPAPGAPTRSPSTPAKSPKLQKKNQKPSQVNGAPGSPTEPAGQKQHQKALPKKGVLGKSPLSALARKKA

Uniprot       RLSLVIRSPSLLQSGAKKKAQVRKAGKP
Splooce       RLSLVIRSPSLLQSGAKKKAQVRKAGKP

----------------------------------------------------------------------------------------------------

O43175 (Uniprot)	versus
NM_006623#(-t:1_P6021118851531) (Splooce)

For more details about the Alternative Splicing Event -> Link to Splooce page

Peptides that support the ASE (Splooce-specific):
NTPNGNSLSAAELTCGMIMCLAR (MAXQUANT)

Alignment:
Uniprot       MAFANLRKVLISDSLDPCCRKILQDGGLQVVEKQNLSKEELIAELQDCEGLIVRSATKVTADVINAAEKLQVVGRAGTGVDNVDLEAATRKGILVMNTPN
Splooce       -----------------------------------------------------------------------------------------------MNTPN

Uniprot       GNSLSAAELTCGMIMCLARQIPQATASMKDGKWERKKFMGTELNGKTLGILGLGRIGREVATRMQSFGMKTIGYDPIISPEVSASFGVQQLPLEEIWPLC
Splooce       GNSLSAAELTCGMIMCLARQIPQATASMKDGKWERKKFMGTELNGKTLGILGLGRIGREVATRMQSFGMKTIGYDPIISPEVSASFGVQQLPLEEIWPLC

Uniprot       DFITVHTPLLPSTTGLLNDNTFAQCKKGVRVVNCARGGIVDEGALLRALQSGQCAGAALDVFTEEPPRDRALVDHENVISCPHLGASTKEAQSRCGEEIA
Splooce       DFITVHTPLLPSTTGLLNDNTFAQCKKGVRVVNCARGGIVDEGALLRALQSGQCAGAALDVFTEEPPRDRALVDHENVISCPHLGASTKEAQSRCGEEIA

Uniprot       VQFVDMVKGKSLTGVVNAQALTSAFSPHTKPWIGLAEALGTLMRAWAGSPKGTIQVITQGTSLKNAGNCLSPAVIVGLLKEASKQADVNLVNAKLLVKEA
Splooce       VQFVDMVKGKSLTGVVNAQALTSAFSPHTKPWIGLAEALGTLMRAWAGSPKGTIQVITQGTSLKNAGNCLSPAVIVGLLKEASKQADVNLVNAKLLVKEA

Uniprot       GLNVTTSHSPAAPGEQGFGECLLAVALAGAPYQAVGLVQGTTPVLQGLNGAVFRPEVPLRRDLPLLLFRTQTSDPAMLPTMIGLLAEAGVRLLSYQTSLV
Splooce       GLNVTTSHSPAAPGEQGFGECLLAVALAGAPYQAVGLVQGTTPVLQGLNGAVFRPEVPLRRDLPLLLFRTQTSDPAMLPTMIGLLAEAGVRLLSYQTSLV

Uniprot       SDGETWHVMGISSLLPSLEAWKQHVTEAFQFHF
Splooce       SDGETWHVMGISSLLPSLEAWKQHVTEAFQFHF

----------------------------------------------------------------------------------------------------

Q96F07 (Uniprot)	versus
NM_001037332#(-s-:5_C9187213009486) (Splooce)

For more details about the Alternative Splicing Event -> Link to Splooce page

Peptides that support the ASE (Splooce-specific):
MFAVLDELK (MAXQUANT)

Alignment:
Uniprot       MKFMYFQRKAIERFCSEVKRLCHAERRKDFVSEAYLLTLGKFINMFAVLDELKNMKCSVKNDHSAYKRAAQFLRKMADPQSIQESQNLSMFLANHNRITQ
Splooce       --------------------------------------------MFAVLDELKNMKCSVKNDHSAYKRAAQFLRKMADPQSIQESQNLSMFLANHNRITQ

Uniprot       CLHQQLEVIPGYEELLADIVNICVDYYENKMYLTPSEKHMLLKVMGFGLYLMDGNVSNIYKLDAKKRINLSKIDKFFKQLQVVPLFGDMQIELARYIKTS
Splooce       CLHQQLEVIPGYEELLADIVNICVDYYENKMYLTPSEKHMLLKVMGFGLYLMDGNVSNIYKLDAKKRINLSKIDKFFKQLQVVPLFGDMQIELARYIKTS

Uniprot       AHYEENKSKWTCTQSSISPQYNICEQMVQIRDDHIRFISELARYSNSEVVTGSGLDSQKSDEEYRELFDLALRGLQLLSKWSAHVMEVYSWKLVHPTDKF
Splooce       AHYEENKSKWTCTQSSISPQYNICEQMVQIRDDHIRFISELARYSNSEVVTGSGLDSQKSDEEYRELFDLALRGLQLLSKWSAHVMEVYSWKLVHPTDKF

Uniprot       CNKDCPGTAEEYERATRYNYTSEEKFAFVEVIAMIKGLQVLMGRMESVFNQAIRNTIYAALQDFAQVTLREPLRQAVRKKKNVLISVLQAIRKTICDWEG
Splooce       CNKDCPGTAEEYERATRYNYTSEEKFAFVEVIAMIKGLQVLMGRMESVFNQAIRNTIYAALQDFAQVTLREPLRQAVRKKKNVLISVLQAIRKTICDWEG

Uniprot       GREPPNDPCLRGEKDPKGGFDIKVPRRAVGPSSTQLYMVRTMLESLIADKSGSKKTLRSSLDGPIVLAIEDFHKQSFFFTHLLNISEALQQCCDLSQLWF
Splooce       GREPPNDPCLRGEKDPKGGFDIKVPRRAVGPSSTQLYMVRTMLESLIADKSGSKKTLRSSLDGPIVLAIEDFHKQSFFFTHLLNISEALQQCCDLSQLWF

Uniprot       REFFLELTMGRRIQFPIEMSMPWILTDHILETKEPSMMEYVLYPLDLYNDSAYYALTKFKKQFLYDEIEAEVNLCFDQFVYKLADQIFAYYKAMAGSVLL
Splooce       REFFLELTMGRRIQFPIEMSMPWILTDHILETKEPSMMEYVLYPLDLYNDSAYYALTKFKKQFLYDEIEAEVNLCFDQFVYKLADQIFAYYKAMAGSVLL

Uniprot       DKRFRAECKNYGVIIPYPPSNRYETLLKQRHVQLLGRSIDLNRLITQRISAAMYKSLDQAISRFESEDLTSIVELEWLLEINRLTHRLLCKHMTLDSFDA
Splooce       DKRFRAECKNYGVIIPYPPSNRYETLLKQRHVQLLGRSIDLNRLITQRISAAMYKSLDQAISRFESEDLTSIVELEWLLEINRLTHRLLCKHMTLDSFDA

Uniprot       MFREANHNVSAPYGRITLHVFWELNFDFLPNYCYNGSTNRFVRTAIPFTQEPQRDKPANVQPYYLYGSKPLNIAYSHIYSSYRNFVGPPHFKTICRLLGY
Splooce       MFREANHNVSAPYGRITLHVFWELNFDFLPNYCYNGSTNRFVRTAIPFTQEPQRDKPANVQPYYLYGSKPLNIAYSHIYSSYRNFVGPPHFKTICRLLGY

Uniprot       QGIAVVMEELLKIVKSLLQGTILQYVKTLIEVMPKICRLPRHEYGSPGILEFFHHQLKDIIEYAELKTDVFQSLREVGNAILFCLLIEQALSQEEVCDLL
Splooce       QGIAVVMEELLKIVKSLLQGTILQYVKTLIEVMPKICRLPRHEYGSPGILEFFHHQLKDIIEYAELKTDVFQSLREVGNAILFCLLIEQALSQEEVCDLL

Uniprot       HAAPFQNILPRVYIKEGERLEVRMKRLEAKYAPLHLVPLIERLGTPQQIAIAREGDLLTKERLCCGLSMFEVILTRIRSYLQDPIWRGPPPTNGVMHVDE
Splooce       HAAPFQNILPRVYIKEGERLEVRMKRLEAKYAPLHLVPLIERLGTPQQIAIAREGDLLTKERLCCGLSMFEVILTRIRSYLQDPIWRGPPPTNGVMHVDE

Uniprot       CVEFHRLWSAMQFVYCIPVGTNEFTAEQCFGDGLNWAGCSIIVLLGQQRRFDLFDFCYHLLKVQRQDGKDEIIKNVPLKKMADRIRKYQILNNEVFAILN
Splooce       CVEFHRLWSAMQFVYCIPVGTNEFTAEQCFGDGLNWAGCSIIVLLGQQRRFDLFDFCYHLLKVQRQDGKDEIIKNVPLKKMADRIRKYQILNNEVFAILN

Uniprot       KYMKSVETDSSTVEHVRCFQPPIHQSLATTC
Splooce       KYMKSVETDSSTVEHVRCFQPPIHQSLATTC

----------------------------------------------------------------------------------------------------

P08238 (Uniprot)	versus
NM_007355#(-s-s-s-s-s-s-s-:6_H1608933338680) (Splooce)

For more details about the Alternative Splicing Event -> Link to Splooce page

Peptides that support the ASE (Splooce-specific):
TEPLDEYCVQQLK (PEAKS)
TEPIDEYCVQQLK (MAXQUANT)
MTEPLDEYCVQQLK (PEAKS)
MTEPIDEYCVQQLK (MAXQUANT)

Alignment:
Uniprot       MPEEVHHGEEEVETFAFQAEIAQLMSLIINTFYSNKEIFLRELISNASDALDKIRYESLTDPSKLDSGKELKIDIIPNPQERTLTLVDTGIGMTKADLIN
Splooce       ----------------------------------------------------------------------------------------------------

Uniprot       NLGTIAKSGTKAFMEALQAGADISMIGQFGVGFYSAYLVAEKVVVITKHNDDEQYAWESSAGGSFTVRADHGEPIGRGTKVILHLKEDQTEYLEERRVKE
Splooce       ----------------------------------------------------------------------------------------------------

Uniprot       VVKKHSQFIGYPITLYLEKEREKEISDDEAEEEKGEKEEEDKDDEEKPKIEDVGSDEEDDSGKDKKKKTKKIKEKYIDQEELNKTKPIWTRNPDDITQEE
Splooce       ----------------------------------------------------------------------------------------------------

Uniprot       YGEFYKSLTNDWEDHLAVKHFSVEGQLEFRALLFIPRRAPFDLFENKKKKNNIKLYVRRVFIMDSCDELIPEYLNFIRGVVDSEDLPLNISREMLQQSKI
Splooce       ----------------------------------------------------------------------------------------------------

Uniprot       LKVIRKNIVKKCLELFSELAEDKENYKKFYEAFSKNLKLGIHEDSTNRRRLSELLRYHTSQSGDEMTSLSEYVSRMKETQKSIYYITGESKEQVANSAFV
Splooce       ----------------------------------------------------------------------------------------------------

Uniprot       ERVRKRGFEVVYMTEPIDEYCVQQLKEFDGKSLVSVTKEGLELPEDEEEKKKMEESKAKFENLCKLMKEILDKKVEKVTISNRLVSSPCCIVTSTYGWTA
Splooce       ------------MTEPIDEYCVQQLKEFDGKSLVSVTKEGLELPEDEEEKKKMEESKAKFENLCKLMKEILDKKVEKVTISNRLVSSPCCIVTSTYGWTA

Uniprot       NMERIMKAQALRDNSTMGYMMAKKHLEINPDHPIVETLRQKAEADKNDKAVKDLVVLLFETALLSSGFSLEDPQTHSNRIYRMIKLGLGIDEDEVAAEEP
Splooce       NMERIMKAQALRDNSTMGYMMAKKHLEINPDHPIVETLRQKAEADKNDKAVKDLVVLLFETALLSSGFSLEDPQTHSNRIYRMIKLGLGIDEDEVAAEEP

Uniprot       NAAVPDEIPPLEGDEDASRMEEVD
Splooce       NAAVPDEIPPLEGDEDASRMEEVD

----------------------------------------------------------------------------------------------------

P61978 (Uniprot)	versus
NM_031262#(-s-:9_H287376056818) (Splooce)

For more details about the Alternative Splicing Event -> Link to Splooce page

Peptides that support the ASE (Splooce-specific):
EEEQAFK (MAXQUANT)
MEEEQAFK (MAXQUANT + PEAKS)
EEEQAFKR (MAXQUANT)

Alignment:
Uniprot       METEQPEETFPNTETNGEFGKRPAEDMEEEQAFKRSRNTDEMVELRILLQSKNAGAVIGKGGKNIKALRTDYNASVSVPDSSGPERILSISADIETIGEI
Splooce       --------------------------MEEEQAFKRSRNTDEMVELRILLQSKNAGAVIGKGGKNIKALRTDYNASVSVPDSSGPERILSISADIETIGEI

Uniprot       LKKIIPTLEEGLQLPSPTATSQLPLESDAVECLNYQHYKGSDFDCELRLLIHQSLAGGIIGVKGAKIKELRENTQTTIKLFQECCPHSTDRVVLIGGKPD
Splooce       LKKIIPTLEEGLQLPSPTATSQLPLESDAVECLNYQHYKGSDFDCELRLLIHQSLAGGIIGVKGAKIKELRENTQTTIKLFQECCPHSTDRVVLIGGKPD

Uniprot       RVVECIKIILDLISESPIKGRAQPYDPNFYDETYDYGGFTMMFDDRRGRPVGFPMRGRGGFDRMPPGRGGRPMPPSRRDYDDMSPRRGPPPPPPGRGGRG
Splooce       RVVECIKIILDLISESPIKGRAQPYDPNFYDETYDYGGFTMMFDDRRGRPVGFPMRGRGGFDRMPPGRGGRPMPPSRRDYDDMSPRRGPPPPPPGRGGRG

Uniprot       GSRARNLPLPPPPPPRGGDLMAYDRRGRPGDRYDGMVGFSADETWDSAIDTWSPSEWQMAYEPQVEYHSYYSYAGGRGSYGDLGGPIITTQVTIPKDLAG
Splooce       GSRARNLPLPPPPPPRGGDLMAYDRRGRPGDRYDGMVGFSADETWDSAIDTWSPSEWQMAYEPQVEYHSYYSYAGGRGSYGDLGGPIITTQVTIPKDLAG

Uniprot       SIIGKGGQRIKQIRHESGASIKIDEPLEGSEDRIITITGTQDQIQNAQYLLQNSVKQYSGKFF
Splooce       SIIGKGGQRIKQIRHESGASIKIDEPLEGSEDRIITITGTQDQIQNAQYLLQNSVKQYSGKFF

----------------------------------------------------------------------------------------------------

P50395 (Uniprot)	versus
NM_001494#(-s-:10_G5023519291992) (Splooce)

For more details about the Alternative Splicing Event -> Link to Splooce page

Peptides that support the ASE (Splooce-specific):
MLNKPIEEIIVQNGK (MAXQUANT)

Alignment:
Uniprot       MNEEYDVIVLGTGLTECILSGIMSVNGKKVLHMDRNPYYGGESASITPLEDLYKRFKIPGSPPESMGRGRDWNVDLIPKFLMANGQLVKMLLYTEVTRYL
Splooce       ----------------------------------------------------------------------------------------------------

Uniprot       DFKVTEGSFVYKGGKIYKVPSTEAEALASSLMGLFEKRRFRKFLVYVANFDEKDPRTFEGIDPKKTTMRDVYKKFDLGQDVIDFTGHALALYRTDDYLDQ
Splooce       ----------------------------------------------------------------------------------------------------

Uniprot       PCYETINRIKLYSESLARYGKSPYLYPLYGLGELPQGFARLSAIYGGTYMLNKPIEEIIVQNGKVIGVKSEGEIARCKQLICDPSYVKDRVEKVGQVIRV
Splooce       -------------------------------------------------MLNKPIEEIIVQNGKVIGVKSEGEIARCKQLICDPSYVKDRVEKVGQVIRV

Uniprot       ICILSHPIKNTNDANSCQIIIPQNQVNRKSDIYVCMISFAHNVAAQGKYIAIVSTTVETKEPEKEIRPALELLEPIEQKFVSISDLLVPKDLGTESQIFI
Splooce       ICILSHPIKNTNDANSCQIIIPQNQVNRKSDIYVCMISFAHNVAAQGKYIAIVSTTVETKEPEKEIRPALELLEPIEQKFVSISDLLVPKDLGTESQIFI

Uniprot       SRTYDATTHFETTCDDIKNIYKRMTGSEFDFEEMKRKKNDIYGED
Splooce       SRTYDATTHFETTCDDIKNIYKRMTGSEFDFEEMKRKKNDIYGED

----------------------------------------------------------------------------------------------------

P15121 (Uniprot)	versus
NM_001628#(-t:7_A1858427990071) (Splooce)

For more details about the Alternative Splicing Event -> Link to Splooce page

Peptides that support the ASE (Splooce-specific):
EELVDEGLVK (MAXQUANT)
MEELVDEGLVK (MAXQUANT + PEAKS)

Alignment:
Uniprot       MASRLLLNNGAKMPILGLGTWKSPPGQVTEAVKVAIDVGYRHIDCAHVYQNENEVGVAIQEKLREQVVKREELFIVSKLWCTYHEKGLVKGACQKTLSDL
Splooce       ----------------------------------------------------------------------------------------------------

Uniprot       KLDYLDLYLIHWPTGFKPGKEFFPLDESGNVVPSDTNILDTWAAMEELVDEGLVKAIGISNFNHLQVEMILNKPGLKYKPAVNQIECHPYLTQEKLIQYC
Splooce       --------------------------------------------MEELVDEGLVKAIGISNFNHLQVEMILNKPGLKYKPAVNQIECHPYLTQEKLIQYC

Uniprot       QSKGIVVTAYSPLGSPDRPWAKPEDPSLLEDPRIKAIAAKHNKTTAQVLIRFPMQRNLVVIPKSVTPERIAENFKVFDFELSSQDMTTLLSYNRNWRVCA
Splooce       QSKGIVVTAYSPLGSPDRPWAKPEDPSLLEDPRIKAIAAKHNKTTAQVLIRFPMQRNLVVIPKSVTPERIAENFKVFDFELSSQDMTTLLSYNRNWRVCA

Uniprot       LLSCTSHKDYPFHEEF
Splooce       LLSCTSHKDYPFHEEF

----------------------------------------------------------------------------------------------------

Q9NXH9 (Uniprot)	versus
NM_017722#(-s-s-s-s-:19_T3445767374895) (Splooce)

For more details about the Alternative Splicing Event -> Link to Splooce page

Peptides that support the ASE (Splooce-specific):
WAEPIHDLDFVGR (MAXQUANT)

Alignment:
Uniprot       MQGSSLWLSLTFRSARVLSRARFFEWQSPGLPNTAAMENGTGPYGEERPREVQETTVTEGAAKIAFPSANEVFYNPVQEFNRDLTCAVITEFARIQLGAK
Splooce       ----------------------------------------------------------------------------------------------------

Uniprot       GIQIKVPGEKDTQKVVVDLSEQEEEKVELKESENLASGDQPRTAAVGEICEEGLHVLEGLAASGLRSIRFALEVPGLRSVVANDASTRAVDLIRRNVQLN
Splooce       ----------------------------------------------------------------------------------------------------

Uniprot       DVAHLVQPSQADARMLMYQHQRVSERFDVIDLDPYGSPATFLDAAVQAVSEGGLLCVTCTDMAVLAGNSGETCYSKYGAMALKSRACHEMALRIVLHSLD
Splooce       ----------------------------------------------------------------------------------------------------

Uniprot       LRANCYQRFVVPLLSISADFYVRVFVRVFTGQAKVKASASKQALVFQCVGCGAFHLQRLGKASGVPSGRAKFSAACGPPVTPECEHCGQRHQLGGPMWAE
Splooce       ------------------------------------------------------------------------------------------------MWAE

Uniprot       PIHDLDFVGRVLEAVSANPGRFHTSERIRGVLSVITEELPDVPLYYTLDQLSSTIHCNTPSLLQLRSALLHADFRVSLSHACKNAVKTDAPASALWDIMR
Splooce       PIHDLDFVGRVLEAVSANPGRFHTSERIRGVLSVITEELPDVPLYYTLDQLSSTIHCNTPSLLQLRSALLHADFRVSLSHACKNAVKTDAPASALWDIMR

Uniprot       CWEKECPVKRERLSETSPAFRILSVEPRLQANFTIREDANPSSRQRGLKRFQANPEANWGPRPRARPGGKAADEAMEERRRLLQNKRKEPPEDVAQRAAR
Splooce       CWEKECPVKRERLSETSPAFRILSVEPRLQANFTIREDANPSSRQRGLKRFQANPEANWGPRPRARPGGKAADEAMEERRRLLQNKRKEPPEDVAQRAAR

Uniprot       LKTFPCKRFKEGTCQRGDQCCYSHSPPTPRVSADAAPDCPETSNQTPPGPGAAAGPGID
Splooce       LKTFPCKRFKEGTCQRGDQCCYSHSPPTPRVSADAAPDCPETSNQTPPGPGAAAGPGID

----------------------------------------------------------------------------------------------------

P22314 (Uniprot)	versus
NM_153280#(-s-s-s-:X_U6112185564596) (Splooce)

For more details about the Alternative Splicing Event -> Link to Splooce page

Peptides that support the ASE (Splooce-specific):
ILTDSNGEQPLSAMVSMVTK (MAXQUANT)

Alignment:
Uniprot       MSSSPLSKKRRVSGPDPKPGSNCSPAQSVLSEVPSVPTNGMAKNGSEADIDEGLYSRQLYVLGHEAMKRLQTSSVLVSGLRGLGVEIAKNIILGGVKAVT
Splooce       ----------------------------------------------------------------------------------------------------

Uniprot       LHDQGTAQWADLSSQFYLREEDIGKNRAEVSQPRLAELNSYVPVTAYTGPLVEDFLSGFQVVVLTNTPLEDQLRVGEFCHNRGIKLVVADTRGLFGQLFC
Splooce       ----------------------------------------------------------------------------------------------------

Uniprot       DFGEEMILTDSNGEQPLSAMVSMVTKDNPGVVTCLDEARHGFESGDFVSFSEVQGMVELNGNQPMEIKVLGPYTFSICDTSNFSDYIRGGIVSQVKVPKK
Splooce       -----MILTDSNGEQPLSAMVSMVTKDNPGVVTCLDEARHGFESGDFVSFSEVQGMVELNGNQPMEIKVLGPYTFSICDTSNFSDYIRGGIVSQVKVPKK

Uniprot       ISFKSLVASLAEPDFVVTDFAKFSRPAQLHIGFQALHQFCAQHGRPPRPRNEEDAAELVALAQAVNARALPAVQQNNLDEDLIRKLAYVAAGDLAPINAF
Splooce       ISFKSLVASLAEPDFVVTDFAKFSRPAQLHIGFQALHQFCAQHGRPPRPRNEEDAAELVALAQAVNARALPAVQQNNLDEDLIRKLAYVAAGDLAPINAF

Uniprot       IGGLAAQEVMKACSGKFMPIMQWLYFDALECLPEDKEVLTEDKCLQRQNRYDGQVAVFGSDLQEKLGKQKYFLVGAGAIGCELLKNFAMIGLGCGEGGEI
Splooce       IGGLAAQEVMKACSGKFMPIMQWLYFDALECLPEDKEVLTEDKCLQRQNRYDGQVAVFGSDLQEKLGKQKYFLVGAGAIGCELLKNFAMIGLGCGEGGEI

Uniprot       IVTDMDTIEKSNLNRQFLFRPWDVTKLKSDTAAAAVRQMNPHIRVTSHQNRVGPDTERIYDDDFFQNLDGVANALDNVDARMYMDRRCVYYRKPLLESGT
Splooce       IVTDMDTIEKSNLNRQFLFRPWDVTKLKSDTAAAAVRQMNPHIRVTSHQNRVGPDTERIYDDDFFQNLDGVANALDNVDARMYMDRRCVYYRKPLLESGT

Uniprot       LGTKGNVQVVIPFLTESYSSSQDPPEKSIPICTLKNFPNAIEHTLQWARDEFEGLFKQPAENVNQYLTDPKFVERTLRLAGTQPLEVLEAVQRSLVLQRP
Splooce       LGTKGNVQVVIPFLTESYSSSQDPPEKSIPICTLKNFPNAIEHTLQWARDEFEGLFKQPAENVNQYLTDPKFVERTLRLAGTQPLEVLEAVQRSLVLQRP

Uniprot       QTWADCVTWACHHWHTQYSNNIRQLLHNFPPDQLTSSGAPFWSGPKRCPHPLTFDVNNPLHLDYVMAAANLFAQTYGLTGSQDRAAVATFLQSVQVPEFT
Splooce       QTWADCVTWACHHWHTQYSNNIRQLLHNFPPDQLTSSGAPFWSGPKRCPHPLTFDVNNPLHLDYVMAAANLFAQTYGLTGSQDRAAVATFLQSVQVPEFT

Uniprot       PKSGVKIHVSDQELQSANASVDDSRLEELKATLPSPDKLPGFKMYPIDFEKDDDSNFHMDFIVAASNLRAENYDIPSADRHKSKLIAGKIIPAIATTTAA
Splooce       PKSGVKIHVSDQELQSANASVDDSRLEELKATLPSPDKLPGFKMYPIDFEKDDDSNFHMDFIVAASNLRAENYDIPSADRHKSKLIAGKIIPAIATTTAA

Uniprot       VVGLVCLELYKVVQGHRQLDSYKNGFLNLALPFFGFSEPLAAPRHQYYNQEWTLWDRFEVQGLQPNGEEMTLKQFLDYFKTEHKLEITMLSQGVSMLYSF
Splooce       VVGLVCLELYKVVQGHRQLDSYKNGFLNLALPFFGFSEPLAAPRHQYYNQEWTLWDRFEVQGLQPNGEEMTLKQFLDYFKTEHKLEITMLSQGVSMLYSF

Uniprot       FMPAAKLKERLDQPMTEIVSRVSKRKLGRHVRALVLELCCNDESGEDVEVPYVRYTIR
Splooce       FMPAAKLKERLDQPMTEIVSRVSKRKLGRHVRALVLELCCNDESGEDVEVPYVRYTIR

----------------------------------------------------------------------------------------------------

Q9Y490 (Uniprot)	versus
NM_006289#(-s-s-s-s-:9_T4128056644306) (Splooce)

For more details about the Alternative Splicing Event -> Link to Splooce page

Peptides that support the ASE (Splooce-specific):
AGQAPSQEDR (MAXQUANT)

Alignment:
Uniprot       MVALSLKISIGNVVKTMQFEPSTMVYDACRIIRERIPEAPAGPPSDFGLFLSDDDPKKGIWLEAGKALDYYMLRNGDTMEYRKKQRPLKIRMLDGTVKTI
Splooce       ----------------------------------------------------------------------------------------------------

Uniprot       MVDDSKTVTDMLMTICARIGITNHDEYSLVRELMEEKKEEITGTLRKDKTLLRDEKKMEKLKQKLHTDDELNWLDHGRTLREQGVEEHETLLLRRKFFYS
Splooce       ----------------------------------------------------------------------------------------------------

Uniprot       DQNVDSRDPVQLNLLYVQARDDILNGSHPVSFDKACEFAGFQCQIQFGPHNEQKHKAGFLDLKDFLPKEYVKQKGERKIFQAHKNCGQMSEIEAKVRYVK
Splooce       ----------------------------------------------------------------------------------------------------

Uniprot       LARSLKTYGVSFFLVKEKMKGKNKLVPRLLGITKECVMRVDEKTKEVIQEWNLTNIKRWAASPKSFTLDFGDYQDGYYSVQTTEGEQIAQLIAGYIDIIL
Splooce       ----------------------------------------------------------------------------------------------------

Uniprot       KKKKSKDHFGLEGDEESTMLEDSVSPKKSTVLQQQYNRVGKVEHGSVALPAIMRSGASGPENFQVGSMPPAQQQITSGQMHRGHMPPLTSAQQALTGTIN
Splooce       ----------------------------------------------------------------------------------------------------

Uniprot       SSMQAVQAAQATLDDFDTLPPLGQDAASKAWRKNKMDESKHEIHSQVDAITAGTASVVNLTAGDPAETDYTAVGCAVTTISSNLTEMSRGVKLLAALLED
Splooce       ----------------------------------------------------------------------------------------------------

Uniprot       EGGSGRPLLQAAKGLAGAVSELLRSAQPASAEPRQNLLQAAGNVGQASGELLQQIGESDTDPHFQDALMQLAKAVASAAAALVLKAKSVAQRTEDSGLQT
Splooce       ----------------------------------------------------------------------------------------------------

Uniprot       QVIAAATQCALSTSQLVACTKVVAPTISSPVCQEQLVEAGRLVAKAVEGCVSASQAATEDGQLLRGVGAAATAVTQALNELLQHVKAHATGAGPAGRYDQ
Splooce       ----------------------------------------------------------------------------------------------------

Uniprot       ATDTILTVTENIFSSMGDAGEMVRQARILAQATSDLVNAIKADAEGESDLENSRKLLSAAKILADATAKMVEAAKGAAAHPDSEEQQQRLREAAEGLRMA
Splooce       ----------------------------------------------------------------------------------------------------

Uniprot       TNAAAQNAIKKKLVQRLEHAAKQAAASATQTIAAAQHAASTPKASAGPQPLLVQSCKAVAEQIPLLVQGVRGSQAQPDSPSAQLALIAASQSFLQPGGKM
Splooce       ----------------------------------------------------------------------------------------------------

Uniprot       VAAAKASVPTIQDQASAMQLSQCAKNLGTALAELRTAAQKAQEACGPLEMDSALSVVQNLEKDLQEVKAAARDGKLKPLPGETMEKCTQDLGNSTKAVSS
Splooce       ----------------------------------------------------------------------------------------------------

Uniprot       AIAQLLGEVAQGNENYAGIAARDVAGGLRSLAQAARGVAALTSDPAVQAIVLDTASDVLDKASSLIEEAKKAAGHPGDPESQQRLAQVAKAVTQALNRCV
Splooce       ----------------------------------------------------------------------------------------------------

Uniprot       SCLPGQRDVDNALRAVGDASKRLLSDSLPPSTGTFQEAQSRLNEAAAGLNQAATELVQASRGTPQDLARASGRFGQDFSTFLEAGVEMAGQAPSQEDRAQ
Splooce       ---------------------------------------------------------------------------------------MAGQAPSQEDRAQ

Uniprot       VVSNLKGISMSSSKLLLAAKALSTDPAAPNLKSQLAAAARAVTDSINQLITMCTQQAPGQKECDNALRELETVRELLENPVQPINDMSYFGCLDSVMENS
Splooce       VVSNLKGISMSSSKLLLAAKALSTDPAAPNLKSQLAAAARAVTDSINQLITMCTQQAPGQKECDNALRELETVRELLENPVQPINDMSYFGCLDSVMENS

Uniprot       KVLGEAMTGISQNAKNGNLPEFGDAISTASKALCGFTEAAAQAAYLVGVSDPNSQAGQQGLVEPTQFARANQAIQMACQSLGEPGCTQAQVLSAATIVAK
Splooce       KVLGEAMTGISQNAKNGNLPEFGDAISTASKALCGFTEAAAQAAYLVGVSDPNSQAGQQGLVEPTQFARANQAIQMACQSLGEPGCTQAQVLSAATIVAK

Uniprot       HTSALCNSCRLASARTTNPTAKRQFVQSAKEVANSTANLVKTIKALDGAFTEENRAQCRAATAPLLEAVDNLSAFASNPEFSSIPAQISPEGRAAMEPIV
Splooce       HTSALCNSCRLASARTTNPTAKRQFVQSAKEVANSTANLVKTIKALDGAFTEENRAQCRAATAPLLEAVDNLSAFASNPEFSSIPAQISPEGRAAMEPIV

Uniprot       ISAKTMLESAGGLIQTARALAVNPRDPPSWSVLAGHSRTVSDSIKKLITSMRDKAPGQLECETAIAALNSCLRDLDQASLAAVSQQLAPREGISQEALHT
Splooce       ISAKTMLESAGGLIQTARALAVNPRDPPSWSVLAGHSRTVSDSIKKLITSMRDKAPGQLECETAIAALNSCLRDLDQASLAAVSQQLAPREGISQEALHT

Uniprot       QMLTAVQEISHLIEPLANAARAEASQLGHKVSQMAQYFEPLTLAAVGAASKTLSHPQQMALLDQTKTLAESALQLLYTAKEAGGNPKQAAHTQEALEEAV
Splooce       QMLTAVQEISHLIEPLANAARAEASQLGHKVSQMAQYFEPLTLAAVGAASKTLSHPQQMALLDQTKTLAESALQLLYTAKEAGGNPKQAAHTQEALEEAV

Uniprot       QMMTEAVEDLTTTLNEAASAAGVVGGMVDSITQAINQLDEGPMGEPEGSFVDYQTTMVRTAKAIAVTVQEMVTKSNTSPEELGPLANQLTSDYGRLASEA
Splooce       QMMTEAVEDLTTTLNEAASAAGVVGGMVDSITQAINQLDEGPMGEPEGSFVDYQTTMVRTAKAIAVTVQEMVTKSNTSPEELGPLANQLTSDYGRLASEA

Uniprot       KPAAVAAENEEIGSHIKHRVQELGHGCAALVTKAGALQCSPSDAYTKKELIECARRVSEKVSHVLAALQAGNRGTQACITAASAVSGIIADLDTTIMFAT
Splooce       KPAAVAAENEEIGSHIKHRVQELGHGCAALVTKAGALQCSPSDAYTKKELIECARRVSEKVSHVLAALQAGNRGTQACITAASAVSGIIADLDTTIMFAT

Uniprot       AGTLNREGTETFADHREGILKTAKVLVEDTKVLVQNAAGSQEKLAQAAQSSVATITRLADVVKLGAASLGAEDPETQVVLINAVKDVAKALGDLISATKA
Splooce       AGTLNREGTETFADHREGILKTAKVLVEDTKVLVQNAAGSQEKLAQAAQSSVATITRLADVVKLGAASLGAEDPETQVVLINAVKDVAKALGDLISATKA

Uniprot       AAGKVGDDPAVWQLKNSAKVMVTNVTSLLKTVKAVEDEATKGTRALEATTEHIRQELAVFCSPEPPAKTSTPEDFIRMTKGITMATAKAVAAGNSCRQED
Splooce       AAGKVGDDPAVWQLKNSAKVMVTNVTSLLKTVKAVEDEATKGTRALEATTEHIRQELAVFCSPEPPAKTSTPEDFIRMTKGITMATAKAVAAGNSCRQED

Uniprot       VIATANLSRRAIADMLRACKEAAYHPEVAPDVRLRALHYGRECANGYLELLDHVLLTLQKPSPELKQQLTGHSKRVAGSVTELIQAAEAMKGTEWVDPED
Splooce       VIATANLSRRAIADMLRACKEAAYHPEVAPDVRLRALHYGRECANGYLELLDHVLLTLQKPSPELKQQLTGHSKRVAGSVTELIQAAEAMKGTEWVDPED

Uniprot       PTVIAENELLGAAAAIEAAAKKLEQLKPRAKPKEADESLNFEEQILEAAKSIAAATSALVKAASAAQRELVAQGKVGAIPANALDDGQWSQGLISAARMV
Splooce       PTVIAENELLGAAAAIEAAAKKLEQLKPRAKPKEADESLNFEEQILEAAKSIAAATSALVKAASAAQRELVAQGKVGAIPANALDDGQWSQGLISAARMV

Uniprot       AAATNNLCEAANAAVQGHASQEKLISSAKQVAASTAQLLVACKVKADQDSEAMKRLQAAGNAVKRASDNLVKAAQKAAAFEEQENETVVVKEKMVGGIAQ
Splooce       AAATNNLCEAANAAVQGHASQEKLISSAKQVAASTAQLLVACKVKADQDSEAMKRLQAAGNAVKRASDNLVKAAQKAAAFEEQENETVVVKEKMVGGIAQ

Uniprot       IIAAQEEMLRKERELEEARKKLAQIRQQQYKFLPSELRDEH
Splooce       IIAAQEEMLRKERELEEARKKLAQIRQQQYKFLPSELRDEH

----------------------------------------------------------------------------------------------------

E7EUT4 (Uniprot)	versus
NM_002046#(f-T:12_G9072278901721) (Splooce)

For more details about the Alternative Splicing Event -> Link to Splooce page

Peptides that support the ASE (Splooce-specific):
FVMGVNHEK (MAXQUANT + PEAKS)

Alignment:
Uniprot       MGKVKVGVNGFGRIGRLVTRAAFNSGKVDIVAINDPFIDLNYMVYMFQYDSTHGKFHGTVKAENGKLVINGNPITIFQERDPSKIKWGDAGAEYVVESTG
Splooce       ----------------------------------------------------------------------------------------------------

Uniprot       VFTTMEKAGAHLQGGAKRVIISAPSADAPMFVMGVNHEKYDNSLKIISNASCTTNCLAPLAKVIHDNFGIVEGLMTTVHAITATQKTVDGPSGKLWRDGR
Splooce       -----------------------------MFVMGVNHEKYDNSLKIISNASCTTNCLAPLAKVIHDNFGIVEGLMTTVHAITATQKTVDGPSGKLWRDGR

Uniprot       GALQNIIPASTGAAKAVGKVIPELNGKLTGMAFRVPTANVSVVDLTCRLEKPAKYDDIKKVVKQASEGPLKGILGYTEHQVVSSDFNSDTHSSTFDAGAG
Splooce       GALQNIIPASTGAAKAVGKVIPELNGKLTGMAFRVPTANVSVVDLTCRLEKPAKYDDIKKVVKQASEGPLKGILGYTEHQVVSSDFNSDTHSSTFDAGAG

Uniprot       IALNDHFVKLISWYDNEFGYSNRVVDLMAHMASKE
Splooce       IALNDHFVKLISWYDNEFGYSNRVVDLMAHMASKE

----------------------------------------------------------------------------------------------------

Q9H6R4 (Uniprot)	versus
NM_022917#(-s-s-s-s-s-s-s-:9_N3468247428895) (Splooce)

For more details about the Alternative Splicing Event -> Link to Splooce page

Peptides that support the ASE (Splooce-specific):
TVVCHLEGSGQWPQDAEAVQR (MAXQUANT)

Alignment:
Uniprot       MGPAPAGEQLRGATGEPEVMEPALEGTGKEGKKASSRKRTLAEPPAKGLLQPVKLSRAELYKEPTNEELNRLRETEILFHSSLLRLQVEELLKEVRLSEK
Splooce       ----------------------------------------------------------------------------------------------------

Uniprot       KKDRIDAFLREVNQRVVRVPSVPETELTDQAWLPAGVRVPLHQVPYAVKGCFRFLPPAQVTVVGSYLLGTCIRPDINVDVALTMPREILQDKDGLNQRYF
Splooce       ----------------------------------------------------------------------------------------------------

Uniprot       RKRALYLAHLAHHLAQDPLFGSVCFSYTNGCHLKPSLLLRPRGKDERLVTVRLHPCPPPDFFRPCRLLPTKNNVRSAWYRGQSPAGDGSPEPPTPRYNTW
Splooce       ----------------------------------------------------------------------------------------------------

Uniprot       VLQDTVLESHLQLLSTILSSAQGLKDGVALLKVWLRQRELDKGQGGFTGFLVSMLVVFLVSTRKIHTTMSGYQVLRSVLQFLATTDLTVNGISLCLSSDP
Splooce       ----------------------------------------------------------------------------------------------------

Uniprot       SLPALADFHQAFSVVFLDSSGHLNLCADVTASTYHQVQHEARLSMMLLDSRADDGFHLLLMTPKPMIRAFDHVLHLRPLSRLQAACHRLKLWPELQDNGG
Splooce       ----------------------------------------------------------------------------------------------------

Uniprot       DYVSAALGPLTTLLEQGLGARLNLLAHSRPPVPEWDISQDPPKHKDSGTLTLGLLLRPEGLTSVLELGPEADQPEAAKFRQFWGSRSELRRFQDGAIREA
Splooce       ----------------------------------------------------------------------------------------------------

Uniprot       VVWEAASMSQKRLIPHQVVTHLLALHADIPETCVHYVGGPLDALIQGLKETSSTGEEALVAAVRCYDDLSRLLWGLEGLPLTVSAVQGAHPVLRYTEVFP
Splooce       ----------------------------------------------------------------------------------------------------

Uniprot       PTPVRPAFSFYETLRERSSLLPRLDKPCPAYVEPMTVVCHLEGSGQWPQDAEAVQRVRAAFQLRLAELLTQQHGLQCRATATHTDVLKDGFVFRIRVAYQ
Splooce       ----------------------------------MTVVCHLEGSGQWPQDAEAVQRVRAAFQLRLAELLTQQHGLQCRATATHTDVLKDGFVFRIRVAYQ

Uniprot       REPQILKEVQSPEGMISLRDTAASLRLERDTRQLPLLTSALHGLQQQHPAFSGVARLAKRWVRAQLLGEGFADESLDLVAAALFLHPEPFTPPSSPQVGF
Splooce       REPQILKEVQSPEGMISLRDTAASLRLERDTRQLPLLTSALHGLQQQHPAFSGVARLAKRWVRAQLLGEGFADESLDLVAAALFLHPEPFTPPSSPQVGF

Uniprot       LRFLFLVSTFDWKNNPLFVNLNNELTVEEQVEIRSGFLAARAQLPVMVIVTPQDRKNSVWTQDGPSAQILQQLVVLAAEALPMLEKQLMDPRGPGDIRTV
Splooce       LRFLFLVSTFDWKNNPLFVNLNNELTVEEQVEIRSGFLAARAQLPVMVIVTPQDRKNSVWTQDGPSAQILQQLVVLAAEALPMLEKQLMDPRGPGDIRTV

Uniprot       FRPPLDIYDVLIRLSPRHIPRHRQAVDSPAASFCRGLLSQPGPSSLMPVLGYDPPQLYLTQLREAFGDLALFFYDQHGGEVIGVLWKPTSFQPQPFKASS
Splooce       FRPPLDIYDVLIRLSPRHIPRHRQAVDSPAASFCRGLLSQPGPSSLMPVLGYDPPQLYLTQLREAFGDLALFFYDQHGGEVIGVLWKPTSFQPQPFKASS

Uniprot       TKGRMVMSRGGELVMVPNVEAILEDFAVLGEGLVQTVEARSERWTV
Splooce       TKGRMVMSRGGELVMVPNVEAILEDFAVLGEGLVQTVEARSERWTV

----------------------------------------------------------------------------------------------------

O00203 (Uniprot)	versus
NM_003664#(-s-:5_A3852971971467) (Splooce)

For more details about the Alternative Splicing Event -> Link to Splooce page

Peptides that support the ASE (Splooce-specific):
MLIEVIEK (MAXQUANT)

Alignment:
Uniprot       MSSNSFPYNEQSGGGEATELGQEATSTISPSGAFGLFSSDLKKNEDLKQMLESNKDSAKLDAMKRIVGMIAKGKNASELFPAVVKNVASKNIEIKKLVYV
Splooce       ----------------------------------------------------------------------------------------------------

Uniprot       YLVRYAEEQQDLALLSISTFQRALKDPNQLIRASALRVLSSIRVPIIVPIMMLAIKEASADLSPYVRKNAAHAIQKLYSLDPEQKEMLIEVIEKLLKDKS
Splooce       --------------------------------------------------------------------------------------MLIEVIEKLLKDKS

Uniprot       TLVAGSVVMAFEEVCPDRIDLIHKNYRKLCNLLVDVEEWGQVVIIHMLTRYARTQFVSPWKEGDELEDNGKNFYESDDDQKEKTDKKKKPYTMDPDHRLL
Splooce       TLVAGSVVMAFEEVCPDRIDLIHKNYRKLCNLLVDVEEWGQVVIIHMLTRYARTQFVSPWKEGDELEDNGKNFYESDDDQKEKTDKKKKPYTMDPDHRLL

Uniprot       IRNTKPLLQSRNAAVVMAVAQLYWHISPKSEAGIISKSLVRLLRSNREVQYIVLQNIATMSIQRKGMFEPYLKSFYVRSTDPTMIKTLKLEILTNLANEA
Splooce       IRNTKPLLQSRNAAVVMAVAQLYWHISPKSEAGIISKSLVRLLRSNREVQYIVLQNIATMSIQRKGMFEPYLKSFYVRSTDPTMIKTLKLEILTNLANEA

Uniprot       NISTLLREFQTYVKSQDKQFAAATIQTIGRCATNILEVTDTCLNGLVCLLSNRDEIVVAESVVVIKKLLQMQPAQHGEIIKHMAKLLDSITVPVARASIL
Splooce       NISTLLREFQTYVKSQDKQFAAATIQTIGRCATNILEVTDTCLNGLVCLLSNRDEIVVAESVVVIKKLLQMQPAQHGEIIKHMAKLLDSITVPVARASIL

Uniprot       WLIGENCERVPKIAPDVLRKMAKSFTSEDDLVKLQILNLGAKLYLTNSKQTKLLTQYILNLGKYDQNYDIRDRTRFIRQLIVPNVKSGALSKYAKKIFLA
Splooce       WLIGENCERVPKIAPDVLRKMAKSFTSEDDLVKLQILNLGAKLYLTNSKQTKLLTQYILNLGKYDQNYDIRDRTRFIRQLIVPNVKSGALSKYAKKIFLA

Uniprot       QKPAPLLESPFKDRDHFQLGTLSHTLNIKATGYLELSNWPEVAPDPSVRNVEVIELAKEWTPAGKAKQENSAKKFYSESEEEEDSSDSSSDSESESGSES
Splooce       QKPAPLLESPFKDRDHFQLGTLSHTLNIKATGYLELSNWPEVAPDPSVRNVEVIELAKEWTPAGKAKQENSAKKFYSESEEEEDSSDSSSDSESESGSES

Uniprot       GEQGESGEEGDSNEDSSEDSSSEQDSESGRESGLENKRTAKRNSKAKGKSDSEDGEKENEKSKTSDSSNDESSSIEDSSSDSESESEPESESESRRVTKE
Splooce       GEQGESGEEGDSNEDSSEDSSSEQDSESGRESGLENKRTAKRNSKAKGKSDSEDGEKENEKSKTSDSSNDESSSIEDSSSDSESESEPESESESRRVTKE

Uniprot       KEKKTKQDRTPLTKDVSLLDLDDFNPVSTPVALPTPALSPSLMADLEGLHLSTSSSVISVSTPAFVPTKTHVLLHRMSGKGLAAHYFFPRQPCIFGDKMV
Splooce       KEKKTKQDRTPLTKDVSLLDLDDFNPVSTPVALPTPALSPSLMADLEGLHLSTSSSVISVSTPAFVPTKTHVLLHRMSGKGLAAHYFFPRQPCIFGDKMV

Uniprot       SIQITLNNTTDRKIENIHIGEKKLPIGMKMHVFNPIDSLEPEGSITVSMGIDFCDSTQTASFQLCTKDDCFNVNIQPPVGELLLPVAMSEKDFKKEQGVL
Splooce       SIQITLNNTTDRKIENIHIGEKKLPIGMKMHVFNPIDSLEPEGSITVSMGIDFCDSTQTASFQLCTKDDCFNVNIQPPVGELLLPVAMSEKDFKKEQGVL

Uniprot       TGMNETSAVIIAAPQNFTPSVIFQKVVNVANVGAVPSGQDNIHRFAAKTVHSGSLMLVTVELKEGSTAQLIINTEKTVIGSVLLRELKPVLSQG
Splooce       TGMNETSAVIIAAPQNFTPSVIFQKVVNVANVGAVPSGQDNIHRFAAKTVHSGSLMLVTVELKEGSTAQLIINTEKTVIGSVLLRELKPVLSQG

----------------------------------------------------------------------------------------------------

P60709 (Uniprot)	versus
NM_001101#(-t:7_A8744989064738) (Splooce)

For more details about the Alternative Splicing Event -> Link to Splooce page

Peptides that support the ASE (Splooce-specific):
MATAASSSSLEK (PEAKS)
ATAASSSSLEK (PEAKS)

Alignment:
Uniprot       MDDDIAALVVDNGSGMCKAGFAGDDAPRAVFPSIVGRPRHQGVMVGMGQKDSYVGDEAQSKRGILTLKYPIEHGIVTNWDDMEKIWHHTFYNELRVAPEE
Splooce       ----------------------------------------------------------------------------------------------------

Uniprot       HPVLLTEAPLNPKANREKMTQIMFETFNTPAMYVAIQAVLSLYASGRTTGIVMDSGDGVTHTVPIYEGYALPHAILRLDLAGRDLTDYLMKILTERGYSF
Splooce       ----------------------------------------------------------------------------------------------------

Uniprot       TTTAEREIVRDIKEKLCYVALDFEQEMATAASSSSLEKSYELPDGQVITIGNERFRCPEALFQPSFLGMESCGIHETTFNSIMKCDVDIRKDLYANTVLS
Splooce       --------------------------MATAASSSSLEKSYELPDGQVITIGNERFRCPEALFQPSFLGMESCGIHETTFNSIMKCDVDIRKDLYANTVLS

Uniprot       GGTTMYPGIADRMQKEITALAPSTMKIKIIAPPERKYSVWIGGSILASLSTFQQMWISKQEYDESGPSIVHRKCF
Splooce       GGTTMYPGIADRMQKEITALAPSTMKIKIIAPPERKYSVWIGGSILASLSTFQQMWISKQEYDESGPSIVHRKCF

----------------------------------------------------------------------------------------------------

O00560 (Uniprot)	versus
NM_001007069#(-s-:8_S456310070762) (Splooce)

For more details about the Alternative Splicing Event -> Link to Splooce page

Peptides that support the ASE (Splooce-specific):
MGLSLNEEEIRANVAVVSGAPLQGLVAR (MAXQUANT)
MGLSLNEEEIR (MAXQUANT)

Alignment:
Uniprot       MSLYPSLEDLKVDKVIQAQTAFSANPANPAILSEASAPIPHDGNLYPRLYPELSQYMGLSLNEEEIRANVAVVSGAPLQGLVARPSSINYMVAPVTGNDV
Splooce       --------------------------------------------------------MGLSLNEEEIRANVAVVSGAPLQGLVARPSSINYMVAPVTGNDV

Uniprot       GIRRAEIKQGIREVILCKDQDGKIGLRLKSIDNGIFVQLVQANSPASLVGLRFGDQVLQINGENCAGWSSDKAHKVLKQAFGEKITMTIRDRPFERTITM
Splooce       GIRRAEIKQGIREVILCKDQDGKIGLRLKSIDNGIFVQLVQANSPASLVGLRFGDQVLQINGENCAGWSSDKAHKVLKQAFGEKITMTIRDRPFERTITM

Uniprot       HKDSTGHVGFIFKNGKITSIVKDSSAARNGLLTEHNICEINGQNVIGLKDSQIADILSTSGTVVTITIMPAFIFEHIIKRMAPSIMKSLMDHTIPEV
Splooce       HKDSTGHVGFIFKNGKITSIVKDSSAARNGLLTEHNICEINGQNVIGLKDSQIADILSTSGTVVTITIMPAFIFEHIIKRMAPSIMKSLMDHTIPEV

----------------------------------------------------------------------------------------------------

Q8WU68 (Uniprot)	versus
NM_144987#(r:19_U8019407120021) (Splooce)

For more details about the Alternative Splicing Event -> Link to Splooce page

Peptides that support the ASE (Splooce-specific):
NVCDNLGDHLVGNVYVK (MAXQUANT)

Alignment:
Uniprot       MAEYLASIFGTEKDKVNCSFYFKIGVCRHGDRCSRLHNKPTFSQEVFTELQEKYGEIEEMNVCDNLGDHLVGNVYVKFRREEDGERAVAELSNRWFNGQA
Splooce       -----------------------------------------------------------MNVCDNLGDHLVGNVYVKFRREEDGERAVAELSNRWFNGQA

Uniprot       VHGNVPEVASATSCICGPFPRTSRGSSMGGDPGAGHPRGSILATIPERGTIGVPLITGMAASEALAPLPFTPNRDRCSWQDLSSKPPSLSCPILPRLPGS
Splooce       VHGNVPEVASATSCICGPFPRTSRGSSMGGDPGAGHPRGSILATIPERGTIGVPLITGMAASEALAPLPFTPNRDRCSWQDLSSKPPSLSCPILPRLPGS

Uniprot       IM
Splooce       IM

----------------------------------------------------------------------------------------------------

Q15393 (Uniprot)	versus
NM_012426#(-s-:16_S2510845940444) (Splooce)

For more details about the Alternative Splicing Event -> Link to Splooce page

Peptides that support the ASE (Splooce-specific):
DPSGQLNEYTER (MAXQUANT)

Alignment:
Uniprot       MFLYNLTLQRATGISFAIHGNFSGTKQQEIVVSRGKILELLRPDPNTGKVHTLLTVEVFGVIRSLMAFRLTGGTKDYIVVGSDSGRIVILEYQPSKNMFE
Splooce       ----------------------------------------------------------------------------------------------------

Uniprot       KIHQETFGKSGCRRIVPGQFLAVDPKGRAVMISAIEKQKLVYILNRDAAARLTISSPLEAHKANTLVYHVVGVDVGFENPMFACLEMDYEEADNDPTGEA
Splooce       ----------------------------------------------------------------------------------------------------

Uniprot       AANTQQTLTFYELDLGLNHVVRKYSEPLEEHGNFLITVPGGSDGPSGVLICSENYITYKNFGDQPDIRCPIPRRRNDLDDPERGMIFVCSATHKTKSMFF
Splooce       ----------------------------------------------------------------------------------------------------

Uniprot       FLAQTEQGDIFKITLETDEDMVTEIRLKYFDTVPVAAAMCVLKTGFLFVASEFGNHYLYQIAHLGDDDEEPEFSSAMPLEEGDTFFFQPRPLKNLVLVDE
Splooce       ----------------------------------------------------------------------------------------------------

Uniprot       LDSLSPILFCQIADLANEDTPQLYVACGRGPRSSLRVLRHGLEVSEMAVSELPGNPNAVWTVRRHIEDEFDAYIIVSFVNATLVLSIGETVEEVTDSGFL
Splooce       ----------------------------------------------------------------------------------------------------

Uniprot       GTTPTLSCSLLGDDALVQVYPDGIRHIRADKRVNEWKTPGKKTIVKCAVNQRQVVIALTGGELVYFEMDPSGQLNEYTERKEMSADVVCMSLANVPPGEQ
Splooce       -------------------------------------------------------------------MDPSGQLNEYTERKEMSADVVCMSLANVPPGEQ

Uniprot       RSRFLAVGLVDNTVRIISLDPSDCLQPLSMQALPAQPESLCIVEMGGTEKQDELGERGSIGFLYLNIGLQNGVLLRTVLDPVTGDLSDTRTRYLGSRPVK
Splooce       RSRFLAVGLVDNTVRIISLDPSDCLQPLSMQALPAQPESLCIVEMGGTEKQDELGERGSIGFLYLNIGLQNGVLLRTVLDPVTGDLSDTRTRYLGSRPVK

Uniprot       LFRVRMQGQEAVLAMSSRSWLSYSYQSRFHLTPLSYETLEFASGFASEQCPEGIVAISTNTLRILALEKLGAVFNQVAFPLQYTPRKFVIHPESNNLIII
Splooce       LFRVRMQGQEAVLAMSSRSWLSYSYQSRFHLTPLSYETLEFASGFASEQCPEGIVAISTNTLRILALEKLGAVFNQVAFPLQYTPRKFVIHPESNNLIII

Uniprot       ETDHNAYTEATKAQRKQQMAEEMVEAAGEDERELAAEMAAAFLNENLPESIFGAPKAGNGQWASVIRVMNPIQGNTLDLVQLEQNEAAFSVAVCRFSNTG
Splooce       ETDHNAYTEATKAQRKQQMAEEMVEAAGEDERELAAEMAAAFLNENLPESIFGAPKAGNGQWASVIRVMNPIQGNTLDLVQLEQNEAAFSVAVCRFSNTG

Uniprot       EDWYVLVGVAKDLILNPRSVAGGFVYTYKLVNNGEKLEFLHKTPVEEVPAAIAPFQGRVLIGVGKLLRVYDLGKKKLLRKCENKHIANYISGIQTIGHRV
Splooce       EDWYVLVGVAKDLILNPRSVAGGFVYTYKLVNNGEKLEFLHKTPVEEVPAAIAPFQGRVLIGVGKLLRVYDLGKKKLLRKCENKHIANYISGIQTIGHRV

Uniprot       IVSDVQESFIWVRYKRNENQLIIFADDTYPRWVTTASLLDYDTVAGADKFGNICVVRLPPNTNDEVDEDPTGNKALWDRGLLNGASQKAEVIMNYHVGET
Splooce       IVSDVQESFIWVRYKRNENQLIIFADDTYPRWVTTASLLDYDTVAGADKFGNICVVRLPPNTNDEVDEDPTGNKALWDRGLLNGASQKAEVIMNYHVGET

Uniprot       VLSLQKTTLIPGGSESLVYTTLSGGIGILVPFTSHEDHDFFQHVEMHLRSEHPPLCGRDHLSFRSYYFPVKNVIDGDLCEQFNSMEPNKQKNVSEELDRT
Splooce       VLSLQKTTLIPGGSESLVYTTLSGGIGILVPFTSHEDHDFFQHVEMHLRSEHPPLCGRDHLSFRSYYFPVKNVIDGDLCEQFNSMEPNKQKNVSEELDRT

Uniprot       PPEVSKKLEDIRTRYAF
Splooce       PPEVSKKLEDIRTRYAF

----------------------------------------------------------------------------------------------------

P29401 (Uniprot)	versus
NM_001135055#(-s-s-s-:3_T1762488991399) (Splooce)

For more details about the Alternative Splicing Event -> Link to Splooce page

Peptides that support the ASE (Splooce-specific):
MVSLAVGCATR (PEAKS)
MVSIAVGCATR (MAXQUANT)

Alignment:
Uniprot       MESYHKPDQQKLQALKDTANRLRISSIQATTAAGSGHPTSCCSAAEIMAVLFFHTMRYKSQDPRNPHNDRFVLSKGHAAPILYAVWAEAGFLAEAELLNL
Splooce       ----------------------------------------------------------------------------------------------------

Uniprot       RKISSDLDGHPVPKQAFTDVATGSLGQGLGAACGMAYTGKYFDKASYRVYCLLGDGELSEGSVWEAMAFASIYKLDNLVAILDINRLGQSDPAPLQHQMD
Splooce       ----------------------------------------------------------------------------------------------------

Uniprot       IYQKRCEAFGWHAIIVDGHSVEELCKAFGQAKHQPTAIIAKTFKGRGITGVEDKESWHGKPLPKNMAEQIIQEIYSQIQSKKKILATPPQEDAPSVDIAN
Splooce       ----------------------------------------------------------------------------------------------------

Uniprot       IRMPSLPSYKVGDKIATRKAYGQALAKLGHASDRIIALDGDTKNSTFSEIFKKEHPDRFIECYIAEQNMVSIAVGCATRNRTVPFCSTFAAFFTRAFDQI
Splooce       --------------------------------------------------------------------MVSIAVGCATRNRTVPFCSTFAAFFTRAFDQI

Uniprot       RMAAISESNINLCGSHCGVSIGEDGPSQMALEDLAMFRSVPTSTVFYPSDGVATEKAVELAANTKGICFIRTSRPENAIIYNNNEDFQVGQAKVVLKSKD
Splooce       RMAAISESNINLCGSHCGVSIGEDGPSQMALEDLAMFRSVPTSTVFYPSDGVATEKAVELAANTKGICFIRTSRPENAIIYNNNEDFQVGQAKVVLKSKD

Uniprot       DQVTVIGAGVTLHEALAAAELLKKEKINIRVLDPFTIKPLDRKLILDSARATKGRILTVEDHYYEGGIGEAVSSAVVGEPGITVTHLAVNRVPRSGKPAE
Splooce       DQVTVIGAGVTLHEALAAAELLKKEKINIRVLDPFTIKPLDRKLILDSARATKGRILTVEDHYYEGGIGEAVSSAVVGEPGITVTHLAVNRVPRSGKPAE

Uniprot       LLKMFGIDRDAIAQAVRGLITKA
Splooce       LLKMFGIDRDAIAQAVRGLITKA

----------------------------------------------------------------------------------------------------

O00267 (Uniprot)	versus
NM_001111020#(-s-s-s-s-s-s-s-s-:19_S4221793777564) (Splooce)

For more details about the Alternative Splicing Event -> Link to Splooce page

Peptides that support the ASE (Splooce-specific):
SAVITEGVKPTLSELEK (MAXQUANT)

Alignment:
Uniprot       MSDSEDSNFSEEEDSERSSDGEEAEVDEERRSAAGSEKEEEPEDEEEEEEEEEYDEEEEEEDDDRPPKKPRHGGFILDEADVDDEYEDEDQWEDGAEDIL
Splooce       ----------------------------------------------------------------------------------------------------

Uniprot       EKGVCEPSSNIDNVVLDEDRSGARRLQNLWRDQREEELGEYYMKKYAKSSVGETVYGGSDELSDDITQQQLLPGVKDPNLWTVKCKIGEERATAISLMRK
Splooce       ----------------------------------------------------------------------------------------------------

Uniprot       FIAYQFTDTPLQIKSVVAPEHVKGYIYVEAYKQTHVKQAIEGVGNLRLGYWNQQMVPIKEMTDVLKVVKEVANLKPKSWVRLKRGIYKDDIAQVDYVEPS
Splooce       ----------------------------------------------------------------------------------------------------

Uniprot       QNTISLKMIPRIDYDRIKARMSLKDWFAKRKKFKRPPQRLFDAEKIRSLGGDVASDGDFLIFEGNRYSRKGFLFKSFAMSAVITEGVKPTLSELEKFEDQ
Splooce       ------------------------------------------------------------------------------MSAVITEGVKPTLSELEKFEDQ

Uniprot       PEGIDLEVVTESTGKEREHNFQPGDNVEVCEGELINLQGKILSVDGNKITIMPKHEDLKDMLEFPAQELRKYFKMGDHVKVIAGRFEGDTGLIVRVEENF
Splooce       PEGIDLEVVTESTGKEREHNFQPGDNVEVCEGELINLQGKILSVDGNKITIMPKHEDLKDMLEFPAQELRKYFKMGDHVKVIAGRFEGDTGLIVRVEENF

Uniprot       VILFSDLTMHELKVLPRDLQLCSETASGVDVGGQHEWGELVQLDPQTVGVIVRLERETFQVLNMYGKVVTVRHQAVTRKKDNRFAVALDSEQNNIHVKDI
Splooce       VILFSDLTMHELKVLPRDLQLCSETASGVDVGGQHEWGELVQLDPQTVGVIVRLERETFQVLNMYGKVVTVRHQAVTRKKDNRFAVALDSEQNNIHVKDI

Uniprot       VKVIDGPHSGREGEIRHLFRSFAFLHCKKLVENGGMFVCKTRHLVLAGGSKPRDVTNFTVGGFAPMSPRISSPMHPSAGGQRGGFGSPGGGSGGMSRGRG
Splooce       VKVIDGPHSGREGEIRHLFRSFAFLHCKKLVENGGMFVCKTRHLVLAGGSKPRDVTNFTVGGFAPMSPRISSPMHPSAGGQRGGFGSPGGGSGGMSRGRG

Uniprot       RRDNELIGQTVRISQGPYKGYIGVVKDATESTARVELHSTCQTISVDRQRLTTVGSRRPGGMTSTYGRTPMYGSQTPMYGSGSRTPMYGSQTPLQDGSRT
Splooce       RRDNELIGQTVRISQGPYKGYIGVVKDATESTARVELHSTCQTISVDRQRLTTVGSRRPGGMTSTYGRTPMYGSQTPMYGSGSRTPMYGSQTPLQDGSRT

Uniprot       PHYGSQTPLHDGSRTPAQSGAWDPNNPNTPSRAEEEYEYAFDDEPTPSPQAYGGTPNPQTPGYPDPSSPQVNPQYNPQTPGTPAMYNTDQFSPYAAPSPQ
Splooce       PHYGSQTPLHDGSRTPAQSGAWDPNNPNTPSRAEEEYEYAFDDEPTPSPQAYGGTPNPQTPGYPDPSSPQVNPQYNPQTPGTPAMYNTDQFSPYAAPSPQ

Uniprot       GSYQPSPSPQSYHQVAPSPAGYQNTHSPASYHPTPSPMAYQASPSPSPVGYSPMTPGAPSPGGYNPHTPGSGIEQNSSDWVTTDIQVKVRDTYLDTQVVG
Splooce       GSYQPSPSPQSYHQVAPSPAGYQNTHSPASYHPTPSPMAYQASPSPSPVGYSPMTPGAPSPGGYNPHTPGSGIEQNSSDWVTTDIQVKVRDTYLDTQVVG

Uniprot       QTGVIRSVTGGMCSVYLKDSEKVVSISSEHLEPITPTKNNKVKVILGEDREATGVLLSIDGEDGIVRMDLDEQLKILNLRFLGKLLEA
Splooce       QTGVIRSVTGGMCSVYLKDSEKVVSISSEHLEPITPTKNNKVKVILGEDREATGVLLSIDGEDGIVRMDLDEQLKILNLRFLGKLLEA

----------------------------------------------------------------------------------------------------

Q00796 (Uniprot)	versus
NM_003104#(r:15_S560241504221) (Splooce)

For more details about the Alternative Splicing Event -> Link to Splooce page

Peptides that support the ASE (Splooce-specific):
VLGHEASGTVEK (MAXQUANT + PEAKS)

Alignment:
Uniprot       MAAAAKPNNLSLVVHGPGDLRLENYPIPEPGPNEVLLRMHSVGICGSDVHYWEYGRIGNFIVKKPMVLGHEASGTVEKVGSSVKHLKPGDRVAIEPGAPR
Splooce       -----------------------------------------------------------------MVLGHEASGTVEKVGSSVKHLKPGDRVAIEPGAPR

Uniprot       ENDEFCKMGRYNLSPSIFFCATPPDDGNLCRFYKHNAAFCYKLPDNVTFEEGALIEPLSVGIHACRRGGVTLGHKVLVCGAGPIGMVTLLVAKAMGAAQV
Splooce       ENDEFCKMGRYNLSPSIFFCATPPDDGNLCRFYKHNAAFCYKLPDNVTFEEGALIEPLSVGIHACRRGGVTLGHKVLVCGAGPIGMVTLLVAKAMGAAQV

Uniprot       VVTDLSATRLSKAKEIGADLVLQISKESPQEIARKVEGQLGCKPEVTIECTGAEASIQAGIYATRSGGNLVLVGLGSEMTTVPLLHAAIREVDIKGVFRY
Splooce       VVTDLSATRLSKAKEIGADLVLQISKESPQEIARKVEGQLGCKPEVTIECTGAEASIQAGIYATRSGGNLVLVGLGSEMTTVPLLHAAIREVDIKGVFRY

Uniprot       CNTWPVAISMLASKSVNVKPLVTHRFPLEKALEAFETFKKGLGLKIMLKCDPSDQNP
Splooce       CNTWPVAISMLASKSVNVKPLVTHRFPLEKALEAFETFKKGLGLKIMLKCDPSDQNP

----------------------------------------------------------------------------------------------------

P12277 (Uniprot)	versus
NM_001823#(rrrr:14_C5720812776536) (Splooce)

For more details about the Alternative Splicing Event -> Link to Splooce page

Peptides that support the ASE (Splooce-specific):
TVGCVAGDEESYEVFK (MAXQUANT)

Alignment:
Uniprot       MPFSNSHNALKLRFPAEDEFPDLSAHNNHMAKVLTPELYAELRAKSTPSGFTLDDVIQTGVDNPGHPYIMTVGCVAGDEESYEVFKDLFDPIIEDRHGGY
Splooce       ---------------------------------------------------------------------MTVGCVAGDEESYEVFKDLFDPIIEDRHGGY

Uniprot       KPSDEHKTDLNPDNLQ========================GGDDLDPNYVLSSRVRTGRSIRGFCLPPHCSRGERRAIEKLAVEALSSLDGDLAGRYYAL
Splooce       KPSDEHKTDLNPDNLQVRGCGRAGRAGPGSSGAHSRLASQGGDDLDPNYVLSSRVRTGRSIRGFCLPPHCSRGERRAIEKLAVE----------GRGRAG

Uniprot       KSMTEAEQQQLIDDHFLFDKPVSPLLLASGMARDWPDARGIWHNDNKTFLVWVNEEDHLRVISMQKGGNMKEVFTRFCTGLTQIETLFKSKDYEFMWNPH
Splooce       RGAAAAASPSR--------RGPRPLLFTS------PGS-----GSRRRALICARPG------SVSRRDRGTEAQPRAHSGLGPREG--GSWRGVTAWDRR

Uniprot       LGYILTCPSNLGTGLRAGVHIKLPNLGKHEKFSEVLKRLRLQKRGTGGVDTAAVGGVFDVSNADRLGFSEVELVQMVVDGVKLLIEMEQRLEQGQAIDDL
Splooce       PGREDWTPADPGG----------------------WGPLTSPEVGHGGGRVRAAG--WRGGRGSRAS---------------------------------

Uniprot       MPAQK
Splooce       -----

----------------------------------------------------------------------------------------------------

Q14847 (Uniprot)	versus
NM_006148#(-s-:17_L3892802890880) (Splooce)

For more details about the Alternative Splicing Event -> Link to Splooce page

Peptides that support the ASE (Splooce-specific):
VADTPENLR (MAXQUANT + PEAKS)

Alignment:
Uniprot       MNPNCARCGKIVYPTEKVNCLDKFWHKACFHCETCKMTLNMKNYKGYEKKPYCNAHYPKQSFTMVADTPENLRLKQQSELQSQVRYKEEFEKNKGKGFSV
Splooce       ---------------------------------------------------------------MVADTPENLRLKQQSELQSQVRYKEEFEKNKGKGFSV

Uniprot       VADTPELQRIKKTQDQISNIKYHEEFEKSRMGPSGGEGMEPERRDSQDGSSYRRPLEQQQPHHIPTSAPVYQQPQQQPVAQSYGGYKEPAAPVSIQRSAP
Splooce       VADTPELQRIKKTQDQISNIKYHEEFEKSRMGPSGGEGMEPERRDSQDGSSYRRPLEQQQPHHIPTSAPVYQQPQQQPVAQSYGGYKEPAAPVSIQRSAP

Uniprot       GGGGKRYRAVYDYSAADEDEVSFQDGDTIVNVQQIDDGWMYGTVERTGDTGMLPANYVEAI
Splooce       GGGGKRYRAVYDYSAADEDEVSFQDGDTIVNVQQIDDGWMYGTVERTGDTGMLPANYVEAI

----------------------------------------------------------------------------------------------------

P61978 (Uniprot)	versus
NM_002140#(-s-:9_H287376056818) (Splooce)

For more details about the Alternative Splicing Event -> Link to Splooce page

Peptides that support the ASE (Splooce-specific):
EEEQAFKR (MAXQUANT)
MEEEQAFK (MAXQUANT + PEAKS)
EEEQAFK (MAXQUANT)

Alignment:
Uniprot       METEQPEETFPNTETNGEFGKRPAEDMEEEQAFKRSRNTDEMVELRILLQSKNAGAVIGKGGKNIKALRTDYNASVSVPDSSGPERILSISADIETIGEI
Splooce       --------------------------MEEEQAFKRSRNTDEMVELRILLQSKNAGAVIGKGGKNIKALRTDYNASVSVPDSSGPERILSISADIETIGEI

Uniprot       LKKIIPTLEEGLQLPSPTATSQLPLESDAVECLNYQHYKGSDFDCELRLLIHQSLAGGIIGVKGAKIKELRENTQTTIKLFQECCPHSTDRVVLIGGKPD
Splooce       LKKIIPTLEEGLQLPSPTATSQLPLESDAVECLNYQHYKGSDFDCELRLLIHQSLAGGIIGVKGAKIKELRENTQTTIKLFQECCPHSTDRVVLIGGKPD

Uniprot       RVVECIKIILDLISESPIKGRAQPYDPNFYDETYDYGGFTMMFDDRRGRPVGFPMRGRGGFDRMPPGRGGRPMPPSRRDYDDMSPRRGPPPPPPGRGGRG
Splooce       RVVECIKIILDLISESPIKGRAQPYDPNFYDETYDYGGFTMMFDDRRGRPVGFPMRGRGGFDRMPPGRGGRPMPPSRRDYDDMSPRRGPPPPPPGRGGRG

Uniprot       GSRARNLPLPPPPPPRGGDLMAYDRRGRPGDRYDGMVGFSADETWDSAIDTWSPSEWQMAYEPQVEYHSYYSYAGGRGSYGDLGGPIITTQVTIPKDLAG
Splooce       GSRARNLPLPPPPPPRGGDLMAYDRRGRPGDRYDGMVGFSADETWDSAIDTWSPSEWQMAYEPQVEYHSYYSYAGGRGSYGDLGGPIITTQVTIPKDLAG

Uniprot       SIIGKGGQRIKQIRHESGASIKIDEPLEGSEDRIITITGTQDQIQNAQYLLQNSVKQYADVEGF
Splooce       SIIGKGGQRIKQIRHESGASIKIDEPLEGSEDRIITITGTQDQIQNAQYLLQNSVKQYADVEGF

----------------------------------------------------------------------------------------------------

Q8N335 (Uniprot)	versus
NM_015141#(-s-:3_G9116530712845) (Splooce)

For more details about the Alternative Splicing Event -> Link to Splooce page

Peptides that support the ASE (Splooce-specific):
SNLSEAVQDADLLVFVIPHQFIHR (MAXQUANT)

Alignment:
Uniprot       MAAAPLKVCIVGSGNWGSAVAKIIGNNVKKLQKFASTVKMWVFEETVNGRKLTDIINNDHENVKYLPGHKLPENVVAMSNLSEAVQDADLLVFVIPHQFI
Splooce       -----------------------------------------------------------------------------MSNLSEAVQDADLLVFVIPHQFI

Uniprot       HRICDEITGRVPKKALGITLIKGIDEGPEGLKLISDIIREKMGIDISVLMGANIANEVAAEKFCETTIGSKVMENGLLFKELLQTPNFRITVVDDADTVE
Splooce       HRICDEITGRVPKKALGITLIKGIDEGPEGLKLISDIIREKMGIDISVLMGANIANEVAAEKFCETTIGSKVMENGLLFKELLQTPNFRITVVDDADTVE

Uniprot       LCGALKNIVAVGAGFCDGLRCGDNTKAAVIRLGLMEMIAFARIFCKGQVSTATFLESCGVADLITTCYGGRNRRVAEAFARTGKTIEELEKEMLNGQKLQ
Splooce       LCGALKNIVAVGAGFCDGLRCGDNTKAAVIRLGLMEMIAFARIFCKGQVSTATFLESCGVADLITTCYGGRNRRVAEAFARTGKTIEELEKEMLNGQKLQ

Uniprot       GPQTSAEVYRILKQKGLLDKFPLFTAVYQICYESRPVQEMLSCLQSHPEHT
Splooce       GPQTSAEVYRILKQKGLLDKFPLFTAVYQICYESRPVQEMLSCLQSHPEHT

----------------------------------------------------------------------------------------------------

P49792 (Uniprot)	versus
NM_006267#(-s-s-s-:2_R7649204857140) (Splooce)

For more details about the Alternative Splicing Event -> Link to Splooce page

Peptides that support the ASE (Splooce-specific):
LGSGLNSFYDQR (MAXQUANT)

Alignment:
Uniprot       MRRSKADVERYIASVQGSTPSPRQKSMKGFYFAKLYYEAKEYDLAKKYICTYINVQERDPKAHRFLGLLYELEENTDKAVECYRRSVELNPTQKDLVLKI
Splooce       ----------------------------------------------------------------------------------------------------

Uniprot       AELLCKNDVTDGRAKYWLERAAKLFPGSPAIYKLKEQLLDCEGEDGWNKLFDLIQSELYVRPDDVHVNIRLVEVYRSTKRLKDAVAHCHEAERNIALRSS
Splooce       ----------------------------------------------------------------------------------------------------

Uniprot       LEWNSCVVQTLKEYLESLQCLESDKSDWRATNTDLLLAYANLMLLTLSTRDVQESRELLQSFDSALQSVKSLGGNDELSATFLEMKGHFYMHAGSLLLKM
Splooce       ----------------------------------------------------------------------------------------------------

Uniprot       GQHSSNVQWRALSELAALCYLIAFQVPRPKIKLIKGEAGQNLLEMMACDRLSQSGHMLLNLSRGKQDFLKEIVETFANKSGQSALYDALFSSQSPKDTSF
Splooce       ----------------------------------------------------------------------------------------------------

Uniprot       LGSDDIGNIDVREPELEDLTRYDVGAIRAHNGSLQHLTWLGLQWNSLPALPGIRKWLKQLFHHLPHETSRLETNAPESICILDLEVFLLGVVYTSHLQLK
Splooce       ----------------------------------------------------------------------------------------------------

Uniprot       EKCNSHHSSYQPLCLPLPVCKQLCTERQKSWWDAVCTLIHRKAVPGNVAKLRLLVQHEINTLRAQEKHGLQPALLVHWAECLQKTGSGLNSFYDQREYIG
Splooce       -----------------------------------------------------------------------------------MLGSGLNSFYDQREYIG

Uniprot       RSVHYWKKVLPLLKIIKKKNSIPEPIDPLFKHFHSVDIQASEIVEYEEDAHITFAILDAVNGNIEDAVTAFESIKSVVSYWNLALIFHRKAEDIENDALS
Splooce       RSVHYWKKVLPLLKIIKKKNSIPEPIDPLFKHFHSVDIQASEIVEYEEDAHITFAILDAVNGNIEDAVTAFESIKSVVSYWNLALIFHRKAEDIENDALS

Uniprot       PEEQEECKNYLRKTRDYLIKIIDDSDSNLSVVKKLPVPLESVKEMLNSVMQELEDYSEGGPLYKNGSLRNADSEIKHSTPSPTRYSLSPSKSYKYSPKTP
Splooce       PEEQEECKNYLRKTRDYLIKIIDDSDSNLSVVKKLPVPLESVKEMLNSVMQELEDYSEGGPLYKNGSLRNADSEIKHSTPSPTRYSLSPSKSYKYSPKTP

Uniprot       PRWAEDQNSLLKMICQQVEAIKKEMQELKLNSSNSASPHRWPTENYGPDSVPDGYQGSQTFHGAPLTVATTGPSVYYSQSPAYNSQYLLRPAANVTPTKG
Splooce       PRWAEDQNSLLKMICQQVEAIKKEMQELKLNSSNSASPHRWPTENYGPDSVPDGYQGSQTFHGAPLTVATTGPSVYYSQSPAYNSQYLLRPAANVTPTKG

Uniprot       PVYGMNRLPPQQHIYAYPQQMHTPPVQSSSACMFSQEMYGPPALRFESPATGILSPRGDDYFNYNVQQTSTNPPLPEPGYFTKPPIAAHASRSAESKTIE
Splooce       PVYGMNRLPPQQHIYAYPQQMHTPPVQSSSACMFSQEMYGPPALRFESPATGILSPRGDDYFNYNVQQTSTNPPLPEPGYFTKPPIAAHASRSAESKTIE

Uniprot       FGKTNFVQPMPGEGLRPSLPTQAHTTQPTPFKFNSNFKSNDGDFTFSSPQVVTQPPPAAYSNSESLLGLLTSDKPLQGDGYSGAKPIPGGQTIGPRNTFN
Splooce       FGKTNFVQPMPGEGLRPSLPTQAHTTQPTPFKFNSNFKSNDGDFTFSSPQVVTQPPPAAYSNSESLLGLLTSDKPLQGDGYSGAKPIPGGQTIGPRNTFN

Uniprot       FGSKNVSGISFTENMGSSQQKNSGFRRSDDMFTFHGPGKSVFGTPTLETANKNHETDGGSAHGDDDDDGPHFEPVVPLPDKIEVKTGEEDEEEFFCNRAK
Splooce       FGSKNVSGISFTENMGSSQQKNSGFRRSDDMFTFHGPGKSVFGTPTLETANKNHETDGGSAHGDDDDDGPHFEPVVPLPDKIEVKTGEEDEEEFFCNRAK

Uniprot       LFRFDVESKEWKERGIGNVKILRHKTSGKIRLLMRREQVLKICANHYISPDMKLTPNAGSDRSFVWHALDYADELPKPEQLAIRFKTPEEAALFKCKFEE
Splooce       LFRFDVESKEWKERGIGNVKILRHKTSGKIRLLMRREQVLKICANHYISPDMKLTPNAGSDRSFVWHALDYADELPKPEQLAIRFKTPEEAALFKCKFEE

Uniprot       AQSILKAPGTNVAMASNQAVRIVKEPTSHDNKDICKSDAGNLNFEFQVAKKEGSWWHCNSCSLKNASTAKKCVSCQNLNPSNKELVGPPLAETVFTPKTS
Splooce       AQSILKAPGTNVAMASNQAVRIVKEPTSHDNKDICKSDAGNLNFEFQVAKKEGSWWHCNSCSLKNASTAKKCVSCQNLNPSNKELVGPPLAETVFTPKTS

Uniprot       PENVQDRFALVTPKKEGHWDCSICLVRNEPTVSRCIACQNTKSANKSGSSFVHQASFKFGQGDLPKPINSDFRSVFSTKEGQWDCSACLVQNEGSSTKCA
Splooce       PENVQDRFALVTPKKEGHWDCSICLVRNEPTVSRCIACQNTKSANKSGSSFVHQASFKFGQGDLPKPINSDFRSVFSTKEGQWDCSACLVQNEGSSTKCA

Uniprot       ACQNPRKQSLPATSIPTPASFKFGTSETSKTLKSGFEDMFAKKEGQWDCSSCLVRNEANATRCVACQNPDKPSPSTSVPAPASFKFGTSETSKAPKSGFE
Splooce       ACQNPRKQSLPATSIPTPASFKFGTSETSKTLKSGFEDMFAKKEGQWDCSSCLVRNEANATRCVACQNPDKPSPSTSVPAPASFKFGTSETSKAPKSGFE

Uniprot       GMFTKKEGQWDCSVCLVRNEASATKCIACQNPGKQNQTTSAVSTPASSETSKAPKSGFEGMFTKKEGQWDCSVCLVRNEASATKCIACQNPGKQNQTTSA
Splooce       GMFTKKEGQWDCSVCLVRNEASATKCIACQNPGKQNQTTSAVSTPASSETSKAPKSGFEGMFTKKEGQWDCSVCLVRNEASATKCIACQNPGKQNQTTSA

Uniprot       VSTPASSETSKAPKSGFEGMFTKKEGQWDCSVCLVRNEASATKCIACQCPSKQNQTTAISTPASSEISKAPKSGFEGMFIRKGQWDCSVCCVQNESSSLK
Splooce       VSTPASSETSKAPKSGFEGMFTKKEGQWDCSVCLVRNEASATKCIACQCPSKQNQTTAISTPASSEISKAPKSGFEGMFIRKGQWDCSVCCVQNESSSLK

Uniprot       CVACDASKPTHKPIAEAPSAFTLGSEMKLHDSSGSQVGTGFKSNFSEKASKFGNTEQGFKFGHVDQENSPSFMFQGSSNTEFKSTKEGFSIPVSADGFKF
Splooce       CVACDASKPTHKPIAEAPSAFTLGSEMKLHDSSGSQVGTGFKSNFSEKASKFGNTEQGFKFGHVDQENSPSFMFQGSSNTEFKSTKEGFSIPVSADGFKF

Uniprot       GISEPGNQEKKSEKPLENGTGFQAQDISGQKNGRGVIFGQTSSTFTFADLAKSTSGEGFQFGKKDPNFKGFSGAGEKLFSSQYGKMANKANTSGDFEKDD
Splooce       GISEPGNQEKKSEKPLENGTGFQAQDISGQKNGRGVIFGQTSSTFTFADLAKSTSGEGFQFGKKDPNFKGFSGAGEKLFSSQYGKMANKANTSGDFEKDD

Uniprot       DAYKTEDSDDIHFEPVVQMPEKVELVTGEEDEKVLYSQRVKLFRFDAEVSQWKERGLGNLKILKNEVNGKLRMLMRREQVLKVCANHWITTTMNLKPLSG
Splooce       DAYKTEDSDDIHFEPVVQMPEKVELVTGEEDEKVLYSQRVKLFRFDAEVSQWKERGLGNLKILKNEVNGKLRMLMRREQVLKVCANHWITTTMNLKPLSG

Uniprot       SDRAWMWLASDFSDGDAKLEQLAAKFKTPELAEEFKQKFEECQRLLLDIPLQTPHKLVDTGRAAKLIQRAEEMKSGLKDFKTFLTNDQTKVTEEENKGSG
Splooce       SDRAWMWLASDFSDGDAKLEQLAAKFKTPELAEEFKQKFEECQRLLLDIPLQTPHKLVDTGRAAKLIQRAEEMKSGLKDFKTFLTNDQTKVTEEENKGSG

Uniprot       TGAAGASDTTIKPNPENTGPTLEWDNYDLREDALDDSVSSSSVHASPLASSPVRKNLFRFGESTTGFNFSFKSALSPSKSPAKLNQSGTSVGTDEESDVT
Splooce       TGAAGASDTTIKPNPENTGPTLEWDNYDLREDALDDSVSSSSVHASPLASSPVRKNLFRFGESTTGFNFSFKSALSPSKSPAKLNQSGTSVGTDEESDVT

Uniprot       QEEERDGQYFEPVVPLPDLVEVSSGEENEQVVFSHRAKLYRYDKDVGQWKERGIGDIKILQNYDNKQVRIVMRRDQVLKLCANHRITPDMTLQNMKGTER
Splooce       QEEERDGQYFEPVVPLPDLVEVSSGEENEQVVFSHRAKLYRYDKDVGQWKERGIGDIKILQNYDNKQVRIVMRRDQVLKLCANHRITPDMTLQNMKGTER

Uniprot       VWLWTACDFADGERKVEHLAVRFKLQDVADSFKKIFDEAKTAQEKDSLITPHVSRSSTPRESPCGKIAVAVLEETTRERTDVIQGDDVADATSEVEVSST
Splooce       VWLWTACDFADGERKVEHLAVRFKLQDVADSFKKIFDEAKTAQEKDSLITPHVSRSSTPRESPCGKIAVAVLEETTRERTDVIQGDDVADATSEVEVSST

Uniprot       SETTPKAVVSPPKFVFGSESVKSIFSSEKSKPFAFGNSSATGSLFGFSFNAPLKSNNSETSSVAQSGSESKVEPKKCELSKNSDIEQSSDSKVKNLFASF
Splooce       SETTPKAVVSPPKFVFGSESVKSIFSSEKSKPFAFGNSSATGSLFGFSFNAPLKSNNSETSSVAQSGSESKVEPKKCELSKNSDIEQSSDSKVKNLFASF

Uniprot       PTEESSINYTFKTPEKAKEKKKPEDSPSDDDVLIVYELTPTAEQKALATKLKLPPTFFCYKNRPDYVSEEEEDDEDFETAVKKLNGKLYLDGSEKCRPLE
Splooce       PTEESSINYTFKTPEKAKEKKKPEDSPSDDDVLIVYELTPTAEQKALATKLKLPPTFFCYKNRPDYVSEEEEDDEDFETAVKKLNGKLYLDGSEKCRPLE

Uniprot       ENTADNEKECIIVWEKKPTVEEKAKADTLKLPPTFFCGVCSDTDEDNGNGEDFQSELQKVQEAQKSQTEEITSTTDSVYTGGTEVMVPSFCKSEEPDSIT
Splooce       ENTADNEKECIIVWEKKPTVEEKAKADTLKLPPTFFCGVCSDTDEDNGNGEDFQSELQKVQEAQKSQTEEITSTTDSVYTGGTEVMVPSFCKSEEPDSIT

Uniprot       KSISSPSVSSETMDKPVDLSTRKEIDTDSTSQGESKIVSFGFGSSTGLSFADLASSNSGDFAFGSKDKNFQWANTGAAVFGTQSVGTQSAGKVGEDEDGS
Splooce       KSISSPSVSSETMDKPVDLSTRKEIDTDSTSQGESKIVSFGFGSSTGLSFADLASSNSGDFAFGSKDKNFQWANTGAAVFGTQSVGTQSAGKVGEDEDGS

Uniprot       DEEVVHNEDIHFEPIVSLPEVEVKSGEEDEEILFKERAKLYRWDRDVSQWKERGVGDIKILWHTMKNYYRILMRRDQVFKVCANHVITKTMELKPLNVSN
Splooce       DEEVVHNEDIHFEPIVSLPEVEVKSGEEDEEILFKERAKLYRWDRDVSQWKERGVGDIKILWHTMKNYYRILMRRDQVFKVCANHVITKTMELKPLNVSN

Uniprot       NALVWTASDYADGEAKVEQLAVRFKTKEVADCFKKTFEECQQNLMKLQKGHVSLAAELSKETNPVVFFDVCADGEPLGRITMELFSNIVPRTAENFRALC
Splooce       NALVWTASDYADGEAKVEQLAVRFKTKEVADCFKKTFEECQQNLMKLQKGHVSLAAELSKETNPVVFFDVCADGEPLGRITMELFSNIVPRTAENFRALC

Uniprot       TGEKGFGFKNSIFHRVIPDFVCQGGDITKHDGTGGQSIYGDKFEDENFDVKHTGPGLLSMANQGQNTNNSQFVITLKKAEHLDFKHVVFGFVKDGMDTVK
Splooce       TGEKGFGFKNSIFHRVIPDFVCQGGDITKHDGTGGQSIYGDKFEDENFDVKHTGPGLLSMANQGQNTNNSQFVITLKKAEHLDFKHVVFGFVKDGMDTVK

Uniprot       KIESFGSPKGSVCRRITITECGQI
Splooce       KIESFGSPKGSVCRRITITECGQI

----------------------------------------------------------------------------------------------------

Q9Y2X3 (Uniprot)	versus
NM_015934#(f-t:2_N6702330311198) (Splooce)

For more details about the Alternative Splicing Event -> Link to Splooce page

Peptides that support the ASE (Splooce-specific):
CLGLAHSLSR (MAXQUANT)

Alignment:
Uniprot       MLVLFETSVGYAIFKVLNEKKLQEVDSLWKEFETPEKANKIVKLKHFEKFQDTAEALAAFTALMEGKINKQLKKVLKKIVKEAHEPLAVADAKLGGVIKE
Splooce       ----------------------------------------------------------------------------------------------------

Uniprot       KLNLSCIHSPVVNELMRGIRSQMDGLIPGVEPREMAAMCLGLAHSLSRYRLKFSADKVDTMIVQAISLLDDLDKELNNYIMRCREWYGWHFPELGKIISD
Splooce       -------------------------------------MCLGLAHSLSRYRLKFSADKVDTMIVQAISLLDDLDKELNNYIMRCREWYGWHFPELGKIISD

Uniprot       NLTYCKCLQKVGDRKNYASAKLSELLPEEVEAEVKAAAEISMGTEVSEEDICNILHLCTQVIEISEYRTQLYEYLQNRMMAIAPNVTVMVGELVGARLIA
Splooce       NLTYCKCLQKVGDRKNYASAKLSELLPEEVEAEVKAAAEISMGTEVSEEDICNILHLCTQVIEISEYRTQLYEYLQNRMMAIAPNVTVMVGELVGARLIA

Uniprot       HAGSLLNLAKHAASTVQILGAEKALFRALKSRRDTPKYGLIYHASLVGQTSPKHKGKISRMLAAKTVLAIRYDAFGEDSSSAMGVENRAKLEARLRTLED
Splooce       HAGSLLNLAKHAASTVQILGAEKALFRALKSRRDTPKYGLIYHASLVGQTSPKHKGKISRMLAAKTVLAIRYDAFGEDSSSAMGVENRAKLEARLRTLED

Uniprot       RGIRKISGTGKALAKTEKYEHKSEVKTYDPSGDSTLPTCSKKRKIEQVDKEDEITEKKAKKAKIKVKVEEEEEEKVAEEEETSVKKKKKRGKKKHIKEEP
Splooce       RGIRKISGTGKALAKTEKYEHKSEVKTYDPSGDSTLPTCSKKRKIEQVDKEDEITEKKAKKAKIKVKVEEEEEEKVAEEEETSVKKKKKRGKKKHIKEEP

Uniprot       LSEEEPCTSTAIASPEKKKKKKKKRENED
Splooce       LSEEEPCTSTAIASPEKKKKKKKKRENED

----------------------------------------------------------------------------------------------------

P62318 (Uniprot)	versus
NM_004175#(f-:22_S2072082384797) (Splooce)

For more details about the Alternative Splicing Event -> Link to Splooce page

Peptides that support the ASE (Splooce-specific):
SNITVTYR (MAXQUANT)

Alignment:
Uniprot       MSIGVPIKVLHEAEGHIVTCETNTGEVYRGKLIEAEDNMNCQMSNITVTYRDGRVAQLEQVYIRGSKIRFLILPDMLKNAPMLKSMKNKNQGSGAGRGKA
Splooce       ------------------------------------------MSNITVTYRDGRVAQLEQVYIRGSKIRFLILPDMLKNAPMLKSMKNKNQGSGAGRGKA

Uniprot       AILKAQVAARGRGRGMGRGNIFQKRR
Splooce       AILKAQVAARGRGRGMGRGNIFQKRR

----------------------------------------------------------------------------------------------------

P06733 (Uniprot)	versus
NM_001428#(-s-s-s-s-s-:1_E377033599376) (Splooce)

For more details about the Alternative Splicing Event -> Link to Splooce page

Peptides that support the ASE (Splooce-specific):
QEFMILPVGAANFR (MAXQUANT)
MQEFMILPVGAANFR (MAXQUANT)

Alignment:
Uniprot       MSILKIHAREIFDSRGNPTVEVDLFTSKGLFRAAVPSGASTGIYEALELRDNDKTRYMGKGVSKAVEHINKTIAPALVSKKLNVTEQEKIDKLMIEMDGT
Splooce       ----------------------------------------------------------------------------------------------------

Uniprot       ENKSKFGANAILGVSLAVCKAGAVEKGVPLYRHIADLAGNSEVILPVPAFNVINGGSHAGNKLAMQEFMILPVGAANFREAMRIGAEVYHNLKNVIKEKY
Splooce       ----------------------------------------------------------------MQEFMILPVGAANFREAMRIGAEVYHNLKNVIKEKY

Uniprot       GKDATNVGDEGGFAPNILENKEGLELLKTAIGKAGYTDKVVIGMDVAASEFFRSGKYDLDFKSPDDPSRYISPDQLADLYKSFIKDYPVVSIEDPFDQDD
Splooce       GKDATNVGDEGGFAPNILENKEGLELLKTAIGKAGYTDKVVIGMDVAASEFFRSGKYDLDFKSPDDPSRYISPDQLADLYKSFIKDYPVVSIEDPFDQDD

Uniprot       WGAWQKFTASAGIQVVGDDLTVTNPKRIAKAVNEKSCNCLLLKVNQIGSVTESLQACKLAQANGWGVMVSHRSGETEDTFIADLVVGLCTGQIKTGAPCR
Splooce       WGAWQKFTASAGIQVVGDDLTVTNPKRIAKAVNEKSCNCLLLKVNQIGSVTESLQACKLAQANGWGVMVSHRSGETEDTFIADLVVGLCTGQIKTGAPCR

Uniprot       SERLAKYNQLLRIEEELGSKAKFAGRNFRNPLAK
Splooce       SERLAKYNQLLRIEEELGSKAKFAGRNFRNPLAK

----------------------------------------------------------------------------------------------------

P07437 (Uniprot)	versus
NM_178014#(-t:6_T6539097215752) (Splooce)

For more details about the Alternative Splicing Event -> Link to Splooce page

Peptides that support the ASE (Splooce-specific):
MLNVQNK (MAXQUANT)

Alignment:
Uniprot       MREIVHIQAGQCGNQIGAKFWEVISDEHGIDPTGTYHGDSDLQLDRISVYYNEATGGKYVPRAILVDLEPGTMDSVRSGPFGQIFRPDNFVFGQSGAGNN
Splooce       ----------------------------------------------------------------------------------------------------

Uniprot       WAKGHYTEGAELVDSVLDVVRKEAESCDCLQGFQLTHSLGGGTGSGMGTLLISKIREEYPDRIMNTFSVVPSPKVSDTVVEPYNATLSVHQLVENTDETY
Splooce       ----------------------------------------------------------------------------------------------------

Uniprot       CIDNEALYDICFRTLKLTTPTYGDLNHLVSATMSGVTTCLRFPGQLNADLRKLAVNMVPFPRLHFFMPGFAPLTSRGSQQYRALTVPELTQQVFDAKNMM
Splooce       ----------------------------------------------------------------------------------------------------

Uniprot       AACDPRHGRYLTVAAVFRGRMSMKEVDEQMLNVQNKNSSYFVEWIPNNVKTAVCDIPPRGLKMAVTFIGNSTAIQELFKRISEQFTAMFRRKAFLHWYTG
Splooce       -----------------------------MLNVQNKNSSYFVEWIPNNVKTAVCDIPPRGLKMAVTFIGNSTAIQELFKRISEQFTAMFRRKAFLHWYTG

Uniprot       EGMDEMEFTEAESNMNDLVSEYQQYQDATAEEEEDFGEEAEEEA
Splooce       EGMDEMEFTEAESNMNDLVSEYQQYQDATAEEEEDFGEEAEEEA

----------------------------------------------------------------------------------------------------

E9PBU3 (Uniprot)	versus
NM_004044#(-s-:2_A3609679526834) (Splooce)

For more details about the Alternative Splicing Event -> Link to Splooce page

Peptides that support the ASE (Splooce-specific):
MVYDLYK (PEAKS)

Alignment:
Uniprot       MAPGQLALFSVSDKTGLVEFARNLTALGLNLVASGGTAKALRDAGLAVRDVSELTGFPEMLGGRVKTLHPAVHAGILARNIPEDNADMARLDFNLIRVVA
Splooce       ----------------------------------------------------------------------------------------------------

Uniprot       CNLYPFVKTVASPGVTVEEAVEQIDIGGVTLLRAAAKNHARVTVVCEPEDYVVVSTEMQSSESKDTSLETRRQLALKAFTHTAQYDEAISDYFRKQYSKG
Splooce       ----------------------------------------------------------------------------------------------------

Uniprot       VSQMPLRYGMNPHQTPAQLYTLQPKLPITVLNGAPGFINLCDALNAWQLVKELKEALGIPAAASFKHVSPAGAAVGIPLSEDEAKVCMVYDLYKTLTPIS
Splooce       ---------------------------------------------------------------------------------------MVYDLYKTLTPIS

Uniprot       AAYARARGADRMSSFGDFVALSDVCDVPTAKIISREVSDGIIAPGYEEEALTILSKKKNGNYCVLQMDQSYKPDENEVRTLFGLHLSQKRNNGVVDKSLF
Splooce       AAYARARGADRMSSFGDFVALSDVCDVPTAKIISREVSDGIIAPGYEEEALTILSKKKNGNYCVLQMDQSYKPDENEVRTLFGLHLSQKRNNGVVDKSLF

Uniprot       SNVVTKNKDLPESALRDLIVATIAVKYTQSNSVCYAKNGQVIGIGAGQQSRIHCTRLAGDKANYWWLRHHPQVLSMKFKTGVKRAEISNAIDQYVTGTIG
Splooce       SNVVTKNKDLPESALRDLIVATIAVKYTQSNSVCYAKNGQVIGIGAGQQSRIHCTRLAGDKANYWWLRHHPQVLSMKFKTGVKRAEISNAIDQYVTGTIG

Uniprot       EDEDLIKWKALFEEVPELLTEAEKKEWVEKLTEVSISSDAFFPFRDNVDRAKRSGVAYIAAPSGSAADKVVIEACDELGIILAHTNLRLFHH
Splooce       EDEDLIKWKALFEEVPELLTEAEKKEWVEKLTEVSISSDAFFPFRDNVDRAKRSGVAYIAAPSGSAADKVVIEACDELGIILAHTNLRLFHH

----------------------------------------------------------------------------------------------------

O00291 (Uniprot)	versus
NM_005338#(-s-:7_H2360767189357) (Splooce)

For more details about the Alternative Splicing Event -> Link to Splooce page

Peptides that support the ASE (Splooce-specific):
FDYLECELNLFQTVFNSLDMSR (MAXQUANT)

Alignment:
Uniprot       MDRMASSMKQVPNPLPKVLSRRGVGAGLEAAERESFERTQTVSINKAINTQEVAVKEKHARTCILGTHHEKGAQTFWSVVNRLPLSSNAVLCWKFCHVFH
Splooce       ----------------------------------------------------------------------------------------------------

Uniprot       KLLRDGHPNVLKDSLRYRNELSDMSRMWGHLSEGYGQLCSIYLKLLRTKMEYHTKNPRFPGNLQMSDRQLDEAGESDVNNFFQLTVEMFDYLECELNLFQ
Splooce       ---------------------------------------------------------------------------------------MFDYLECELNLFQ

Uniprot       TVFNSLDMSRSVSVTAAGQCRLAPLIQVILDCSHLYDYTVKLLFKLHSCLPADTLQGHRDRFMEQFTKLKDLFYRSSNLQYFKRLIQIPQLPENPPNFLR
Splooce       TVFNSLDMSRSVSVTAAGQCRLAPLIQVILDCSHLYDYTVKLLFKLHSCLPADTLQGHRDRFMEQFTKLKDLFYRSSNLQYFKRLIQIPQLPENPPNFLR

Uniprot       ASALSEHISPVVVIPAEASSPDSEPVLEKDDLMDMDASQQNLFDNKFDDIFGSSFSSDPFNFNSQNGVNKDEKDHLIERLYREISGLKAQLENMKTESQR
Splooce       ASALSEHISPVVVIPAEASSPDSEPVLEKDDLMDMDASQQNLFDNKFDDIFGSSFSSDPFNFNSQNGVNKDEKDHLIERLYREISGLKAQLENMKTESQR

Uniprot       VVLQLKGHVSELEADLAEQQHLRQQAADDCEFLRAELDELRRQREDTEKAQRSLSEIERKAQANEQRYSKLKEKYSELVQNHADLLRKNAEVTKQVSMAR
Splooce       VVLQLKGHVSELEADLAEQQHLRQQAADDCEFLRAELDELRRQREDTEKAQRSLSEIERKAQANEQRYSKLKEKYSELVQNHADLLRKNAEVTKQVSMAR

Uniprot       QAQVDLEREKKELEDSLERISDQGQRKTQEQLEVLESLKQELATSQRELQVLQGSLETSAQSEANWAAEFAELEKERDSLVSGAAHREEELSALRKELQD
Splooce       QAQVDLEREKKELEDSLERISDQGQRKTQEQLEVLESLKQELATSQRELQVLQGSLETSAQSEANWAAEFAELEKERDSLVSGAAHREEELSALRKELQD

Uniprot       TQLKLASTEESMCQLAKDQRKMLLVGSRKAAEQVIQDALNQLEEPPLISCAGSADHLLSTVTSISSCIEQLEKSWSQYLACPEDISGLLHSITLLAHLTS
Splooce       TQLKLASTEESMCQLAKDQRKMLLVGSRKAAEQVIQDALNQLEEPPLISCAGSADHLLSTVTSISSCIEQLEKSWSQYLACPEDISGLLHSITLLAHLTS

Uniprot       DAIAHGATTCLRAPPEPADSLTEACKQYGRETLAYLASLEEEGSLENADSTAMRNCLSKIKAIGEELLPRGLDIKQEELGDLVDKEMAATSAAIETATAR
Splooce       DAIAHGATTCLRAPPEPADSLTEACKQYGRETLAYLASLEEEGSLENADSTAMRNCLSKIKAIGEELLPRGLDIKQEELGDLVDKEMAATSAAIETATAR

Uniprot       IEEMLSKSRAGDTGVKLEVNERILGCCTSLMQAIQVLIVASKDLQREIVESGRGTASPKEFYAKNSRWTEGLISASKAVGWGATVMVDAADLVVQGRGKF
Splooce       IEEMLSKSRAGDTGVKLEVNERILGCCTSLMQAIQVLIVASKDLQREIVESGRGTASPKEFYAKNSRWTEGLISASKAVGWGATVMVDAADLVVQGRGKF

Uniprot       EELMVCSHEIAASTAQLVAASKVKADKDSPNLAQLQQASRGVNQATAGVVASTISGKSQIEETDNMDFSSMTLTQIKRQEMDSQVRVLELENELQKERQK
Splooce       EELMVCSHEIAASTAQLVAASKVKADKDSPNLAQLQQASRGVNQATAGVVASTISGKSQIEETDNMDFSSMTLTQIKRQEMDSQVRVLELENELQKERQK

Uniprot       LGELRKKHYELAGVAEGWEEGTEASPPTLQEVVTEKE
Splooce       LGELRKKHYELAGVAEGWEEGTEASPPTLQEVVTEKE

----------------------------------------------------------------------------------------------------

G5E9V1 (Uniprot)	versus
NM_001007565#(f-:3_T243937707082) (Splooce)

For more details about the Alternative Splicing Event -> Link to Splooce page

Peptides that support the ASE (Splooce-specific):
AASMSAFDPLKNQDEINK (MAXQUANT)
AASMSAFDPLK (MAXQUANT + PEAKS)

Alignment:
Uniprot       MNGQLDLSGKLIIKAQLGEDIRRIPIHNEDITYDELVLMMQRVFRGKLLSNDEVTIKYKDEDGDLITIFDSSDLSFAIQCSRILKLTLFVNGQPRPLESS
Splooce       ----------------------------------------------------------------------------------------------------

Uniprot       QVKYLRRELIELRNKVNRLLDSLEPPGEPGPSTNIPENDTVDGREEKSASDSSGKQSTQVMAASMSAFDPLKNQDEINKNVMSAFGLTDDQVSGPPSAPA
Splooce       ------------------------------------------------------------MAASMSAFDPLKNQDEINKNVMSAFGLTDDQVSGPPSAPA

Uniprot       EDRSGTPDSIASSSSAAHPPGVQPQQPPYTGAQTQAGQIEGQMYQQYQQQAGYGAQQPQAPPQQPQQYGIQYSASYSQQTGPQQPQQFQGYGQQPTSQAP
Splooce       EDRSGTPDSIASSSSAAHPPGVQPQQPPYTGAQTQAGQIEGQMYQQYQQQAGYGAQQPQAPPQQPQQYGIQYSASYSQQTGPQQPQQFQGYGQQPTSQAP

Uniprot       APAFSGQPQQLPAQPPQQYQASNYPAQTYTAQTSQPTNYTVAPASQPGMAPSQPGAYQPRPGFTSLPGSTMTPPPSGPNPYARNRPPFGQGYTQPGPGYR
Splooce       APAFSGQPQQLPAQPPQQYQASNYPAQTYTAQTSQPTNYTVAPASQPGMAPSQPGAYQPRPGFTSLPGSTMTPPPSGPNPYARNRPPFGQGYTQPGPGYR

----------------------------------------------------------------------------------------------------

P07237 (Uniprot)	versus
NM_000918#(-s-s-s-s-s-s-:17_P3395002943688) (Splooce)

For more details about the Alternative Splicing Event -> Link to Splooce page

Peptides that support the ASE (Splooce-specific):
MSQELPEDWDKQPVK (MAXQUANT)
SQELPEDWDKQPVK (MAXQUANT)
SQELPEDWDK (MAXQUANT)

Alignment:
Uniprot       MLRRALLCLAVAALVRADAPEEEDHVLVLRKSNFAEALAAHKYLLVEFYAPWCGHCKALAPEYAKAAGKLKAEGSEIRLAKVDATEESDLAQQYGVRGYP
Splooce       ----------------------------------------------------------------------------------------------------

Uniprot       TIKFFRNGDTASPKEYTAGREADDIVNWLKKRTGPAATTLPDGAAAESLVESSEVAVIGFFKDVESDSAKQFLQAAEAIDDIPFGITSNSDVFSKYQLDK
Splooce       ----------------------------------------------------------------------------------------------------

Uniprot       DGVVLFKKFDEGRNNFEGEVTKENLLDFIKHNQLPLVIEFTEQTAPKIFGGEIKTHILLFLPKSVSDYDGKLSNFKTAAESFKGKILFIFIDSDHTDNQR
Splooce       ----------------------------------------------------------------------------------------------------

Uniprot       ILEFFGLKKEECPAVRLITLEEEMTKYKPESEELTAERITEFCHRFLEGKIKPHLMSQELPEDWDKQPVKVLVGKNFEDVAFDEKKNVFVEFYAPWCGHC
Splooce       -------------------------------------------------------MSQELPEDWDKQPVKVLVGKNFEDVAFDEKKNVFVEFYAPWCGHC

Uniprot       KQLAPIWDKLGETYKDHENIVIAKMDSTANEVEAVKVHSFPTLKFFPASADRTVIDYNGERTLDGFKKFLESGGQDGAGDDDDLEDLEEAEEPDMEEDDD
Splooce       KQLAPIWDKLGETYKDHENIVIAKMDSTANEVEAVKVHSFPTLKFFPASADRTVIDYNGERTLDGFKKFLESGGQDGAGDDDDLEDLEEAEEPDMEEDDD

Uniprot       QKAVKDEL
Splooce       QKAVKDEL

----------------------------------------------------------------------------------------------------

P07900 (Uniprot)	versus
NM_005348#(f-S-T:14_H9566651045289) (Splooce)

For more details about the Alternative Splicing Event -> Link to Splooce page

Peptides that support the ASE (Splooce-specific):
DNCEELIPEYLNFIR (MAXQUANT)

Alignment:
Uniprot       MPEETQTQDQPMEEEEVETFAFQAEIAQLMSLIINTFYSNKEIFLRELISNSSDALDKIRYESLTDPSKLDSGKELHINLIPNKQDRTLTIVDTGIGMTK
Splooce       ----------------------------------------------------------------------------------------------------

Uniprot       ADLINNLGTIAKSGTKAFMEALQAGADISMIGQFGVGFYSAYLVAEKVTVITKHNDDEQYAWESSAGGSFTVRTDTGEPMGRGTKVILHLKEDQTEYLEE
Splooce       ----------------------------------------------------------------------------------------------------

Uniprot       RRIKEIVKKHSQFIGYPITLFVEKERDKEVSDDEAEEKEDKEEEKEKEEKESEDKPEIEDVGSDEEEEKKDGDKKKKKKIKEKYIDQEELNKTKPIWTRN
Splooce       ----------------------------------------------------------------------------------------------------

Uniprot       PDDITNEEYGEFYKSLTNDWEDHLAVKHFSVEGQLEFRALLFVPRRAPFDLFENRKKKNNIKLYVRRVFIMDNCEELIPEYLNFIRGVVDSEDLPLNISR
Splooce       ----------------------------------------------------------------------MDNCEELIPEYLNFIRGVVDSEDLPLNISR

Uniprot       EMLQQSKILKVIRKNLVKKCLELFTELAEDKENYKKFYEQFSKNIKLGIHEDSQNRKKLSELLRYYTSASGDEMVSLKDYCTRMKENQKHIYYITGETKD
Splooce       EMLQQSKILKVIRKNLVKKCLELFTELAEDKENYKKFYEQFSKNIKLGIHEDSQNRKKLSELLRYYTSASGDEMVSLKDYCTRMKENQKHIYYITGETKD

Uniprot       QVANSAFVERLRKHGLEVIYMIEPIDEYCVQQLKEFEGKTLVSVTKEGLELPEDEEEKKKQEEKKTKFENLCKIMKDILEKKVEKVVVSNRLVTSPCCIV
Splooce       QVANSAFVERLRKHGLEVIYMIEPIDEYCVQQLKEFEGKTLVSVTKEGLELPEDEEEKKKQEEKKTKFENLCKIMKDILEKKVEKVVVSNRLVTSPCCIV

Uniprot       TSTYGWTANMERIMKAQALRDNSTMGYMAAKKHLEINPDHSIIETLRQKAEADKNDKSVKDLVILLYETALLSSGFSLEDPQTHANRIYRMIKLGLGIDE
Splooce       TSTYGWTANMERIMKAQALRDNSTMGYMAAKKHLEINPDHSIIETLRQKAEADKNDKSVKDLVILLYETALLSSGFSLEDPQTHANRIYRMIKLGLGIDE

Uniprot       DDPTADDTSAAVTEEMPPLEGDDDTSRMEEVD
Splooce       DDPTADDTSAAVTEEMPPLEGDDDTSRMEEVD

----------------------------------------------------------------------------------------------------

P20290 (Uniprot)	versus
NM_001037637#(-t:5_B831272602288) (Splooce)

For more details about the Alternative Splicing Event -> Link to Splooce page

Peptides that support the ASE (Splooce-specific):
FTNQGTVIHFNNPK (MAXQUANT)

Alignment:
Uniprot       MRRTGAPAQADSRGRGRARGGCPGGEATLSQPPPRGGTRGQEPQMKETIMNQEKLAKLQAQVRIGGKGTARRKKKVVHRTATADDKKLQFSLKKLGVNNI
Splooce       ----------------------------------------------------------------------------------------------------

Uniprot       SGIEEVNMFTNQGTVIHFNNPKVQASLAANTFTITGHAETKQLTEMLPSILNQLGADSLTSLRRLAEALPKQSVDGKAPLATGEDDDDEVPDLVENFDEA
Splooce       -------MFTNQGTVIHFNNPKVQASLAANTFTITGHAETKQLTEMLPSILNQLGADSLTSLRRLAEALPKQSVDGKAPLATGEDDDDEVPDLVENFDEA

Uniprot       SKNEAN
Splooce       SKNEAN

----------------------------------------------------------------------------------------------------

P06733 (Uniprot)	versus
NM_001428#(-s-s-s-s-s-s-:1_E2067814744864) (Splooce)

For more details about the Alternative Splicing Event -> Link to Splooce page

Peptides that support the ASE (Splooce-specific):
DVAASEFFR (MAXQUANT)

Alignment:
Uniprot       MSILKIHAREIFDSRGNPTVEVDLFTSKGLFRAAVPSGASTGIYEALELRDNDKTRYMGKGVSKAVEHINKTIAPALVSKKLNVTEQEKIDKLMIEMDGT
Splooce       ----------------------------------------------------------------------------------------------------

Uniprot       ENKSKFGANAILGVSLAVCKAGAVEKGVPLYRHIADLAGNSEVILPVPAFNVINGGSHAGNKLAMQEFMILPVGAANFREAMRIGAEVYHNLKNVIKEKY
Splooce       ----------------------------------------------------------------------------------------------------

Uniprot       GKDATNVGDEGGFAPNILENKEGLELLKTAIGKAGYTDKVVIGMDVAASEFFRSGKYDLDFKSPDDPSRYISPDQLADLYKSFIKDYPVVSIEDPFDQDD
Splooce       -------------------------------------------MDVAASEFFRSGKYDLDFKSPDDPSRYISPDQLADLYKSFIKDYPVVSIEDPFDQDD

Uniprot       WGAWQKFTASAGIQVVGDDLTVTNPKRIAKAVNEKSCNCLLLKVNQIGSVTESLQACKLAQANGWGVMVSHRSGETEDTFIADLVVGLCTGQIKTGAPCR
Splooce       WGAWQKFTASAGIQVVGDDLTVTNPKRIAKAVNEKSCNCLLLKVNQIGSVTESLQACKLAQANGWGVMVSHRSGETEDTFIADLVVGLCTGQIKTGAPCR

Uniprot       SERLAKYNQLLRIEEELGSKAKFAGRNFRNPLAK
Splooce       SERLAKYNQLLRIEEELGSKAKFAGRNFRNPLAK

----------------------------------------------------------------------------------------------------

P24001 (Uniprot)	versus
NM_001012634#(-t:16_I6845707903384) (Splooce)

For more details about the Alternative Splicing Event -> Link to Splooce page

Peptides that support the ASE (Splooce-specific):
SSLAELEDDFK (PEAKS)

Alignment:
Uniprot       MCFPKVLSDDMKKLKARMVMSSLAELEDDFKEGYLETVAAYYEEQHPELTPLLEKERDGLRCRGNRSPVPDVEDPATEEPGESFCDKVMRWFQAMLQRLQ
Splooce       -------------------MSSLAELEDDFKEGYLETVAAYYEEQHPELTPLLEKERDGLRCRGNRSPVPDVEDPATEEPGESFCDKVMRWFQAMLQRLQ

Uniprot       TWWHGVLAWVKEKVVALVHAVQALWKQFQSFCCSLSELFMSSFQSYGAPRGDKEELTPQKCSEPQSSK
Splooce       TWWHGVLAWVKEKVVALVHAVQALWKQFQSFCCSLSELFMSSFQSYGAPRGDKEELTPQKCSEPQSSK

----------------------------------------------------------------------------------------------------

O00567 (Uniprot)	versus
NM_006392#(-t:20_N4201078078050) (Splooce)

For more details about the Alternative Splicing Event -> Link to Splooce page

Peptides that support the ASE (Splooce-specific):
IIQSISLLDQLDK (MAXQUANT)

Alignment:
Uniprot       MVLLHVLFEHAVGYALLALKEVEEISLLQPQVEESVLNLGKFHSIVRLVAFCPFASSQVALENANAVSEGVVHEDLRLLLETHLPSKKKKVLLGVGDPKI
Splooce       ----------------------------------------------------------------------------------------------------

Uniprot       GAAIQEELGYNCQTGGVIAEILRGVRLHFHNLVKGLTDLSACKAQLGLGHSYSRAKVKFNVNRVDNMIIQSISLLDQLDKDINTFSMRVREWYGYHFPEL
Splooce       ------------------------------------------------------------------MIIQSISLLDQLDKDINTFSMRVREWYGYHFPEL

Uniprot       VKIINDNATYCRLAQFIGNRRELNEDKLEKLEELTMDGAKAKAILDASRSSMGMDISAIDLINIESFSSRVVSLSEYRQSLHTYLRSKMSQVAPSLSALI
Splooce       VKIINDNATYCRLAQFIGNRRELNEDKLEKLEELTMDGAKAKAILDASRSSMGMDISAIDLINIESFSSRVVSLSEYRQSLHTYLRSKMSQVAPSLSALI

Uniprot       GEAVGARLIAHAGSLTNLAKYPASTVQILGAEKALFRALKTRGNTPKYGLIFHSTFIGRAAAKNKGRISRYLANKCSIASRIDCFSEVPTSVFGEKLREQ
Splooce       GEAVGARLIAHAGSLTNLAKYPASTVQILGAEKALFRALKTRGNTPKYGLIFHSTFIGRAAAKNKGRISRYLANKCSIASRIDCFSEVPTSVFGEKLREQ

Uniprot       VEERLSFYETGEIPRKNLDVMKEAMVQAEEAAAEITRKLEKQEKKRLKKEKKRLAALALASSENSSSTPEECEEMSEKPKKKKKQKPQEVPQENGMEDPS
Splooce       VEERLSFYETGEIPRKNLDVMKEAMVQAEEAAAEITRKLEKQEKKRLKKEKKRLAALALASSENSSSTPEECEEMSEKPKKKKKQKPQEVPQENGMEDPS

Uniprot       ISFSKPKKKKSFSKEELMSSDLEETAGSTSIPKRKKSTPKEETVNDPEEAGHRSGSKKKRKFSKEEPVSSGPEEAVGKSSSKKKKKFHKASQED
Splooce       ISFSKPKKKKSFSKEELMSSDLEETAGSTSIPKRKKSTPKEETVNDPEEAGHRSGSKKKRKFSKEEPVSSGPEEAVGKSSSKKKKKFHKASQED

----------------------------------------------------------------------------------------------------

Q8N543 (Uniprot)	versus
NM_018233#(-s-s-:16_O1967993408355) (Splooce)

For more details about the Alternative Splicing Event -> Link to Splooce page

Peptides that support the ASE (Splooce-specific):
GGTLDLYSIDEHFQPK (MAXQUANT)

Alignment:
Uniprot       MNGKRPAEPGPARVGKKGKKEVMAEFSDAVTEETLKKQVAEAWSRRTPFSHEVIVMDMDPFLHCVIPNFIQSQDFLEGLQKELMNLDFHEKYNDLYKFQQ
Splooce       ----------------------------------------------------------------------------------------------------

Uniprot       SDDLKKRREPHISTLRKILFEDFRSWLSDISKIDLESTIDMSCAKYEFTDALLCHDDELEGRRIAFILYLVPPWDRSMGGTLDLYSIDEHFQPKQIVKSL
Splooce       -----------------------------------------------------------------------------MGGTLDLYSIDEHFQPKQIVKSL

Uniprot       IPSWNKLVFFEVSPVSFHQVSEVLSEEKSRLSISGWFHGPSLTRPPNYFEPPIPRSPHIPQDHEILYDWINPTYLDMDYQVQIQEEFEESSEILLKEFLK
Splooce       IPSWNKLVFFEVSPVSFHQVSEVLSEEKSRLSISGWFHGPSLTRPPNYFEPPIPRSPHIPQDHEILYDWINPTYLDMDYQVQIQEEFEESSEILLKEFLK

Uniprot       PEKFTKVCEALEHGHVEWSSRGPPNKRFYEKAEESKLPEILKECMKLFRSEALFLLLSNFTGLKLHFLAPSEEDEMNDKKEAETTDITEEGTSHSPPEPE
Splooce       PEKFTKVCEALEHGHVEWSSRGPPNKRFYEKAEESKLPEILKECMKLFRSEALFLLLSNFTGLKLHFLAPSEEDEMNDKKEAETTDITEEGTSHSPPEPE

Uniprot       NNQMAISNNSQQSNEQTDPEPEENETKKESSVPMCQGELRHWKTGHYTLIHDHSKAEFALDLILYCGCEGWEPEYGGFTSYIAKGEDEELLTVNPESNSL
Splooce       NNQMAISNNSQQSNEQTDPEPEENETKKESSVPMCQGELRHWKTGHYTLIHDHSKAEFALDLILYCGCEGWEPEYGGFTSYIAKGEDEELLTVNPESNSL

Uniprot       ALVYRDRETLKFVKHINHRSLEQKKTFPNRTGFWDFSFIYYE
Splooce       ALVYRDRETLKFVKHINHRSLEQKKTFPNRTGFWDFSFIYYE

----------------------------------------------------------------------------------------------------

P17844 (Uniprot)	versus
NM_004396#(r:17_D9511011415116) (Splooce)

For more details about the Alternative Splicing Event -> Link to Splooce page

Peptides that support the ASE (Splooce-specific):
WSATWPK (MAXQUANT)

Alignment:
Uniprot       MSGYSSDRDRGRDRGFGAPRFGGSRAGPLSGKKFGNPGEKLVKKKWNLDELPKFEKNFYQEHPDLARRTAQEVETYRRSKEITVRGHNCPKPVLNFYEAN
Splooce       ----------------------------------------------------------------------------------------------------

Uniprot       FPANVMDVIARQNFTEPTAIQAQGWPVALSGLDMVGVAQTGSGKTLSYLLPAIVHINHQPFLERGDGPICLVLAPTRELAQQVQQVAAEYCRACRLKSTC
Splooce       ----------------------------------------------------------------------------------------------------

Uniprot       IYGGAPKGPQIRDLERGVEICIATPGRLIDFLECGKTNLRRTTYLVLDEADRMLDMGFEPQIRKIVDQIRPDRQTLMWSATWPKEVRQLAEDFLKDYIHI
Splooce       ----------------------------------------------------------------------------MWSATWPKEVRQLAEDFLKDYIHI

Uniprot       NIGALELSANHNILQIVDVCHDVEKDEKLIRLMEEIMSEKENKTIVFVETKRRCDELTRKMRRDGWPAMGIHGDKSQQERDWVLNEFKHGKAPILIATDV
Splooce       NIGALELSANHNILQIVDVCHDVEKDEKLIRLMEEIMSEKENKTIVFVETKRRCDELTRKMRRDGWPAMGIHGDKSQQERDWVLNEFKHGKAPILIATDV

Uniprot       ASRGLDVEDVKFVINYDYPNSSEDYIHRIGRTARSTKTGTAYTFFTPNNIKQVSDLISVLREANQAINPKLLQLVEDRGSGRSRGRGGMKDDRRDRYSAG
Splooce       ASRGLDVEDVKFVINYDYPNSSEDYIHRIGRTARSTKTGTAYTFFTPNNIKQVSDLISVLREANQAINPKLLQLVEDRGSGRSRGRGGMKDDRRDRYSAG

Uniprot       KRGGFNTFRDRENYDRGYSSLLKRDFGAKTQNGVYSAANYTNGSFGSNFVSAGIQTSFRTGNPTGTYQNGYDSTQQYGSNVPNMHNGMNQQAYAYPATAA
Splooce       KRGGFNTFRDRENYDRGYSSLLKRDFGAKTQNGVYSAANYTNGSFGSNFVSAGIQTSFRTGNPTGTYQNGYDSTQQYGSNVPNMHNGMNQQAYAYPATAA

Uniprot       APMIGYPMPTGYSQ
Splooce       APMIGYPMPTGYSQ

----------------------------------------------------------------------------------------------------

Q9NR45 (Uniprot)	versus
NM_018946#(-s-:9_N2598954260829) (Splooce)

For more details about the Alternative Splicing Event -> Link to Splooce page

Peptides that support the ASE (Splooce-specific):
VLSSGMQSMDTMK (PEAKS)
VISSGMQSMDTMK (MAXQUANT)

Alignment:
Uniprot       MPLELELCPGRWVGGQHPCFIIAEIGQNHQGDLDVAKRMIRMAKECGADCAKFQKSELEFKFNRKALERPYTSKHSWGKTYGEHKRHLEFSHDQYRELQR
Splooce       ----------------------------------------------------------------------------------------------------

Uniprot       YAEEVGIFFTASGMDEMAVEFLHELNVPFFKVGSGDTNNFPYLEKTAKKGRPMVISSGMQSMDTMKQVYQIVKPLNPNFCFLQCTSAYPLQPEDVNLRVI
Splooce       ----------------------------------------------------MVISSGMQSMDTMKQVYQIVKPLNPNFCFLQCTSAYPLQPEDVNLRVI

Uniprot       SEYQKLFPDIPIGYSGHETGIAISVAAVALGAKVLERHITLDKTWKGSDHSASLEPGELAELVRSVRLVERALGSPTKQLLPCEMACNEKLGKSVVAKVK
Splooce       SEYQKLFPDIPIGYSGHETGIAISVAAVALGAKVLERHITLDKTWKGSDHSASLEPGELAELVRSVRLVERALGSPTKQLLPCEMACNEKLGKSVVAKVK

Uniprot       IPEGTILTMDMLTVKVGEPKGYPPEDIFNLVGKKVLVTVEEDDTIMEELVDNHGKKIKS
Splooce       IPEGTILTMDMLTVKVGEPKGYPPEDIFNLVGKKVLVTVEEDDTIMEELVDNHGKKIKS

----------------------------------------------------------------------------------------------------

O14925 (Uniprot)	versus
NM_006327#(-s-:10_T2653669856017) (Splooce)

For more details about the Alternative Splicing Event -> Link to Splooce page

Peptides that support the ASE (Splooce-specific):
MNPLSPYLNVDPR (MAXQUANT)

Alignment:
Uniprot       MEGGGGSGNKTTGGLAGFFGAGGAGYSHADLAGVPLTGMNPLSPYLNVDPRYLVQDTDEFILPTGANKTRGRFELAFFTIGGCCMTGAAFGAMNGLRLGL
Splooce       --------------------------------------MNPLSPYLNVDPRYLVQDTDEFILPTGANKTRGRFELAFFTIGGCCMTGAAFGAMNGLRLGL

Uniprot       KETQNMAWSKPRNVQILNMVTRQGALWANTLGSLALLYSAFGVIIEKTRGAEDDLNTVAAGTMTGMLYKCTGGLRGIARGGLTGLTLTSLYALYNNWEHM
Splooce       KETQNMAWSKPRNVQILNMVTRQGALWANTLGSLALLYSAFGVIIEKTRGAEDDLNTVAAGTMTGMLYKCTGGLRGIARGGLTGLTLTSLYALYNNWEHM

Uniprot       KGSLLQQSL
Splooce       KGSLLQQSL

----------------------------------------------------------------------------------------------------

Q13630 (Uniprot)	versus
NM_003313#(-s-s-:8_T8935231908060) (Splooce)

For more details about the Alternative Splicing Event -> Link to Splooce page

Peptides that support the ASE (Splooce-specific):
ELDFLER (PEAKS)

Alignment:
Uniprot       MGEPQGSMRILVTGGSGLVGKAIQKVVADGAGLPGEDWVFVSSKDADLTDTAQTRALFEKVQPTHVIHLAAMVGGLFRNIKYNLDFWRKNVHMNDNVLHS
Splooce       ---------------------------------------------MELDFLERTGCLSPLKTPISRIQHRPAP--CLR---------RSNPHTSSILLQW

Uniprot       AFEVGARKVVSCLSTCIFPDKTTYPIDETMIHNGPPHNSNFGYSYAKRMIDVQNRAYFQQYGCTFTAVIPTNVFGPHDNFNIEDGHVLPGLIHKVHLAKS
Splooce       WGACSG-------------------ISNTIWTSG---------------------AYFQQYGCTFTAVIPTNVFGPHDNFNIEDGHVLPGLIHKVHLAKS

Uniprot       SGSALTVWGTGNPRRQFIYSLDLAQLFIWVLREYNEVEPIILSVGEEDEVSIKEAAEAVVEAMDFHGEVTFDTTKSDGQFKKTASNSKLRTYLPDFRFTP
Splooce       SGSALTVWGTGNPRRQFIYSLDLAQLFIWVLREYNEVEPIILSVGEEDEVSIKEAAEAVVEAMDFHGEVTFDTTKSDGQFKKTASNSKLRTYLPDFRFTP

Uniprot       FKQAVKETCAWFTDNYEQARK
Splooce       FKQAVKETCAWFTDNYEQARK

----------------------------------------------------------------------------------------------------

Q15046 (Uniprot)	versus
NM_005548#(r:16_K3464874012308) (Splooce)

For more details about the Alternative Splicing Event -> Link to Splooce page

Peptides that support the ASE (Splooce-specific):
MLPHLHFGLK (MAXQUANT + PEAKS)

Alignment:
Uniprot       MAAVQAAEVKVDGSEPKLSKNELKRRLKAEKKVAEKEAKQKELSEKQLSQATAAATNHTTDNGVGPEEESVDPNQYYKIRSQAIHQLKVNGEDPYPHKFH
Splooce       ----------------------------------------------------------------------------------------------------

Uniprot       VDISLTDFIQKYSHLQPGDHLTDITLKVAGRIHAKRASGGKLIFYDLRGEGVKLQVMANSRNYKSEEEFIHINNKLRRGDIIGVQGNPGKTKKGELSIIP
Splooce       ----------------------------------------------------------------------------------------------------

Uniprot       YEITLLSPCLHMLPHLHFGLKDKETRYRQRYLDLILNDFVRQKFIIRSKIITYIRSFLDELGFLEIETPMMNIIPGGAVAKPFITYHNELDMNLYMRIAP
Splooce       -----------MLPHLHFGLKDKETRYRQRYLDLILNDFVRQKFIIRSKIITYIRSFLDELGFLEIETPMMNIIPGGAVAKPFITYHNELDMNLYMRIAP

Uniprot       ELYHKMLVVGGIDRVYEIGRQFRNEGIDLTHNPEFTTCEFYMAYADYHDLMEITEKMVSGMVKHITGSYKVTYHPDGPEGQAYDVDFTPPFRRINMVEEL
Splooce       ELYHKMLVVGGIDRVYEIGRQFRNEGIDLTHNPEFTTCEFYMAYADYHDLMEITEKMVSGMVKHITGSYKVTYHPDGPEGQAYDVDFTPPFRRINMVEEL

Uniprot       EKALGMKLPETNLFETEETRKILDDICVAKAVECPPPRTTARLLDKLVGEFLEVTCINPTFICDHPQIMSPLAKWHRSKEGLTERFELFVMKKEICNAYT
Splooce       EKALGMKLPETNLFETEETRKILDDICVAKAVECPPPRTTARLLDKLVGEFLEVTCINPTFICDHPQIMSPLAKWHRSKEGLTERFELFVMKKEICNAYT

Uniprot       ELNDPMRQRQLFEEQAKAKAAGDDEAMFIDENFCTALEYGLPPTAGWGMGIDRVAMFLTDSNNIKEVLLFPAMKPEDKKENVATTDTLESTTVGTSV
Splooce       ELNDPMRQRQLFEEQAKAKAAGDDEAMFIDENFCTALEYGLPPTAGWGMGIDRVAMFLTDSNNIKEVLLFPAMKPEDKKENVATTDTLESTTVGTSV

----------------------------------------------------------------------------------------------------

P14550 (Uniprot)	versus
NM_153326#(-t:1_A4109191434672) (Splooce)

For more details about the Alternative Splicing Event -> Link to Splooce page

Peptides that support the ASE (Splooce-specific):
MHWPYAFER (MAXQUANT)

Alignment:
Uniprot       MAASCVLLHTGQKMPLIGLGTWKSEPGQVKAAVKYALSVGYRHIDCAAIYGNEPEIGEALKEDVGPGKAVPREELFVTSKLWNTKHHPEDVEPALRKTLA
Splooce       ----------------------------------------------------------------------------------------------------

Uniprot       DLQLEYLDLYLMHWPYAFERGDNPFPKNADGTICYDSTHYKETWKALEALVAKGLVQALGLSNFNSRQIDDILSVASVRPAVLQVECHPYLAQNELIAHC
Splooce       -----------MHWPYAFERGDNPFPKNADGTICYDSTHYKETWKALEALVAKGLVQALGLSNFNSRQIDDILSVASVRPAVLQVECHPYLAQNELIAHC

Uniprot       QARGLEVTAYSPLGSSDRAWRDPDEPVLLEEPVVLALAEKYGRSPAQILLRWQVQRKVICIPKSITPSRILQNIKVFDFTFSPEEMKQLNALNKNWRYIV
Splooce       QARGLEVTAYSPLGSSDRAWRDPDEPVLLEEPVVLALAEKYGRSPAQILLRWQVQRKVICIPKSITPSRILQNIKVFDFTFSPEEMKQLNALNKNWRYIV

Uniprot       PMLTVDGKRVPRDAGHPLYPFNDPY
Splooce       PMLTVDGKRVPRDAGHPLYPFNDPY

----------------------------------------------------------------------------------------------------

O00401 (Uniprot)	versus
NM_003941#(f-:7_W337103792926) (Splooce)

For more details about the Alternative Splicing Event -> Link to Splooce page

Peptides that support the ASE (Splooce-specific):
SSAVVQLYAADR (MAXQUANT)

Alignment:
Uniprot       MSSVQQQPPPPRRVTNVGSLLLTPQENESLFTFLGKKCVTMSSAVVQLYAADRNCMWSKKCSGVACLVKDNPQRSYFLRIFDIKDGKLLWEQELYNNFVY
Splooce       ----------------------------------------MSSAVVQLYAADRNCMWSKKCSGVACLVKDNPQRSYFLRIFDIKDGKLLWEQELYNNFVY

Uniprot       NSPRGYFHTFAGDTCQVALNFANEEEAKKFRKAVTDLLGRRQRKSEKRRDPPNGPNLPMATVDIKNPEITTNRFYGPQVNNISHTKEKKKGKAKKKRLTK
Splooce       NSPRGYFHTFAGDTCQVALNFANEEEAKKFRKAVTDLLGRRQRKSEKRRDPPNGPNLPMATVDIKNPEITTNRFYGPQVNNISHTKEKKKGKAKKKRLTK

Uniprot       ADIGTPSNFQHIGHVGWDPNTGFDLNNLDPELKNLFDMCGISEAQLKDRETSKVIYDFIEKTGGVEAVKNELRRQAPPPPPPSRGGPPPPPPPPHNSGPP
Splooce       ADIGTPSNFQHIGHVGWDPNTGFDLNNLDPELKNLFDMCGISEAQLKDRETSKVIYDFIEKTGGVEAVKNELRRQAPPPPPPSRGGPPPPPPPPHNSGPP

Uniprot       PPPARGRGAPPPPPSRAPTAAPPPPPPSRPSVAVPPPPPNRMYPPPPPALPSSAPSGPPPPPPSVLGVGPVAPPPPPPPPPPPGPPPPPGLPSDGDHQVP
Splooce       PPPARGRGAPPPPPSRAPTAAPPPPPPSRPSVAVPPPPPNRMYPPPPPALPSSAPSGPPPPPPSVLGVGPVAPPPPPPPPPPPGPPPPPGLPSDGDHQVP

Uniprot       TTAGNKAALLDQIREGAQLKKVEQNSRPVSCSGRDALLDQIRQGIQLKSVADGQESTPPTPAPTSGIVGALMEVMQKRSKAIHSSDEDEDEDDEEDFEDD
Splooce       TTAGNKAALLDQIREGAQLKKVEQNSRPVSCSGRDALLDQIRQGIQLKSVADGQESTPPTPAPTSGIVGALMEVMQKRSKAIHSSDEDEDEDDEEDFEDD

Uniprot       DEWED
Splooce       DEWED

----------------------------------------------------------------------------------------------------

O95373 (Uniprot)	versus
NM_006391#(-s-s-s-s-s-s-s-s-:11_I2300728055572) (Splooce)

For more details about the Alternative Splicing Event -> Link to Splooce page

Peptides that support the ASE (Splooce-specific):
MTFNQVIQTGPDEEGSDDK (MAXQUANT)
TFNQVIQTGPDEEGSDDK (MAXQUANT)

Alignment:
Uniprot       MDPNTIIEALRGTMDPALREAAERQLNEAHKSLNFVSTLLQITMSEQLDLPVRQAGVIYLKNMITQYWPDRETAPGDISPYTIPEEDRHCIRENIVEAII
Splooce       ----------------------------------------------------------------------------------------------------

Uniprot       HSPELIRVQLTTCIHHIIKHDYPSRWTAIVDKIGFYLQSDNSACWLGILLCLYQLVKNYEYKKPEERSPLVAAMQHFLPVLKDRFIQLLSDQSDQSVLIQ
Splooce       ----------------------------------------------------------------------------------------------------

Uniprot       KQIFKIFYALVQYTLPLELINQQNLTEWIEILKTVVNRDVPNETLQVEEDDRPELPWWKCKKWALHILARLFERYGSPGNVSKEYNEFAEVFLKAFAVGV
Splooce       ----------------------------------------------------------------------------------------------------

Uniprot       QQVLLKVLYQYKEKQYMAPRVLQQTLNYINQGVSHALTWKNLKPHIQGIIQDVIFPLMCYTDADEELWQEDPYEYIRMKFDVFEDFISPTTAAQTLLFTA
Splooce       ----------------------------------------------------------------------------------------------------

Uniprot       CSKRKEVLQKTMGFCYQILTEPNADPRKKDGALHMIGSLAEILLKKKIYKDQMEYMLQNHVFPLFSSELGYMRARACWVLHYFCEVKFKSDQNLQTALEL
Splooce       ----------------------------------------------------------------------------------------------------

Uniprot       TRRCLIDDREMPVKVEAAIALQVLISNQEKAKEYITPFIRPVMQALLHIIRETENDDLTNVIQKMICEYSEEVTPIAVEMTQHLAMTFNQVIQTGPDEEG
Splooce       -------------------------------------------------------------------------------------MTFNQVIQTGPDEEG

Uniprot       SDDKAVTAMGILNTIDTLLSVVEDHKEITQQLEGICLQVIGTVLQQHVLEFYEEIFSLAHSLTCQQVSPQMWQLLPLVFEVFQQDGFDYFTDMMPLLHNY
Splooce       SDDKAVTAMGILNTIDTLLSVVEDHKEITQQLEGICLQVIGTVLQQHVLEFYEEIFSLAHSLTCQQVSPQMWQLLPLVFEVFQQDGFDYFTDMMPLLHNY

Uniprot       VTVDTDTLLSDTKYLEMIYSMCKKVLTGVAGEDAECHAAKLLEVIILQCKGRGIDQCIPLFVEAALERLTREVKTSELRTMCLQVAIAALYYNPHLLLNT
Splooce       VTVDTDTLLSDTKYLEMIYSMCKKVLTGVAGEDAECHAAKLLEVIILQCKGRGIDQCIPLFVEAALERLTREVKTSELRTMCLQVAIAALYYNPHLLLNT

Uniprot       LENLRFPNNVEPVTNHFITQWLNDVDCFLGLHDRKMCVLGLCALIDMEQIPQVLNQVSGQILPAFILLFNGLKRAYACHAEHENDSDDDDEAEDDDETEE
Splooce       LENLRFPNNVEPVTNHFITQWLNDVDCFLGLHDRKMCVLGLCALIDMEQIPQVLNQVSGQILPAFILLFNGLKRAYACHAEHENDSDDDDEAEDDDETEE

Uniprot       LGSDEDDIDEDGQEYLEILAKQAGEDGDDEDWEEDDAEETALEGYSTIIDDEDNPVDEYQIFKAIFQTIQNRNPVWYQALTHGLNEEQRKQLQDIATLAD
Splooce       LGSDEDDIDEDGQEYLEILAKQAGEDGDDEDWEEDDAEETALEGYSTIIDDEDNPVDEYQIFKAIFQTIQNRNPVWYQALTHGLNEEQRKQLQDIATLAD

Uniprot       QRRAAHESKMIEKHGGYKFSAPVVPSSFNFGGPAPGMN
Splooce       QRRAAHESKMIEKHGGYKFSAPVVPSSFNFGGPAPGMN

----------------------------------------------------------------------------------------------------

O94979 (Uniprot)	versus
NM_001077207#(-s-s-s-:4_S1864175720620) (Splooce)

For more details about the Alternative Splicing Event -> Link to Splooce page

Peptides that support the ASE (Splooce-specific):
MADPELLLSCGK (MAXQUANT)

Alignment:
Uniprot       MKLKEVDRTAMQAWSPAQNHPIYLATGTSAQQLDATFSTNASLEIFELDLSDPSLDMKSCATFSSSHRYHKLIWGPYKMDSKGDVSGVLIAGGENGNIIL
Splooce       ----------------------------------------------------------------------------------------------------

Uniprot       YDPSKIIAGDKEVVIAQNDKHTGPVRALDVNIFQTNLVASGANESEIYIWDLNNFATPMTPGAKTQPPEDISCIAWNRQVQHILASASPSGRATVWDLRK
Splooce       ----------------------------------------------------------------------------------------------------

Uniprot       NEPIIKVSDHSNRMHCSGLAWHPDVATQMVLASEDDRLPVIQMWDLRFASSPLRVLENHARGILAIAWSMADPELLLSCGKDAKILCSNPNTGEVLYELP
Splooce       ---------------------------------------------------------------------MADPELLLSCGKDAKILCSNPNTGEVLYELP

Uniprot       TNTQWCFDIQWCPRNPAVLSAASFDGRISVYSIMGGSTDGLRQKQVDKLSSSFGNLDPFGTGQPLPPLQIPQQTAQHSIVLPLKKPPKWIRRPVGASFSF
Splooce       TNTQWCFDIQWCPRNPAVLSAASFDGRISVYSIMGGSTDGLRQKQVDKLSSSFGNLDPFGTGQPLPPLQIPQQTAQHSIVLPLKKPPKWIRRPVGASFSF

Uniprot       GGKLVTFENVRMPSHQGAEQQQQQHHVFISQVVTEKEFLSRSDQLQQAVQSQGFINYCQKKIDASQTEFEKNVWSFLKVNFEDDSRGKYLELLGYRKEDL
Splooce       GGKLVTFENVRMPSHQGAEQQQQQHHVFISQVVTEKEFLSRSDQLQQAVQSQGFINYCQKKIDASQTEFEKNVWSFLKVNFEDDSRGKYLELLGYRKEDL

Uniprot       GKKIALALNKVDGANVALKDSDQVAQSDGEESPAAEEQLLGEHIKEEKEESEFLPSSGGTFNISVSGDIDGLITQALLTGNFESAVDLCLHDNRMADAII
Splooce       GKKIALALNKVDGANVALKDSDQVAQSDGEESPAAEEQLLGEHIKEEKEESEFLPSSGGTFNISVSGDIDGLITQALLTGNFESAVDLCLHDNRMADAII

Uniprot       LAIAGGQELLARTQKKYFAKSQSKITRLITAVVMKNWKEIVESCDLKNWREALAAVLTYAKPDEFSALCDLLGTRLENEGDSLLQTQACLCYICAGNVEK
Splooce       LAIAGGQELLARTQKKYFAKSQSKITRLITAVVMKNWKEIVESCDLKNWREALAAVLTYAKPDEFSALCDLLGTRLENEGDSLLQTQACLCYICAGNVEK

Uniprot       LVACWTKAQDGSHPLSLQDLIEKVVILRKAVQLTQAMDTSTVGVLLAAKMSQYANLLAAQGSIAAALAFLPDNTNQPNIMQLRDRLCRAQGEPVAGHESP
Splooce       LVACWTKAQDGSHPLSLQDLIEKVVILRKAVQLTQAMDTSTVGVLLAAKMSQYANLLAAQGSIAAALAFLPDNTNQPNIMQLRDRLCRAQGEPVAGHESP

Uniprot       KIPYEKQQLPKGRPGPVAGHHQMPRVQTQQYYPHGENPPPPGFIMHGNVNPNAAGQLPTSPGHMHTQVPPYPQPQPYQPAQPYPFGTGGSAMYRPQQPVA
Splooce       KIPYEKQQLPKGRPGPVAGHHQMPRVQTQQYYPHGENPPPPGFIMHGNVNPNAAGQLPTSPGHMHTQVPPYPQPQPYQPAQPYPFGTGGSAMYRPQQPVA

Uniprot       PPTSNAYPNTPYISSASSYTGQSQLYAAQHQASSPTSSPATSFPPPPSSGASFQHGGPGAPPSSSAYALPPGTTGTLPAASELPASQRTGPQNGWNDPPA
Splooce       PPTSNAYPNTPYISSASSYTGQSQLYAAQHQASSPTSSPATSFPPPPSSGASFQHGGPGAPPSSSAYALPPGTTGTLPAASELPASQRTGPQNGWNDPPA

Uniprot       LNRVPKKKKMPENFMPPVPITSPIMNPLGDPQSQMLQQQPSAPVPLSSQSSFPQPHLPGGQPFHGVQQPLGQTGMPPSFSKPNIEGAPGAPIGNTFQHVQ
Splooce       LNRVPKKKKMPENFMPPVPITSPIMNPLGDPQSQMLQQQPSAPVPLSSQSSFPQPHLPGGQPFHGVQQPLGQTGMPPSFSKPNIEGAPGAPIGNTFQHVQ

Uniprot       SLPTKKITKKPIPDEHLILKTTFEDLIQRCLSSATDPQTKRKLDDASKRLEFLYDKLREQTLSPTITSGLHNIARSIETRNYSEGLTMHTHIVSTSNFSE
Splooce       SLPTKKITKKPIPDEHLILKTTFEDLIQRCLSSATDPQTKRKLDDASKRLEFLYDKLREQTLSPTITSGLHNIARSIETRNYSEGLTMHTHIVSTSNFSE

Uniprot       TSAFMPVLKVVLTQANKLGV
Splooce       TSAFMPVLKVVLTQANKLGV

----------------------------------------------------------------------------------------------------

P63241 (Uniprot)	versus
NM_001970#(-s-:17_E2683783024997) (Splooce)

For more details about the Alternative Splicing Event -> Link to Splooce page

Peptides that support the ASE (Splooce-specific):
MDVPNIK (MAXQUANT)
MDVPNLK (PEAKS)

Alignment:
Uniprot       MADDLDFETGDAGASATFPMQCSALRKNGFVVLKGRPCKIVEMSTSKTGKHGHAKVHLVGIDIFTGKKYEDICPSTHNMDVPNIKRNDFQLIGIQDGYLS
Splooce       ------------------------------------------------------------------------------MDVPNIKRNDFQLIGIQDGYLS

Uniprot       LLQDSGEVREDLRLPEGDLGKEIEQKYDCGEEILITVLSAMTEEAAVAIKAMAK
Splooce       LLQDSGEVREDLRLPEGDLGKEIEQKYDCGEEILITVLSAMTEEAAVAIKAMAK

----------------------------------------------------------------------------------------------------

Q9UHX1 (Uniprot)	versus
NM_078480#(-s-:8_P9095383569583) (Splooce)

For more details about the Alternative Splicing Event -> Link to Splooce page

Peptides that support the ASE (Splooce-specific):
NLFDLGGQYLR (MAXQUANT)

Alignment:
Uniprot       MATATIALQVNGQQGGGSEPAAAAAVVAAGDKWKPPQGTDSIKMENGQSTAAKLGLPPLTPEQQEALQKAKKYAMEQSIKSVLVKQTIAHQQQQLTNLQM
Splooce       ----------------------------------------------------------------------------------------------------

Uniprot       AAVTMGFGDPLSPLQSMAAQRQRALAIMCRVYVGSIYYELGEDTIRQAFAPFGPIKSIDMSWDSVTMKHKGFAFVEYEVPEAAQLALEQMNSVMLGGRNI
Splooce       ----------------------------------------------------------------------------------------------------

Uniprot       KVGRPSNIGQAQPIIDQLAEEARAFNRIYVASVHQDLSDDDIKSVFEAFGKIKSCTLARDPTTGKHKGYGFIEYEKAQSSQDAVSSMNLFDLGGQYLRVG
Splooce       --------------------------------------------------------------------------------------MNLFDLGGQYLRVG

Uniprot       KAVTPPMPLLTPATPGGLPPAAAVAAAAATAKITAQEAVAGAAVLGTLGTPGLVSPALTLAQPLGTLPQAVMAAQAPGVITGVTPARPPIPVTIPSVGVV
Splooce       KAVTPPMPLLTPATPGGLPPAAAVAAAAATAKITAQEAVAGAAVLGTLGTPGLVSPALTLAQPLGTLPQAVMAAQAPGVITGVTPARPPIPVTIPSVGVV

Uniprot       NPILASPPTLGLLEPKKEKEEEELFPESERPEMLSEQEHMSISGSSARHMVMQKLLRKQESTVMVLRNMVDPKDIDDDLEGEVTEECGKFGAVNRVIIYQ
Splooce       NPILASPPTLGLLEPKKEKEEEELFPESERPEMLSEQEHMSISGSSARHMVMQKLLRKQESTVMVLRNMVDPKDIDDDLEGEVTEECGKFGAVNRVIIYQ

Uniprot       EKQGEEEDAEIIVKIFVEFSIASETHKAIQALNGRWFAGRKVVAEVYDQERFDNSDLSA
Splooce       EKQGEEEDAEIIVKIFVEFSIASETHKAIQALNGRWFAGRKVVAEVYDQERFDNSDLSA

----------------------------------------------------------------------------------------------------

A8MVQ3 (Uniprot)	versus
NM_001014438#(-s-s-s-s-:11_C3872030735396) (Splooce)

For more details about the Alternative Splicing Event -> Link to Splooce page

Peptides that support the ASE (Splooce-specific):
AGTLLGASMDIHGGGFDLR (MAXQUANT)

Alignment:
Uniprot       MQTPPLQQPHQEQVFLAFLVIVIPSFLTKEVFIPQDGKKVTWYCCGPTVYDASHMGHARSYISFDILRRVLKDYFKFDVFYCMNITDIDDKIIKRARQNH
Splooce       ----------------------------------------------------------------------------------------------------

Uniprot       LFEQYREKRPEAAQLLEDVQAALKPFSVKLNETTDPDKKQMLERIQHAVQLATEPLEKAVQSRLTGEEVNSCVEVLLEEAKDLLSDWLDSTLGCDVTDNS
Splooce       ----------------------------------------------------------------------------------------------------

Uniprot       IFSKLPKFWEGDFHRDMEALNVLPPDVLTRVSEYVPEIVNFVQKIVDNGYGYVSNGSVYFDTAKFASSEKHSYGKLVPEAVGDQKALQEGEGDLSISADR
Splooce       ----------------------------------------------------------------------------------------------------

Uniprot       LSEKRSPNDFALWKASKPGEPSWPCPWGKGRPGWHIECSAMAGTLLGASMDIHGGGFDLRFPHHDNELAQSEAYFENDCWVRYFLHTGHLTIAGCKMSKS
Splooce       ----------------------------------------MAGTLLGASMDIHGGGFDLRFPHHDNELAQSEAYFENDCWVRYFLHTGHLTIAGCKMSKS

Uniprot       LKNFITIKDALKKHSARQLRLAFLMHSWKDTLDYSSNTMESALQYEKFLNEFFLNVKDILRAPVDITGQFEKWGEEEAELNKNFYDKKTAIHKALCDNVD
Splooce       LKNFITIKDALKKHSARQLRLAFLMHSWKDTLDYSSNTMESALQYEKFLNEFFLNVKDILRAPVDITGQFEKWGEEEAELNKNFYDKKTAIHKALCDNVD

Uniprot       TRTVMEEMRALVSQCNLYMAARKAVRKRPNQALLENIALYLTHMLKIFGAVEEDSSLGFPVGGPGTSLSLEATVMPYLQVLSEFREGVRKIAREQKVPEI
Splooce       TRTVMEEMRALVSQCNLYMAARKAVRKRPNQALLENIALYLTHMLKIFGAVEEDSSLGFPVGGPGTSLSLEATVMPYLQVLSEFREGVRKIAREQKVPEI

Uniprot       LQLSDALRDNILPELGVRFEDHEGLPTVVKLVDRNTLLKEREEKRRVEEEKRKKKEEAARRKQEQEAAKLAKMKIPPSEMFLSETDKYSKFDENGLPTHD
Splooce       LQLSDALRDNILPELGVRFEDHEGLPTVVKLVDRNTLLKEREEKRRVEEEKRKKKEEAARRKQEQEAAKLAKMKIPPSEMFLSETDKYSKFDENGLPTHD

Uniprot       MEGKELSKGQAKKLKKLFEAQEKLYKEYLQMAQNGSFQ
Splooce       MEGKELSKGQAKKLKKLFEAQEKLYKEYLQMAQNGSFQ

----------------------------------------------------------------------------------------------------

O75306 (Uniprot)	versus
NM_004550#(f-:1_N6515137451741) (Splooce)

For more details about the Alternative Splicing Event -> Link to Splooce page

Peptides that support the ASE (Splooce-specific):
MCNEQAYSLAVEK (MAXQUANT + PEAKS)

Alignment:
Uniprot       MAALRALCGFRGVAAQVLRPGAGVRLPIQPSRGVRQWQPDVEWAQQFGGAVMYPSKETAHWKPPPWNDVDPPKDTIVKNITLNFGPQHPAAHGVLRLVME
Splooce       ----------------------------------------------------------------------------------------------------

Uniprot       LSGEMVRKCDPHIGLLHRGTEKLIEYKTYLQALPYFDRLDYVSMMCNEQAYSLAVEKLLNIRPPPRAQWIRVLFGEITRLLNHIMAVTTHALDLGAMTPF
Splooce       -------------------------------------------MMCNEQAYSLAVEKLLNIRPPPRAQWIRVLFGEITRLLNHIMAVTTHALDLGAMTPF

Uniprot       FWLFEEREKMFEFYERVSGARMHAAYIRPGGVHQDLPLGLMDDIYQFSKNFSLRLDELEELLTNNRIWRNRTIDIGVVTAEEALNYGFSGVMLRGSGIQW
Splooce       FWLFEEREKMFEFYERVSGARMHAAYIRPGGVHQDLPLGLMDDIYQFSKNFSLRLDELEELLTNNRIWRNRTIDIGVVTAEEALNYGFSGVMLRGSGIQW

Uniprot       DLRKTQPYDVYDQVEFDVPVGSRGDCYDRYLCRVEEMRQSLRIIAQCLNKMPPGEIKVDDAKVSPPKRAEMKTSMESLIHHFKLYTEGYQVPPGATYTAI
Splooce       DLRKTQPYDVYDQVEFDVPVGSRGDCYDRYLCRVEEMRQSLRIIAQCLNKMPPGEIKVDDAKVSPPKRAEMKTSMESLIHHFKLYTEGYQVPPGATYTAI

Uniprot       EAPKGEFGVYLVSDGSSRPYRCKIKAPGFAHLAGLDKMSKGHMLADVVAIIGTQDIVFGEVDR
Splooce       EAPKGEFGVYLVSDGSSRPYRCKIKAPGFAHLAGLDKMSKGHMLADVVAIIGTQDIVFGEVDR

----------------------------------------------------------------------------------------------------

Q14974 (Uniprot)	versus
NM_002265#(-s-s-s-s-s-s-s-:17_K229893614285) (Splooce)

For more details about the Alternative Splicing Event -> Link to Splooce page

Peptides that support the ASE (Splooce-specific):
LLATCCEDDIVPHVLPFIK (MAXQUANT)

Alignment:
Uniprot       MELITILEKTVSPDRLELEAAQKFLERAAVENLPTFLVELSRVLANPGNSQVARVAAGLQIKNSLTSKDPDIKAQYQQRWLAIDANARREVKNYVLQTLG
Splooce       ----------------------------------------------------------------------------------------------------

Uniprot       TETYRPSSASQCVAGIACAEIPVNQWPELIPQLVANVTNPNSTEHMKESTLEAIGYICQDIDPEQLQDKSNEILTAIIQGMRKEEPSNNVKLAATNALLN
Splooce       ----------------------------------------------------------------------------------------------------

Uniprot       SLEFTKANFDKESERHFIMQVVCEATQCPDTRVRVAALQNLVKIMSLYYQYMETYMGPALFAITIEAMKSDIDEVALQGIEFWSNVCDEEMDLAIEASEA
Splooce       ----------------------------------------------------------------------------------------------------

Uniprot       AEQGRPPEHTSKFYAKGALQYLVPILTQTLTKQDENDDDDDWNPCKAAGVCLMLLATCCEDDIVPHVLPFIKEHIKNPDWRYRDAAVMAFGCILEGPEPS
Splooce       ----------------------------------------------------MLLATCCEDDIVPHVLPFIKEHIKNPDWRYRDAAVMAFGCILEGPEPS

Uniprot       QLKPLVIQAMPTLIELMKDPSVVVRDTAAWTVGRICELLPEAAINDVYLAPLLQCLIEGLSAEPRVASNVCWAFSSLAEAAYEAADVADDQEEPATYCLS
Splooce       QLKPLVIQAMPTLIELMKDPSVVVRDTAAWTVGRICELLPEAAINDVYLAPLLQCLIEGLSAEPRVASNVCWAFSSLAEAAYEAADVADDQEEPATYCLS

Uniprot       SSFELIVQKLLETTDRPDGHQNNLRSSAYESLMEIVKNSAKDCYPAVQKTTLVIMERLQQVLQMESHIQSTSDRIQFNDLQSLLCATLQNVLRKVQHQDA
Splooce       SSFELIVQKLLETTDRPDGHQNNLRSSAYESLMEIVKNSAKDCYPAVQKTTLVIMERLQQVLQMESHIQSTSDRIQFNDLQSLLCATLQNVLRKVQHQDA

Uniprot       LQISDVVMASLLRMFQSTAGSGGVQEDALMAVSTLVEVLGGEFLKYMEAFKPFLGIGLKNYAEYQVCLAAVGLVGDLCRALQSNIIPFCDEVMQLLLENL
Splooce       LQISDVVMASLLRMFQSTAGSGGVQEDALMAVSTLVEVLGGEFLKYMEAFKPFLGIGLKNYAEYQVCLAAVGLVGDLCRALQSNIIPFCDEVMQLLLENL

Uniprot       GNENVHRSVKPQILSVFGDIALAIGGEFKKYLEVVLNTLQQASQAQVDKSDYDMVDYLNELRESCLEAYTGIVQGLKGDQENVHPDVMLVQPRVEFILSF
Splooce       GNENVHRSVKPQILSVFGDIALAIGGEFKKYLEVVLNTLQQASQAQVDKSDYDMVDYLNELRESCLEAYTGIVQGLKGDQENVHPDVMLVQPRVEFILSF

Uniprot       IDHIAGDEDHTDGVVACAAGLIGDLCTAFGKDVLKLVEARPMIHELLTEGRRSKTNKAKTLATWATKELRKLKNQA
Splooce       IDHIAGDEDHTDGVVACAAGLIGDLCTAFGKDVLKLVEARPMIHELLTEGRRSKTNKAKTLATWATKELRKLKNQA

----------------------------------------------------------------------------------------------------

P15924 (Uniprot)	versus
NM_001008844#(-s-s-s-s-s-s-:6_D2184109508668) (Splooce)

For more details about the Alternative Splicing Event -> Link to Splooce page

Peptides that support the ASE (Splooce-specific):
QLYECQLLDK (PEAKS)
QLYECQLIDK (MAXQUANT)

Alignment:
Uniprot       MSCNGGSHPRINTLGRMIRAESGPDLRYEVTSGGGGTSRMYYSRRGVITDQNSDGYCQTGTMSRHQNQNTIQELLQNCSDCLMRAELIVQPELKYGDGIQ
Splooce       ----------------------------------------------------------------------------------------------------

Uniprot       LTRSRELDECFAQANDQMEILDSLIREMRQMGQPCDAYQKRLLQLQEQMRALYKAISVPRVRRASSKGGGGYTCQSGSGWDEFTKHVTSECLGWMRQQRA
Splooce       ----------------------------------------------------------------------------------------------------

Uniprot       EMDMVAWGVDLASVEQHINSHRGIHNSIGDYRWQLDKIKADLREKSAIYQLEEEYENLLKASFERMDHLRQLQNIIQATSREIMWINDCEEEELLYDWSD
Splooce       ----------------------------------------------------------------------------------------------------

Uniprot       KNTNIAQKQEAFSIRMSQLEVKEKELNKLKQESDQLVLNQHPASDKIEAYMDTLQTQWSWILQITKCIDVHLKENAAYFQFFEEAQSTEAYLKGLQDSIR
Splooce       ----------------------------------------------------------------------------------------------------

Uniprot       KKYPCDKNMPLQHLLEQIKELEKEREKILEYKRQVQNLVNKSKKIVQLKPRNPDYRSNKPIILRALCDYKQDQKIVHKGDECILKDNNERSKWYVTGPGG
Splooce       ----------------------------------------------------------------------------------------------------

Uniprot       VDMLVPSVGLIIPPPNPLAVDLSCKIEQYYEAILALWNQLYINMKSLVSWHYCMIDIEKIRAMTIAKLKTMRQEDYMKTIADLELHYQEFIRNSQGSEMF
Splooce       ----------------------------------------------------------------------------------------------------

Uniprot       GDDDKRKIQSQFTDAQKHYQTLVIQLPGYPQHQTVTTTEITHHGTCQDVNHNKVIETNRENDKQETWMLMELQKIRRQIEHCEGRMTLKNLPLADQGSSH
Splooce       ----------------------------------------------------------------------------------------------------

Uniprot       HITVKINELKSVQNDSQAIAEVLNQLKDMLANFRGSEKYCYLQNEVFGLFQKLENINGVTDGYLNSLCTVRALLQAILQTEDMLKVYEARLTEEETVCLD
Splooce       ----------------------------------------------------------------------------------------------------

Uniprot       LDKVEAYRCGLKKIKNDLNLKKSLLATMKTELQKAQQIHSQTSQQYPLYDLDLGKFGEKVTQLTDRWQRIDKQIDFRLWDLEKQIKQLRNYRDNYQAFCK
Splooce       ----------------------------------------------------------------------------------------------------

Uniprot       WLYDAKRRQDSLESMKFGDSNTVMRFLNEQKNLHSEISGKRDKSEEVQKIAELCANSIKDYELQLASYTSGLETLLNIPIKRTMIQSPSGVILQEAADVH
Splooce       ----------------------------------------------------------------------------------------------------

Uniprot       ARYIELLTRSGDYYRFLSEMLKSLEDLKLKNTKIEVLEEELRLARDANSENCNKNKFLDQNLQKYQAECSQFKAKLASLEELKRQAELDGKSAKQNLDKC
Splooce       ----------------------------------------------------------------------------------------------------

Uniprot       YGQIKELNEKITRLTYEIEDEKRRRKSVEDRFDQQKNDYDQLQKARQCEKENLGWQKLESEKAIKEKEYEIERLRVLLQEEGTRKREYENELAKASNRIQ
Splooce       ----------------------------------------------------------------------------------------------------

Uniprot       ESKNQCTQVVQERESLLVKIKVLEQDKARLQRLEDELNRAKSTLEAETRVKQRLECEKQQIQNDLNQWKTQYSRKEEAIRKIESEREKSEREKNSLRSEI
Splooce       ----------------------------------------------------------------------------------------------------

Uniprot       ERLQAEIKRIEERCRRKLEDSTRETQSQLETERSRYQREIDKLRQRPYGSHRETQTECEWTVDTSKLVFDGLRKKVTAMQLYECQLIDKTTLDKLLKGKK
Splooce       ------------------------------------------------------------------------------MQLYECQLIDKTTLDKLLKGKK

Uniprot       SVEEVASEIQPFLRGAGSIAGASASPKEKYSLVEAKRKKLISPESTVMLLEAQAATGGIIDPHRNEKLTVDSAIARDLIDFDDRQQIYAAEKAITGFDDP
Splooce       SVEEVASEIQPFLRGAGSIAGASASPKEKYSLVEAKRKKLISPESTVMLLEAQAATGGIIDPHRNEKLTVDSAIARDLIDFDDRQQIYAAEKAITGFDDP

Uniprot       FSGKTVSVSEAIKKNLIDRETGMRLLEAQIASGGVVDPVNSVFLPKDVALARGLIDRDLYRSLNDPRDSQKNFVDPVTKKKVSYVQLKERCRIEPHTGLL
Splooce       FSGKTVSVSEAIKKNLIDRETGMRLLEAQIASGGVVDPVNSVFLPKDVALARGLIDRDLYRSLNDPRDSQKNFVDPVTKKKVSYVQLKERCRIEPHTGLL

Uniprot       LLSVQKRSMSFQGIRQPVTVTELVDSGILRPSTVNELESGQISYDEVGERIKDFLQGSSCIAGIYNETTKQKLGIYEAMKIGLVRPGTALELLEAQAATG
Splooce       LLSVQKRSMSFQGIRQPVTVTELVDSGILRPSTVNELESGQISYDEVGERIKDFLQGSSCIAGIYNETTKQKLGIYEAMKIGLVRPGTALELLEAQAATG

Uniprot       FIVDPVSNLRLPVEEAYKRGLVGIEFKEKLLSAERAVTGYNDPETGNIISLFQAMNKELIEKGHGIRLLEAQIATGGIIDPKESHRLPVDIAYKRGYFNE
Splooce       FIVDPVSNLRLPVEEAYKRGLVGIEFKEKLLSAERAVTGYNDPETGNIISLFQAMNKELIEKGHGIRLLEAQIATGGIIDPKESHRLPVDIAYKRGYFNE

Uniprot       ELSEILSDPSDDTKGFFDPNTEENLTYLQLKERCIKDEETGLCLLPLKEKKKQVQTSQKNTLRKRRVVIVDPETNKEMSVQEAYKKGLIDYETFKELCEQ
Splooce       ELSEILSDPSDDTKGFFDPNTEENLTYLQLKERCIKDEETGLCLLPLKEKKKQVQTSQKNTLRKRRVVIVDPETNKEMSVQEAYKKGLIDYETFKELCEQ

Uniprot       ECEWEEITITGSDGSTRVVLVDRKTGSQYDIQDAIDKGLVDRKFFDQYRSGSLSLTQFADMISLKNGVGTSSSMGSGVSDDVFSSSRHESVSKISTISSV
Splooce       ECEWEEITITGSDGSTRVVLVDRKTGSQYDIQDAIDKGLVDRKFFDQYRSGSLSLTQFADMISLKNGVGTSSSMGSGVSDDVFSSSRHESVSKISTISSV

Uniprot       RNLTIRSSSFSDTLEESSPIAAIFDTENLEKISITEGIERGIVDSITGQRLLEAQACTGGIIHPTTGQKLSLQDAVSQGVIDQDMATRLKPAQKAFIGFE
Splooce       RNLTIRSSSFSDTLEESSPIAAIFDTENLEKISITEGIERGIVDSITGQRLLEAQACTGGIIHPTTGQKLSLQDAVSQGVIDQDMATRLKPAQKAFIGFE

Uniprot       GVKGKKKMSAAEAVKEKWLPYEAGQRFLEFQYLTGGLVDPEVHGRISTEEAIRKGFIDGRAAQRLQDTSSYAKILTCPKTKLKISYKDAINRSMVEDITG
Splooce       GVKGKKKMSAAEAVKEKWLPYEAGQRFLEFQYLTGGLVDPEVHGRISTEEAIRKGFIDGRAAQRLQDTSSYAKILTCPKTKLKISYKDAINRSMVEDITG

Uniprot       LRLLEAASVSSKGLPSPYNMSSAPGSRSGSRSGSRSGSRSGSRSGSRRGSFDATGNSSYSYSYSFSSSSIGH
Splooce       LRLLEAASVSSKGLPSPYNMSSAPGSRSGSRSGSRSGSRSGSRSGSRRGSFDATGNSSYSYSYSFSSSSIGH

----------------------------------------------------------------------------------------------------

P30838 (Uniprot)	versus
NM_000691#(-s-s-:17_A7611471040033) (Splooce)

For more details about the Alternative Splicing Event -> Link to Splooce page

Peptides that support the ASE (Splooce-specific):
MASLLATIIPQYLDK (MAXQUANT)

Alignment:
Uniprot       MSKISEAVKRARAAFSSGRTRPLQFRIQQLEALQRLIQEQEQELVGALAADLHKNEWNAYYEEVVYVLEEIEYMIQKLPEWAADEPVEKTPQTQQDELYI
Splooce       ----------------------------------------------------------------------------------------------------

Uniprot       HSEPLGVVLVIGTWNYPFNLTIQPMVGAIAAGNSVVLKPSELSENMASLLATIIPQYLDKDLYPVINGGVPETTELLKERFDHILYTGSTGVGKIIMTAA
Splooce       ---------------------------------------------MASLLATIIPQYLDKDLYPVINGGVPETTELLKERFDHILYTGSTGVGKIIMTAA

Uniprot       AKHLTPVTLELGGKSPCYVDKNCDLDVACRRIAWGKFMNSGQTCVAPDYILCDPSIQNQIVEKLKKSLKEFYGEDAKKSRDYGRIISARHFQRVMGLIEG
Splooce       AKHLTPVTLELGGKSPCYVDKNCDLDVACRRIAWGKFMNSGQTCVAPDYILCDPSIQNQIVEKLKKSLKEFYGEDAKKSRDYGRIISARHFQRVMGLIEG

Uniprot       QKVAYGGTGDAATRYIAPTILTDVDPQSPVMQEEIFGPVLPIVCVRSLEEAIQFINQREKPLALYMFSSNDKVIKKMIAETSSGGVAANDVIVHITLHSL
Splooce       QKVAYGGTGDAATRYIAPTILTDVDPQSPVMQEEIFGPVLPIVCVRSLEEAIQFINQREKPLALYMFSSNDKVIKKMIAETSSGGVAANDVIVHITLHSL

Uniprot       PFGGVGNSGMGSYHGKKSFETFSHRRSCLVRPLMNDEGLKVRYPPSPAKMTQH
Splooce       PFGGVGNSGMGSYHGKKSFETFSHRRSCLVRPLMNDEGLKVRYPPSPAKMTQH

----------------------------------------------------------------------------------------------------

F5GXH4 (Uniprot)	versus
NM_004192#(-s-:X_A2086052799127) (Splooce)

For more details about the Alternative Splicing Event -> Link to Splooce page

Peptides that support the ASE (Splooce-specific):
LVESVHGDFLNVVGFPLNHFCK (MAXQUANT)

Alignment:
Uniprot       MVLCPVIGKLLHKRVVLASASPRRQEILSNAGLRFEVVPSKFKEKLDKASFATPYGYAMETAKQKALEVANRLYQKDLRAPDVVIGADTIVTVGGLILEK
Splooce       ----------------------------------------------------------------------------------------------------

Uniprot       PVDKQDAYRMLSRLSGREHSVFTGVAIVHCSSKDHQLDTRVSEFYEETKVKFSELSEELLWEYVHSGEPMDKAGGYGIQALGGMLVESVHGDFLNVVGFP
Splooce       -----------------------------------------------------------------------------------MLVESVHGDFLNVVGFP

Uniprot       LNHFCKQLVKLYYPPRPEDLRRSVKHDSIPAADTFEDLSDVEGGGSEPTQRDAGSRDEKAEAGEAGQATAEAECHRTRETLPPFPTRLLELIEGFMLSKG
Splooce       LNHFCKQLVKLYYPPRPEDLRRSVKHDSIPAADTFEDLSDVEGGGSEPTQRDAGSRDEKAEAGEAGQATAEAECHRTRETLPPFPTRLLELIEGFMLSKG

Uniprot       LLTACKLKVFDLLKDEAPQKAADIASKVDASACGMERLLDICAAMGLLEKTEQGYSNTETANVYLASDGEYSLHGFIMHNNDLTWNLFTYLEFAIREGTN
Splooce       LLTACKLKVFDLLKDEAPQKAADIASKVDASACGMERLLDICAAMGLLEKTEQGYSNTETANVYLASDGEYSLHGFIMHNNDLTWNLFTYLEFAIREGTN

Uniprot       QHHRALGKKAEDLFQDAYYQSPETRLRFMRAMHGMTKLTACQVATAFNLSRFSSACDVGGCTGALARELAREYPRMQVTVFDLPDIIELAAHFQPPGPQA
Splooce       QHHRALGKKAEDLFQDAYYQSPETRLRFMRAMHGMTKLTACQVATAFNLSRFSSACDVGGCTGALARELAREYPRMQVTVFDLPDIIELAAHFQPPGPQA

Uniprot       VQIHFAAGDFFRDPLPSAELYVLCRILHDWPDDKVHKLLSRVAESCKPGAGLLLVETLLDEEKRVAQRALMQSLNMLVQTEGKERSLGEYQCLLELHGFH
Splooce       VQIHFAAGDFFRDPLPSAELYVLCRILHDWPDDKVHKLLSRVAESCKPGAGLLLVETLLDEEKRVAQRALMQSLNMLVQTEGKERSLGEYQCLLELHGFH

Uniprot       QVQVVHLGGVLDAILATKVAP
Splooce       QVQVVHLGGVLDAILATKVAP

----------------------------------------------------------------------------------------------------

J3KP72 (Uniprot)	versus
NM_005389#(-s-:6_P3226985344693) (Splooce)

For more details about the Alternative Splicing Event -> Link to Splooce page

Peptides that support the ASE (Splooce-specific):
MHAYALELLFDQLHEGAK (MAXQUANT)

Alignment:
Uniprot       MAWKSGGASHSELIHNLRKNGIIKTDKVFEVMLATDRSHYAKCNPYMDSPQSIGFQATISAPHMHAYALELLFDQLHEGAKALDVGSGSGILTACFARMV
Splooce       ---------------------------------------------------------------MHAYALELLFDQLHEGAKALDVGSGSGILTACFARMV

Uniprot       GCTGKVIGIDHIKELVDDSVNNVRKDDPTLLSSGRVQLVVGDGRMGYAEEAPYDAIHVGAAAPVVPQALIDQLKPGGRLILPVGPAGGNQMLEQYDKLQD
Splooce       GCTGKVIGIDHIKELVDDSVNNVRKDDPTLLSSGRVQLVVGDGRMGYAEEAPYDAIHVGAAAPVVPQALIDQLKPGGRLILPVGPAGGNQMLEQYDKLQD

Uniprot       GSIKMKPLMGVIYVPLTDKEKQWSRWK
Splooce       GSIKMKPLMGVIYVPLTDKEKQWSRWK

----------------------------------------------------------------------------------------------------

Q96RP9 (Uniprot)	versus
NM_024996#(-s-:3_G7467729120840) (Splooce)

For more details about the Alternative Splicing Event -> Link to Splooce page

Peptides that support the ASE (Splooce-specific):
MQIPMGLEGNFK (MAXQUANT)

Alignment:
Uniprot       MRLLGAAAVAALGRGRAPASLGWQRKQVNWKACRWSSSGVIPNEKIRNIGISAHIDSGKTTLTERVLYYTGRIAKMHEVKGKDGVGAVMDSMELERQRGI
Splooce       ----------------------------------------------------------------------------------------------------

Uniprot       TIQSAATYTMWKDVNINIIDTPGHVDFTIEVERALRVLDGAVLVLCAVGGVQCQTMTVNRQMKRYNVPFLTFINKLDRMGSNPARALQQMRSKLNHNAAF
Splooce       ----------------------------------------------------------------------------------------------------

Uniprot       MQIPMGLEGNFKGIVDLIEERAIYFDGDFGQIVRYGEIPAELRAAATDHRQELIECVANSDEQLGEMFLEEKIPSISDLKLAIRRATLKRSFTPVFLGSA
Splooce       MQIPMGLEGNFKGIVDLIEERAIYFDGDFGQIVRYGEIPAELRAAATDHRQELIECVANSDEQLGEMFLEEKIPSISDLKLAIRRATLKRSFTPVFLGSA

Uniprot       LKNKGVQPLLDAVLEYLPNPSEVQNYAILNKEDDSKEKTKILMNSSRDNSHPFVGLAFKLEVGRFGQLTYVRSYQGELKKGDTIYNTRTRKKVRLQRLAR
Splooce       LKNKGVQPLLDAVLEYLPNPSEVQNYAILNKEDDSKEKTKILMNSSRDNSHPFVGLAFKLEVGRFGQLTYVRSYQGELKKGDTIYNTRTRKKVRLQRLAR

Uniprot       MHADMMEDVEEVYAGDICALFGIDCASGDTFTDKANSGLSMESIHVPDPVISIAMKPSNKNDLEKFSKGIGRFTREDPTFKVYFDTENKETVISGMGELH
Splooce       MHADMMEDVEEVYAGDICALFGIDCASGDTFTDKANSGLSMESIHVPDPVISIAMKPSNKNDLEKFSKGIGRFTREDPTFKVYFDTENKETVISGMGELH

Uniprot       LEIYAQRLEREYGCPCITGKPKVAFRETITAPVPFDFTHKKQSGGAGQYGKVIGVLEPLDPEDYTKLEFSDETFGSNIPKQFVPAVEKGFLDACEKGPLS
Splooce       LEIYAQRLEREYGCPCITGKPKVAFRETITAPVPFDFTHKKQSGGAGQYGKVIGVLEPLDPEDYTKLEFSDETFGSNIPKQFVPAVEKGFLDACEKGPLS

Uniprot       GHKLSGLRFVLQDGAHHMVDSNEISFIRAGEGALKQALANATLCILEPIMAVEVVAPNEFQGQVIAGINRRHGVITGQDGVEDYFTLYADVPLNDMFGYS
Splooce       GHKLSGLRFVLQDGAHHMVDSNEISFIRAGEGALKQALANATLCILEPIMAVEVVAPNEFQGQVIAGINRRHGVITGQDGVEDYFTLYADVPLNDMFGYS

Uniprot       TELRSCTEGKGEYTMEYSRYQPCLPSTQEDVINKYLEATGQLPVKKGKAKN
Splooce       TELRSCTEGKGEYTMEYSRYQPCLPSTQEDVINKYLEATGQLPVKKGKAKN

----------------------------------------------------------------------------------------------------

O95433 (Uniprot)	versus
NM_012111#(-s-:14_A712841114510) (Splooce)

For more details about the Alternative Splicing Event -> Link to Splooce page

Peptides that support the ASE (Splooce-specific):
ILPTMNGESVDPVGQPALK (MAXQUANT)

Alignment:
Uniprot       MAKWGEGDPRWIVEERADATNVNNWHWTERDASNWSTDKLKTLFLAVQVQNEEGKCEVTEVSKLDGEASINNRKGKLIFFYEWSVKLNWTGTSKSGVQYK
Splooce       ----------------------------------------------------------------------------------------------------

Uniprot       GHVEIPNLSDENSVDEVEISVSLAKDEPDTNLVALMKEEGVKLLREAMGIYISTLKTEFTQGMILPTMNGESVDPVGQPALKTEERKAKPAPSKTQARPV
Splooce       --------------------------------------------------------------MILPTMNGESVDPVGQPALKTEERKAKPAPSKTQARPV

Uniprot       GVKIPTCKITLKETFLTSPEELYRVFTTQELVQAFTHAPATLEADRGGKFHMVDGNVSGEFTDLVPEKHIVMKWRFKSWPEGHFATITLTFIDKNGETEL
Splooce       GVKIPTCKITLKETFLTSPEELYRVFTTQELVQAFTHAPATLEADRGGKFHMVDGNVSGEFTDLVPEKHIVMKWRFKSWPEGHFATITLTFIDKNGETEL

Uniprot       CMEGRGIPAPEEERTRQGWQRYYFEGIKQTFGYGARLF
Splooce       CMEGRGIPAPEEERTRQGWQRYYFEGIKQTFGYGARLF

----------------------------------------------------------------------------------------------------

P09429 (Uniprot)	versus
NM_002128#(-s-s-:13_H2887168653732) (Splooce)

For more details about the Alternative Splicing Event -> Link to Splooce page

Peptides that support the ASE (Splooce-specific):
WNNTAADDK (MAXQUANT)
WNNTAADDKQPYEK (MAXQUANT + PEAKS)

Alignment:
Uniprot       MGKGDPKKPRGKMSSYAFFVQTCREEHKKKHPDASVNFSEFSKKCSERWKTMSAKEKGKFEDMAKADKARYEREMKTYIPPKGETKKKFKDPNAPKRPPS
Splooce       ----------------------------------------------------------------------------------------------------

Uniprot       AFFLFCSEYRPKIKGEHPGLSIGDVAKKLGEMWNNTAADDKQPYEKKAAKLKEKYEKDIAAYRAKGKPDAAKKGVVKAEKSKKKKEEEEDEEDEEDEEEE
Splooce       -------------------------------MWNNTAADDKQPYEKKAAKLKEKYEKDIAAYRAKGKPDAAKKGVVKAEKSKKKKEEEEDEEDEEDEEEE

Uniprot       EDEEDEDEEEDDDDE
Splooce       EDEEDEDEEEDDDDE

----------------------------------------------------------------------------------------------------

Q9Y4L1 (Uniprot)	versus
NM_001130991#(-t:11_H9370691807938) (Splooce)

For more details about the Alternative Splicing Event -> Link to Splooce page

Peptides that support the ASE (Splooce-specific):
SVDLGSESMK (MAXQUANT + PEAKS)

Alignment:
Uniprot       MADKVRRQRPRRRVCWALVAVLLADLLALSDTLAVMSVDLGSESMKVAIVKPGVPMEIVLNKESRRKTPVIVTLKENERFFGDSAASMAIKNPKATLRYF
Splooce       -----------------------------------MSVDLGSESMKVAIVKPGVPMEIVLNKESRRKTPVIVTLKENERFFGDSAASMAIKNPKATLRYF

Uniprot       QHLLGKQADNPHVALYQARFPEHELTFDPQRQTVHFQISSQLQFSPEEVLGMVLNYSRSLAEDFAEQPIKDAVITVPVFFNQAERRAVLQAARMAGLKVL
Splooce       QHLLGKQADNPHVALYQARFPEHELTFDPQRQTVHFQISSQLQFSPEEVLGMVLNYSRSLAEDFAEQPIKDAVITVPVFFNQAERRAVLQAARMAGLKVL

Uniprot       QLINDNTATALSYGVFRRKDINTTAQNIMFYDMGSGSTVCTIVTYQMVKTKEAGMQPQLQIRGVGFDRTLGGLEMELRLRERLAGLFNEQRKGQRAKDVR
Splooce       QLINDNTATALSYGVFRRKDINTTAQNIMFYDMGSGSTVCTIVTYQMVKTKEAGMQPQLQIRGVGFDRTLGGLEMELRLRERLAGLFNEQRKGQRAKDVR

Uniprot       ENPRAMAKLLREANRLKTVLSANADHMAQIEGLMDDVDFKAKVTRVEFEELCADLFERVPGPVQQALQSAEMSLDEIEQVILVGGATRVPRVQEVLLKAV
Splooce       ENPRAMAKLLREANRLKTVLSANADHMAQIEGLMDDVDFKAKVTRVEFEELCADLFERVPGPVQQALQSAEMSLDEIEQVILVGGATRVPRVQEVLLKAV

Uniprot       GKEELGKNINADEAAAMGAVYQAAALSKAFKVKPFVVRDAVVYPILVEFTREVEEEPGIHSLKHNKRVLFSRMGPYPQRKVITFNRYSHDFNFHINYGDL
Splooce       GKEELGKNINADEAAAMGAVYQAAALSKAFKVKPFVVRDAVVYPILVEFTREVEEEPGIHSLKHNKRVLFSRMGPYPQRKVITFNRYSHDFNFHINYGDL

Uniprot       GFLGPEDLRVFGSQNLTTVKLKGVGDSFKKYPDYESKGIKAHFNLDESGVLSLDRVESVFETLVEDSAEEESTLTKLGNTISSLFGGGTTPDAKENGTDT
Splooce       GFLGPEDLRVFGSQNLTTVKLKGVGDSFKKYPDYESKGIKAHFNLDESGVLSLDRVESVFETLVEDSAEEESTLTKLGNTISSLFGGGTTPDAKENGTDT

Uniprot       VQEEEESPAEGSKDEPGEQVELKEEAEAPVEDGSQPPPPEPKGDATPEGEKATEKENGDKSEAQKPSEKAEAGPEGVAPAPEGEKKQKPARKRRMVEEIG
Splooce       VQEEEESPAEGSKDEPGEQVELKEEAEAPVEDGSQPPPPEPKGDATPEGEKATEKENGDKSEAQKPSEKAEAGPEGVAPAPEGEKKQKPARKRRMVEEIG

Uniprot       VELVVLDLPDLPEDKLAQSVQKLQDLTLRDLEKQEREKAANSLEAFIFETQDKLYQPEYQEVSTEEQREEISGKLSAASTWLEDEGVGATTVMLKEKLAE
Splooce       VELVVLDLPDLPEDKLAQSVQKLQDLTLRDLEKQEREKAANSLEAFIFETQDKLYQPEYQEVSTEEQREEISGKLSAASTWLEDEGVGATTVMLKEKLAE

Uniprot       LRKLCQGLFFRVEERKKWPERLSALDNLLNHSSMFLKGARLIPEMDQIFTEVEMTTLEKVINETWAWKNATLAEQAKLPATEKPVLLSKDIEAKMMALDR
Splooce       LRKLCQGLFFRVEERKKWPERLSALDNLLNHSSMFLKGARLIPEMDQIFTEVEMTTLEKVINETWAWKNATLAEQAKLPATEKPVLLSKDIEAKMMALDR

Uniprot       EVQYLLNKAKFTKPRPRPKDKNGTRAEPPLNASASDQGEKVIPPAGQTEDAEPISEPEKVETGSEPGDTEPLELGGPGAEPEQKEQSTGQKRPLKNDEL
Splooce       EVQYLLNKAKFTKPRPRPKDKNGTRAEPPLNASASDQGEKVIPPAGQTEDAEPISEPEKVETGSEPGDTEPLELGGPGAEPEQKEQSTGQKRPLKNDEL

----------------------------------------------------------------------------------------------------

Q14764 (Uniprot)	versus
NM_005115#(-s-s-s-s-:16_M9875086112532) (Splooce)

For more details about the Alternative Splicing Event -> Link to Splooce page

Peptides that support the ASE (Splooce-specific):
LTQDEVLWEK (PEAKS)
MLTQDEVLWEK (MAXQUANT)

Alignment:
Uniprot       MATEEFIIRIPPYHYIHVLDQNSNVSRVEVGPKTYIRQDNERVLFAPMRMVTVPPRHYCTVANPVSRDAQGLVLFDVTGQVRLRHADLEIRLAQDPFPLY
Splooce       ----------------------------------------------------------------------------------------------------

Uniprot       PGEVLEKDITPLQVVLPNTALHLKALLDFEDKDGDKVVAGDEWLFEGPGTYIPRKEVEVVEIIQATIIRQNQALRLRARKECWDRDGKERVTGEEWLVTT
Splooce       ----------------------------------------------------------------------------------------------------

Uniprot       VGAYLPAVFEEVLDLVDAVILTEKTALHLRARRNFRDFRGVSRRTGEEWLVTVQDTEAHVPDVHEEVLGVVPITTLGPHNYCVILDPVGPDGKNQLGQKR
Splooce       ----------------------------------------------------------------------------------------------------

Uniprot       VVKGEKSFFLQPGEQLEQGIQDVYVLSEQQGLLLRALQPLEEGEDEEKVSHQAGDHWLIRGPLEYVPSAKVEVVEERQAIPLDENEGIYVQDVKTGKVRA
Splooce       ----------------------------------------------------------------------------------------------------

Uniprot       VIGSTYMLTQDEVLWEKELPPGVEELLNKGQDPLADRGEKDTAKSLQPLAPRNKTRVVSYRVPHNAAVQVYDYREKRARVVFGPELVSLGPEEQFTVLSL
Splooce       ------MLTQDEVLWEKELPPGVEELLNKGQDPLADRGEKDTAKSLQPLAPRNKTRVVSYRVPHNAAVQVYDYREKRARVVFGPELVSLGPEEQFTVLSL

Uniprot       SAGRPKRPHARRALCLLLGPDFFTDVITIETADHARLQLQLAYNWHFEVNDRKDPQETAKLFSVPDFVGDACKAIASRVRGAVASVTFDDFHKNSARIIR
Splooce       SAGRPKRPHARRALCLLLGPDFFTDVITIETADHARLQLQLAYNWHFEVNDRKDPQETAKLFSVPDFVGDACKAIASRVRGAVASVTFDDFHKNSARIIR

Uniprot       TAVFGFETSEAKGPDGMALPRPRDQAVFPQNGLVVSSVDVQSVEPVDQRTRDALQRSVQLAIEITTNSQEAAAKHEAQRLEQEARGRLERQKILDQSEAE
Splooce       TAVFGFETSEAKGPDGMALPRPRDQAVFPQNGLVVSSVDVQSVEPVDQRTRDALQRSVQLAIEITTNSQEAAAKHEAQRLEQEARGRLERQKILDQSEAE

Uniprot       KARKELLELEALSMAVESTGTAKAEAESRAEAARIEGEGSVLQAKLKAQALAIETEAELQRVQKVRELELVYARAQLELEVSKAQQLAEVEVKKFKQMTE
Splooce       KARKELLELEALSMAVESTGTAKAEAESRAEAARIEGEGSVLQAKLKAQALAIETEAELQRVQKVRELELVYARAQLELEVSKAQQLAEVEVKKFKQMTE

Uniprot       AIGPSTIRDLAVAGPEMQVKLLQSLGLKSTLITDGSTPINLFNTAFGLLGMGPEGQPLGRRVASGPSPGEGISPQSAQAPQAPGDNHVVPVLR
Splooce       AIGPSTIRDLAVAGPEMQVKLLQSLGLKSTLITDGSTPINLFNTAFGLLGMGPEGQPLGRRVASGPSPGEGISPQSAQAPQAPGDNHVVPVLR

----------------------------------------------------------------------------------------------------

P63261 (Uniprot)	versus
NM_001614#(r:17_A4048171743981) (Splooce)

For more details about the Alternative Splicing Event -> Link to Splooce page

Peptides that support the ASE (Splooce-specific):
MASHHTFYNELR (MAXQUANT)
ASHHTFYNELR (MAXQUANT)

Alignment:
Uniprot       MEEEIAALVIDNGSGMCKAGFAGDDAPRAVFPSIVGRPRHQGVMVGMGQKDSYVGDEAQSKRGILTLKYPIEHGIVTNWDDMEKIWHHTFYNELRVAPEE
Splooce       ----------------------------------------------------------------------MAS-------------HHTFYNELRVAPEE

Uniprot       HPVLLTEAPLNPKANREKMTQIMFETFNTPAMYVAIQAVLSLYASGRTTGIVMDSGDGVTHTVPIYEGYALPHAILRLDLAGRDLTDYLMKILTERGYSF
Splooce       HPVLLTEAPLNPKANREKMTQIMFETFNTPAMYVAIQAVLSLYASGRTTGIVMDSGDGVTHTVPIYEGYALPHAILRLDLAGRDLTDYLMKILTERGYSF

Uniprot       TTTAEREIVRDIKEKLCYVALDFEQEMATAASSSSLEKSYELPDGQVITIGNERFRCPEALFQPSFLGMESCGIHETTFNSIMKCDVDIRKDLYANTVLS
Splooce       TTTAEREIVRDIKEKLCYVALDFEQEMATAASSSSLEKSYELPDGQVITIGNERFRCPEALFQPSFLGMESCGIHETTFNSIMKCDVDIRKDLYANTVLS

Uniprot       GGTTMYPGIADRMQKEITALAPSTMKIKIIAPPERKYSVWIGGSILASLSTFQQMWISKQEYDESGPSIVHRKCF
Splooce       GGTTMYPGIADRMQKEITALAPSTMKIKIIAPPERKYSVWIGGSILASLSTFQQMWISKQEYDESGPSIVHRKCF

----------------------------------------------------------------------------------------------------

Q96TA1 (Uniprot)	versus
NM_022833#(-s-:9_F9230019853826) (Splooce)

For more details about the Alternative Splicing Event -> Link to Splooce page

Peptides that support the ASE (Splooce-specific):
MVPTSQGFTEVR (MAXQUANT)

Alignment:
Uniprot       MGDVLSTHLDDARRQHIAEKTGKILTEFLQFYEDQYGVALFNSMRHEIEGTGLPQAQLLWRKVPLDERIVFSGNLFQHQEDSKKWRNRFSLVPHNYGLVL
Splooce       ----------------------------------------------------------------------------------------------------

Uniprot       YENKAAYERQVPPRAVINSAGYKILTSVDQYLELIGNSLPGTTAKSGSAPILKCPTQFPLILWHPYARHYYFCMMTEAEQDKWQAVLQDCIRHCNNGIPE
Splooce       ----------------------------------------------------------------------------------------------------

Uniprot       DSKVEGPAFTDAIRMYRQSKELYGTWEMLCGNEVQILSNLVMEELGPELKAELGPRLKGKPQERQRQWIQISDAVYHMVYEQAKARFEEVLSKVQQVQPA
Splooce       ----------------------------------------------------------------------------------------------------

Uniprot       MQAVIRTDMDQIITSKEHLASKIRAFILPKAEVCVRNHVQPYIPSILEALMVPTSQGFTEVRDVFFKEVTDMNLNVINEGGIDKLGEYMEKLSRLAYHPL
Splooce       --------------------------------------------------MVPTSQGFTEVRDVFFKEVTDMNLNVINEGGIDKLGEYMEKLSRLAYHPL

Uniprot       KMQSCYEKMESLRLDGLQQRFDVSSTSVFKQRAQIHMREQMDNAVYTFETLLHQELGKGPTKEELCKSIQRVLERVLKKYDYDSSSVRKRFFREALLQIS
Splooce       KMQSCYEKMESLRLDGLQQRFDVSSTSVFKQRAQIHMREQMDNAVYTFETLLHQELGKGPTKEELCKSIQRVLERVLKKYDYDSSSVRKRFFREALLQIS

Uniprot       IPFLLKKLAPTCKSELPRFQELIFEDFARFILVENTYEEVVLQTVMKDILQAVKEAAVQRKHNLYRDSMVMHNSDPNLHLLAEGAPIDWGEEYSNSGGGG
Splooce       IPFLLKKLAPTCKSELPRFQELIFEDFARFILVENTYEEVVLQTVMKDILQAVKEAAVQRKHNLYRDSMVMHNSDPNLHLLAEGAPIDWGEEYSNSGGGG

Uniprot       SPSPSTPESATLSEKRRRAKQVVSVVQDEEVGLPFEASPESPPPASPDGVTEIRGLLAQGLRPESPPPAGPLLNGAPAGESPQPKAAPEASSPPASPLQH
Splooce       SPSPSTPESATLSEKRRRAKQVVSVVQDEEVGLPFEASPESPPPASPDGVTEIRGLLAQGLRPESPPPAGPLLNGAPAGESPQPKAAPEASSPPASPLQH

Uniprot       LLPGKAVDLGPPKPSDQETGEQVSSPSSHPALHTTTEDSAGVQTEF
Splooce       LLPGKAVDLGPPKPSDQETGEQVSSPSSHPALHTTTEDSAGVQTEF

----------------------------------------------------------------------------------------------------

Q5VU43 (Uniprot)	versus
NM_014644#(-s-s-s-:1_P3934549846068) (Splooce)

For more details about the Alternative Splicing Event -> Link to Splooce page

Peptides that support the ASE (Splooce-specific):
MLEGLVDER (MAXQUANT)

Alignment:
Uniprot       MSNGYRTLSQHLNDLKKENFSLKLRIYFLEERMQQKYEASREDIYKRNIELKVEVESLKRELQDKKQHLDKTWADVENLNSQNEAELRRQFEERQQETEH
Splooce       ----------------------------------------------------------------------------------------------------

Uniprot       VYELLENKIQLLQEESRLAKNEAARMAALVEAEKECNLELSEKLKGVTKNWEDVPGDQVKPDQYTEALAQRDKRIEELNQSLAAQERLVEQLSREKQQLL
Splooce       ----------------------------------------------------------------------------------------------------

Uniprot       HLLEEPTSMEVQPMTEELLKQQKLNSHETTITQQSVSDSHLAELQEKIQQTEATNKILQEKLNEMSYELKCAQESSQKQDGTIQNLKETLKSRERETEEL
Splooce       ----------------------------------------------------------------------------------------------------

Uniprot       YQVIEGQNDTMAKLREMLHQSQLGQLHSSEGTSPAQQQVALLDLQSALFCSQLEIQKLQRVVRQKERQLADAKQCVQFVEAAAHESEQQKEASWKHNQEL
Splooce       ----------------------------------------------------------------------------------------------------

Uniprot       RKALQQLQEELQNKSQQLRAWEAEKYNEIRTQEQNIQHLNHSLSHKEQLLQEFRELLQYRDNSDKTLEANEMLLEKLRQRIHDKAVALERAIDEKFSALE
Splooce       ----------------------------------------------------------------------------------------------------

Uniprot       EKEKELRQLRLAVRERDHDLERLRDVLSSNEATMQSMESLLRAKGLEVEQLSTTCQNLQWLKEEMETKFSRWQKEQESIIQQLQTSLHDRNKEVEDLSAT
Splooce       ----------------------------------------------------------------------------------------------------

Uniprot       LLCKLGPGQSEIAEELCQRLQRKERMLQDLLSDRNKQVLEHEMEIQGLLQSVSTREQESQAAAEKLVQALMERNSELQALRQYLGGRDSLMSQAPISNQQ
Splooce       ----------------------------------------------------------------------------------------------------

Uniprot       AEVTPTGCLGKQTDQGSMQIPSRDDSTSLTAKEDVSIPRSTLGDLDTVAGLEKELSNAKEELELMAKKERESQMELSALQSMMAVQEEELQVQAADMESL
Splooce       ----------------------------------------------------------------------------------------------------

Uniprot       TRNIQIKEDLIKDLQMQLVDPEDIPAMERLTQEVLLLREKVASVESQGQEISGNRRQQLLLMLEGLVDERSRLNEALQAERQLYSSLVKFHAHPESSERD
Splooce       -------------------------------------------------------------MLEGLVDERSRLNEALQAERQLYSSLVKFHAHPESSERD

Uniprot       RTLQVELEGAQVLRSRLEEVLGRSLERLNRLETLAAIGGAAAGDDTEDTSTEFTDSIEEEAAHHSHQQLVKVALEKSLATVETQNPSFSPPSPMGGDSNR
Splooce       RTLQVELEGAQVLRSRLEEVLGRSLERLNRLETLAAIGGAAAGDDTEDTSTEFTDSIEEEAAHHSHQQLVKVALEKSLATVETQNPSFSPPSPMGGDSNR

Uniprot       CLQEEMLHLRAEFHQHLEEKRKAEEELKELKAQIEEAGFSSVSHIRNTMLSLCLENAELKEQMGEAMSDGWEIEEDKEKGEVMVETVVTKEGLSESSLQA
Splooce       CLQEEMLHLRAEFHQHLEEKRKAEEELKELKAQIEEAGFSSVSHIRNTMLSLCLENAELKEQMGEAMSDGWEIEEDKEKGEVMVETVVTKEGLSESSLQA

Uniprot       EFRKLQGKLKNAHNIINLLKEQLVLSSKEGNSKLTPELLVHLTSTIERINTELVGSPGKHQHQEEGNVTVRPFPRPQSLDLGATFTVDAHQLDNQSQPRD
Splooce       EFRKLQGKLKNAHNIINLLKEQLVLSSKEGNSKLTPELLVHLTSTIERINTELVGSPGKHQHQEEGNVTVRPFPRPQSLDLGATFTVDAHQLDNQSQPRD

Uniprot       PGPQSAFSLPGSTQHLRSQLSQCKQRYQDLQEKLLLSEATVFAQANELEKYRVMLTGESLVKQDSKQIQVDLQDLGYETCGRSENEAEREETTSPECEEH
Splooce       PGPQSAFSLPGSTQHLRSQLSQCKQRYQDLQEKLLLSEATVFAQANELEKYRVMLTGESLVKQDSKQIQVDLQDLGYETCGRSENEAEREETTSPECEEH

Uniprot       NSLKEMVLMEGLCSEQGRRGSTLASSSERKPLENQLGKQEEFRVYGKSENILVLRKDIKDLKAQLQNANKVIQNLKSRVRSLSVTSDYSSSLERPWKLRA
Splooce       NSLKEMVLMEGLCSEQGRRGSTLASSSERKPLENQLGKQEEFRVYGKSENILVLRKDIKDLKAQLQNANKVIQNLKSRVRSLSVTSDYSSSLERPWKLRA

Uniprot       VGTLEGSSPHSVPDEDEGWLSDGTGAFYSPGLQAKKDLESLIQRVSQLEAQLPKNGLEEKLAEELRSASWPGKYDSLIQDQARELSYLRQKIREGRGICY
Splooce       VGTLEGSSPHSVPDEDEGWLSDGTGAFYSPGLQAKKDLESLIQRVSQLEAQLPKNGLEEKLAEELRSASWPGKYDSLIQDQARELSYLRQKIREGRGICY

Uniprot       LITRHAKDTVKSFEDLLRSNDIDYYLGQSFREQLAQGSQLTERLTSKLSTKDHKSEKDQAGLEPLALRLSRELQEKEKVIEVLQAKLDARSLTPSSSHAL
Splooce       LITRHAKDTVKSFEDLLRSNDIDYYLGQSFREQLAQGSQLTERLTSKLSTKDHKSEKDQAGLEPLALRLSRELQEKEKVIEVLQAKLDARSLTPSSSHAL

Uniprot       SDSHRSPSSTSFLSDELEACSDMDIVSEYTHYEEKKASPSHSDSIHHSSHSAVLSSKPSSTSASQGAKAESNSNPISLPTPQNTPKEANQAHSGFHFHSI
Splooce       SDSHRSPSSTSFLSDELEACSDMDIVSEYTHYEEKKASPSHSDSIHHSSHSAVLSSKPSSTSASQGAKAESNSNPISLPTPQNTPKEANQAHSGFHFHSI

Uniprot       PKLASLPQAPLPSAPSSFLPFSPTGPLLLGCCETPVVSLAEAQQELQMLQKQLGESASTVPPASTATLLSNDLEADSSYYLNSAQPHSPPRGTIELGRIL
Splooce       PKLASLPQAPLPSAPSSFLPFSPTGPLLLGCCETPVVSLAEAQQELQMLQKQLGESASTVPPASTATLLSNDLEADSSYYLNSAQPHSPPRGTIELGRIL

Uniprot       EPGYLGSSGKWDVMRPQKGSVSGDLSSGSSVYQLNSKPTGADLLEEHLGEIRNLRQRLEESICINDRLREQLEHRLTSTARGRGSTSNFYSQGLESIPQL
Splooce       EPGYLGSSGKWDVMRPQKGSVSGDLSSGSSVYQLNSKPTGADLLEEHLGEIRNLRQRLEESICINDRLREQLEHRLTSTARGRGSTSNFYSQGLESIPQL

Uniprot       CNENRVLREDNRRLQAQLSHVSREHSQETESLREALLSSRSHLQELEKELEHQKVERQQLLEDLREKQQEVLHFREERLSLQENDSRLQHKLVLLQQQCE
Splooce       CNENRVLREDNRRLQAQLSHVSREHSQETESLREALLSSRSHLQELEKELEHQKVERQQLLEDLREKQQEVLHFREERLSLQENDSRLQHKLVLLQQQCE

Uniprot       EKQQLFESLQSELQIYEALYGNSKKGLKAYSLDACHQIPLSSDLSHLVAEVRALRGQLEQSIQGNNCLRLQLQQQLESGAGKASLSPSSINQNFPASTDP
Splooce       EKQQLFESLQSELQIYEALYGNSKKGLKAYSLDACHQIPLSSDLSHLVAEVRALRGQLEQSIQGNNCLRLQLQQQLESGAGKASLSPSSINQNFPASTDP

Uniprot       GNKQLLLQDSAVSPPVRDVGMNSPALVFPSSASSTPGSETPIINRANGLGLDTSPVMKTPPKLEGDATDGSFANKHGRHVIGHIDDYSALRQQIAEGKLL
Splooce       GNKQLLLQDSAVSPPVRDVGMNSPALVFPSSASSTPGSETPIINRANGLGLDTSPVMKTPPKLEGDATDGSFANKHGRHVIGHIDDYSALRQQIAEGKLL

Uniprot       VKKIVSLVRSACSFPGLEAQGTEVLGSKGIHELRSSTSALHHALEESASLLTMFWRAALPSTHIPVLPGKVGESTERELLELRTKVSKQERLLQSTTEHL
Splooce       VKKIVSLVRSACSFPGLEAQGTEVLGSKGIHELRSSTSALHHALEESASLLTMFWRAALPSTHIPVLPGKVGESTERELLELRTKVSKQERLLQSTTEHL

Uniprot       KNANQQKESMEQFIVSQLTRTHDVLKKARTNLEVKSLRALPCTPAL
Splooce       KNANQQKESMEQFIVSQLTRTHDVLKKARTNLEVKSLRALPCTPAL

----------------------------------------------------------------------------------------------------

UNIPROT? (Uniprot)	versus
NM_001039917#(r:12_Z7660615036100) (Splooce)

For more details about the Alternative Splicing Event -> Link to Splooce page

Peptides that support the ASE (Splooce-specific):
IVSALPPGSQALQVVPDLSK (MAXQUANT)

Alignment:
Uniprot       -------
Splooce       ----------------------------------------------------------------------------------------------------

Uniprot       -------
Splooce       ---------------------------------------------MIVSALPPGSQALQVVPDLSKKVASTLTEEGGGGGGGGGSVAPKPPRGRKKKRML

Uniprot       -------
Splooce       ESGLPEMNDPYVLSPEDDDDHQKDGKTYRCRMCSLTFYSKSEMQIHSKSHTETKPHKCPHCSKTFANSSYLAQHIRIHSGAKPYSCNFCEKSFRQLSHLQ

Uniprot       -------
Splooce       QHTRIHTGDRPYKCAHPGCEKAFTQLSNLQSHRRQHNKDKPFKCHNCHRAYTDAASLEVHLSTHTVKHAKVYTCTICSRAYTSETYLMKHMRKHNPPDLQ

Uniprot       -------
Splooce       QQVQAAAAAAAVAQAQAQAQAQAQAQAQAQAQAQASQASQQQQQQQQQQQQQQQQPPPHFQSPGAAPQGGGGGDSNPNPPPQCSFDLTPYKTAEHHKDIC

Uniprot       -------
Splooce       LTVTTSTIQVEHLASS

----------------------------------------------------------------------------------------------------

Q05682 (Uniprot)	versus
NM_033157#(-s-:7_C171399389673) (Splooce)

For more details about the Alternative Splicing Event -> Link to Splooce page

Peptides that support the ASE (Splooce-specific):
MIGGMLK (MAXQUANT)

Alignment:
Uniprot       MDDFERRRELRRQKREEMRLEAERIAYQRNDDDEEEAARERRRRARQERLRQKQEEESLGQVTDQVEVNAQNSVPDEEAKTTTTNTQVEGDDEAAFLERL
Splooce       ----MIGGMLKKTRKK---------------------TRKRRRRKRR--------------------------------------SQSEG----------

Uniprot       ARREERRQKRLQEALERQKEFDPTITDASLSLPSRRMQNDTAENETTEKEEKSESRQERYEIEETETVTKSYQKNDWRDAEENKKEDKEKEEEEEEKPKR
Splooce       -------------ALEKIR----------------------------------EKRRE------------------------------------------

Uniprot       GSIGENQGEEKGTKVQAKREKLQEDKPTFKKEEIKDEKIKKDKEPKEEVKSFMDRKKGFTEVKSQNGEFMTHKLKHTENTFSRPGGRASVDTKEAEGAPQ
Splooce       -----------------------------LKCKLKEKSSKKTSLP--------SKKK------------------------SRPGGRASVDTKEAEGAPQ

Uniprot       VEAGKRLEELRRRRGETESEEFEKLKQKQQEAALELEELKKKREERRKVLEEEEQRRKQEEADRKLREEEEKRRLKEEIERRRAEAAEKRQKMPEDGLSD
Splooce       VEAGKRLEELRRRRGETESEEFEKLKQKQQEAALELEELKKKREERRKVLEEEEQRRKQEEADRKLREEEEKRRLKEEIERRRAEAAEKRQKMPEDGLSD

Uniprot       DKKPFKCFTPKGSSLKIEERAEFLNKSVQKSSGVKSTHQAAIVSKIDSRLEQYTSAIEGTKSAKPTKPAASDLPVPAEGVRNIKSMWEKGNVFSSPTAAG
Splooce       DKKPFKCFTPKGSSLKIEERAEFLNKSVQKSSGVKSTHQAAIVSKIDSRLEQYTSAIEGTKSAKPTKPAASDLPVPAEGVRNIKSMWEKGNVFSSPTAAG

Uniprot       TPNKETAGLKVGVSSRINEWLTKTPDGNKSPAPKPSDLRPGDVSSKRNLWEKQSVDKVTSPTKV
Splooce       TPNKETAGLKVGVSSRINEWLTKTPDGNKSPAPKPSDLRPGDVSSKRNLWEKQSVDKVTSPTKV

----------------------------------------------------------------------------------------------------

P00558 (Uniprot)	versus
NM_000291#(f-T:X_P3853256651548) (Splooce)

For more details about the Alternative Splicing Event -> Link to Splooce page

Peptides that support the ASE (Splooce-specific):
SHLGRPDGVPMPDK (MAXQUANT)

Alignment:
Uniprot       MSLSNKLTLDKLDVKGKRVVMRVDFNVPMKNNQITNNQRIKAAVPSIKFCLDNGAKSVVLMSHLGRPDGVPMPDKYSLEPVAVELKSLLGKDVLFLKDCV
Splooce       ------------------------------------------------------------MSHLGRPDGVPMPDKYSLEPVAVELKSLLGKDVLFLKDCV

Uniprot       GPEVEKACANPAAGSVILLENLRFHVEEEGKGKDASGNKVKAEPAKIEAFRASLSKLGDVYVNDAFGTAHRAHSSMVGVNLPQKAGGFLMKKELNYFAKA
Splooce       GPEVEKACANPAAGSVILLENLRFHVEEEGKGKDASGNKVKAEPAKIEAFRASLSKLGDVYVNDAFGTAHRAHSSMVGVNLPQKAGGFLMKKELNYFAKA

Uniprot       LESPERPFLAILGGAKVADKIQLINNMLDKVNEMIIGGGMAFTFLKVLNNMEIGTSLFDEEGAKIVKDLMSKAEKNGVKITLPVDFVTADKFDENAKTGQ
Splooce       LESPERPFLAILGGAKVADKIQLINNMLDKVNEMIIGGGMAFTFLKVLNNMEIGTSLFDEEGAKIVKDLMSKAEKNGVKITLPVDFVTADKFDENAKTGQ

Uniprot       ATVASGIPAGWMGLDCGPESSKKYAEAVTRAKQIVWNGPVGVFEWEAFARGTKALMDEVVKATSRGCITIIGGGDTATCCAKWNTEDKVSHVSTGGGASL
Splooce       ATVASGIPAGWMGLDCGPESSKKYAEAVTRAKQIVWNGPVGVFEWEAFARGTKALMDEVVKATSRGCITIIGGGDTATCCAKWNTEDKVSHVSTGGGASL

Uniprot       ELLEGKVLPGVDALSNI
Splooce       ELLEGKVLPGVDALSNI

----------------------------------------------------------------------------------------------------

Q14697 (Uniprot)	versus
NM_198334#(-s-s-s-s-s-s-s-s-s-:11_G2068348209120) (Splooce)

For more details about the Alternative Splicing Event -> Link to Splooce page

Peptides that support the ASE (Splooce-specific):
MATADGLR (MAXQUANT + PEAKS)

Alignment:
Uniprot       MAAVAAVAARRRRSWASLVLAFLGVCLGITLAVDRSNFKTCEESSFCKRQRSIRPGLSPYRALLDSLQLGPDSLTVHLIHEVTKVLLVLELQGLQKNMTR
Splooce       ----------------------------------------------------------------------------------------------------

Uniprot       FRIDELEPRRPRYRVPDVLVADPPIARLSVSGRDENSVELTMAEGPYKIILTARPFRLDLLEDRSLLLSVNARGLLEFEHQRAPRVSQGSKDPAEGDGAQ
Splooce       ----------------------------------------------------------------------------------------------------

Uniprot       PEETPRDGDKAKETQGKAEKDEPGAWEETFKTHSDSKPYGPMSVGLDFSLPGMEHVYGIPEHADNLRLKVTEGGEPYRLYNLDVFQYELYNPMALYGSVP
Splooce       ----------------------------------------------------------------------------------------------------

Uniprot       VLLAHNPHRDLGIFWLNAAETWVDISSNTAGKTLFGKMMDYLQGSGETPQTDVRWMSETGIIDVFLLLGPSISDVFRQYASLTGTQALPPLFSLGYHQSR
Splooce       ----------------------------------------------------------------------------------------------------

Uniprot       WNYRDEADVLEVDQGFDDHNLPCDVIWLDIEHADGKRYFTWDPSRFPQPRTMLERLASKRRKLVAIVDPHIKVDSGYRVHEELRNLGLYVKTRDGSDYEG
Splooce       ----------------------------------------------------------------------------------------------------

Uniprot       WCWPGSAGYPDFTNPTMRAWWANMFSYDNYEGSAPNLFVWNDMNEPSVFNGPEVTMLKDAQHYGGWEHRDVHNIYGLYVHMATADGLRQRSGGMERPFVL
Splooce       --------------------------------------------------------------------------------MATADGLRQRSGGMERPFVL

Uniprot       ARAFFAGSQRFGAVWTGDNTAEWDHLKISIPMCLSLGLVGLSFCGADVGGFFKNPEPELLVRWYQMGAYQPFFRAHAHLDTGRREPWLLPSQHNDIIRDA
Splooce       ARAFFAGSQRFGAVWTGDNTAEWDHLKISIPMCLSLGLVGLSFCGADVGGFFKNPEPELLVRWYQMGAYQPFFRAHAHLDTGRREPWLLPSQHNDIIRDA

Uniprot       LGQRYSLLPFWYTLLYQAHREGIPVMRPLWVQYPQDVTTFNIDDQYLLGDALLVHPVSDSGAHGVQVYLPGQGEVWYDIQSYQKHHGPQTLYLPVTLSSI
Splooce       LGQRYSLLPFWYTLLYQAHREGIPVMRPLWVQYPQDVTTFNIDDQYLLGDALLVHPVSDSGAHGVQVYLPGQGEVWYDIQSYQKHHGPQTLYLPVTLSSI

Uniprot       PVFQRGGTIVPRWMRVRRSSECMKDDPITLFVALSPQGTAQGELFLDDGHTFNYQTRQEFLLRRFSFSGNTLVSSSADPEGHFETPIWIERVVIIGAGKP
Splooce       PVFQRGGTIVPRWMRVRRSSECMKDDPITLFVALSPQGTAQGELFLDDGHTFNYQTRQEFLLRRFSFSGNTLVSSSADPEGHFETPIWIERVVIIGAGKP

Uniprot       AAVVLQTKGSPESRLSFQHDPETSVLVLRKPGINVASDWSIHLR
Splooce       AAVVLQTKGSPESRLSFQHDPETSVLVLRKPGINVASDWSIHLR

----------------------------------------------------------------------------------------------------

P21912 (Uniprot)	versus
NM_003000#(f-:1_S1399885915606) (Splooce)

For more details about the Alternative Splicing Event -> Link to Splooce page

Peptides that support the ASE (Splooce-specific):
VLDALLK (PEAKS)
VLDALIK (MAXQUANT)

Alignment:
Uniprot       MAAVVALSLRRRLPATTLGGACLQASRGAQTAAATAPRIKKFAIYRWDPDKAGDKPHMQTYEVDLNKCGPMVLDALIKIKNEVDSTLTFRRSCREGICGS
Splooce       ----------------------------------------------------------------------MVLDALIKIKNEVDSTLTFRRSCREGICGS

Uniprot       CAMNINGGNTLACTRRIDTNLNKVSKIYPLPHMYVIKDLVPDLSNFYAQYKSIEPYLKKKDESQEGKQQYLQSIEEREKLDGLYECILCACCSTSCPSYW
Splooce       CAMNINGGNTLACTRRIDTNLNKVSKIYPLPHMYVIKDLVPDLSNFYAQYKSIEPYLKKKDESQEGKQQYLQSIEEREKLDGLYECILCACCSTSCPSYW

Uniprot       WNGDKYLGPAVLMQAYRWMIDSRDDFTEERLAKLQDPFSLYRCHTIMNCTRTCPKGLNPGKAIAEIKKMMATYKEKKASV
Splooce       WNGDKYLGPAVLMQAYRWMIDSRDDFTEERLAKLQDPFSLYRCHTIMNCTRTCPKGLNPGKAIAEIKKMMATYKEKKASV

----------------------------------------------------------------------------------------------------

Q9BUK6 (Uniprot)	versus
NM_018116#(-s-s-:1_M2967965066856) (Splooce)

For more details about the Alternative Splicing Event -> Link to Splooce page

Peptides that support the ASE (Splooce-specific):
VSGGSNPFPMAK (MAXQUANT)

Alignment:
Uniprot       MAGGAREVLTLQLGHFAGFVGAHWWNQQDAALGRATDSKEPPGELCPDVLYRTGRTLHGQETYTPRLILMDLKGSLSSLKEEGGLYRDKQLDAAIAWQGK
Splooce       ----------------------------------------------------------------------------------------------------

Uniprot       LTTHKEELYPKNPYLQDFLSAEGVLSSDGVWRVKSIPNGKGSSPLPTATTPKPLIPTEASIRVWSDFLRVHLHPRSICMIQKYNHDGEAGRLEAFGQGES
Splooce       -----------------------------------MVSG-GSNPFPMA----------------------------------------------------

Uniprot       VLKEPKYQEELEDRLHFYVEECDYLQGFQILCDLHDGFSGVGAKAAELLQDEYSGRGIITWGLLPGPYHRGEAQRNIYRLLNTAFGLVHLTAHSSLVCPL
Splooce       -------------------------KGFQILCDLHDGFSGVGAKAAELLQDEYSGRGIITWGLLPGPYHRGEAQRNIYRLLNTAFGLVHLTAHSSLVCPL

Uniprot       SLGGSLGLRPEPPVSFPYLHYDATLPFHCSAILATALDTVTVPYRLCSSPVSMVHLADMLSFCGKKVVTAGAIIPFPLAPGQSLPDSLMQFGGATPWTPL
Splooce       SLGGSLGLRPEPPVSFPYLHYDATLPFHCSAILATALDTVTVPYRLCSSPVSMVHLADMLSFCGKKVVTAGAIIPFPLAPGQSLPDSLMQFGGATPWTPL

Uniprot       SACGEPSGTRCFAQSVVLRGIDRACHTSQLTPGTPPPSALHACTTGEEILAQYLQQQQPGVMSSSHLLLTPCRVAPPYPHLFSSCSPPGMVLDGSPKGAA
Splooce       SACGEPSGTRCFAQSVVLRGIDRACHTSQLTPGTPPPSALHACTTGEEILAQYLQQQQPGVMSSSHLLLTPCRVAPPYPHLFSSCSPPGMVLDGSPKGAA

Uniprot       VESIPVFGALCSSSSLHQTLEALARDLTKLDLRRWASFMDAGVEHDDVAELLQELQSLAQCYQGGDSLVD
Splooce       VESIPVFGALCSSSSLHQTLEALARDLTKLDLRRWASFMDAGVEHDDVAELLQELQSLAQCYQGGDSLVD

----------------------------------------------------------------------------------------------------

Q9NP58 (Uniprot)	versus
NM_005689#(r:2_A931045604579) (Splooce)

For more details about the Alternative Splicing Event -> Link to Splooce page

Peptides that support the ASE (Splooce-specific):
MFFNAWFGLIVFLCMSLYLTLTIVVTEWR (MAXQUANT)

Alignment:
Uniprot       MVTVGNYCEAEGPVGPAWMQDGLSPCFFFTLVPSTRMALGTLALVLALPCRRRERPAGADSLSWGAGPRISPYVLQLLLATLQAALPLAGLAGRVGTARG
Splooce       ----------------------------------------------------------------------------------------------------

Uniprot       APLPSYLLLASVLESLAGACGLWLLVVERSQARQRLAMGIWIKFRHSPGLLLLWTVAFAAENLALVSWNSPQWWWARADLGQQVQFSLWVLRYVVSGGLF
Splooce       ----------------------------------------------------------------------------------------------------

Uniprot       VLGLWAPGLRPQSYTLQVHEEDQDVERSQVRSAAQQSTWRDFGRKLRLLSGYLWPRGSPALQLVVLICLGLMGLERALNVLVPIFYRNIVNLLTEKAPWN
Splooce       ----------------------------------------------------------------------------------------------------

Uniprot       SLAWTVTSYVFLKFLQGGGTGSTGFVSNLRTFLWIRVQQFTSRRVELLIFSHLHELSLRWHLGRRTGEVLRIADRGTSSVTGLLSYLVFNVIPTLADIII
Splooce       ----------------------------------------------------------------------------------------------------

Uniprot       GIIYFSMFFNAWFGLIVFLCMSLYLTLTIVVTEWRTKFRRAMNTQENATRARAVDSLLNFETVKYYNAESYEVERYREAIIKYQGLEWKSSASLVLLNQT
Splooce       ------MFFNAWFGLIVFLCMSLYLTLTIVVTEWRTKFRRAMNTQENATRARAVDSLLNFETVKYYNAESYEVERYREAIIKYQGLEWKSSASLVLLNQT

Uniprot       QNLVIGLGLLAGSLLCAYFVTEQKLQVGDYVLFGTYIIQLYMPLNWFGTYYRMIQTNFIDMENMFDLLKEETEVKDLPGAGPLRFQKGRIEFENVHFSYA
Splooce       QNLVIGLGLLAGSLLCAYFVTEQKLQVGDYVLFGTYIIQLYMPLNWFGTYYRMIQTNFIDMENMFDLLKEETEVKDLPGAGPLRFQKGRIEFENVHFSYA

Uniprot       DGRETLQDVSFTVMPGQTLALVGPSGAGKSTILRLLFRFYDISSGCIRIDGQDISQVTQASLRSHIGVVPQDTVLFNDTIADNIRYGRVTAGNDEVEAAA
Splooce       DGRETLQDVSFTVMPGQTLALVGPSGAGKSTILRLLFRFYDISSGCIRIDGQDISQVTQASLRSHIGVVPQDTVLFNDTIADNIRYGRVTAGNDEVEAAA

Uniprot       QAAGIHDAIMAFPEGYRTQVGERGLKLSGGEKQRVAIARTILKAPGIILLDEATSALDTSNERAIQASLAKVCANRTTIVVAHRLSTVVNADQILVIKDG
Splooce       QAAGIHDAIMAFPEGYRTQVGERGLKLSGGEKQRVAIARTILKAPGIILLDEATSALDTSNERAIQASLAKVCANRTTIVVAHRLSTVVNADQILVIKDG

Uniprot       CIVERGRHEALLSRGGVYADMWQLQQGQEETSEDTKPQTMER
Splooce       CIVERGRHEALLSRGGVYADMWQLQQGQEETSEDTKPQTMER

----------------------------------------------------------------------------------------------------

P62906 (Uniprot)	versus
NM_007104#(-t:6_R3760248009882) (Splooce)

For more details about the Alternative Splicing Event -> Link to Splooce page

Peptides that support the ASE (Splooce-specific):
MDIEALK (MAXQUANT)
MDLEALK (PEAKS)

Alignment:
Uniprot       MSSKVSRDTLYEAVREVLHGNQRKRRKFLETVELQISLKNYDPQKDKRFSGTVRLKSTPRPKFSVCVLGDQQHCDEAKAVDIPHMDIEALKKLNKNKKLV
Splooce       ------------------------------------------------------------------------------------MDIEALKKLNKNKKLV

Uniprot       KKLAKKYDAFLASESLIKQIPRILGPGLNKAGKFPSLLTHNENMVAKVDEVKSTIKFQMKKVLCLAVAVGHVKMTDDELVYNIHLAVNFLVSLLKKNWQN
Splooce       KKLAKKYDAFLASESLIKQIPRILGPGLNKAGKFPSLLTHNENMVAKVDEVKSTIKFQMKKVLCLAVAVGHVKMTDDELVYNIHLAVNFLVSLLKKNWQN

Uniprot       VRALYIKSTMGKPQRLY
Splooce       VRALYIKSTMGKPQRLY

----------------------------------------------------------------------------------------------------

Q9BQG0 (Uniprot)	versus
NM_014520#(-s-s-s-s-s-s-:17_M1663159480483) (Splooce)

For more details about the Alternative Splicing Event -> Link to Splooce page

Peptides that support the ASE (Splooce-specific):
MALDQSLASLFAEQK (MAXQUANT)

Alignment:
Uniprot       MESRDPAQPMSPGEATQSGARPADRYGLLKHSREFLDFFWDIAKPEQETRLAATEKLLEYLRGRPKGSEMKYALKRLITGLGVGRETARPCYSLALAQLL
Splooce       ----------------------------------------------------------------------------------------------------

Uniprot       QSFEDLPLCSILQQIQEKYDLHQVKKAMLRPALFANLFGVLALFQSGRLVKDQEALMKSVKLLQALAQYQNHLQEQPRKALVDILSEVSKATLQEILPEV
Splooce       ----------------------------------------------------------------------------------------------------

Uniprot       LKADLNIILSSPEQLELFLLAQQKVPSKLKKLVGSVNLFSDENVPRLVNVLKMAASSVKKDRKLPAIALDLLRLALKEDKFPRFWKEVVEQGLLKMQFWP
Splooce       ----------------------------------------------------------------------------------------------------

Uniprot       ASYLCFRLLGAALPLLTKEQLHLVMQGDVIRHYGEHVCTAKLPKQFKFAPEMDDYVGTFLEGCQDDPERQLAVLVAFSSVTNQGLPVTPTFWRVVRFLSP
Splooce       ----------------------------------------------------------------------------------------------------

Uniprot       PALQGYVAWLRAMFLQPDLDSLVDFSTNNQKKAQDSSLHMPERAVFRLRKWIIFRLVSIVDSLHLEMEEALTEQVARFCLFHSFFVTKKPTSQIPETKHP
Splooce       ----------------------------------------------------------------------------------------------------

Uniprot       FSFPLENQAREAVSSAFFSLLQTLSTQFKQAPGQTQGGQPWTYHLVQFADLLLNHSHNVTTVTPFTAQQRQAWDRMLQTLKELEAHSAEARAAAFQHLLL
Splooce       ----------------------------------------------------------------------------------------------------

Uniprot       LVGIHLLKSPAESCDLLGDIQTCIRKSLGEKPRRSRTKTIDPQEPPWVEVLVEILLALLAQPSHLMRQVARSVFGHICSHLTPRALQLILDVLNPETSED
Splooce       ----------------------------------------------------------------------------------------------------

Uniprot       ENDRVVVTDDSDERRLKGAEDKSEEGEDNRSSESEEESEGEESEEEERDGDVDQGFREQLMTVLQAGKALGGEDSENEEELGDEAMMALDQSLASLFAEQ
Splooce       -------------------------------------------------------------------------------------MMALDQSLASLFAEQ

Uniprot       KLRIQARRDEKNKLQKEKALRRDFQIRVLDLVEVLVTKQPENALVLELLEPLLSIIRRSLRSSSSKQEQDLLHKTARIFTHHLCRARRYCHDLGERAGAL
Splooce       KLRIQARRDEKNKLQKEKALRRDFQIRVLDLVEVLVTKQPENALVLELLEPLLSIIRRSLRSSSSKQEQDLLHKTARIFTHHLCRARRYCHDLGERAGAL

Uniprot       HAQVERLVQQAGRQPDSPTALYHFNASLYLLRVLKGNTAEGCVHETQEKQKAGTDPSHMPTGPQAASCLDLNLVTRVYSTALSSFLTKRNSPLTVPMFLS
Splooce       HAQVERLVQQAGRQPDSPTALYHFNASLYLLRVLKGNTAEGCVHETQEKQKAGTDPSHMPTGPQAASCLDLNLVTRVYSTALSSFLTKRNSPLTVPMFLS

Uniprot       LFSRHPVLCQSLLPILVQHITGPVRPRHQACLLLQKTLSMREVRSCFEDPEWKQLMGQVLAKVTENLRVLGEAQTKAQHQQALSSLELLNVLFRTCKHEK
Splooce       LFSRHPVLCQSLLPILVQHITGPVRPRHQACLLLQKTLSMREVRSCFEDPEWKQLMGQVLAKVTENLRVLGEAQTKAQHQQALSSLELLNVLFRTCKHEK

Uniprot       LTLDLTVLLGVLQGQQQSLQQGAHSTGSSRLHDLYWQAMKTLGVQRPKLEKKDAKEIPSATQSPISKKRKKKGFLPETKKRKKRKSEDGTPAEDGTPAAT
Splooce       LTLDLTVLLGVLQGQQQSLQQGAHSTGSSRLHDLYWQAMKTLGVQRPKLEKKDAKEIPSATQSPISKKRKKKGFLPETKKRKKRKSEDGTPAEDGTPAAT

Uniprot       GGSQPPSMGRKKRNRTKAKVPAQANGTPTTKSPAPGAPTRSPSTPAKSPKLQKKNQKPSQVNGAPGSPTEPAGQKQHQKALPKKGVLGKSPLSALARKKA
Splooce       GGSQPPSMGRKKRNRTKAKVPAQANGTPTTKSPAPGAPTRSPSTPAKSPKLQKKNQKPSQVNGAPGSPTEPAGQKQHQKALPKKGVLGKSPLSALARKKA

Uniprot       RLSLVIRSPSLLQSGAKKKAQVRKAGKP
Splooce       RLSLVIRSPSLLQSGAKKKAQVRKAGKP

----------------------------------------------------------------------------------------------------

B5MCW9 (Uniprot)	versus
NM_019063#(-s-:2_E6695792003648) (Splooce)

For more details about the Alternative Splicing Event -> Link to Splooce page

Peptides that support the ASE (Splooce-specific):
SCITNGSGANR (MAXQUANT)

Alignment:
Uniprot       MDGFAGSLDDSISAASTSDVQDRLSALESRVQQQEDEITVLKAALADVLRRLAISEDHVASVKKSVSSKGQPSPRAVIPMSCITNGSGANRKPSHTSAVS
Splooce       -------------------------------------------------------------------------------MSCITNGSGANRKPSHTSAVS

Uniprot       IAGKETLSSAAKSGTEKKKEKPQGQREKKEESHSNDQSPQIRASPSPQPSSQPLQIHRQTPESKNATPTKSIKRPSPAEKSHNSWENSDDSRNKLSKIPS
Splooce       IAGKETLSSAAKSGTEKKKEKPQGQREKKEESHSNDQSPQIRASPSPQPSSQPLQIHRQTPESKNATPTKSIKRPSPAEKSHNSWENSDDSRNKLSKIPS

Uniprot       TPKLIPKVTKTADKHKDVIINQEGEYIKMFMRGRPITMFIPSDVDNYDDIRTELPPEKLKLEWAYGYRGKDCRANVYLLPTGKIVYFIASVVVLFNYEER
Splooce       TPKLIPKVTKTADKHKDVIINQEGEYIKMFMRGRPITMFIPSDVDNYDDIRTELPPEKLKLEWAYGYRGKDCRANVYLLPTGKIVYFIASVVVLFNYEER

Uniprot       TQRHYLGHTDCVKCLAIHPDKIRIATGQIAGVDKDGRPLQPHVRVWDSVTLSTLQIIGLGTFERGVGCLDFSKADSGVHLCIIDDSNEHMLTVWDWQKKA
Splooce       TQRHYLGHTDCVKCLAIHPDKIRIATGQIAGVDKDGRPLQPHVRVWDSVTLSTLQIIGLGTFERGVGCLDFSKADSGVHLCIIDDSNEHMLTVWDWQKKA

Uniprot       KGAEIKTTNEVVLAVEFHPTDANTIITCGKSHIFFWTWSGNSLTRKQGIFGKYEKPKFVQCLAFLGNGDVLTGDSGGVMLIWSKTTVEPTPGKGPKGVYQ
Splooce       KGAEIKTTNEVVLAVEFHPTDANTIITCGKSHIFFWTWSGNSLTRKQGIFGKYEKPKFVQCLAFLGNGDVLTGDSGGVMLIWSKTTVEPTPGKGPKGVYQ

Uniprot       ISKQIKAHDGSVFTLCQMRNGMLLTGGGKDRKIILWDHDLNPEREIEVPDQYGTIRAVAEGKADQFLVGTSRNFILRGTFNDGFQIEVQGHTDELWGLAT
Splooce       ISKQIKAHDGSVFTLCQMRNGMLLTGGGKDRKIILWDHDLNPEREIEVPDQYGTIRAVAEGKADQFLVGTSRNFILRGTFNDGFQIEVQGHTDELWGLAT

Uniprot       HPFKDLLLTCAQDRQVCLWNSMEHRLEWTRLVDEPGHCADFHPSGTVVAIGTHSGRWFVLDAETRDLVSIHTDGNEQLSVMRYSIDGTFLAVGSHDNFIY
Splooce       HPFKDLLLTCAQDRQVCLWNSMEHRLEWTRLVDEPGHCADFHPSGTVVAIGTHSGRWFVLDAETRDLVSIHTDGNEQLSVMRYSIDGTFLAVGSHDNFIY

Uniprot       LYVVSENGRKYSRYGRCTGHSSYITHLDWSPDNKYIMSNSGDYEILYWDIPNGCKLIRNRSDCKDIDWTTYTCVLGFQVFGVWPEGSDGTDINALVRSHN
Splooce       LYVVSENGRKYSRYGRCTGHSSYITHLDWSPDNKYIMSNSGDYEILYWDIPNGCKLIRNRSDCKDIDWTTYTCVLGFQVFGVWPEGSDGTDINALVRSHN

Uniprot       RKVIAVADDFCKVHLFQYPCSKAKAPSHKYSAHSSHVTNVSFTHNDSHLISTGGKDMSIIQWKLVEKLSLPQNETVADTTLTKAPVSSTESVIQSNTPTP
Splooce       RKVIAVADDFCKVHLFQYPCSKAKAPSHKYSAHSSHVTNVSFTHNDSHLISTGGKDMSIIQWKLVEKLSLPQNETVADTTLTKAPVSSTESVIQSNTPTP

Uniprot       PPSQPLNETAEEESRISSSPTLLENSLEQTVEPSEDHSEEESEEGSGDLGEPLYEEPCNEISKEQAKATLLEDQQDPSPSS
Splooce       PPSQPLNETAEEESRISSSPTLLENSLEQTVEPSEDHSEEESEEGSGDLGEPLYEEPCNEISKEQAKATLLEDQQDPSPSS

----------------------------------------------------------------------------------------------------

Q9Y2S2 (Uniprot)	versus
NM_015974#(f-t:13_C5230565100412) (Splooce)

For more details about the Alternative Splicing Event -> Link to Splooce page

Peptides that support the ASE (Splooce-specific):
HLQECVPEDLELK (PEAKS)
HIQECVPEDLELK (MAXQUANT)

Alignment:
Uniprot       MASSAAGCVVIVGSGVIGRSWAMLFASGGFQVKLYDIEQQQIRNALENIRKEMKLLEQAGSLKGSLSVEEQLSLISGCPNIQEAVEGAMHIQECVPEDLE
Splooce       ----------------------------------------------------------------------------------------MHIQECVPEDLE

Uniprot       LKKKIFAQLDSIIDDRVILSSSTSCLMPSKLFAGLVHVKQCIVAHPVNPPYYIPLVELVPHPETAPTTVDRTHALMKKIGQCPMRVQKEVAGFVLNRLQY
Splooce       LKKKIFAQLDSIIDDRVILSSSTSCLMPSKLFAGLVHVKQCIVAHPVNPPYYIPLVELVPHPETAPTTVDRTHALMKKIGQCPMRVQKEVAGFVLNRLQY

Uniprot       AIISEAWRLVEEGIVSPSDLDLVMSEGLGMRYAFIGPLETMHLNAEGMLSYCDRYSEGIKHVLQTFGPIPEFSRATAEKVNQDMCMKVPDDPEHLAARRQ
Splooce       AIISEAWRLVEEGIVSPSDLDLVMSEGLGMRYAFIGPLETMHLNAEGMLSYCDRYSEGIKHVLQTFGPIPEFSRATAEKVNQDMCMKVPDDPEHLAARRQ

Uniprot       WRDECLMRLAKLKSQVQPQ
Splooce       WRDECLMRLAKLKSQVQPQ

----------------------------------------------------------------------------------------------------

O43491 (Uniprot)	versus
NM_001431#(-s-s-s-s-:6_E8965119287959) (Splooce)

For more details about the Alternative Splicing Event -> Link to Splooce page

Peptides that support the ASE (Splooce-specific):
YGVDLHHAK (MAXQUANT)

Alignment:
Uniprot       MTTEVGSVSEVKKDSSQLGTDATKEKPKEVAENQQNQSSDPEEEKGSQPPPAAESQSSLRRQKREKETSESRGISRFIPPWLKKQKSYTLVVAKDGGDKK
Splooce       ----------------------------------------------------------------------------------------------------

Uniprot       EPTQAVVEEQVLDKEEPLPEEQRQAKGDAEEMAQKKQEIKVEVKEEKPSVSKEEKPSVSKVEMQPTELVSKEREEKVKETQEDKLEGGAAKRETKEVQTN
Splooce       ----------------------------------------------------------------------------------------------------

Uniprot       ELKAEKASQKVTKKTKTVQCKVTLLDGTEYSCDLEKHAKGQVLFDKVCEHLNLLEKDYFGLLFQESPEQKNWLDPAKEIKRQLRNLPWLFTFNVKFYPPD
Splooce       ----------------------------------------------------------------------------------------------------

Uniprot       PSQLTEDITRYFLCLQLRQDIASGRLPCSFVTHALLGSYTLQAELGDYDPEEHGSIDLSEFQFAPTQTKELEEKVAELHKTHRGLSPAQADSQFLENAKR
Splooce       ----------------------------------------------------------------------------------------------------

Uniprot       LSMYGVDLHHAKDSEGVDIKLGVCANGLLIYKDRLRINRFAWPKILKISYKRSNFYIKVRPAELEQFESTIGFKLPNHRAAKRLWKVCVEHHTFYRLVSP
Splooce       --MYGVDLHHAKDSEGVDIKLGVCANGLLIYKDRLRINRFAWPKILKISYKRSNFYIKVRPAELEQFESTIGFKLPNHRAAKRLWKVCVEHHTFYRLVSP

Uniprot       EQPPKAKFLTLGSKFRYSGRTQAQTRQASTLIDRPAPHFERTSSKRVSRSLDGAPIGVMDQSLMKDFPGAAGEISAYGPGLVSIAVVQDGDGRREVRSPT
Splooce       EQPPKAKFLTLGSKFRYSGRTQAQTRQASTLIDRPAPHFERTSSKRVSRSLDGAPIGVMDQSLMKDFPGAAGEISAYGPGLVSIAVVQDGDGRREVRSPT

Uniprot       KAPHLQLIEGKKNSLRVEGDNIYVRHSNLMLEELDKAQEDILKHQASISELKRNFMESTPEPRPNEWEKRRITPLSLQTQGSSHETLNIVEEKKRAEVGK
Splooce       KAPHLQLIEGKKNSLRVEGDNIYVRHSNLMLEELDKAQEDILKHQASISELKRNFMESTPEPRPNEWEKRRITPLSLQTQGSSHETLNIVEEKKRAEVGK

Uniprot       DERVITEEMNGKEISPGSGPGEIRKVEPVTQKDSTSLSSESSSSSSESEEEDVGEYRPHHRVTEGTIREEQEYEEEVEEEPRPAAKVVEREEAVPEASPV
Splooce       DERVITEEMNGKEISPGSGPGEIRKVEPVTQKDSTSLSSESSSSSSESEEEDVGEYRPHHRVTEGTIREEQEYEEEVEEEPRPAAKVVEREEAVPEASPV

Uniprot       TQAGASVITVETVIQENVGAQKIPGEKSVHEGALKQDMGEEAEEEPQKVNGEVSHVDIDVLPQIICCSEPPVVKTEMVTISDASQRTEISTKEVPIVQTE
Splooce       TQAGASVITVETVIQENVGAQKIPGEKSVHEGALKQDMGEEAEEEPQKVNGEVSHVDIDVLPQIICCSEPPVVKTEMVTISDASQRTEISTKEVPIVQTE

Uniprot       TKTITYESPQIDGGAGGDSGTLLTAQTITSESVSTTTTTHITKTVKGGISETRIEKRIVITGDGDIDHDQALAQAIREAREQHPDMSVTRVVVHKETELA
Splooce       TKTITYESPQIDGGAGGDSGTLLTAQTITSESVSTTTTTHITKTVKGGISETRIEKRIVITGDGDIDHDQALAQAIREAREQHPDMSVTRVVVHKETELA

Uniprot       EEGED
Splooce       EEGED

----------------------------------------------------------------------------------------------------

F8WB34 (Uniprot)	versus
NM_005971#(f-:19_F9034024990777) (Splooce)

For more details about the Alternative Splicing Event -> Link to Splooce page

Peptides that support the ASE (Splooce-specific):
ALEALPFKDPR (MAXQUANT)

Alignment:
Uniprot       ------------------------------------------------------MQKVTLGLLVFLAGFPVLDANDLEDKNSPFYYDWHSLQVGGLICAG
Splooce       MALEALPFKDPRTFPIQGLLRAWQLRINAGLRLAARFLPEPLLSLVNHTGQRSDMQKVTLGLLVFLAGFPVLDANDLEDKNSPFYYDWHSLQVGGLICAG

Uniprot       VLCAMGIIIVMSAKCKCKFGQKSGHHPGETPPLITPGSAQS
Splooce       VLCAMGIIIVMSAKCKCKFGQKSGHHPGETPPLITPGSAQS

----------------------------------------------------------------------------------------------------

P49792 (Uniprot)	versus
NM_006267#(-s-:2_R425612852379) (Splooce)

For more details about the Alternative Splicing Event -> Link to Splooce page

Peptides that support the ASE (Splooce-specific):
MLLNLSR (MAXQUANT + PEAKS)

Alignment:
Uniprot       MRRSKADVERYIASVQGSTPSPRQKSMKGFYFAKLYYEAKEYDLAKKYICTYINVQERDPKAHRFLGLLYELEENTDKAVECYRRSVELNPTQKDLVLKI
Splooce       ----------------------------------------------------------------------------------------------------

Uniprot       AELLCKNDVTDGRAKYWLERAAKLFPGSPAIYKLKEQLLDCEGEDGWNKLFDLIQSELYVRPDDVHVNIRLVEVYRSTKRLKDAVAHCHEAERNIALRSS
Splooce       ----------------------------------------------------------------------------------------------------

Uniprot       LEWNSCVVQTLKEYLESLQCLESDKSDWRATNTDLLLAYANLMLLTLSTRDVQESRELLQSFDSALQSVKSLGGNDELSATFLEMKGHFYMHAGSLLLKM
Splooce       ----------------------------------------------------------------------------------------------------

Uniprot       GQHSSNVQWRALSELAALCYLIAFQVPRPKIKLIKGEAGQNLLEMMACDRLSQSGHMLLNLSRGKQDFLKEIVETFANKSGQSALYDALFSSQSPKDTSF
Splooce       --------------------------------------------------------MLLNLSRGKQDFLKEIVETFANKSGQSALYDALFSSQSPKDTSF

Uniprot       LGSDDIGNIDVREPELEDLTRYDVGAIRAHNGSLQHLTWLGLQWNSLPALPGIRKWLKQLFHHLPHETSRLETNAPESICILDLEVFLLGVVYTSHLQLK
Splooce       LGSDDIGNIDVREPELEDLTRYDVGAIRAHNGSLQHLTWLGLQWNSLPALPGIRKWLKQLFHHLPHETSRLETNAPESICILDLEVFLLGVVYTSHLQLK

Uniprot       EKCNSHHSSYQPLCLPLPVCKQLCTERQKSWWDAVCTLIHRKAVPGNVAKLRLLVQHEINTLRAQEKHGLQPALLVHWAECLQKTGSGLNSFYDQREYIG
Splooce       EKCNSHHSSYQPLCLPLPVCKQLCTERQKSWWDAVCTLIHRKAVPGNVAKLRLLVQHEINTLRAQEKHGLQPALLVHWAECLQKTGSGLNSFYDQREYIG

Uniprot       RSVHYWKKVLPLLKIIKKKNSIPEPIDPLFKHFHSVDIQASEIVEYEEDAHITFAILDAVNGNIEDAVTAFESIKSVVSYWNLALIFHRKAEDIENDALS
Splooce       RSVHYWKKVLPLLKIIKKKNSIPEPIDPLFKHFHSVDIQASEIVEYEEDAHITFAILDAVNGNIEDAVTAFESIKSVVSYWNLALIFHRKAEDIENDALS

Uniprot       PEEQEECKNYLRKTRDYLIKIIDDSDSNLSVVKKLPVPLESVKEMLNSVMQELEDYSEGGPLYKNGSLRNADSEIKHSTPSPTRYSLSPSKSYKYSPKTP
Splooce       PEEQEECKNYLRKTRDYLIKIIDDSDSNLSVVKKLPVPLESVKEMLNSVMQELEDYSEGGPLYKNGSLRNADSEIKHSTPSPTRYSLSPSKSYKYSPKTP

Uniprot       PRWAEDQNSLLKMICQQVEAIKKEMQELKLNSSNSASPHRWPTENYGPDSVPDGYQGSQTFHGAPLTVATTGPSVYYSQSPAYNSQYLLRPAANVTPTKG
Splooce       PRWAEDQNSLLKMICQQVEAIKKEMQELKLNSSNSASPHRWPTENYGPDSVPDGYQGSQTFHGAPLTVATTGPSVYYSQSPAYNSQYLLRPAANVTPTKG

Uniprot       PVYGMNRLPPQQHIYAYPQQMHTPPVQSSSACMFSQEMYGPPALRFESPATGILSPRGDDYFNYNVQQTSTNPPLPEPGYFTKPPIAAHASRSAESKTIE
Splooce       PVYGMNRLPPQQHIYAYPQQMHTPPVQSSSACMFSQEMYGPPALRFESPATGILSPRGDDYFNYNVQQTSTNPPLPEPGYFTKPPIAAHASRSAESKTIE

Uniprot       FGKTNFVQPMPGEGLRPSLPTQAHTTQPTPFKFNSNFKSNDGDFTFSSPQVVTQPPPAAYSNSESLLGLLTSDKPLQGDGYSGAKPIPGGQTIGPRNTFN
Splooce       FGKTNFVQPMPGEGLRPSLPTQAHTTQPTPFKFNSNFKSNDGDFTFSSPQVVTQPPPAAYSNSESLLGLLTSDKPLQGDGYSGAKPIPGGQTIGPRNTFN

Uniprot       FGSKNVSGISFTENMGSSQQKNSGFRRSDDMFTFHGPGKSVFGTPTLETANKNHETDGGSAHGDDDDDGPHFEPVVPLPDKIEVKTGEEDEEEFFCNRAK
Splooce       FGSKNVSGISFTENMGSSQQKNSGFRRSDDMFTFHGPGKSVFGTPTLETANKNHETDGGSAHGDDDDDGPHFEPVVPLPDKIEVKTGEEDEEEFFCNRAK

Uniprot       LFRFDVESKEWKERGIGNVKILRHKTSGKIRLLMRREQVLKICANHYISPDMKLTPNAGSDRSFVWHALDYADELPKPEQLAIRFKTPEEAALFKCKFEE
Splooce       LFRFDVESKEWKERGIGNVKILRHKTSGKIRLLMRREQVLKICANHYISPDMKLTPNAGSDRSFVWHALDYADELPKPEQLAIRFKTPEEAALFKCKFEE

Uniprot       AQSILKAPGTNVAMASNQAVRIVKEPTSHDNKDICKSDAGNLNFEFQVAKKEGSWWHCNSCSLKNASTAKKCVSCQNLNPSNKELVGPPLAETVFTPKTS
Splooce       AQSILKAPGTNVAMASNQAVRIVKEPTSHDNKDICKSDAGNLNFEFQVAKKEGSWWHCNSCSLKNASTAKKCVSCQNLNPSNKELVGPPLAETVFTPKTS

Uniprot       PENVQDRFALVTPKKEGHWDCSICLVRNEPTVSRCIACQNTKSANKSGSSFVHQASFKFGQGDLPKPINSDFRSVFSTKEGQWDCSACLVQNEGSSTKCA
Splooce       PENVQDRFALVTPKKEGHWDCSICLVRNEPTVSRCIACQNTKSANKSGSSFVHQASFKFGQGDLPKPINSDFRSVFSTKEGQWDCSACLVQNEGSSTKCA

Uniprot       ACQNPRKQSLPATSIPTPASFKFGTSETSKTLKSGFEDMFAKKEGQWDCSSCLVRNEANATRCVACQNPDKPSPSTSVPAPASFKFGTSETSKAPKSGFE
Splooce       ACQNPRKQSLPATSIPTPASFKFGTSETSKTLKSGFEDMFAKKEGQWDCSSCLVRNEANATRCVACQNPDKPSPSTSVPAPASFKFGTSETSKAPKSGFE

Uniprot       GMFTKKEGQWDCSVCLVRNEASATKCIACQNPGKQNQTTSAVSTPASSETSKAPKSGFEGMFTKKEGQWDCSVCLVRNEASATKCIACQNPGKQNQTTSA
Splooce       GMFTKKEGQWDCSVCLVRNEASATKCIACQNPGKQNQTTSAVSTPASSETSKAPKSGFEGMFTKKEGQWDCSVCLVRNEASATKCIACQNPGKQNQTTSA

Uniprot       VSTPASSETSKAPKSGFEGMFTKKEGQWDCSVCLVRNEASATKCIACQCPSKQNQTTAISTPASSEISKAPKSGFEGMFIRKGQWDCSVCCVQNESSSLK
Splooce       VSTPASSETSKAPKSGFEGMFTKKEGQWDCSVCLVRNEASATKCIACQCPSKQNQTTAISTPASSEISKAPKSGFEGMFIRKGQWDCSVCCVQNESSSLK

Uniprot       CVACDASKPTHKPIAEAPSAFTLGSEMKLHDSSGSQVGTGFKSNFSEKASKFGNTEQGFKFGHVDQENSPSFMFQGSSNTEFKSTKEGFSIPVSADGFKF
Splooce       CVACDASKPTHKPIAEAPSAFTLGSEMKLHDSSGSQVGTGFKSNFSEKASKFGNTEQGFKFGHVDQENSPSFMFQGSSNTEFKSTKEGFSIPVSADGFKF

Uniprot       GISEPGNQEKKSEKPLENGTGFQAQDISGQKNGRGVIFGQTSSTFTFADLAKSTSGEGFQFGKKDPNFKGFSGAGEKLFSSQYGKMANKANTSGDFEKDD
Splooce       GISEPGNQEKKSEKPLENGTGFQAQDISGQKNGRGVIFGQTSSTFTFADLAKSTSGEGFQFGKKDPNFKGFSGAGEKLFSSQYGKMANKANTSGDFEKDD

Uniprot       DAYKTEDSDDIHFEPVVQMPEKVELVTGEEDEKVLYSQRVKLFRFDAEVSQWKERGLGNLKILKNEVNGKLRMLMRREQVLKVCANHWITTTMNLKPLSG
Splooce       DAYKTEDSDDIHFEPVVQMPEKVELVTGEEDEKVLYSQRVKLFRFDAEVSQWKERGLGNLKILKNEVNGKLRMLMRREQVLKVCANHWITTTMNLKPLSG

Uniprot       SDRAWMWLASDFSDGDAKLEQLAAKFKTPELAEEFKQKFEECQRLLLDIPLQTPHKLVDTGRAAKLIQRAEEMKSGLKDFKTFLTNDQTKVTEEENKGSG
Splooce       SDRAWMWLASDFSDGDAKLEQLAAKFKTPELAEEFKQKFEECQRLLLDIPLQTPHKLVDTGRAAKLIQRAEEMKSGLKDFKTFLTNDQTKVTEEENKGSG

Uniprot       TGAAGASDTTIKPNPENTGPTLEWDNYDLREDALDDSVSSSSVHASPLASSPVRKNLFRFGESTTGFNFSFKSALSPSKSPAKLNQSGTSVGTDEESDVT
Splooce       TGAAGASDTTIKPNPENTGPTLEWDNYDLREDALDDSVSSSSVHASPLASSPVRKNLFRFGESTTGFNFSFKSALSPSKSPAKLNQSGTSVGTDEESDVT

Uniprot       QEEERDGQYFEPVVPLPDLVEVSSGEENEQVVFSHRAKLYRYDKDVGQWKERGIGDIKILQNYDNKQVRIVMRRDQVLKLCANHRITPDMTLQNMKGTER
Splooce       QEEERDGQYFEPVVPLPDLVEVSSGEENEQVVFSHRAKLYRYDKDVGQWKERGIGDIKILQNYDNKQVRIVMRRDQVLKLCANHRITPDMTLQNMKGTER

Uniprot       VWLWTACDFADGERKVEHLAVRFKLQDVADSFKKIFDEAKTAQEKDSLITPHVSRSSTPRESPCGKIAVAVLEETTRERTDVIQGDDVADATSEVEVSST
Splooce       VWLWTACDFADGERKVEHLAVRFKLQDVADSFKKIFDEAKTAQEKDSLITPHVSRSSTPRESPCGKIAVAVLEETTRERTDVIQGDDVADATSEVEVSST

Uniprot       SETTPKAVVSPPKFVFGSESVKSIFSSEKSKPFAFGNSSATGSLFGFSFNAPLKSNNSETSSVAQSGSESKVEPKKCELSKNSDIEQSSDSKVKNLFASF
Splooce       SETTPKAVVSPPKFVFGSESVKSIFSSEKSKPFAFGNSSATGSLFGFSFNAPLKSNNSETSSVAQSGSESKVEPKKCELSKNSDIEQSSDSKVKNLFASF

Uniprot       PTEESSINYTFKTPEKAKEKKKPEDSPSDDDVLIVYELTPTAEQKALATKLKLPPTFFCYKNRPDYVSEEEEDDEDFETAVKKLNGKLYLDGSEKCRPLE
Splooce       PTEESSINYTFKTPEKAKEKKKPEDSPSDDDVLIVYELTPTAEQKALATKLKLPPTFFCYKNRPDYVSEEEEDDEDFETAVKKLNGKLYLDGSEKCRPLE

Uniprot       ENTADNEKECIIVWEKKPTVEEKAKADTLKLPPTFFCGVCSDTDEDNGNGEDFQSELQKVQEAQKSQTEEITSTTDSVYTGGTEVMVPSFCKSEEPDSIT
Splooce       ENTADNEKECIIVWEKKPTVEEKAKADTLKLPPTFFCGVCSDTDEDNGNGEDFQSELQKVQEAQKSQTEEITSTTDSVYTGGTEVMVPSFCKSEEPDSIT

Uniprot       KSISSPSVSSETMDKPVDLSTRKEIDTDSTSQGESKIVSFGFGSSTGLSFADLASSNSGDFAFGSKDKNFQWANTGAAVFGTQSVGTQSAGKVGEDEDGS
Splooce       KSISSPSVSSETMDKPVDLSTRKEIDTDSTSQGESKIVSFGFGSSTGLSFADLASSNSGDFAFGSKDKNFQWANTGAAVFGTQSVGTQSAGKVGEDEDGS

Uniprot       DEEVVHNEDIHFEPIVSLPEVEVKSGEEDEEILFKERAKLYRWDRDVSQWKERGVGDIKILWHTMKNYYRILMRRDQVFKVCANHVITKTMELKPLNVSN
Splooce       DEEVVHNEDIHFEPIVSLPEVEVKSGEEDEEILFKERAKLYRWDRDVSQWKERGVGDIKILWHTMKNYYRILMRRDQVFKVCANHVITKTMELKPLNVSN

Uniprot       NALVWTASDYADGEAKVEQLAVRFKTKEVADCFKKTFEECQQNLMKLQKGHVSLAAELSKETNPVVFFDVCADGEPLGRITMELFSNIVPRTAENFRALC
Splooce       NALVWTASDYADGEAKVEQLAVRFKTKEVADCFKKTFEECQQNLMKLQKGHVSLAAELSKETNPVVFFDVCADGEPLGRITMELFSNIVPRTAENFRALC

Uniprot       TGEKGFGFKNSIFHRVIPDFVCQGGDITKHDGTGGQSIYGDKFEDENFDVKHTGPGLLSMANQGQNTNNSQFVITLKKAEHLDFKHVVFGFVKDGMDTVK
Splooce       TGEKGFGFKNSIFHRVIPDFVCQGGDITKHDGTGGQSIYGDKFEDENFDVKHTGPGLLSMANQGQNTNNSQFVITLKKAEHLDFKHVVFGFVKDGMDTVK

Uniprot       KIESFGSPKGSVCRRITITECGQI
Splooce       KIESFGSPKGSVCRRITITECGQI

----------------------------------------------------------------------------------------------------

P05362 (Uniprot)	versus
NM_000201#(r:19_I1521608983962) (Splooce)

For more details about the Alternative Splicing Event -> Link to Splooce page

Peptides that support the ASE (Splooce-specific):
CQAWGNPLPELK (MAXQUANT + PEAKS)

Alignment:
Uniprot       MAPSSPRPALPALLVLLGALFPGPGNAQTSVSPSKVILPRGGSVLVTCSTSCDQPKLLGIETPLPKKELLLPGNNRKVYELSNVQEDSQPMCYSNCPDGQ
Splooce       ----------------------------------------------------------------------------------------------------

Uniprot       STAKTFLTVYWTPERVELAPLPSWQPVGKNLTLRCQVEGGAPRANLTVVLLRGEKELKREPAVGEPAEVTTTVLVRRDHHGANFSCRTELDLRPQGLELF
Splooce       ----------------------------------------------------------------------------------------------------

Uniprot       ENTSAPYQLQTFVLPATPPQLVSPRVLEVDTQGTVVCSLDGLFPVSEAQVHLALGDQRLNPTVTYGNDSFSAKASVSVTAEDEGTQRLTCAVILGNQSQE
Splooce       ----------------------------------------------------------------------------------------------------

Uniprot       TLQTVTIYSFPAPNVILTKPEVSEGTEVTVKCEAHPRAKVTLNGVPAQPLGPRAQLLLKATPEDNGRSFSCSATLEVAGQLIHKNQTRELRVLYGPRLDE
Splooce       ----------------------------------------------------------------------------------------------------

Uniprot       RDCPGNWTWPENSQQTPMCQAWGNPLPELKCLKDGTFPLPIGESVTVTRDLEGTYLCRARSTQGEVTRKVTVNVLSPRYEIVIITVVAAAVIMGTAGLST
Splooce       -----------------MCQAWGNPLPELKCLKDGTFPLPIGESVTVTRDLEGTYLCRARSTQGEVTRKVTVNVLSPRYEIVIITVVAAAVIMGTAGLST

Uniprot       YLYNRQRKIKKYRLQQAQKGTPMKPNTQATPP
Splooce       YLYNRQRKIKKYRLQQAQKGTPMKPNTQATPP

----------------------------------------------------------------------------------------------------

Q9NY33 (Uniprot)	versus
NM_005700#(-s-s-s-s-:11_D9045559562094) (Splooce)

For more details about the Alternative Splicing Event -> Link to Splooce page

Peptides that support the ASE (Splooce-specific):
MLAQYIESFTQGSIEAHK (MAXQUANT)
LAQYIESFTQGSIEAHK (MAXQUANT)

Alignment:
Uniprot       MGEPEAGSEFANGAAAAGPMADTQYILPNDIGVSSLDCREAFRLLSPTERLYAYHLSRAAWYGGLAVLLQTSPEAPYIYALLSRLFRAQDPDQLRQHALA
Splooce       ----------------------------------------------------------------------------------------------------

Uniprot       EGLTEEEYQAFLVYAAGVYSNMGNYKSFGDTKFVPNLPKEKLERVILGSEAAQQHPEEVRGLWQTCGELMFSLEPRLRHLGLGKEGITTYFSGNCTMEDA
Splooce       ----------------------------------------------------------------------------------------------------

Uniprot       KLAQDFLDSQNLSAYNTRLFKEVDGEGKPYYEVRLASVLGSEPSLDSEVTSKLKSYEFRGSPFQVTRGDYAPILQKVVEQLEKAKAYAANSHQGQMLAQY
Splooce       -----------------------------------------------------------------------------------------------MLAQY

Uniprot       IESFTQGSIEAHKRGSRFWIQDKGPIVESYIGFIESYRDPFGSRGEFEGFVAVVNKAMSAKFERLVASAEQLLKELPWPPTFEKDKFLTPDFTSLDVLTF
Splooce       IESFTQGSIEAHKRGSRFWIQDKGPIVESYIGFIESYRDPFGSRGEFEGFVAVVNKAMSAKFERLVASAEQLLKELPWPPTFEKDKFLTPDFTSLDVLTF

Uniprot       AGSGIPAGINIPNYDDLRQTEGFKNVSLGNVLAVAYATQREKLTFLEEDDKDLYILWKGPSFDVQVGLHELLGHGSGKLFVQDEKGAFNFDQETVINPET
Splooce       AGSGIPAGINIPNYDDLRQTEGFKNVSLGNVLAVAYATQREKLTFLEEDDKDLYILWKGPSFDVQVGLHELLGHGSGKLFVQDEKGAFNFDQETVINPET

Uniprot       GEQIQSWYRSGETWDSKFSTIASSYEECRAESVGLYLCLHPQVLEIFGFEGADAEDVIYVNWLNMVRAGLLALEFYTPEAFNWRQAHMQARFVILRVLLE
Splooce       GEQIQSWYRSGETWDSKFSTIASSYEECRAESVGLYLCLHPQVLEIFGFEGADAEDVIYVNWLNMVRAGLLALEFYTPEAFNWRQAHMQARFVILRVLLE

Uniprot       AGEGLVTITPTTGSDGRPDARVRLDRSKIRSVGKPALERFLRRLQVLKSTGDVAGGRALYEGYATVTDAPPECFLTLRDTVLLRKESRKLIVQPNTRLEG
Splooce       AGEGLVTITPTTGSDGRPDARVRLDRSKIRSVGKPALERFLRRLQVLKSTGDVAGGRALYEGYATVTDAPPECFLTLRDTVLLRKESRKLIVQPNTRLEG

Uniprot       SDVQLLEYEASAAGLIRSFSERFPEDGPELEEILTQLATADARFWKGPSEAPSGQA
Splooce       SDVQLLEYEASAAGLIRSFSERFPEDGPELEEILTQLATADARFWKGPSEAPSGQA

----------------------------------------------------------------------------------------------------

P62917 (Uniprot)	versus
NM_033301#(f-t:8_R1994695530813) (Splooce)

For more details about the Alternative Splicing Event -> Link to Splooce page

Peptides that support the ASE (Splooce-specific):
PEGTIVCCLEEK (MAXQUANT)
PEGTIVCCLEEKPGDR (MAXQUANT)
PEGTIVCCLEEKPGDRGK (MAXQUANT)

Alignment:
Uniprot       MGRVIRGQRKGAGSVFRAHVKHRKGAARLRAVDFAERHGYIKGIVKDIIHDPGRGAPLAKVVFRDPYRFKKRTELFIAAEGIHTGQFVYCGKKAQLNIGN
Splooce       ----------------------------------------------------------------------------------------------------

Uniprot       VLPVGTMPEGTIVCCLEEKPGDRGKLARASGNYATVISHNPETKKTRVKLPSGSKKVISSANRAVVGVVAGGGRIDKPILKAGRAYHKYKAKRNCWPRVR
Splooce       ------MPEGTIVCCLEEKPGDRGKLARASGNYATVISHNPETKKTRVKLPSGSKKVISSANRAVVGVVAGGGRIDKPILKAGRAYHKYKAKRNCWPRVR

Uniprot       GVAMNPVEHPFGGGNHQHIGKPSTIRRDAPAGRKVGLIAARRTGRLRGTKTVQEKEN
Splooce       GVAMNPVEHPFGGGNHQHIGKPSTIRRDAPAGRKVGLIAARRTGRLRGTKTVQEKEN

----------------------------------------------------------------------------------------------------

Q8TEX9 (Uniprot)	versus
NM_024658#(-s-s-s-s-s-s-s-:14_I7779196594010) (Splooce)

For more details about the Alternative Splicing Event -> Link to Splooce page

Peptides that support the ASE (Splooce-specific):
MECMLQLLR (MAXQUANT)

Alignment:
Uniprot       MESAGLEQLLRELLLPDTERIRRATEQLQIVLRAPAALPALCDLLASAADPQIRQFAAVLTRRRLNTRWRRLAAEQRESLKSLILTALQRETEHCVSLSL
Splooce       ----------------------------------------------------------------------------------------------------

Uniprot       AQLSATIFRKEGLEAWPQLLQLLQHSTHSPHSPEREMGLLLLSVVVTSRPEAFQPHHRELLRLLNETLGEVGSPGLLFYSLRTLTTMAPYLSTEDVPLAR
Splooce       ----------------------------------------------------------------------------------------------------

Uniprot       MLVPKLIMAMQTLIPIDEAKACEALEALDELLESEVPVITPYLSEVLTFCLEVARNVALGNAIRIRILCCLTFLVKVKSKALLKNRLLPPLLHTLFPIVA
Splooce       ----------------------------------------------------------------------------------------------------

Uniprot       AEPPPGQLDPEDQDSEEEELEIELMGETPKHFAVQVVDMLALHLPPEKLCPQLMPMLEEALRSESPYQRKAGLLVLAVLSDGAGDHIRQRLLPPLLQIVC
Splooce       ----------------------------------------------------------------------------------------------------

Uniprot       KGLEDPSQVVRNAALFALGQFSENLQPHISSYSREVMPLLLAYLKSVPLGHTHHLAKACYALENFVENLGPKVQPYLPELMECMLQLLRNPSSPRAKELA
Splooce       --------------------------------------------------------------------------------MECMLQLLRNPSSPRAKELA

Uniprot       VSALGAIATAAQASLLPYFPAIMEHLREFLLTGREDLQPVQIQSLETLGVLARAVGEPMRPLAEECCQLGLGLCDQVDDPDLRRCTYSLFAALSGLMGEG
Splooce       VSALGAIATAAQASLLPYFPAIMEHLREFLLTGREDLQPVQIQSLETLGVLARAVGEPMRPLAEECCQLGLGLCDQVDDPDLRRCTYSLFAALSGLMGEG

Uniprot       LAPHLEQITTLMLLSLRSTEGIVPQYDGSSSFLLFDDESDGEEEEELMDEDVEEEDDSEISGYSVENAFFDEKEDTCAAVGEISVNTSVAFLPYMESVFE
Splooce       LAPHLEQITTLMLLSLRSTEGIVPQYDGSSSFLLFDDESDGEEEEELMDEDVEEEDDSEISGYSVENAFFDEKEDTCAAVGEISVNTSVAFLPYMESVFE

Uniprot       EVFKLLECPHLNVRKAAHEALGQFCCALHKACQSCPSEPNTAALQAALARVVPSYMQAVNRERERQVVMAVLEALTGVLRSCGTLTLKPPGRLAELCGVL
Splooce       EVFKLLECPHLNVRKAAHEALGQFCCALHKACQSCPSEPNTAALQAALARVVPSYMQAVNRERERQVVMAVLEALTGVLRSCGTLTLKPPGRLAELCGVL

Uniprot       KAVLQRKTACQDTDEEEEEEDDDQAEYDAMLLEHAGEAIPALAAAAGGDSFAPFFAGFLPLLVCKTKQGCTVAEKSFAVGTLAETIQGLGAASAQFVSRL
Splooce       KAVLQRKTACQDTDEEEEEEDDDQAEYDAMLLEHAGEAIPALAAAAGGDSFAPFFAGFLPLLVCKTKQGCTVAEKSFAVGTLAETIQGLGAASAQFVSRL

Uniprot       LPVLLSTAQEADPEVRSNAIFGMGVLAEHGGHPAQEHFPKLLGLLFPLLARERHDRVRDNICGALARLLMASPTRKPEPQVLAALLHALPLKEDLEEWVT
Splooce       LPVLLSTAQEADPEVRSNAIFGMGVLAEHGGHPAQEHFPKLLGLLFPLLARERHDRVRDNICGALARLLMASPTRKPEPQVLAALLHALPLKEDLEEWVT

Uniprot       IGRLFSFLYQSSPDQVIDVAPELLRICSLILADNKIPPDTKAALLLLLTFLAKQHTDSFQAALGSLPVDKAQELQAVLGLS
Splooce       IGRLFSFLYQSSPDQVIDVAPELLRICSLILADNKIPPDTKAALLLLLTFLAKQHTDSFQAALGSLPVDKAQELQAVLGLS

----------------------------------------------------------------------------------------------------

E7EUT4 (Uniprot)	versus
NM_002046#(f-t:12_G5852279946153) (Splooce)

For more details about the Alternative Splicing Event -> Link to Splooce page

Peptides that support the ASE (Splooce-specific):
MTTVHALTATQK (PEAKS)
MTTVHAITATQK (MAXQUANT)
TTVHAITATQK (MAXQUANT)
TTVHALTATQK (PEAKS)

Alignment:
Uniprot       MGKVKVGVNGFGRIGRLVTRAAFNSGKVDIVAINDPFIDLNYMVYMFQYDSTHGKFHGTVKAENGKLVINGNPITIFQERDPSKIKWGDAGAEYVVESTG
Splooce       ----------------------------------------------------------------------------------------------------

Uniprot       VFTTMEKAGAHLQGGAKRVIISAPSADAPMFVMGVNHEKYDNSLKIISNASCTTNCLAPLAKVIHDNFGIVEGLMTTVHAITATQKTVDGPSGKLWRDGR
Splooce       --------------------------------------------------------------------------MTTVHAITATQKTVDGPSGKLWRDGR

Uniprot       GALQNIIPASTGAAKAVGKVIPELNGKLTGMAFRVPTANVSVVDLTCRLEKPAKYDDIKKVVKQASEGPLKGILGYTEHQVVSSDFNSDTHSSTFDAGAG
Splooce       GALQNIIPASTGAAKAVGKVIPELNGKLTGMAFRVPTANVSVVDLTCRLEKPAKYDDIKKVVKQASEGPLKGILGYTEHQVVSSDFNSDTHSSTFDAGAG

Uniprot       IALNDHFVKLISWYDNEFGYSNRVVDLMAHMASKE
Splooce       IALNDHFVKLISWYDNEFGYSNRVVDLMAHMASKE

----------------------------------------------------------------------------------------------------

Q6IBW4 (Uniprot)	versus
NM_152299#(f-:22_N5984403540897) (Splooce)

For more details about the Alternative Splicing Event -> Link to Splooce page

Peptides that support the ASE (Splooce-specific):
ALVAPDEMEK (MAXQUANT + PEAKS)

Alignment:
Uniprot       MEDVEARFAHLLQPIRDLTKNWEVDVAAQLGEYLEELDQICISFDEGKTTMNFIEAALLIQGSACVYSKKVEYLYSLVYQALDFISGKRRAKQLSSVQED
Splooce       ----------------------------------------------------------------------------------------------------

Uniprot       RANGVASSGVPQEAENEFLSLDDFPDSRTNVDLKNDQTPSEVLIIPLLPMALVAPDEMEKNNNPLYSRQGEVLASRKDFRMNTCVPHPRGAFMLEPEGMS
Splooce       -------------------------------------------------MALVAPDEMEKNNNPLYSRQGEVLASRKDFRMNTCVPHPRGAFMLEPEGMS

Uniprot       PMEPAGVSPMPGTQKDTGRTEEQPMEVSVCRSPVPALGFSQEPGPSPEGPMPLGGGEDEDAEEAVELPEASAPKAALEPKESRSPQQSAALPRRYMLRER
Splooce       PMEPAGVSPMPGTQKDTGRTEEQPMEVSVCRSPVPALGFSQEPGPSPEGPMPLGGGEDEDAEEAVELPEASAPKAALEPKESRSPQQSAALPRRYMLRER

Uniprot       EGAPEPASCVKETPDPWQSLDPFDSLESKPFKKGRPYSVPPCVEEALGQKRKRKGAAKLQDFHQWYLAAYADHADSRRLRRKGPSFADMEVLYWTHVKEQ
Splooce       EGAPEPASCVKETPDPWQSLDPFDSLESKPFKKGRPYSVPPCVEEALGQKRKRKGAAKLQDFHQWYLAAYADHADSRRLRRKGPSFADMEVLYWTHVKEQ

Uniprot       LETLRKLQRREVAEQWLRPAEEDHLEDSLEDLGAADDFLEPEEYMEPEGADPREAADLDAVPMSLSYEELVRRNVELFIATSQKFVQETELSQRIRDWED
Splooce       LETLRKLQRREVAEQWLRPAEEDHLEDSLEDLGAADDFLEPEEYMEPEGADPREAADLDAVPMSLSYEELVRRNVELFIATSQKFVQETELSQRIRDWED

Uniprot       TVQPLLQEQEQHVPFDIHTYGDQLVSRFPQLNEWCPFAELVAGQPAFEVCRSMLASLQLANDYTVEITQQPGLEMAVDTMSLRLLTHQRAHKRFQTYAAP
Splooce       TVQPLLQEQEQHVPFDIHTYGDQLVSRFPQLNEWCPFAELVAGQPAFEVCRSMLASLQLANDYTVEITQQPGLEMAVDTMSLRLLTHQRAHKRFQTYAAP

Uniprot       SMAQP
Splooce       SMAQP

----------------------------------------------------------------------------------------------------

O43747 (Uniprot)	versus
NM_001030007#(-s-s-s-:16_A3658317500970) (Splooce)

For more details about the Alternative Splicing Event -> Link to Splooce page

Peptides that support the ASE (Splooce-specific):
SGYSPEHDVSGISDPFLQVR (MAXQUANT)

Alignment:
Uniprot       MPAPIRLRELIRTIRTARTQAEEREMIQKECAAIRSSFREEDNTYRCRNVAKLLYMHMLGYPAHFGQLECLKLIASQKFTDKRIGYLGAMLLLDERQDVH
Splooce       ----------------------------------------------------------------------------------------------------

Uniprot       LLMTNCIKNDLNHSTQFVQGLALCTLGCMGSSEMCRDLAGEVEKLLKTSNSYLRKKAALCAVHVIRKVPELMEMFLPATKNLLNEKNHGVLHTSVVLLTE
Splooce       ----------------------------------------------------------------------------------------------------

Uniprot       MCERSPDMLAHFRKLVPQLVRILKNLIMSGYSPEHDVSGISDPFLQVRILRLLRILGRNDDDSSEAMNDILAQVATNTETSKNVGNAILYETVLTIMDIK
Splooce       ---------------------------MSGYSPEHDVSGISDPFLQVRILRLLRILGRNDDDSSEAMNDILAQVATNTETSKNVGNAILYETVLTIMDIK

Uniprot       SESGLRVLAINILGRFLLNNDKNIRYVALTSLLKTVQTDHNAVQRHRSTIVDCLKDLDVSIKRRAMELSFALVNGNNIRGMMKELLYFLDSCEPEFKADC
Splooce       SESGLRVLAINILGRFLLNNDKNIRYVALTSLLKTVQTDHNAVQRHRSTIVDCLKDLDVSIKRRAMELSFALVNGNNIRGMMKELLYFLDSCEPEFKADC

Uniprot       ASGIFLAAEKYAPSKRWHIDTIMRVLTTAGSYVRDDAVPNLIQLITNSVEMHAYTVQRLYKAILGDYSQQPLVQVAAWCIGEYGDLLVSGQCEEEEPIQV
Splooce       ASGIFLAAEKYAPSKRWHIDTIMRVLTTAGSYVRDDAVPNLIQLITNSVEMHAYTVQRLYKAILGDYSQQPLVQVAAWCIGEYGDLLVSGQCEEEEPIQV

Uniprot       TEDEVLDILESVLISNMSTSVTRGYALTAIMKLSTRFTCTVNRIKKVVSIYGSSIDVELQQRAVEYNALFKKYDHMRSALLERMPVMEKVTTNGPTEIVQ
Splooce       TEDEVLDILESVLISNMSTSVTRGYALTAIMKLSTRFTCTVNRIKKVVSIYGSSIDVELQQRAVEYNALFKKYDHMRSALLERMPVMEKVTTNGPTEIVQ

Uniprot       TNGETEPAPLETKPPPSGPQPTSQANDLLDLLGGNDITPVIPTAPTSKPSSAGGELLDLLGDINLTGAPAAAPAPASVPQISQPPFLLDGLSSQPLFNDI
Splooce       TNGETEPAPLETKPPPSGPQPTSQANDLLDLLGGNDITPVIPTAPTSKPSSAGGELLDLLGDINLTGAPAAAPAPASVPQISQPPFLLDGLSSQPLFNDI

Uniprot       AAGIPSITAYSKNGLKIEFTFERSNTNPSVTVITIQASNSTELDMTDFVFQAAVPKTFQLQLLSPSSSIVPAFNTGTITQVIKVLNPQKQQLRMRIKLTY
Splooce       AAGIPSITAYSKNGLKIEFTFERSNTNPSVTVITIQASNSTELDMTDFVFQAAVPKTFQLQLLSPSSSIVPAFNTGTITQVIKVLNPQKQQLRMRIKLTY

Uniprot       NHKGSAMQDLAEVNNFPPQSWQ
Splooce       NHKGSAMQDLAEVNNFPPQSWQ

----------------------------------------------------------------------------------------------------

P53621 (Uniprot)	versus
NM_004371#(-s-:1_C8323990470287) (Splooce)

For more details about the Alternative Splicing Event -> Link to Splooce page

Peptides that support the ASE (Splooce-specific):
MSYNPAENAVLLCTR (MAXQUANT)

Alignment:
Uniprot       MLTKFETKSARVKGLSFHPKRPWILTSLHNGVIQLWDYRMCTLIDKFDEHDGPVRGIDFHKQQPLFVSGGDDYKIKVWNYKLRRCLFTLLGHLDYIRTTF
Splooce       ----------------------------------------------------------------------------------------------------

Uniprot       FHHEYPWILSASDDQTIRVWNWQSRTCVCVLTGHNHYVMCAQFHPTEDLVVSASLDQTVRVWDISGLRKKNLSPGAVESDVRGITGVDLFGTTDAVVKHV
Splooce       ----------------------------------------------------------------------------------------------------

Uniprot       LEGHDRGVNWAAFHPTMPLIVSGADDRQVKIWRMNESKAWEVDTCRGHYNNVSCAVFHPRQELILSNSEDKSIRVWDMSKRTGVQTFRRDHDRFWVLAAH
Splooce       ----------------------------------------------------------------------------------------------------

Uniprot       PNLNLFAAGHDGGMIVFKLERERPAYAVHGNMLHYVKDRFLRQLDFNSSKDVAVMQLRSGSKFPVFNMSYNPAENAVLLCTRASNLENSTYDLYTIPKDA
Splooce       -------------------------------------------------------------------MSYNPAENAVLLCTRASNLENSTYDLYTIPKDA

Uniprot       DSQNPDAPEGKRSSGLTAVWVARNRFAVLDRMHSLLIKNLKNEITKKVQVPNCDEIFYAGTGNLLLRDADSITLFDVQQKRTLASVKISKVKYVIWSADM
Splooce       DSQNPDAPEGKRSSGLTAVWVARNRFAVLDRMHSLLIKNLKNEITKKVQVPNCDEIFYAGTGNLLLRDADSITLFDVQQKRTLASVKISKVKYVIWSADM

Uniprot       SHVALLAKHAIVICNRKLDALCNIHENIRVKSGAWDESGVFIYTTSNHIKYAVTTGDHGIIRTLDLPIYVTRVKGNNVYCLDRECRPRVLTIDPTEFKFK
Splooce       SHVALLAKHAIVICNRKLDALCNIHENIRVKSGAWDESGVFIYTTSNHIKYAVTTGDHGIIRTLDLPIYVTRVKGNNVYCLDRECRPRVLTIDPTEFKFK

Uniprot       LALINRKYDEVLHMVRNAKLVGQSIIAYLQKKGYPEVALHFVKDEKTRFSLALECGNIEIALEAAKALDDKNCWEKLGEVALLQGNHQIVEMCYQRTKNF
Splooce       LALINRKYDEVLHMVRNAKLVGQSIIAYLQKKGYPEVALHFVKDEKTRFSLALECGNIEIALEAAKALDDKNCWEKLGEVALLQGNHQIVEMCYQRTKNF

Uniprot       DKLSFLYLITGNLEKLRKMMKIAEIRKDMSGHYQNALYLGDVSERVRILKNCGQKSLAYLTAATHGLDEEAESLKETFDPEKETIPDIDPNAKLLQPPAP
Splooce       DKLSFLYLITGNLEKLRKMMKIAEIRKDMSGHYQNALYLGDVSERVRILKNCGQKSLAYLTAATHGLDEEAESLKETFDPEKETIPDIDPNAKLLQPPAP

Uniprot       IMPLDTNWPLLTVSKGFFEGTIASKGKGGALAADIDIDTVGTEGWGEDAELQLDEDGFVEATEGLGDDALGKGQEEGGGWDVEEDLELPPELDISPGAAG
Splooce       IMPLDTNWPLLTVSKGFFEGTIASKGKGGALAADIDIDTVGTEGWGEDAELQLDEDGFVEATEGLGDDALGKGQEEGGGWDVEEDLELPPELDISPGAAG

Uniprot       GAEDGFFVPPTKGTSPTQIWCNNSQLPVDHILAGSFETAMRLLHDQVGVIQFGPYKQLFLQTYARGRTTYQALPCLPSMYGYPNRNWKDAGLKNGVPAVG
Splooce       GAEDGFFVPPTKGTSPTQIWCNNSQLPVDHILAGSFETAMRLLHDQVGVIQFGPYKQLFLQTYARGRTTYQALPCLPSMYGYPNRNWKDAGLKNGVPAVG

Uniprot       LKLNDLIQRLQLCYQLTTVGKFEEAVEKFRSILLSVPLLVVDNKQEIAEAQQLITICREYIVGLSVETERKKLPKETLEQQKRICEMAAYFTHSNLQPVH
Splooce       LKLNDLIQRLQLCYQLTTVGKFEEAVEKFRSILLSVPLLVVDNKQEIAEAQQLITICREYIVGLSVETERKKLPKETLEQQKRICEMAAYFTHSNLQPVH

Uniprot       MILVLRTALNLFFKLKNFKTAATFARRLLELGPKPEVAQQTRKILSACEKNPTDAYQLNYDMHNPFDICAASYRPIYRGKPVEKCPLSGACYSPEFKGQI
Splooce       MILVLRTALNLFFKLKNFKTAATFARRLLELGPKPEVAQQTRKILSACEKNPTDAYQLNYDMHNPFDICAASYRPIYRGKPVEKCPLSGACYSPEFKGQI

Uniprot       CRVTTVTEIGKDVIGLRISPLQFR
Splooce       CRVTTVTEIGKDVIGLRISPLQFR

----------------------------------------------------------------------------------------------------

Q9BTD8 (Uniprot)	versus
NM_024321#(-s-s-s-s-s-:19_R5556580412580) (Splooce)

For more details about the Alternative Splicing Event -> Link to Splooce page

Peptides that support the ASE (Splooce-specific):
PLPEPEPLPLPLEVVR (MAXQUANT)

Alignment:
Uniprot       MAGAGPAPGLPGAGGPVVPGPGAGIPGKSGEERLKEMEAEMALFEQEVLGAPVPGIPTAVPAVPTVPTVPTVEAMQVPAAPVIRPIIATNTYQQVQQTLE
Splooce       ----------------------------------------------------------------------------------------------------

Uniprot       ARAAAAATVVPPMVGGPPFVGPVGFGPGDRSHLDSPEAREAMFLRRAAVAPQRAPILRPAFVPHVLQRADSALSSAAAGPRPMALRPPHQALVGPPLPGP
Splooce       ----------------------------------------------------------------------------------------------------

Uniprot       PGPPMMLPPMARAPGPPLGSMAALRPPLEEPAAPRELGLGLGLGLKEKEEAVVAAAAGLEEASAAVAVGAGGAPAGPAVIGPSLPLALAMPLPEPEPLPL
Splooce       -----------------------------------------------------------------------------------------MPLPEPEPLPL

Uniprot       PLEVVRGLLPPLRIPELLSLRPRPRPPRPEPPPGLMALEVPEPLGEDKKKGKPEKLKRCIRTAAGSSWEDPSLLEWDADDFRIFCGDLGNEVNDDILARA
Splooce       PLEVVRGLLPPLRIPELLSLRPRPRPPRPEPPPGLMALEVPEPLGEDKKKGKPEKLKRCIRTAAGSSWEDPSLLEWDADDFRIFCGDLGNEVNDDILARA

Uniprot       FSRFPSFLKAKVIRDKRTGKTKGYGFVSFKDPSDYVRAMREMNGKYVGSRPIKLRKSMWKDRNLDVVRKKQKEKKKLGLR
Splooce       FSRFPSFLKAKVIRDKRTGKTKGYGFVSFKDPSDYVRAMREMNGKYVGSRPIKLRKSMWKDRNLDVVRKKQKEKKKLGLR

----------------------------------------------------------------------------------------------------

P13667 (Uniprot)	versus
NM_004911#(-s-s-s-s-s-s-:7_P6909334756096) (Splooce)

For more details about the Alternative Splicing Event -> Link to Splooce page

Peptides that support the ASE (Splooce-specific):
EPEEFDSDTLR (MAXQUANT)

Alignment:
Uniprot       MRPRKAFLLLLLLGLVQLLAVAGAEGPDEDSSNRENAIEDEEEEEEEDDDEEEDDLEVKEENGVLVLNDANFDNFVADKDTVLLEFYAPWCGHCKQFAPE
Splooce       ----------------------------------------------------------------------------------------------------

Uniprot       YEKIANILKDKDPPIPVAKIDATSASVLASRFDVSGYPTIKILKKGQAVDYEGSRTQEEIVAKVREVSQPDWTPPPEVTLVLTKENFDEVVNDADIILVE
Splooce       ----------------------------------------------------------------------------------------------------

Uniprot       FYAPWCGHCKKLAPEYEKAAKELSKRSPPIPLAKVDATAETDLAKRFDVSGYPTLKIFRKGRPYDYNGPREKYGIVDYMIEQSGPPSKEILTLKQVQEFL
Splooce       ----------------------------------------------------------------------------------------------------

Uniprot       KDGDDVIIIGVFKGESDPAYQQYQDAANNLREDYKFHHTFSTEIAKFLKVSQGQLVVMQPEKFQSKYEPRSHMMDVQGSTQDSAIKDFVLKYALPLVGHR
Splooce       ----------------------------------------------------------------------------------------------------

Uniprot       KVSNDAKRYTRRPLVVVYYSVDFSFDYRAATQFWRSKVLEVAKDFPEYTFAIADEEDYAGEVKDLGLSESGEDVNAAILDESGKKFAMEPEEFDSDTLRE
Splooce       ---------------------------------------------------------------------------------------MEPEEFDSDTLRE

Uniprot       FVTAFKKGKLKPVIKSQPVPKNNKGPVKVVVGKTFDSIVMDPKKDVLIEFYAPWCGHCKQLEPVYNSLAKKYKGQKGLVIAKMDATANDVPSDRYKVEGF
Splooce       FVTAFKKGKLKPVIKSQPVPKNNKGPVKVVVGKTFDSIVMDPKKDVLIEFYAPWCGHCKQLEPVYNSLAKKYKGQKGLVIAKMDATANDVPSDRYKVEGF

Uniprot       PTIYFAPSGDKKNPVKFEGGDRDLEHLSKFIEEHATKLSRTKEEL
Splooce       PTIYFAPSGDKKNPVKFEGGDRDLEHLSKFIEEHATKLSRTKEEL

----------------------------------------------------------------------------------------------------

P22392 (Uniprot)	versus
NM_002512#(-s-s-s-:17_N6953512907635) (Splooce)

For more details about the Alternative Splicing Event -> Link to Splooce page

Peptides that support the ASE (Splooce-specific):
MTGSMNK (MAXQUANT)

Alignment:
Uniprot       MANLERTFIAIKPDGVQRGLVGEIIKRFEQKGFRLVAMKFLRASEEHLKQHYIDLKDRPFFPGLVKYMNSGPVVAMVWEGLNVVKTGRVMLGETNPADSK
Splooce       -------------------MTGSMNKRWTQQ--QSPSAR--RGVSLDTALHSIDLEAT----GLIILL--------------------------------

Uniprot       PGTIRGDFCIQVGRNIIHGSDSVKSAEKEISLWFKPEELVDYKSCAHDWVYE
Splooce       ----------------------------------------------------

----------------------------------------------------------------------------------------------------

P50226 (Uniprot)	versus
NM_001054#(-s-:16_S5971114945857) (Splooce)

For more details about the Alternative Splicing Event -> Link to Splooce page

Peptides that support the ASE (Splooce-specific):
LYQGGDLEK (PEAKS)

Alignment:
Uniprot       MELIQDISRPPLEYVKGVPLIKYFAEALGPLQSFQARPDDLLISTYPKSGTTWVSQILDMIYQGGDLEKCHRAPIFMRVPFLEFKVPGIPSGMETLKNTP
Splooce       -----------------------------------------------------------MIYQGGDLEKCHRAPIFMRVPFLEFKVPGIPSGMETLKNTP

Uniprot       APRLLKTHLPLALLPQTLLDQKVKVVYVARNAKDVAVSYYHFYHMAKVYPHPGTWESFLEKFMAGEVSYGSWYQHVQEWWELSRTHPVLYLFYEDMKENP
Splooce       APRLLKTHLPLALLPQTLLDQKVKVVYVARNAKDVAVSYYHFYHMAKVYPHPGTWESFLEKFMAGEVSYGSWYQHVQEWWELSRTHPVLYLFYEDMKENP

Uniprot       KREIQKILEFVGRSLPEETVDLMVEHTSFKEMKKNPMTNYTTVRREFMDHSISPFMRKGMAGDWKTTFTVAQNERFDADYAKKMAGCSLSFRSEL
Splooce       KREIQKILEFVGRSLPEETVDLMVEHTSFKEMKKNPMTNYTTVRREFMDHSISPFMRKGMAGDWKTTFTVAQNERFDADYAKKMAGCSLSFRSEL

----------------------------------------------------------------------------------------------------

Q9H0D6 (Uniprot)	versus
NM_012255#(-s-s-s-s-s-s-:20_X555863001685) (Splooce)

For more details about the Alternative Splicing Event -> Link to Splooce page

Peptides that support the ASE (Splooce-specific):
LGLATHEPNFTIIR (MAXQUANT)

Alignment:
Uniprot       MGVPAFFRWLSRKYPSIIVNCVEEKPKECNGVKIPVDASKPNPNDVEFDNLYLDMNGIIHPCTHPEDKPAPKNEDEMMVAIFEYIDRLFSIVRPRRLLYM
Splooce       ----------------------------------------------------------------------------------------------------

Uniprot       AIDGVAPRAKMNQQRSRRFRASKEGMEAAVEKQRVREEILAKGGFLPPEEIKERFDSNCITPGTEFMDNLAKCLRYYIADRLNNDPGWKNLTVILSDASA
Splooce       ----------------------------------------------------------------------------------------------------

Uniprot       PGEGEHKIMDYIRRQRAQPNHDPNTHHCLCGADADLIMLGLATHEPNFTIIREEFKPNKPKPCGLCNQFGHEVKDCEGLPREKKGKHDELADSLPCAEGE
Splooce       -------------------------------------MLGLATHEPNFTIIREEFKPNKPKPCGLCNQFGHEVKDCEGLPREKKGKHDELADSLPCAEGE

Uniprot       FIFLRLNVLREYLERELTMASLPFTFDVERSIDDWVFMCFFVGNDFLPHLPSLEIRENAIDRLVNIYKNVVHKTGGYLTESGYVNLQRVQMIMLAVGEVE
Splooce       FIFLRLNVLREYLERELTMASLPFTFDVERSIDDWVFMCFFVGNDFLPHLPSLEIRENAIDRLVNIYKNVVHKTGGYLTESGYVNLQRVQMIMLAVGEVE

Uniprot       DSIFKKRKDDEDSFRRRQKEKRKRMKRDQPAFTPSGILTPHALGSRNSPGSQVASNPRQAAYEMRMQNNSSPSISPNTSFTSDGSPSPLGGIKRKAEDSD
Splooce       DSIFKKRKDDEDSFRRRQKEKRKRMKRDQPAFTPSGILTPHALGSRNSPGSQVASNPRQAAYEMRMQNNSSPSISPNTSFTSDGSPSPLGGIKRKAEDSD

Uniprot       SEPEPEDNVRLWEAGWKQRYYKNKFDVDAADEKFRRKVVQSYVEGLCWVLRYYYQGCASWKWYYPFHYAPFASDFEGIADMPSDFEKGTKPFKPLEQLMG
Splooce       SEPEPEDNVRLWEAGWKQRYYKNKFDVDAADEKFRRKVVQSYVEGLCWVLRYYYQGCASWKWYYPFHYAPFASDFEGIADMPSDFEKGTKPFKPLEQLMG

Uniprot       VFPAASGNFLPPSWRKLMSDPDSSIIDFYPEDFAIDLNGKKYAWQGVALLPFVDERRLRAALEEVYPDLTPEETRRNSLGGDVLFVGKHHPLHDFILELY
Splooce       VFPAASGNFLPPSWRKLMSDPDSSIIDFYPEDFAIDLNGKKYAWQGVALLPFVDERRLRAALEEVYPDLTPEETRRNSLGGDVLFVGKHHPLHDFILELY

Uniprot       QTGSTEPVEVPPELCHGIQGKFSLDEEAILPDQIVCSPVPMLRDLTQNTVVSINFKDPQFAEDYIFKAVMLPGARKPAAVLKPSDWEKSSNGRQWKPQLG
Splooce       QTGSTEPVEVPPELCHGIQGKFSLDEEAILPDQIVCSPVPMLRDLTQNTVVSINFKDPQFAEDYIFKAVMLPGARKPAAVLKPSDWEKSSNGRQWKPQLG

Uniprot       FNRDRRPVHLDQAAFRTLGHVMPRGSGTGIYSNAAPPPVTYQGNLYRPLLRGQAQIPKLMSNMRPQDSWRGPPPLFQQQRFDRGVGAEPLLPWNRMLQTQ
Splooce       FNRDRRPVHLDQAAFRTLGHVMPRGSGTGIYSNAAPPPVTYQGNLYRPLLRGQAQIPKLMSNMRPQDSWRGPPPLFQQQRFDRGVGAEPLLPWNRMLQTQ

Uniprot       NAAFQPNQYQMLAGPGGYPPRRDDRGGRQGYPREGRKYPLPPPSGRYNWN
Splooce       NAAFQPNQYQMLAGPGGYPPRRDDRGGRQGYPREGRKYPLPPPSGRYNWN

----------------------------------------------------------------------------------------------------

O00560 (Uniprot)	versus
NM_001007067#(f-T:8_S9083553032426) (Splooce)

For more details about the Alternative Splicing Event -> Link to Splooce page

Peptides that support the ASE (Splooce-specific):
MVAPVTGNDVGIR (MAXQUANT)
VAPVTGNDVGIR (MAXQUANT)

Alignment:
Uniprot       MSLYPSLEDLKVDKVIQAQTAFSANPANPAILSEASAPIPHDGNLYPRLYPELSQYMGLSLNEEEIRANVAVVSGAPLQGQLVARPSSINYMVAPVTGND
Splooce       -------------------------------------------------------------------------------------------MVAPVTGND

Uniprot       VGIRRAEIKQGIREVILCKDQDGKIGLRLKSIDNGIFVQLVQANSPASLVGLRFGDQVLQINGENCAGWSSDKAHKVLKQAFGEKITMTIRDRPFERTIT
Splooce       VGIRRAEIKQGIREVILCKDQDGKIGLRLKSIDNGIFVQLVQANSPASLVGLRFGDQVLQINGENCAGWSSDKAHKVLKQAFGEKITMTIRDRPFERTIT

Uniprot       MHKDSTGHVGFIFKNGKITSIVKDSSAARNGLLTEHNICEINGQNVIGLKDSQIADILSTSGTVVTITIMPAFIFEHIIKRMAPSIMKSLMDHTIPEV
Splooce       MHKDSTGHVGFIFKNGKITSIVKDSSAARNGLLTEHNICEINGQNVIGLKDSQIADILSTSGTVVTITIMPAFIFEHIIKRMAPSIMKSLMDHTIPEV

----------------------------------------------------------------------------------------------------

E7EQR6 (Uniprot)	versus
NM_001008897#(r:6_T6649360987496) (Splooce)

For more details about the Alternative Splicing Event -> Link to Splooce page

Peptides that support the ASE (Splooce-specific):
LGQAEEVVQER (MAXQUANT + PEAKS)

Alignment:
Uniprot       MSSKIIGINGDFFANMVVDAVLAIKYTDIRGQPRYPVNSVNILKAHGRSQMESMLISGYALNCVVGSQGMPKRIVNAKIACLDFSLQKTKMKLGVQVVIT
Splooce       ----------------------------------------------------------------------------------------------------

Uniprot       DPEKLDQIRQRESDITKERIQKILATGANVILTTGGIDDMCLKYFVEAGAMAVRRVLKRDLKRIAKASGATILSTLANLEGEETFEAAMLGQAEEVVQER
Splooce       ----------------------------------------------------------------------------------------MLGQAEEVVQER

Uniprot       ICDDELILIKNTKARTSASIILRGANDFMCDEMERSLHDALCVVKRVLESKSVVPGGGAVEAALSIYLENYATSMGSREQLAIAEFARSLLVIPNTLAVN
Splooce       ICDDELILIKNTKARTSASIILRGANDFMCDEMERSLHDALCVVKRVLESKSVVPGGGAVEAALSIYLENYATSMGSREQLAIAEFARSLLVIPNTLAVN

Uniprot       AAQDSTDLVAKLRAFHNEAQVNPERKNLKWIGLDLSNGKPRDNKQAGVFEPTIVKVKSLKFATEAAITILRIDDLIKLHPESKDDKHGSYEDAVHSGALN
Splooce       AAQDSTDLVAKLRAFHNEAQVNPERKNLKWIGLDLSNGKPRDNKQAGVFEPTIVKVKSLKFATEAAITILRIDDLIKLHPESKDDKHGSYEDAVHSGALN

Uniprot       D
Splooce       D

----------------------------------------------------------------------------------------------------

P08238 (Uniprot)	versus
NM_007355#(f-t:6_H2523249386284) (Splooce)

For more details about the Alternative Splicing Event -> Link to Splooce page

Peptides that support the ASE (Splooce-specific):
DSCDELIPEYLNFIR (MAXQUANT)

Alignment:
Uniprot       MPEEVHHGEEEVETFAFQAEIAQLMSLIINTFYSNKEIFLRELISNASDALDKIRYESLTDPSKLDSGKELKIDIIPNPQERTLTLVDTGIGMTKADLIN
Splooce       ----------------------------------------------------------------------------------------------------

Uniprot       NLGTIAKSGTKAFMEALQAGADISMIGQFGVGFYSAYLVAEKVVVITKHNDDEQYAWESSAGGSFTVRADHGEPIGRGTKVILHLKEDQTEYLEERRVKE
Splooce       ----------------------------------------------------------------------------------------------------

Uniprot       VVKKHSQFIGYPITLYLEKEREKEISDDEAEEEKGEKEEEDKDDEEKPKIEDVGSDEEDDSGKDKKKKTKKIKEKYIDQEELNKTKPIWTRNPDDITQEE
Splooce       ----------------------------------------------------------------------------------------------------

Uniprot       YGEFYKSLTNDWEDHLAVKHFSVEGQLEFRALLFIPRRAPFDLFENKKKKNNIKLYVRRVFIMDSCDELIPEYLNFIRGVVDSEDLPLNISREMLQQSKI
Splooce       --------------------------------------------------------------MDSCDELIPEYLNFIRGVVDSEDLPLNISREMLQQSKI

Uniprot       LKVIRKNIVKKCLELFSELAEDKENYKKFYEAFSKNLKLGIHEDSTNRRRLSELLRYHTSQSGDEMTSLSEYVSRMKETQKSIYYITGESKEQVANSAFV
Splooce       LKVIRKNIVKKCLELFSELAEDKENYKKFYEAFSKNLKLGIHEDSTNRRRLSELLRYHTSQSGDEMTSLSEYVSRMKETQKSIYYITGESKEQVANSAFV

Uniprot       ERVRKRGFEVVYMTEPIDEYCVQQLKEFDGKSLVSVTKEGLELPEDEEEKKKMEESKAKFENLCKLMKEILDKKVEKVTISNRLVSSPCCIVTSTYGWTA
Splooce       ERVRKRGFEVVYMTEPIDEYCVQQLKEFDGKSLVSVTKEGLELPEDEEEKKKMEESKAKFENLCKLMKEILDKKVEKVTISNRLVSSPCCIVTSTYGWTA

Uniprot       NMERIMKAQALRDNSTMGYMMAKKHLEINPDHPIVETLRQKAEADKNDKAVKDLVVLLFETALLSSGFSLEDPQTHSNRIYRMIKLGLGIDEDEVAAEEP
Splooce       NMERIMKAQALRDNSTMGYMMAKKHLEINPDHPIVETLRQKAEADKNDKAVKDLVVLLFETALLSSGFSLEDPQTHSNRIYRMIKLGLGIDEDEVAAEEP

Uniprot       NAAVPDEIPPLEGDEDASRMEEVD
Splooce       NAAVPDEIPPLEGDEDASRMEEVD

----------------------------------------------------------------------------------------------------

P62701 (Uniprot)	versus
NM_001007#(f-:X_R86583991653) (Splooce)

For more details about the Alternative Splicing Event -> Link to Splooce page

Peptides that support the ASE (Splooce-specific):
MVTGGANLGR (MAXQUANT)
VTGGANLGR (MAXQUANT)

Alignment:
Uniprot       MARGPKKHLKRVAAPKHWMLDKLTGVFAPRPSTGPHKLRECLPLIIFLRNRLKYALTGDEVKKICMQRFIKIDGKVRTDITYPAGFMDVISIDKTGENFR
Splooce       ----------------------------------------------------------------------------------------------------

Uniprot       LIYDTKGRFAVHRITPEEAKYKLCKVRKIFVGTKGIPHLVTHDARTIRYPDPLIKVNDTIQIDLETGKITDFIKFDTGNLCMVTGGANLGRIGVITNRER
Splooce       ---------------------------------------------------------------------------------MVTGGANLGRIGVITNRER

Uniprot       HPGSFDVVHVKDANGNSFATRLSNIFVIGKGNKPWISLPRGKGIRLTIAEERDKRLAAKQSSG
Splooce       HPGSFDVVHVKDANGNSFATRLSNIFVIGKGNKPWISLPRGKGIRLTIAEERDKRLAAKQSSG

----------------------------------------------------------------------------------------------------

Q96QC0 (Uniprot)	versus
NM_002714#(-s-s-s-s-:6_P8536515365401) (Splooce)

For more details about the Alternative Splicing Event -> Link to Splooce page

Peptides that support the ASE (Splooce-specific):
EGLGFLDALNSAPVPGIK (MAXQUANT)

Alignment:
Uniprot       MGSGPIDPKELLKGLDSFLNRDGEVKSVDGISKIFSLMKEARKMVSRCTYLNILLQTRSPEILVKFIDVGGYKLLNNWLTYSKTTNNIPLLQQILLTLQH
Splooce       ----------------------------------------------------------------------------------------------------

Uniprot       LPLTVDHLKQNNTAKLVKQLSKSSEDEELRKLASVLVSDWMAVIRSQSSTQPAEKDKKKRKDEGKSRTTLPERPLTEVKAETRAEEAPEKKREKPKSLRT
Splooce       ----------------------------------------------------------------------------------------------------

Uniprot       TAPSHAKFRSTGLELETPSLVPVKKNASTVVVSDKYNLKPIPLKRQSNVAAPGDATPPAEKKYKPLNTTPNATKEIKVKIIPPQPMEGLGFLDALNSAPV
Splooce       -------------------------------------------------------------------------------------MEGLGFLDALNSAPV

Uniprot       PGIKIKKKKKVLSPTAAKPSPFEGKTSTEPSTAKPSSPEPAPPSEAMDADRPGTPVPPVEVPELMDTASLEPGALDAKPVESPGDPNQLTRKGRKRKSVT
Splooce       PGIKIKKKKKVLSPTAAKPSPFEGKTSTEPSTAKPSSPEPAPPSEAMDADRPGTPVPPVEVPELMDTASLEPGALDAKPVESPGDPNQLTRKGRKRKSVT

Uniprot       WPEEGKLREYFYFELDETERVNVNKIKDFGEAAKREILSDRHAFETARRLSHDNMEEKVPWVCPRPLVLPSPLVTPGSNSQERYIQAEREKGILQELFLN
Splooce       WPEEGKLREYFYFELDETERVNVNKIKDFGEAAKREILSDRHAFETARRLSHDNMEEKVPWVCPRPLVLPSPLVTPGSNSQERYIQAEREKGILQELFLN

Uniprot       KESPHEPDPEPYEPIPPKLIPLDEECSMDETPYVETLEPGGSGGSPDGAGGSKLPPVLANLMGSMGAGKGPQGPGGGGINVQEILTSIMGSPNSHPSEEL
Splooce       KESPHEPDPEPYEPIPPKLIPLDEECSMDETPYVETLEPGGSGGSPDGAGGSKLPPVLANLMGSMGAGKGPQGPGGGGINVQEILTSIMGSPNSHPSEEL

Uniprot       LKQPDYSDKIKQMLVPHGLLGPGPIANGFPPGGPGGPKGMQHFPPGPGGPMPGPHGGPGGPVGPRLLGPPPPPRGGDPFWDGPGDPMRGGPMRGGPGPGP
Splooce       LKQPDYSDKIKQMLVPHGLLGPGPIANGFPPGGPGGPKGMQHFPPGPGGPMPGPHGGPGGPVGPRLLGPPPPPRGGDPFWDGPGDPMRGGPMRGGPGPGP

Uniprot       GPYHRGRGGRGGNEPPPPPPPFRGARGGRSGGGPPNGRGGPGGGMVGGGGHRPHEGPGGGMGNSSGHRPHEGPGGGMGSGHRPHEGPGGSMGGGGGHRPH
Splooce       GPYHRGRGGRGGNEPPPPPPPFRGARGGRSGGGPPNGRGGPGGGMVGGGGHRPHEGPGGGMGNSSGHRPHEGPGGGMGSGHRPHEGPGGSMGGGGGHRPH

Uniprot       EGPGGGISGGSGHRPHEGPGGGMGAGGGHRPHEGPGGSMGGSGGHRPHEGPGHGGPHGHRPHDVPGHRGHDHRGPPPHEHRGHDGPGHGGGGHRGHDGGH
Splooce       EGPGGGISGGSGHRPHEGPGGGMGAGGGHRPHEGPGGSMGGSGGHRPHEGPGHGGPHGHRPHDVPGHRGHDHRGPPPHEHRGHDGPGHGGGGHRGHDGGH

Uniprot       SHGGDMSNRPVCRHFMMKGNCRYENNCAFYHPGVNGPPLP
Splooce       SHGGDMSNRPVCRHFMMKGNCRYENNCAFYHPGVNGPPLP

----------------------------------------------------------------------------------------------------

P68363 (Uniprot)	versus
NM_006082#(-t:12_T1377782446991) (Splooce)

For more details about the Alternative Splicing Event -> Link to Splooce page

Peptides that support the ASE (Splooce-specific):
AGVQIGNACWELYCLEHGIQPDGQMPSDK (MAXQUANT)

Alignment:
Uniprot       MRECISIHVGQAGVQIGNACWELYCLEHGIQPDGQMPSDKTIGGGDDSFNTFFSETGAGKHVPRAVFVDLEPTVIDEVRTGTYRQLFHPEQLITGKEDAA
Splooce       ----------MAGVQIGNACWELYCLEHGIQPDGQMPSDKTIGGGDDSFNTFFSETGAGKHVPRAVFVDLEPTVIDEVRTGTYRQLFHPEQLITGKEDAA

Uniprot       NNYARGHYTIGKEIIDLVLDRIRKLADQCTGLQGFLVFHSFGGGTGSGFTSLLMERLSVDYGKKSKLEFSIYPAPQVSTAVVEPYNSILTTHTTLEHSDC
Splooce       NNYARGHYTIGKEIIDLVLDRIRKLADQCTGLQGFLVFHSFGGGTGSGFTSLLMERLSVDYGKKSKLEFSIYPAPQVSTAVVEPYNSILTTHTTLEHSDC

Uniprot       AFMVDNEAIYDICRRNLDIERPTYTNLNRLISQIVSSITASLRFDGALNVDLTEFQTNLVPYPRIHFPLATYAPVISAEKAYHEQLSVAEITNACFEPAN
Splooce       AFMVDNEAIYDICRRNLDIERPTYTNLNRLISQIVSSITASLRFDGALNVDLTEFQTNLVPYPRIHFPLATYAPVISAEKAYHEQLSVAEITNACFEPAN

Uniprot       QMVKCDPRHGKYMACCLLYRGDVVPKDVNAAIATIKTKRSIQFVDWCPTGFKVGINYQPPTVVPGGDLAKVQRAVCMLSNTTAIAEAWARLDHKFDLMYA
Splooce       QMVKCDPRHGKYMACCLLYRGDVVPKDVNAAIATIKTKRSIQFVDWCPTGFKVGINYQPPTVVPGGDLAKVQRAVCMLSNTTAIAEAWARLDHKFDLMYA

Uniprot       KRAFVHWYVGEGMEEGEFSEAREDMAALEKDYEEVGVDSVEGEGEEEGEEY
Splooce       KRAFVHWYVGEGMEEGEFSEAREDMAALEKDYEEVGVDSVEGEGEEEGEEY

----------------------------------------------------------------------------------------------------

P19174 (Uniprot)	versus
NM_002660#(-s-s-s-s-s-s-:20_P2170779407277) (Splooce)

For more details about the Alternative Splicing Event -> Link to Splooce page

Peptides that support the ASE (Splooce-specific):
MYSENDLSNSLK (PEAKS)

Alignment:
Uniprot       MAGAASPCANGCGPGAPSDAEVLHLCRSLEVGTVMTLFYSKKSQRPERKTFQVKLETRQITWSRGADKIEGAIDIREIKEIRPGKTSRDFDRYQEDPAFR
Splooce       ----------------------------------------------------------------------------------------------------

Uniprot       PDQSHCFVILYGMEFRLKTLSLQATSEDEVNMWIKGLTWLMEDTLQAPTPLQIERWLRKQFYSVDRNREDRISAKDLKNMLSQVNYRVPNMRFLRERLTD
Splooce       ----------------------------------------------------------------------------------------------------

Uniprot       LEQRSGDITYGQFAQLYRSLMYSAQKTMDLPFLEASTLRAGERPELCRVSLPEFQQFLLDYQGELWAVDRLQVQEFMLSFLRDPLREIEEPYFFLDEFVT
Splooce       ----------------------------------------------------------------------------------------------------

Uniprot       FLFSKENSVWNSQLDAVCPDTMNNPLSHYWISSSHNTYLTGDQFSSESSLEAYARCLRMGCRCIELDCWDGPDGMPVIYHGHTLTTKIKFSDVLHTIKEH
Splooce       ----------------------------------------------------------------------------------------------------

Uniprot       AFVASEYPVILSIEDHCSIAQQRNMAQYFKKVLGDTLLTKPVEISADGLPSPNQLKRKILIKHKKLAEGSAYEEVPTSMMYSENDISNSIKNGILYLEDP
Splooce       ------------------------------------------------------------------------------MMYSENDISNSIKNGILYLEDP

Uniprot       VNHEWYPHYFVLTSSKIYYSEETSSDQGNEDEEEPKEVSSSTELHSNEKWFHGKLGAGRDGRHIAERLLTEYCIETGAPDGSFLVRESETFVGDYTLSFW
Splooce       VNHEWYPHYFVLTSSKIYYSEETSSDQGNEDEEEPKEVSSSTELHSNEKWFHGKLGAGRDGRHIAERLLTEYCIETGAPDGSFLVRESETFVGDYTLSFW

Uniprot       RNGKVQHCRIHSRQDAGTPKFFLTDNLVFDSLYDLITHYQQVPLRCNEFEMRLSEPVPQTNAHESKEWYHASLTRAQAEHMLMRVPRDGAFLVRKRNEPN
Splooce       RNGKVQHCRIHSRQDAGTPKFFLTDNLVFDSLYDLITHYQQVPLRCNEFEMRLSEPVPQTNAHESKEWYHASLTRAQAEHMLMRVPRDGAFLVRKRNEPN

Uniprot       SYAISFRAEGKIKHCRVQQEGQTVMLGNSEFDSLVDLISYYEKHPLYRKMKLRYPINEEALEKIGTAEPDYGALYEGRNPGFYVEANPMPTFKCAVKALF
Splooce       SYAISFRAEGKIKHCRVQQEGQTVMLGNSEFDSLVDLISYYEKHPLYRKMKLRYPINEEALEKIGTAEPDYGALYEGRNPGFYVEANPMPTFKCAVKALF

Uniprot       DYKAQREDELTFIKSAIIQNVEKQEGGWWRGDYGGKKQLWFPSNYVEEMVNPVALEPEREHLDENSPLGDLLRGVLDVPACQIAIRPEGKNNRLFVFSIS
Splooce       DYKAQREDELTFIKSAIIQNVEKQEGGWWRGDYGGKKQLWFPSNYVEEMVNPVALEPEREHLDENSPLGDLLRGVLDVPACQIAIRPEGKNNRLFVFSIS

Uniprot       MASVAHWSLDVAADSQEELQDWVKKIREVAQTADARLTEGKIMERRKKIALELSELVVYCRPVPFDEEKIGTERACYRDMSSFPETKAEKYVNKAKGKKF
Splooce       MASVAHWSLDVAADSQEELQDWVKKIREVAQTADARLTEGKIMERRKKIALELSELVVYCRPVPFDEEKIGTERACYRDMSSFPETKAEKYVNKAKGKKF

Uniprot       LQYNRLQLSRIYPKGQRLDSSNYDPLPMWICGSQLVALNFQTPDKPMQMNQALFMTGRHCGYVLQPSTMRDEAFDPFDKSSLRGLEPCAISIEVLGARHL
Splooce       LQYNRLQLSRIYPKGQRLDSSNYDPLPMWICGSQLVALNFQTPDKPMQMNQALFMTGRHCGYVLQPSTMRDEAFDPFDKSSLRGLEPCAISIEVLGARHL

Uniprot       PKNGRGIVCPFVEIEVAGAEYDSTKQKTEFVVDNGLNPVWPAKPFHFQISNPEFAFLRFVVYEEDMFSDQNFLAQATFPVKGLKTGYRAVPLKNNYSEDL
Splooce       PKNGRGIVCPFVEIEVAGAEYDSTKQKTEFVVDNGLNPVWPAKPFHFQISNPEFAFLRFVVYEEDMFSDQNFLAQATFPVKGLKTGYRAVPLKNNYSEDL

Uniprot       ELASLLIKIDIFPAKQENGDLSPFSGTSLRERGSDASGQLFHGRAREGSFESRYQQPFEDFRISQEHLADHFDSRERRAPRRTRVNGDNRL
Splooce       ELASLLIKIDIFPAKQENGDLSPFSGTSLRERGSDASGQLFHGRAREGSFESRYQQPFEDFRISQEHLADHFDSRERRAPRRTRVNGDNRL

----------------------------------------------------------------------------------------------------

Q9UMR2 (Uniprot)	versus
NM_007242#(-s-s-s-s-:16_D9759995911078) (Splooce)

For more details about the Alternative Splicing Event -> Link to Splooce page

Peptides that support the ASE (Splooce-specific):
LSQVEPANK (MAXQUANT)

Alignment:
Uniprot       MATDSWALAVDEQEAAAESLSNLHLKEEKIKPDTNGAVVKTNANAEKTDEEEKEDRAAQSLLNKLIRSNLVDNTNQVEVLQRDPNSPLYSVKSFEELRLK
Splooce       ----------------------------------------------------------------------------------------------------

Uniprot       PQLLQGVYAMGFNRPSKIQENALPLMLAEPPQNLIAQSQSGTGKTAAFVLAMLSQVEPANKYPQCLCLSPTYELALQTGKVIEQMGKFYPELKLAYAVRG
Splooce       ---------------------------------------------------MLSQVEPANKYPQCLCLSPTYELALQTGKVIEQMGKFYPELKLAYAVRG

Uniprot       NKLERGQKISEQIVIGTPGTVLDWCSKLKFIDPKKIKVFVLDEADVMIATQGHQDQSIRIQRMLPRNCQMLLFSATFEDSVWKFAQKVVPDPNVIKLKRE
Splooce       NKLERGQKISEQIVIGTPGTVLDWCSKLKFIDPKKIKVFVLDEADVMIATQGHQDQSIRIQRMLPRNCQMLLFSATFEDSVWKFAQKVVPDPNVIKLKRE

Uniprot       EETLDTIKQYYVLCSSRDEKFQALCNLYGAITIAQAMIFCHTRKTASWLAAELSKEGHQVALLSGEMMVEQRAAVIERFREGKEKVLVTTNVCARGIDVE
Splooce       EETLDTIKQYYVLCSSRDEKFQALCNLYGAITIAQAMIFCHTRKTASWLAAELSKEGHQVALLSGEMMVEQRAAVIERFREGKEKVLVTTNVCARGIDVE

Uniprot       QVSVVINFDLPVDKDGNPDNETYLHRIGRTGRFGKRGLAVNMVDSKHSMNILNRIQEHFNKKIERLDTDDLDEIEKIAN
Splooce       QVSVVINFDLPVDKDGNPDNETYLHRIGRTGRFGKRGLAVNMVDSKHSMNILNRIQEHFNKKIERLDTDDLDEIEKIAN

----------------------------------------------------------------------------------------------------

O75832 (Uniprot)	versus
NM_002814#(f-:X_P3789858353578) (Splooce)

For more details about the Alternative Splicing Event -> Link to Splooce page

Peptides that support the ASE (Splooce-specific):
LLEGGANPDAK (MAXQUANT)

Alignment:
Uniprot       MEGCVSNLMVCNLAYSGKLEELKESILADKSLATRTDQDSRTALHWACSAGHTEIVEFLLQLGVPVNDKDDAGWSPLHIAASAGRDEIVKALLGKGAQVN
Splooce       ----------------------------------------------------------------------------------------------------

Uniprot       AVNQNGCTPLHYAASKNRHEIAVMLLEGGANPDAKDHYEATAMHRAAAKGNLKMIHILLYYKASTNIQDTEGNTPLHLACDEERVEEAKLLVSQGASIYI
Splooce       -----------------------MLLEGGANPDAKDHYEATAMHRAAAKGNLKMIHILLYYKASTNIQDTEGNTPLHLACDEERVEEAKLLVSQGASIYI

Uniprot       ENKEEKTPLQVAKGGLGLILKRMVEG
Splooce       ENKEEKTPLQVAKGGLGLILKRMVEG

----------------------------------------------------------------------------------------------------

Q15437 (Uniprot)	versus
NM_032986#(-s-:20_S8400864503629) (Splooce)

For more details about the Alternative Splicing Event -> Link to Splooce page

Peptides that support the ASE (Splooce-specific):
PQFSTIEYVIQR (MAXQUANT)

Alignment:
Uniprot       MATYLEFIQQNEERDGVRFSWNVWPSSRLEATRMVVPLACLLTPLKERPDLPPVQYEPVLCSRPTCKAVLNPLCQVDYRAKLWACNFCFQRNQFPPAYGG
Splooce       ----------------------------------------------------------------------------------------------------

Uniprot       ISEVNQPAELMPQFSTIEYVIQRGAQSPLIFLYVVDTCLEEDDLQALKESLQMSLSLLPPDALVGLITFGRMVQVHELSCEGISKSYVFRGTKDLTAKQI
Splooce       ----------MPQFSTIEYVIQRGAQSPLIFLYVVDTCLEEDDLQALKESLQMSLSLLPPDALVGLITFGRMVQVHELSCEGISKSYVFRGTKDLTAKQI

Uniprot       QDMLGLTKPAMPMQQARPAQPQEHPFASSRFLQPVHKIDMNLTDLLGELQRDPWPVTQGKRPLRSTGVALSIAVGLLEGTFPNTGARIMLFTGGPPTQGP
Splooce       QDMLGLTKPAMPMQQARPAQPQEHPFASSRFLQPVHKIDMNLTDLLGELQRDPWPVTQGKRPLRSTGVALSIAVGLLEGTFPNTGARIMLFTGGPPTQGP

Uniprot       GMVVGDELKIPIRSWHDIEKDNARFMKKATKHYEMLANRTAANGHCIDIYACALDQTGLLEMKCCANLTGGYMVMGDSFNTSLFKQTFQRIFTKDFNGDF
Splooce       GMVVGDELKIPIRSWHDIEKDNARFMKKATKHYEMLANRTAANGHCIDIYACALDQTGLLEMKCCANLTGGYMVMGDSFNTSLFKQTFQRIFTKDFNGDF

Uniprot       RMAFGATLDVKTSRELKIAGAIGPCVSLNVKGPCVSENELGVGGTSQWKICGLDPTSTLGIYFEVVNQHNTPIPQGGRGAIQFVTHYQHSSTQRRIRVTT
Splooce       RMAFGATLDVKTSRELKIAGAIGPCVSLNVKGPCVSENELGVGGTSQWKICGLDPTSTLGIYFEVVNQHNTPIPQGGRGAIQFVTHYQHSSTQRRIRVTT

Uniprot       IARNWADVQSQLRHIEAAFDQEAAAVLMARLGVFRAESEEGPDVLRWLDRQLIRLCQKFGQYNKEDPTSFRLSDSFSLYPQFMFHLRRSPFLQVFNNSPD
Splooce       IARNWADVQSQLRHIEAAFDQEAAAVLMARLGVFRAESEEGPDVLRWLDRQLIRLCQKFGQYNKEDPTSFRLSDSFSLYPQFMFHLRRSPFLQVFNNSPD

Uniprot       ESSYYRHHFARQDLTQSLIMIQPILYSYSFHGPPEPVLLDSSSILADRILLMDTFFQIVIYLGETIAQWRKAGYQDMPEYENFKHLLQAPLDDAQEILQA
Splooce       ESSYYRHHFARQDLTQSLIMIQPILYSYSFHGPPEPVLLDSSSILADRILLMDTFFQIVIYLGETIAQWRKAGYQDMPEYENFKHLLQAPLDDAQEILQA

Uniprot       RFPMPRYINTEHGGSQARFLLSKVNPSQTHNNLYAWGQETGAPILTDDVSLQVFMDHLKKLAVSSAC
Splooce       RFPMPRYINTEHGGSQARFLLSKVNPSQTHNNLYAWGQETGAPILTDDVSLQVFMDHLKKLAVSSAC

----------------------------------------------------------------------------------------------------

P35749 (Uniprot)	versus
NM_001040113#(-s-:16_M7715832938354) (Splooce)

For more details about the Alternative Splicing Event -> Link to Splooce page

Peptides that support the ASE (Splooce-specific):
FLLEQEEYQR (PEAKS)

Alignment:
Uniprot       MAQKGQLSDDEKFLFVDKNFINSPVAQADWAAKRLVWVPSEKQGFEAASIKEEKGDEVVVELVENGKKVTVGKDDIQKMNPPKFSKVEDMAELTCLNEAS
Splooce       ----------------------------------------------------------------------------------------------------

Uniprot       VLHNLRERYFSGLIYTYSGLFCVVVNPYKHLPIYSEKIVDMYKGKKRHEMPPHIYAIADTAYRSMLQDREDQSILCTGESGAGKTENTKKVIQYLAVVAS
Splooce       ----------------------------------------------------------------------------------------------------

Uniprot       SHKGKKDTSITQGPSFAYGELEKQLLQANPILEAFGNAKTVKNDNSSRFGKFIRINFDVTGYIVGANIETYLLEKSRAIRQARDERTFHIFYYMIAGAKE
Splooce       ----------------------------------------------------------------------------------------------------

Uniprot       KMRSDLLLEGFNNYTFLSNGFVPIPAAQDDEMFQETVEAMAIMGFSEEEQLSILKVVSSVLQLGNIVFKKERNTDQASMPDNTAAQKVCHLMGINVTDFT
Splooce       ----------------------------------------------------------------------------------------------------

Uniprot       RSILTPRIKVGRDVVQKAQTKEQADFAVEALAKATYERLFRWILTRVNKALDKTHRQGASFLGILDIAGFEIFEVNSFEQLCINYTNEKLQQLFNHTMFI
Splooce       -------------------------------------------------------------------------------------------------MFI

Uniprot       LEQEEYQREGIEWNFIDFGLDLQPCIELIERPNNPPGVLALLDEECWFPKATDKSFVEKLCTEQGSHPKFQKPKQLKDKTEFSIIHYAGKVDYNASAWLT
Splooce       LEQEEYQREGIEWNFIDFGLDLQPCIELIERPNNPPGVLALLDEECWFPKATDKSFVEKLCTEQGSHPKFQKPKQLKDKTEFSIIHYAGKVDYNASAWLT

Uniprot       KNMDPLNDNVTSLLNASSDKFVADLWKDVDRIVGLDQMAKMTESSLPSASKTKKGMFRTVGQLYKEQLGKLMTTLRNTTPNFVRCIIPNHEKRSGKLDAF
Splooce       KNMDPLNDNVTSLLNASSDKFVADLWKDVDRIVGLDQMAKMTESSLPSASKTKKGMFRTVGQLYKEQLGKLMTTLRNTTPNFVRCIIPNHEKRSGKLDAF

Uniprot       LVLEQLRCNGVLEGIRICRQGFPNRIVFQEFRQRYEILAANAIPKGFMDGKQACILMIKALELDPNLYRIGQSKIFFRTGVLAHLEEERDLKITDVIMAF
Splooce       LVLEQLRCNGVLEGIRICRQGFPNRIVFQEFRQRYEILAANAIPKGFMDGKQACILMIKALELDPNLYRIGQSKIFFRTGVLAHLEEERDLKITDVIMAF

Uniprot       QAMCRGYLARKAFAKRQQQLTAMKVIQRNCAAYLKLRNWQWWRLFTKVKPLLQVTRQEEEMQAKEDELQKTKERQQKAENELKELEQKHSQLTEEKNLLQ
Splooce       QAMCRGYLARKAFAKRQQQLTAMKVIQRNCAAYLKLRNWQWWRLFTKVKPLLQVTRQEEEMQAKEDELQKTKERQQKAENELKELEQKHSQLTEEKNLLQ

Uniprot       EQLQAETELYAEAEEMRVRLAAKKQELEEILHEMEARLEEEEDRGQQLQAERKKMAQQMLDLEEQLEEEEAARQKLQLEKVTAEAKIKKLEDEILVMDDQ
Splooce       EQLQAETELYAEAEEMRVRLAAKKQELEEILHEMEARLEEEEDRGQQLQAERKKMAQQMLDLEEQLEEEEAARQKLQLEKVTAEAKIKKLEDEILVMDDQ

Uniprot       NNKLSKERKLLEERISDLTTNLAEEEEKAKNLTKLKNKHESMISELEVRLKKEEKSRQELEKLKRKLEGDASDFHEQIADLQAQIAELKMQLAKKEEELQ
Splooce       NNKLSKERKLLEERISDLTTNLAEEEEKAKNLTKLKNKHESMISELEVRLKKEEKSRQELEKLKRKLEGDASDFHEQIADLQAQIAELKMQLAKKEEELQ

Uniprot       AALARLDDEIAQKNNALKKIRELEGHISDLQEDLDSERAARNKAEKQKRDLGEELEALKTELEDTLDSTATQQELRAKREQEVTVLKKALDEETRSHEAQ
Splooce       AALARLDDEIAQKNNALKKIRELEGHISDLQEDLDSERAARNKAEKQKRDLGEELEALKTELEDTLDSTATQQELRAKREQEVTVLKKALDEETRSHEAQ

Uniprot       VQEMRQKHAQAVEELTEQLEQFKRAKANLDKNKQTLEKENADLAGELRVLGQAKQEVEHKKKKLEAQVQELQSKCSDGERARAELNDKVHKLQNEVESVT
Splooce       VQEMRQKHAQAVEELTEQLEQFKRAKANLDKNKQTLEKENADLAGELRVLGQAKQEVEHKKKKLEAQVQELQSKCSDGERARAELNDKVHKLQNEVESVT

Uniprot       GMLNEAEGKAIKLAKDVASLSSQLQDTQELLQEETRQKLNVSTKLRQLEEERNSLQDQLDEEMEAKQNLERHISTLNIQLSDSKKKLQDFASTVEALEEG
Splooce       GMLNEAEGKAIKLAKDVASLSSQLQDTQELLQEETRQKLNVSTKLRQLEEERNSLQDQLDEEMEAKQNLERHISTLNIQLSDSKKKLQDFASTVEALEEG

Uniprot       KKRFQKEIENLTQQYEEKAAAYDKLEKTKNRLQQELDDLVVDLDNQRQLVSNLEKKQRKFDQLLAEEKNISSKYADERDRAEAEAREKETKALSLARALE
Splooce       KKRFQKEIENLTQQYEEKAAAYDKLEKTKNRLQQELDDLVVDLDNQRQLVSNLEKKQRKFDQLLAEEKNISSKYADERDRAEAEAREKETKALSLARALE

Uniprot       EALEAKEELERTNKMLKAEMEDLVSSKDDVGKNVHELEKSKRALETQMEEMKTQLEELEDELQATEDAKLRLEVNMQALKGQFERDLQARDEQNEEKRRQ
Splooce       EALEAKEELERTNKMLKAEMEDLVSSKDDVGKNVHELEKSKRALETQMEEMKTQLEELEDELQATEDAKLRLEVNMQALKGQFERDLQARDEQNEEKRRQ

Uniprot       LQRQLHEYETELEDERKQRALAAAAKKKLEGDLKDLELQADSAIKGREEAIKQLRKLQAQMKDFQRELEDARASRDEIFATAKENEKKAKSLEADLMQLQ
Splooce       LQRQLHEYETELEDERKQRALAAAAKKKLEGDLKDLELQADSAIKGREEAIKQLRKLQAQMKDFQRELEDARASRDEIFATAKENEKKAKSLEADLMQLQ

Uniprot       EDLAAAERARKQADLEKEELAEELASSLSGRNALQDEKRRLEARIAQLEEELEEEQGNMEAMSDRVRKATQQAEQLSNELATERSTAQKNESARQQLERQ
Splooce       EDLAAAERARKQADLEKEELAEELASSLSGRNALQDEKRRLEARIAQLEEELEEEQGNMEAMSDRVRKATQQAEQLSNELATERSTAQKNESARQQLERQ

Uniprot       NKELRSKLHEMEGAVKSKFKSTIAALEAKIAQLEEQVEQEAREKQAATKSLKQKDKKLKEILLQVEDERKMAEQYKEQAEKGNARVKQLKRQLEEAEEES
Splooce       NKELRSKLHEMEGAVKSKFKSTIAALEAKIAQLEEQVEQEAREKQAATKSLKQKDKKLKEILLQVEDERKMAEQYKEQAEKGNARVKQLKRQLEEAEEES

Uniprot       QRINANRRKLQRELDEATESNEAMGREVNALKSKLRGPPPQETSQ
Splooce       QRINANRRKLQRELDEATESNEAMGREVNALKSKLRGPPPQETSQ

----------------------------------------------------------------------------------------------------

P61221 (Uniprot)	versus
NM_001040876#(-s-s-:4_A1914840111016) (Splooce)

For more details about the Alternative Splicing Event -> Link to Splooce page

Peptides that support the ASE (Splooce-specific):
MFDEPSSYLDVK (MAXQUANT + PEAKS)

Alignment:
Uniprot       MADKLTRIAIVNHDKCKPKKCRQECKKSCPVVRMGKLCIEVTPQSKIAWISETLCIGCGICIKKCPFGALSIVNLPSNLEKETTHRYCANAFKLHRLPIP
Splooce       ----------------------------------------------------------------------------------------------------

Uniprot       RPGEVLGLVGTNGIGKSTALKILAGKQKPNLGKYDDPPDWQEILTYFRGSELQNYFTKILEDDLKAIIKPQYVDQIPKAAKGTVGSILDRKDETKTQAIV
Splooce       ----------------------------------------------------------------------------------------------------

Uniprot       CQQLDLTHLKERNVEDLSGGELQRFACAVVCIQKADIFMFDEPSSYLDVKQRLKAAITIRSLINPDRYIIVVEHDLSVLDYLSDFICCLYGVPSAYGVVT
Splooce       --------------------------------------MFDEPSSYLDVKQRLKAAITIRSLINPDRYIIVVEHDLSVLDYLSDFICCLYGVPSAYGVVT

Uniprot       MPFSVREGINIFLDGYVPTENLRFRDASLVFKVAETANEEEVKKMCMYKYPGMKKKMGEFELAIVAGEFTDSEIMVMLGENGTGKTTFIRMLAGRLKPDE
Splooce       MPFSVREGINIFLDGYVPTENLRFRDASLVFKVAETANEEEVKKMCMYKYPGMKKKMGEFELAIVAGEFTDSEIMVMLGENGTGKTTFIRMLAGRLKPDE

Uniprot       GGEVPVLNVSYKPQKISPKSTGSVRQLLHEKIRDAYTHPQFVTDVMKPLQIENIIDQEVQTLSGGELQRVALALCLGKPADVYLIDEPSAYLDSEQRLMA
Splooce       GGEVPVLNVSYKPQKISPKSTGSVRQLLHEKIRDAYTHPQFVTDVMKPLQIENIIDQEVQTLSGGELQRVALALCLGKPADVYLIDEPSAYLDSEQRLMA

Uniprot       ARVVKRFILHAKKTAFVVEHDFIMATYLADRVIVFDGVPSKNTVANSPQTLLAGMNKFLSQLEITFRRDPNNYRPRINKLNSIKDVEQKKSGNYFFLDD
Splooce       ARVVKRFILHAKKTAFVVEHDFIMATYLADRVIVFDGVPSKNTVANSPQTLLAGMNKFLSQLEITFRRDPNNYRPRINKLNSIKDVEQKKSGNYFFLDD

----------------------------------------------------------------------------------------------------

P13693 (Uniprot)	versus
NM_003295#(r:13_T5832837127521) (Splooce)

For more details about the Alternative Splicing Event -> Link to Splooce page

Peptides that support the ASE (Splooce-specific):
NHHLQETSFTK (MAXQUANT)

Alignment:
Uniprot       MIIYRDLISHDEMFSDIYKIREIADGLCLEVEGKMVSRTEGNIDDSLIGGNASAEGPEGEGTESTVITGVDIVMNHHLQETSFTKEAYKKYIKDYMKSIK
Splooce       -------------------------------------------------------------------------MNHHLQETSFTKEAYKKYIKDYMKSIK

Uniprot       GKLEEQRPERVKPFMTGAAEQIKHILANFKNYQFFIGENMNPDGMVALLDYREDGVTPYMIFFKDGLEMEKC
Splooce       GKLEEQRPERVKPFMTGAAEQIKHILANFKNYQFFIGENMNPDGMVALLDYREDGVTPYMIFFKDGLEMEKC

----------------------------------------------------------------------------------------------------

E9PQG7 (Uniprot)	versus
NM_005933#(-s-:11_M3692168634723) (Splooce)

For more details about the Alternative Splicing Event -> Link to Splooce page

Peptides that support the ASE (Splooce-specific):
ELDEQFLGFGSDEEVR (MAXQUANT)

Alignment:
Uniprot       MAHSCRWRFPARPGTTGGGGGGGRRGLGGAPRQRVPALLLPPGPPVGGGGPGAPPSPPAVAAAAAAAGSSGAGVPGGAAAASAASSSSASSSSSSSSSAS
Splooce       ----------------------------------------------------------------------------------------------------

Uniprot       SGPALLRVGPGFDAALQVSAAIGTNLRRFRAVFGESGGGGGSGEDEQFLGFGSDEEVRVRSPTRSPSVKTSPRKPRGRPRSGSDRNSAILSDPSVFSPLN
Splooce       -----------------------------------------MELDEQFLGFGSDEEVRVRSPTRSPSVKTSPRKPRGRPRSGSDRNSAILSDPSVFSPLN

Uniprot       KSETKSGDKIKKKDSKSIEKKRGRPPTFPGVKIKITHGKDISELPKGNKEDSLKKIKRTPSATFQQATKIKKLRAGKLSPLKSKFKTGKLQIGRKGVQIV
Splooce       KSETKSGDKIKKKDSKSIEKKRGRPPTFPGVKIKITHGKDISELPKGNKEDSLKKIKRTPSATFQQATKIKKLRAGKLSPLKSKFKTGKLQIGRKGVQIV

Uniprot       RRRGRPPSTERIKTPSGLLINSELEKPQKVRKDKEGTPPLTKEDKTVVRQSPRRIKPVRIIPSSKRTDATIAKQLLQRAKKGAQKKIEKEAAQLQGRKVK
Splooce       RRRGRPPSTERIKTPSGLLINSELEKPQKVRKDKEGTPPLTKEDKTVVRQSPRRIKPVRIIPSSKRTDATIAKQLLQRAKKGAQKKIEKEAAQLQGRKVK

Uniprot       TQVKNIRQFIMPVVSAISSRIIKTPRRFIEDEDYDPPIKIARLESTPNSRFSAPSCGSSEKSSAASQHSSQMSSDSSRSSSPSVDTSTDSQASEEIQVLP
Splooce       TQVKNIRQFIMPVVSAISSRIIKTPRRFIEDEDYDPPIKIARLESTPNSRFSAPSCGSSEKSSAASQHSSQMSSDSSRSSSPSVDTSTDSQASEEIQVLP

Uniprot       EERSDTPEVHPPLPISQSPENESNDRRSRRYSVSERSFGSRTTKKLSTLQSAPQQQTSSSPPPPLLTPPPPLQPASSISDHTPWLMPPTIPLASPFLPAS
Splooce       EERSDTPEVHPPLPISQSPENESNDRRSRRYSVSERSFGSRTTKKLSTLQSAPQQQTSSSPPPPLLTPPPPLQPASSISDHTPWLMPPTIPLASPFLPAS

Uniprot       TAPMQGKRKSILREPTFRWTSLKHSRSEPQYFSSAKYAKEGLIRKPIFDNFRPPPLTPEDVGFASGFSASGTAASARLFSPLHSGTRFDMHKRSPLLRAP
Splooce       TAPMQGKRKSILREPTFRWTSLKHSRSEPQYFSSAKYAKEGLIRKPIFDNFRPPPLTPEDVGFASGFSASGTAASARLFSPLHSGTRFDMHKRSPLLRAP

Uniprot       RFTPSEAHSRIFESVTLPSNRTSAGTSSSGVSNRKRKRKVFSPIRSEPRSPSHSMRTRSGRLSSSELSPLTPPSSVSSSLSISVSPLATSALNPTFTFPS
Splooce       RFTPSEAHSRIFESVTLPSNRTSAGTSSSGVSNRKRKRKVFSPIRSEPRSPSHSMRTRSGRLSSSELSPLTPPSSVSSSLSISVSPLATSALNPTFTFPS

Uniprot       HSLTQSGESAEKNQRPRKQTSAPAEPFSSSSPTPLFPWFTPGSQTERGRNKDKAPEELSKDRDADKSVEKDKSRERDREREKENKRESRKEKRKKGSEIQ
Splooce       HSLTQSGESAEKNQRPRKQTSAPAEPFSSSSPTPLFPWFTPGSQTERGRNKDKAPEELSKDRDADKSVEKDKSRERDREREKENKRESRKEKRKKGSEIQ

Uniprot       SSSALYPVGRVSKEKVVGEDVATSSSAKKATGRKKSSSHDSGTDITSVTLGDTTAVKTKILIKKGRGNLEKTNLDLGPTAPSLEKEKTLCLSTPSSSTVK
Splooce       SSSALYPVGRVSKEKVVGEDVATSSSAKKATGRKKSSSHDSGTDITSVTLGDTTAVKTKILIKKGRGNLEKTNLDLGPTAPSLEKEKTLCLSTPSSSTVK

Uniprot       HSTSSIGSMLAQADKLPMTDKRVASLLKKAKAQLCKIEKSKSLKQTDQPKAQGQESDSSETSVRGPRIKHVCRRAAVALGRKRAVFPDDMPTLSALPWEE
Splooce       HSTSSIGSMLAQADKLPMTDKRVASLLKKAKAQLCKIEKSKSLKQTDQPKAQGQESDSSETSVRGPRIKHVCRRAAVALGRKRAVFPDDMPTLSALPWEE

Uniprot       REKILSSMGNDDKSSIAGSEDAEPLAPPIKPIKPVTRNKAPQEPPVKKGRRSRRCGQCPGCQVPEDCGVCTNCLDKPKFGGRNIKKQCCKMRKCQNLQWM
Splooce       REKILSSMGNDDKSSIAGSEDAEPLAPPIKPIKPVTRNKAPQEPPVKKGRRSRRCGQCPGCQVPEDCGVCTNCLDKPKFGGRNIKKQCCKMRKCQNLQWM

Uniprot       PSKAYLQKQAKAVKKKEKKSKTSEKKDSKESSVVKNVVDSSQKPTPSAREDPAPKKSSSEPPPRKPVEEKSEEGNVSAPGPESKQATTPASRKSSKQVSQ
Splooce       PSKAYLQKQAKAVKKKEKKSKTSEKKDSKESSVVKNVVDSSQKPTPSAREDPAPKKSSSEPPPRKPVEEKSEEGNVSAPGPESKQATTPASRKSSKQVSQ

Uniprot       PALVIPPQPPTTGPPRKEVPKTTPSEPKKKQPPPPESGPEQSKQKKVAPRPSIPVKQKPKEKEKPPPVNKQENAGTLNILSTLSNGNSSKQKIPADGVHR
Splooce       PALVIPPQPPTTGPPRKEVPKTTPSEPKKKQPPPPESGPEQSKQKKVAPRPSIPVKQKPKEKEKPPPVNKQENAGTLNILSTLSNGNSSKQKIPADGVHR

Uniprot       IRVDFKEDCEAENVWEMGGLGILTSVPITPRVVCFLCASSGHVEFVYCQVCCEPFHKFCLEENERPLEDQLENWCCRRCKFCHVCGRQHQATKQLLECNK
Splooce       IRVDFKEDCEAENVWEMGGLGILTSVPITPRVVCFLCASSGHVEFVYCQVCCEPFHKFCLEENERPLEDQLENWCCRRCKFCHVCGRQHQATKQLLECNK

Uniprot       CRNSYHPECLGPNYPTKPTKKKKVWICTKCVRCKSCGSTTPGKGWDAQWSHDFSLCHDCAKLFAKGNFCPLCDKCYDDDDYESKMMQCGKCDRWVHSKCE
Splooce       CRNSYHPECLGPNYPTKPTKKKKVWICTKCVRCKSCGSTTPGKGWDAQWSHDFSLCHDCAKLFAKGNFCPLCDKCYDDDDYESKMMQCGKCDRWVHSKCE

Uniprot       NLSDEMYEILSNLPESVAYTCVNCTERHPAEWRLALEKELQISLKQVLTALLNSRTTSHLLRYRQAAKPPDLNPETEESIPSRSSPEGPDPPVLTEVSKQ
Splooce       NLSDEMYEILSNLPESVAYTCVNCTERHPAEWRLALEKELQISLKQVLTALLNSRTTSHLLRYRQAAKPPDLNPETEESIPSRSSPEGPDPPVLTEVSKQ

Uniprot       DDQQPLDLEGVKRKMDQGNYTSVLEFSDDIVKIIQAAINSDGGQPEIKKANSMVKSFFIRQMERVFPWFSVKKSRFWEPNKVSSNSGMLPNAVLPPSLDH
Splooce       DDQQPLDLEGVKRKMDQGNYTSVLEFSDDIVKIIQAAINSDGGQPEIKKANSMVKSFFIRQMERVFPWFSVKKSRFWEPNKVSSNSGMLPNAVLPPSLDH

Uniprot       NYAQWQEREENSHTEQPPLMKKIIPAPKPKGPGEPDSPTPLHPPTPPILSTDRSREDSPELNPPPGIEDNRQCALCLTYGDDSANDAGRLLYIGQNEWTH
Splooce       NYAQWQEREENSHTEQPPLMKKIIPAPKPKGPGEPDSPTPLHPPTPPILSTDRSREDSPELNPPPGIEDNRQCALCLTYGDDSANDAGRLLYIGQNEWTH

Uniprot       VNCALWSAEVFEDDDGSLKNVHMAVIRGKQLRCEFCQKPGATVGCCLTSCTSNYHFMCSRAKNCVFLDDKKVYCQRHRDLIKGEVVPENGFEVFRRVFVD
Splooce       VNCALWSAEVFEDDDGSLKNVHMAVIRGKQLRCEFCQKPGATVGCCLTSCTSNYHFMCSRAKNCVFLDDKKVYCQRHRDLIKGEVVPENGFEVFRRVFVD

Uniprot       FEGISLRRKFLNGLEPENIHMMIGSMTIDCLGILNDLSDCEDKLFPIGYQCSRVYWSTTDARKRCVYTCKIVECRPPVVEPDINSTVEHDENRTIAHSPT
Splooce       FEGISLRRKFLNGLEPENIHMMIGSMTIDCLGILNDLSDCEDKLFPIGYQCSRVYWSTTDARKRCVYTCKIVECRPPVVEPDINSTVEHDENRTIAHSPT

Uniprot       SFTESSSKESQNTAEIISPPSPDRPPHSQTSGSCYYHVISKVPRIRTPSYSPTQRSPGCRPLPSAGSPTPTTHEIVTVGDPLLSSGLRSIGSRRHSTSSL
Splooce       SFTESSSKESQNTAEIISPPSPDRPPHSQTSGSCYYHVISKVPRIRTPSYSPTQRSPGCRPLPSAGSPTPTTHEIVTVGDPLLSSGLRSIGSRRHSTSSL

Uniprot       SPQRSKLRIMSPMRTGNTYSRNNVSSVSTTGTATDLESSAKVVDHVLGPLNSSTSLGQNTSTSSNLQRTVVTVGNKNSHLDGSSSSEMKQSSASDLVSKS
Splooce       SPQRSKLRIMSPMRTGNTYSRNNVSSVSTTGTATDLESSAKVVDHVLGPLNSSTSLGQNTSTSSNLQRTVVTVGNKNSHLDGSSSSEMKQSSASDLVSKS

Uniprot       SSLKGEKTKVLSSKSSEGSAHNVAYPGIPKLAPQVHNTTSRELNVSKIGSFAEPSSVSFSSKEALSFPHLHLRGQRNDRDQHTDSTQSANSSPDEDTEVK
Splooce       SSLKGEKTKVLSSKSSEGSAHNVAYPGIPKLAPQVHNTTSRELNVSKIGSFAEPSSVSFSSKEALSFPHLHLRGQRNDRDQHTDSTQSANSSPDEDTEVK

Uniprot       TLKLSGMSNRSSIINEHMGSSSRDRRQKGKKSCKETFKEKHSSKSFLEPGQVTTGEEGNLKPEFMDEVLTPEYMGQRPCNNVSSDKIGDKGLSMPGVPKA
Splooce       TLKLSGMSNRSSIINEHMGSSSRDRRQKGKKSCKETFKEKHSSKSFLEPGQVTTGEEGNLKPEFMDEVLTPEYMGQRPCNNVSSDKIGDKGLSMPGVPKA

Uniprot       PPMQVEGSAKELQAPRKRTVKVTLTPLKMENESQSKNALKESSPASPLQIESTSPTEPISASENPGDGPVAQPSPNNTSCQDSQSNNYQNLPVQDRNLML
Splooce       PPMQVEGSAKELQAPRKRTVKVTLTPLKMENESQSKNALKESSPASPLQIESTSPTEPISASENPGDGPVAQPSPNNTSCQDSQSNNYQNLPVQDRNLML

Uniprot       PDGPKPQEDGSFKRRYPRRSARARSNMFFGLTPLYGVRSYGEEDIPFYSSSTGKKRGKRSAEGQVDGADDLSTSDEDDLYYYNFTRTVISSGGEERLASH
Splooce       PDGPKPQEDGSFKRRYPRRSARARSNMFFGLTPLYGVRSYGEEDIPFYSSSTGKKRGKRSAEGQVDGADDLSTSDEDDLYYYNFTRTVISSGGEERLASH

Uniprot       NLFREEEQCDLPKISQLDGVDDGTESDTSVTATTRKSSQIPKRNGKENGTENLKIDRPEDAGEKEHVTKSSVGHKNEPKMDNCHSVSRVKTQGQDSLEAQ
Splooce       NLFREEEQCDLPKISQLDGVDDGTESDTSVTATTRKSSQIPKRNGKENGTENLKIDRPEDAGEKEHVTKSSVGHKNEPKMDNCHSVSRVKTQGQDSLEAQ

Uniprot       LSSLESSRRVHTSTPSDKNLLDTYNTELLKSDSDNNNSDDCGNILPSDIMDFVLKNTPSMQALGESPESSSSELLNLGEGLGLDSNREKDMGLFEVFSQQ
Splooce       LSSLESSRRVHTSTPSDKNLLDTYNTELLKSDSDNNNSDDCGNILPSDIMDFVLKNTPSMQALGESPESSSSELLNLGEGLGLDSNREKDMGLFEVFSQQ

Uniprot       LPTTEPVDSSVSSSISAEEQFELPLELPSDLSVLTTRSPTVPSQNPSRLAVISDSGEKRVTITEKSVASSESDPALLSPGVDPTPEGHMTPDHFIQGHMD
Splooce       LPTTEPVDSSVSSSISAEEQFELPLELPSDLSVLTTRSPTVPSQNPSRLAVISDSGEKRVTITEKSVASSESDPALLSPGVDPTPEGHMTPDHFIQGHMD

Uniprot       ADHISSPPCGSVEQGHGNNQDLTRNSSTPGLQVPVSPTVPIQNQKYVPNSTDSPGPSQISNAAVQTTPPHLKPATEKLIVVNQNMQPLYVLQTLPNGVTQ
Splooce       ADHISSPPCGSVEQGHGNNQDLTRNSSTPGLQVPVSPTVPIQNQKYVPNSTDSPGPSQISNAAVQTTPPHLKPATEKLIVVNQNMQPLYVLQTLPNGVTQ

Uniprot       KIQLTSSVSSTPSVMETNTSVLGPMGGGLTLTTGLNPSLPTSQSLFPSASKGLLPMSHHQHLHSFPAATQSSFPPNISNPPSGLLIGVQPPPDPQLLVSE
Splooce       KIQLTSSVSSTPSVMETNTSVLGPMGGGLTLTTGLNPSLPTSQSLFPSASKGLLPMSHHQHLHSFPAATQSSFPPNISNPPSGLLIGVQPPPDPQLLVSE

Uniprot       SSQRTDLSTTVATPSSGLKKRPISRLQTRKNKKLAPSSTPSNIAPSDVVSNMTLINFTPSQLPNHPSLLDLGSLNTSSHRTVPNIIKRSKSSIMYFEPAP
Splooce       SSQRTDLSTTVATPSSGLKKRPISRLQTRKNKKLAPSSTPSNIAPSDVVSNMTLINFTPSQLPNHPSLLDLGSLNTSSHRTVPNIIKRSKSSIMYFEPAP

Uniprot       LLPQSVGGTAATAAGTSTISQDTSHLTSGSVSGLASSSSVLNVVSMQTTTTPTSSASVPGHVTLTNPRLLGTPDIGSISNLLIKASQQSLGIQDQPVALP
Splooce       LLPQSVGGTAATAAGTSTISQDTSHLTSGSVSGLASSSSVLNVVSMQTTTTPTSSASVPGHVTLTNPRLLGTPDIGSISNLLIKASQQSLGIQDQPVALP

Uniprot       PSSGMFPQLGTSQTPSTAAITAASSICVLPSTQTTGITAASPSGEADEHYQLQHVNQLLASKTGIHSSQRDLDSASGPQVSNFTQTVDAPNSMGLEQNKA
Splooce       PSSGMFPQLGTSQTPSTAAITAASSICVLPSTQTTGITAASPSGEADEHYQLQHVNQLLASKTGIHSSQRDLDSASGPQVSNFTQTVDAPNSMGLEQNKA

Uniprot       LSSAVQASPTSPGGSPSSPSSGQRSASPSVPGPTKPKPKTKRFQLPLDKGNGKKHKVSHLRTSSSEAHIPDQETTSLTSGTGTPGAEAEQQDTASVEQSS
Splooce       LSSAVQASPTSPGGSPSSPSSGQRSASPSVPGPTKPKPKTKRFQLPLDKGNGKKHKVSHLRTSSSEAHIPDQETTSLTSGTGTPGAEAEQQDTASVEQSS

Uniprot       QKECGQPAGQVAVLPEVQVTQNPANEQESAEPKTVEEEESNFSSPLMLWLQQEQKRKESITEKKPKKGLVFEISSDDGFQICAESIEDAWKSLTDKVQEA
Splooce       QKECGQPAGQVAVLPEVQVTQNPANEQESAEPKTVEEEESNFSSPLMLWLQQEQKRKESITEKKPKKGLVFEISSDDGFQICAESIEDAWKSLTDKVQEA

Uniprot       RSNARLKQLSFAGVNGLRMLGILHDAVVFLIEQLSGAKHCRNYKFRFHKPEEANEPPLNPHGSARAEVHLRQSAFDMFNFLASKHRQPPEYNPNDEEEEE
Splooce       RSNARLKQLSFAGVNGLRMLGILHDAVVFLIEQLSGAKHCRNYKFRFHKPEEANEPPLNPHGSARAEVHLRQSAFDMFNFLASKHRQPPEYNPNDEEEEE

Uniprot       VQLKSARRATSMDLPMPMRFRHLKKTSKEAVGVYRSPIHGRGLFCKRNIDAGEMVIEYAGNVIRSIQTDKREKYYDSKGIGCYMFRIDDSEVVDATMHGN
Splooce       VQLKSARRATSMDLPMPMRFRHLKKTSKEAVGVYRSPIHGRGLFCKRNIDAGEMVIEYAGNVIRSIQTDKREKYYDSKGIGCYMFRIDDSEVVDATMHGN

Uniprot       AARFINHSCEPNCYSRVINIDGQKHIVIFAMRKIYRGEELTYDYKFPIEDASNKLPCNCGAKKCRKFLN
Splooce       AARFINHSCEPNCYSRVINIDGQKHIVIFAMRKIYRGEELTYDYKFPIEDASNKLPCNCGAKKCRKFLN

----------------------------------------------------------------------------------------------------

Q96TA1 (Uniprot)	versus
NM_022833#(-s-:9_F2188690202861) (Splooce)

For more details about the Alternative Splicing Event -> Link to Splooce page

Peptides that support the ASE (Splooce-specific):
MTEAEQDKWQAVLQDCIR (MAXQUANT)
MMTEAEQDKWQAVLQDCIR (MAXQUANT)

Alignment:
Uniprot       MGDVLSTHLDDARRQHIAEKTGKILTEFLQFYEDQYGVALFNSMRHEIEGTGLPQAQLLWRKVPLDERIVFSGNLFQHQEDSKKWRNRFSLVPHNYGLVL
Splooce       ----------------------------------------------------------------------------------------------------

Uniprot       YENKAAYERQVPPRAVINSAGYKILTSVDQYLELIGNSLPGTTAKSGSAPILKCPTQFPLILWHPYARHYYFCMMTEAEQDKWQAVLQDCIRHCNNGIPE
Splooce       -------------------------------------------------------------------------MMTEAEQDKWQAVLQDCIRHCNNGIPE

Uniprot       DSKVEGPAFTDAIRMYRQSKELYGTWEMLCGNEVQILSNLVMEELGPELKAELGPRLKGKPQERQRQWIQISDAVYHMVYEQAKARFEEVLSKVQQVQPA
Splooce       DSKVEGPAFTDAIRMYRQSKELYGTWEMLCGNEVQILSNLVMEELGPELKAELGPRLKGKPQERQRQWIQISDAVYHMVYEQAKARFEEVLSKVQQVQPA

Uniprot       MQAVIRTDMDQIITSKEHLASKIRAFILPKAEVCVRNHVQPYIPSILEALMVPTSQGFTEVRDVFFKEVTDMNLNVINEGGIDKLGEYMEKLSRLAYHPL
Splooce       MQAVIRTDMDQIITSKEHLASKIRAFILPKAEVCVRNHVQPYIPSILEALMVPTSQGFTEVRDVFFKEVTDMNLNVINEGGIDKLGEYMEKLSRLAYHPL

Uniprot       KMQSCYEKMESLRLDGLQQRFDVSSTSVFKQRAQIHMREQMDNAVYTFETLLHQELGKGPTKEELCKSIQRVLERVLKKYDYDSSSVRKRFFREALLQIS
Splooce       KMQSCYEKMESLRLDGLQQRFDVSSTSVFKQRAQIHMREQMDNAVYTFETLLHQELGKGPTKEELCKSIQRVLERVLKKYDYDSSSVRKRFFREALLQIS

Uniprot       IPFLLKKLAPTCKSELPRFQELIFEDFARFILVENTYEEVVLQTVMKDILQAVKEAAVQRKHNLYRDSMVMHNSDPNLHLLAEGAPIDWGEEYSNSGGGG
Splooce       IPFLLKKLAPTCKSELPRFQELIFEDFARFILVENTYEEVVLQTVMKDILQAVKEAAVQRKHNLYRDSMVMHNSDPNLHLLAEGAPIDWGEEYSNSGGGG

Uniprot       SPSPSTPESATLSEKRRRAKQVVSVVQDEEVGLPFEASPESPPPASPDGVTEIRGLLAQGLRPESPPPAGPLLNGAPAGESPQPKAAPEASSPPASPLQH
Splooce       SPSPSTPESATLSEKRRRAKQVVSVVQDEEVGLPFEASPESPPPASPDGVTEIRGLLAQGLRPESPPPAGPLLNGAPAGESPQPKAAPEASSPPASPLQH

Uniprot       LLPGKAVDLGPPKPSDQETGEQVSSPSSHPALHTTTEDSAGVQTEF
Splooce       LLPGKAVDLGPPKPSDQETGEQVSSPSSHPALHTTTEDSAGVQTEF

----------------------------------------------------------------------------------------------------

P51571 (Uniprot)	versus
NM_006280#(-s-:X_S4499666619117) (Splooce)

For more details about the Alternative Splicing Event -> Link to Splooce page

Peptides that support the ASE (Splooce-specific):
MALYADVGGK (PEAKS)

Alignment:
Uniprot       MAAMASLGALALLLLSSLSRCSAEACLEPQITPSYYTTSDAVISTETVFIVEISLTCKNRVQNMALYADVGGKQFPVTRGQDVGRYQVSWSLDHKSAHAG
Splooce       ---------------------------------------------------------------MALYADVGGKQFPVTRGQDVGRYQVSWSLDHKSAHAG

Uniprot       TYEVRFFDEESYSLLRKAQRNNEDISIIPPLFTVSVDHRGTWNGPWVSTEVLAAAIGLVIYYLAFSAKSHIQA
Splooce       TYEVRFFDEESYSLLRKAQRNNEDISIIPPLFTVSVDHRGTWNGPWVSTEVLAAAIGLVIYYLAFSAKSHIQA

----------------------------------------------------------------------------------------------------

Q9NVH1 (Uniprot)	versus
NM_018198#(-t:1_D9666029552967) (Splooce)

For more details about the Alternative Splicing Event -> Link to Splooce page

Peptides that support the ASE (Splooce-specific):
EGWEVVER (MAXQUANT)

Alignment:
Uniprot       MATALSEEELDNEDYYSLLNVRREASSEELKAAYRRLCMLYHPDKHRDPELKSQAERLFNLVHQAYEVLSDPQTRAIYDIYGKRGLEMEGWEVVERRRTP
Splooce       ---------------------------------------------------------------------------------------MEGWEVVERRRTP

Uniprot       AEIREEFERLQREREERRLQQRTNPKGTISVGVDATDLFDRYDEEYEDVSGSSFPQIEINKMHISQSIEAPLTATDTAILSGSLSTQNGNGGGSINFALR
Splooce       AEIREEFERLQREREERRLQQRTNPKGTISVGVDATDLFDRYDEEYEDVSGSSFPQIEINKMHISQSIEAPLTATDTAILSGSLSTQNGNGGGSINFALR

Uniprot       RVTSAKGWGELEFGAGDLQGPLFGLKLFRNLTPRCFVTTNCALQFSSRGIRPGLTTVLARNLDKNTVGYLQWRWGIQSAMNTSIVRDTKTSHFTVALQLG
Splooce       RVTSAKGWGELEFGAGDLQGPLFGLKLFRNLTPRCFVTTNCALQFSSRGIRPGLTTVLARNLDKNTVGYLQWRWGIQSAMNTSIVRDTKTSHFTVALQLG

Uniprot       IPHSFALISYQHKFQDDDQTRVKGSLKAGFFGTVVEYGAERKISRHSVLGAAVSVGVPQGVSLKVKLNRASQTYFFPIHLTDQLLPSAMFYATVGPLVVY
Splooce       IPHSFALISYQHKFQDDDQTRVKGSLKAGFFGTVVEYGAERKISRHSVLGAAVSVGVPQGVSLKVKLNRASQTYFFPIHLTDQLLPSAMFYATVGPLVVY

Uniprot       FAMHRLIIKPYLRAQKEKELEKQRESAATDVLQKKQEAESAVRLMQESVRRIIEAEESRMGLIIVNAWYGKFVNDKSRKSEKVKVIDVTVPLQCLVKDSK
Splooce       FAMHRLIIKPYLRAQKEKELEKQRESAATDVLQKKQEAESAVRLMQESVRRIIEAEESRMGLIIVNAWYGKFVNDKSRKSEKVKVIDVTVPLQCLVKDSK

Uniprot       LILTEASKAGLPGFYDPCVGEEKNLKVLYQFRGVLHQVMVLDSEALRIPKQSHRIDTDG
Splooce       LILTEASKAGLPGFYDPCVGEEKNLKVLYQFRGVLHQVMVLDSEALRIPKQSHRIDTDG

----------------------------------------------------------------------------------------------------

P27708 (Uniprot)	versus
NM_004341#(r:2_C822560548985) (Splooce)

For more details about the Alternative Splicing Event -> Link to Splooce page

Peptides that support the ASE (Splooce-specific):
NVAYTDGDLER (MAXQUANT)

Alignment:
Uniprot       MAALVLEDGSVLRGQPFGAAVSTAGEVVFQTGMVGYPEALTDPSYKAQILVLTYPLIGNYGIPPDEMDEFGLCKWFESSGIHVAALVVGECCPTPSHWSA
Splooce       ----------------------------------------------------------------------------------------------------

Uniprot       TRTLHEWLQQHGIPGLQGVDTRELTKKLREQGSLLGKLVQNGTEPSSLPFLDPNARPLVPEVSIKTPRVFNTGGAPRILALDCGLKYNQIRCLCQRGAEV
Splooce       ----------------------------------------------------------------------------------------------------

Uniprot       TVVPWDHALDSQEYEGLFLSNGPGDPASYPSVVSTLSRVLSEPNPRPVFGICLGHQLLALAIGAKTYKMRYGNRGHNQPCLLVGSGRCFLTSQNHGFAVE
Splooce       ----------------------------------------------------------------------------------------------------

Uniprot       TDSLPADWAPLFTNANDGSNEGIVHNSLPFFSVQFHPEHQAGPSDMELLFDIFLETVKEATAGNPGGQTVRERLTERLCPPGIPTPGSGLPPPRKVLILG
Splooce       ----------------------------------------------------------------------------------------------------

Uniprot       SGGLSIGQAGEFDYSGSQAIKALKEENIQTLLINPNIATVQTSQGLADKVYFLPITPHYVTQVIRNERPDGVLLTFGGQTALNCGVELTKAGVLARYGVR
Splooce       ----------------------------------------------------------------------------------------------------

Uniprot       VLGTPVETIELTEDRRAFAARMAEIGEHVAPSEAANSLEQAQAAAERLGYPVLVRAAFALGGLGSGFASNREELSALVAPAFAHTSQVLVDKSLKGWKEI
Splooce       ----------------------------------------------------------------------------------------------------

Uniprot       EYEVVRDAYGNCVTVCNMENLDPLGIHTGESIVVAPSQTLNDREYQLLRQTAIKVTQHLGIVGECNVQYALNPESEQYYIIEVNARLSRSSALASKATGY
Splooce       ----------------------------------------------------------------------------------------------------

Uniprot       PLAYVAAKLALGIPLPELRNSVTGGTAAFEPSVDYCVVKIPRWDLSKFLRVSTKIGSCMKSVGEVMGIGRSFEEAFQKALRMVDENCVGFDHTVKPVSDM
Splooce       ----------------------------------------------------------------------------------------------------

Uniprot       ELETPTDKRIFVVAAALWAGYSVDRLYELTRIDRWFLHRMKRIIAHAQLLEQHRGQPLPPDLLQQAKCLGFSDKQIALAVLSTELAVRKLRQELGICPAV
Splooce       ----------------------------------------------------------------------------------------------------

Uniprot       KQIDTVAAEWPAQTNYLYLTYWGTTHDLTFRTPHVLVLGSGVYRIGSSVEFDWCAVGCIQQLRKMGYKTIMVNYNPETVSTDYDMCDRLYFDEISFEVVM
Splooce       ----------------------------------------------------------------------------------------------------

Uniprot       DIYELENPEGVILSMGGQLPNNMAMALHRQQCRVLGTSPEAIDSAENRFKFSRLLDTIGISQPQWRELSDLESARQFCQTVGYPCVVRPSYVLSGAAMNV
Splooce       -------------------------------------------------------------------------------------------------MNV

Uniprot       AYTDGDLERFLSSAAAVSKEHPVVISKFIQEAKEIDVDAVASDGVVAAIAISEHVENAGVHSGDATLVTPPQDITAKTLERIKAIVHAVGQELQVTGPFN
Splooce       AYTDGDLERFLSSAAAVSKEHPVVISKFIQEAKEIDVDAVASDGVVAAIAISEHVENAGVHSGDATLVTPPQDITAKTLERIKAIVHAVGQELQVTGPFN

Uniprot       LQLIAKDDQLKVIECNVRVSRSFPFVSKTLGVDLVALATRVIMGEEVEPVGLMTGSGVVGVKVPQFSFSRLAGADVVLGVEMTSTGEVAGFGESRCEAYL
Splooce       LQLIAKDDQLKVIECNVRVSRSFPFVSKTLGVDLVALATRVIMGEEVEPVGLMTGSGVVGVKVPQFSFSRLAGADVVLGVEMTSTGEVAGFGESRCEAYL

Uniprot       KAMLSTGFKIPKKNILLTIGSYKNKSELLPTVRLLESLGYSLYASLGTADFYTEHGVKVTAVDWHFEEAVDGECPPQRSILEQLAEKNFELVINLSMRGA
Splooce       KAMLSTGFKIPKKNILLTIGSYKNKSELLPTVRLLESLGYSLYASLGTADFYTEHGVKVTAVDWHFEEAVDGECPPQRSILEQLAEKNFELVINLSMRGA

Uniprot       GGRRLSSFVTKGYRTRRLAADFSVPLIIDIKCTKLFVEALGQIGPAPPLKVHVDCMTSQKLVRLPGLIDVHVHLREPGGTHKEDFASGTAAALAGGITMV
Splooce       GGRRLSSFVTKGYRTRRLAADFSVPLIIDIKCTKLFVEALGQIGPAPPLKVHVDCMTSQKLVRLPGLIDVHVHLREPGGTHKEDFASGTAAALAGGITMV

Uniprot       CAMPNTRPPIIDAPALALAQKLAEAGARCDFALFLGASSENAGTLGTVAGSAAGLKLYLNETFSELRLDSVVQWMEHFETWPSHLPIVAHAEQQTVAAVL
Splooce       CAMPNTRPPIIDAPALALAQKLAEAGARCDFALFLGASSENAGTLGTVAGSAAGLKLYLNETFSELRLDSVVQWMEHFETWPSHLPIVAHAEQQTVAAVL

Uniprot       MVAQLTQRSVHICHVARKEEILLIKAAKARGLPVTCEVAPHHLFLSHDDLERLGPGKGEVRPELGSRQDVEALWENMAVIDCFASDHAPHTLEEKCGSRP
Splooce       MVAQLTQRSVHICHVARKEEILLIKAAKARGLPVTCEVAPHHLFLSHDDLERLGPGKGEVRPELGSRQDVEALWENMAVIDCFASDHAPHTLEEKCGSRP

Uniprot       PPGFPGLETMLPLLLTAVSEGRLSLDDLLQRLHHNPRRIFHLPPQEDTYVEVDLEHEWTIPSHMPFSKAHWTPFEGQKVKGTVRRVVLRGEVAYIDGQVL
Splooce       PPGFPGLETMLPLLLTAVSEGRLSLDDLLQRLHHNPRRIFHLPPQEDTYVEVDLEHEWTIPSHMPFSKAHWTPFEGQKVKGTVRRVVLRGEVAYIDGQVL

Uniprot       VPPGYGQDVRKWPQGAVPQLPPSAPATSEMTTTPERPRRGIPGLPDGRFHLPPRIHRASDPGLPAEEPKEKSSRKVAEPELMGTPDGTCYPPPPVPRQAS
Splooce       VPPGYGQDVRKWPQGAVPQLPPSAPATSEMTTTPERPRRGIPGLPDGRFHLPPRIHRASDPGLPAEEPKEKSSRKVAEPELMGTPDGTCYPPPPVPRQAS

Uniprot       PQNLGTPGLLHPQTSPLLHSLVGQHILSVQQFTKDQMSHLFNVAHTLRMMVQKERSLDILKGKVMASMFYEVSTRTSSSFAAAMARLGGAVLSFSEATSS
Splooce       PQNLGTPGLLHPQTSPLLHSLVGQHILSVQQFTKDQMSHLFNVAHTLRMMVQKERSLDILKGKVMASMFYEVSTRTSSSFAAAMARLGGAVLSFSEATSS

Uniprot       VQKGESLADSVQTMSCYADVVVLRHPQPGAVELAAKHCRRPVINAGDGVGEHPTQALLDIFTIREELGTVNGMTITMVGDLKHGRTVHSLACLLTQYRVS
Splooce       VQKGESLADSVQTMSCYADVVVLRHPQPGAVELAAKHCRRPVINAGDGVGEHPTQALLDIFTIREELGTVNGMTITMVGDLKHGRTVHSLACLLTQYRVS

Uniprot       LRYVAPPSLRMPPTVRAFVASRGTKQEEFESIEEALPDTDVLYMTRIQKERFGSTQEYEACFGQFILTPHIMTRAKKKMVVMHPMPRVNEISVEVDSDPR
Splooce       LRYVAPPSLRMPPTVRAFVASRGTKQEEFESIEEALPDTDVLYMTRIQKERFGSTQEYEACFGQFILTPHIMTRAKKKMVVMHPMPRVNEISVEVDSDPR

Uniprot       AAYFRQAENGMYIRMALLATVLGRF
Splooce       AAYFRQAENGMYIRMALLATVLGRF

----------------------------------------------------------------------------------------------------

Q15149 (Uniprot)	versus
NM_201381#(-s-s-s-s-s-s-s-s-s-s-:8_P6382015390916) (Splooce)

For more details about the Alternative Splicing Event -> Link to Splooce page

Peptides that support the ASE (Splooce-specific):
QTVQQEQLLQETQALQQSFLSEK (MAXQUANT)

Alignment:
Uniprot       MKIVPDERDRVQKKTFTKWVNKHLIKAQRHISDLYEDLRDGHNLISLLEVLSGDSLPREKGRMRFHKLQNVQIALDYLRHRQVKLVNIRNDDIADGNPKL
Splooce       ----------------------------------------------------------------------------------------------------

Uniprot       TLGLIWTIILHFQISDIQVSGQSEDMTAKEKLLLWSQRMVEGYQGLRCDNFTSSWRDGRLFNAIIHRHKPLLIDMNKVYRQTNLENLDQAFSVAERDLGV
Splooce       ----------------------------------------------------------------------------------------------------

Uniprot       TRLLDPEDVDVPQPDEKSIITYVSSLYDAMPRVPDVQDGVRANELQLRWQEYRELVLLLLQWMRHHTAAFEERRFPSSFEEIEILWSQFLKFKEMELPAK
Splooce       ----------------------------------------------------------------------------------------------------

Uniprot       EADKNRSKGIYQSLEGAVQAGQLKVPPGYHPLDVEKEWGKLHVAILEREKQLRSEFERLECLQRIVTKLQMEAGLCEEQLNQADALLQSDVRLLAAGKVP
Splooce       ----------------------------------------------------------------------------------------------------

Uniprot       QRAGEVERDLDKADSMIRLLFNDVQTLKDGRHPQGEQMYRRVYRLHERLVAIRTEYNLRLKAGVAAPATQVAQVTLQSVQRRPELEDSTLRYLQDLLAWV
Splooce       ----------------------------------------------------------------------------------------------------

Uniprot       EENQHRVDGAEWGVDLPSVEAQLGSHRGLHQSIEEFRAKIERARSDEGQLSPATRGAYRDCLGRLDLQYAKLLNSSKARLRSLESLHSFVAAATKELMWL
Splooce       ----------------------------------------------------------------------------------------------------

Uniprot       NEKEEEEVGFDWSDRNTNMTAKKESYSALMRELELKEKKIKELQNAGDRLLREDHPARPTVESFQAALQTQWSWMLQLCCCIEAHLKENAAYFQFFSDVR
Splooce       ----------------------------------------------------------------------------------------------------

Uniprot       EAEGQLQKLQEALRRKYSCDRSATVTRLEDLLQDAQDEKEQLNEYKGHLSGLAKRAKAVVQLKPRHPAHPMRGRLPLLAVCDYKQVEVTVHKGDECQLVG
Splooce       ----------------------------------------------------------------------------------------------------

Uniprot       PAQPSHWKVLSSSGSEAAVPSVCFLVPPPNQEAQEAVTRLEAQHQALVTLWHQLHVDMKSLLAWQSLRRDVQLIRSWSLATFRTLKPEEQRQALHSLELH
Splooce       ----------------------------------------------------------------------------------------------------

Uniprot       YQAFLRDSQDAGGFGPEDRLMAEREYGSCSHHYQQLLQSLEQGAQEESRCQRCISELKDIRLQLEACETRTVHRLRLPLDKEPARECAQRIAEQQKAQAE
Splooce       ----------------------------------------------------------------------------------------------------

Uniprot       VEGLGKGVARLSAEAEKVLALPEPSPAAPTLRSELELTLGKLEQVRSLSAIYLEKLKTISLVIRGTQGAEEVLRAHEEQLKEAQAVPATLPELEATKASL
Splooce       ----------------------------------------------------------------------------------------------------

Uniprot       KKLRAQAEAQQPTFDALRDELRGAQEVGERLQQRHGERDVEVERWRERVAQLLERWQAVLAQTDVRQRELEQLGRQLRYYRESADPLGAWLQDARRRQEQ
Splooce       ----------------------------------------------------------------------------------------------------

Uniprot       IQAMPLADSQAVREQLRQEQALLEEIERHGEKVEECQRFAKQYINAIKDYELQLVTYKAQLEPVASPAKKPKVQSGSESVIQEYVDLRTHYSELTTLTSQ
Splooce       ----------------------------------------------------------------------------------------------------

Uniprot       YIKFISETLRRMEEEERLAEQQRAEERERLAEVEAALEKQRQLAEAHAQAKAQAEREAKELQQRMQEEVVRREEAAVDAQQQKRSIQEELQQLRQSSEAE
Splooce       ----------------------------------------------------------------------------------------------------

Uniprot       IQAKARQAEAAERSRLRIEEEIRVVRLQLEATERQRGGAEGELQALRARAEEAEAQKRQAQEEAERLRRQVQDESQRKRQAEVELASRVKAEAEAAREKQ
Splooce       ----------------------------------------------------------------------------------------------------

Uniprot       RALQALEELRLQAEEAERRLRQAEVERARQVQVALETAQRSAEAELQSKRASFAEKTAQLERSLQEEHVAVAQLREEAERRAQQQAEAERAREEAERELE
Splooce       ----------------------------------------------------------------------------------------------------

Uniprot       RWQLKANEALRLRLQAEEVAQQKSLAQAEAEKQKEEAEREARRRGKAEEQAVRQRELAEQELEKQRQLAEGTAQQRLAAEQELIRLRAETEQGEQQRQLL
Splooce       ----------------------------------------------------------------------------------------------------

Uniprot       EEELARLQREAAAATQKRQELEAELAKVRAEMEVLLASKARAEEESRSTSEKSKQRLEAEAGRFRELAEEAARLRALAEEAKRQRQLAEEDAARQRAEAE
Splooce       ----------------------------------------------------------------------------------------------------

Uniprot       RVLAEKLAAIGEATRLKTEAEIALKEKEAENERLRRLAEDEAFQRRRLEEQAAQHKADIEERLAQLRKASDSELERQKGLVEDTLRQRRQVEEEILALKA
Splooce       ----------------------------------------------------------------------------------------------------

Uniprot       SFEKAAAGKAELELELGRIRSNAEDTLRSKEQAELEAARQRQLAAEEERRRREAEERVQKSLAAEEEAARQRKAALEEVERLKAKVEEARRLRERAEQES
Splooce       ----------------------------------------------------------------------------------------------------

Uniprot       ARQLQLAQEAAQKRLQAEEKAHAFAVQQKEQELQQTLQQEQSVLDQLRGEAEAARRAAEEAEEARVQAEREAAQSRRQVEEAERLKQSAEEQAQARAQAQ
Splooce       ----------------------------------------------------------------------------------------------------

Uniprot       AAAEKLRKEAEQEAARRAQAEQAALRQKQAADAEMEKHKKFAEQTLRQKAQVEQELTTLRLQLEETDHQKNLLDEELQRLKAEATEAARQRSQVEEELFS
Splooce       ----------------------------------------------------------------------------------------------------

Uniprot       VRVQMEELSKLKARIEAENRALILRDKDNTQRFLQEEAEKMKQVAEEAARLSVAAQEAARLRQLAEEDLAQQRALAEKMLKEKMQAVQEATRLKAEAELL
Splooce       ----------------------------------------------------------------------------------------------------

Uniprot       QQQKELAQEQARRLQEDKEQMAQQLAEETQGFQRTLEAERQRQLEMSAEAERLKLRVAEMSRAQARAEEDAQRFRKQAEEIGEKLHRTELATQEKVTLVQ
Splooce       ----------------------------------------------------------------------------------------------------

Uniprot       TLEIQRQQSDHDAERLREAIAELEREKEKLQQEAKLLQLKSEEMQTVQQEQLLQETQALQQSFLSEKDSLLQRERFIEQEKAKLEQLFQDEVAKAQQLRE
Splooce       -------------------------------------------MQTVQQEQLLQETQALQQSFLSEKDSLLQRERFIEQEKAKLEQLFQDEVAKAQQLRE

Uniprot       EQQRQQQQMEQERQRLVASMEEARRRQHEAEEGVRRKQEELQQLEQQRRQQEELLAEENQRLREQLQLLEEQHRAALAHSEEVTASQVAATKTLPNGRDA
Splooce       EQQRQQQQMEQERQRLVASMEEARRRQHEAEEGVRRKQEELQQLEQQRRQQEELLAEENQRLREQLQLLEEQHRAALAHSEEVTASQVAATKTLPNGRDA

Uniprot       LDGPAAEAEPEHSFDGLRRKVSAQRLQEAGILSAEELQRLAQGHTTVDELARREDVRHYLQGRSSIAGLLLKATNEKLSVYAALQRQLLSPGTALILLEA
Splooce       LDGPAAEAEPEHSFDGLRRKVSAQRLQEAGILSAEELQRLAQGHTTVDELARREDVRHYLQGRSSIAGLLLKATNEKLSVYAALQRQLLSPGTALILLEA

Uniprot       QAASGFLLDPVRNRRLTVNEAVKEGVVGPELHHKLLSAERAVTGYKDPYTGQQISLFQAMQKGLIVREHGIRLLEAQIATGGVIDPVHSHRVPVDVAYRR
Splooce       QAASGFLLDPVRNRRLTVNEAVKEGVVGPELHHKLLSAERAVTGYKDPYTGQQISLFQAMQKGLIVREHGIRLLEAQIATGGVIDPVHSHRVPVDVAYRR

Uniprot       GYFDEEMNRVLADPSDDTKGFFDPNTHENLTYLQLLERCVEDPETGLCLLPLTDKAAKGGELVYTDSEARDVFEKATVSAPFGKFQGKTVTIWEIINSEY
Splooce       GYFDEEMNRVLADPSDDTKGFFDPNTHENLTYLQLLERCVEDPETGLCLLPLTDKAAKGGELVYTDSEARDVFEKATVSAPFGKFQGKTVTIWEIINSEY

Uniprot       FTAEQRRDLLRQFRTGRITVEKIIKIIITVVEEQEQKGRLCFEGLRSLVPAAELLESRVIDRELYQQLQRGERSVRDVAEVDTVRRALRGANVIAGVWLE
Splooce       FTAEQRRDLLRQFRTGRITVEKIIKIIITVVEEQEQKGRLCFEGLRSLVPAAELLESRVIDRELYQQLQRGERSVRDVAEVDTVRRALRGANVIAGVWLE

Uniprot       EAGQKLSIYNALKKDLLPSDMAVALLEAQAGTGHIIDPATSARLTVDEAVRAGLVGPEFHEKLLSAEKAVTGYRDPYTGQSVSLFQALKKGLIPREQGLR
Splooce       EAGQKLSIYNALKKDLLPSDMAVALLEAQAGTGHIIDPATSARLTVDEAVRAGLVGPEFHEKLLSAEKAVTGYRDPYTGQSVSLFQALKKGLIPREQGLR

Uniprot       LLDAQLSTGGIVDPSKSHRVPLDVACARGCLDEETSRALSAPRADAKAYSDPSTGEPATYGELQQRCRPDQLTGLSLLPLSEKAARARQEELYSELQARE
Splooce       LLDAQLSTGGIVDPSKSHRVPLDVACARGCLDEETSRALSAPRADAKAYSDPSTGEPATYGELQQRCRPDQLTGLSLLPLSEKAARARQEELYSELQARE

Uniprot       TFEKTPVEVPVGGFKGRTVTVWELISSEYFTAEQRQELLRQFRTGKVTVEKVIKILITIVEEVETLRQERLSFSGLRAPVPASELLASGVLSRAQFEQLK
Splooce       TFEKTPVEVPVGGFKGRTVTVWELISSEYFTAEQRQELLRQFRTGKVTVEKVIKILITIVEEVETLRQERLSFSGLRAPVPASELLASGVLSRAQFEQLK

Uniprot       DGKTTVKDLSELGSVRTLLQGSGCLAGIYLEDTKEKVSIYEAMRRGLLRATTAALLLEAQAATGFLVDPVRNQRLYVHEAVKAGVVGPELHEQLLSAEKA
Splooce       DGKTTVKDLSELGSVRTLLQGSGCLAGIYLEDTKEKVSIYEAMRRGLLRATTAALLLEAQAATGFLVDPVRNQRLYVHEAVKAGVVGPELHEQLLSAEKA

Uniprot       VTGYRDPYSGSTISLFQAMQKGLVLRQHGIRLLEAQIATGGIIDPVHSHRVPVDVAYQRGYFSEEMNRVLADPSDDTKGFFDPNTHENLTYRQLLERCVE
Splooce       VTGYRDPYSGSTISLFQAMQKGLVLRQHGIRLLEAQIATGGIIDPVHSHRVPVDVAYQRGYFSEEMNRVLADPSDDTKGFFDPNTHENLTYRQLLERCVE

Uniprot       DPETGLRLLPLKGAEKAEVVETTQVYTEEETRRAFEETQIDIPGGGSHGGSTMSLWEVMQSDLIPEEQRAQLMADFQAGRVTKERMIIIIIEIIEKTEII
Splooce       DPETGLRLLPLKGAEKAEVVETTQVYTEEETRRAFEETQIDIPGGGSHGGSTMSLWEVMQSDLIPEEQRAQLMADFQAGRVTKERMIIIIIEIIEKTEII

Uniprot       RQQGLASYDYVRRRLTAEDLFEARIISLETYNLLREGTRSLREALEAESAWCYLYGTGSVAGVYLPGSRQTLSIYQALKKGLLSAEVARLLLEAQAATGF
Splooce       RQQGLASYDYVRRRLTAEDLFEARIISLETYNLLREGTRSLREALEAESAWCYLYGTGSVAGVYLPGSRQTLSIYQALKKGLLSAEVARLLLEAQAATGF

Uniprot       LLDPVKGERLTVDEAVRKGLVGPELHDRLLSAERAVTGYRDPYTEQTISLFQAMKKELIPTEEALRLLDAQLATGGIVDPRLGFHLPLEVAYQRGYLNKD
Splooce       LLDPVKGERLTVDEAVRKGLVGPELHDRLLSAERAVTGYRDPYTEQTISLFQAMKKELIPTEEALRLLDAQLATGGIVDPRLGFHLPLEVAYQRGYLNKD

Uniprot       THDQLSEPSEVRSYVDPSTDERLSYTQLLRRCRRDDGTGQLLLPLSDARKLTFRGLRKQITMEELVRSQVMDEATALQLREGLTSIEEVTKNLQKFLEGT
Splooce       THDQLSEPSEVRSYVDPSTDERLSYTQLLRRCRRDDGTGQLLLPLSDARKLTFRGLRKQITMEELVRSQVMDEATALQLREGLTSIEEVTKNLQKFLEGT

Uniprot       SCIAGVFVDATKERLSVYQAMKKGIIRPGTAFELLEAQAATGYVIDPIKGLKLTVEEAVRMGIVGPEFKDKLLSAERAVTGYKDPYSGKLISLFQAMKKG
Splooce       SCIAGVFVDATKERLSVYQAMKKGIIRPGTAFELLEAQAATGYVIDPIKGLKLTVEEAVRMGIVGPEFKDKLLSAERAVTGYKDPYSGKLISLFQAMKKG

Uniprot       LILKDHGIRLLEAQIATGGIIDPEESHRLPVEVAYKRGLFDEEMNEILTDPSDDTKGFFDPNTEENLTYLQLMERCITDPQTGLCLLPLKEKKRERKTSS
Splooce       LILKDHGIRLLEAQIATGGIIDPEESHRLPVEVAYKRGLFDEEMNEILTDPSDDTKGFFDPNTEENLTYLQLMERCITDPQTGLCLLPLKEKKRERKTSS

Uniprot       KSSVRKRRVVIVDPETGKEMSVYEAYRKGLIDHQTYLELSEQECEWEEITISSSDGVVKSMIIDRRSGRQYDIDDAIAKNLIDRSALDQYRAGTLSITEF
Splooce       KSSVRKRRVVIVDPETGKEMSVYEAYRKGLIDHQTYLELSEQECEWEEITISSSDGVVKSMIIDRRSGRQYDIDDAIAKNLIDRSALDQYRAGTLSITEF

Uniprot       ADMLSGNAGGFRSRSSSVGSSSSYPISPAVSRTQLASWSDPTEETGPVAGILDTETLEKVSITEAMHRNLVDNITGQRLLEAQACTGGIIDPSTGERFPV
Splooce       ADMLSGNAGGFRSRSSSVGSSSSYPISPAVSRTQLASWSDPTEETGPVAGILDTETLEKVSITEAMHRNLVDNITGQRLLEAQACTGGIIDPSTGERFPV

Uniprot       TDAVNKGLVDKIMVDRINLAQKAFCGFEDPRTKTKMSAAQALKKGWLYYEAGQRFLEVQYLTGGLIEPDTPGRVPLDEALQRGTVDARTAQKLRDVGAYS
Splooce       TDAVNKGLVDKIMVDRINLAQKAFCGFEDPRTKTKMSAAQALKKGWLYYEAGQRFLEVQYLTGGLIEPDTPGRVPLDEALQRGTVDARTAQKLRDVGAYS

Uniprot       KYLTCPKTKLKISYKDALDRSMVEEGTGLRLLEAAAQSTKGYYSPYSVSGSGSTAGSRTGSRTGSRAGSRRGSFDATGSGFSMTFSSSSYSSSGYGRRYA
Splooce       KYLTCPKTKLKISYKDALDRSMVEEGTGLRLLEAAAQSTKGYYSPYSVSGSGSTAGSRTGSRTGSRAGSRRGSFDATGSGFSMTFSSSSYSSSGYGRRYA

Uniprot       SGSSASLGGPESAVA
Splooce       SGSSASLGGPESAVA

----------------------------------------------------------------------------------------------------

Q9Y2A7 (Uniprot)	versus
NM_205842#(-s-s-s-:2_N5436505182917) (Splooce)

For more details about the Alternative Splicing Event -> Link to Splooce page

Peptides that support the ASE (Splooce-specific):
MFLDEMAK (MAXQUANT)

Alignment:
Uniprot       MSRSVLQPSQQKLAEKLTILNDRGVGMLTRLYNIKKQGQVWKACGDPKAKPSYLIDKNLESAVKFIVRKFPAVETRNNNQQLAQLQKEKSEILKNLALYY
Splooce       ----------------------------------------------------------------------------------------------------

Uniprot       FTFVDVMEFKDHVCELLNTIDVCQVFFDITVNFDLTKNYLDLIITYTTLMILLSRIEERKAIIGLYNYAHEMTHGASDREYPRLGQMIVDYENPLKKMME
Splooce       ----------------------------------------------------------------------------------------------------

Uniprot       EFVPHSKSLSDALISLQMVYPRRNLSADQWRNAQLLSLISAPSTMLNPAQSDTMPCEYLSLDAMEKWIIFGFILCHGILNTDATALNLWKLALQSSSCLS
Splooce       ----------------------------------------------------------------------------------------------------

Uniprot       LFRDEVFHIHKAAEDLFVNIRGYNKRINDIRECKEAAVSHAGSMHRERRKFLRSALKELATVLSDQPGLLGPKALFVFMALSFARDEIIWLLRHADNMPK
Splooce       ----------------------------------------------------------------------------------------------------

Uniprot       KSADDFIDKHIAELIFYMEELRAHVRKYGPVMQRYYVQYLSGFDAVVLNELVQNLSVCPEDESIIMSSFVNTMTSLSVKQVEDGEVFDFRGMRLDWFRLQ
Splooce       ----------------------------------------------------------------------------------------------------

Uniprot       AYTSVSKASLGLADHRELGKMMNTIIFHTKMVDSLVEMLVETSDLSIFCFYSRAFEKMFQQCLELPSQSRYSIAFPLLCTHFMSCTHELCPEERHHIGDR
Splooce       ----------------------------------------------------------------------------------------------------

Uniprot       SLSLCNMFLDEMAKQARNLITDICTEQCTLSDQLLPKHCAKTISQAVNKKSKKQTGKKGEPEREKPGVESMRKNRLVVTNLDKLHTALSELCFSINYVPN
Splooce       ------MFLDEMAKQARNLITDICTEQCTLSDQLLPKHCAKTISQAVNKKSKKQTGKKGEPEREKPGVESMRKNRLVVTNLDKLHTALSELCFSINYVPN

Uniprot       MVVWEHTFTPREYLTSHLEIRFTKSIVGMTMYNQATQEIAKPSELLTSVRAYMTVLQSIENYVQIDITRVFNNVLLQQTQHLDSHGEPTITSLYTNWYLE
Splooce       MVVWEHTFTPREYLTSHLEIRFTKSIVGMTMYNQATQEIAKPSELLTSVRAYMTVLQSIENYVQIDITRVFNNVLLQQTQHLDSHGEPTITSLYTNWYLE

Uniprot       TLLRQVSNGHIAYFPAMKAFVNLPTENELTFNAEEYSDISEMRSLSELLGPYGMKFLSESLMWHISSQVAELKKLVVENVDVLTQMRTSFDKPDQMAALF
Splooce       TLLRQVSNGHIAYFPAMKAFVNLPTENELTFNAEEYSDISEMRSLSELLGPYGMKFLSESLMWHISSQVAELKKLVVENVDVLTQMRTSFDKPDQMAALF

Uniprot       KRLSSVDSVLKRMTIIGVILSFRSLAQEALRDVLSYHIPFLVSSIEDFKDHIPRETDMKVAMNVYELSSAAGLPCEIDPALVVALSSQKSENISPEEEYK
Splooce       KRLSSVDSVLKRMTIIGVILSFRSLAQEALRDVLSYHIPFLVSSIEDFKDHIPRETDMKVAMNVYELSSAAGLPCEIDPALVVALSSQKSENISPEEEYK

Uniprot       IACLLMVFVAVSLPTLASNVMSQYSPAIEGHCNNIHCLAKAINQIAAALFTIHKGSIEDRLKEFLALASSSLLKIGQETDKTTTRNRESVYLLLDMIVQE
Splooce       IACLLMVFVAVSLPTLASNVMSQYSPAIEGHCNNIHCLAKAINQIAAALFTIHKGSIEDRLKEFLALASSSLLKIGQETDKTTTRNRESVYLLLDMIVQE

Uniprot       SPFLTMDLLESCFPYVLLRNAYHAVYKQSVTSSA
Splooce       SPFLTMDLLESCFPYVLLRNAYHAVYKQSVTSSA

----------------------------------------------------------------------------------------------------

P11766 (Uniprot)	versus
NM_000671#(-t:4_A3856934419591) (Splooce)

For more details about the Alternative Splicing Event -> Link to Splooce page

Peptides that support the ASE (Splooce-specific):
MGTSTFSEYTVVADISVAK (MAXQUANT)

Alignment:
Uniprot       MANEVIKCKAAVAWEAGKPLSIEEIEVAPPKAHEVRIKIIATAVCHTDAYTLSGADPEGCFPVILGHEGAGIVESVGEGVTKLKAGDTVIPLYIPQCGEC
Splooce       ----------------------------------------------------------------------------------------------------

Uniprot       KFCLNPKTNLCQKIRVTQGKGLMPDGTSRFTCKGKTILHYMGTSTFSEYTVVADISVAKIDPLAPLDKVCLLGCGISTGYGAAVNTAKLEPGSVCAVFGL
Splooce       ----------------------------------------MGTSTFSEYTVVADISVAKIDPLAPLDKVCLLGCGISTGYGAAVNTAKLEPGSVCAVFGL

Uniprot       GGVGLAVIMGCKVAGASRIIGVDINKDKFARAKEFGATECINPQDFSKPIQEVLIEMTDGGVDYSFECIGNVKVMRAALEACHKGWGVSVVVGVAASGEE
Splooce       GGVGLAVIMGCKVAGASRIIGVDINKDKFARAKEFGATECINPQDFSKPIQEVLIEMTDGGVDYSFECIGNVKVMRAALEACHKGWGVSVVVGVAASGEE

Uniprot       IATRPFQLVTGRTWKGTAFGGWKSVESVPKLVSEYMSKKIKVDEFVTHNLSFDEINKAFELMHSGKSIRTVVKI
Splooce       IATRPFQLVTGRTWKGTAFGGWKSVESVPKLVSEYMSKKIKVDEFVTHNLSFDEINKAFELMHSGKSIRTVVKI

----------------------------------------------------------------------------------------------------

P14618 (Uniprot)	versus
NM_182470#(-t:15_P6760853117556) (Splooce)

For more details about the Alternative Splicing Event -> Link to Splooce page

Peptides that support the ASE (Splooce-specific):
ADTFLEHMCR (PEAKS)
MADTFLEHMCR (PEAKS)

Alignment:
Uniprot       MSKPHSEAGTAFIQTQQLHAAMADTFLEHMCRLDIDSPPITARNTGIICTIGPASRSVETLKEMIKSGMNVARLNFSHGTHEYHAETIKNVRTATESFAS
Splooce       ---------------------MADTFLEHMCRLDIDSPPITARNTGIICTIGPASRSVETLKEMIKSGMNVARLNFSHGTHEYHAETIKNVRTATESFAS

Uniprot       DPILYRPVAVALDTKGPEIRTGLIKGSGTAEVELKKGATLKITLDNAYMEKCDENILWLDYKNICKVVEVGSKIYVDDGLISLQVKQKGADFLVTEVENG
Splooce       DPILYRPVAVALDTKGPEIRTGLIKGSGTAEVELKKGATLKITLDNAYMEKCDENILWLDYKNICKVVEVGSKIYVDDGLISLQVKQKGADFLVTEVENG

Uniprot       GSLGSKKGVNLPGAAVDLPAVSEKDIQDLKFGVEQDVDMVFASFIRKASDVHEVRKVLGEKGKNIKIISKIENHEGVRRFDEILEASDGIMVARGDLGIE
Splooce       GSLGSKKGVNLPGAAVDLPAVSEKDIQDLKFGVEQDVDMVFASFIRKASDVHEVRKVLGEKGKNIKIISKIENHEGVRRFDEILEASDGIMVARGDLGIE

Uniprot       IPAEKVFLAQKMMIGRCNRAGKPVICATQMLESMIKKPRPTRAEGSDVANAVLDGADCIMLSGETAKGDYPLEAVRMQHLIAREAEAAMFHRKLFEELVR
Splooce       IPAEKVFLAQKMMIGRCNRAGKPVICATQMLESMIKKPRPTRAEGSDVANAVLDGADCIMLSGETAKGDYPLEAVRMQHLIAREAEAAMFHRKLFEELVR

Uniprot       ASSHSTDLMEAMAMGSVEASYKCLAAALIVLTESGRSAHQVARYRPRAPIIAVTRNPQTARQAHLYRGIFPVLCKDPVQEAWAEDVDLRVNFAMNVGKAR
Splooce       ASSHSTDLMEAMAMGSVEASYKCLAAALIVLTESGRSAHQVARYRPRAPIIAVTRNPQTARQAHLYRGIFPVLCKDPVQEAWAEDVDLRVNFAMNVGKAR

Uniprot       GFFKKGDVVIVLTGWRPGSGFTNTMRVVPVP
Splooce       GFFKKGDVVIVLTGWRPGSGFTNTMRVVPVP

----------------------------------------------------------------------------------------------------

O75533 (Uniprot)	versus
NM_012433#(-s-s-s-:2_S8934413179831) (Splooce)

For more details about the Alternative Splicing Event -> Link to Splooce page

Peptides that support the ASE (Splooce-specific):
SMTPEQLQAWR (MAXQUANT)

Alignment:
Uniprot       MAKIAKTHEDIEAQIREIQGKKAALDEAQGVGLDSTGYYDQEIYGGSDSRFAGYVTSIAATELEDDDDDYSSSTSLLGQKKPGYHAPVALLNDIPQSTEQ
Splooce       ----------------------------------------------------------------------------------------------------

Uniprot       YDPFAEHRPPKIADREDEYKKHRRTMIISPERLDPFADGGKTPDPKMNARTYMDVMREQHLTKEEREIRQQLAEKAKAGELKVVNGAAASQPPSKRKRRW
Splooce       ----------------------------------------------------------------------------------------------------

Uniprot       DQTADQTPGATPKKLSSWDQAETPGHTPSLRWDETPGRAKGSETPGATPGSKIWDPTPSHTPAGAATPGRGDTPGHATPGHGGATSSARKNRWDETPKTE
Splooce       ----------------------------------------------------------------------------------------------------

Uniprot       RDTPGHGSGWAETPRTDRGGDSIGETPTPGASKRKSRWDETPASQMGGSTPVLTPGKTPIGTPAMNMATPTPGHIMSMTPEQLQAWRWEREIDERNRPLS
Splooce       ---------------------------------------------------------------------------MSMTPEQLQAWRWEREIDERNRPLS

Uniprot       DEELDAMFPEGYKVLPPPAGYVPIRTPARKLTATPTPLGGMTGFHMQTEDRTMKSVNDQPSGNLPFLKPDDIQYFDKLLVDVDESTLSPEEQKERKIMKL
Splooce       DEELDAMFPEGYKVLPPPAGYVPIRTPARKLTATPTPLGGMTGFHMQTEDRTMKSVNDQPSGNLPFLKPDDIQYFDKLLVDVDESTLSPEEQKERKIMKL

Uniprot       LLKIKNGTPPMRKAALRQITDKAREFGAGPLFNQILPLLMSPTLEDQERHLLVKVIDRILYKLDDLVRPYVHKILVVIEPLLIDEDYYARVEGREIISNL
Splooce       LLKIKNGTPPMRKAALRQITDKAREFGAGPLFNQILPLLMSPTLEDQERHLLVKVIDRILYKLDDLVRPYVHKILVVIEPLLIDEDYYARVEGREIISNL

Uniprot       AKAAGLATMISTMRPDIDNMDEYVRNTTARAFAVVASALGIPSLLPFLKAVCKSKKSWQARHTGIKIVQQIAILMGCAILPHLRSLVEIIEHGLVDEQQK
Splooce       AKAAGLATMISTMRPDIDNMDEYVRNTTARAFAVVASALGIPSLLPFLKAVCKSKKSWQARHTGIKIVQQIAILMGCAILPHLRSLVEIIEHGLVDEQQK

Uniprot       VRTISALAIAALAEAATPYGIESFDSVLKPLWKGIRQHRGKGLAAFLKAIGYLIPLMDAEYANYYTREVMLILIREFQSPDEEMKKIVLKVVKQCCGTDG
Splooce       VRTISALAIAALAEAATPYGIESFDSVLKPLWKGIRQHRGKGLAAFLKAIGYLIPLMDAEYANYYTREVMLILIREFQSPDEEMKKIVLKVVKQCCGTDG

Uniprot       VEANYIKTEILPPFFKHFWQHRMALDRRNYRQLVDTTVELANKVGAAEIISRIVDDLKDEAEQYRKMVMETIEKIMGNLGAADIDHKLEEQLIDGILYAF
Splooce       VEANYIKTEILPPFFKHFWQHRMALDRRNYRQLVDTTVELANKVGAAEIISRIVDDLKDEAEQYRKMVMETIEKIMGNLGAADIDHKLEEQLIDGILYAF

Uniprot       QEQTTEDSVMLNGFGTVVNALGKRVKPYLPQICGTVLWRLNNKSAKVRQQAADLISRTAVVMKTCQEEKLMGHLGVVLYEYLGEEYPEVLGSILGALKAI
Splooce       QEQTTEDSVMLNGFGTVVNALGKRVKPYLPQICGTVLWRLNNKSAKVRQQAADLISRTAVVMKTCQEEKLMGHLGVVLYEYLGEEYPEVLGSILGALKAI

Uniprot       VNVIGMHKMTPPIKDLLPRLTPILKNRHEKVQENCIDLVGRIADRGAEYVSAREWMRICFELLELLKAHKKAIRRATVNTFGYIAKAIGPHDVLATLLNN
Splooce       VNVIGMHKMTPPIKDLLPRLTPILKNRHEKVQENCIDLVGRIADRGAEYVSAREWMRICFELLELLKAHKKAIRRATVNTFGYIAKAIGPHDVLATLLNN

Uniprot       LKVQERQNRVCTTVAIAIVAETCSPFTVLPALMNEYRVPELNVQNGVLKSLSFLFEYIGEMGKDYIYAVTPLLEDALMDRDLVHRQTASAVVQHMSLGVY
Splooce       LKVQERQNRVCTTVAIAIVAETCSPFTVLPALMNEYRVPELNVQNGVLKSLSFLFEYIGEMGKDYIYAVTPLLEDALMDRDLVHRQTASAVVQHMSLGVY

Uniprot       GFGCEDSLNHLLNYVWPNVFETSPHVIQAVMGALEGLRVAIGPCRMLQYCLQGLFHPARKVRDVYWKIYNSIYIGSQDALIAHYPRIYNDDKNTYIRYEL
Splooce       GFGCEDSLNHLLNYVWPNVFETSPHVIQAVMGALEGLRVAIGPCRMLQYCLQGLFHPARKVRDVYWKIYNSIYIGSQDALIAHYPRIYNDDKNTYIRYEL

Uniprot       DYIL
Splooce       DYIL

----------------------------------------------------------------------------------------------------

E7EUT4 (Uniprot)	versus
NM_002046#(f-t:12_G5852279946153) (Splooce)

For more details about the Alternative Splicing Event -> Link to Splooce page

Peptides that support the ASE (Splooce-specific):
MTTVHALTATQK (PEAKS)
MTTVHAITATQK (MAXQUANT)
TTVHAITATQK (MAXQUANT)
TTVHALTATQK (PEAKS)

Alignment:
Uniprot       MGKVKVGVNGFGRIGRLVTRAAFNSGKVDIVAINDPFIDLNYMVYMFQYDSTHGKFHGTVKAENGKLVINGNPITIFQERDPSKIKWGDAGAEYVVESTG
Splooce       ----------------------------------------------------------------------------------------------------

Uniprot       VFTTMEKAGAHLQGGAKRVIISAPSADAPMFVMGVNHEKYDNSLKIISNASCTTNCLAPLAKVIHDNFGIVEGLMTTVHAITATQKTVDGPSGKLWRDGR
Splooce       --------------------------------------------------------------------------MTTVHAITATQKTVDGPSGKLWRDGR

Uniprot       GALQNIIPASTGAAKAVGKVIPELNGKLTGMAFRVPTANVSVVDLTCRLEKPAKYDDIKKVVKQASEGPLKGILGYTEHQVVSSDFNSDTHSSTFDAGAG
Splooce       GALQNIIPASTGAAKAVGKVIPELNGKLTGMAFRVPTANVSVVDLTCRLEKPAKYDDIKKVVKQASEGPLKGILGYTEHQVVSSDFNSDTHSSTFDAGAG

Uniprot       IALNDHFVKLISWYDNEFGYSNRVVDLMAHMASKE
Splooce       IALNDHFVKLISWYDNEFGYSNRVVDLMAHMASKE

----------------------------------------------------------------------------------------------------

Q99798 (Uniprot)	versus
NM_001098#(-t-e-e-f-:22_A358723049025) (Splooce)

For more details about the Alternative Splicing Event -> Link to Splooce page

Peptides that support the ASE (Splooce-specific):
SHFEPNEYIHYDLLEK (MAXQUANT)
SHFEPNEYLHYDLLEK (PEAKS)
MSHFEPNEYIHYDLLEK (MAXQUANT)

Alignment:
Uniprot       MAPYSLLVTRLQKALGVRQYHVASVLCQRAKVAMSHFEPNEYIHYDLLEKNINIVRKRLNRPLTLSEKIVYGHLDDPASQEIERGKSYLRLRPDRVAMQD
Splooce       ---------------------------------MSHFEPNEYIHYDLLEKNINIVRKRLNRPLTLSEKIVYGHLDDPASQEIERGKSYLRLRPDRVAMQD

Uniprot       ATAQMAMLQFISSGLSKVAVPSTIHCDHLIEAQVGGEKDLRRAKDINQEVYNFLATAGAKYGVGFWKPGSGIIHQIILENYAYPGVLLIGTDSHTPNGGG
Splooce       ATAQMAMLQFISSGLSKVAVPSTIHCDHLIEAQVGGEKDLRRAKDINQEVYNFLATAGAKYGVGFWKPGSGIIHQIILENYAYPGVLLIGTDSHTPNGGG

Uniprot       LGGICIGVGGADAVDVMAGIPWELKCPKV--IGVKLTGSLSGWSSPKDVILKVAGILTVKGGTGAIVEYHGPGVDSISCTGMATICNMGAEIGATTSVFP
Splooce       LGGICIGVGGADAVDVMAGIPWELKCPKVRVIGVKLTGSLSGWSSPKDVILKVAGILTVKGGTGAIVEYHGPGVDSISCTGMATICNMGAEIGATTSVFP

Uniprot       YNHRMKKYLSKTGREDIANLADEFKDHLVPDPGCHYDQLIEINLSELKPHINGPFTPDLAHPVAEVGKVAEKEGWPLDIRVGLIGSCTNSSYEDMGRSAA
Splooce       YNHRMKKYLSKTGREDIANLADEFKDHLVPDPGCHYDQLIEINLSELKPHINGPFTPDLAHPVAEVGKVAEKEGWPLDIRVGLIGSCTNSSYEDMGRSAA

Uniprot       VAKQALAHGLKCKSQFTITPGSEQIRATIERDGYAQILRDLGGIVLANACGPCIGQWDRKDIKKGEKNTIVTSYNRNFTGRNDANPETHAFVTSPEIVTA
Splooce       VAKQALAHGLKCKSQFTITPGSEQIRATIERDGYAQILRDLGGIVLANACGPCIGQWDRKDIKKGEKNTIVTSYNRNFTGRNDANPETHAFVTSPEIVTA

Uniprot       LAIAGTLKFNPETDYLTGTDGKKFRLEAPDADELPKGEFDPGQDTYQHPPKDSSGQHVDVSPTSQRLQLLEPFDKWDGKDLEDLQILIKVKGKCTTDHIS
Splooce       LAIAGTLKFNPETDYLTGTDGKKFRLEAPDADELPKGEFDPGQDTYQHPPKDSSGQHVDVSPTSQRLQLLEPFDKWDGKDLEDLQILIKVKGKCTTDHIS

Uniprot       AAGPWLKFRGHLDNISNNLLIGAINIENGKANSVRNAVTQEFGPVPDTARYYKKHGIRWVVIGDENYGEGSSREHAALEPRHLGGRAIITKSFARIHETN
Splooce       AAGPWLKFRGHLDNISNNLLIGAINIENGKANSVRNAVTQEFGPVPDTARYYKKHGIRWVVIGDENYGEGSSREHAALEPRHLGGRAIITKSFARIHETN

Uniprot       LKKQGLLPLTFADPADYNKIHPVDKLTIQGLKDFTPGKPLKCIIKHPNGTQETILLNHTFNETQIEWFRAGSALNRMKELQQ
Splooce       LKKQGLLPLTFADPADYNKIHPVDKLTIQGLKDFTPGKPLKCIIKHPNGTQETILLNHTFNETQIEWFRAGSALNRMKELQQ

----------------------------------------------------------------------------------------------------

P22234 (Uniprot)	versus
NM_006452#(-s-s-:4_P4010305192004) (Splooce)

For more details about the Alternative Splicing Event -> Link to Splooce page

Peptides that support the ASE (Splooce-specific):
SHATQAIFEILEK (MAXQUANT)

Alignment:
Uniprot       MLPPGRQDLSSASLPGAVAALSPLRIMATAEVLNIGKKLYEGKTKEVYELLDSPGKVLLQSKDQITAGNAARKNHLEGKAAISNKITSCIFQLLQEAGIK
Splooce       ----------------------------------------------------------------------------------------------------

Uniprot       TAFTRKCGETAFIAPQCEMIPIEWVCRRIATGSFLKRNPGVKEGYKFYPPKVELFFKDDANNDPQWSEEQLIAAKFCFAGLLIGQTEVDIMSHATQAIFE
Splooce       ------------------------------------------------------------------------------------------MSHATQAIFE

Uniprot       ILEKSWLPQNCTLVDMKIEFGVDVTTKEIVLADVIDNDSWRLWPSGDRSQQKDKQSYRDLKEVTPEGLQMVKKNFEWVAERVELLLKSESQCRVVVLMGS
Splooce       ILEKSWLPQNCTLVDMKIEFGVDVTTKEIVLADVIDNDSWRLWPSGDRSQQKDKQSYRDLKEVTPEGLQMVKKNFEWVAERVELLLKSESQCRVVVLMGS

Uniprot       TSDLGHCEKIKKACGNFGIPCELRVTSAHKGPDETLRIKAEYEGDGIPTVFVAVAGRSNGLGPVMSGNTAYPVISCPPLTPDWGVQDVWSSLRLPSGLGC
Splooce       TSDLGHCEKIKKACGNFGIPCELRVTSAHKGPDETLRIKAEYEGDGIPTVFVAVAGRSNGLGPVMSGNTAYPVISCPPLTPDWGVQDVWSSLRLPSGLGC

Uniprot       STVLSPEGSAQFAAQIFGLSNHLVWSKLRASILNTWISLKQADKKIRECNL
Splooce       STVLSPEGSAQFAAQIFGLSNHLVWSKLRASILNTWISLKQADKKIRECNL

----------------------------------------------------------------------------------------------------

P22314 (Uniprot)	versus
NM_153280#(-s-s-s-s-s-s-s-s-s-s-s-:X_U3769449095174) (Splooce)

For more details about the Alternative Splicing Event -> Link to Splooce page

Peptides that support the ASE (Splooce-specific):
IGLGCGEGGEIIVTDMDTIEK (MAXQUANT)

Alignment:
Uniprot       MSSSPLSKKRRVSGPDPKPGSNCSPAQSVLSEVPSVPTNGMAKNGSEADIDEGLYSRQLYVLGHEAMKRLQTSSVLVSGLRGLGVEIAKNIILGGVKAVT
Splooce       ----------------------------------------------------------------------------------------------------

Uniprot       LHDQGTAQWADLSSQFYLREEDIGKNRAEVSQPRLAELNSYVPVTAYTGPLVEDFLSGFQVVVLTNTPLEDQLRVGEFCHNRGIKLVVADTRGLFGQLFC
Splooce       ----------------------------------------------------------------------------------------------------

Uniprot       DFGEEMILTDSNGEQPLSAMVSMVTKDNPGVVTCLDEARHGFESGDFVSFSEVQGMVELNGNQPMEIKVLGPYTFSICDTSNFSDYIRGGIVSQVKVPKK
Splooce       ----------------------------------------------------------------------------------------------------

Uniprot       ISFKSLVASLAEPDFVVTDFAKFSRPAQLHIGFQALHQFCAQHGRPPRPRNEEDAAELVALAQAVNARALPAVQQNNLDEDLIRKLAYVAAGDLAPINAF
Splooce       ----------------------------------------------------------------------------------------------------

Uniprot       IGGLAAQEVMKACSGKFMPIMQWLYFDALECLPEDKEVLTEDKCLQRQNRYDGQVAVFGSDLQEKLGKQKYFLVGAGAIGCELLKNFAMIGLGCGEGGEI
Splooce       ----------------------------------------------------------------------------------------MIGLGCGEGGEI

Uniprot       IVTDMDTIEKSNLNRQFLFRPWDVTKLKSDTAAAAVRQMNPHIRVTSHQNRVGPDTERIYDDDFFQNLDGVANALDNVDARMYMDRRCVYYRKPLLESGT
Splooce       IVTDMDTIEKSNLNRQFLFRPWDVTKLKSDTAAAAVRQMNPHIRVTSHQNRVGPDTERIYDDDFFQNLDGVANALDNVDARMYMDRRCVYYRKPLLESGT

Uniprot       LGTKGNVQVVIPFLTESYSSSQDPPEKSIPICTLKNFPNAIEHTLQWARDEFEGLFKQPAENVNQYLTDPKFVERTLRLAGTQPLEVLEAVQRSLVLQRP
Splooce       LGTKGNVQVVIPFLTESYSSSQDPPEKSIPICTLKNFPNAIEHTLQWARDEFEGLFKQPAENVNQYLTDPKFVERTLRLAGTQPLEVLEAVQRSLVLQRP

Uniprot       QTWADCVTWACHHWHTQYSNNIRQLLHNFPPDQLTSSGAPFWSGPKRCPHPLTFDVNNPLHLDYVMAAANLFAQTYGLTGSQDRAAVATFLQSVQVPEFT
Splooce       QTWADCVTWACHHWHTQYSNNIRQLLHNFPPDQLTSSGAPFWSGPKRCPHPLTFDVNNPLHLDYVMAAANLFAQTYGLTGSQDRAAVATFLQSVQVPEFT

Uniprot       PKSGVKIHVSDQELQSANASVDDSRLEELKATLPSPDKLPGFKMYPIDFEKDDDSNFHMDFIVAASNLRAENYDIPSADRHKSKLIAGKIIPAIATTTAA
Splooce       PKSGVKIHVSDQELQSANASVDDSRLEELKATLPSPDKLPGFKMYPIDFEKDDDSNFHMDFIVAASNLRAENYDIPSADRHKSKLIAGKIIPAIATTTAA

Uniprot       VVGLVCLELYKVVQGHRQLDSYKNGFLNLALPFFGFSEPLAAPRHQYYNQEWTLWDRFEVQGLQPNGEEMTLKQFLDYFKTEHKLEITMLSQGVSMLYSF
Splooce       VVGLVCLELYKVVQGHRQLDSYKNGFLNLALPFFGFSEPLAAPRHQYYNQEWTLWDRFEVQGLQPNGEEMTLKQFLDYFKTEHKLEITMLSQGVSMLYSF

Uniprot       FMPAAKLKERLDQPMTEIVSRVSKRKLGRHVRALVLELCCNDESGEDVEVPYVRYTIR
Splooce       FMPAAKLKERLDQPMTEIVSRVSKRKLGRHVRALVLELCCNDESGEDVEVPYVRYTIR

----------------------------------------------------------------------------------------------------

Q8TB37 (Uniprot)	versus
NM_025152#(-t:14_N1546243757449) (Splooce)

For more details about the Alternative Splicing Event -> Link to Splooce page

Peptides that support the ASE (Splooce-specific):
SMGFLVEESEPVVWR (MAXQUANT)

Alignment:
Uniprot       MGIWQRLLLFGGVSLRAGGGATAPLGGSRAMVCGRQLSGAGSETLKQRRTQIMSRGLPKQKPIEGVKQVIVVASGKGGVGKSTTAVNLALALAANDSSKA
Splooce       ----------------------------------------------------------------------------------------------------

Uniprot       IGLLDVDVYGPSVPKMMNLKGNPELSQSNLMRPLLNYGIACMSMGFLVEESEPVVWRGLMVMSAIEKLLRQVDWGQLDYLVVDMPPGTGDVQLSVSQNIP
Splooce       -----------------------------------------MSMGFLVEESEPVVWRGLMVMSAIEKLLRQVDWGQLDYLVVDMPPGTGDVQLSVSQNIP

Uniprot       ITGAVIVSTPQDIALMDAHKGAEMFRRVHVPVLGLVQNMSVFQCPKCKHKTHIFGADGARKLAQTLGLEVLGDIPLHLNIREASDTGQPIVFSQPESDEA
Splooce       ITGAVIVSTPQDIALMDAHKGAEMFRRVHVPVLGLVQNMSVFQCPKCKHKTHIFGADGARKLAQTLGLEVLGDIPLHLNIREASDTGQPIVFSQPESDEA

Uniprot       KAYLRIAVEVVRRLPSPSE
Splooce       KAYLRIAVEVVRRLPSPSE

----------------------------------------------------------------------------------------------------

P68104 (Uniprot)	versus
NM_001402#(-s-s-s-s-:6_E5555551751174) (Splooce)

For more details about the Alternative Splicing Event -> Link to Splooce page

Peptides that support the ASE (Splooce-specific):
MVVTFAPVNVTTEVK (MAXQUANT)
VVTFAPVNVTTEVK (MAXQUANT + PEAKS)

Alignment:
Uniprot       MGKEKTHINIVVIGHVDSGKSTTTGHLIYKCGGIDKRTIEKFEKEAAEMGKGSFKYAWVLDKLKAERERGITIDISLWKFETSKYYVTIIDAPGHRDFIK
Splooce       ----------------------------------------------------------------------------------------------------

Uniprot       NMITGTSQADCAVLIVAAGVGEFEAGISKNGQTREHALLAYTLGVKQLIVGVNKMDSTEPPYSQKRYEEIVKEVSTYIKKIGYNPDTVAFVPISGWNGDN
Splooce       ----------------------------------------------------------------------------------------------------

Uniprot       MLEPSANMPWFKGWKVTRKDGNASGTTLLEALDCILPPTRPTDKPLRLPLQDVYKIGGIGTVPVGRVETGVLKPGMVVTFAPVNVTTEVKSVEMHHEALS
Splooce       ---------------------------------------------------------------------------MVVTFAPVNVTTEVKSVEMHHEALS

Uniprot       EALPGDNVGFNVKNVSVKDVRRGNVAGDSKNDPPMEAAGFTAQVIILNHPGQISAGYAPVLDCHTAHIACKFAELKEKIDRRSGKKLEDGPKFLKSGDAA
Splooce       EALPGDNVGFNVKNVSVKDVRRGNVAGDSKNDPPMEAAGFTAQVIILNHPGQISAGYAPVLDCHTAHIACKFAELKEKIDRRSGKKLEDGPKFLKSGDAA

Uniprot       IVDMVPGKPMCVESFSDYPPLGRFAVRDMRQTVAVGVIKAVDKKAAGAGKVTKSAQKAQKAK
Splooce       IVDMVPGKPMCVESFSDYPPLGRFAVRDMRQTVAVGVIKAVDKKAAGAGKVTKSAQKAQKAK

----------------------------------------------------------------------------------------------------

Q9Y490 (Uniprot)	versus
NM_006289#(-s-s-s-s-s-:9_T7364987111340) (Splooce)

For more details about the Alternative Splicing Event -> Link to Splooce page

Peptides that support the ASE (Splooce-specific):
MSDFGDYQDGYYSVQTTEGEQIAQLIAGYIDIILK (MAXQUANT)

Alignment:
Uniprot       MVALSLKISIGNVVKTMQFEPSTMVYDACRIIRERIPEAPAGPPSDFGLFLSDDDPKKGIWLEAGKALDYYMLRNGDTMEYRKKQRPLKIRMLDGTVKTI
Splooce       ----------------------------------------------------------------------------------------------------

Uniprot       MVDDSKTVTDMLMTICARIGITNHDEYSLVRELMEEKKEEITGTLRKDKTLLRDEKKMEKLKQKLHTDDELNWLDHGRTLREQGVEEHETLLLRRKFFYS
Splooce       ----------------------------------------------------------------------------------------------------

Uniprot       DQNVDSRDPVQLNLLYVQARDDILNGSHPVSFDKACEFAGFQCQIQFGPHNEQKHKAGFLDLKDFLPKEYVKQKGERKIFQAHKNCGQMSEIEAKVRYVK
Splooce       ----------------------------------------------------------------------------------------------------

Uniprot       LARSLKTYGVSFFLVKEKMKGKNKLVPRLLGITKECVMRVDEKTKEVIQEWNLTNIKRWAASPKSFTLDFGDYQDGYYSVQTTEGEQIAQLIAGYIDIIL
Splooce       -----------------------------------------------------------------MMSDFGDYQDGYYSVQTTEGEQIAQLIAGYIDIIL

Uniprot       KKKKSKDHFGLEGDEESTMLEDSVSPKKSTVLQQQYNRVGKVEHGSVALPAIMRSGASGPENFQVGSMPPAQQQITSGQMHRGHMPPLTSAQQALTGTIN
Splooce       KKKKSKDHFGLEGDEESTMLEDSVSPKKSTVLQQQYNRVGKVEHGSVALPAIMRSGASGPENFQVGSMPPAQQQITSGQMHRGHMPPLTSAQQALTGTIN

Uniprot       SSMQAVQAAQATLDDFDTLPPLGQDAASKAWRKNKMDESKHEIHSQVDAITAGTASVVNLTAGDPAETDYTAVGCAVTTISSNLTEMSRGVKLLAALLED
Splooce       SSMQAVQAAQATLDDFDTLPPLGQDAASKAWRKNKMDESKHEIHSQVDAITAGTASVVNLTAGDPAETDYTAVGCAVTTISSNLTEMSRGVKLLAALLED

Uniprot       EGGSGRPLLQAAKGLAGAVSELLRSAQPASAEPRQNLLQAAGNVGQASGELLQQIGESDTDPHFQDALMQLAKAVASAAAALVLKAKSVAQRTEDSGLQT
Splooce       EGGSGRPLLQAAKGLAGAVSELLRSAQPASAEPRQNLLQAAGNVGQASGELLQQIGESDTDPHFQDALMQLAKAVASAAAALVLKAKSVAQRTEDSGLQT

Uniprot       QVIAAATQCALSTSQLVACTKVVAPTISSPVCQEQLVEAGRLVAKAVEGCVSASQAATEDGQLLRGVGAAATAVTQALNELLQHVKAHATGAGPAGRYDQ
Splooce       QVIAAATQCALSTSQLVACTKVVAPTISSPVCQEQLVEAGRLVAKAVEGCVSASQAATEDGQLLRGVGAAATAVTQALNELLQHVKAHATGAGPAGRYDQ

Uniprot       ATDTILTVTENIFSSMGDAGEMVRQARILAQATSDLVNAIKADAEGESDLENSRKLLSAAKILADATAKMVEAAKGAAAHPDSEEQQQRLREAAEGLRMA
Splooce       ATDTILTVTENIFSSMGDAGEMVRQARILAQATSDLVNAIKADAEGESDLENSRKLLSAAKILADATAKMVEAAKGAAAHPDSEEQQQRLREAAEGLRMA

Uniprot       TNAAAQNAIKKKLVQRLEHAAKQAAASATQTIAAAQHAASTPKASAGPQPLLVQSCKAVAEQIPLLVQGVRGSQAQPDSPSAQLALIAASQSFLQPGGKM
Splooce       TNAAAQNAIKKKLVQRLEHAAKQAAASATQTIAAAQHAASTPKASAGPQPLLVQSCKAVAEQIPLLVQGVRGSQAQPDSPSAQLALIAASQSFLQPGGKM

Uniprot       VAAAKASVPTIQDQASAMQLSQCAKNLGTALAELRTAAQKAQEACGPLEMDSALSVVQNLEKDLQEVKAAARDGKLKPLPGETMEKCTQDLGNSTKAVSS
Splooce       VAAAKASVPTIQDQASAMQLSQCAKNLGTALAELRTAAQKAQEACGPLEMDSALSVVQNLEKDLQEVKAAARDGKLKPLPGETMEKCTQDLGNSTKAVSS

Uniprot       AIAQLLGEVAQGNENYAGIAARDVAGGLRSLAQAARGVAALTSDPAVQAIVLDTASDVLDKASSLIEEAKKAAGHPGDPESQQRLAQVAKAVTQALNRCV
Splooce       AIAQLLGEVAQGNENYAGIAARDVAGGLRSLAQAARGVAALTSDPAVQAIVLDTASDVLDKASSLIEEAKKAAGHPGDPESQQRLAQVAKAVTQALNRCV

Uniprot       SCLPGQRDVDNALRAVGDASKRLLSDSLPPSTGTFQEAQSRLNEAAAGLNQAATELVQASRGTPQDLARASGRFGQDFSTFLEAGVEMAGQAPSQEDRAQ
Splooce       SCLPGQRDVDNALRAVGDASKRLLSDSLPPSTGTFQEAQSRLNEAAAGLNQAATELVQASRGTPQDLARASGRFGQDFSTFLEAGVEMAGQAPSQEDRAQ

Uniprot       VVSNLKGISMSSSKLLLAAKALSTDPAAPNLKSQLAAAARAVTDSINQLITMCTQQAPGQKECDNALRELETVRELLENPVQPINDMSYFGCLDSVMENS
Splooce       VVSNLKGISMSSSKLLLAAKALSTDPAAPNLKSQLAAAARAVTDSINQLITMCTQQAPGQKECDNALRELETVRELLENPVQPINDMSYFGCLDSVMENS

Uniprot       KVLGEAMTGISQNAKNGNLPEFGDAISTASKALCGFTEAAAQAAYLVGVSDPNSQAGQQGLVEPTQFARANQAIQMACQSLGEPGCTQAQVLSAATIVAK
Splooce       KVLGEAMTGISQNAKNGNLPEFGDAISTASKALCGFTEAAAQAAYLVGVSDPNSQAGQQGLVEPTQFARANQAIQMACQSLGEPGCTQAQVLSAATIVAK

Uniprot       HTSALCNSCRLASARTTNPTAKRQFVQSAKEVANSTANLVKTIKALDGAFTEENRAQCRAATAPLLEAVDNLSAFASNPEFSSIPAQISPEGRAAMEPIV
Splooce       HTSALCNSCRLASARTTNPTAKRQFVQSAKEVANSTANLVKTIKALDGAFTEENRAQCRAATAPLLEAVDNLSAFASNPEFSSIPAQISPEGRAAMEPIV

Uniprot       ISAKTMLESAGGLIQTARALAVNPRDPPSWSVLAGHSRTVSDSIKKLITSMRDKAPGQLECETAIAALNSCLRDLDQASLAAVSQQLAPREGISQEALHT
Splooce       ISAKTMLESAGGLIQTARALAVNPRDPPSWSVLAGHSRTVSDSIKKLITSMRDKAPGQLECETAIAALNSCLRDLDQASLAAVSQQLAPREGISQEALHT

Uniprot       QMLTAVQEISHLIEPLANAARAEASQLGHKVSQMAQYFEPLTLAAVGAASKTLSHPQQMALLDQTKTLAESALQLLYTAKEAGGNPKQAAHTQEALEEAV
Splooce       QMLTAVQEISHLIEPLANAARAEASQLGHKVSQMAQYFEPLTLAAVGAASKTLSHPQQMALLDQTKTLAESALQLLYTAKEAGGNPKQAAHTQEALEEAV

Uniprot       QMMTEAVEDLTTTLNEAASAAGVVGGMVDSITQAINQLDEGPMGEPEGSFVDYQTTMVRTAKAIAVTVQEMVTKSNTSPEELGPLANQLTSDYGRLASEA
Splooce       QMMTEAVEDLTTTLNEAASAAGVVGGMVDSITQAINQLDEGPMGEPEGSFVDYQTTMVRTAKAIAVTVQEMVTKSNTSPEELGPLANQLTSDYGRLASEA

Uniprot       KPAAVAAENEEIGSHIKHRVQELGHGCAALVTKAGALQCSPSDAYTKKELIECARRVSEKVSHVLAALQAGNRGTQACITAASAVSGIIADLDTTIMFAT
Splooce       KPAAVAAENEEIGSHIKHRVQELGHGCAALVTKAGALQCSPSDAYTKKELIECARRVSEKVSHVLAALQAGNRGTQACITAASAVSGIIADLDTTIMFAT

Uniprot       AGTLNREGTETFADHREGILKTAKVLVEDTKVLVQNAAGSQEKLAQAAQSSVATITRLADVVKLGAASLGAEDPETQVVLINAVKDVAKALGDLISATKA
Splooce       AGTLNREGTETFADHREGILKTAKVLVEDTKVLVQNAAGSQEKLAQAAQSSVATITRLADVVKLGAASLGAEDPETQVVLINAVKDVAKALGDLISATKA

Uniprot       AAGKVGDDPAVWQLKNSAKVMVTNVTSLLKTVKAVEDEATKGTRALEATTEHIRQELAVFCSPEPPAKTSTPEDFIRMTKGITMATAKAVAAGNSCRQED
Splooce       AAGKVGDDPAVWQLKNSAKVMVTNVTSLLKTVKAVEDEATKGTRALEATTEHIRQELAVFCSPEPPAKTSTPEDFIRMTKGITMATAKAVAAGNSCRQED

Uniprot       VIATANLSRRAIADMLRACKEAAYHPEVAPDVRLRALHYGRECANGYLELLDHVLLTLQKPSPELKQQLTGHSKRVAGSVTELIQAAEAMKGTEWVDPED
Splooce       VIATANLSRRAIADMLRACKEAAYHPEVAPDVRLRALHYGRECANGYLELLDHVLLTLQKPSPELKQQLTGHSKRVAGSVTELIQAAEAMKGTEWVDPED

Uniprot       PTVIAENELLGAAAAIEAAAKKLEQLKPRAKPKEADESLNFEEQILEAAKSIAAATSALVKAASAAQRELVAQGKVGAIPANALDDGQWSQGLISAARMV
Splooce       PTVIAENELLGAAAAIEAAAKKLEQLKPRAKPKEADESLNFEEQILEAAKSIAAATSALVKAASAAQRELVAQGKVGAIPANALDDGQWSQGLISAARMV

Uniprot       AAATNNLCEAANAAVQGHASQEKLISSAKQVAASTAQLLVACKVKADQDSEAMKRLQAAGNAVKRASDNLVKAAQKAAAFEEQENETVVVKEKMVGGIAQ
Splooce       AAATNNLCEAANAAVQGHASQEKLISSAKQVAASTAQLLVACKVKADQDSEAMKRLQAAGNAVKRASDNLVKAAQKAAAFEEQENETVVVKEKMVGGIAQ

Uniprot       IIAAQEEMLRKERELEEARKKLAQIRQQQYKFLPSELRDEH
Splooce       IIAAQEEMLRKERELEEARKKLAQIRQQQYKFLPSELRDEH

----------------------------------------------------------------------------------------------------

Q12874 (Uniprot)	versus
NM_006802#(f-s-:1_S3673528875064) (Splooce)

For more details about the Alternative Splicing Event -> Link to Splooce page

Peptides that support the ASE (Splooce-specific):
SVEFEELLK (MAXQUANT + PEAKS)

Alignment:
Uniprot       METILEQQRRYHEEKERLMDVMAKEMLTKKSTLRDQINSDHRTRAMQDRYMEVSGNLRDLYDDKDGLRKEELNAISGPNEFAEFYNRLKQIKEFHRKHPN
Splooce       ----------------------------------------------------------------------------------------------------

Uniprot       EICVPMSVEFEELLKARENPSEEAQNLVEFTDEEGYGRYLDLHDCYLKYINLKASEKLDYITYLSIFDQLFDIPKERKNAEYKRYLEMLLEYLQDYTDRV
Splooce       -----MSVEFEELLKARENPSEEAQNLVEFTDEEGYGRYLDLHDCYLKYINLKASEKLDYITYLSIFDQLFDIPKERKNAEYKRYLEMLLEYLQDYTDRV

Uniprot       KPLQDQNELFGKIQAEFEKKWENGTFPGWPKETSSALTHAGAHLDLSAFSSWEELASLGLDRLKSALLALGLKCGGTLEERAQRLFSTKGKSLESLDTSL
Splooce       KPLQDQNELFGKIQAEFEKKWENGTFPGWPKETSSALTHAGAHLDLSAFSSWEELASLGLDRLKSALLALGLKCGGTLEERAQRLFSTKGKSLESLDTSL

Uniprot       FAKNPKSKGTKRDTERNKDIAFLEAQIYEYVEILGEQRHLTHENVQRKQARTGEEREEEEEEQISESESEDEENEIIYNPKNLPLGWDGKPIPYWLYKLH
Splooce       FAKNPKSKGTKRDTERNKDIAFLEAQIYEYVEILGEQRHLTHENVQRKQARTGEEREEEEEEQISESESEDEENEIIYNPKNLPLGWDGKPIPYWLYKLH

Uniprot       GLNINYNCEICGNYTYRGPKAFQRHFAEWRHAHGMRCLGIPNTAHFANVTQIEDAVSLWAKLKLQKASERWQPDTEEEYEDSSGNVVNKKTYEDLKRQGL
Splooce       GLNINYNCEICGNYTYRGPKAFQRHFAEWRHAHGMRCLGIPNTAHFANVTQIEDAVSLWAKLKLQKASERWQPDTEEEYEDSSGNVVNKKTYEDLKRQGL

Uniprot       L
Splooce       L

----------------------------------------------------------------------------------------------------

Q01518 (Uniprot)	versus
NM_006367#(f-:1_C7235634113274) (Splooce)

For more details about the Alternative Splicing Event -> Link to Splooce page

Peptides that support the ASE (Splooce-specific):
MMPPCFIQTEDK (MAXQUANT)

Alignment:
Uniprot       MADMQNLVERLERAVGRLEAVSHTSDMHRGYADSPSKAGAAPYVQAFDSLLAGPVAEYLKISKEIGGDVQKHAEMVHTGLKLERALLVTASQCQQPAENK
Splooce       ----------------------------------------------------------------------------------------------------

Uniprot       LSDLLAPISEQIKEVITFREKNRGSKLFNHLSAVSESIQALGWVAMAPKPGPYVKEMNDAAMFYTNRVLKEYKDVDKKHVDWVKAYLSIWTELQAYIKEF
Splooce       --------------------------------------------------------MMPPCFIQTE---------DKKHVDWVKAYLSIWTELQAYIKEF

Uniprot       HTTGLAWSKTGPVAKELSGLPSGPSAGSCPPPPPPCPPPPPVSTISCSYESASRSSLFAQINQGESITHALKHVSDDMKTHKNPALKAQSGPVRSGPKPF
Splooce       HTTGLAWSKTGPVAKELSGLPSGPSAGSCPPPPPPCPPPPPVSTISCSYESASRSSLFAQINQGESITHALKHVSDDMKTHKNPALKAQSGPVRSGPKPF

Uniprot       SAPKPQTSPSPKRATKKEPAVLELEGKKWRVENQENVSNLVIEDTELKQVAYIYKCVNTTLQIKGKINSITVDNCKKLGLVFDDVVGIVEIINSKDVKVQ
Splooce       SAPKPQTSPSPKRATKKEPAVLELEGKKWRVENQENVSNLVIEDTELKQVAYIYKCVNTTLQIKGKINSITVDNCKKLGLVFDDVVGIVEIINSKDVKVQ

Uniprot       VMGKVPTISINKTDGCHAYLSKNSLDCEIVSAKSSEMNVLIPTEGGDFNEFPVPEQFKTLWNGQKLVTTVTEIAG
Splooce       VMGKVPTISINKTDGCHAYLSKNSLDCEIVSAKSSEMNVLIPTEGGDFNEFPVPEQFKTLWNGQKLVTTVTEIAG

----------------------------------------------------------------------------------------------------

P21980 (Uniprot)	versus
NM_198951#(-s-s-s-s-:20_T1402260288719) (Splooce)

For more details about the Alternative Splicing Event -> Link to Splooce page

Peptides that support the ASE (Splooce-specific):
TRPDLQPGYEGWQALDPTPQEK (MAXQUANT)
MTRPDLQPGYEGWQALDPTPQEK (MAXQUANT)

Alignment:
Uniprot       MAEELVLERCDLELETNGRDHHTADLCREKLVVRRGQPFWLTLHFEGRNYEASVDSLTFSVVTGPAPSQEAGTKARFPLRDAVEEGDWTATVVDQQDCTL
Splooce       ----------------------------------------------------------------------------------------------------

Uniprot       SLQLTTPANAPIGLYRLSLEASTGYQGSSFVLGHFILLFNAWCPADAVYLDSEEERQEYVLTQQGFIYQGSAKFIKNIPWNFGQFEDGILDICLILLDVN
Splooce       ----------------------------------------------------------------------------------------------------

Uniprot       PKFLKNAGRDCSRRSSPVYVGRVVSGMVNCNDDQGVLLGRWDNNYGDGVSPMSWIGSVDILRRWKNHGCQRVKYGQCWVFAAVACTVLRCLGIPTRVVTN
Splooce       ----------------------------------------------------------------------------------------------------

Uniprot       YNSAHDQNSNLLIEYFRNEFGEIQGDKSEMIWNFHCWVESWMTRPDLQPGYEGWQALDPTPQEKSEGTYCCGPVPVRAIKEGDLSTKYDAPFVFAEVNAD
Splooce       -----------------------------------------MTRPDLQPGYEGWQALDPTPQEKSEGTYCCGPVPVRAIKEGDLSTKYDAPFVFAEVNAD

Uniprot       VVDWIQQDDGSVHKSINRSLIVGLKISTKSVGRDEREDITHTYKYPEGSSEEREAFTRANHLNKLAEKEETGMAMRIRVGQSMNMGSDFDVFAHITNNTA
Splooce       VVDWIQQDDGSVHKSINRSLIVGLKISTKSVGRDEREDITHTYKYPEGSSEEREAFTRANHLNKLAEKEETGMAMRIRVGQSMNMGSDFDVFAHITNNTA

Uniprot       EEYVCRLLLCARTVSYNGILGPECGTKYLLNLNLEPFSGKALCSWSIC
Splooce       EEYVCRLLLCARTVSYNGILGPECGTKYLLNLNLEPFSGKALCSWSIC

----------------------------------------------------------------------------------------------------

P68363 (Uniprot)	versus
NM_006082#(-t:12_T719833960172) (Splooce)

For more details about the Alternative Splicing Event -> Link to Splooce page

Peptides that support the ASE (Splooce-specific):
VDNEAIYDICRR (MAXQUANT)
MVDNEAIYDICRR (MAXQUANT)
MVDNEALYDLCR (PEAKS)
MVDNEAIYDICR (MAXQUANT)
VDNEALYDLCR (PEAKS)
VDNEAIYDICR (MAXQUANT)

Alignment:
Uniprot       MRECISIHVGQAGVQIGNACWELYCLEHGIQPDGQMPSDKTIGGGDDSFNTFFSETGAGKHVPRAVFVDLEPTVIDEVRTGTYRQLFHPEQLITGKEDAA
Splooce       ----------------------------------------------------------------------------------------------------

Uniprot       NNYARGHYTIGKEIIDLVLDRIRKLADQCTGLQGFLVFHSFGGGTGSGFTSLLMERLSVDYGKKSKLEFSIYPAPQVSTAVVEPYNSILTTHTTLEHSDC
Splooce       ----------------------------------------------------------------------------------------------------

Uniprot       AFMVDNEAIYDICRRNLDIERPTYTNLNRLISQIVSSITASLRFDGALNVDLTEFQTNLVPYPRIHFPLATYAPVISAEKAYHEQLSVAEITNACFEPAN
Splooce       --MVDNEAIYDICRRNLDIERPTYTNLNRLISQIVSSITASLRFDGALNVDLTEFQTNLVPYPRIHFPLATYAPVISAEKAYHEQLSVAEITNACFEPAN

Uniprot       QMVKCDPRHGKYMACCLLYRGDVVPKDVNAAIATIKTKRSIQFVDWCPTGFKVGINYQPPTVVPGGDLAKVQRAVCMLSNTTAIAEAWARLDHKFDLMYA
Splooce       QMVKCDPRHGKYMACCLLYRGDVVPKDVNAAIATIKTKRSIQFVDWCPTGFKVGINYQPPTVVPGGDLAKVQRAVCMLSNTTAIAEAWARLDHKFDLMYA

Uniprot       KRAFVHWYVGEGMEEGEFSEAREDMAALEKDYEEVGVDSVEGEGEEEGEEY
Splooce       KRAFVHWYVGEGMEEGEFSEAREDMAALEKDYEEVGVDSVEGEGEEEGEEY

----------------------------------------------------------------------------------------------------

P52566 (Uniprot)	versus
NM_001175#(f-:12_A850356601900) (Splooce)

For more details about the Alternative Splicing Event -> Link to Splooce page

Peptides that support the ASE (Splooce-specific):
DLTGDLEALKK (MAXQUANT)
DLTGDLEALK (MAXQUANT)

Alignment:
Uniprot       MTEKAPEPHVEEDDDDELDSKLNYKPPPQKSLKELQEMDKDDESLIKYKKTLLGDGPVVTDPKAPNVVVTRLTLVCESAPGPITMDLTGDLEALKKETIV
Splooce       ------------------------------------------------------------------------------------MDLTGDLEALKKETIV

Uniprot       LKEGSEYRVKIHFKVNRDIVSGLKYVQHTYRTGVKVDKATFMVGSYGPRPEEYEFLTPVEEAPKGMLARGTYHNKSFFTDDDKQDHLSWEWNLSIKKEWT
Splooce       LKEGSEYRVKIHFKVNRDIVSGLKYVQHTYRTGVKVDKATFMVGSYGPRPEEYEFLTPVEEAPKGMLARGTYHNKSFFTDDDKQDHLSWEWNLSIKKEWT

Uniprot       E
Splooce       E

----------------------------------------------------------------------------------------------------

P49591 (Uniprot)	versus
NM_006513#(f-:1_S8206985118591) (Splooce)

For more details about the Alternative Splicing Event -> Link to Splooce page

Peptides that support the ASE (Splooce-specific):
VDGFEGEK (MAXQUANT)

Alignment:
Uniprot       MVLDLDLFRVDKGGDPALIRETQEKRFKDPGLVDQLVKADSEWRRCRFRADNLNKLKNLCSKTIGEKMKKKEPVGDDESVPENVLSFDDLTADALANLKV
Splooce       ----------------------------------------------------------------------------------------------------

Uniprot       SQIKKVRLLIDEAILKCDAERIKLEAERFENLREIGNLLHPSVPISNDEDVDNKVERIWGDCTVRKKYSHVDLVVMVDGFEGEKGAVVAGSRGYFLKGVL
Splooce       ---------------------------------------------------------------------------MVDGFEGEKGAVVAGSRGYFLKGVL

Uniprot       VFLEQALIQYALRTLGSRGYIPIYTPFFMRKEVMQEVAQLSQFDEELYKVIGKGSEKSDDNSYDEKYLIATSEQPIAALHRDEWLRPEDLPIKYAGLSTC
Splooce       VFLEQALIQYALRTLGSRGYIPIYTPFFMRKEVMQEVAQLSQFDEELYKVIGKGSEKSDDNSYDEKYLIATSEQPIAALHRDEWLRPEDLPIKYAGLSTC

Uniprot       FRQEVGSHGRDTRGIFRVHQFEKIEQFVYSSPHDNKSWEMFEEMITTAEEFYQSLGIPYHIVNIVSGSLNHAASKKLDLEAWFPGSGAFRELVSCSNCTD
Splooce       FRQEVGSHGRDTRGIFRVHQFEKIEQFVYSSPHDNKSWEMFEEMITTAEEFYQSLGIPYHIVNIVSGSLNHAASKKLDLEAWFPGSGAFRELVSCSNCTD

Uniprot       YQARRLRIRYGQTKKMMDKVEFVHMLNATMCATTRTICAILENYQTEKGITVPEKLKEFMPPGLQELIPFVKPAPIEQEPSKKQKKQHEGSKKKAAARDV
Splooce       YQARRLRIRYGQTKKMMDKVEFVHMLNATMCATTRTICAILENYQTEKGITVPEKLKEFMPPGLQELIPFVKPAPIEQEPSKKQKKQHEGSKKKAAARDV

Uniprot       TLENRLQNMEVTDA
Splooce       TLENRLQNMEVTDA

----------------------------------------------------------------------------------------------------

Q9NR12 (Uniprot)	versus
NM_203352#(f-:5_P5839393864633) (Splooce)

For more details about the Alternative Splicing Event -> Link to Splooce page

Peptides that support the ASE (Splooce-specific):
MQDPDEEHLK (MAXQUANT)

Alignment:
Uniprot       MDSFKVVLEGPAPWGFRLQGGKDFNVPLSISRLTPGGKAAQAGVAVGDWVLSIDGENAGSLTHIEAQNKIRACGERLSLGLSRAQPVQSKPQKVQTPDKQ
Splooce       ----------------------------------------------------------------------------------------------------

Uniprot       PLRPLVPDASKQRLMENTEDWRPRPGTGQSRSFRILAHLTGTEFMQDPDEEHLKKSSQVPRTEAPAPASSTPQEPWPGPTAPSPTSRPPWAVDPAFAERY
Splooce       --------------------------------------------MQDPDEEHLKKSSQVPRTEAPAPASSTPQEPWPGPTAPSPTSRPPWAVDPAFAERY

Uniprot       APDKTSTVLTRHSQPATPTPLQSRTSIVQAAAGGVPGGGSNNGKTPVCHQCHKVIRGRYLVALGHAYHPEEFVCSQCGKVLEEGGFFEEKGAIFCPPCYD
Splooce       APDKTSTVLTRHSQPATPTPLQSRTSIVQAAAGGVPGGGSNNGKTPVCHQCHKVIRGRYLVALGHAYHPEEFVCSQCGKVLEEGGFFEEKGAIFCPPCYD

Uniprot       VRYAPSCAKCKKKITGEIMHALKMTWHVHCFTCAACKTPIRNRAFYMEEGVPYCERDYEKMFGTKCHGCDFKIDAGDRFLEALGFSWHDTCFVCAICQIN
Splooce       VRYAPSCAKCKKKITGEIMHALKMTWHVHCFTCAACKTPIRNRAFYMEEGVPYCERDYEKMFGTKCHGCDFKIDAGDRFLEALGFSWHDTCFVCAICQIN

Uniprot       LEGKTFYSKKDRPLCKSHAFSHV
Splooce       LEGKTFYSKKDRPLCKSHAFSHV

----------------------------------------------------------------------------------------------------

Q13228 (Uniprot)	versus
NM_003944#(-s-:1_S4145090175680) (Splooce)

For more details about the Alternative Splicing Event -> Link to Splooce page

Peptides that support the ASE (Splooce-specific):
ISTEWAAPNVLR (MAXQUANT)

Alignment:
Uniprot       MATKCGNCGPGYSTPLEAMKGPREEIVYLPCIYRNTGTEAPDYLATVDVDPKSPQYCQVIHRLPMPNLKDELHHSGWNTCSSCFGDSTKSRTKLVLPSLI
Splooce       ----------------------------------------------------------------------------------------------------

Uniprot       SSRIYVVDVGSEPRAPKLHKVIEPKDIHAKCELAFLHTSHCLASGEVMISSLGDVKGNGKGGFVLLDGETFEVKGTWERPGGAAPLGYDFWYQPRHNVMI
Splooce       --------------------------------------------------------------------------------------------------MI

Uniprot       STEWAAPNVLRDGFNPADVEAGLYGSHLYVWDWQRHEIVQTLSLKDGLIPLEIRFLHNPDAAQGFVGCALSSTIQRFYKNEGGTWSVEKVIQVPPKKVKG
Splooce       STEWAAPNVLRDGFNPADVEAGLYGSHLYVWDWQRHEIVQTLSLKDGLIPLEIRFLHNPDAAQGFVGCALSSTIQRFYKNEGGTWSVEKVIQVPPKKVKG

Uniprot       WLLPEMPGLITDILLSLDDRFLYFSNWLHGDLRQYDISDPQRPRLTGQLFLGGSIVKGGPVQVLEDEELKSQPEPLVVKGKRVAGGPQMIQLSLDGKRLY
Splooce       WLLPEMPGLITDILLSLDDRFLYFSNWLHGDLRQYDISDPQRPRLTGQLFLGGSIVKGGPVQVLEDEELKSQPEPLVVKGKRVAGGPQMIQLSLDGKRLY

Uniprot       ITTSLYSAWDKQFYPDLIREGSVMLQVDVDTVKGGLKLNPNFLVDFGKEPLGPALAHELRYPGGDCSSDIWI
Splooce       ITTSLYSAWDKQFYPDLIREGSVMLQVDVDTVKGGLKLNPNFLVDFGKEPLGPALAHELRYPGGDCSSDIWI

----------------------------------------------------------------------------------------------------

P15104 (Uniprot)	versus
NM_001033044#(-t:1_G9104259088430) (Splooce)

For more details about the Alternative Splicing Event -> Link to Splooce page

Peptides that support the ASE (Splooce-specific):
YIWIDGTGEGLR (MAXQUANT)
MYIWIDGTGEGLR (MAXQUANT)

Alignment:
Uniprot       MTTSASSHLNKGIKQVYMSLPQGEKVQAMYIWIDGTGEGLRCKTRTLDSEPKCVEELPEWNFDGSSTLQSEGSNSDMYLVPAAMFRDPFRKDPNKLVLCE
Splooce       ----------------------------MYIWIDGTGEGLRCKTRTLDSEPKCVEELPEWNFDGSSTLQSEGSNSDMYLVPAAMFRDPFRKDPNKLVLCE

Uniprot       VFKYNRRPAETNLRHTCKRIMDMVSNQHPWFGMEQEYTLMGTDGHPFGWPSNGFPGPQGPYYCGVGADRAYGRDIVEAHYRACLYAGVKIAGTNAEVMPA
Splooce       VFKYNRRPAETNLRHTCKRIMDMVSNQHPWFGMEQEYTLMGTDGHPFGWPSNGFPGPQGPYYCGVGADRAYGRDIVEAHYRACLYAGVKIAGTNAEVMPA

Uniprot       QWEFQIGPCEGISMGDHLWVARFILHRVCEDFGVIATFDPKPIPGNWNGAGCHTNFSTKAMREENGLKYIEEAIEKLSKRHQYHIRAYDPKGGLDNARRL
Splooce       QWEFQIGPCEGISMGDHLWVARFILHRVCEDFGVIATFDPKPIPGNWNGAGCHTNFSTKAMREENGLKYIEEAIEKLSKRHQYHIRAYDPKGGLDNARRL

Uniprot       TGFHETSNINDFSAGVANRSASIRIPRTVGQEKKGYFEDRRPSANCDPFSVTEALIRTCLLNETGDEPFQYKN
Splooce       TGFHETSNINDFSAGVANRSASIRIPRTVGQEKKGYFEDRRPSANCDPFSVTEALIRTCLLNETGDEPFQYKN

----------------------------------------------------------------------------------------------------

O75533 (Uniprot)	versus
NM_012433#(-s-s-s-s-:2_S3685980683004) (Splooce)

For more details about the Alternative Splicing Event -> Link to Splooce page

Peptides that support the ASE (Splooce-specific):
MISTMRPDIDNMDEYVR (MAXQUANT)
ISTMRPDIDNMDEYVR (MAXQUANT)

Alignment:
Uniprot       MAKIAKTHEDIEAQIREIQGKKAALDEAQGVGLDSTGYYDQEIYGGSDSRFAGYVTSIAATELEDDDDDYSSSTSLLGQKKPGYHAPVALLNDIPQSTEQ
Splooce       ----------------------------------------------------------------------------------------------------

Uniprot       YDPFAEHRPPKIADREDEYKKHRRTMIISPERLDPFADGGKTPDPKMNARTYMDVMREQHLTKEEREIRQQLAEKAKAGELKVVNGAAASQPPSKRKRRW
Splooce       ----------------------------------------------------------------------------------------------------

Uniprot       DQTADQTPGATPKKLSSWDQAETPGHTPSLRWDETPGRAKGSETPGATPGSKIWDPTPSHTPAGAATPGRGDTPGHATPGHGGATSSARKNRWDETPKTE
Splooce       ----------------------------------------------------------------------------------------------------

Uniprot       RDTPGHGSGWAETPRTDRGGDSIGETPTPGASKRKSRWDETPASQMGGSTPVLTPGKTPIGTPAMNMATPTPGHIMSMTPEQLQAWRWEREIDERNRPLS
Splooce       ----------------------------------------------------------------------------------------------------

Uniprot       DEELDAMFPEGYKVLPPPAGYVPIRTPARKLTATPTPLGGMTGFHMQTEDRTMKSVNDQPSGNLPFLKPDDIQYFDKLLVDVDESTLSPEEQKERKIMKL
Splooce       ----------------------------------------------------------------------------------------------------

Uniprot       LLKIKNGTPPMRKAALRQITDKAREFGAGPLFNQILPLLMSPTLEDQERHLLVKVIDRILYKLDDLVRPYVHKILVVIEPLLIDEDYYARVEGREIISNL
Splooce       ----------------------------------------------------------------------------------------------------

Uniprot       AKAAGLATMISTMRPDIDNMDEYVRNTTARAFAVVASALGIPSLLPFLKAVCKSKKSWQARHTGIKIVQQIAILMGCAILPHLRSLVEIIEHGLVDEQQK
Splooce       --------MISTMRPDIDNMDEYVRNTTARAFAVVASALGIPSLLPFLKAVCKSKKSWQARHTGIKIVQQIAILMGCAILPHLRSLVEIIEHGLVDEQQK

Uniprot       VRTISALAIAALAEAATPYGIESFDSVLKPLWKGIRQHRGKGLAAFLKAIGYLIPLMDAEYANYYTREVMLILIREFQSPDEEMKKIVLKVVKQCCGTDG
Splooce       VRTISALAIAALAEAATPYGIESFDSVLKPLWKGIRQHRGKGLAAFLKAIGYLIPLMDAEYANYYTREVMLILIREFQSPDEEMKKIVLKVVKQCCGTDG

Uniprot       VEANYIKTEILPPFFKHFWQHRMALDRRNYRQLVDTTVELANKVGAAEIISRIVDDLKDEAEQYRKMVMETIEKIMGNLGAADIDHKLEEQLIDGILYAF
Splooce       VEANYIKTEILPPFFKHFWQHRMALDRRNYRQLVDTTVELANKVGAAEIISRIVDDLKDEAEQYRKMVMETIEKIMGNLGAADIDHKLEEQLIDGILYAF

Uniprot       QEQTTEDSVMLNGFGTVVNALGKRVKPYLPQICGTVLWRLNNKSAKVRQQAADLISRTAVVMKTCQEEKLMGHLGVVLYEYLGEEYPEVLGSILGALKAI
Splooce       QEQTTEDSVMLNGFGTVVNALGKRVKPYLPQICGTVLWRLNNKSAKVRQQAADLISRTAVVMKTCQEEKLMGHLGVVLYEYLGEEYPEVLGSILGALKAI

Uniprot       VNVIGMHKMTPPIKDLLPRLTPILKNRHEKVQENCIDLVGRIADRGAEYVSAREWMRICFELLELLKAHKKAIRRATVNTFGYIAKAIGPHDVLATLLNN
Splooce       VNVIGMHKMTPPIKDLLPRLTPILKNRHEKVQENCIDLVGRIADRGAEYVSAREWMRICFELLELLKAHKKAIRRATVNTFGYIAKAIGPHDVLATLLNN

Uniprot       LKVQERQNRVCTTVAIAIVAETCSPFTVLPALMNEYRVPELNVQNGVLKSLSFLFEYIGEMGKDYIYAVTPLLEDALMDRDLVHRQTASAVVQHMSLGVY
Splooce       LKVQERQNRVCTTVAIAIVAETCSPFTVLPALMNEYRVPELNVQNGVLKSLSFLFEYIGEMGKDYIYAVTPLLEDALMDRDLVHRQTASAVVQHMSLGVY

Uniprot       GFGCEDSLNHLLNYVWPNVFETSPHVIQAVMGALEGLRVAIGPCRMLQYCLQGLFHPARKVRDVYWKIYNSIYIGSQDALIAHYPRIYNDDKNTYIRYEL
Splooce       GFGCEDSLNHLLNYVWPNVFETSPHVIQAVMGALEGLRVAIGPCRMLQYCLQGLFHPARKVRDVYWKIYNSIYIGSQDALIAHYPRIYNDDKNTYIRYEL

Uniprot       DYIL
Splooce       DYIL

----------------------------------------------------------------------------------------------------

P13667 (Uniprot)	versus
NM_004911#(-s-s-s-:7_P9834865994093) (Splooce)

For more details about the Alternative Splicing Event -> Link to Splooce page

Peptides that support the ASE (Splooce-specific):
MIEQSGPPSK (MAXQUANT)

Alignment:
Uniprot       MRPRKAFLLLLLLGLVQLLAVAGAEGPDEDSSNRENAIEDEEEEEEEDDDEEEDDLEVKEENGVLVLNDANFDNFVADKDTVLLEFYAPWCGHCKQFAPE
Splooce       ----------------------------------------------------------------------------------------------------

Uniprot       YEKIANILKDKDPPIPVAKIDATSASVLASRFDVSGYPTIKILKKGQAVDYEGSRTQEEIVAKVREVSQPDWTPPPEVTLVLTKENFDEVVNDADIILVE
Splooce       ----------------------------------------------------------------------------------------------------

Uniprot       FYAPWCGHCKKLAPEYEKAAKELSKRSPPIPLAKVDATAETDLAKRFDVSGYPTLKIFRKGRPYDYNGPREKYGIVDYMIEQSGPPSKEILTLKQVQEFL
Splooce       ------------------------------------------------------------------------------MIEQSGPPSKEILTLKQVQEFL

Uniprot       KDGDDVIIIGVFKGESDPAYQQYQDAANNLREDYKFHHTFSTEIAKFLKVSQGQLVVMQPEKFQSKYEPRSHMMDVQGSTQDSAIKDFVLKYALPLVGHR
Splooce       KDGDDVIIIGVFKGESDPAYQQYQDAANNLREDYKFHHTFSTEIAKFLKVSQGQLVVMQPEKFQSKYEPRSHMMDVQGSTQDSAIKDFVLKYALPLVGHR

Uniprot       KVSNDAKRYTRRPLVVVYYSVDFSFDYRAATQFWRSKVLEVAKDFPEYTFAIADEEDYAGEVKDLGLSESGEDVNAAILDESGKKFAMEPEEFDSDTLRE
Splooce       KVSNDAKRYTRRPLVVVYYSVDFSFDYRAATQFWRSKVLEVAKDFPEYTFAIADEEDYAGEVKDLGLSESGEDVNAAILDESGKKFAMEPEEFDSDTLRE

Uniprot       FVTAFKKGKLKPVIKSQPVPKNNKGPVKVVVGKTFDSIVMDPKKDVLIEFYAPWCGHCKQLEPVYNSLAKKYKGQKGLVIAKMDATANDVPSDRYKVEGF
Splooce       FVTAFKKGKLKPVIKSQPVPKNNKGPVKVVVGKTFDSIVMDPKKDVLIEFYAPWCGHCKQLEPVYNSLAKKYKGQKGLVIAKMDATANDVPSDRYKVEGF

Uniprot       PTIYFAPSGDKKNPVKFEGGDRDLEHLSKFIEEHATKLSRTKEEL
Splooce       PTIYFAPSGDKKNPVKFEGGDRDLEHLSKFIEEHATKLSRTKEEL

----------------------------------------------------------------------------------------------------

P55265 (Uniprot)	versus
NM_001025107#(-s-:1_A7004841870891) (Splooce)

For more details about the Alternative Splicing Event -> Link to Splooce page

Peptides that support the ASE (Splooce-specific):
ISESLDNLESMMPNK (MAXQUANT)

Alignment:
Uniprot       MAEIKEKICDYLFNVSDSSALNLAKNIGLTKARDINAVLIDMERQGDVYRQGTTPPIWHLTDKKRERMQIKRNTNSVPETAPAAIPETKRNAEFLTCNIP
Splooce       ----------------------------------------------------------------------------------------------------

Uniprot       TSNASNNMVTTEKVENGQEPVIKLENRQEARPEPARLKPPVHYNGPSKAGYVDFENGQWATDDIPDDLNSIRAAPGEFRAIMEMPSFYSHGLPRCSPYKK
Splooce       ----------------------------------------------------------------------------------------------------

Uniprot       LTECQLKNPISGLLEYAQFASQTCEFNMIEQSGPPHEPRFKFQVVINGREFPPAEAGSKKVAKQDAAMKAMTILLEEAKAKDSGKSEESSHYSTEKESEK
Splooce       ----------------------------------------------------------------------------------------------------

Uniprot       TAESQTPTPSATSFFSGKSPVTTLLECMHKLGNSCEFRLLSKEGPAHEPKFQYCVAVGAQTFPSVSAPSKKVAKQMAAEEAMKALHGEATNSMASDNQPE
Splooce       ----------------------------------------------------------------------------------------------------

Uniprot       GMISESLDNLESMMPNKVRKIGELVRYLNTNPVGGLLEYARSHGFAAEFKLVDQSGPPHEPKFVYQAKVGGRWFPAVCAHSKKQGKQEAADAALRVLIGE
Splooce       -MISESLDNLESMMPNKVRKIGELVRYLNTNPVGGLLEYARSHGFAAEFKLVDQSGPPHEPKFVYQAKVGGRWFPAVCAHSKKQGKQEAADAALRVLIGE

Uniprot       NEKAERMGFTEVTPVTGASLRRTMLLLSRSPEAQPKTLPLTGSTFHDQIAMLSHRCFNTLTNSFQPSLLGRKILAAIIMKKDSEDMGVVVSLGTGNRCVK
Splooce       NEKAERMGFTEVTPVTGASLRRTMLLLSRSPEAQPKTLPLTGSTFHDQIAMLSHRCFNTLTNSFQPSLLGRKILAAIIMKKDSEDMGVVVSLGTGNRCVK

Uniprot       GDSLSLKGETVNDCHAEIISRRGFIRFLYSELMKYNSQTAKDSIFEPAKGGEKLQIKKTVSFHLYISTAPCGDGALFDKSCSDRAMESTESRHYPVFENP
Splooce       GDSLSLKGETVNDCHAEIISRRGFIRFLYSELMKYNSQTAKDSIFEPAKGGEKLQIKKTVSFHLYISTAPCGDGALFDKSCSDRAMESTESRHYPVFENP

Uniprot       KQGKLRTKVENGEGTIPVESSDIVPTWDGIRLGERLRTMSCSDKILRWNVLGLQGALLTHFLQPIYLKSVTLGYLFSQGHLTRAICCRVTRDGSAFEDGL
Splooce       KQGKLRTKVENGEGTIPVESSDIVPTWDGIRLGERLRTMSCSDKILRWNVLGLQGALLTHFLQPIYLKSVTLGYLFSQGHLTRAICCRVTRDGSAFEDGL

Uniprot       RHPFIVNHPKVGRVSIYDSKRQSGKTKETSVNWCLADGYDLEILDGTRGTVDGPRNELSRVSKKNIFLLFKKLCSFRYRRDLLRLSYGEAKKAARDYETA
Splooce       RHPFIVNHPKVGRVSIYDSKRQSGKTKETSVNWCLADGYDLEILDGTRGTVDGPRNELSRVSKKNIFLLFKKLCSFRYRRDLLRLSYGEAKKAARDYETA

Uniprot       KNYFKKGLKDMGYGNWISKPQEEKNFYLCPV
Splooce       KNYFKKGLKDMGYGNWISKPQEEKNFYLCPV

----------------------------------------------------------------------------------------------------

Q9Y624 (Uniprot)	versus
NM_016946#(r:1_F8585961338690) (Splooce)

For more details about the Alternative Splicing Event -> Link to Splooce page

Peptides that support the ASE (Splooce-specific):
VSEEGGNSYGEVK (MAXQUANT + PEAKS)

Alignment:
Uniprot       MGTKAQVERKLLCLFILAILLCSLALGSVTVHSSEPEVRIPENNPVKLSCAYSGFSSPRVEWKFDQGDTTRLVCYNNKITASYEDRVTFLPTGITFKSVT
Splooce       ----------------------------------------------------------------------------------------------------

Uniprot       REDTGTYTCMVSEEGGNSYGEVKVKLIVLVPPSKPTVNIPSSATIGNRAVLTCSEQDGSPPSEYTWFKDGIVMPTNPKSTRAFSNSSYVLNPTTGELVFD
Splooce       ---------MVSEEGGNSYGEVKVKLIVLVPPSKPTVNIPSSATIGNRAVLTCSEQDGSPPSEYTWFKDGIVMPTNPKSTRAFSNSSYVLNPTTGELVFD

Uniprot       PLSASDTGEYSCEARNGYGTPMTSNAVRMEAVERNVGVIVAAVLVTLILLGILVFGIWFAYSRGHFDSKTSSKKVIYSQPSARSEGEFKQTSSFLV
Splooce       PLSASDTGEYSCEARNGYGTPMTSNAVRMEAVERNVGVIVAAVLVTLILLGILVFGIWFAYSRGHFDSKTSSKKVIYSQPSARSEGEFKQTSSFLV

----------------------------------------------------------------------------------------------------

Q15437 (Uniprot)	versus
NM_032986#(-s-s-:20_S8016100175698) (Splooce)

For more details about the Alternative Splicing Event -> Link to Splooce page

Peptides that support the ASE (Splooce-specific):
SLSLLPPDALVGLITFGR (MAXQUANT)

Alignment:
Uniprot       MATYLEFIQQNEERDGVRFSWNVWPSSRLEATRMVVPLACLLTPLKERPDLPPVQYEPVLCSRPTCKAVLNPLCQVDYRAKLWACNFCFQRNQFPPAYGG
Splooce       ----------------------------------------------------------------------------------------------------

Uniprot       ISEVNQPAELMPQFSTIEYVIQRGAQSPLIFLYVVDTCLEEDDLQALKESLQMSLSLLPPDALVGLITFGRMVQVHELSCEGISKSYVFRGTKDLTAKQI
Splooce       ----------------------------------------------------MSLSLLPPDALVGLITFGRMVQVHELSCEGISKSYVFRGTKDLTAKQI

Uniprot       QDMLGLTKPAMPMQQARPAQPQEHPFASSRFLQPVHKIDMNLTDLLGELQRDPWPVTQGKRPLRSTGVALSIAVGLLEGTFPNTGARIMLFTGGPPTQGP
Splooce       QDMLGLTKPAMPMQQARPAQPQEHPFASSRFLQPVHKIDMNLTDLLGELQRDPWPVTQGKRPLRSTGVALSIAVGLLEGTFPNTGARIMLFTGGPPTQGP

Uniprot       GMVVGDELKIPIRSWHDIEKDNARFMKKATKHYEMLANRTAANGHCIDIYACALDQTGLLEMKCCANLTGGYMVMGDSFNTSLFKQTFQRIFTKDFNGDF
Splooce       GMVVGDELKIPIRSWHDIEKDNARFMKKATKHYEMLANRTAANGHCIDIYACALDQTGLLEMKCCANLTGGYMVMGDSFNTSLFKQTFQRIFTKDFNGDF

Uniprot       RMAFGATLDVKTSRELKIAGAIGPCVSLNVKGPCVSENELGVGGTSQWKICGLDPTSTLGIYFEVVNQHNTPIPQGGRGAIQFVTHYQHSSTQRRIRVTT
Splooce       RMAFGATLDVKTSRELKIAGAIGPCVSLNVKGPCVSENELGVGGTSQWKICGLDPTSTLGIYFEVVNQHNTPIPQGGRGAIQFVTHYQHSSTQRRIRVTT

Uniprot       IARNWADVQSQLRHIEAAFDQEAAAVLMARLGVFRAESEEGPDVLRWLDRQLIRLCQKFGQYNKEDPTSFRLSDSFSLYPQFMFHLRRSPFLQVFNNSPD
Splooce       IARNWADVQSQLRHIEAAFDQEAAAVLMARLGVFRAESEEGPDVLRWLDRQLIRLCQKFGQYNKEDPTSFRLSDSFSLYPQFMFHLRRSPFLQVFNNSPD

Uniprot       ESSYYRHHFARQDLTQSLIMIQPILYSYSFHGPPEPVLLDSSSILADRILLMDTFFQIVIYLGETIAQWRKAGYQDMPEYENFKHLLQAPLDDAQEILQA
Splooce       ESSYYRHHFARQDLTQSLIMIQPILYSYSFHGPPEPVLLDSSSILADRILLMDTFFQIVIYLGETIAQWRKAGYQDMPEYENFKHLLQAPLDDAQEILQA

Uniprot       RFPMPRYINTEHGGSQARFLLSKVNPSQTHNNLYAWGQETGAPILTDDVSLQVFMDHLKKLAVSSAC
Splooce       RFPMPRYINTEHGGSQARFLLSKVNPSQTHNNLYAWGQETGAPILTDDVSLQVFMDHLKKLAVSSAC

----------------------------------------------------------------------------------------------------

O75843 (Uniprot)	versus
NM_003917#(-s-s-s-s-s-s-s-s-:14_A8569981871961) (Splooce)

For more details about the Alternative Splicing Event -> Link to Splooce page

Peptides that support the ASE (Splooce-specific):
MSVTMAGTHVR (MAXQUANT)

Alignment:
Uniprot       MVVPSLKLQDLIEEIRGAKTQAQEREVIQKECAHIRASFRDGDPVHRHRQLAKLLYVHMLGYPAHFGQMECLKLIASSRFTDKRVGYLGAMLLLDERHDA
Splooce       ----------------------------------------------------------------------------------------------------

Uniprot       HLLITNSIKNDLSQGIQPVQGLALCTLSTMGSAEMCRDLAPEVEKLLLQPSPYVRKKAILTAVHMIRKVPELSSVFLPPCAQLLHERHHGILLGTITLIT
Splooce       ----------------------------------------------------------------------------------------------------

Uniprot       ELCERSPAALRHFRKVVPQLVHILRTLVTMGYSTEHSISGVSDPFLQVQILRLLRILGRNHEESSETMNDLLAQVATNTDTSRNAGNAVLFETVLTIMDI
Splooce       ----------------------------------------------------------------------------------------------------

Uniprot       RSAAGLRVLAVNILGRFLLNSDRNIRYVALTSLLRLVQSDHSAVQRHRPTVVECLRETDASLSRRALELSLALVNSSNVRAMMQELQAFLESCPPDLRAD
Splooce       ----------------------------------------------------------------------------------------------------

Uniprot       CASGILLAAERFAPTKRWHIDTILHVLTTAGTHVRDDAVANLTQLIGGAQELHAYSVRRLYNALAEDISQQPLVQVAAWCIGEYGDLLLAGNCEEIEPLQ
Splooce       ------------------------MSVTMAGTHVRDDAVANLTQLIGGAQELHAYSVRRLYNALAEDISQQPLVQVAAWCIGEYGDLLLAGNCEEIEPLQ

Uniprot       VDEEEVLALLEKVLQSHMSLPATRGYALTALMKLSTRLCGDNNRIRQVVSIYGSCLDVELQQRAVEYDTLFRKYDHMRAAILEKMPLVERDGPQADEEAK
Splooce       VDEEEVLALLEKVLQSHMSLPATRGYALTALMKLSTRLCGDNNRIRQVVSIYGSCLDVELQQRAVEYDTLFRKYDHMRAAILEKMPLVERDGPQADEEAK

Uniprot       ESKEAAQLSEAAPVPTEPQASQLLDLLDLLDGASGDVQHPPHLDPSPGGALVHLLDLPCVPPPPAPIPDLKVFEREGVQLNLSFIRPPENPALLLITITA
Splooce       ESKEAAQLSEAAPVPTEPQASQLLDLLDLLDGASGDVQHPPHLDPSPGGALVHLLDLPCVPPPPAPIPDLKVFEREGVQLNLSFIRPPENPALLLITITA

Uniprot       TNFSEGDVTHFICQAAVPKSLQLQLQAPSGNTVPARGGLPITQLFRILNPNKAPLRLKLRLTYDHFHQSVQEIFEVNNLPVESWQ
Splooce       TNFSEGDVTHFICQAAVPKSLQLQLQAPSGNTVPARGGLPITQLFRILNPNKAPLRLKLRLTYDHFHQSVQEIFEVNNLPVESWQ

----------------------------------------------------------------------------------------------------

P07814 (Uniprot)	versus
NM_004446#(-s-:1_E4814869076632) (Splooce)

For more details about the Alternative Splicing Event -> Link to Splooce page

Peptides that support the ASE (Splooce-specific):
EHTEIDHWLEFSATK (MAXQUANT)

Alignment:
Uniprot       MATLSLTVNSGDPPLGALLAVEHVKDDVSISVEEGKENILHVSENVIFTDVNSILRYLARVATTAGLYGSNLMEHTEIDHWLEFSATKLSSCDSFTSTIN
Splooce       ------------------------------------------------------------------------MEHTEIDHWLEFSATKLSSCDSFTSTIN

Uniprot       ELNHCLSLRTYLVGNSLSLADLCVWATLKGNAAWQEQLKQKKAPVHVKRWFGFLEAQQAFQSVGTKWDVSTTKARVAPEKKQDVGKFVELPGAEMGKVTV
Splooce       ELNHCLSLRTYLVGNSLSLADLCVWATLKGNAAWQEQLKQKKAPVHVKRWFGFLEAQQAFQSVGTKWDVSTTKARVAPEKKQDVGKFVELPGAEMGKVTV

Uniprot       RFPPEASGYLHIGHAKAALLNQHYQVNFKGKLIMRFDDTNPEKEKEDFEKVILEDVAMLHIKPDQFTYTSDHFETIMKYAEKLIQEGKAYVDDTPAEQMK
Splooce       RFPPEASGYLHIGHAKAALLNQHYQVNFKGKLIMRFDDTNPEKEKEDFEKVILEDVAMLHIKPDQFTYTSDHFETIMKYAEKLIQEGKAYVDDTPAEQMK

Uniprot       AEREQRIDSKHRKNPIEKNLQMWEEMKKGSQFGQSCCLRAKIDMSSNNGCMRDPTLYRCKIQPHPRTGNKYNVYPTYDFACPIVDSIEGVTHALRTTEYH
Splooce       AEREQRIDSKHRKNPIEKNLQMWEEMKKGSQFGQSCCLRAKIDMSSNNGCMRDPTLYRCKIQPHPRTGNKYNVYPTYDFACPIVDSIEGVTHALRTTEYH

Uniprot       DRDEQFYWIIEALGIRKPYIWEYSRLNLNNTVLSKRKLTWFVNEGLVDGWDDPRFPTVRGVLRRGMTVEGLKQFIAAQGSSRSVVNMEWDKIWAFNKKVI
Splooce       DRDEQFYWIIEALGIRKPYIWEYSRLNLNNTVLSKRKLTWFVNEGLVDGWDDPRFPTVRGVLRRGMTVEGLKQFIAAQGSSRSVVNMEWDKIWAFNKKVI

Uniprot       DPVAPRYVALLKKEVIPVNVPEAQEEMKEVAKHPKNPEVGLKPVWYSPKVFIEGADAETFSEGEMVTFINWGNLNITKIHKNADGKIISLDAKLNLENKD
Splooce       DPVAPRYVALLKKEVIPVNVPEAQEEMKEVAKHPKNPEVGLKPVWYSPKVFIEGADAETFSEGEMVTFINWGNLNITKIHKNADGKIISLDAKLNLENKD

Uniprot       YKKTTKVTWLAETTHALPIPVICVTYEHLITKPVLGKDEDFKQYVNKNSKHEELMLGDPCLKDLKKGDIIQLQRRGFFICDQPYEPVSPYSCKEAPCVLI
Splooce       YKKTTKVTWLAETTHALPIPVICVTYEHLITKPVLGKDEDFKQYVNKNSKHEELMLGDPCLKDLKKGDIIQLQRRGFFICDQPYEPVSPYSCKEAPCVLI

Uniprot       YIPDGHTKEMPTSGSKEKTKVEATKNETSAPFKERPTPSLNNNCTTSEDSLVLYNRVAVQGDVVRELKAKKAPKEDVDAAVKQLLSLKAEYKEKTGQEYK
Splooce       YIPDGHTKEMPTSGSKEKTKVEATKNETSAPFKERPTPSLNNNCTTSEDSLVLYNRVAVQGDVVRELKAKKAPKEDVDAAVKQLLSLKAEYKEKTGQEYK

Uniprot       PGNPPAEIGQNISSNSSASILESKSLYDEVAAQGEVVRKLKAEKSPKAKINEAVECLLSLKAQYKEKTGKEYIPGQPPLSQSSDSSPTRNSEPAGLETPE
Splooce       PGNPPAEIGQNISSNSSASILESKSLYDEVAAQGEVVRKLKAEKSPKAKINEAVECLLSLKAQYKEKTGKEYIPGQPPLSQSSDSSPTRNSEPAGLETPE

Uniprot       AKVLFDKVASQGEVVRKLKTEKAPKDQVDIAVQELLQLKAQYKSLIGVEYKPVSATGAEDKDKKKKEKENKSEKQNKPQKQNDGQRKDPSKNQGGGLSSS
Splooce       AKVLFDKVASQGEVVRKLKTEKAPKDQVDIAVQELLQLKAQYKSLIGVEYKPVSATGAEDKDKKKKEKENKSEKQNKPQKQNDGQRKDPSKNQGGGLSSS

Uniprot       GAGEGQGPKKQTRLGLEAKKEENLADWYSQVITKSEMIEYHDISGCYILRPWAYAIWEAIKDFFDAEIKKLGVENCYFPMFVSQSALEKEKTHVADFAPE
Splooce       GAGEGQGPKKQTRLGLEAKKEENLADWYSQVITKSEMIEYHDISGCYILRPWAYAIWEAIKDFFDAEIKKLGVENCYFPMFVSQSALEKEKTHVADFAPE

Uniprot       VAWVTRSGKTELAEPIAIRPTSETVMYPAYAKWVQSHRDLPIKLNQWCNVVRWEFKHPQPFLRTREFLWQEGHSAFATMEEAAEEVLQILDLYAQVYEEL
Splooce       VAWVTRSGKTELAEPIAIRPTSETVMYPAYAKWVQSHRDLPIKLNQWCNVVRWEFKHPQPFLRTREFLWQEGHSAFATMEEAAEEVLQILDLYAQVYEEL

Uniprot       LAIPVVKGRKTEKEKFAGGDYTTTIEAFISASGRAIQGGTSHHLGQNFSKMFEIVFEDPKIPGEKQFAYQNSWGLTTRTIGVMTMVHGDNMGLVLPPRVA
Splooce       LAIPVVKGRKTEKEKFAGGDYTTTIEAFISASGRAIQGGTSHHLGQNFSKMFEIVFEDPKIPGEKQFAYQNSWGLTTRTIGVMTMVHGDNMGLVLPPRVA

Uniprot       CVQVVIIPCGITNALSEEDKEALIAKCNDYRRRLLSVNIRVRADLRDNYSPGWKFNHWELKGVPIRLEVGPRDMKSCQFVAVRRDTGEKLTVAENEAETK
Splooce       CVQVVIIPCGITNALSEEDKEALIAKCNDYRRRLLSVNIRVRADLRDNYSPGWKFNHWELKGVPIRLEVGPRDMKSCQFVAVRRDTGEKLTVAENEAETK

Uniprot       LQAILEDIQVTLFTRASEDLKTHMVVANTMEDFQKILDSGKIVQIPFCGEIDCEDWIKKTTARDQDLEPGAPSMGAKSLCIPFKPLCELQPGAKCVCGKN
Splooce       LQAILEDIQVTLFTRASEDLKTHMVVANTMEDFQKILDSGKIVQIPFCGEIDCEDWIKKTTARDQDLEPGAPSMGAKSLCIPFKPLCELQPGAKCVCGKN

Uniprot       PAKYYTLFGRSY
Splooce       PAKYYTLFGRSY

----------------------------------------------------------------------------------------------------

P68363 (Uniprot)	versus
NM_006082#(-t:12_T1305596890809) (Splooce)

For more details about the Alternative Splicing Event -> Link to Splooce page

Peptides that support the ASE (Splooce-specific):
GIQPDGQMPSDK (MAXQUANT)

Alignment:
Uniprot       MRECISIHVGQAGVQIGNACWELYCLEHGIQPDGQMPSDKTIGGGDDSFNTFFSETGAGKHVPRAVFVDLEPTVIDEVRTGTYRQLFHPEQLITGKEDAA
Splooce       ---------------------------MGIQPDGQMPSDKTIGGGDDSFNTFFSETGAGKHVPRAVFVDLEPTVIDEVRTGTYRQLFHPEQLITGKEDAA

Uniprot       NNYARGHYTIGKEIIDLVLDRIRKLADQCTGLQGFLVFHSFGGGTGSGFTSLLMERLSVDYGKKSKLEFSIYPAPQVSTAVVEPYNSILTTHTTLEHSDC
Splooce       NNYARGHYTIGKEIIDLVLDRIRKLADQCTGLQGFLVFHSFGGGTGSGFTSLLMERLSVDYGKKSKLEFSIYPAPQVSTAVVEPYNSILTTHTTLEHSDC

Uniprot       AFMVDNEAIYDICRRNLDIERPTYTNLNRLISQIVSSITASLRFDGALNVDLTEFQTNLVPYPRIHFPLATYAPVISAEKAYHEQLSVAEITNACFEPAN
Splooce       AFMVDNEAIYDICRRNLDIERPTYTNLNRLISQIVSSITASLRFDGALNVDLTEFQTNLVPYPRIHFPLATYAPVISAEKAYHEQLSVAEITNACFEPAN

Uniprot       QMVKCDPRHGKYMACCLLYRGDVVPKDVNAAIATIKTKRSIQFVDWCPTGFKVGINYQPPTVVPGGDLAKVQRAVCMLSNTTAIAEAWARLDHKFDLMYA
Splooce       QMVKCDPRHGKYMACCLLYRGDVVPKDVNAAIATIKTKRSIQFVDWCPTGFKVGINYQPPTVVPGGDLAKVQRAVCMLSNTTAIAEAWARLDHKFDLMYA

Uniprot       KRAFVHWYVGEGMEEGEFSEAREDMAALEKDYEEVGVDSVEGEGEEEGEEY
Splooce       KRAFVHWYVGEGMEEGEFSEAREDMAALEKDYEEVGVDSVEGEGEEEGEEY

----------------------------------------------------------------------------------------------------

P07948 (Uniprot)	versus
NM_002350#(-s-s-s-:8_L6168363713558) (Splooce)

For more details about the Alternative Splicing Event -> Link to Splooce page

Peptides that support the ASE (Splooce-specific):
SVQAFLEEANLMK (MAXQUANT)

Alignment:
Uniprot       MGCIKSKGKDSLSDDGVDLKTQPVRNTERTIYVRDPTSNKQQRPVPESQLLPGQRFQTKDPEEQGDIVVALYPYDGIHPDDLSFKKGEKMKVLEEHGEWW
Splooce       ----------------------------------------------------------------------------------------------------

Uniprot       KAKSLLTKKEGFIPSNYVAKLNTLETEEWFFKDITRKDAERQLLAPGNSAGAFLIRESETLKGSFSLSVRDFDPVHGDVIKHYKIRSLDNGGYYISPRIT
Splooce       ----------------------------------------------------------------------------------------------------

Uniprot       FPCISDMIKHYQKQADGLCRRLEKACISPKPQKPWDKDAWEIPRESIKLVKRLGAGQFGEVWMGYYNNSTKVAVKTLKPGTMSVQAFLEEANLMKTLQHD
Splooce       ---------------------------------------------------------------------------------MSVQAFLEEANLMKTLQHD

Uniprot       KLVRLYAVVTREEPIYIITEYMAKGSLLDFLKSDEGGKVLLPKLIDFSAQIAEGMAYIERKNYIHRDLRAANVLVSESLMCKIADFGLARVIEDNEYTAR
Splooce       KLVRLYAVVTREEPIYIITEYMAKGSLLDFLKSDEGGKVLLPKLIDFSAQIAEGMAYIERKNYIHRDLRAANVLVSESLMCKIADFGLARVIEDNEYTAR

Uniprot       EGAKFPIKWTAPEAINFGCFTIKSDVWSFGILLYEIVTYGKIPYPGRTNADVMTALSQGYRMPRVENCPDELYDIMKMCWKEKAEERPTFDYLQSVLDDF
Splooce       EGAKFPIKWTAPEAINFGCFTIKSDVWSFGILLYEIVTYGKIPYPGRTNADVMTALSQGYRMPRVENCPDELYDIMKMCWKEKAEERPTFDYLQSVLDDF

Uniprot       YTATEGQYQQQP
Splooce       YTATEGQYQQQP

----------------------------------------------------------------------------------------------------

P31150 (Uniprot)	versus
NM_001493#(-s-s-s-s-:X_G6664253175290) (Splooce)

For more details about the Alternative Splicing Event -> Link to Splooce page

Peptides that support the ASE (Splooce-specific):
MISYAHNVAAQGK (MAXQUANT)

Alignment:
Uniprot       MDEEYDVIVLGTGLTECILSGIMSVNGKKVLHMDRNPYYGGESSSITPLEELYKRFQLLEGPPESMGRGRDWNVDLIPKFLMANGQLVKMLLYTEVTRYL
Splooce       ----------------------------------------------------------------------------------------------------

Uniprot       DFKVVEGSFVYKGGKIYKVPSTETEALASNLMGMFEKRRFRKFLVFVANFDENDPKTFEGVDPQTTSMRDVYRKFDLGQDVIDFTGHALALYRTDDYLDQ
Splooce       ----------------------------------------------------------------------------------------------------

Uniprot       PCLETVNRIKLYSESLARYGKSPYLYPLYGLGELPQGFARLSAIYGGTYMLNKPVDDIIMENGKVVGVKSEGEVARCKQLICDPSYIPDRVRKAGQVIRI
Splooce       ----------------------------------------------------------------------------------------------------

Uniprot       ICILSHPIKNTNDANSCQIIIPQNQVNRKSDIYVCMISYAHNVAAQGKYIAIASTTVETTDPEKEVEPALELLEPIDQKFVAISDLYEPIDDGCESQVFC
Splooce       -----------------------------------MISYAHNVAAQGKYIAIASTTVETTDPEKEVEPALELLEPIDQKFVAISDLYEPIDDGCESQVFC

Uniprot       SCSYDATTHFETTCNDIKDIYKRMAGTAFDFENMKRKQNDVFGEAEQ
Splooce       SCSYDATTHFETTCNDIKDIYKRMAGTAFDFENMKRKQNDVFGEAEQ

----------------------------------------------------------------------------------------------------

P09972 (Uniprot)	versus
NM_005165#(f-:17_A3625789402532) (Splooce)

For more details about the Alternative Splicing Event -> Link to Splooce page

Peptides that support the ASE (Splooce-specific):
MVTPGHACPIK (MAXQUANT)

Alignment:
Uniprot       MPHSYPALSAEQKKELSDIALRIVAPGKGILAADESVGSMAKRLSQIGVENTEENRRLYRQVLFSADDRVKKCIGGVIFFHETLYQKDDNGVPFVRTIQD
Splooce       ----------------------------------------------------------------------------------------------------

Uniprot       KGIVVGIKVDKGVVPLAGTDGETTTQGLDGLSERCAQYKKDGADFAKWRCVLKISERTPSALAILENANVLARYASICQQNGIVPIVEPEILPDGDHDLK
Splooce       ----------------------------------------------------------------------------------------------------

Uniprot       RCQYVTEKVLAAVYKALSDHHVYLEGTLLKPNMVTPGHACPIKYTPEEIAMATVTALRRTVPPAVPGVTFLSGGQSEEEASFNLNAINRCPLPRPWALTF
Splooce       --------------------------------MVTPGHACPIKYTPEEIAMATVTALRRTVPPAVPGVTFLSGGQSEEEASFNLNAINRCPLPRPWALTF

Uniprot       SYGRALQASALNAWRGQRDNAGAATEEFIKRAEVNGLAAQGKYEGSGEDGGAAAQSLYIANHAY
Splooce       SYGRALQASALNAWRGQRDNAGAATEEFIKRAEVNGLAAQGKYEGSGEDGGAAAQSLYIANHAY

----------------------------------------------------------------------------------------------------

UNIPROT? (Uniprot)	versus
NM_139201#(-s-s-s-s-:12_G4584643683393) (Splooce)

For more details about the Alternative Splicing Event -> Link to Splooce page

Peptides that support the ASE (Splooce-specific):
MADSSLDLSELAK (MAXQUANT + PEAKS)

Alignment:
Uniprot       -------
Splooce       ----------------------------------------------------------------------------------------------------

Uniprot       -------
Splooce       ----------------------------------------------------------------------------------------------------

Uniprot       -------
Splooce       ---------------------------------------------------MADSSLDLSELAKAAKKKLQSLSNHLFEELAMDVYDEVDRRETDAVWLA

Uniprot       -------
Splooce       TQNHSALVTETTVVPFLPVNPEYSSTRNQANRS

----------------------------------------------------------------------------------------------------

Q9UGP8 (Uniprot)	versus
NM_007214#(-s-s-:6_S2246845314431) (Splooce)

For more details about the Alternative Splicing Event -> Link to Splooce page

Peptides that support the ASE (Splooce-specific):
PGATVAEIKK (MAXQUANT)

Alignment:
Uniprot       MAGQQFQYDDSGNTFFYFLTSFVGLIVIPATYYLWPRDQNAEQIRLKNIRKVYGRCMWYRLRLLKPQPNIIPTVKKIVLLAGWALFLFLAYKVSKTDREY
Splooce       ----------------------------------------------------------------------------------------------------

Uniprot       QEYNPYEVLNLDPGATVAEIKKQYRLLSLKYHPDKGGDEVMFMRIAKAYAALTDEESRKNWEEFGNPDGPQATSFGIALPAWIVDQKNSILVLLVYGLAF
Splooce       -----------MPGATVAEIKKQYRLLSLKYHPDKGGDEVMFMRIAKAYAALTDEESRKNWEEFGNPDGPQATSFGIALPAWIVDQKNSILVLLVYGLAF

Uniprot       MVILPVVVGSWWYRSIRYSGDQILIRTTQIYTYFVYKTRNMDMKRLIMVLAGASEFDPQYNKDATSRPTDNILIPQLIREIGSINLKKNEPPLTCPYSLK
Splooce       MVILPVVVGSWWYRSIRYSGDQILIRTTQIYTYFVYKTRNMDMKRLIMVLAGASEFDPQYNKDATSRPTDNILIPQLIREIGSINLKKNEPPLTCPYSLK

Uniprot       ARVLLLSHLARMKIPETLEEDQQFMLKKCPALLQEMVNVICQLIVMARNREEREFRAPTLASLENCMKLSQMAVQGLQQFKSPLLQLPHIEEDNLRRVSN
Splooce       ARVLLLSHLARMKIPETLEEDQQFMLKKCPALLQEMVNVICQLIVMARNREEREFRAPTLASLENCMKLSQMAVQGLQQFKSPLLQLPHIEEDNLRRVSN

Uniprot       HKKYKIKTIQDLVSLKESDRHTLLHFLEDEKYEEVMAVLGSFPYVTMDIKSQVLDDEDSNNITVGSLVTVLVKLTRQTMAEVFEKEQSICAAEEQPAEDG
Splooce       HKKYKIKTIQDLVSLKESDRHTLLHFLEDEKYEEVMAVLGSFPYVTMDIKSQVLDDEDSNNITVGSLVTVLVKLTRQTMAEVFEKEQSICAAEEQPAEDG

Uniprot       QGETNKNRTKGGWQQKSKGPKKTAKSKKKKPLKKKPTPVLLPQSKQQKQKQANGVVGNEAAVKEDEEEVSDKGSDSEEEETNRDSQSEKDDGSDRDSDRE
Splooce       QGETNKNRTKGGWQQKSKGPKKTAKSKKKKPLKKKPTPVLLPQSKQQKQKQANGVVGNEAAVKEDEEEVSDKGSDSEEEETNRDSQSEKDDGSDRDSDRE

Uniprot       QDEKQNKDDEAEWQELQQSIQRKERALLETKSKITHPVYSLYFPEEKQEWWWLYIADRKEQTLISMPYHVCTLKDTEEVELKFPAPGKPGNYQYTVFLRS
Splooce       QDEKQNKDDEAEWQELQQSIQRKERALLETKSKITHPVYSLYFPEEKQEWWWLYIADRKEQTLISMPYHVCTLKDTEEVELKFPAPGKPGNYQYTVFLRS

Uniprot       DSYMGLDQIKPLKLEVHEAKPVPENHPQWDTAIEGDEDQEDSEGFEDSFEEEEEEEEDDD
Splooce       DSYMGLDQIKPLKLEVHEAKPVPENHPQWDTAIEGDEDQEDSEGFEDSFEEEEEEEEDDD

----------------------------------------------------------------------------------------------------

Q96DB9 (Uniprot)	versus
NM_144779#(-s-:19_F4410861986079) (Splooce)

For more details about the Alternative Splicing Event -> Link to Splooce page

Peptides that support the ASE (Splooce-specific):
DIQVPTR (MAXQUANT)

Alignment:
Uniprot       MSPSGRLCLLTIVGLILPTRGQTLKDTTSSSSADSTIMDIQVPTRAPDAVYTELQPTSPTPTWPADETPQPQTQTQQLEGTDGPLVTDPETHKSTKAAHP
Splooce       -------------------------------------MDIQVPTRAPDAVYTELQPTSPTPTWPADETPQPQTQTQQLEGTDGPLVTDPETHKSTKAAHP

Uniprot       TDDTTTLSERPSPSTDVQTDPQTLKPSGFHEDDPFFYDEHTLRKRGLLVAAVLFITGIIILTSGKCRQLSRLCRNRCR
Splooce       TDDTTTLSERPSPSTDVQTDPQTLKPSGFHEDDPFFYDEHTLRKRGLLVAAVLFITGIIILTSGKCRQLSRLCRNRCR

----------------------------------------------------------------------------------------------------

Q14444 (Uniprot)	versus
NM_005898#(-s-s-s-s-s-s-s-:11_C8481503078689) (Splooce)

For more details about the Alternative Splicing Event -> Link to Splooce page

Peptides that support the ASE (Splooce-specific):
QTVFNMNAPVPPVNEPETLK (MAXQUANT)

Alignment:
Uniprot       MPSATSHSGSGSKSSGPPPPSGSSGSEAAAGAGAAAPASQHPATGTGAVQTEAMKQILGVIDKKLRNLEKKKGKLDDYQERMNKGERLNQDQLDAVSKYQ
Splooce       ----------------------------------------------------------------------------------------------------

Uniprot       EVTNNLEFAKELQRSFMALSQDIQKTIKKTARREQLMREEAEQKRLKTVLELQYVLDKLGDDEVRTDLKQGLNGVPILSEEELSLLDEFYKLVDPERDMS
Splooce       ----------------------------------------------------------------------------------------------------

Uniprot       LRLNEQYEHASIHLWDLLEGKEKPVCGTTYKVLKEIVERVFQSNYFDSTHNHQNGLCEEEEAASAPAVEDQVPEAEPEPAEEYTEQSEVESTEYVNRQFM
Splooce       ----------------------------------------------------------------------------------------------------

Uniprot       AETQFTSGEKEQVDEWTVETVEVVNSLQQQPQAASPSVPEPHSLTPVAQADPLVRRQRVQDLMAQMQGPYNFIQDSMLDFENQTLDPAIVSAQPMNPTQN
Splooce       ----------------------------------------------------------------------------------------------------

Uniprot       MDMPQLVCPPVHSESRLAQPNQVPVQPEATQVPLVSSTSEGYTASQPLYQPSHATEQRPQKEPIDQIQATISLNTDQTTASSSLPAASQPQVFQAGTSKP
Splooce       ----------------------------------------------------------------------------------------------------

Uniprot       LHSSGINVNAAPFQSMQTVFNMNAPVPPVNEPETLKQQNQYQASYNQSFSSQPHQVEQTELQQEQLQTVVGTYHGSPDQSHQVTGNHQQPPQQNTGFPRS
Splooce       ---------------MQTVFNMNAPVPPVNEPETLKQQNQYQASYNQSFSSQPHQVEQTELQQEQLQTVVGTYHGSPDQSHQVTGNHQQPPQQNTGFPRS

Uniprot       NQPYYNSRGVSRGGSRGARGLMNGYRGPANGFRGGYDGYRPSFSNTPNSGYTQSQFSAPRDYSGYQRDGYQQNFKRGSGQSGPRGAPRGRGGPPRPNRGM
Splooce       NQPYYNSRGVSRGGSRGARGLMNGYRGPANGFRGGYDGYRPSFSNTPNSGYTQSQFSAPRDYSGYQRDGYQQNFKRGSGQSGPRGAPRGRGGPPRPNRGM

Uniprot       PQMNTQQVN
Splooce       PQMNTQQVN

----------------------------------------------------------------------------------------------------

Q8WWP7 (Uniprot)	versus
NM_130759#(f-:7_G5707218498253) (Splooce)

For more details about the Alternative Splicing Event -> Link to Splooce page

Peptides that support the ASE (Splooce-specific):
FGEDVLK (MAXQUANT)

Alignment:
Uniprot       MGGRKMATDEENVYGLEENAQSRQESTRRLILVGRTGAGKSATGNSILGQRRFFSRLGATSVTRACTTGSRRWDKCHVEVVDTPDIFSSQVSKTDPGCEE
Splooce       ----------------------------------------------------------------------------------------------------

Uniprot       RGHCYLLSAPGPHALLLVTQLGRFTAQDQQAVRQVRDMFGEDVLKWMVIVFTRKEDLAGGSLHDYVSNTENRALRELVAECGGRVCAFDNRATGREQEAQ
Splooce       -------------------------------------MFGEDVLKWMVIVFTRKEDLAGGSLHDYVSNTENRALRELVAECGGRVCAFDNRATGREQEAQ

Uniprot       VEQLLGMVEGLVLEHKGAHYSNEVYELAQVLRWAGPEERLRRVAERVAARVQRRPWGAWLSARLWKWLKSPRSWRLGLALLLGGALLFWVLLHRRWSEAV
Splooce       VEQLLGMVEGLVLEHKGAHYSNEVYELAQVLRWAGPEERLRRVAERVAARVQRRPWGAWLSARLWKWLKSPRSWRLGLALLLGGALLFWVLLHRRWSEAV

Uniprot       AEVGPD
Splooce       AEVGPD

----------------------------------------------------------------------------------------------------

P10809 (Uniprot)	versus
NM_199440#(f-:2_H4077504892702) (Splooce)

For more details about the Alternative Splicing Event -> Link to Splooce page

Peptides that support the ASE (Splooce-specific):
MLAVDAVLAELKK (PEAKS)
LAVDAVIAELKK (MAXQUANT)
MLAVDAVLAELK (PEAKS)
MLAVDAVIAELK (MAXQUANT)
LAVDAVLAELK (PEAKS)
LAVDAVIAELK (MAXQUANT)
LAVDAVLAELKK (PEAKS)
MLAVDAVIAELKK (MAXQUANT)

Alignment:
Uniprot       MLRLPTVFRQMRPVSRVLAPHLTRAYAKDVKFGADARALMLQGVDLLADAVAVTMGPKGRTVIIEQSWGSPKVTKDGVTVAKSIDLKDKYKNIGAKLVQD
Splooce       ----------------------------------------------------------------------------------------------------

Uniprot       VANNTNEEAGDGTTTATVLARSIAKEGFEKISKGANPVEIRRGVMLAVDAVIAELKKQSKPVTTPEEIAQVATISANGDKEIGNIISDAMKKVGRKGVIT
Splooce       --------------------------------------------MLAVDAVIAELKKQSKPVTTPEEIAQVATISANGDKEIGNIISDAMKKVGRKGVIT

Uniprot       VKDGKTLNDELEIIEGMKFDRGYISPYFINTSKGQKCEFQDAYVLLSEKKISSIQSIVPALEIANAHRKPLVIIAEDVDGEALSTLVLNRLKVGLQVVAV
Splooce       VKDGKTLNDELEIIEGMKFDRGYISPYFINTSKGQKCEFQDAYVLLSEKKISSIQSIVPALEIANAHRKPLVIIAEDVDGEALSTLVLNRLKVGLQVVAV

Uniprot       KAPGFGDNRKNQLKDMAIATGGAVFGEEGLTLNLEDVQPHDLGKVGEVIVTKDDAMLLKGKGDKAQIEKRIQEIIEQLDVTTSEYEKEKLNERLAKLSDG
Splooce       KAPGFGDNRKNQLKDMAIATGGAVFGEEGLTLNLEDVQPHDLGKVGEVIVTKDDAMLLKGKGDKAQIEKRIQEIIEQLDVTTSEYEKEKLNERLAKLSDG

Uniprot       VAVLKVGGTSDVEVNEKKDRVTDALNATRAAVEEGIVLGGGCALLRCIPALDSLTPANEDQKIGIEIIKRTLKIPAMTIAKNAGVEGSLIVEKIMQSSSE
Splooce       VAVLKVGGTSDVEVNEKKDRVTDALNATRAAVEEGIVLGGGCALLRCIPALDSLTPANEDQKIGIEIIKRTLKIPAMTIAKNAGVEGSLIVEKIMQSSSE

Uniprot       VGYDAMAGDFVNMVEKGIIDPTKVVRTALLDAAGVASLLTTAEVVVTEIPKEEKDPGMGAMGGMGGGMGGGMF
Splooce       VGYDAMAGDFVNMVEKGIIDPTKVVRTALLDAAGVASLLTTAEVVVTEIPKEEKDPGMGAMGGMGGGMGGGMF

----------------------------------------------------------------------------------------------------

Q9Y490 (Uniprot)	versus
NM_006289#(-s-s-s-s-s-:9_T6676158681582) (Splooce)

For more details about the Alternative Splicing Event -> Link to Splooce page

Peptides that support the ASE (Splooce-specific):
ASLNPYLGR (MAXQUANT)

Alignment:
Uniprot       MVALSLKISIGNVVKTMQFEPSTMVYDACRIIRERIPEAPAGPPSDFGLFLSDDDPKKGIWLEAGKALDYYMLRNGDTMEYRKKQRPLKIRMLDGTVKTI
Splooce       ----------------------------------------------------------------------------------------------------

Uniprot       MVDDSKTVTDMLMTICARIGITNHDEYSLVRELMEEKKEEITGTLRKDKTLLRDEKKMEKLKQKLHTDDELNWLDHGRTLREQGVEEHETLLLRRKFFYS
Splooce       ----------------------------------------------------------------------------------------------------

Uniprot       DQNVDSRDPVQLNLLYVQARDDILNGSHPVSFDKACEFAGFQCQIQFGPHNEQKHKAGFLDLKDFLPKEYVKQKGERKIFQAHKNCGQMSEIEAKVRYVK
Splooce       ----------------------------------------------------------------------------------------------------

Uniprot       LARSLKTYGVSFFLVKEKMKGKNKLVPRLLGITKECVMRVDEKTKEVIQEWNLTNIKRWAASPKSFTLDFGDYQDGYYSVQTTEGEQIAQLIAGYIDIIL
Splooce       ----------------------------------------------------------------------------------------------------

Uniprot       KKKKSKDHFGLEGDEESTMLEDSVSPKKSTVLQQQYNRVGKVEHGSVALPAIMRSGASGPENFQVGSMPPAQQQITSGQMHRGHMPPLTSAQQALTGTIN
Splooce       ----------------------------------------------------------------------------------------------------

Uniprot       SSMQAVQAAQATLDDFDTLPPLGQDAASKAWRKNKMDESKHEIHSQVDAITAGTASVVNLTAGDPAETDYTAVGCAVTTISSNLTEMSRGVKLLAALLED
Splooce       ----------------------------------------------------------------------------------------------------

Uniprot       EGGSGRPLLQAAKGLAGAVSELLRSAQPASAEPRQNLLQAAGNVGQASGELLQQIGESDTDPHFQDALMQLAKAVASAAAALVLKAKSVAQRTEDSGLQT
Splooce       ----------------------------------------------------------------------------------------------------
[truncated: 302,037 more chars]
