# Supplementary material for: Identification of rare alternative splicing events in MS/MS data reveals a significant fraction of alternative translation initiation sites
Source: PeerJ. 2014 Nov 13;2:e673. doi: 10.7717/peerj.673 (PMC4232841; doi:10.7717/peerj.673)
Supplement: File S3 — Peptides in yellow are specific to the Splooce database and do not exist in Uniprot as tryptic peptides. [file peerj-02-673-s003.zip › Supplementary_S3.html]

```
# Supplementary Material (S3)

List and sequence alignment of all identified events which do not characterize TIS changes.
  Peptides in yellow are specific to the Splooce database and do not exist in Uniprot as tryptic peptides.

----------------------------------------------------------------------------------------------------


Q9Y6Y8 (Uniprot)	versus
NM_007190#(-t:10_S9574156845867) (Splooce)

For more details about the Alternative Splicing Event -> Link to Splooce page

Peptides that support the ASE (Splooce-specific):
VANQIKEEEEKQVVEEK (MAXQUANT)

Alignment:
Uniprot       MAERKPNGGSGGASTSSSGTNLLFSSSATEFSFNVPFIPVTQASASPASLLLPGEDSTDVGEEDSFLGQTSIHTSAPQTFSYFSQVSSSSDPFGNIGQSP
Splooce       MAERKPNGGSGGASTSSSGTNLLFSSSATEFSFNVPFIPVTQASASPASLLLPGEDSTDVGEEDSFLGQTSIHTSAPQTFSYFSQVSSSSDPFGNIGQSP

Uniprot       LTTAATSVGQSGFPKPLTALPFTTGSQDVSNAFSPSISKAQPGAPPSSLMGINSYLPSQPSSLPPSYFGNQPQGIPQPGYNPYRHTPGSSRANPYIAPPQ
Splooce       LTTAATSVGQSGFPKPLTALPFTTGSQDVSNAFSPSISKAQPGAPPSSLMGINSYLPSQPSSLPPSYFGNQPQGIPQPGYNPYRHTPGSSRANPYIAPPQ

Uniprot       LQQCQTPGPPAHPPPSGPPVQMYQMPPGSLPPVPSSVQSPAQQQVPARPGAPSVQVPSPFLLQNQYEPVQPHWFYCKEVEYKQLWMPFSVFDSLNLEEIY
Splooce       LQQCQTPGPPAHPPPSGPPVQMYQMPPGSLPPVPSSVQSPAQQQVPARPGAPSVQVPSPFLLQNQYEPVQPHWFYCKEVEYKQLWMPFSVFDSLNLEEIY

Uniprot       NSVQPDPESVVLGTDGGRYDVYLYDRIRKAAYWEEEPAEVRRCTWFYKGDTDSRFIPYTEEFSEKLEAEYKKAVTTNQWHRRLEFPSGETIVMHNPKVIV
Splooce       NSVQPDPESVVLGTDGGRYDVYLYDRIRKAAYWEEEPAEVRRCTWFYKGDTDSRFIPYTEEFSEKLEAEYKKAVTTNQWHRRLEFPSGETIVMHNPKVIV

Uniprot       QFQPSSVPDEWGTTQDGQTRPRVVKRGIDDNLDEIPDGEMPQVDHLVFVVHGIGPVCDLRFRSIIECVDDFRVVSLKLLRTHFKKSLDDGKVSRVEFLPV
Splooce       QFQPSSVPDEWGTTQDGQTRPRVVKRGIDDNLDEIPDGEMPQVDHLVFVVHGIGPVCDLRFRSIIECVDDFRVVSLKLLRTHFKKSLDDGKVSRVEFLPV

Uniprot       HWHSSLGGDATGVDRNIKKITLPSIGRFRHFTNETLLDILFYNSPTYCQTIVEKVGMEINHLHALFMSRNPDFKGGVSVAGHSLGSLILFDILSNQKDLN
Splooce       HWHSSLGGDATGVDRNIKKITLPSIGRFRHFTNETLLDILFYNSPTYCQTIVEKVGMEINHLHALFMSRNPDFKGGVSVAGHSLGSLILFDILSNQKDLN

Uniprot       LSKCPGPLAVANGVVKQLHFQEKQMPEEPKLTLDESYDLVVENKEVLTLQETLEALSLSEYFSTFEKEKIDMESLLMCTVDDLKEMGIPLGPRKKIANFV
Splooce       LSKCPGPLAVANGVVKQLHFQEKQMPEEPKLTLDESYDLVVENKEVLTLQETLEALSLSEYFSTFEKEKIDMESLLMCTVDDLKEMGIPLGPRKKIANFV

Uniprot       EHKAAKLKKAASEKKAVAATSTKGQEQSAQKTKDMASLPSESNEPKRKLPVGACVSSVCVNYESFEVGAGQVSVAYNSLDFEPEIFFALGSPIAMFLTIR
Splooce       EHKAAKLKKAASEKKAVAATSTKGQEQSAQKTKDMASLPSESNEPKRKLPVGACVSSVCVNYESFEVGAGQVSVAYNSLDFEPEIFFALGSPIAMFLTIR

Uniprot       GVDRIDENYSLPTCKGFFNIYHPLDPVAYRLEPMIVPDLDLKAVLIPHHKGRKRLHLELKESLSRMGSDLKQGFISSLKSAWQTLNEFARAHTSSTQLQE
Splooce       GVDRIDENYSLPTCKGFFNIYHPLDPVAYRLEPMIVPDLDLKAVLIPHHKGRKRLHLELKESLSRMGSDLKQGFISSLKSAWQTLNEFARAHTSSTQLQE

Uniprot       ELEKVANQIKEEEEKQVVEAEKVVESPDFSKDEDYLGKVGMLNGGRRIDYVLQEKPIESFNEYLFALQSHLCYWESEDTALLLLKEIYRTMNISPEQPQH
Splooce       ELEKVANQIKEEEEKQVVE-EKVVESPDFSKDEDYLGKVGMLNGGRRIDYVLQEKPIESFNEYLFALQSHLCYWESEDTALLLLKEIYRTMNISPEQPQH

----------------------------------------------------------------------------------------------------

Q8WX93 (Uniprot)	versus
NM_016081#(-s-s-s-s-s-s-:4_P1095886566346) (Splooce)

For more details about the Alternative Splicing Event -> Link to Splooce page

Peptides that support the ASE (Splooce-specific):
FLDINSEPHQENK (MAXQUANT)

Alignment:
Uniprot       MSGTSSHESFYDSLSDMQEESKNTDFFPGLSAFLSQEEINKSLDLARRAIADSETEDFDSEKEISQIFSTSPASLCEHPSHKETKLGEHASRRPQDNRST
Splooce       MSGTSSHESFYDSLSDMQEESKNTDFFPGLSAFLSQEEINKSLDLARRAIADSETEDFDSEKEISQIFSTSPASLCEHPSHKETKLGEHASRRPQDNRST

Uniprot       PVQPLAEKQTKSISSPVSKRKPAMSPLLTRPSYIRSLRKAEKRGAKTPSTNVKPKTPHQRKGGPQSQLCDKAANLIEELTSIFKAAKPRNRSPNGESSSP
Splooce       PVQPLAEKQTKSISSPVSKRKPAMSPLLTRPSYIRSLRKAEKRGAKTPSTNVKPKTPHQRKGGPQSQLCDKAANLIEELTSIFKAAKPRNRSPNGESSSP

Uniprot       DSGYLSPKNQPSALLSASASQSPMEDQGEMEREVKSPGARHCYQDNQDLAVPHNRKSHPQPHSALHFPAAPRFIQKLRSQEVAEGSRVYLECRVTGNPTP
Splooce       DSGYLSPKNQPSALLSASASQSPMEDQGEMEREVKSPGARHCYQDNQDLAVPHNRKSHPQPHSALHFPAAPRFIQKLRSQEVAEGSRVYLECRVTGNPTP

Uniprot       RVRWFCEGKELHNTPDIQIHCEGGDLHTLIIAEAFEDDTGRYTCLATNPSGSDTTSAEVFIEGASSTDSDSESLAFKSRAGAMPQAQKKTTSVSLTIGSS
Splooce       RVRWFCEGKELHNTPDIQIHCEGGDLHTLIIAEAFEDDTGRYTCLATNPSGSDTTSAEVFIEGASSTDSDSESLAFKSRAGAMPQAQKKTTSVSLTIGSS

Uniprot       SPKTGVTTAVIQPLSVPVQQVHSPTSYLCRPDGTTTAYFPPVFTKELQNTAVAEGQVVVLECRVRGAPPLQVQWFRQGSEIQDSPDFRILQKKPRSTAEP
Splooce       SPKTGVTTAVIQPLSVPVQQVHSPTSYLCRPDGTTTAYFPPVFTKELQNTAVAEGQVVVLECRVRGAPPLQVQWFRQGSEIQDSPDFRILQKKPRSTAEP

Uniprot       EEICTLVIAETFPEDAGIFTCSARNDYGSATSTAQLVVTSANTENCSYESMGESNNDHFQHFPPPPPILETSSLELASKKPSEIQQVNNPELGLSRAALQ
Splooce       EEICTLVIAETFPEDAGIFTCSARNDYGSATSTAQLVVTSANTENCSYESMGESNNDHFQHFPPPPPILETSSLELASKKPSEIQQVNNPELGLSRAALQ

Uniprot       MQFNAAERETNGVHPSRGVNGLINGKANSNKSLPTPAVLLSPTKEPPPLLAKPKLGFPKKASRTARIASDEEIQGTKDAVIQDLERKLRFKEDLLNNGQP
Splooce       MQFNAAERETNGVHPSRGVNGLINGKANSNKSLPTPAVLLSPTKEPPPLLAKPKLGFPKKASRTARIASDEEIQGTKDAVIQDLERKLRFKEDLLNNGQP

Uniprot       RLTYEERMARRLLGADSATVFNIQEPEEETANQEYKVSSCEQRLISEIEYRLERSPVDESGDEVQYGDVPVENGMAPFFEMKLKHYKIFEGMPVTFTCRV
Splooce       RLTYEERMARRLLGADSATVFNIQEPEEETANQEYKVSSCEQRLISEIEYRLERSPVDESGDEVQYGDVPVENGMAPFFEMKLKHYKIFEGMPVTFTCRV

Uniprot       AGNPKPKIYWFKDGKQISPKSDHYTIQRDLDGTCSLHTTASTLDDDGNYTIMAANPQGRISCTGRLMVQAVNQRGRSPRSPSGHPHVRRPRSRSRDSGDE
Splooce       AGNPKPKFLDIN----------------------------------------------------------------------------------------

Uniprot       NEPIQERFFRPHFLQAPGDLTVQEGKLCRMDCKVSGLPTPDLSWQLDGKPVRPDSAHKMLVRENGVHSLIIEPVTSRDAGIYTCIATNRAGQNSFSLELV
Splooce       SEPHQENKYPTQWHQQ--------------------------SQSTKPKKVRPSASRYAALSDQGLD=================================

Uniprot       VAAKEAHKPPVFIEKLQNTGVADGYPVRLECRVLGVPPPQIFWKKENESLTHSTDRVSMHQDNHGYICLLIQGATKEDAGWYTVSAKNEAGIVSCTARLD
Splooce       ==IKAAFQP=============EANPSHLT===============LNTALVESEDL--------------------------------------------

Uniprot       VYISRH
Splooce       ------

----------------------------------------------------------------------------------------------------

Q12816 (Uniprot)	versus
NM_001039705#(-s-s-s-:X_T4358705065184) (Splooce)

For more details about the Alternative Splicing Event -> Link to Splooce page

Peptides that support the ASE (Splooce-specific):
SLNEQATLWR (MAXQUANT)

Alignment:
Uniprot       MDRRNDYGYRVPLFQGPLPPPGSLGLPFPPDIQTETTEEDSVLLMHTLLAATKDSLAMDPPVVNRPKKSKTKKAPIKTITKAAPAAPPVPAANEIATNKP
Splooce       ----------------------------------------------------------------------------------------------------

Uniprot       KITWQALNLPVITQISQALPTTEVTNTQASSVTAQPKKANKMKRVTAKAAQGSQSPTGHEGGTIQLKSPLQVLKLPVISQNIHAPIANESASSQALITSI
Splooce       ----------------------------------------------------------------------------------------------------

Uniprot       KPKKASKAKKAANKAIASATEVSLAATATHTATTQGQITNETASIHTTAASIRTKKASKARKTIAKVINTDTEHIEALNVTDAATRQIEASVVAIRPKKS
Splooce       ----------------------------------------------------------------------------------------------------

Uniprot       KGKKAASRGPNSVSEISEAPLATQIVTNQALAATLRVKRGSRARKAATKARATESQTPNADQGAQAKIASAQTNVSALETQVAAAVQALADDYLAQLSLE
Splooce       ----------------------------------------------------------------------------------------------------

Uniprot       PTTRTRGKRNRKSKHLNGDERSGSNYRRIPWGRRPAPPRDVAILQERANKLVKYLLVKDQTKIPIKRSDMLRDVIQEYDEYFPEIIERASYTLEKMFRVN
Splooce       ------------------------------------------------------------------------------------------MSSKNMMNIS

Uniprot       LKEIDKQSSLYILISTQESSAGILGTTKDTPKLGLLMVILSVIFMNGNKASEAVIWEVLRKLGLRPGVRHSLFGEVRKLITDEFVKQKYLEYKRVPNSRP
Splooce       QKSLNEQATLWR------------------------------------------------------RVRHSLFGEVRKLITDEFVKQKYLEYKRVPNSRP

Uniprot       PEYEFFWGLRSYHETSKMKVLKFACRVQKKDPKDWAVQYREAVEMEVQAAAVAVAEAEARAEARAQMGIGEEAVAGPWNWDDMDIDCLTREELGDDAQAW
Splooce       PEYEFFWGLRSYHETSKMKVLKFACRVQKKDPKDWAVQYREAVEMEVQAAAVAVAEAEARAEARAQMGIGEEAVAGPWNWDDMDIDCLTREELGDDAQAW

Uniprot       SRFSFEIEARAQENADASTNVNFSRGASTRAGFSDGASISFNGAPSSSGGFSGGPGITFGVAPSTSASFSNTASISFGGTLSTSSSFSSAASISFGCAHS
Splooce       SRFSFEIEARAQENADASTNVNFSRGASTRAGFSDGASISFNGAPSSSGGFSGGPGITFGVAPSTSASFSNTASISFGGTLSTSSSFSSAASISFGCAHS

Uniprot       TSTSFSSEASISFGGMPCTSASFSGGVSSSFSGPLSTSATFSGGASSGFGGTLSTTAGFSGVLSTSTSFGSAPTTSTVFSSALSTSTGFGGILSTSVCFG
Splooce       TSTSFSSEASISFGGMPCTSASFSGGVSSSFSGPLSTSATFSGGASSGFGGTLSTTAGFSGVLSTSTSFGSAPTTSTVFSSALSTSTGFGGILSTSVCFG

Uniprot       GSPSSSGSFGGTLSTSICFGGSPCTSTGFGGTLSTSVSFGGSSSTSANFGGTLSTSICFDGSPSTGAGFGGALNTSASFGSVLNTSTGFGGAMSTSADFG
Splooce       GSPSSSGSFGGTLSTSICFGGSPCTSTGFGGTLSTSVSFGGSSSTSANFGGTLSTSICFDGSPSTGAGFGGALNTSASFGSVLNTSTGFGGAMSTSADFG

Uniprot       GTLSTSVCFGGSPGTSVSFGSALNTNAGYGGAVSTNTDFGGTLSTSVCFGGSPSTSAGFGGALNTNASFGCAVSTSASFSGAVSTSACFSGAPITNPGFG
Splooce       GTLSTSVCFGGSPGTSVSFGSALNTNAGYGGAVSTNTDFGGTLSTSVCFGGSPSTSAGFGGALNTNASFGCAVSTSASFSGAVSTSACFSGAPITNPGFG

Uniprot       GAFSTSAGFGGALSTAADFGGTPSNSIGFGAAPSTSVSFGGAHGTSLCFGGAPSTSLCFGSASNTNLCFGGPPSTSACFSGATSPSFCDGPSTSTGFSFG
Splooce       GAFSTSAGFGGALSTAADFGGTPSNSIGFGAAPSTSVSFGGAHGTSLCFGGAPSTSLCFGSASNTNLCFGGPPSTSACFSGATSPSFCDGPSTSTGFSFG

Uniprot       NGLSTNAGFGGGLNTSAGFGGGLGTSAGFSGGLSTSSGFDGGLGTSAGFGGGPGTSTGFGGGLGTSAGFSGGLGTSAGFGGGLVTSDGFGGGLGTNASFG
Splooce       NGLSTNAGFGGGLNTSAGFGGGLGTSAGFSGGLSTSSGFDGGLGTSAGFGGGPGTSTGFGGGLGTSAGFSGGLGTSAGFGGGLVTSDGFGGGLGTNASFG

Uniprot       STLGTSAGFSGGLSTSDGFGSRPNASFDRGLSTIIGFGSGSNTSTGFTGEPSTSTGFSSGPSSIVGFSGGPSTGVGFCSGPSTSGFSGGPSTGAGFGGGP
Splooce       STLGTSAGFSGGLSTSDGFGSRPNASFDRGLSTIIGFGSGSNTSTGFTGEPSTSTGFSSGPSSIVGFSGGPSTGVGFCSGPSTSGFSGGPSTGAGFGGGP

Uniprot       NTGAGFGGGPSTSAGFGSGAASLGACGFSYG
Splooce       NTGAGFGGGPSTSAGFGSGAASLGACGFSYG

----------------------------------------------------------------------------------------------------

P27816 (Uniprot)	versus
NM_001134364#(-s-:3_M9130409277669) (Splooce)

For more details about the Alternative Splicing Event -> Link to Splooce page

Peptides that support the ASE (Splooce-specific):
DSYVPLELAK (MAXQUANT)

Alignment:
Uniprot       MADLSLADALTEPSPDIEGEIKRDFIATLEAEAFDDVVGETVGKTDYIPLLDVDEKTGNSESKKKPCSETSQIEDTPSSKPTLLANGGHGVEGSDTTGSP
Splooce       MADLSLADALTEPSPDIEGEIKRDFIATLEAEAFDDVVGETVGKTDYIPLLDVDEKTGNSESKKKPCSETSQIEDTPSSKPTLLANGGHGVEGSDTTGSP

Uniprot       TEFLEEKMAYQEYPNSQNWPEDTNFCFQPEQVVDPIQTDPFKMYHDDDLADLVFPSSATADTSIFAGQNDPLKDSYGMSPCNTAVVPQGWSVEALNSPHS
Splooce       TEFLEEKMAYQEYPNSQNWPEDTNFCFQPEQVVDPIQTDPFKMYHDDDLADLVFPSSATADTSIFAGQNDPLKDSY------------------------

Uniprot       ESFVSPEAVAEPPQPTAVPLELAKEIEMASEERPPAQALEIMMGLKTTDMAPSKETEMALAKDMALATKTEVALAKDMESPTKLDVTLAKDMQPSMESDM
Splooce       -----------------VPLELAKEIEMASEERPPAQALEIMMGLKTTDMAPSKETEMALAKDMALATKTEVALAKDMESPTKLDVTLAKDMQPSMESDM

Uniprot       ALVKDMELPTEKEVALVKDVRWPTETDVSSAKNVVLPTETEVAPAKDVTLLKETERASPIKMDLAPSKDMGPPKENKKETERASPIKMDLAPSKDMGPPK
Splooce       ALVKDMELPTEKEVALVKDVRWPTETDVSSAKNVVLPTETEVAPAKDVTLLKETERASPIKMDLAPSKDMGPPKENKKETERASPIKMDLAPSKDMGPPK

Uniprot       ENKIVPAKDLVLLSEIEVAQANDIISSTEISSAEKVALSSETEVALARDMTLPPETNVILTKDKALPLEAEVAPVKDMAQLPETEIAPAKDVAPSTVKEV
Splooce       ENKIVPAKDLVLLSEIEVAQANDIISSTEISSAEKVALSSETEVALARDMTLPPETNVILTKDKALPLEAEVAPVKDMAQLPETEIAPAKDVAPSTVKEV

Uniprot       GLLKDMSPLSETEMALGKDVTPPPETEVVLIKNVCLPPEMEVALTEDQVPALKTEAPLAKDGVLTLANNVTPAKDVPPLSETEATPVPIKDMEIAQTQKG
Splooce       GLLKDMSPLSETEMALGKDVTPPPETEVVLIKNVCLPPEMEVALTEDQVPALKTEAPLAKDGVLTLANNVTPAKDVPPLSETEATPVPIKDMEIAQTQKG

Uniprot       ISEDSHLESLQDVGQSAAPTFMISPETVTGTGKKCSLPAEEDSVLEKLGERKPCNSQPSELSSETSGIARPEEGRPVVSGTGNDITTPPNKELPPSPEKK
Splooce       ISEDSHLESLQDVGQSAAPTFMISPETVTGTGKKCSLPAEEDSVLEKLGERKPCNSQPSELSSETSGIARPEEGRPVVSGTGNDITTPPNKELPPSPEKK

Uniprot       TKPLATTQPAKTSTSKAKTQPTSLPKQPAPTTIGGLNKKPMSLASGLVPAAPPKRPAVASARPSILPSKDVKPKPIADAKAPEKRASPSKPASAPASRSG
Splooce       TKPLATTQPAKTSTSKAKTQPTSLPKQPAPTTIGGLNKKPMSLASGLVPAAPPKRPAVASARPSILPSKDVKPKPIADAKAPEKRASPSKPASAPASRSG

Uniprot       SKSTQTVAKTTTAAAVASTGPSSRSPSTLLPKKPTAIKTEGKPAEVKKMTAKSVPADLSRPKSTSTSSMKKTTTLSGTAPAAGVVPSRVKATPMPSRPST
Splooce       SKSTQTVAKTTTAAAVASTGPSSRSPSTLLPKKPTAIKTEGKPAEVKKMTAKSVPADLSRPKSTSTSSMKKTTTLSGTAPAAGVVPSRVKATPMPSRPST

Uniprot       TPFIDKKPTSAKPSSTTPRLSRLATNTSAPDLKNVRSKVGSTENIKHQPGGGRAKVEKKTEAAATTRKPESNAVTKTAGPIASAQKQPAGKVQIVSKKVS
Splooce       TPFIDKKPTSAKPSSTTPRLSRLATNTSAPDLKNVRSKVGSTENIKHQPGGGRAKVEKKTEAAATTRKPESNAVTKTAGPIASAQKQPAGKVQIVSKKVS

Uniprot       YSHIQSKCGSKDNIKHVPGGGNVQIQNKKVDISKVSSKCGSKANIKHKPGGGDVKIESQKLNFKEKAQAKVGSLDNVGHLPAGGAVKIETYRLTFRANAR
Splooce       YSHIQSKCGSKDNIKHVPGGGNVQIQNKKVDISKVSSKCGSKANIKHKPGGGDVKIESQKLNFKEKAQAKVGSLDNVGHLPAGGAVKIETYRLTFRANAR

Uniprot       ARTDHGADIVSRPPHFPGGPNSGSRVLGPLSRAVH
Splooce       ARTDHGADIVSRPPHFPGGPNSGSRVLGPLSRAVH

----------------------------------------------------------------------------------------------------

P10909 (Uniprot)	versus
NM_203339#(f-:8_C2537502576945) (Splooce)

For more details about the Alternative Splicing Event -> Link to Splooce page

Peptides that support the ASE (Splooce-specific):
YDALNETR (MAXQUANT)

Alignment:
Uniprot       MMKTLLLFVGLLLTWESGQVLGDQTVSDNELQEMSNQGSKYVNKEIQNAVNGVKQIKTLIEKTNEERKTLLSNLEEAKKKKEDALNETRESETKLKELPG
Splooce       MMKTLLLFVGLLLTWESGQVLGDQTVSDNELQEMSNQGSKY-----------------------------------------DALNETRESETKLKELPG

Uniprot       VCNETMMALWEECKPCLKQTCMKFYARVCRSGSGLVGRQLEEFLNQSSPFYFWMNGDRIDSLLENDRQQTHMLDVMQDHFSRASSIIDELFQDRFFTREP
Splooce       VCNETMMALWEECKPCLKQTCMKFYARVCRSGSGLVGRQLEEFLNQSSPFYFWMNGDRIDSLLENDRQQTHMLDVMQDHFSRASSIIDELFQDRFFTREP

Uniprot       QDTYHYLPFSLPHRRPHFFFPKSRIVRSLMPFSPYEPLNFHAMFQPFLEMIHEAQQAMDIHFHSPAFQHPPTEFIREGDDDRTVCREIRHNSTGCLRMKD
Splooce       QDTYHYLPFSLPHRRPHFFFPKSRIVRSLMPFSPYEPLNFHAMFQPFLEMIHEAQQAMDIHFHSPAFQHPPTEFIREGDDDRTVCREIRHNSTGCLRMKD

Uniprot       QCDKCREILSVDCSTNNPSQAKLRRELDESLQVAERLTRKYNELLKSYQWKMLNTSSLLEQLNEQFNWVSRLANLTQGEDQYYLRVTTVASHTSDSDVPS
Splooce       QCDKCREILSVDCSTNNPSQAKLRRELDESLQVAERLTRKYNELLKSYQWKMLNTSSLLEQLNEQFNWVSRLANLTQGEDQYYLRVTTVASHTSDSDVPS

Uniprot       GVTEVVVKLFDSDPITVTVPVEVSRKNPKFMETVAEKALQEYRKKHREE
Splooce       GVTEVVVKLFDSDPITVTVPVEVSRKNPKFMETVAEKALQEYRKKHREE

----------------------------------------------------------------------------------------------------

P11388 (Uniprot)	versus
NM_001067#(-t:17_T1613947974844) (Splooce)

For more details about the Alternative Splicing Event -> Link to Splooce page

Peptides that support the ASE (Splooce-specific):
YLLDMPLWYLTK (MAXQUANT)

Alignment:
Uniprot       MEVSPLQPVNENMQVNKIKKNEDAKKRLSVERIYQKKTQLEHILLRPDTYIGSVELVTQQMWVYDEDVGINYREVTFVPGLYKIFDEILVNAADNKQRDP
Splooce       MEVSPLQPVNENMQVNKIKKNEDAKKRLSVERIYQKKTQLEHILLRPDTYIGSVELVTQQMWVYDEDVGINYREVTFVPGLYKIFDEILVNAADNKQRDP

Uniprot       KMSCIRVTIDPENNLISIWNNGKGIPVVEHKVEKMYVPALIFGQLLTSSNYDDDEKKVTGGRNGYGAKLCNIFSTKFTVETASREYKKMFKQTWMDNMGR
Splooce       KMSCIRVTIDPENNLISIWNNGKGIPVVEHKVEKMYVPALIFGQLLTSSNYDDDEKKVTGGRNGYGAKLCNIFSTKFTVETASREYKKMFKQTWMDNMGR

Uniprot       AGEMELKPFNGEDYTCITFQPDLSKFKMQSLDKDIVALMVRRAYDIAGSTKDVKVFLNGNKLPVKGFRSYVDMYLKDKLDETGNSLKVIHEQVNHRWEVC
Splooce       AGEMELKPFNGEDYTCITFQPDLSKFKMQSLDKDIVALMVRRAYDIAGSTKDVKVFLNGNKLPVKGFRSYVDMYLKDKLDETGNSLKVIHEQVNHRWEVC

Uniprot       LTMSEKGFQQISFVNSIATSKGGRHVDYVADQIVTKLVDVVKKKNKGGVAVKAHQVKNHMWIFVNALIENPTFDSQTKENMTLQPKSFGSTCQLSEKFIK
Splooce       LTMSEKGFQQISFVNSIATSKGGRHVDYVADQIVTKLVDVVKKKNKGGVAVKAHQVKNHMWIFVNALIENPTFDSQTKENMTLQPKSFGSTCQLSEKFIK

Uniprot       AAIGCGIVESILNWVKFKAQVQLNKKCSAVKHNRIKGIPKLDDANDAGGRNSTECTLILTEGDSAKTLAVSGLGVVGRDKYGVFPLRGKILNVREASHKQ
Splooce       AAIGCGIVESILNWVKFKAQVQLNKKCSAVKHNRIKGIPKLDDANDAGGRNSTECTLILTEGDSAKTLAVSGLGVVGRDKYGVFPLRGKILNVREASHKQ

Uniprot       IMENAEINNIIKIVGLQYKKNYEDEDSLKTLRYGKIMIMTDQDQDGSHIKGLLINFIHHNWPSLLRHRFLEEFITPIVKVSKNKQEMAFYSLPEFEEWKS
Splooce       IMENAEINNIIKIVGLQYKKNYEDEDSLKTLRYGKIMIMTDQDQDGSHIKGLLINFIHHNWPSLLRHRFLEEFITPIVKVSKNKQEMAFYSLPEFEEWKS

Uniprot       STPNHKKWKVKYYKGLGTSTSKEAKEYFADMKRHRIQFKYSGPEDDAAISLAFSKKQIDDRKEWLTNFMEDRRQRKLLGLPEDYLYGQTTTYLTYNDFIN
Splooce       STPNHKKWKVKYYKGLGTSTSKEAKEYFADMKRHRIQFKYSGPEDDAAISLAFSKKQIDDRKEWLTNFMEDRRQRKLLGLPEDYLYGQTTTYLTYNDFIN

Uniprot       KELILFSNSDNERSIPSMVDGLKPGQRKVLFTCFKRNDKREVKVAQLAGSVAEMSSYHHGEMSLMMTIINLAQNFVGSNNLNLLQPIGQFGTRLHGGKDS
Splooce       KELILFSNSDNERSIPSMVDGLKPGQRKVLFTCFKRNDKREVKVAQLAGSVAEMSSYHHGEMSLMMTIINLAQNFVGSNNLNLLQPIGQFGTRLHGGKDS

Uniprot       ASPRYIFTMLSSLARLLFPPKDDHTLKFLYDDNQRVEPEWYIPIIPMVLINGAEGIGTGWSCKIPNFDVREIVNNIRRLMDGEEPLPMLPSYKNFKGTIE
Splooce       ASPRYIFTMLSSLARLLFPPKDDHTLKFLYDDNQRVEPEWYIPIIPMVLINGAEGIGTGWSCKIPNFDVREIVNNIRRLMDGEEPLPMLPSYKNFKGTIE

Uniprot       ELAPNQYVISGEVAILNSTTIEISELPVRTWTQTYKEQVLEPMLNGTEKTPPLITDYREYHTDTTVKFVVKMTEEKLAEAERVGLHKVFKLQTSLTCNSM
Splooce       ELAPNQYVISGEVAILNSTTIEISELPVRTWTQTYKEQVLEPMLNGTEKTPPLITDYREYHTDTTVKFVVKMTEEKLAEAERVGLHKVFKLQTSLTCNSM

Uniprot       VLFDHVGCLKKYDTVLDILRDFFELRLKYYGLRKEWLLGMLGAESAKLNNQARFILEKIDGKIIIENKPKKELIKVLIQRGYDSDPVKAWKEAQQKVPDE
Splooce       VLFDHVGCLKKYDTVLDILRDFFELRLKYYGLRKEWLLGMLGAESAKLNNQARFILEKIDGKIIIENKPKKELIKVLIQRGYDSDPVKAWKEAQQK----

Uniprot       EENEESDNEKETEKSDSVTDSGPTFNYLLDMPLWYLTKEKKDELCRLRNEKEQELDTLKRKSPSDLWKEDLATFIEELEAVEAKEKQDEQVGLPGKGGKA
Splooce       --------------------------YLLDMPLWYLTKEKKDELCRLRNEKEQELDTLKRKSPSDLWKEDLATFIEELEAVEAKEKQDEQVGLPGKGGKA

Uniprot       KGKKTQMAEVLPSPRGQRVIPRITIEMKAEAEKKNKKKIKNENTEGSPQEDGVELEGLKQRLEKKQKREPGTKTKKQTTLAFKPIKKGKKRNPWSDSESD
Splooce       KGKKTQMAEVLPSPRGQRVIPRITIEMKAEAEKKNKKKIKNENTEGSPQEDGVELEGLKQRLEKKQKREPGTKTKKQTTLAFKPIKKGKKRNPWSDSESD

Uniprot       RSSDESNFDVPPRETEPRRAATKTKFTMDLDSDEDFSDFDEKTDDEDFVPSDASPPKTKTSPKLSNKELKPQKSVVSDLEADDVKGSVPLSSSPPATHFP
Splooce       RSSDESNFDVPPRETEPRRAATKTKFTMDLDSDEDFSDFDEKTDDEDFVPSDASPPKTKTSPKLSNKELKPQKSVVSDLEADDVKGSVPLSSSPPATHFP

Uniprot       DETEITNPVPKKNVTVKKTAAKSQSSTSTTGAKKRAAPKGTKRDPALNSGVSQKPDPAKTKNRRKRKPSTSDDSDSNFEKIVSKAVTSKKSKGESDDFHM
Splooce       DETEITNPVPKKNVTVKKTAAKSQSSTSTTGAKKRAAPKGTKRDPALNSGVSQKPDPAKTKNRRKRKPSTSDDSDSNFEKIVSKAVTSKKSKGESDDFHM

Uniprot       DFDSAVAPRAKSVRAKKPIKYLEESDEDDLF
Splooce       DFDSAVAPRAKSVRAKKPIKYLEESDEDDLF

----------------------------------------------------------------------------------------------------

Q9UPT6 (Uniprot)	versus
NM_015133#(-s-s-s-s-s-s-s-s-:16_M3098230693658) (Splooce)

For more details about the Alternative Splicing Event -> Link to Splooce page

Peptides that support the ASE (Splooce-specific):
PLEFFPDEHVK (MAXQUANT)

Alignment:
Uniprot       MMEIQMDEGGGVVVYQDDYCSGSVMSERVSGLAGSIYREFERLIHCYDEEVVKELMPLVVNVLENLDSVLSENQEHEVELELLREDNEQLLTQYEREKAL
Splooce       MMEIQMDEGGGVVVYQDDYCSGSVMSERVSGLAGSIYREFERLIHCYDEEVVKELMPLVVNVLENLDSVLSENQEHEVELELLREDNEQLLTQYEREKAL

Uniprot       RRQAEEKFIEFEDALEQEKKELQIQVEHYEFQTRQLELKAKNYADQISRLEERESEMKKEYNALHQRHTEMIQTYVEHIERSKMQQVGGNSQTESSLPGR
Splooce       RRQAEEKFIEFEDALEQEKKELQIQVEHYEFQTRQLELKAKNYADQISRLEERESEMKKEYNALHQRHTEMIQTYVEHIERSKMQQVGGNSQTESSLPGR

Uniprot       RKERPTSLNVFPLADGTVRAQIGGKLVPAGDHWHLSDLGQLQSSSSYQCPQDEMSESGQSSAAATPSTTGTKSNTPTSSVPSAAVTPLNESLQPLGDYGV
Splooce       RKERPTSLNVFPLADGTVRAQIGGKLVPAGDHWHLSDLGQLQSSSSYQCPQDEMSESGQSSAAATPSTTGTKSNTPTSSVPSAAVTPLNESLQPLGDYGV

Uniprot       GSKNSKRAREKRDSRNMEVQVTQEMRNVSIGMGSSDEWSDVQDIIDSTPELDMCPETRLDRTGSSPTQGIVNKAFGINTDSLYHELSTAGSEVIGDVDEG
Splooce       GSKNSKRAREKRDSRNMEVQVTQEMRNVSIGMGSSDEWSDVQDIIDSTPELDMCPETRLDRTGSSPTQGIVNKAFGINTDSLYHELSTAGSEVIGDVDEG

Uniprot       ADLLGDGSLVRDDFFGMGKEVGNLLLENSQLLETKNALNVVKNDLIAKVDQLSGEQEVLRGELEAAKQAKVKLENRIKELEEELKRVKSEAIIARREPKE
Splooce       ADLLGDGSLVRDDFFGMGKEVGNLLLENSQLLETKNALNVVKNDLIAKVDQLSGEQEVLRGELEAAKQAKVKLENRIKELEEELKRVKSEAIIARREPKE

Uniprot       EAEDVSSYLCTESDKIPMAQRRRFTRVEMARVLMERNQYKERLMELQEAVRWTEMIRASREHPSVQEKKKSTIWQFFSRLFSSSSSPPPAKRPYPSVNIH
Splooce       EAEDVSSYLCTESDKIPMAQRRRFTRVEMARVLMERNQYKERLMELQEAVRWTEMIRASREHPSVQEKKKSTIWQFFSRLFSSSSSPPPAKRPYPSVNIH

Uniprot       YKSPTTAGFSQRRNHAMCPISAGSRPLEFFPDDDCTSSARREQKREQYRQVREHVRNDDGRLQACGWSLPAKYKQLSPNGGQEDTRMKNVPVPVYCRPLV
Splooce       YKSPTTAGFSQRRNHAMCPISAGSRPLEFFPDE-------------------------------------------------------------------

Uniprot       EKDPTMKLWCAAGVNLSGWRPNEDDAGNGVKPAPGRDPLTCDREGDGEPKSAHTSPEKKKAKELPEMDATSSRVWILTSTLTTSKVVIIDANQPGTVVDQ
Splooce       ----------------------------------------------------------------------------------------------------

Uniprot       FTVCNAHVLCISSIPAASDSDYPPGEMFLDSDVNPEDPGADGVLAGITLVGCATRCNVPRSNCSSRGDTPVLDKGQGEVATIANGKVNPSQSTEEATEAT
Splooce       ----------------------------------------------------------------------------------------------------

Uniprot       EVPDPGPSEPETATLRPGPLTEHVFTDPAPTPSSGPQPGSENGPEPDSSSTRPEPEPSGDPTGAGSSAAPTMWLGAQNGWLYVHSAVANWKKCLHSIKLK
Splooce       ----------------------------------------------------------------------------------------------------

Uniprot       DSVLSLVHVKGRVLVALADGTLAIFHRGEDGQWDLSNYHLMDLGHPHHSIRCMAVVYDRVWCGYKNKVHVIQPKTMQIEKSFDAHPRRESQVRQLAWIGD
Splooce       -------HVKGRVLVALADGTLAIFHRGEDGQWDLSNYHLMDLGHPHHSIRCMAVVYDRVWCGYKNKVHVIQPKTMQIEKSFDAHPRRESQVRQLAWIGD

Uniprot       GVWVSIRLDSTLRLYHAHTHQHLQDVDIEPYVSKMLGTGKLGFSFVRITALLVAGSRLWVGTGNGVVISIPLTETVVLHRGQLLGLRANKTSPTSGEGAR
Splooce       GVWVSIRLDSTLRLYHAHTHQHLQDVDIEPYVSKMLGTGKLGFSFVRITALLVAGSRLWVGTGNGVVISIPLTETVVLHRGQLLGLRANKTSPTSGEGAR

Uniprot       PGGIIHVYGDDSSDRAASSFIPYCSMAQAQLCFHGHRDAVKFFVSVPGNVLATLNGSVLDSPAEGPGPAAPASEVEGQKLRNVLVLSGGEGYIDFRIGDG
Splooce       PGGIIHVYGDDSSDRAASSFIPYCSMAQAQLCFHGHRDAVKFFVSVPGNVLATLNGSVLDSPAEGPGPAAPASEVEGQKLRNVLVLSGGEGYIDFRIGDG

Uniprot       EDDETEEGAGDMSQVKPVLSKAERSHIIVWQVSYTPE
Splooce       EDDETEEGAGDMSQVKPVLSKAERSHIIVWQVSYTPE

----------------------------------------------------------------------------------------------------

Q9UHX1 (Uniprot)	versus
NM_078480#(r:8_P314355280962) (Splooce)

For more details about the Alternative Splicing Event -> Link to Splooce page

Peptides that support the ASE (Splooce-specific):
KPFVAGPGLTGLSISPPLPQVGR (MAXQUANT)

Alignment:
Uniprot       MATATIALQVNGQQGGGSEPAAAAAVVAAGDKWKPPQGTDSIKMENGQSTAAKLGLPPLTPEQQEALQKAKKYAMEQSIKSVLVKQTIAHQQQQLTNLQM
Splooce       MATATIALQVNGQQGGGSEPAAAAAVVAAGDKWKPPQGTDSIKMENGQSTAAKLGLPPLTPEQQEALQKAKKYAMEQSIKSVLVKQTIAHQQQQLTNLQM

Uniprot       AAVTMGFGDPLSPLQSMAAQRQRALAIMCRVYVGSIYYELGEDTIRQAFAPFGPIKSIDMSWDSVTMKHKGFAFVEYEVPEAAQLALEQMNSVMLGGRNI
Splooce       AAVTMGFGDPLSPLQSMAAQRQRALAIMCRVYVGSIYYELGEDTIRQAFAPFGPIKSIDMSWDSVTMKHKGFAFVEYEVPEAAQLALEQMNSVMLGGRNI

Uniprot       KV=======================================GRPSNIGQAQPIIDQLAEEARAFNRIYVASVHQDLSDDDIKSVFEAFGKIKSCTLARDP
Splooce       KVRQGAKASIPQACRAAPPRKPFVAGPGLTGLSISPPLPQVGRPSNIGQAQPIIDQLAEEARAFNRIYVASVHQDLSDDDIKSVFEAFGKIKSCTLARDP

Uniprot       TTGKHKGYGFIEYEKAQSSQDAVSSMNLFDLGGQYLRVGKAVTPPMPLLTPATPGGLPPAAAVAAAAATAKITAQEAVAGAAVLGTLGTPGLVSPALTLA
Splooce       TTGKHKGYGFIEYEKAQSSQDAVSSMNLFDLGGQYLRVGKAVTPPMPLLTPATPGGLPPAAAVAAAAATAKITAQEAVAGAAVLGTLGTPGLVSPALTLA

Uniprot       QPLGTLPQAVMAAQAPGVITGVTPARPPIPVTIPSVGVVNPILASPPTLGLLEPKKEKEEEELFPESERPEMLSEQEHMSISGSSARHMVMQKLLRKQES
Splooce       QPLGTLPQAVMAAQAPGVITGVTPARPPIPVTIPSVGVVNPILASPPTLGLLEPKKEKEEEELFPESERPEMLSEQEHMSISGSSARHMVMQKLLRKQES

Uniprot       TVMVLRNMVDPKDIDDDLEGEVTEECGKFGAVNRVIIYQEKQGEEEDAEIIVKIFVEFSIASETHKAIQALNGRWFAGRKVVAEVYDQERFDNSDLSA
Splooce       TVMVLRNMVDPKDIDDDLEGEVTEECGKFGAVNRVIIYQEKQGEEEDAEIIVKIFVEFSIASETHKAIQALNGRWFAGRKVVAEVYDQERFDNSDLSA

----------------------------------------------------------------------------------------------------

P35749 (Uniprot)	versus
NM_001040114#(-s-s-s-s-s-s-s-s-:16_M3200964908840) (Splooce)

For more details about the Alternative Splicing Event -> Link to Splooce page

Peptides that support the ASE (Splooce-specific):
HISTLNIQR (MAXQUANT)

Alignment:
Uniprot       MAQKGQLSDDEKFLFVDKNFINSPVAQADWAAKRLVWVPSEKQGFEAASIKEEKGDEVVVELVENGKKVTVGKDDIQKMNPPKFSKVEDMAELTCLNEAS
Splooce       MAQKGQLSDDEKFLFVDKNFINSPVAQADWAAKRLVWVPSEKQGFEAASIKEEKGDEVVVELVENGKKVTVGKDDIQKMNPPKFSKVEDMAELTCLNEAS

Uniprot       VLHNLRERYFSGLIYTYSGLFCVVVNPYKHLPIYSEKIVDMYKGKKRHEMPPHIYAIADTAYRSMLQDREDQSILCTGESGAGKTENTKKVIQYLAVVAS
Splooce       VLHNLRERYFSGLIYTYSGLFCVVVNPYKHLPIYSEKIVDMYKGKKRHEMPPHIYAIADTAYRSMLQDREDQSILCTGESGAGKTENTKKVIQYLAVVAS

Uniprot       SHKGKKDTSITQGPSFAYGELEKQLLQANPILEAFGNAKTVKNDNSSRFGKFIRINFDVTGYIVGANIETYLLEKSRAIRQARDERTFHIFYYMIAGAKE
Splooce       SHKGKKDTSITQGPSFAYGELEKQLLQANPILEAFGNAKTVKNDNSSRFGKFIRINFDVTGYIVGANIETYLLEKSRAIRQARDERTFHIFYYMIAGAKE

Uniprot       KMRSDLLLEGFNNYTFLSNGFVPIPAAQDDEMFQETVEAMAIMGFSEEEQLSILKVVSSVLQLGNIVFKKERNTDQASMPDNTAAQKVCHLMGINVTDFT
Splooce       KMRSDLLLEGFNNYTFLSNGFVPIPAAQDDEMFQETVEAMAIMGFSEEEQLSILKVVSSVLQLGNIVFKKERNTDQASMPDNTAAQKVCHLMGINVTDFT

Uniprot       RSILTPRIKVGRDVVQKAQTKEQADFAVEALAKATYERLFRWILTRVNKALDKTHRQGASFLGILDIAGFEIFEVNSFEQLCINYTNEKLQQLFNHTMFI
Splooce       RSILTPRIKVGRDVVQKAQTKEQADFAVEALAKATYERLFRWILTRVNKALDKTHRQGASFLGILDIAGFEIFEVNSFEQLCINYTNEKLQQLFNHTMFI

Uniprot       LEQEEYQREGIEWNFIDFGLDLQPCIELIERPNNPPGVLALLDEECWFPKATDKSFVEKLCTEQGSHPKFQKPKQLKDKTEFSIIHYAGKVDYNASAWLT
Splooce       LEQEEYQREGIEWNFIDFGLDLQPCIELIERPNNPPGVLALLDEECWFPKATDKSFVEKLCTEQGSHPKFQKPKQLKDKTEFSIIHYAGKVDYNASAWLT

Uniprot       KNMDPLNDNVTSLLNASSDKFVADLWKDVDRIVGLDQMAKMTESSLPSASKTKKGMFRTVGQLYKEQLGKLMTTLRNTTPNFVRCIIPNHEKRSGKLDAF
Splooce       KNMDPLNDNVTSLLNASSDKFVADLWKDVDRIVGLDQMAKMTESSLPSASKTKKGMFRTVGQLYKEQLGKLMTTLRNTTPNFVRCIIPNHEKRSGKLDAF

Uniprot       LVLEQLRCNGVLEGIRICRQGFPNRIVFQEFRQRYEILAANAIPKGFMDGKQACILMIKALELDPNLYRIGQSKIFFRTGVLAHLEEERDLKITDVIMAF
Splooce       LVLEQLRCNGVLEGIRICRQGFPNRIVFQEFRQRYEILAANAIPKGFMDGKQACILMIKALELDPNLYRIGQSKIFFRTGVLAHLEEERDLKITDVIMAF

Uniprot       QAMCRGYLARKAFAKRQQQLTAMKVIQRNCAAYLKLRNWQWWRLFTKVKPLLQVTRQEEEMQAKEDELQKTKERQQKAENELKELEQKHSQLTEEKNLLQ
Splooce       QAMCRGYLARKAFAKRQQQLTAMKVIQRNCAAYLKLRNWQWWRLFTKVKPLLQVTRQEEEMQAKEDELQKTKERQQKAENELKELEQKHSQLTEEKNLLQ

Uniprot       EQLQAETELYAEAEEMRVRLAAKKQELEEILHEMEARLEEEEDRGQQLQAERKKMAQQMLDLEEQLEEEEAARQKLQLEKVTAEAKIKKLEDEILVMDDQ
Splooce       EQLQAETELYAEAEEMRVRLAAKKQELEEILHEMEARLEEEEDRGQQLQAERKKMAQQMLDLEEQLEEEEAARQKLQLEKVTAEAKIKKLEDEILVMDDQ

Uniprot       NNKLSKERKLLEERISDLTTNLAEEEEKAKNLTKLKNKHESMISELEVRLKKEEKSRQELEKLKRKLEGDASDFHEQIADLQAQIAELKMQLAKKEEELQ
Splooce       NNKLSKERKLLEERISDLTTNLAEEEEKAKNLTKLKNKHESMISELEVRLKKEEKSRQELEKLKRKLEGDASDFHEQIADLQAQIAELKMQLAKKEEELQ

Uniprot       AALARLDDEIAQKNNALKKIRELEGHISDLQEDLDSERAARNKAEKQKRDLGEELEALKTELEDTLDSTATQQELRAKREQEVTVLKKALDEETRSHEAQ
Splooce       AALARLDDEIAQKNNALKKIRELEGHISDLQEDLDSERAARNKAEKQKRDLGEELEALKTELEDTLDSTATQQELRAKREQEVTVLKKALDEETRSHEAQ

Uniprot       VQEMRQKHAQAVEELTEQLEQFKRAKANLDKNKQTLEKENADLAGELRVLGQAKQEVEHKKKKLEAQVQELQSKCSDGERARAELNDKVHKLQNEVESVT
Splooce       VQEMRQKHAQAVEELTEQLEQFKRAKANLDKNKQTLEKENADLAGELRVLGQAKQEVEHKKKKLEAQVQELQSKCSDGERARAELNDKVHKLQNEVESVT

Uniprot       GMLNEAEGKAIKLAKDVASLSSQLQDTQELLQEETRQKLNVSTKLRQLEEERNSLQDQLDEEMEAKQNLERHISTLNIQLSDSKKKLQDFASTVEALEEG
Splooce       GMLNEAEGKAIKLAKDVASLSSQLQDTQELLQEETRQKLNVSTKLRQLEEERNSLQDQLDEEMEAKQNLERHISTLNIQRETG-----------------

Uniprot       KKRFQKEIENLTQQYEEKAAAYDKLEKTKNRLQQELDDLVVDLDNQRQLVSNLEKKQRKFDQLLAEEKNISSKYADERDRAEAEAREKETKALSLARALE
Splooce       -----------------------------------------------------------------------GHQVAEAERQEAEG---------------

Uniprot       EALEAKEELERTNKMLKAEMEDLVSSKDDVGKNVHELEKSKRALETQMEEMKTQLEELEDELQATEDAKLRLEVNMQALKGQFERDLQARDEQNEEKRRQ
Splooce       --------------------------------------------------------------------------NLAAGGGRAQ---DGRAVQGAGRERQ

Uniprot       LQRQLHEYETELEDERKQRALAAAAKKKLEGDLKDLELQADSAIKGREEAIKQLRKLQAQMKDFQRELEDARASRDEIFATAKENEKKAKSLEADLMQLQ
Splooce       CQG-------------------------------------------------------------QAAQEAAGGGRGGVPAHQR--------------QPQ

Uniprot       EDLAAAERARKQADLEKEELAEELASSLSGRNALQDEKRRLEARIAQLEEELEEEQGNMEAMSDRVRKATQQAEQLSNELATERSTAQKNESARQQLERQ
Splooce       EAAAGAG---------------------------------------------------------------------------------------------

Uniprot       NKELRSKLHEMEGAVKSKFKSTIAALEAKIAQLEEQVEQEAREKQAATKSLKQKDKKLKEILLQVEDERKMAEQYKEQAEKGNARVKQLKRQLEEAEEES
Splooce       ----------------------------------------------------------------------------------------------------

Uniprot       QRINANRRKLQRELDEATESNEAMGREVNALKSKLRRGNETSFVPSRRSGGRRVIENADGSEEETDTRDADFNGTKASE
Splooce       -------------------------------------------------------------------------------

----------------------------------------------------------------------------------------------------

Q86VP1 (Uniprot)	versus
NM_006024#(f-T:7_T5645758201167) (Splooce)

For more details about the Alternative Splicing Event -> Link to Splooce page

Peptides that support the ASE (Splooce-specific):
LELAEVQDNFR (MAXQUANT)

Alignment:
Uniprot       MTSFQEVPLQTSNFAHVIFQNVAKSYLPNAHLECHYTLTPYIHPHPKDWVGIFKVGWSTARDYYTFLWSPMPEHYVEGSTVNCVLAFQGYYLPNDDGEFY
Splooce       MTSFQEVPLQTSNFAHVIFQNVAKSYLPNAHLECHYTLTPYIHPHPKDWVGIFKVGWSTARDYYTFLWSPMPEHYVEGSTVNCVLAFQGYYLPNDDGEFY

Uniprot       QFCYVTHKGEIRGASTPFQFRASSPVEELLTMEDEGNSDMLVVTTKAGLLELKIEKTMKEKEELLKLIAVLEKETAQLREQVGRMERELNHEKERCDQLQ
Splooce       QFCYVTHKGEIRGASTPFQFRASSPVEELLTMEDEGNSDMLVVTTKAGLLELKIEKTMKEKEELLKLIAVLEKETAQLREQVGRMERELNHEKERCDQLQ

Uniprot       AEQKGLTEVTQSLKMENEEFKKRFSDATSKAHQLEEDIVSVTHKAIEKETELDSLKDKLKKAQHEREQLECQLKTEKDEKELYKVHLKNTEIENTKLMSE
Splooce       AEQKGLTEVTQSLKMENEEFKKRFSDATSKAHQLEEDIVSVTHKAIEKETELDSLKDKLKKAQHEREQLECQLKTEKDEKELYKVHLKNTEIENTKLMSE

Uniprot       VQTLKNLDGNKESVITHFKEEIGRLQLCLAEKENLQRTFLLTTSSKEDTCFLKEQLRKAEEQVQATRQEVVFLAKELSDAVNVRDRTMADLHTARLENEK
Splooce       VQTLKNLDGNKESVITHFKEEIGRLQLCLAEKENLQRTFLLTTSSKEDTCFLKEQLRKAEEQVQATRQEVVFLAKELSDAVNVRDRTMADLHTARLENEK

Uniprot       VKKQLADAVAELKLNAMKKDQDKTDTLEHELRREVEDLKLRLQMAADHYKEKFKECQRLQKQINKLSDQSANNNNVFTKKTGNQQKVNDASVNTDPATSA
Splooce       VKKQLADAVAELKLNAMKKDQDKTDTLEHELRREVEDLKLRLQMAADHYKEKFKECQRLQKQINKLSDQSANNNNVFTKKTGNQQKVNDASVNTDPATSA

Uniprot       STVDVKPSPSAAEADFDIVTKGQVCEMTKEIADKTEKYNKCKQLLQDEKAKCNKYADELAKMELKWKEQVKIAENVKLELAEVQDNYKELKRSLENPAER
Splooce       STVDVKPSPSAAEADFDIVTKGQVCEMTKEIADKTEKYNKCKQLLQDEKAKCNKYADELAKMELKWKEQVKIAENVKLELAEVQDNFRNLKGV-------

Uniprot       KMEGQNSQSPQCFKTCSEQNGYVLTLSNAQPVLQYGNPYASQETRDGADGAFYPDEIQRPPVRVPSWGLEDNVVCSQPARNFSRPDGLEDSEDSKEDENV
Splooce       ----------------------------------------------------------------------------------------------------

Uniprot       PTAPDPPSQHLRGHGTGFCFDSSFDVHKKCPLCELMFPPNYDQSKFEEHVESHWKVCPMCSEQFPPDYDQQVFERHVQTHFDQNVLNFD
Splooce       -----------------------------------------------------------------------------------------

----------------------------------------------------------------------------------------------------

Q00341 (Uniprot)	versus
NM_203346#(f-:2_H9472380759862) (Splooce)

For more details about the Alternative Splicing Event -> Link to Splooce page

Peptides that support the ASE (Splooce-specific):
LADVVDSEALQVYMK (MAXQUANT)

Alignment:
Uniprot       MSSVAVLTQESFAEHRSGLVPQQIKVATLNSEEESDPPTYKDAFPPLPEKAACLESAQEPSGAWGNKIRPIKASVITQVFHVPLEERKYKDMNQFGEGEQ
Splooce       MSSVAVLTQESFAEHRSGLVPQQIKVATLNSEEESDPPTYKDAFPPLPEKAACLESAQEPSGAWGNKIRPIKASVITQVFHVPLEERKYKDMNQFGEGEQ

Uniprot       AKICLEIMQRTGAHLELSLAKDQGLSIMVSGKLDAVMKARKDIVARLQTQASATVAIPKEHHRFVIGKNGEKLQDLELKTATKIQIPRPDDPSNQIKITG
Splooce       AKICLEIMQRTGAHLELSLAKDQGLSIMVSGKLDAVMKARKDIVARLQTQASATVAIPKEHHRFVIGKNGEKLQDLELKTATKIQIPRPDDPSNQIKITG

Uniprot       TKEGIEKARHEVLLISAEQDKRAVERLEVEKAFHPFIAGPYNRLVGEIMQETGTRINIPPPSVNRTEIVFTGEKEQLAQAVARIKKIYEEKKKKTTTIAV
Splooce       TKEGIEKARHEVLLISAEQDKRAVERLEVEKAFHPFIAGPYNRLVGEIMQETGTRINIPPPSVNRTEIVFTGEKEQLAQAVARIKKIYEEKKKKTTTIAV

Uniprot       EVKKSQHKYVIGPKGNSLQEILERTGVSVEIPPSDSISETVILRGEPEKLGQALTEVYAKANSFTVSSVAAPSWLHRFIIGKKGQNLAKITQQMPKVHIE
Splooce       EVKKSQHKYVIGPKGNSLQEILERTGVSVEIPPSDSISETVILRGEPEKLGQALTEVYAKANSFTVSSVAAPSWLHRFIIGKKGQNLAKITQQMPKVHIE

Uniprot       FTEGEDKITLEGPTEDVNVAQEQIEGMVKDLINRMDYVEINIDHKFHRHLIGKSGANINRIKDQYKVSVRIPPDSEKSNLIRIEGDPQGVQQAKRELLEL
Splooce       FTEGEDKITLEGPTEDVNVAQEQIEGMVKDLINRMDYVEINIDHKFHRHLIGKSGANINRIKDQYKVSVRIPPDSEKSNLIRIEGDPQGVQQAKRELLEL

Uniprot       ASRMENERTKDLIIEQRFHRTIIGQKGERIREIRDKFPEVIINFPDPAQKSDIVQLRGPKNEVEKCTKYMQKMVADLVENSYSISVPIFKQFHKNIIGKG
Splooce       ASRMENERTKDLIIEQRFHRTIIGQKGERIREIRDKFPEVIINFPDPAQKSDIVQLRGPKNEVEKCTKYMQKMVADLVENSYSISVPIFKQFHKNIIGKG

Uniprot       GANIKKIREESNTKIDLPAENSNSETIIITGKRANCEAARSRILSIQKDLANIAEVEVSIPAKLHNSLIGTKGRLIRSIMEECGGVHIHFPVEGSGSDTV
Splooce       GANIKKIREESNTKIDLPAENSNSETIIITGKRANCEAARSRILSIQKDLANIAEVEVSIPAKLHNSLIGTKGRLIRSIMEECGGVHIHFPVEGSGSDTV

Uniprot       VIRGPSSDVEKAKKQLLHLAEEKQTKSFTVDIRAKPEYHKFLIGKGGGKIRKVRDSTGARVIFPAAEDKDQDLITIIGKEDAVREAQKELEALIQNLDNV
Splooce       VIRGPSSDVEKAKKQLLHLAEEKQTKSFTVDIRAKPEYHKFLIGKGGGKIRKVRDSTGARVIFPAAEDKDQDLITIIGKEDAVREAQKELEALIQNLDNV

Uniprot       VEDSMLVDPKHHRHFVIRRGQVLREIAEEYGGVMVSFPRSGTQSDKVTLKGAKDCVEAAKKRIQEIIEDLEAQVTLECAIPQKFHRSVMGPKGSRIQQIT
Splooce       VEDSMLVDPKHHRHFVIRRGQVLREIAEEYGGVMVSFPRSGTQSDKVTLKGAKDCVEAAKKRIQEIIEDLEAQVTLECAIPQKFHRSVMGPKGSRIQQIT

Uniprot       RDFSVQIKFPDREENAVHSTEPVVQENGDEAGEGREAKDCDPGSPRRCDIIIISGRKEKCEAAKEALEALVPVTIEVEVPFDLHRYVIGQKGSGIRKMMD
Splooce       RDFSVQIKFPDREENAVHSTEPVVQENGDEAGEGREAKDCDPGSPRRCDIIIISGRKEKCEAAKEALEALVPVTIEVEVPFDLHRYVIGQKGSGIRKMMD

Uniprot       EFEVNIHVPAPELQSDIIAITGLAANLDRAKAGLLERVKELQAEQEDRALRSFKLSVTVDPKYHPKIIGRKGAVITQIRLEHDVNIQFPDKDDGNQPQDQ
Splooce       EFEVNIHVPAPELQSDIIAITGLAANLDRAKAGLLERVKELQAEQEDRALRSFKLSVTVDPKYHPKIIGRKGAVITQIRLEHDVNIQFPDKDDGNQPQDQ

Uniprot       ITITGYEKNTEAARDAILRIVGELEQMVSEDVPLDHRVHARIIGARGKAIRKIMDEFKVDIRFPQSGAPDPNCVTVTGLPENVEEAIDHILNLEEEY---
Splooce       ITITGYEKNTEAARDAILRIVGELEQMVSEDVPLDHRVHARIIGARGKAIRKIMDEFKVDIRFPQSGAPDPNCVTVTGLPENVEEAIDHILNLEEEYVSL

Uniprot       -------------------------LADVVDSEALQVYMKPPAHEEAKAPSRGFVVRDAPWTASSSEKAPDMSSSEEFPSFGAQVAPKTLPWGPKR
Splooce       CGPWSPEAPWHVHRPEAQPGASGDKLADVVDSEALQVYMKPPAHEEAKAPSRGFVVRDAPWTASSSEKAPDMSSSEEFPSFGAQVAPKTLPWGPKR

----------------------------------------------------------------------------------------------------

Q14839 (Uniprot)	versus
NM_001273#(-s-s-s-:12_C3136388242159) (Splooce)

For more details about the Alternative Splicing Event -> Link to Splooce page

Peptides that support the ASE (Splooce-specific):
LELHCQVMFR (MAXQUANT)

Alignment:
Uniprot       MASGLGSPSPCSAGSEEEDMDALLNNSLPPPHPENEEDPEEDLSETETPKLKKKKKPKKPRDPKIPKSKRQKKERMLLCRQLGDSSGEGPEFVEEEEEVA
Splooce       ----------------------------------------------------------------------------------------------------

Uniprot       LRSDSEGSDYTPGKKKKKKLGPKKEKKSKSKRKEEEEEEDDDDDSKEPKSSAQLLEDWGMEDIDHVFSEEDYRTLTNYKAFSQFVRPLIAAKNPKIAVSK
Splooce       ----------------------------------------------------------------------------------------------------

Uniprot       MMMVLGAKWREFSTNNPFKGSSGASVAAAAAAAVAVVESMVTATEVAPPPPPVEVPIRKAKTKEGKGPNARRKPKGSPRVPDAKKPKPKKVAPLKIKLGG
Splooce       ----------------------------------------------------------------------------------------------------

Uniprot       FGSKRKRSSSEDDDLDVESDFDDASINSYSVSDGSTSRSSRSRKKLRTTKKKKKGEEEVTAVDGYETDHQDYCEVCQQGGEIILCDTCPRAYHMVCLDPD
Splooce       ----------------------------------------------------------------------------------------------------

Uniprot       MEKAPEGKWSCPHCEKEGIQWEAKEDNSEGEEILEEVGGDLEEEDDHHMEFCRVCKDGGELLCCDTCPSSYHIHCLNPPLPEIPNGEWLCPRCTCPALKG
Splooce       ---------------------------------------------------------------------MWNLTSMMP----------------------

Uniprot       KVQKILIWKWGQPPSPTPVPRPPDADPNTPSPKPLEGRPERQFFVKWQGMSYWHCSWVSELQLELHCQVMFRNYQRKNDMDEPPSGDFGGDEEKSRKRKN
Splooce       -VSIAILFLMVPPAVVAAAAR---------NSEPLKRKR----------------------KLELHCQVMFRNYQRKNDMDEPPSGDFGGDEEKSRKRKN

Uniprot       KDPKFAEMEERFYRYGIKPEWMMIHRILNHSVDKKGHVHYLIKWRDLPYDQASWESEDVEIQDYDLFKQSYWNHRELMRGEEGRPGKKLKKVKLRKLERP
Splooce       KDPKFAEMEERFYRYGIKPEWMMIHRILNHSVDKKGHVHYLIKWRDLPYDQASWESEDVEIQDYDLFKQSYWNHRELMRGEEGRPGKKLKKVKLRKLERP

Uniprot       PETPTVDPTVKYERQPEYLDATGGTLHPYQMEGLNWLRFSWAQGTDTILADEMGLGKTVQTAVFLYSLYKEGHSKGPFLVSAPLSTIINWEREFEMWAPD
Splooce       PETPTVDPTVKYERQPEYLDATGGTLHPYQMEGLNWLRFSWAQGTDTILADEMGLGKTVQTAVFLYSLYKEGHSKGPFLVSAPLSTIINWEREFEMWAPD

Uniprot       MYVVTYVGDKDSRAIIRENEFSFEDNAIRGGKKASRMKKEASVKFHVLLTSYELITIDMAILGSIDWACLIVDEAHRLKNNQSKFFRVLNGYSLQHKLLL
Splooce       MYVVTYVGDKDSRAIIRENEFSFEDNAIRGGKKASRMKKEASVKFHVLLTSYELITIDMAILGSIDWACLIVDEAHRLKNNQSKFFRVLNGYSLQHKLLL

Uniprot       TGTPLQNNLEELFHLLNFLTPERFHNLEGFLEEFADIAKEDQIKKLHDMLGPHMLRRLKADVFKNMPSKTELIVRVELSPMQKKYYKYILTRNFEALNAR
Splooce       TGTPLQNNLEELFHLLNFLTPERFHNLEGFLEEFADIAKEDQIKKLHDMLGPHMLRRLKADVFKNMPSKTELIVRVELSPMQKKYYKYILTRNFEALNAR

Uniprot       GGGNQVSLLNVVMDLKKCCNHPYLFPVAAMEAPKMPNGMYDGSALIRASGKLLLLQKMLKNLKEGGHRVLIFSQMTKMLDLLEDFLEHEGYKYERIDGGI
Splooce       GGGNQVSLLNVVMDLKKCCNHPYLFPVAAMEAPKMPNGMYDGSALIRASGKLLLLQKMLKNLKEGGHRVLIFSQMTKMLDLLEDFLEHEGYKYERIDGGI

Uniprot       TGNMRQEAIDRFNAPGAQQFCFLLSTRAGGLGINLATADTVIIYDSDWNPHNDIQAFSRAHRIGQNKKVMIYRFVTRASVEERITQVAKKKMMLTHLVVR
Splooce       TGNMRQEAIDRFNAPGAQQFCFLLSTRAGGLGINLATADTVIIYDSDWNPHNDIQAFSRAHRIGQNKKVMIYRFVTRASVEERITQVAKKKMMLTHLVVR

Uniprot       PGLGSKTGSMSKQELDDILKFGTEELFKDEATDGGGDNKEGEDSSVIHYDDKAIERLLDRNQDETEDTELQGMNEYLSSFKVAQYVVREEEMGEEEEVER
Splooce       PGLGSKTGSMSKQELDDILKFGTEELFKDEATDGGGDNKEGEDSSVIHYDDKAIERLLDRNQDETEDTELQGMNEYLSSFKVAQYVVREEEMGEEEEVER

Uniprot       EIIKQEESVDPDYWEKLLRHHYEQQQEDLARNLGKGKRIRKQVNYNDGSQEDRDWQDDQSDNQSDYSVASEEGDEDFDERSEAPRRPSRKGLRNDKDKPL
Splooce       EIIKQEESVDPDYWEKLLRHHYEQQQEDLARNLGKGKRIRKQVNYNDGSQEDRDWQDDQSDNQSDYSVASEEGDEDFDERSEAPRRPSRKGLRNDKDKPL

Uniprot       PPLLARVGGNIEVLGFNARQRKAFLNAIMRYGMPPQDAFTTQWLVRDLRGKSEKEFKAYVSLFMRHLCEPGADGAETFADGVPREGLSRQHVLTRIGVMS
Splooce       PPLLARVGGNIEVLGFNARQRKAFLNAIMRYGMPPQDAFTTQWLVRDLRGKSEKEFKAYVSLFMRHLCEPGADGAETFADGVPREGLSRQHVLTRIGVMS

Uniprot       LIRKKVQEFEHVNGRWSMPELAEVEENKKMSQPGSPSPKTPTPSTPGDTQPNTPAPVPPAEDGIKIEENSLKEEESIEGEKEVKSTAPETAIECTQAPAP
Splooce       LIRKKVQEFEHVNGRWSMPELAEVEENKKMSQPGSPSPKTPTPSTPGDTQPNTPAPVPPAEDGIKIEENSLKEEESIEGEKEVKSTAPETAIECTQAPAP

Uniprot       ASEDEKVVVEPPEGEEKVEKAEVKERTEEPMETEPKGAADVEKVEEKSAIDLTPIVVEDKEEKKEEEEKKEVMLQNGETPKDLNDEKQKKNIKQRFMFNI
Splooce       ASEDEKVVVEPPEGEEKVEKAEVKERTEEPMETEPKGAADVEKVEEKSAIDLTPIVVEDKEEKKEEEEKKEVMLQNGETPKDLNDEKQKKNIKQRFMFNI

Uniprot       ADGGFTELHSLWQNEERAATVTKKTYEIWHRRHDYWLLAGIINHGYARWQDIQNDPRYAILNEPFKGEMNRGNFLEIKNKFLARRFKLLEQALVIEEQLR
Splooce       ADGGFTELHSLWQNEERAATVTKKTYEIWHRRHDYWLLAGIINHGYARWQDIQNDPRYAILNEPFKGEMNRGNFLEIKNKFLARRFKLLEQALVIEEQLR

Uniprot       RAAYLNMSEDPSHPSMALNTRFAEVECLAESHQHLSKESMAGNKPANAVLHKVLKQLEELLSDMKADVTRLPATIARIPPVAVRLQMSERNILSRLANRA
Splooce       RAAYLNMSEDPSHPSMALNTRFAEVECLAESHQHLSKESMAGNKPANAVLHKVLKQLEELLSDMKADVTRLPATIARIPPVAVRLQMSERNILSRLANRA

Uniprot       PEPTPQQVAQQQ
Splooce       PEPTPQQVAQQQ

----------------------------------------------------------------------------------------------------

Q0ZGT2 (Uniprot)	versus
NM_144573#(-s-:1_N8490427836624) (Splooce)

For more details about the Alternative Splicing Event -> Link to Splooce page

Peptides that support the ASE (Splooce-specific):
QEMGEEDDVDVRPAR (MAXQUANT)

Alignment:
Uniprot       MNDISQKAEILLSSSKPVPKTYVPKLGKGDVKDKFEAMQRAREERNQRRSRDEKQRRKEQYIREREWNRRKQEIKEMLASDDEEDVSSKVEKAYVPKLTG
Splooce       MNDISQKAEILLSSSKPVPKTYVPKLGKGDVKDKFEAMQRAREERNQRRSRDEKQRRKEQYIREREWNRRKQEIKEMLASDDEEDVSSKVEKAYVPKLTG

Uniprot       TVKGRFAEMEKQRQEEQRKRTEEERKRRIEQDMLEKRKIQRELAKRAEQIEDINNTGTESASEEGDDSLLITVVPVKSYKTSGKMKKNFEDLEKEREEKE
Splooce       TVKGRFAEMEKQRQEEQRKRTEEERKRRIEQDMLEKRKIQRELAKRAEQIEDINNTGTESASEEGDDSLLITVVPVKSYKTSGKMKKNFEDLEKEREEKE

Uniprot       RIKYEEDKRIRYEEQRPSLKEAKCLSLVMDDEIESEAKKESLSPGKLKLTFEELERQRQENRKKQAEEEARKRLEEEKRAFEEARRQMVNEDEENQDTAK
Splooce       RIKYEEDKRIRYEEQRPSLKEAKCLSLVMDDEIESEAKKESLSPGKLKLTFEELERQRQENRKKQAEEEARKRLEEEKRAFEEARRQMVNEDEENQDTAK

Uniprot       IFKGYRPGKLKLSFEEMERQRREDEKRKAEEEARRRIEEEKKAFAEARRNMVVDDDSPEMYKTISQEFLTPGKLEINFEELLKQKMEEEKRRTEEERKHK
Splooce       IFKGYRPGKLKLSFEEMERQRREDEKRKAEEEARRRIEEEKKAFAEARRNMVVDDDSPEMYKTISQEFLTPGKLEINFEELLKQKMEEEKRRTEEERKHK

Uniprot       LEMEKQEFEQLRQEMGEEEEENETFGLSREYEELIKLKRSGSIQAKNLKSKFEKIGQLSEKEIQKKIEEERARRRAIDLEIKEREAENFHEEDDVDVRPA
Splooce       LEMEKQEFEQLRQEMGEE--------------------------------------------------------------------------DDVDVRPA

Uniprot       RKSEAPFTHKVNMKARFEQMAKAREEEEQRRIEEQKLLRMQFEQREIDAALQKKREEEEEEEGSIMNGSTAEDEEQTRSGAPWFKKPLKNTSVVDSEPVR
Splooce       RKSEAPFTHKVNMKARFEQMAKAREEEEQRRIEEQKLLRMQFEQREIDAALQKKREEEEEEEGSIMNGSTAEDEEQTRSGAPWFKKPLKNTSVVDSEPVR

Uniprot       FTVKVTGEPKPEITWWFEGEILQDGEDYQYIERGETYCLYLPETFPEDGGEYMCKAVNNKGSAASTCILTIESKN
Splooce       FTVKVTGEPKPEITWWFEGEILQDGEDYQYIERGETYCLYLPETFPEDGGEYMCKAVNNKGSAASTCILTIESKN

----------------------------------------------------------------------------------------------------

P13798 (Uniprot)	versus
NM_001640#(-s-s-s-s-:3_A2316569363743) (Splooce)

For more details about the Alternative Splicing Event -> Link to Splooce page

Peptides that support the ASE (Splooce-specific):
VLQPPPEQENVQYGAWWR (MAXQUANT)

Alignment:
Uniprot       MERQVLLSEPEEAAALYRGLSRQPALSAACLGPEVTTQYGGQYRTVHTEWTQRDLERMENIRFCRQYLVFHDGDSVVFAGPAGNSVETRGELLSRESPSG
Splooce       MERQVLLSEPEEAAALYRGLSRQPALSAACLGPEVTTQYGGQYRTVHTEWTQRDLERMENIRFCRQYLVFHDGDSVVFAGPAGNSVETRGELLSRESPSG

Uniprot       TMKAVLRKAGGTGPGEEKQFLEVWEKNRKLKSFNLSALEKHGPVYEDDCFGCLSWSHSETHLLYVAEKKRPKAESFFQTKALDVSASDDEIARLKKPDQA
Splooce       TMKAVLRKAGGTGPGEEKQFLEVWEKNRKLKSFNLSALEKHGPVYEDDCFGCLSWSHSETHLLYVAEKKRPKAESFFQTKALDVSASDDEIARLKKPDQA

Uniprot       IKGDQFVFYEDWGENMVSKSIPVLCVLDVESGNISVLEGVPENVSPGQAFWAPGDAGVVFVGWWHEPFRLGIRFCTNRRSALYYVDLIGGKCELLSDDSL
Splooce       IKGDQFVFYEDWGENMVSKSIPVLCVLDVESGNISVLEGVPENVSPGQAFWAPGDAGVVFVGWWHEPFRLGIRFCTNRRSALYYVDLIGGKCELLSDDSL

Uniprot       AVSSPRLSPDQCRIVYLQYPSLIPHHQCSQLCLYDWYTKVTSVVVDVVPRQLGENFSGIYCSLLPLGCWSADSQRVVFDSAQRSRQDLFAVDTQVGTVTS
Splooce       AVSSPRLSPDQCRIVYLQYPSLIPHHQCSQLCLYDWYTKVTSVVVDVVPRQLGENFSGIYCSLLPLGCWSADSQRVVFDSAQRSRQDLFAVDTQVGTVTS

Uniprot       LTAGGSGGSWKLLTIDQDLMVAQFSTPSLPPTLKVGFLPSAGKEQSVLWVSLEEAEPIPDIHWGIRVLQPPPEQENVQYAGLDFEAILLQPGSPPDKTQV
Splooce       LTAGGSGGSWKLLTIDQDLMVAQFSTPSLPPTLKVGFLPSAGKEQSVLWVSLEEAEPIPDIHWGIRVLQPPPEQENVQYG--------------------

Uniprot       PMVVMPHGGPHSSFVTAWMLFPAMLCKMGFAVLLVNYRGSTGFGQDSILSLPGNVGHQDVKDVQFAVEQVLQEEHFDASHVALMGGSHGGFISCHLIGQY
Splooce       ----------------AWWRLAFLSAVTACQTSACGLRCWTNRPSDTSLR--------------------------------------------------

Uniprot       PETYRACVARNPVINIASMLGSTDIPDWCVVEAGFPFSSDCLPDLSVWAEMLDKSPIRYIPQVKTPLLLMLGQEDRRVPFKQGMEYYRALKTRNVPVRLL
Splooce       ----------------------------------------------------------------------------------------------------

Uniprot       LYPKSTHALSEVEVESDSFMNAVLWLRTHLGS
Splooce       --------------------------------

----------------------------------------------------------------------------------------------------

Q86TI2 (Uniprot)	versus
NM_139159#(-s-s-s-s-s-s-s-s-s-:19_D7682186977503) (Splooce)

For more details about the Alternative Splicing Event -> Link to Splooce page

Peptides that support the ASE (Splooce-specific):
QMLDHFQIWVNEETK (MAXQUANT)

Alignment:
Uniprot       MRKVKKLRLDKENTGSWRSFSLNSEGAERMATTGTPTADRGDAAATDDPAARFQVQKHSWDGLRSIIHGSRKYSGLIVNKAPHDFQFVQKTDESGPHSHR
Splooce       MRKVKKLRLDKENTGSWRSFSLNSEGAERMATTGTPTADRGDAAATDDPAARFQVQKHSWDGLRSIIHGSRKYSGLIVNKAPHDFQFVQKTDESGPHSHR

Uniprot       LYYLGMPYGSRENSLLYSEIPKKVRKEALLLLSWKQMLDHFQATPHHGVYSREEELLRERKRLGVFGITSYDFHSESGLFLFQASNSLFHCRDGGKNGFM
Splooce       LYYLGMPYGSRENSLLYSEIPKKVRKEALLLLSWKQMLDHFQ----------------------------------------------------------

Uniprot       VSPMKPLEIKTQCSGPRMDPKICPADPAFFSFINNSDLWVANIETGEERRLTFCHQGLSNVLDDPKSAGVATFVIQEEFDRFTGYWWCPTASWEGSEGLK
Splooce       ----------------------------------------------------------------------------------------------------

Uniprot       TLRILYEEVDESEVEVIHVPSPALEERKTDSYRYPRTGSKNPKIALKLAEFQTDSQGKIVSTQEKELVQPFSSLFPKVEYIARAGWTRDGKYAWAMFLDR
Splooce       ----------------------------------------------------------------------------------------------------

Uniprot       PQQWLQLVLLPPALFIPSTENEEQRLASARAVPRNVQPYVVYEEVTNVWINVHDIFYPFPQSEGEDELCFLRANECKTGFCHLYKVTAVLKSQGYDWSEP
Splooce       ----------------------------------------------------------------------------------------------------

Uniprot       FSPGEDEFKCPIKEEIALTSGEWEVLARHGSKIWVNEETKLVYFQGTKDTPLEHHLYVVSYEAAGEIVRLTTPGFSHSCSMSQNFDMFVSHYSSVSTPPC
Splooce       --------------------------------IWVNEETKLVYFQGTKDTPLEHHLYVVSYEAAGEIVRLTTPGFSHSCSMSQNFDMFVSHYSSVSTPPC

Uniprot       VHVYKLSGPDDDPLHKQPRFWASMMEAASCPPDYVPPEIFHFHTRSDVRLYGMIYKPHALQPGKKHPTVLFVYGGPQVQLVNNSFKGIKYLRLNTLASLG
Splooce       VHVYKLSGPDDDPLHKQPRFWASMMEAASCPPDYVPPEIFHFHTRSDVRLYGMIYKPHALQPGKKHPTVLFVYGGPQVQLVNNSFKGIKYLRLNTLASLG

Uniprot       YAVVVIDGRGSCQRGLRFEGALKNQMGQVEIEDQVEGLQFVAEKYGFIDLSRVAIHGWSYGGFLSLMGLIHKPQVFKVAIAGAPVTVWMAYDTGYTERYM
Splooce       YAVVVIDGRGSCQRGLRFEGALKNQMGQVEIEDQVEGLQFVAEKYGFIDLSRVAIHGWSYGGFLSLMGLIHKPQVFKVAIAGAPVTVWMAYDTGYTERYM

Uniprot       DVPENNQHGYEAGSVALHVEKLPNEPNRLLILHGFLDENVHFFHTNFLVSQLIRAGKPYQLQIYPNERHSIRCPESGEHYEVTLLHFLQEYL
Splooce       DVPENNQHGYEAGSVALHVEKLPNEPNRLLILHGFLDENVHFFHTNFLVSQLIRAGKPYQLQIYPNERHSIRCPESGEHYEVTLLHFLQEYL

----------------------------------------------------------------------------------------------------

P08240 (Uniprot)	versus
NM_003139#(-s-s-s-:11_S2407583710265) (Splooce)

For more details about the Alternative Splicing Event -> Link to Splooce page

Peptides that support the ASE (Splooce-specific):
HSHSSINWTTSLSWCLCK (MAXQUANT)

Alignment:
Uniprot       MLDFFTIFSKGGLVLWCFQGVSDSCTGPVNALIRSVLLQERGGNNSFTHEALTLKYKLDNQFELVFVVGFQKILTLTYVDKLIDDVHRLFRDKYRTEIQQ
Splooce       ----------------------------------------------------------------------------------------------------

Uniprot       QSALSLLNGTFDFQNDFLRLLREAEESSKIRAPTTMKKFEDSEKAKKPVRSMIETRGEKPKEKAKNSKKKGAKKEGSDGPLATSKPVPAEKSGLPVGPEN
Splooce       --------------------MRHSHSS--INWTTSLSWC-------------------------------------------------------------

Uniprot       GVELSKEELIRRKREEFIQKHGRGMEKSNKSTKSDAPKEKGKKAPRVWELGGCANKEVLDYSTPTTNGTPEAALSEDINLIRGTGSGGQLQDLDCSSSDD
Splooce       ---------------------------LCKSTKSDAPKEKGKKAPRVWELGGCANKEVLDYSTPTTNGTPEAALSEDINLIRGTGSGGQLQDLDCSSSDD

Uniprot       EGAAQNSTKPSATKGTLGGMFGMLKGLVGSKSLSREDMESVLDKMRDHLIAKNVAADIAVQLCESVANKLEGKVMGTFSTVTSTVKQALQESLVQILQPQ
Splooce       EGAAQNSTKPSATKGTLGGMFGMLKGLVGSKSLSREDMESVLDKMRDHLIAKNVAADIAVQLCESVANKLEGKVMGTFSTVTSTVKQALQESLVQILQPQ

Uniprot       RRVDMLRDIMDAQRRQRPYVVTFCGVNGVGKSTNLAKISFWLLENGFSVLIAACDTFRAGAVEQLRTHTRRLSALHPPEKHGGRTMVQLFEKGYGKDAAG
Splooce       RRVDMLRDIMDAQRRQRPYVVTFCGVNGVGKSTNLAKISFWLLENGFSVLIAACDTFRAGAVEQLRTHTRRLSALHPPEKHGGRTMVQLFEKGYGKDAAG

Uniprot       IAMEAIAFARNQGFDVVLVDTAGRMQDNAPLMTALAKLITVNTPDLVLFVGEALVGNEAVDQLVKFNRALADHSMAQTPRLIDGIVLTKFDTIDDKVGAA
Splooce       IAMEAIAFARNQGFDVVLVDTAGRMQDNAPLMTALAKLITVNTPDLVLFVGEALVGNEAVDQLVKFNRALADHSMAQTPRLIDGIVLTKFDTIDDKVGAA

Uniprot       ISMTYITSKPIVFVGTGQTYCDLRSLNAKAVVAALMKA
Splooce       ISMTYITSKPIVFVGTGQTYCDLRSLNAKAVVAALMKA

----------------------------------------------------------------------------------------------------

Q10471 (Uniprot)	versus
NM_004481#(-s-s-s-:1_G6568049815005) (Splooce)

For more details about the Alternative Splicing Event -> Link to Splooce page

Peptides that support the ASE (Splooce-specific):
AAVWDTCSGSSTPTR (MAXQUANT)

Alignment:
Uniprot       MRRRSRMLLCFAFLWVLGIAYYMYSGGGSALAGGAGGGAGRKEDWNEIDPIKKKDLHHSNGEEKAQSMETLPPGKVRWPDFNQEAYVGGTMVRSGQDPYA
Splooce       MRRRSRMLLCFAFLWVLGIAYYMYSGGGSALAGGAGGGAGRKEDWNEIDPIKKKDLHHSNGEEKAQSMETLPPGKVRWPDFNQEAYVGGTMVRSGQDPYA

Uniprot       RNKFNQVESDKLRMDRAIPDTRHDQCQRKQWRVDLPATSVVITFHNEARSALLRTVVSVLKKSPPHLIKEIILVDDYSNDPEDGALLGKIEKVRVLRNDR
Splooce       RNKFNQVESDKLRMDRAIPDTRHDQCQRKQWRVDLPATSVVITFHNEARSALLRTVVSVLKKSPPHLIKEIILVDDYSNDPEDGALLGKIEKVRVLRNDR

Uniprot       REGLMRSRVRGADAAQAKVLTFLDSHCECNEHWLEPLLERVAEDRTRVVSPIIDVINMDNFQYVGASADLKGGFDWNLVFKWDYMTPEQRRSRQGNPVAP
Splooce       REGLMRSRVRGADAAQAKVLTFLDSHCECNEHWLEPLLERVAE-----------------------------------------------RSRS------

Uniprot       IKTPMIAGGLFVMDKFYFEELGKYDMMMDVWGGENLEISFRVWQCGGSLEIIPCSRVGHVFRKQHPYTFPGGSGTVFARNTRRAAEVWMDEYKNFYYAAV
Splooce       ----------------------------------------------------ACGSVVAAWRSSRAAVWDTCSGSSTPTRSRVAVALSLPETP-------

Uniprot       PSARNVPYGNIQSRLELRKKLSCKPFKWYLENVYPELRVPDHQDIAFGALQQGTNCLDTLGHFADGVVGVYECHNAGGNQEWALTKEKSVKHMDLCLTVV
Splooce       --AGQQRSGWMNTKISIMQ--QCLLLETFLMEIF--------------------------------------------RADWSLGRN-------------

Uniprot       DRAPGSLIKLQGCRENDSRQKWEQIEGNSKLRHVGSNLCLDSRTAKSGGLSVEVCGPALSQQWKFTLNLQQ
Splooce       -----------------------------------SAASLSNGTLK---MSIQS-----------------

----------------------------------------------------------------------------------------------------

P55039 (Uniprot)	versus
NM_001388#(-s-s-s-:17_D3944457655856) (Splooce)

For more details about the Alternative Splicing Event -> Link to Splooce page

Peptides that support the ASE (Splooce-specific):
STFLSLMTSTASEAASYEFTTLTCIPGVIEPKK (MAXQUANT)

Alignment:
Uniprot       MGILEKISEIEKEIARTQKNKATEYHLGLLKAKLAKYRAQLLEPSKSASSKGEGFDVMKSGDARVALIGFPSVGKSTFLSLMTSTASEAASYEFTTLTCI
Splooce       MGILEKISEIEKEIARTQKNKATEYHLGLLKAKLAKYRAQLLEPSKSASSKGEGFDVMKSGDARVALIGFPSVGKSTFLSLMTSTASEAASYEFTTLTCI

Uniprot       PGVIEYKGANIQLLDLPGIIEGAAQGKGRGRQVIAVARTADVIIMMLDATKGEVQRSLLEKELESVGIRLNKHKPNIYFKPKKGGGISFNSTVTLTQCSE
Splooce       PGVIE---------------------------------------------------------------------------PKKGGGISFNSTVTLTQCSE

Uniprot       KLVQLILHEYKIFNAEVLFREDCSPDEFIDVIVGNRVYMPCLYVYNKIDQISMEEVDRLARKPNSVVISCGMKLNLDYLLEMLWEYLALTCIYTKKRGQR
Splooce       KLVQLILHEYKIFNAEVLFREDCSPDEFIDVIVGNRVYMPCLYVYNKIDQISMEEVDRLARKPNSVVISCGMKLNLDYLLEMLWEYLALTCIYTKKRGQR

Uniprot       PDFTDAIILRKGASVEHVCHRIHRSLASQFKYALVWGTSTKYSPQRVGLTHTMEHEDVIQIVKK
Splooce       PDFTDAIILRKGASVEHVCHRIHRSLASQFKYALVWGTSTKYSPQRVGLTHTMEHEDVIQIVKK

----------------------------------------------------------------------------------------------------

O14579 (Uniprot)	versus
NM_007263#(-t:19_C2898000443269) (Splooce)

For more details about the Alternative Splicing Event -> Link to Splooce page

Peptides that support the ASE (Splooce-specific):
FADYLAHESR (MAXQUANT)

Alignment:
Uniprot       MAPPAPGPASGGSGEVDELFDVKNAFYIGSYQQCINEAQRVKLSSPERDVERDVFLYRAYLAQRKFGVVLDEIKPSSAPELQAVRMFADYLAHESRRDSI
Splooce       -------------------------------------------------------------------------------------MFADYLAHESRRDSI

Uniprot       VAELDREMSRSVDVTNTTFLLMAASIYLHDQNPDAALRALHQGDSLECTAMTVQILLKLDRLDLARKELKRMQDLDEDATLTQLATAWVSLATGGEKLQD
Splooce       VAELDREMSRSVDVTNTTFLLMAASIYLHDQNPDAALRALHQGDSLECTAMTVQILLKLDRLDLARKELKRMQDLDEDATLTQLATAWVSLATGGEKLQD

Uniprot       AYYIFQEMADKCSPTLLLLNGQAACHMAQGRWEAAEGLLQEALDKDSGYPETLVNLIVLSQHLGKPPEVTNRYLSQLKDAHRSHPFIKEYQAKENDFDRL
Splooce       AYYIFQEMADKCSPTLLLLNGQAACHMAQGRWEAAEGLLQEALDKDSGYPETLVNLIVLSQHLGKPPEVTNRYLSQLKDAHRSHPFIKEYQAKENDFDRL

Uniprot       VLQYAPSA
Splooce       VLQYAPSA

----------------------------------------------------------------------------------------------------

Q9UKY7 (Uniprot)	versus
NM_001134422#(-t:3_C624029773546) (Splooce)

For more details about the Alternative Splicing Event -> Link to Splooce page

Peptides that support the ASE (Splooce-specific):
VQAMQISEKEEDDNEKR (MAXQUANT)
VQAMQISEKEEDDNEK (MAXQUANT)

Alignment:
Uniprot       MAETEERSLDNFFAKRDKKKKKERSNRAASAAGAAGSAGGSSGAAGAAGGGAGAGTRPGDGGTASAGAAGPGAATKAVTKDEDEWKELEQKEVDYSGLRV
Splooce       MAETEERSLDNFFAKRDKKKKKERSNRAASAAGAAGSAGGSSGAAGAAGGGAGAGTRPGDGGTASAGAAGPGAATKAVTKDEDEWKELEQKEVDYSGLRV

Uniprot       QAMQISSEKEEDDNEKRQDPGDNWEEGGGGGGGMEKSSGPWNKTAPVQAPPAPVIVTETPEPAMTSGVYRPPGARLTTTRKTPQGPPEIYSDTQFPSLQS
Splooce       QAMQIS-EKEEDDNEKRQDPGDNWEEGGGGGGGMEKSSGPWNKTAPVQAPPAPVIVTETPEPAMTSGVYRPPGARLTTTRKTPQGPPEIYSDTQFPSLQS

Uniprot       TAKHVESRNRYLK
Splooce       TAKHVESRNRYLK

----------------------------------------------------------------------------------------------------

Q9H4K7 (Uniprot)	versus
NM_015666#(f-:20_G6426010142294) (Splooce)

For more details about the Alternative Splicing Event -> Link to Splooce page

Peptides that support the ASE (Splooce-specific):
LLELEQLACLLLR (MAXQUANT)

Alignment:
Uniprot       MAPARCFSARLRTVFQGVGHWALSTWAGLKPSRLLPQRASPRLLSVGRADLAKHQELPGKKLLSEKKL------------------KRYFVDYRRVLVCG
Splooce       MAPARCFSARLRTVFQGVGHWALSTWAGLKPSRLLPQRASPRLLSVGRADLAKHQELPGKKLLSEKKLVRLLELEQLACLLLRRHRKRYFVDYRRVLVCG

Uniprot       GNGGAGASCFHSEPRKEFGGPDGGDGGNGGHVILRVDQQVKSLSSVLSRYQGFSGEDGGSKNCFGRSGAVLYIRVPVGTLVKEGGRVVADLSCVGDEYIA
Splooce       GNGGAGASCFHSEPRKEFGGPDGGDGGNGGHVILRVDQQVKSLSSVLSRYQGFSGEDGGSKNCFGRSGAVLYIRVPVGTLVKEGGRVVADLSCVGDEYIA

Uniprot       ALGGAGGKGNRFFLANNNRAPVTCTPGQPGQQRVLHLELKTVAHAGMVGFPNAGKSSLLRAISNARPAVASYPFTTLKPHVGIVHYEGHLQIAVADIPGI
Splooce       ALGGAGGKGNRFFLANNNRAPVTCTPGQPGQQRVLHLELKTVAHAGMVGFPNAGKSSLLRAISNARPAVASYPFTTLKPHVGIVHYEGHLQIAVADIPGI

Uniprot       IRGAHQNRGLGSAFLRHIERCRFLLFVVDLSQPEPWTQVDDLKYELEMYEKGLSARPHAIVANKIDLPEAQANLSQLRDHLGQEVIVLSALTGENLEQLL
Splooce       IRGAHQNRGLGSAFLRHIERCRFLLFVVDLSQPEPWTQVDDLKYELEMYEKGLSARPHAIVANKIDLPEAQANLSQLRDHLGQEVIVLSALTGENLEQLL

Uniprot       LHLKVLYDAYAEAELGQGRQPLRW
Splooce       LHLKVLYDAYAEAELGQGRQPLRW

----------------------------------------------------------------------------------------------------

P17096 (Uniprot)	versus
NM_002131#(f-T:6_H1312819409117) (Splooce)

For more details about the Alternative Splicing Event -> Link to Splooce page

Peptides that support the ASE (Splooce-specific):
KQPPVSPGTALVGSQEPSEVPTPK (MAXQUANT)

Alignment:
Uniprot       MSESSSKSSQPLASKQEKDGTEKRGRGRPRKQPP----------KEPSEVPTPKRPRGRPKGSKNKGAAKTRKTTTTPGRKPRGRPKKLEKEEEEGISQE
Splooce       MSESSSKSSQPLASKQEKDGTEKRGRGRPRKQPPVSPGTALVGSQEPSEVPTPKRPRGRPKGSKNKGAAKTRKTTTTPGRKPRGRPKKLEKEEEEGISQE

Uniprot       SSEEEQ
Splooce       SSEEEQ

----------------------------------------------------------------------------------------------------

Q7L576 (Uniprot)	versus
NM_014608#(-s-s-s-s-s-s-s-s-s-s-:15_C7996364890293) (Splooce)

For more details about the Alternative Splicing Event -> Link to Splooce page

Peptides that support the ASE (Splooce-specific):
LMNFMYFQLYMVR (MAXQUANT)

Alignment:
Uniprot       MAAQVTLEDALSNVDLLEELPLPDQQPCIEPPPSSLLYQPNFNTNFEDRNAFVTGIARYIEQATVHSSMNEMLEEGQEYAVMLYTWRSCSRAIPQVKCNE
Splooce       MAAQVTLEDALSNVDLLEELPLPDQQPCIEPPPSSLLYQPNFNTNFEDRNAFVTGIARYIEQATVHSSMNEMLEEGQEYAVMLYTWRSCSRAIPQVKCNE

Uniprot       QPNRVEIYEKTVEVLEPEVTKLMNFMYFQRNAIERFCGEVRRLCHAERRKDFVSEAYLITLGKFINMFAVLDELKNMKCSVKNDHSAYKRAAQFLRKMAD
Splooce       QPNRVEIYEKTVEVLEPEVTKLMNFMYFQ-----------------------------------------------------------------------

Uniprot       PQSIQESQNLSMFLANHNKITQSLQQQLEVISGYEELLADIVNLCVDYYENRMYLTPSEKHMLLKVMGFGLYLMDGSVSNIYKLDAKKRINLSKIDKYFK
Splooce       ----------------------------------------------------------------------------------------------------

Uniprot       QLQVVPLFGDMQIELARYIKTSAHYEENKSRWTCTSSGSSPQYNICEQMIQIREDHMRFISELARYSNSEVVTGSGRQEAQKTDAEYRKLFDLALQGLQL
Splooce       ----------------------------------------------------------------------------------------------------

Uniprot       LSQWSAHVMEVYSWKLVHPTDKYSNKDCPDSAEEYERATRYNYTSEEKFALVEVIAMIKGLQVLMGRMESVFNHAIRHTVYAALQDFSQVTLREPLRQAI
Splooce       ----------------------------------------------------------------------------------------------------

Uniprot       KKKKNVIQSVLQAIRKTVCDWETGHEPFNDPALRGEKDPKSGFDIKVPRRAVGPSSTQLYMVRTMLESLIADKSGSKKTLRSSLEGPTILDIEKFHRESF
Splooce       ----------------------------------------------------------LYMVRTMLESLIADKSGSKKTLRSSLEGPTILDIEKFHRESF

Uniprot       FYTHLINFSETLQQCCDLSQLWFREFFLELTMGRRIQFPIEMSMPWILTDHILETKEASMMEYVLYSLDLYNDSAHYALTRFNKQFLYDEIEAEVNLCFD
Splooce       FYTHLINFSETLQQCCDLSQLWFREFFLELTMGRRIQFPIEMSMPWILTDHILETKEASMMEYVLYSLDLYNDSAHYALTRFNKQFLYDEIEAEVNLCFD

Uniprot       QFVYKLADQIFAYYKVMAGSLLLDKRLRSECKNQGATIHLPPSNRYETLLKQRHVQLLGRSIDLNRLITQRVSAAMYKSLELAIGRFESEDLTSIVELDG
Splooce       QFVYKLADQIFAYYKVMAGSLLLDKRLRSECKNQGATIHLPPSNRYETLLKQRHVQLLGRSIDLNRLITQRVSAAMYKSLELAIGRFESEDLTSIVELDG

Uniprot       LLEINRMTHKLLSRYLTLDGFDAMFREANHNVSAPYGRITLHVFWELNYDFLPNYCYNGSTNRFVRTVLPFSQEFQRDKQPNAQPQYLHGSKALNLAYSS
Splooce       LLEINRMTHKLLSRYLTLDGFDAMFREANHNVSAPYGRITLHVFWELNYDFLPNYCYNGSTNRFVRTVLPFSQEFQRDKQPNAQPQYLHGSKALNLAYSS

Uniprot       IYGSYRNFVGPPHFQVICRLLGYQGIAVVMEELLKVVKSLLQGTILQYVKTLMEVMPKICRLPRHEYGSPGILEFFHHQLKDIVEYAELKTVCFQNLREV
Splooce       IYGSYRNFVGPPHFQVICRLLGYQGIAVVMEELLKVVKSLLQGTILQYVKTLMEVMPKICRLPRHEYGSPGILEFFHHQLKDIVEYAELKTVCFQNLREV

Uniprot       GNAILFCLLIEQSLSLEEVCDLLHAAPFQNILPRVHVKEGERLDAKMKRLESKYAPLHLVPLIERLGTPQQIAIAREGDLLTKERLCCGLSMFEVILTRI
Splooce       GNAILFCLLIEQSLSLEEVCDLLHAAPFQNILPRVHVKEGERLDAKMKRLESKYAPLHLVPLIERLGTPQQIAIAREGDLLTKERLCCGLSMFEVILTRI

Uniprot       RSFLDDPIWRGPLPSNGVMHVDECVEFHRLWSAMQFVYCIPVGTHEFTVEQCFGDGLHWAGCMIIVLLGQQRRFAVLDFCYHLLKVQKHDGKDEIIKNVP
Splooce       RSFLDDPIWRGPLPSNGVMHVDECVEFHRLWSAMQFVYCIPVGTHEFTVEQCFGDGLHWAGCMIIVLLGQQRRFAVLDFCYHLLKVQKHDGKDEIIKNVP

Uniprot       LKKMVERIRKFQILNDEIITILDKYLKSGDGEGTPVEHVRCFQPPIHQSLASS
Splooce       LKKMVERIRKFQILNDEIITILDKYLKSGDGEGTPVEHVRCFQPPIHQSLASS

----------------------------------------------------------------------------------------------------

P19021 (Uniprot)	versus
NM_138821#(-s-:5_P5583641780042) (Splooce)

For more details about the Alternative Splicing Event -> Link to Splooce page

Peptides that support the ASE (Splooce-specific):
NWNIDQLK (MAXQUANT)

Alignment:
Uniprot       MAGRVPSLLVLLVFPSSCLAFRSPLSVFKRFKETTRPFSNECLGTTRPVVPIDSSDFALDIRMPGVTPKQSDTYFCMSMRIPVDEEAFVIDFKPRASMDT
Splooce       MAGRVPSLLVLLVFPSSCLAFRSPLSVFKRFKETTRPFSNECLGTTRPVVPIDSSDFALDIRMPGVTPKQSDTYFCMSMRIPVDEEAFVIDFKPRASMDT

Uniprot       VHHMLLFGCNMPSSTGSYWFCDEGTCTDKANILYAWARNAPPTRLPKGVGFRVGGETGSKYFVLQVHYGDISAFRDNNKDCSGVSLHLTRLPQPLIAGMY
Splooce       VHHMLLFGCNMPSSTGSYWFCDEGTCTDKANILYAWARNAPPTRLPKGVGFRVGGETGSKYFVLQVHYGDISAFRDNNKDCSGVSLHLTRLPQPLIAGMY

Uniprot       LMMSVDTVIPAGEKVVNSDISCHYKNYPMHVFAYRVHTHHLGKVVSGYRVRNGQWTLIGRQSPQLPQAFYPVGHPVDVSFGDLLAARCVFTGEGRTEATH
Splooce       LMMSVDTVIPAGEKVVNSDISCHYKNYPMHVFAYRVHTHHLGKVVSGYRVRNGQWTLIGRQSPQLPQAFYPVGHPVDVSFGDLLAARCVFTGEGRTEATH

Uniprot       IGGTSSDEMCNLYIMYYMEAKHAVSFMTCTQNVAPDMFRTIPPEANIPIPVKSDMVMMHEHHKETEYKDKIPLLQQPKREEEEVLDQDFHMEEALDWPGV
Splooce       IGGTSSDEMCNLYIMYYMEAKHAVSFMTCTQNVAPDMFRTIPPEANIPIPVKSDMVMMHEHHKETEYKDKIPLLQQPKREEEEVLDQDFHMEEALDWPGV

Uniprot       YLLPGQVSGVALDPKNNLVIFHRGDHVWDGNSFDSKFVYQQIGLGPIEEDTILVIDPNNAAVLQSSGKNLFYLPHGLSIDKDGNYWVTDVALHQVFKLDP
Splooce       YLLPGQVSGVALDPKNNLVIFHRGDHVWDGNSFDSKFVYQQIGLGPIEEDTILVIDPNNAAVLQSSGKNLFYLPHGLSIDKDGNYWVTDVALHQVFKLDP

Uniprot       NNKEGPVLILGRSMQPGSDQNHFCQPTDVAVDPGTGAIYVSDGYCNSRIVQFSPSGKFITQWGEESSGSSPLPGQFTVPHSLALVPLLGQLCVADRENGR
Splooce       NNKEGPVLILGRSMQPGSDQNHFCQPTDVAVDPGTGAIYVSDGYCNSRIVQFSPSGKFITQWGEESSGSSPLPGQFTVPHSLALVPLLGQLCVADRENGR

Uniprot       IQCFKTDTKEFVREIKHSSFGRNVFAISYIPGLLFAVNGKPHFGDQEPVQGFVMNFSNGEIIDIFKPVRKHFDMPHDIVASEDGTVYIGDAHTNTVWKFT
Splooce       IQCFKTDTKEFVREIKHSSFGRNVFAISYIPGLLFAVNGKPHFGDQEPVQGFVMNFSNGEIIDIFKPVRKN-------------------------WNID

Uniprot       LTEKLEHRSVKKAGIEVQEIKEAEAVVETKMENKPTSSELQKMQEKQKLIKEPGSGVPVVLITTLLVIPVVVLLAIAIFIRWKKSRAFGDSEHKLETSSG
Splooce       QLKRLALRSRKS--------KKPRQLLKPKWRTNPPPQNCRRCKRNRN----------------------------------------------------

Uniprot       RVLGRFRGKGSGGLNLGNFFASRKGYSRKGFDRLSTEGSDQEKEDDGSESEEEYSAPLPALAPSSS
Splooce       ------------------------------------------------------------------

----------------------------------------------------------------------------------------------------

Q6Q0C0 (Uniprot)	versus
NM_032271#(-s-s-s-:16_T3824896076798) (Splooce)

For more details about the Alternative Splicing Event -> Link to Splooce page

Peptides that support the ASE (Splooce-specific):
LYSGSADCTIIVWDIESK (MAXQUANT)

Alignment:
Uniprot       MSSGKSARYNRFSGGPSNLPTPDVTTGTRMETTFGPAFSAVTTITKADGTSTYKQHCRTPSSSSTLAYSPRDEEDSMPPISTPRRSDSAISVRSLHSESS
Splooce       MSSGKSARYNRFSGGPSNLPTPDVTTGTRMETTFGPAFSAVTTITKADGTSTYKQHCRTPSSSSTLAYSPRDEEDSMPPISTPRRSDSAISVRSLHSESS

Uniprot       MSLRSTFSLPEEEEEPEPLVFAEQPSVKLCCQLCCSVFKDPVITTCGHTFCRRCALKSEKCPVDNVKLTVVVNNIAVAEQIGELFIHCRHGCRVAGSGKP
Splooce       MSLRSTFSLPEEEEEPEPLVFAEQPSVKLCCQLCCSVFKDPVITTCGHTFCRRCALKSEKCPVDNVKLTVVVNNIAVAEQIGELFIHCRHGCRVAGSGKP

Uniprot       PIFEVDPRGCPFTIKLSARKDHEGSCDYRPVRCPNNPSCPPLLRMNLEAHLKECEHIKCPHSKYGCTFIGNQDTYETHLETCRFEGLKEFLQQTDDRFHE
Splooce       PIFEVDPRGCPFTIKLSARKDHEGSCDYRPVRCPNNPSCPPLLRMNLEAHLKECEHIKCPHSKYGCTFIGNQDTYETHLETCRFEGLKEFLQQTDDRFHE

Uniprot       MHVALAQKDQEIAFLRSMLGKLSEKIDQLEKSLELKFDVLDENQSKLSEDLMEFRRDASMLNDELSHINARLNMGILGSYDPQQIFKCKGTFVGHQGPVW
Splooce       MHVALAQKDQEIAFLRSMLGKLSEKIDQLEKSLELKFDVLDENQSKLSEDLMEFRRDASMLNDELSHINARLNMGILGSYDPQQIFKCKGTFVGHQGPVW

Uniprot       CLCVYSMGDLLFSGSSDKTIKVWDTCTTYKCQKTLEGHDGIVLALCIQGCKLYSGSADCTIIVWDIQNLQKVNTIRAHDNPVCTLVSSHNVLFSGSLKAI
Splooce       CLCVYSMGDLLFSGSSDKTIKVWDTCTTYKCQKTLEGHDGIVLALCIQGCKLYSGSADCTIIVWDIE---------------------------------

Uniprot       KVWDIVGTELKLKKELTGLNHWVRALVAAQSYLYSGSYQTIKIWDIRTLDCIHVLQTSGGSVYSIAVTNHHIVCGTYENLIHVWDIESKEQVRTLTGHVG
Splooce       ---------------------------------------------------------------------------------------SKEQVRTLTGHVG

Uniprot       TVYALAVISTPDQTKVFSASYDRSLRVWSMDNMICTQTLLRHQGSVTALAVSRGRLFSGAVDSTVKVWTC
Splooce       TVYALAVISTPDQTKVFSASYDRSLRVWSMDNMICTQTLLRHQGSVTALAVSRGRLFSGAVDSTVKVWTC

----------------------------------------------------------------------------------------------------

Q9UM47 (Uniprot)	versus
NM_000435#(-s-s-s-s-s-:19_N8911172752754) (Splooce)

For more details about the Alternative Splicing Event -> Link to Splooce page

Peptides that support the ASE (Splooce-specific):
VNGFSCTCPSGR (MAXQUANT)

Alignment:
Uniprot       MGPGARGRRRRRRPMSPPPPPPPVRALPLLLLLAGPGAAAPPCLDGSPCANGGRCTQLPSREAACLCPPGWVGERCQLEDPCHSGPCAGRGVCQSSVVAG
Splooce       MGPGARGRRRRRRPMSPPPPPPPVRALPLLLLLAGPGAAAPPCLDGSPCANGGRCTQLPSREAACLCPPGWVGERCQLEDPCHSGPCAGRGVCQSSVVAG

Uniprot       TARFSCRCPRGFRGPDCSLPDPCLSSPCAHGARCSVGPDGRFLCSCPPGYQGRSCRSDVDECRVGEPCRHGGTCLNTPGSFRCQCPAGYTGPLCENPAVP
Splooce       TARFSCRCPRGFRGPDCSLPDPCLSSPCAHGARCSVGPDGRFLCSCPPGYQGRSCRSDVDECRVGEPCRHGGTCLNTPGSFRCQCPAGYTGPLCENPAVP

Uniprot       CAPSPCRNGGTCRQSGDLTYDCACLPGFEGQNCEVNVDDCPGHRCLNGGTCVDGVNTYNCQCPPEWTGQFCTEDVDECQLQPNACHNGGTCFNTLGGHSC
Splooce       CAPSPCRNGGTCRQSGDLTYDCACLPGFEGQNCEVNVDDCPGHRCLNGGTCVDGVNTYNCQCPPEWTGQFCTEDVDECQLQPNACHNGGTCFNTLGGHSC

Uniprot       VCVNGWTGESCSQNIDDCATAVCFHGATCHDRVASFYCACPMGKTGLLCHLDDACVSNPCHEDAICDTNPVNGRAICTCPPGFTGGACDQDVDECSIGAN
Splooce       VCVNGWTGESCSQNIDDCATAVCFHGATCHDRVASFYCACPMGKTGLLCHLDDACVSNPCHEDAICDTNPVNGRAICTCPPGFTGGACDQDVDECSIGAN

Uniprot       PCEHLGRCVNTQGSFLCQCGRGYTGPRCETDVNECLSGPCRNQATCLDRIGQFTCICMAGFTGTYCEVDIDECQSSPCVNGGVCKDRVNGFSCTCPSGFS
Splooce       PCEHLGRCVNTQGSFLCQCGRGYTGPRCETDVNECLSGPCRNQATCLDRIGQFTCICMAGFTGTYCEVDIDECQSSPCVNGGVCKDRVNGFSCTCPS---

Uniprot       GSTCQLDVDECASTPCRNGAKCVDQPDGYECRCAEGFEGTLCDRNVDDCSPDPCHHGRCVDGIASFSCACAPGYTGTRCESQVDECRSQPCRHGGKCLDL
Splooce       ----------------------------------------------------------------------------------------------------

Uniprot       VDKYLCRCPSGTTGVNCEVNIDDCASNPCTFGVCRDGINRYDCVCQPGFTGPLCNVEINECASSPCGEGGSCVDGENGFRCLCPPGSLPPLCLPPSHPCA
Splooce       ----------------------------------------------------------------------------------------------------

Uniprot       HEPCSHGICYDAPGGFRCVCEPGWSGPRCSQSLARDACESQPCRAGGTCSSDGMGFHCTCPPGVQGRQCELLSPCTPNPCEHGGRCESAPGQLPVCSCPQ
Splooce       -----------------------------------------------------------------GRQCELLSPCTPNPCEHGGRCESAPGQLPVCSCPQ

Uniprot       GWQGPRCQQDVDECAGPAPCGPHGICTNLAGSFSCTCHGGYTGPSCDQDINDCDPNPCLNGGSCQDGVGSFSCSCLPGFAGPRCARDVDECLSNPCGPGT
Splooce       GWQGPRCQQDVDECAGPAPCGPHGICTNLAGSFSCTCHGGYTGPSCDQDINDCDPNPCLNGGSCQDGVGSFSCSCLPGFAGPRCARDVDECLSNPCGPGT

Uniprot       CTDHVASFTCTCPPGYGGFHCEQDLPDCSPSSCFNGGTCVDGVNSFSCLCRPGYTGAHCQHEADPCLSRPCLHGGVCSAAHPGFRCTCLESFTGPQCQTL
Splooce       CTDHVASFTCTCPPGYGGFHCEQDLPDCSPSSCFNGGTCVDGVNSFSCLCRPGYTGAHCQHEADPCLSRPCLHGGVCSAAHPGFRCTCLESFTGPQCQTL

Uniprot       VDWCSRQPCQNGGRCVQTGAYCLCPPGWSGRLCDIRSLPCREAAAQIGVRLEQLCQAGGQCVDEDSSHYCVCPEGRTGSHCEQEVDPCLAQPCQHGGTCR
Splooce       VDWCSRQPCQNGGRCVQTGAYCLCPPGWSGRLCDIRSLPCREAAAQIGVRLEQLCQAGGQCVDEDSSHYCVCPEGRTGSHCEQEVDPCLAQPCQHGGTCR

Uniprot       GYMGGYMCECLPGYNGDNCEDDVDECASQPCQHGGSCIDLVARYLCSCPPGTLGVLCEINEDDCGPGPPLDSGPRCLHNGTCVDLVGGFRCTCPPGYTGL
Splooce       GYMGGYMCECLPGYNGDNCEDDVDECASQPCQHGGSCIDLVARYLCSCPPGTLGVLCEINEDDCGPGPPLDSGPRCLHNGTCVDLVGGFRCTCPPGYTGL

Uniprot       RCEADINECRSGACHAAHTRDCLQDPGGGFRCLCHAGFSGPRCQTVLSPCESQPCQHGGQCRPSPGPGGGLTFTCHCAQPFWGPRCERVARSCRELQCPV
Splooce       RCEADINECRSGACHAAHTRDCLQDPGGGFRCLCHAGFSGPRCQTVLSPCESQPCQHGGQCRPSPGPGGGLTFTCHCAQPFWGPRCERVARSCRELQCPV

Uniprot       GVPCQQTPRGPRCACPPGLSGPSCRSFPGSPPGASNASCAAAPCLHGGSCRPAPLAPFFRCACAQGWTGPRCEAPAAAPEVSEEPRCPRAACQAKRGDQR
Splooce       GVPCQQTPRGPRCACPPGLSGPSCRSFPGSPPGASNASCAAAPCLHGGSCRPAPLAPFFRCACAQGWTGPRCEAPAAAPEVSEEPRCPRAACQAKRGDQR

Uniprot       CDRECNSPGCGWDGGDCSLSVGDPWRQCEALQCWRLFNNSRCDPACSSPACLYDNFDCHAGGRERTCNPVYEKYCADHFADGRCDQGCNTEECGWDGLDC
Splooce       CDRECNSPGCGWDGGDCSLSVGDPWRQCEALQCWRLFNNSRCDPACSSPACLYDNFDCHAGGRERTCNPVYEKYCADHFADGRCDQGCNTEECGWDGLDC

Uniprot       ASEVPALLARGVLVLTVLLPPEELLRSSADFLQRLSAILRTSLRFRLDAHGQAMVFPYHRPSPGSEPRARRELAPEVIGSVVMLEIDNRLCLQSPENDHC
Splooce       ASEVPALLARGVLVLTVLLPPEELLRSSADFLQRLSAILRTSLRFRLDAHGQAMVFPYHRPSPGSEPRARRELAPEVIGSVVMLEIDNRLCLQSPENDHC

Uniprot       FPDAQSAADYLGALSAVERLDFPYPLRDVRGEPLEPPEPSVPLLPLLVAGAVLLLVILVLGVMVARRKREHSTLWFPEGFSLHKDVASGHKGRREPVGQD
Splooce       FPDAQSAADYLGALSAVERLDFPYPLRDVRGEPLEPPEPSVPLLPLLVAGAVLLLVILVLGVMVARRKREHSTLWFPEGFSLHKDVASGHKGRREPVGQD

Uniprot       ALGMKNMAKGESLMGEVATDWMDTECPEAKRLKVEEPGMGAEEAVDCRQWTQHHLVAADIRVAPAMALTPPQGDADADGMDVNVRGPDGFTPLMLASFCG
Splooce       ALGMKNMAKGESLMGEVATDWMDTECPEAKRLKVEEPGMGAEEAVDCRQWTQHHLVAADIRVAPAMALTPPQGDADADGMDVNVRGPDGFTPLMLASFCG

Uniprot       GALEPMPTEEDEADDTSASIISDLICQGAQLGARTDRTGETALHLAARYARADAAKRLLDAGADTNAQDHSGRTPLHTAVTADAQGVFQILIRNRSTDLD
Splooce       GALEPMPTEEDEADDTSASIISDLICQGAQLGARTDRTGETALHLAARYARADAAKRLLDAGADTNAQDHSGRTPLHTAVTADAQGVFQILIRNRSTDLD

Uniprot       ARMADGSTALILAARLAVEGMVEELIASHADVNAVDELGKSALHWAAAVNNVEATLALLKNGANKDMQDSKEETPLFLAAREGSYEAAKLLLDHFANREI
Splooce       ARMADGSTALILAARLAVEGMVEELIASHADVNAVDELGKSALHWAAAVNNVEATLALLKNGANKDMQDSKEETPLFLAAREGSYEAAKLLLDHFANREI

Uniprot       TDHLDRLPRDVAQERLHQDIVRLLDQPSGPRSPPGPHGLGPLLCPPGAFLPGLKAAQSGSKKSRRPPGKAGLGPQGPRGRGKKLTLACPGPLADSSVTLS
Splooce       TDHLDRLPRDVAQERLHQDIVRLLDQPSGPRSPPGPHGLGPLLCPPGAFLPGLKAAQSGSKKSRRPPGKAGLGPQGPRGRGKKLTLACPGPLADSSVTLS

Uniprot       PVDSLDSPRPFGGPPASPGGFPLEGPYAAATATAVSLAQLGGPGRAGLGRQPPGGCVLSLGLLNPVAVPLDWARLPPPAPPGPSFLLPLAPGPQLLNPGT
Splooce       PVDSLDSPRPFGGPPASPGGFPLEGPYAAATATAVSLAQLGGPGRAGLGRQPPGGCVLSLGLLNPVAVPLDWARLPPPAPPGPSFLLPLAPGPQLLNPGT

Uniprot       PVSPQERPPPYLAVPGHGEEYPAAGAHSSPPKARFLRVPSEHPYLTPSPESPEHWASPSPPSLSDWSESTPSPATATGAMATTTGALPAQPLPLSVPSSL
Splooce       PVSPQERPPPYLAVPGHGEEYPAAGAHSSPPKARFLRVPSEHPYLTPSPESPEHWASPSPPSLSDWSESTPSPATATGAMATTTGALPAQPLPLSVPSSL

Uniprot       AQAQTQLGPQPEVTPKRQVLA
Splooce       AQAQTQLGPQPEVTPKRQVLA

----------------------------------------------------------------------------------------------------

Q63ZY3 (Uniprot)	versus
NM_015493#(-s-s-s-s-s-:19_K1698569626142) (Splooce)

For more details about the Alternative Splicing Event -> Link to Splooce page

Peptides that support the ASE (Splooce-specific):
SLQFVGVNGGMGAQL (MAXQUANT)

Alignment:
Uniprot       MAQVLHVPAPFPGTPGPASPPAFPAKDPDPPYSVETPYGYRLDLDFLKYVDDIEKGHTLRRVAVQRRPRLSSLPRGPGSWWTSTESLCSNASGDSRHSAY
Splooce       MAQVLHVPAPFPGTPGPASPPAFPAKDPDPPYSVETPYGYRLDLDFLKYVDDIEKGHTLRRVAVQRRPRLSSLPRGPGSWWTSTESLCSNASGDSRHSAY

Uniprot       SYCGRGFYPQYGALETRGGFNPRVERTLLDARRRLEDQAATPTGLGSLTPSAAGSTASLVGVGLPPPTPRSSGLSTPVPPSAGHLAHVREQMAGALRKLR
Splooce       SYCGRGFYPQYGALETRGGFNPRVERTLLDARRRLEDQAATPTGLGSLTPSAAGSTASLVGVGLPPPTPRSSGLSTPVPPSAGHLAHVREQMAGALRKLR

Uniprot       QLEEQVKLIPVLQVKLSVLQEEKRQLTVQLKSQKFLGHPTAGRGRSELCLDLPDPPEDPVALETRSVGTWVRERDLGMPDGEAALAAKVAVLETQLKKAL
Splooce       QLEEQVKLIPVLQVKLSVLQEEKRQLTVQLKSQKFLGHPTAGRGRSELCLDLPDPPEDPVALETRSVGTWVRERDLGMPDGEAALAAKVAVLETQLKKAL

Uniprot       QELQAAQARQADPQPQAWPPPDSPVRVDTVRVVEGPREVEVVASTAAGAPAQRAQSLEPYGTGLRALAMPGRPESPPVFRSQEVVETMCPVPAAATSNVH
Splooce       QELQAAQARQADPQPQAWPPPDSPVRVDTVRVVEGPREVEVVASTAAGAPAQRAQSLEPYGTGLRALAMPGRPESPPVFRSQEVVETMCPVPAAATSNVH

Uniprot       MVKKISITERSCDGAAGLPEVPAESSSSPPGSEVASLTQPEKSTGRVPTQEPTHREPTRQAASQESEEAGGTGGPPAGVRSIMKRKEEVADPTAHRRSLQ
Splooce       MVKKISITERSCDGAAGLPEVPAESSSSPPGSEVASLTQPEKSTGRVPTQEPTHREPTRQAASQESEEAGGTGGPPAGVRSIMKRKEEVADPTAHRRSLQ

Uniprot       FVGVNGGYESSSEDSSTAENISDNDSTENEAPEPRERVPSVAEAPQLRPAGTAAAKTSRQECQLSRESQHIPTAEGASGSNTEEEIRMELSPDLISACLA
Splooce       FVGVNGGMGAQL----------------------------------------------------------------------------------------

Uniprot       LEKYLDNPNALTERELKVAYTTVLQEWLRLACRSDAHPELVRRHLVTFRAMSARLLDYVVNIADSNGNTALHYSVSHANFPVVQQLLDSGVCKVDKQNRA
Splooce       ----------------------------------------------------------------------------------------------------

Uniprot       GYSPIMLTALATLKTQDDIETVLQLFRLGNINAKASQAGQTALMLAVSHGRVDVVKALLACEADVNVQDDDGSTALMCACEHGHKEIAGLLLAVPSCDIS
Splooce       ----------------------------------------------------------------------------------------------------

Uniprot       LTDRDGSTALMVALDAGQSEIASMLYSRMNIKCSFAPMSDDESPTSSSAEE
Splooce       ---------------------------------------------------

----------------------------------------------------------------------------------------------------

Q6P1N0 (Uniprot)	versus
NM_017721#(-s-s-s-s-s-s-:19_C9581966419330) (Splooce)

For more details about the Alternative Splicing Event -> Link to Splooce page

Peptides that support the ASE (Splooce-specific):
ELLELQR (PEAKS)

Alignment:
Uniprot       MHKRKGPPGPPGRGAAAARQLGLLVDLSPDGLMIPEDGANDEELEAEFLALVGGQPPALEKLKGKGPLPMEAIEKMASLCMRDPDEDEEEGTDEDDLEAD
Splooce       MHKRKGPPGPPGRGAAAARQLGLLVDLSPDGLMIPEDGANDEELEAEFLALVGGQPPALEKLKGKGPLPMEAIEKMASLCMRDPDEDEEEGTDEDDLEAD

Uniprot       DDLLAELNEVLGEEQKASETPPPVAQPKPEAPHPGLETTLQERLALYQTAIESARQAGDSAKMRRYDRGLKTLENLLASIRKGNAIDEADIPPPVAIGKG
Splooce       DDLLAELNEVLGEEQKASETPPPVAQPKPEAPHPGLETTLQERLALYQTAIESARQAGDSAKMRRYDRGLKTLENLLASIRKGNAIDEADIPPPVAIGKG

Uniprot       PASTPTYSPAPTQPAPRIASAPEPRVTLEGPSATAPASSPGLAKPQMPPGPCSPGPLAQLQSRQRDYKLAALHAKQQGDTTAAARHFRVAKSFDAVLEAL
Splooce       PASTPTYSPAPTQPAPRIASAPEPRVTLEGPSATAPASSPGLAKPQMPPGPCSPGPLAQLQSRQRDYKLAALHAKQQGDTTAAARHFRVAKSFDAVLEAL

Uniprot       SRGEPVDLSCLPPPPDQLPPDPPSPPSQPPTPATAPSTTEVPPPPRTLLEALEQRMERYQVAAAQAKSKGDQRKARMHERIVKQYQDAIRAHKAGRAVDV
Splooce       SRGEPVDLSCLPPPPDQLPPDPPSPPSQPPTPATAPSTTEVPPPPRTLLEALEQRMERYQVAAAQAKSKGDQRKARMHERIVKQYQDAIRAHKAGRAVDV

Uniprot       AELPVPPGFPPIQGLEATKPTQQSLVGVLETAMKLANQDEGPEDEEDEVPKKQNSPVAPTAQPKAPPSRTPQSGSAPTAKAPPKATSTRAQQQLAFLEGR
Splooce       AELPVPPGFPPIQGLEATKPTQQSLVGVLETAMKLANQDEGPEDEEDEVPKKQNSPVAPTAQPKAPPSRTPQSGSAPTAKAPPKATSTRAQQQLAFLEGR

Uniprot       KKQLLQAALRAKQKNDVEGAKMHLRQAKGLEPMLEASRNGLPVDITKVPPAPVNKDDFALVQRPGPGLSQEAARRYGELTKLIRQQHEMCLNHSNQFTQL
Splooce       KKQLLQAALRAKQKNDVEGAKMHLRQAKGLEPMLEASRNGLPVDITKVPPAPVNKDDFALVQRPGPGLSQEAARRYGELTKLIRQQHEMCLNHSNQFTQL

Uniprot       GNITETTKFEKLAEDCKRSMDILKQAFVRGLPTPTARFEQRTFSVIKIFPDLSSNDMLLFIVKGINLPTPPGLSPGDLDVFVRFDFPYPNVEEAQKDKTS
Splooce       GNITETTKFEKLAEDCKRSMDILKQAFVRGLPTPTARFEQRTFSVIKIFPDLSSNDMLLFIVKGINLPTPPGLSPGDLDVFVRFDFPYPNVEEAQKDKTS

Uniprot       VIKNTDSPEFKEQFKLCINRSHRGFRRAIQTKGIKFEVVHKGGLFKTDRVLGTAQLKLDALEIACEVREILEVLDGRRPTGGRLEVMVRIREPLTAQQLE
Splooce       VIKNTDSPEFKEQFKLCINRSHRGFRRAIQTKGIKFEVVHKGGLFKTDRVLGTAQLKLDALEIACEVREILELQRLRR----------------------

Uniprot       TTTERWLVIDPVPAAVPTQVAGPKGKAPPVPAPARESGNRSARPLHSLSVLAFDQERLERKILALRQARRPVPPEVAQQYQDIMQRSQWQRAQLEQGGVG
Splooce       ----------------------------------------------------------------------------------------------------

Uniprot       IRREYAAQLERQLQFYTEAARRLGNDGSRDAAKEALYRRNLVESELQRLRR
Splooce       ---------------------------------------------------

----------------------------------------------------------------------------------------------------

Q99848 (Uniprot)	versus
NM_006824#(r:1_E9494128322953) (Splooce)

For more details about the Alternative Splicing Event -> Link to Splooce page

Peptides that support the ASE (Splooce-specific):
LLCDQMWYVFFCR (MAXQUANT)

Alignment:
Uniprot       MDTPPLSDSESESDESLVTDRELQDAFSRGLLKPGLNVVLEGPKKAVNDVNGLKQCLAEFKRDLEWVERLDVTLGPVPEIGGSEAPAPQNKDQKAVDPED
Splooce       MDTPPLSDSESESDESLVTDRELQDAFSRGLLKPGLNVVLEGPKKAVNDVNGLKQCLAEFKRDLEWVERLDVTLGPVPEIGGSEAPAPQNKDQKAVDPED

Uniprot       DFQREMSFYRQAQAAVLAVLPRLHQLKVPTKRPTDYFAEMAKSDLQMQKIRQKLQTKQAAMERSEKAKQLRALRKYGKKVQTEVLQKRQQEKAHMMNAIK
Splooce       DFQREMSFYRQAQAAVLAVLPRLHQLKVPTKRPTDYFAEMAKSDLQMQKIRQKLQTKQAAMERSEKAKQLRALRKYGKKVQTEVLQKRQQEKAHMMNAIK

Uniprot       KYQKGFSDKLDFLEGDQKPLAQRKKAGAKGQQMRKGPSAKRRYKNQKFGFGGKKKGSKWNTRESYDDVSSFRAKTAHGRGLKRPGKKGSNKR----PGKR
Splooce       KYQKGFSDKLDFLEGDQKPLAQRKKAGAKGQQMRKGPSAKRRYKNQKFGFGGKKKGSKWNTRESYDDVSSFRAKTAHGRGLKRPGKKGSNVSEPRCPGEE

Uniprot       --------------------------------------TREKMKNRTH--
Splooce       QAQPPLPLPTLPSPFSSVRPHPGAEGKLLCDQMWYVFFCRRDLENEQERR

----------------------------------------------------------------------------------------------------

O75533 (Uniprot)	versus
NM_012433#(-s-s-:2_S9621630744897) (Splooce)

For more details about the Alternative Splicing Event -> Link to Splooce page

Peptides that support the ASE (Splooce-specific):
RHQVVQR (MAXQUANT)

Alignment:
Uniprot       MAKIAKTHEDIEAQIREIQGKKAALDEAQGVGLDSTGYYDQEIYGGSDSRFAGYVTSIAATELEDDDDDYSSSTSLLGQKKPGYHAPVALLNDIPQSTEQ
Splooce       ----------------------------------------------------------------------------------------------------

Uniprot       YDPFAEHRPPKIADREDEYKKHRRTMIISPERLDPFADGGKTPDPKMNARTYMDVMREQHLTKEEREIRQQLAEKAKAGELKVVNGAAASQPPSKRKRRW
Splooce       -------------------------------------------------------MRHQVVQREAR-----LLEQPQAQKYGILHLATHQR---------

Uniprot       DQTADQTPGATPKKLSSWDQAETPGHTPSLRWDETPGRAKGSETPGATPGSKIWDPTPSHTPAGAATPGRGDTPGHATPGHGGATSSARKNRWDETPKTE
Splooce       ---------------------------ELLLLDEVIHQAMR------------------HQAMEAQLPVLVKTDGMKPP---------------------

Uniprot       RDTPGHGSGWAETPRTDRGGDSIGETPTPGASKRKSRWDETPASQMGGSTPVLTPGKTPIGTPAMNMATPTPGHIMSMTPEQLQAWRWEREIDERNRPLS
Splooce       ----------------------------------KQR---------------------------------------------------------------

Uniprot       DEELDAMFPEGYKVLPPPAGYVPIRTPARKLTATPTPLGGMTGFHMQTEDRTMKSVNDQPSGNLPFLKPDDIQYFDKLLVDVDESTLSPEEQKERKIMKL
Splooce       ------------EVLPPPAGYVPIRTPARKLTATPTPLGGMTGFHMQTEDRTMKSVNDQPSGNLPFLKPDDIQYFDKLLVDVDESTLSPEEQKERKIMKL

Uniprot       LLKIKNGTPPMRKAALRQITDKAREFGAGPLFNQILPLLMSPTLEDQERHLLVKVIDRILYKLDDLVRPYVHKILVVIEPLLIDEDYYARVEGREIISNL
Splooce       LLKIKNGTPPMRKAALRQITDKAREFGAGPLFNQILPLLMSPTLEDQERHLLVKVIDRILYKLDDLVRPYVHKILVVIEPLLIDEDYYARVEGREIISNL

Uniprot       AKAAGLATMISTMRPDIDNMDEYVRNTTARAFAVVASALGIPSLLPFLKAVCKSKKSWQARHTGIKIVQQIAILMGCAILPHLRSLVEIIEHGLVDEQQK
Splooce       AKAAGLATMISTMRPDIDNMDEYVRNTTARAFAVVASALGIPSLLPFLKAVCKSKKSWQARHTGIKIVQQIAILMGCAILPHLRSLVEIIEHGLVDEQQK

Uniprot       VRTISALAIAALAEAATPYGIESFDSVLKPLWKGIRQHRGKGLAAFLKAIGYLIPLMDAEYANYYTREVMLILIREFQSPDEEMKKIVLKVVKQCCGTDG
Splooce       VRTISALAIAALAEAATPYGIESFDSVLKPLWKGIRQHRGKGLAAFLKAIGYLIPLMDAEYANYYTREVMLILIREFQSPDEEMKKIVLKVVKQCCGTDG

Uniprot       VEANYIKTEILPPFFKHFWQHRMALDRRNYRQLVDTTVELANKVGAAEIISRIVDDLKDEAEQYRKMVMETIEKIMGNLGAADIDHKLEEQLIDGILYAF
Splooce       VEANYIKTEILPPFFKHFWQHRMALDRRNYRQLVDTTVELANKVGAAEIISRIVDDLKDEAEQYRKMVMETIEKIMGNLGAADIDHKLEEQLIDGILYAF

Uniprot       QEQTTEDSVMLNGFGTVVNALGKRVKPYLPQICGTVLWRLNNKSAKVRQQAADLISRTAVVMKTCQEEKLMGHLGVVLYEYLGEEYPEVLGSILGALKAI
Splooce       QEQTTEDSVMLNGFGTVVNALGKRVKPYLPQICGTVLWRLNNKSAKVRQQAADLISRTAVVMKTCQEEKLMGHLGVVLYEYLGEEYPEVLGSILGALKAI

Uniprot       VNVIGMHKMTPPIKDLLPRLTPILKNRHEKVQENCIDLVGRIADRGAEYVSAREWMRICFELLELLKAHKKAIRRATVNTFGYIAKAIGPHDVLATLLNN
Splooce       VNVIGMHKMTPPIKDLLPRLTPILKNRHEKVQENCIDLVGRIADRGAEYVSAREWMRICFELLELLKAHKKAIRRATVNTFGYIAKAIGPHDVLATLLNN

Uniprot       LKVQERQNRVCTTVAIAIVAETCSPFTVLPALMNEYRVPELNVQNGVLKSLSFLFEYIGEMGKDYIYAVTPLLEDALMDRDLVHRQTASAVVQHMSLGVY
Splooce       LKVQERQNRVCTTVAIAIVAETCSPFTVLPALMNEYRVPELNVQNGVLKSLSFLFEYIGEMGKDYIYAVTPLLEDALMDRDLVHRQTASAVVQHMSLGVY

Uniprot       GFGCEDSLNHLLNYVWPNVFETSPHVIQAVMGALEGLRVAIGPCRMLQYCLQGLFHPARKVRDVYWKIYNSIYIGSQDALIAHYPRIYNDDKNTYIRYEL
Splooce       GFGCEDSLNHLLNYVWPNVFETSPHVIQAVMGALEGLRVAIGPCRMLQYCLQGLFHPARKVRDVYWKIYNSIYIGSQDALIAHYPRIYNDDKNTYIRYEL

Uniprot       DYIL
Splooce       DYIL

----------------------------------------------------------------------------------------------------

UNIPROT? (Uniprot)	versus
NM_019862#(f-:16_A1480001279587) (Splooce)

For more details about the Alternative Splicing Event -> Link to Splooce page

Peptides that support the ASE (Splooce-specific):
GVTGVSGPGK (MAXQUANT)

Alignment:
Uniprot       -------
Splooce       MALRGFCSADGSDPLWDWNVTWNTSNPDFTKCFQNTVLVWVPCFYLWACFPFYFLYLSRHDRGYIQMTPLNKTKTALGFLLWIVCWADLFYSFWERSRGI

Uniprot       -------
Splooce       FLAPVFLVSPTLLGITMLLATFLIQLERRKGVQSSGIMLTFWLVALVCALAILRSKIMTALKEDAQVDLFRDITFYVYFSLLLIQLVLSCFSDRSPLFSE

Uniprot       -------
Splooce       TIHDPNPCPESSASFLSRITFWWITGLIVRGYRQPLEGSDLWSLNKEDTSEQVVPVLVKNWKKECAKTRKQPVKVVYSSKDPAQPKESSKVDANEEVEAL

Uniprot       -------
Splooce       IVKSPQKEWNPSLFKVLYKTFGPYFLMSFFFKAIHDLMMFSGPQILKLLIKFVNDTKAPDWQGYFYTVLLFVTACLQTLVLHQYFHICFVSGMRIKTAVI

Uniprot       -------
Splooce       GAVYRKALVITNSARKSSTVGEIVNLMSVDAQRFMDLATYINMIWSAPLQVILALYLLWLNLGPSVLAGVAVMVLMVPVNAVMAMKTKTYQVAHMKSKDN

Uniprot       -------
Splooce       RIKLMNEILNGIKVLKLYAWELAFKDKVLAIRQEELKVLKKSAYLSAVGTFTWVCTPFLVALCTFAVYVTIDENNILDAQTAFVSLALFNILRFPLNILP

Uniprot       -------
Splooce       MVISSIVQASVSLKRLRIFLSHEELEPDSIERRPVKDGGGTNSITVRNATFTWARSDPPTLNGITFSIPEGALVAVVGQVGCGKSSLLSALLAEMDKVEG

Uniprot       -------
Splooce       HVAIKGVNLSGGQKQRVSLARAVYSNADIYLFDDPLSAVDAHVGKHIFENVIGPKGMLKNKTRILVTHSMSYLPQVDVIIVMSGGKISEMGSYQELLARD

Uniprot       -------
Splooce       GAFAEFLRTYASTEQEQDAEENGRGVTGVSGPGKEAKQMENGMLVTDSAGKQLQRQLSSSSSYSGDISRHHNSTAELQKAEAKKEETWKLMEADKAQTGQ

Uniprot       -------
Splooce       VKLSVYWDYMKAIGLFISFLSIFLFMCNHVSALASNYWLSLWTDDPIVNGTQEHTKVRLSVYGALGISQGIAVFGYSMAVSIGGILASRCLHVDLLHSIL

Uniprot       -------
Splooce       RSPMSFFERTPSGNLVNRFSKELDTVDSMIPEVIKMFMGSLFNVIGACIVILLATPIAAIIIPPLGLIYFFVQRFYVASSRQLKRLESVSRSPVYSHFNE

Uniprot       -------
Splooce       TLLGVSVIRAFEEQERFIHQSDLKVDENQKAYYPSIVANRWLAVRLECVGNCIVLFAALFAVISRHSLSAGLVGLSVSYSLQVTTYLNWLVRMSSEMETN

Uniprot       -------
Splooce       IVAVERLKEYSETEKEAPWQIQETAPPSSWPQVGRVEFRNYCLRYREDLDFVLRHINVTINGGEKVGIVGRTGAGKSSLTLGLFRINESAEGEIIIDGIN

Uniprot       -------
Splooce       IAKIGLHDLRFKITIIPQDPVLFSGSLRMNLDPFSQYSDEEVWTSLELAHLKDFVSALPDKLDHECAEGGENLSVGQRQLVCLARALLRKTKILVLDEAT

Uniprot       -------
Splooce       AAVDLETDDLIQSTIRTQFEDCTVLTIAHRLNTIMDYTRVIVLDKGEIQEYGAPSDLLQQRGLFYSMAKDAGLV

----------------------------------------------------------------------------------------------------

Q15435 (Uniprot)	versus
NM_002712#(-s-:2_P3498135645060) (Splooce)

For more details about the Alternative Splicing Event -> Link to Splooce page

Peptides that support the ASE (Splooce-specific):
DVDLNHYR (MAXQUANT)

Alignment:
Uniprot       MAAERGAGQQQSQEMMEVDRRVESEESGDEEGKKHSSGIVADLSEQSLKDGEERGEEDPEEEHELPVDMETINLDRDAEDVDLNHYRIGKIEGFEVLKKV
Splooce       --------------------------MGRSGGR------------------------------------------RTQKDVDLNHYRIGKIEGFEVLKKV

Uniprot       KTLCLRQNLIKCIENLEELQSLRELDLYDNQIKKIENLEALTELEILDISFNLLRNIEGVDKLTRLKKLFLVNNKISKIENLSNLHQLQMLELGSNRIRA
Splooce       KTLCLRQNLIKCIENLEELQSLRELDLYDNQIKKIENLEALTELEILDISFNLLRNIEGVDKLTRLKKLFLVNNKISKIENLSNLHQLQMLELGSNRIRA

Uniprot       IENIDTLTNLESLFLGKNKITKLQNLDALTNLTVLSMQSNRLTKIEGLQNLVNLRELYLSHNGIEVIEGLENNNKLTMLDIASNRIKKIENISHLTELQE
Splooce       IENIDTLTNLESLFLGKNKITKLQNLDALTNLTVLSMQSNRLTKIEGLQNLVNLRELYLSHNGIEVIEGLENNNKLTMLDIASNRIKKIENISHLTELQE

Uniprot       FWMNDNLLESWSDLDELKGARSLETVYLERNPLQKDPQYRRKVMLALPSVRQIDATFVRF
Splooce       FWMNDNLLESWSDLDELKGARSLETVYLERNPLQKDPQYRRKVMLALPSVRQIDATFVRF

----------------------------------------------------------------------------------------------------

Q08378 (Uniprot)	versus
NM_005895#(-s-:12_G9718448281757) (Splooce)

For more details about the Alternative Splicing Event -> Link to Splooce page

Peptides that support the ASE (Splooce-specific):
QIEELQQEARKK (MAXQUANT)

Alignment:
Uniprot       MDGASAEQDGLQEDRSHSGPSSLPEAPLKPPGPLVPPDQQDKVQCAEVNRASTEGESPDGPGQGGLCQNGPTPPFPDPPSSLDPTTSPVGPDASPGVAGF
Splooce       MDGASAEQDGLQEDRSHSGPSSLPEAPLKPPGPLVPPDQQDKVQCAEVNRASTEGESPDGPGQGGLCQNGPTPPFPDPPSSLDPTTSPVGPDASPGVAGF

Uniprot       HDNLRKSQGTSAEGSVRKEALQSLRLSLPMQETQLCSTDSPLPLEKEEQVRLQARKWLEEQLKQYRVKRQQERSSQPATKTRLFSTLDPELMLNPENLPR
Splooce       HDNLRKSQGTSAEGSVRKEALQSLRLSLPMQETQLCSTDSPLPLEKEEQVRLQARKWLEEQLKQYRVKRQQERSSQPATKTRLFSTLDPELMLNPENLPR

Uniprot       ASTLAMTKEYSFLRTSVPRGPKVGSLGLPAHPREKKTSKSSKIRSLADYRTEDSNAGNSGGNVPAPDSTKGSLKQNRSSAASVVSEISLSPDTDDRLENT
Splooce       ASTLAMTKEYSFLRTSVPRGPKVGSLGLPAHPREKKTSKSSKIRSLADYRTEDSNAGNSGGNVPAPDSTKGSLKQNRSSAASVVSEISLSPDTDDRLENT

Uniprot       SLAGDSVSEVDGNDSDSSSYSSASTRGTYGILSKTVGTQDTPYMVNGQEIPADTLGQFPSIKDVLQAAAAEHQDQGQEVNGEVRSRRDSICSSVSLESSA
Splooce       SLAGDSVSEVDGNDSDSSSYSSASTRGTYGILSKTVGTQDTPYMVNGQEIPADTLGQFPSIKDVLQAAAAEHQDQGQEVNGEVRSRRDSICSSVSLESSA

Uniprot       AETQEEMLQVLKEKMRLEGQLEALSLEASQALKEKAELQAQLAALSTKLQAQVECSHSSQQRQDSLSSEVDTLKQSCWDLERAMTDLQNMLEAKNASLAS
Splooce       AETQEEMLQVLKEKMRLEGQLEALSLEASQALKEKAELQAQLAALSTKLQAQVECSHSSQQRQDSLSSEVDTLKQSCWDLERAMTDLQNMLEAKNASLAS

Uniprot       SNNDLQVAEEQYQRLMAKVEDMQRSMLSKDNTVHDLRQQMTALQSQLQQVQLERTTLTSKLKASQAEISSLQSVRQWYQQQLALAQEARVRLQGEMAHIQ
Splooce       SNNDLQVAEEQYQRLMAKVEDMQRSMLSKDNTVHDLRQQMTALQSQLQQVQLERTTLTSKLKASQAEISSLQSVRQWYQQQLALAQEARVRLQGEMAHIQ

Uniprot       VGQMTQAGLLEHLKLENVSLSQQLTETQHRSMKEKGRIAAQLQGIEADMLDQEAAFMQIQEAKTMVEEDLQRRLEEFEGERERLQRMADSAASLEQQLEQ
Splooce       VGQMTQAGLLEHLKLENVSLSQQLTETQHRSMKEKGRIAAQLQGIEADMLDQEAAFMQIQEAKTMVEEDLQRRLEEFEGERERLQRMADSAASLEQQLEQ

Uniprot       VKLTLLQRDQQLEALQQEHLDLMKQLTLTQEALQSREQSLDALQTHYDELQARLGELQGEAASREDTICLLQNEKIILEAALQAAKSGKEELDRGARRLE
Splooce       VKLTLLQRDQQLEALQQEHLDLMKQLTLTQEALQSREQSLDALQTHYDELQARLGELQGEAASREDTICLLQNEKIILEAALQAAKSGKEELDRGARRLE

Uniprot       EGTEETSETLEKLREELAIKSGQVEHLQQETAALKKQMQKIKEQFLQQKVMVEAYRRDATSKDQLISELKATRKRLDSELKELRQELMQVHGEKRTAEAE
Splooce       EGTEETSETLEKLREELAIKSGQVEHLQQETAALKKQMQKIKEQFLQQKVMVEAYRRDATSKDQLISELKATRKRLDSELKELRQELMQVHGEKRTAEAE

Uniprot       LSRLHREVAQVRQHMADLEGHLQSAQKERDEMETHLQSLQFDKEQMVAVTEANEALKKQIEELQQEARKAITEQKQKMRRLGSDLTSAQKEMKTKHKAYE
Splooce       LSRLHREVAQVRQHMADLEGHLQSAQKERDEMETHLQSLQFDKEQMVAVTEANEALKKQIEELQQEARKKGSRPWRRSCRLS------------------

Uniprot       NAVGILSRRLQEALAAKEAADAELGQLRAQGGSSDSSLALHERIQALEAELQAVSHSKTLLEKELQEVIALTSQELEESREKVLELEDELQESRGFRKKI
Splooce       ----VIARRCWKRNCRRS----------------------------------------------------------------------------------

Uniprot       KRLEESNKKLALELEHEKGKLTGLGQSNAALREHNSILETALAKREADLVQLNLQVQAVLQRKEEEDRQMKHLVQALQASLEKEKEKVNSLKEQVAAAKV
Splooce       ----------------------------------------------------------------------------------------------------

Uniprot       EAGHNRRHFKAASLELSEVKKELQAKEHLVQKLQAEADDLQIREGKHSQEIAQFQAELAEARAQLQLLQKQLDEQLSKQPVGNQEMENLKWEVDQKEREI
Splooce       ----------------------------------------------------------------------------------------------------

Uniprot       QSLKQQLDLTEQQGRKELEGLQQLLQNVKSELEMAQEDLSMTQKDKFMLQAKVSELKNNMKTLLQQNQQLKLDLRRGAAKTRKEPKGEASSSNPATPIKI
Splooce       ----------------------------------------------------------------------------------------------------

Uniprot       PDCPVPASLLEELLRPPPAVSKEPLKNLNSCLQQLKQEMDSLQRQMEEHALTVHESLSSWTPLEPATASPVPPGGHAGPRGDPQRHSQSRASKEGPGE
Splooce       --------------------------------------------------------------------------------------------------

----------------------------------------------------------------------------------------------------

O96005 (Uniprot)	versus
NM_001294#(-s-s-s-:19_C4449420006763) (Splooce)

For more details about the Alternative Splicing Event -> Link to Splooce page

Peptides that support the ASE (Splooce-specific):
GSVPPPLDQSGPR (MAXQUANT)

Alignment:
Uniprot       MAAAQEADGARSAVVAAGGGSSGQVTSNGSIGRDPPAETQPQNPPAQPAPNAWQVIKGVLFRIFIIWAISSWFRRGPAPQDQAGPGGAPRVASRNLFPKD
Splooce       MAAAQEADGARSAVVAAGGGSSGQVTSNGSIGRDPPAETQPQNPPAQPAPNAWQVIKGVLFRIFIIWAISSWFRRGPAPQDQAGPGGAPRVASRNLFPKD

Uniprot       TLMNLHVYISEHEHFTDFNATSALFWEQHDLVYGDWTSGENSDGCYEHFAELDIPQSVQQNGSIYIHVYFTKSGFHPDPRQKALYRRLATVHMSRMINKY
Splooce       TLMNLHVYISEHEHFTDFNATSALFWEQHDLVYGDWTSGENSDGCYEHFAELDIPQSVQQNGSIYIHVYFTKSGFHPDPRQKALYRRLATVHMSRMINKY

Uniprot       KRRRFQKTKNLLTGETEADPEMIKRAEDYGPVEVISHWHPNITINIVDDHTPWVKGSVPPPLDQYVKFDAVSGDYYPIIYFNDYWNLQKDYYPINESLAS
Splooce       KRRRFQKTKNLLTGETEADPEMIKRAEDYGPVEVISHWHPNITINIVDDHTPWVKGSVPPPLDQ-------SG-------------------PRAQGGRN

Uniprot       LPLRVSFCPLSLWRWQLYAAQSTKSPWNFLGDELYEQSDEEQDSVKVALLETNPYLLALTIIVSIVHSVFEFLAFKNDIQFWNSRQSLEGLSVRSVFFGV
Splooce       LPP-----------------------------------------------------------------------------------------------PI

Uniprot       FQSFVVLLYILDNETNFVVQVSVFIGVLIDLWKITKVMDVRLDREHRVAGIFPRLSFKDKSTYIESSTKVYDDMAFRYLSWILFPLLGCYAVYSLLYLEH
Splooce       LQGQVHVYRVLDQSV-------------------------------------------------------------------------------------

Uniprot       KGWYSWVLSMLYGFLLTFGFITMTPQLFINYKLKSVAHLPWRMLTYKALNTFIDDLFAFVIKMPVMYRIGCLRDDVVFFIYLYQRWIYRVDPTRVNEFGM
Splooce       ----------------------------------------------------------------------------------------------------

Uniprot       SGEDPTAAAPVAEVPTAAGALTPTPAPTTTTATREEASTSLPTKPTQGASSASEPQEAPPKPAEDKKKD
Splooce       ---------------------------------------------------------------------

----------------------------------------------------------------------------------------------------

Q15052 (Uniprot)	versus
NM_004840#(-s-s-S-:X_A8466179310879) (Splooce)

For more details about the Alternative Splicing Event -> Link to Splooce page

Peptides that support the ASE (Splooce-specific):
LNPVAGLK (MAXQUANT)

Alignment:
Uniprot       MNPEEQIVTWLISLGVLESPKKTICDPEEFLKSSLKNGVVLCKLINRLMPGSVEKFCLDPQTEADCINNINDFLKGCATLQVEIFDPDDLYSGVNFSKVL
Splooce       ----------------------------------------------------------------------------------------------------

Uniprot       STLLAVNKATEDQLSERPCGRSSSLSAANTSQTNPQGAVSSTVSGLQRQSKTVEMTENGSHQLIVKARFNFKQTNEDELSVCKGDIIYVTRVEEGGWWEG
Splooce       ------------------MGEQAGSPVIMSVKLNP-------VAGLKLL---------GSSDPPALAFQSAGITEMEFRSCCPG-------------WS-

Uniprot       TLNGRTGWFPSNYVREIKSSERPLSPKAVKGFETAPLTKNYYTVVLQNILDTEKEYAKELQSLLVTYLRPLQSNNNLSTVEVTSLLGNFEEVCTFQQTLC
Splooce       ------------------------------AMARSQLT------VLQNILDTEKEYAKELQSLLVTYLRPLQSNNNLSTVEVTSLLGNFEEVCTFQQTLC

Uniprot       QALEECSKFPENQHKVGGCLLSLMPHFKSMYLAYCANHPSAVNVLTQHSDELEQFMENQGASSPGILILTTNLSKPFMRLEKYVTLLQELERHMEDTHPD
Splooce       QALEECSKFPENQHKVGGCLLSLMPHFKSMYLAYCANHPSAVNVLTQHSDELEQFMENQGASSPGILILTTNLSKPFMRLEKYVTLLQELERHMEDTHPD

Uniprot       HQDILKAIVAFKTLMGQCQDLRKRKQLELQILSEPIQAWEGEDIKNLGNVIFMSQVMVQYGACEEKEERYLMLFSNVLIMLSASPRMSGFIYQGKIPIAG
Splooce       HQDILKAIVAFKTLMGQCQDLRKRKQLELQILSEPIQAWEGEDIKNLGNVIFMSQVMVQYGACEEKEERYLMLFSNVLIMLSASPRMSGFIYQGKIPIAG

Uniprot       TVVTRLDEIEGNDCTFEITGNTVERIVVHCNNNQDFQEWLEQLNRLIRGPASCSSLSKTSSSSCSAHSSFSSTGQPRGPLEPPQIIKPWSLSCLRPAPPL
Splooce       TVVTRLDEIEGNDCTFEITGNTVERIVVHCNNNQDFQEWLEQLNRLIRGPASCSSLSKTSSSSCSAHSSFSSTGQPRGPLEPPQIIKPWSLSCLRPAPPL

Uniprot       RPSAALGYKERMSYILKESSKSPKTMKKFLHKRKTERKPSEEEYVIRKSTAALEEDAQILKVIEAYCTSANFQQGHGSSTRKDSIPQVLLPEEEKLIIEE
Splooce       RPSAALGYKERMSYILKESSKSPKTMKKFLHKRKTERKPSEEEYVIRKSTAALEEDAQILKVIEAYCTSANFQQGHGSSTRKDSIPQVLLPEEEKLIIEE

Uniprot       TRSNGQTIMEEKSLVDTVYALKDEVRELKQENKRMKQCLEEELKSRRDLEKLVRRLLKQTDECIRGESSSKTSILP
Splooce       TRSNGQTIMEEKSLVDTVYALKDEVRELKQENKRMKQCLEEELKSRRDLEKLVRRLLKQTDECIRGESSSKTSILP

----------------------------------------------------------------------------------------------------

Q9UBU9 (Uniprot)	versus
NM_006362#(-s-s-s-s-s-s-:11_N3318526494301) (Splooce)

For more details about the Alternative Splicing Event -> Link to Splooce page

Peptides that support the ASE (Splooce-specific):
SLVLHFLQQCLQDNNWDYTR (MAXQUANT)

Alignment:
Uniprot       MADEGKSYSEHDDERVNFPQRKKKGRGPFRWKYGEGNRRSGRGGSGIRSSRLEEDDGDVAMSDAQDGPRVRYNPYTTRPNRRGDTWHDRDRIHVTVRRDR
Splooce       MADEGKSYSEHDDERVNFPQRKKKGRGPFRWKYGEGNRRSGRGGSGIRSSRLEEDDGDVAMSDAQDGPRVRYNPYTTRPNRRGDTWHDRDRIHVTVRRDR

Uniprot       APPERGGAGTSQDGTSKNWFKITIPYGRKYDKAWLLSMIQSKCSVPFTPIEFHYENTRAQFFVEDASTASALKAVNYKILDRENRRISIIINSSAPPHTI
Splooce       APPERGGAGTSQDGTSKNWFKITIPYGRKYDKAWLLSMIQSKCSVPFTPIEFHYENTRAQFFVEDASTASALKAVNYKILDRENRRISIIINSSAPPHTI

Uniprot       LNELKPEQVEQLKLIMSKRYDGSQQALDLKGLRSDPDLVAQNIDVVLNRRSCMAATLRIIEENIPELLSLNLSNNRLYRLDDMSSIVQKAPNLKILNLSG
Splooce       LNELKPEQVEQLKLIMSKRYDGSQQALDLKGLRSDPDLVAQNIDVVLNRRSCMAATLRIIEENIPELLSLNLSNNRLYRLDDMSSIVQKAPNLKILNLSG

Uniprot       NELKSERELDKIKGLKLEELWLDGNSLCDTFRDQSTYISAIRERFPKLLRLDGHELPPPIAFDVEAPTTLPPCKGSYFGTENLKSLVLHFLQQYYAIYDS
Splooce       NELKSERELDKIKGLKLEELWLDGNSLCDTFRDQSTYISAIRERFPKLLRLDGHELPPPIAFDVEAPTTLPPCKGSYFGTENLKSLVLHFLQQ-------

Uniprot       GDRQGLLDAYHDGACCSLSIPFIPQNPARSSLAEYFKDSRNVKKLKDPTLRFRLLKHTRLNVVAFLNELPKTQHDVNSFVVDISAQTSTLLCFSVNGVFK
Splooce       ----------------------------------------------------------------------------------------------------

Uniprot       EVDGKSRDSLRAFTRTFIAVPASNSGLCIVNDELFVRNASSEEIQRAFAMPAPTPSSSPVPTLSPEQQEMLQAFSTQSGMNLEWSQKCLQDNNWDYTRSA
Splooce       ---------------------------------------------------------------------------------------CLQDNNWDYTRSA

Uniprot       QAFTHLKAKGEIPEVAFMK
Splooce       QAFTHLKAKGEIPEVAFMK

----------------------------------------------------------------------------------------------------

P63092 (Uniprot)	versus
NM_001077488#(f-:20_G1806892836259) (Splooce)

For more details about the Alternative Splicing Event -> Link to Splooce page

Peptides that support the ASE (Splooce-specific):
LQGATAMGSSEK (MAXQUANT)

Alignment:
Uniprot       MGCLGNSKTEDQRNEEKAQREANKKIEKQLQKDKQVYRATHRLLLLGAGESGKSTIVKQMRILHVNGFNGEGGEEDPQAARSNSDGSEKATKVQDIKNNL
Splooce       -------------------------------------------MLMGLMER----AAKRTRRLQG-------------ATAMGS--SEKATKVQDIKNNL

Uniprot       KEAIETIVAAMSNLVPPVELANPENQFRVDYILSVMNVPDFDFPPEFYEHAKALWEDEGVRACYERSNEYQLIDCAQYFLDKIDVIKQADYVPSDQDLLR
Splooce       KEAIETIVAAMSNLVPPVELANPENQFRVDYILSVMNVPDFDFPPEFYEHAKALWEDEGVRACYERSNEYQLIDCAQYFLDKIDVIKQADYVPSDQDLLR

Uniprot       CRVLTSGIFETKFQVDKVNFHMFDVGGQRDERRKWIQCFNDVTAIIFVVASSSYNMVIREDNQTNRLQEALNLFKSIWNNRWLRTISVILFLNKQDLLAE
Splooce       CRVLTSGIFETKFQVDKVNFHMFDVGGQRDERRKWIQCFNDVTAIIFVVASSSYNMVIREDNQTNRLQEALNLFKSIWNNRWLRTISVILFLNKQDLLAE

Uniprot       KVLAGKSKIEDYFPEFARYTTPEDATPEPGEDPRVTRAKYFIRDEFLRISTASGDGRHYCYPHFTCAVDTENIRRVFNDCRDIIQRMHLRQYELL
Splooce       KVLAGKSKIEDYFPEFARYTTPEDATPEPGEDPRVTRAKYFIRDEFLRISTASGDGRHYCYPHFTCAVDTENIRRVFNDCRDIIQRMHLRQYELL

----------------------------------------------------------------------------------------------------

Q14444 (Uniprot)	versus
NM_203364#(f-T:11_C8341575471969) (Splooce)

For more details about the Alternative Splicing Event -> Link to Splooce page

Peptides that support the ASE (Splooce-specific):
VPGLDDYQER (MAXQUANT)

Alignment:
Uniprot       MPSATSHSGSGSKSSGPPPPSGSSGSEAAAGAGAAAPASQHPATGTGAVQTEAMKQILGVIDKKLRNLEKKKGK=LDDYQERMNKGERLNQDQLDAVSKY
Splooce       MPSATSHSGSGSKSSGPPPPSGSSGSEAAAGAGAAAPASQHPATGTGAVQTEAMKQILGVIDKKLRNLEKKKVPGLDDYQERMNKGERLNQDQLDAVSKY

Uniprot       QEVTNNLEFAKELQRSFMALSQDIQKTIKKTARREQLMREEAEQKRLKTVLELQYVLDKLGDDEVRTDLKQGLNGVPILSEEELSLLDEFYKLVDPERDM
Splooce       QEVTNNLEFAKELQRSFMALSQDIQKTIKKTARREQLMREEAEQKRLKTVLELQYVLDKLGDDEVRTDLKQGLNGVPILSEEELSLLDEFYKLVDPERDM

Uniprot       SLRLNEQYEHASIHLWDLLEGKEKPVCGTTYKVLKEIVERVFQSNYFDSTHNHQNGLCEEEEAASAPAVEDQVPEAEPEPAEEYTEQSEVESTEYVNRQF
Splooce       SLRLNEQYEHASIHLWDLLEGKEKPVCGTTYKVLKEIVERVFQSNYFDSTHNHQNGLCEEEEAASAPAVEDQVPEAEPEPAEEYTEQSEVESTEYVNRQF

Uniprot       MAETQFTSGEKEQVDEWTVETVEVVNSLQQQPQAASPSVPEPHSLTPVAQADPLVRRQRVQDLMAQMQGPYNFIQDSMLDFENQTLDPAIVSAQPMNPTQ
Splooce       MAETQFTSGEKEQVDEWTVETVEVVNSLQQQPQAASPSVPEPHSLTPVAQADPLVRRQRVQDLMAQMQGPYNFIQDSMLDFENQTLDPAIVSAQPMNPTQ

Uniprot       NMDMPQLVCPPVHSESRLAQPNQVPVQPEATQVPLVSSTSEGYTASQPLYQPSHATEQRPQKEPIDQIQATISLNTDQTTASSSLPAASQPQVFQAGTSK
Splooce       NMDMPQLVCPPVHSESRLAQPNQVPVQPEATQVPLVSSTSEGYTASQPLYQPSHATEQRPQKEPIDQIQATISLNTDQTTASSSLPAASQPQVFQAGTSK

Uniprot       PLHSSGINVNAAPFQSMQTVFNMNAPVPPVNEPETLKQQNQYQASYNQSFSSQPHQVEQTELQQEQLQTVVGTYHGSPDQSHQVTGNHQQPPQQNTGFPR
Splooce       PLHSSGINVNAAPFQSMQTVFNMNAPVPPVNEPETLKQQNQYQASYNQSFSSQPHQVEQTELQQEQLQTVVGTYHGSPDQSHQVTGNHQQPPQQNTGFPR

Uniprot       SNQPYYNSRGVSRGGSRGARGLMNGYRGPANGFRGGYDGYRPSFSNTPNSGYTQSQFSAPRDYSGYQRDGYQQNFKRGSGQSGPRGAPRGNILWW
Splooce       SNQPYYNSRGVSRGGSRGARGLMNGYRGPANGFRGGYDGYRPSFSNTPNSGYTQSQFSAPRDYSGYQRDGYQQNFKRGSGQSGPRGAPRGNILWW

----------------------------------------------------------------------------------------------------

Q13435 (Uniprot)	versus
NM_006842#(f-:11_S1862761049899) (Splooce)

For more details about the Alternative Splicing Event -> Link to Splooce page

Peptides that support the ASE (Splooce-specific):
QMALSLLEAFHQCLLEWR (MAXQUANT)

Alignment:
Uniprot       MATEHPEPPKAELQLPPPPPPGHYGAWAAQELQAKLAEIGAPIQGNREELVERLQSYTRQTGIVLNRPVLRGEDGDKAAPPPMSAQLPGIPMPPPPLGLP
Splooce       MATEHPEPPKAELQLPPPPPPGHYGAWAAQELQAKLAEIGAPIQGNREELVERLQSYTRQTGIVLNRPVLRGEDGDKAAPPPMSAQLPGIPMPPPPLGLP

Uniprot       PLQPPPPPPPPPPGLGLGFPMAHPPNLGPPPPLRVGEPVALSEEERLKLAQQQAALLMQQEERAKQQGDHSLKEHELLEQQKRAAVLLEQERQQEIAKMG
Splooce       PLQPPPPPPPPPPGLGLGFPMAHPPNLGPPPPLRVGEPVALSEEERLKLAQQQAALLMQQEERAKQQGDHSLKEHELLEQQKRAAVLLEQERQQEIAKMG

Uniprot       TPVPRPPQDMGQIGVRTPLGPRVAAPVGPVGPTPTVLPMGAPVPRPRGPPPPPGDENREMDDPSVGPKIPQALEKILQLKESRQEEMNSQQEEEEMETDA
Splooce       TPVPRPPQDMGQIGVRTPLGPRVAAPVGPVGPTPTVLPMGAPVPRPRGPPPPPGDENREMDDPSVGPKIPQALEKILQLKESRQEEMNSQQEEEEMETDA

Uniprot       RSSLGQSASETEEDTVSVSKKEKNRKRRNRKKKKKPQRVRGVSSESSGDREKDSTRSRGSDSPAADVEIEYVTEEPEIYEPNFIFFKRIFEAFKLTDDVK
Splooce       RSSLGQSASETEEDTVSVSKKEKNRKRRNRKKKKKPQRVRGVSSESSGDREKDSTRSRGSDSPAADVEIEYVTEEPEIYEPNFIFFKRIFEAFKLTDDVK

Uniprot       KEKEKEPEKLDKLENSAAPKKKGFEEEHKDSDDDSSDDEQEKKPEAPKLSKKKLRRMNRFTVAELKQLVARPDVVEMHDVTAQDPKLLVHLKATRNSVPV
Splooce       KEKEKEPEKLDKLENSAAPKKKGFEEEHKDSDDDSSDDEQEKKPEAPKLSKKKLRRMNRFTVAELKQLVARPDVVEMHDVTAQDPKLLVHLKATRNSVPV

Uniprot       PRHWCFKRKYLQGKRGIEKPPFELPDFIKRTGIQEMREALQEKEEQKTMKSKMREKVRPKMGKIDIDYQKLHDAFFKWQTKPKLTIHGDLYYEGKEFETR
Splooce       PRHWCFKRKYLQGKRGIEKPPFELPDFIKRTGIQEMREALQEKEEQKTMKSKMREKVRPKMGKIDIDYQKLHDAFFKWQTKPKLTIHGDLYYEGKEFETR

Uniprot       LKEKKPGDLSDELRISLGMPVGPNAHKVPPPWLIAMQRYGPPPSYPNLKIPGLNSPIPESCSFGYHAGGWGKPPVDETGKPLYGDVFGTNAAEFQTKTEE
Splooce       LKEKKPGDLSDELRISLGMPVGPNAHKVPPPWLIAMQRYGPPPSYPNLKIPGLNSPIPESCSFGYHAGGWGKPPVDETGKPLYGDVFGTNAAEFQTKTEE

Uniprot       EEIDRTPWGELEPSDEESSEEEEEEESDEDKPDETGFITPADSGLITPGGFSSVPAGMETPELIELRKKKIEEAMDGSETPQLFTVLPEKRTATVGGAMM
Splooce       EEIDRTPWGELEPSDEESSEEEEEEESDEDKPDETGFITPADR----------------------------------------------------WGKQM

Uniprot       GSTHIYDMSTVMSRKGPAPELQGVEVALAPEELELDPMAMTQKYEEHVREQQAQVEKEDFSDMVAEHAAKQKQKKRKAQPQDSRGGSKKYKEFKF
Splooce       ALSLLEAFHQCLLEWRPLNSLS-------------------------------------------------------------------------

----------------------------------------------------------------------------------------------------

O14578 (Uniprot)	versus
NM_007174#(f-:12_C8115714254569) (Splooce)

For more details about the Alternative Splicing Event -> Link to Splooce page

Peptides that support the ASE (Splooce-specific):
SLEQEYQAQVEEMR (MAXQUANT)

Alignment:
Uniprot       MLKFKYGARNPLDAGAAEPIASRASRLNLFFQGKPPFMTQQQMSPLSREGILDALFVLFEECSQPALMKIKHVSNFVRKYSDTIAELQELQPSAKDFEVR
Splooce       MLKFKYGARNPLDAGAAEPIASRASRLNLFFQGKPPFMTQQQMSPLSREGILDALFVLFEECSQPALMKIKHVSNFVRKYSDTIAELQELQPSAKDFEVR

Uniprot       SLVGCGHFAEVQVVREKATGDIYAMKVMKKKALLAQEQVSFFEEERNILSRSTSPWIPQLQYAFQDKNHLYLVMEYQPGGDLLSLLNRYEDQLDENLIQF
Splooce       SLVGCGHFAEVQVVREKATGDIYAMKVMKKKALLAQEQVSFFEEERNILSRSTSPWIPQLQYAFQDKNHLYLVMEYQPGGDLLSLLNRYEDQLDENLIQF

Uniprot       YLAELILAVHSVHLMGYVHRDIKPENILVDRTGHIKLVDFGSAAKMNSNKMVNAKLPIGTPDYMAPEVLTVMNGDGKGTYGLDCDWWSVGVIAYEMIYGR
Splooce       YLAELILAVHSVHLMGYVHRDIKPENILVDRTGHIKLVDFGSAAKMNSNKMVNAKLPIGTPDYMAPEVLTVMNGDGKGTYGLDCDWWSVGVIAYEMIYGR

Uniprot       SPFAEGTSARTFNNIMNFQRFLKFPDDPKVSSDFLDLIQSLLCGQKERLKFEGLCCHPFFSKIDWNNIRNSPPPFVPTLKSDDDTSNFDEPEKNSWVSSS
Splooce       SPFAEGTSARTFNNIMNFQRFLKFPDDPKVSSDFLDLIQSLLCGQKERLKFEGLCCHPFFSKIDWNNIRNSPPPFVPTLKSDDDTSNFDEPEKNSWVSSS

Uniprot       PCQLSPSGFSGEELPFVGFSYSKALGILGRSESVVSGLDSPAKTSSMEKKLLIKSKELQDSQDKCHKMEQEMTRLHRRVSEVEAVLSQKEVELKASETQR
Splooce       PCQLSPSGFSGEELPFVGFSYSKALGILGRSESVVSGLDSPAKTSSMEKKLLIKSKELQDSQDKCHKMEQEMTRLHRRVSEVEAVLSQKEVELKASETQR

Uniprot       SLLEQDLATYITECSSLKRSLEQARMEVSQEDDKALQLLHDIREQSRKLQEIKEQEYQAQVEEMRLMMNQLEEDLVSARRRSDLYESELRESRLAAEEFK
Splooce       SLLEQDLATYITECSSLKRSLEQ--------------------------------EYQAQVEEMRLMMNQLEEDLVSARRRSDLYESELRESRLAAEEFK

Uniprot       RKATECQHKLLKAKDQGKPEVGEYAKLEKINAEQQLKIQELQEKLEKAVKASTEATELLQNIRQAKERAERELEKLQNREDSSEGIRKKLVEAEELEEKH
Splooce       RKATECQHKLLKAKDQGKPEVGEYAKLEKINAEQQLKIQELQEKLEKAVKASTEATELLQNIRQAKERAERELEKLQNREDSSEGIRKKLVEAEELEEKH

Uniprot       REAQVSAQHLEVHLKQKEQHYEEKIKVLDNQIKKDLADKETLENMMQRHEEEAHEKGKILSEQKAMINAMDSKIRSLEQRIVELSEANKLAANSSLFTQR
Splooce       REAQVSAQHLEVHLKQKEQHYEEKIKVLDNQIKKDLADKETLENMMQRHEEEAHEKGKILSEQKAMINAMDSKIRSLEQRIVELSEANKLAANSSLFTQR

Uniprot       NMKAQEEMISELRQQKFYLETQAGKLEAQNRKLEEQLEKISHQDHSDKNRLLELETRLREVSLEHEEQKLELKRQLTELQLSLQERESQLTALQAARAAL
Splooce       NMKAQEEMISELRQQKFYLETQAGKLEAQNRKLEEQLEKISHQDHSDKNRLLELETRLREVSLEHEEQKLELKRQLTELQLSLQERESQLTALQAARAAL

Uniprot       ESQLRQAKTELEETTAEAEEEIQALTAHRDEIQRKFDALRNSCTVITDLEEQLNQLTEDNAELNNQNFYLSKQLDEASGANDEIVQLRSEVDHLRREITE
Splooce       ESQLRQAKTELEETTAEAEEEIQALTAHRDEIQRKFDALRNSCTVITDLEEQLNQLTEDNAELNNQNFYLSKQLDEASGANDEIVQLRSEVDHLRREITE

Uniprot       REMQLTSQKQTMEALKTTCTMLEEQVMDLEALNDELLEKERQWEAWRSVLGDEKSQFECRVRELQRMLDTEKQSRARADQRITESRQVVELAVKEHKAEI
Splooce       REMQLTSQKQTMEALKTTCTMLEEQVMDLEALNDELLEKERQWEAWRSVLGDEKSQFECRVRELQRMLDTEKQSRARADQRITESRQVVELAVKEHKAEI

Uniprot       LALQQALKEQKLKAESLSDKLNDLEKKHAMLEMNARSLQQKLETERELKQRLLEEQAKLQQQMDLQKNHIFRLTQGLQEALDRADLLKTERSDLEYQLEN
Splooce       LALQQALKEQKLKAESLSDKLNDLEKKHAMLEMNARSLQQKLETERELKQRLLEEQAKLQQQMDLQKNHIFRLTQGLQEALDRADLLKTERSDLEYQLEN

Uniprot       IQVLYSHEKVKMEGTISQQTKLIDFLQAKMDQPAKKKKGLFSRRKEDPALPTQVPLQYNELKLALEKEKARCAELEEALQKTRIELRSAREEAAHRKATD
Splooce       IQVLYSHEKVKMEGTISQQTKLIDFLQAKMDQPAKKKKGLFSRRKEDPALPTQVPLQYNELKLALEKEKARCAELEEALQKTRIELRSAREEAAHRKATD

Uniprot       HPHPSTPATARQQIAMSAIVRSPEHQPSAMSLLAPPSSRRKESSTPEEFSRRLKERMHHNIPHRFNVGLNMRATKCAVCLDTVHFGRQASKCLECQVMCH
Splooce       HPHPSTPATARQQIAMSAIVRSPEHQPSAMSLLAPPSSRRKESSTPEEFSRRLKERMHHNIPHRFNVGLNMRATKCAVCLDTVHFGRQASKCLECQVMCH

Uniprot       PKCSTCLPATCGLPAEYATHFTEAFCRDKMNSPGLQTKEPSSSLHLEGWMKVPRNNKRGQQGWDRKYIVLEGSKVLIYDNEAREAGQRPVEEFELCLPDG
Splooce       PKCSTCLPATCGLPAEYATHFTEAFCRDKMNSPGLQTKEPSSSLHLEGWMKVPRNNKRGQQGWDRKYIVLEGSKVLIYDNEAREAGQRPVEEFELCLPDG

Uniprot       DVSIHGAVGASELANTAKADVPYILKMESHPHTTCWPGRTLYLLAPSFPDKQRWVTALESVVAGGRVSREKAEADAKLLGNSLLKLEGDDRLDMNCTLPF
Splooce       DVSIHGAVGASELANTAKADVPYILKMESHPHTTCWPGRTLYLLAPSFPDKQRWVTALESVVAGGRVSREKAEADAKLLGNSLLKLEGDDRLDMNCTLPF

Uniprot       SDQVVLVGTEEGLYALNVLKNSLTHVPGIGAVFQIYIIKDLEKLLMIAGEERALCLVDVKKVKQSLAQSHLPAQPDISPNIFEAVKGCHLFGAGKIENGL
Splooce       SDQVVLVGTEEGLYALNVLKNSLTHVPGIGAVFQIYIIKDLEKLLMIAGEERALCLVDVKKVKQSLAQSHLPAQPDISPNIFEAVKGCHLFGAGKIENGL

Uniprot       CICAAMPSKVVILRYNENLSKYCIRKEIETSEPCSCIHFTNYSILIGTNKFYEIDMKQYTLEEFLDKNDHSLAPAVFAASSNSFPVSIVQVNSAGQREEY
Splooce       CICAAMPSKVVILRYNENLSKYCIRKEIETSEPCSCIHFTNYSILIGTNKFYEIDMKQYTLEEFLDKNDHSLAPAVFAASSNSFPVSIVQVNSAGQREEY

Uniprot       LLCFHEFGVFVDSYGRRSRTDDLKWSRLPLAFAYREPYLFVTHFNSLEVIEIQARSSAGTPARAYLDIPNPRYLGPAISSGAIYLASSYQDKLRVICCKG
Splooce       LLCFHEFGVFVDSYGRRSRTDDLKWSRLPLAFAYREPYLFVTHFNSLEVIEIQARSSAGTPARAYLDIPNPRYLGPAISSGAIYLASSYQDKLRVICCKG

Uniprot       NLVKESGTEHHRGPSTSRSSPNKRGPPTYNEHITKRVASSPAPPEGPSHPREPSTPHRYREGRTELRRDKSPGRPLEREKSPGRMLSTRRERSPGRLFED
Splooce       NLVKESGTEHHRGPSTSRSSPNKRGPPTYNEHITKRVASSPAPPEGPSHPREPSTPHRYREGRTELRRDKSPGRPLEREKSPGRMLSTRRERSPGRLFED

Uniprot       SSRGRLPAGAVRTPLSQVNKVWDQSSV
Splooce       SSRGRLPAGAVRTPLSQVNKVWDQSSV

----------------------------------------------------------------------------------------------------

O60218 (Uniprot)	versus
NM_020299#(-t:7_A6365737165200) (Splooce)

For more details about the Alternative Splicing Event -> Link to Splooce page

Peptides that support the ASE (Splooce-specific):
IGSDPFPYPEECDCHPQVCDTSTHC (MAXQUANT)

Alignment:
Uniprot       MATFVELSTKAKMPIVGLGTWKSPLGKVKEAVKVAIDAGYRHIDCAYVYQNEHEVGEAIQEKIQEKAVKREDLFIVSKLWPTFFERPLVRKAFEKTLKDL
Splooce       MATFVELSTKAKMPIVGLGTWKSPLGKVKEAVKVAIDAGYRHIDCAYVYQNEHEVGEAIQEKIQEKAVKREDLFIVSKLWPTFFERPLVRKAFEKTLKDL

Uniprot       KLSYLDVYLIHWPQGFKSGDDLFPKDDKGNAIGGKATFLDAWEAMEELVDEGLVKALGVSNFSHFQIEKLLNKPGLKYKPVTNQVECHPYLTQEKLIQYC
Splooce       KLSYLDVYLIHWPQGFKSGDDLFPKDDKGNAIGGKATFLDAWEAMEELVDEGLVKALGVSNFSHFQIEKLLNKPGLKYKPVTNQVECHPYLTQEKLIQYC

Uniprot       HSKGITVTAYSPLGSPDRPWAKPEDPSLLEDPKIKEIAAKHKKTAAQVLIRFHIQRNVIVIPKSVTPARIVENIQVFDFKLSDEEMATILSFNRNWRACN
Splooce       HSKGITVTAYSPLGSPDRPWAKPEDPSLLEDPKIKEIAAKHKKTAAQ--------EGSLELSGSLMSP----------FRIGSDPFPYPEECDCHPQVCD

Uniprot       VLQSSHLEDYPFNAEY
Splooce       T--STHC---------

----------------------------------------------------------------------------------------------------

O14787 (Uniprot)	versus
NM_013433#(-s-:19_T8249473338949) (Splooce)

For more details about the Alternative Splicing Event -> Link to Splooce page

Peptides that support the ASE (Splooce-specific):
GLLFHPEWVVKESGILVLGAIAEVLLPPWR (MAXQUANT)

Alignment:
Uniprot       MDWQPDEQGLQQVLQLLKDSQSPNTATQRIVQDKLKQLNQFPDFNNYLIFVLTRLKSEDEPTRSLSGLILKNNVKAHYQSFPPPVADFIKQECLNNIGDA
Splooce       MDWQPDEQGLQQVLQLLKDSQSPNTATQRIVQDKLKQLNQFPDFNNYLIFVLTRLKSEDEPTRSLSGLILKNNVKAHYQSFPPPVADFIKQECLNNIGDA

Uniprot       SSLIRATIGILITTIASKGELQMWPELLPQLCNLLNSEDYNTCEGAFGALQKICEDSSELLDSDALNRPLNIMIPKFLQFFKHCSPKIRSHAIACVNQFI
Splooce       SSLIRATIGILITTIASKGELQMWPELLPQLCNLLNSEDYNTCEGAFGALQKICEDSSELLDSDALNRPLNIMIPKFLQFFKHCSPKIRSHAIACVNQFI

Uniprot       MDRAQALMDNIDTFIEHLFALAVDDDPEVRKNVCRALVMLLEVRIDRLIPHMHSIIQYMLQRTQDHDENVALEACEFWLTLAEQPICKEVLASHLVQLIP
Splooce       MDRAQALMDNIDTFIEHLFALAVDDDPEVRKNVCRALVMLLEVRIDRLIPHMHSIIQYMLQRTQDHDENVALEACEFWLTLAEQPICKEVLASHLVQLIP

Uniprot       ILVNGMKYSEIDIILLKGDVEEDEAVPDSEQDIKPRFHKSRTVTLPHEAERPDGSEDAEDDDDDDALSDWNLRKCSAAALDVLANVFREELLPHLLPLLK
Splooce       ILVNGMKYSEIDIILLKGDVEEDEAVPDSEQDIKPRFHKSRTVTLPHEAERPDGSEDAEDDDDDDALSDWNLRKCSAAALDVLANVFREELLPHLLPLLK

Uniprot       GLLFHPEWVVKESGILVLGAIAEGCMQGMVPYLPELIPHLIQCLSDKKALVRSIACWTLSRYAHWVVSQPPDMHLKPLMTELLKRILDGNKRVQEAACSA
Splooce       GLLFHPEWVVKESGILVLGAIAEVLLP---PWR-----------------KRPARSWCPTSATSWTPLSLP----------------LGNTSTRTCSS--

Uniprot       FATLEEEACTELVPYLSYILDTLVFAFGKYQHKNLLILYDAIGTLADSVGHHLNQPEYIQKLMPPLIQKWNELKDEDKDLFPLLECLSSVATALQSGFLP
Splooce       -------SMTPLAPWPTL----------------------------------------------------------------------------------

Uniprot       YCEPVYQRCVTLVQKTLAQAMMYTQHPEQYEAPDKDFMIVALDLLSGLAEGLGGHVEQLVARSNIMTLLFQCMQDSMPEVRQSSFALLGDLTKACFIHVK
Splooce       ----------------------------------------------------------------------------------------------------

Uniprot       PCIAEFMPILGTNLNPEFISVCNNATWAIGEICMQMGAEMQPYVQMVLNNLVEIINRPNTPKTLLENTAITIGRLGYVCPQEVAPMLQQFIRPWCTSLRN
Splooce       ----------------------------------------------------------------------------------------------------

Uniprot       IRDNEEKDSAFRGICMMIGVNPGGVVQDFIFFCDAVASWVSPKDDLRDMFYKILHGFKDQVGEDNWQQFSEQFPPLLKERLAAFYGV
Splooce       ---------------------------------------------------------------------------------------

----------------------------------------------------------------------------------------------------

Q14152 (Uniprot)	versus
NM_003750#(-s-s-:10_E9685052885379) (Splooce)

For more details about the Alternative Splicing Event -> Link to Splooce page

Peptides that support the ASE (Splooce-specific):
MTGALGETWMMIAFQDVLMMIGFPDGVMTQDLVLGDH (MAXQUANT)

Alignment:
Uniprot       MPAYFQRPENALKRANEFLEVGKKQPALDVLYDVMKSKKHRTWQKIHEPIMLKYLELCVDLRKSHLAKEGLYQYKNICQQVNIKSLEDVVRAYLKMAEEK
Splooce       MPAYFQRPENALKRANEFLEVGKKQPALDVLYDVMKSKKHRTWQKIHEPIMLKYLELCVDLRKSHLAKEGLYQYKNICQQVNIKSLEDVVRAYLKMAEEK

Uniprot       TEAAKEESQQMVLDIEDLDNIQTPESVLLSAVSGEDTQDRTDRLLLTPWVKFLWESYRQCLDLLRNNSRVERLYHDIAQQAFKFCLQYTRKAEFRKLCDN
Splooce       TEAAKEESQQMVLDIEDLDNIQTPESVLLSAVSGEDTQDRTDRLLLTPWVKFLWESYRQCLDLLRNNSRVERLYHDIAQQAFKFCLQYTRKAEFRKLCDN

Uniprot       LRMHLSQIQRHHNQSTAINLNNPESQSMHLETRLVQLDSAISMELWQEAFKAVEDIHGLFSLSKKPPKPQLMANYYNKVSTVFWKSGNALFHASTLHRLY
Splooce       LRMHLSQIQRHHNQSTAINLNNPESQSMHLETRLVQLDSAISMELWQEAFKAVEDIHGLFSLSKKPPKPQLMANYYNKVSTVFWKSGNALFHASTLHRLY

Uniprot       HLSREMRKNLTQDEMQRMSTRVLLATLSIPITPERTDIARLLDMDGIIVEKQRRLATLLGLQAPPTRIGLINDMVRFNVLQYVVPEVKDLYNWLEVEFNP
Splooce       HLSREMRKNLTQDEMQRMSTRVLLATLSIPITPERTDIARLLDMDGIIVEKQRRLATLLGLQAPPTRIGLINDMVRFNVLQYVVPEVKDLYNWLEVEFNP

Uniprot       LKLCERVTKVLNWVREQPEKEPELQQYVPQLQNNTILRLLQQVSQIYQSIEFSRLTSLVPFVDAFQLERAIVDAARHCDLQVRIDHTSRTLSFGSDLNYA
Splooce       LKLCERVTKVLNWVREQPEKEPELQQYVPQLQNNTILRLLQQVSQIYQSIEFSRLTSLVPFVDAFQLERAIVDAARHCDLQVRIDHTSRTLSFGSDLNYA

Uniprot       TREDAPIGPHLQSMPSEQIRNQLTAMSSVLAKALEVIKPAHILQEKEEQHQLAVTAYLKNSRKEHQRILARRQTIEERKERLESLNIQREKEELEQREAE
Splooce       TREDAPIGPHLQSMPSEQIRNQLTAMSSVLAKALEVIKPAHILQEKEEQHQLAVTAYLKNSRKEHQRILARRQTIEERKERLESLNIQREKEELEQREAE

Uniprot       LQKVRKAEEERLRQEAKEREKERILQEHEQIKKKTVRERLEQIKKTELGAKAFKDIDIEDLEELDPDFIMAKQVEQLEKEKKELQERLKNQEKKIDYFER
Splooce       LQKVRKAEEERLRQEAKEREKERILQEHEQIKKKTVRERLEQIKKTELGAKAFKDIDIEDLEELDPDFIMAKQVEQLEKEKKELQERLKNQEKKIDYFER

Uniprot       AKRLEEIPLIKSAYEEQRIKDMDLWEQQEEERITTMQLEREKALEHKNRMSRMLEDRDLFVMRLKAARQSVYEEKLKQFEERLAEERHNRLEERKRQRKE
Splooce       AKRLEEIPLIKSAYEEQRIKDMDLWEQQEEERITTMQLEREKALEHKNRMSRMLEDRDLFVMRLKAARQSVYEEKLKQFEERLAEERHNRLEERKRQRKE

Uniprot       ERRITYYREKEEEEQRRAEEQMLKEREERERAERAKREEELREYQERVKKLEEVERKKRQRELEIEERERRREEERRLGDSSLSRKDSRWGDRDSEGTWR
Splooce       ERRITYYREKEEEEQRRAEEQMLKGSGDVEKGE------------MRTGLIEEMKSGP--------------------GVWGMMKIESPLLDQTMIG---

Uniprot       KGPEADSEWRRGPPEKEWRRGEGRDEDRSHRRDEERPRRLGDDEDREPSLRPDDDRVPRRGMDDDRGPRRGPEEDRFSRRGADDDRPSWRNTDDDRPPRR
Splooce       ------------FPGVAWMMTEALD---------VVLRKIG-------SLVVGQTMTGLPGVTQ-------------MMTGLPDELP--MKTGETGVMRM

Uniprot       IADEDRGNWRHADDDRPPRRGLDEDRGSWRTADEDRGPRRGMDDDRGPRRGGADDERSSWRNADDDRGPRRGLDDDRGPRRGMDDDRGPRRGMDDDRGPR
Splooce       MTDHLDEDWMRTEEAG---------EQLMRTEDQDVG--WMMTGGRGEEALMMSDHPGVMLMMTGVPGEGWMMIGVPGEAWMMTGVPGEAWMMTGVPGEA

Uniprot       RGMDDDRGPRRGLDDDRGPWRNADDDRIPRRGAEDDRGPWRNMDDDRLSRRADDDRFPRRGDDSRPGPWRPLVKPGGWREKEKAREESWGPPRESRPSEE
Splooce       WMMTGVPGEGWMMIEDLG-------------------GTPMMTEFPGVVQRMTG----ALGETWMMIAFQDVLMMIGFPDGVMTQDLVLGDH--------

Uniprot       REWDREKERDRDNQDREENDKDPERERDRERDVDREDRFRRPRDEGGWRRGPAEESSSWRDSSRRDDRDRDDRRRERDDRRDLRERRDLRDDRDRRGPPL
Splooce       ----------------------------------------------------------------------------------------------------

Uniprot       RSEREEVSSWRRADDRKDDRVEERDPPRRVPPPALSRDRERDRDREREGEKEKASWRAEKDRESLRRTKNETDEDGWTTVRR
Splooce       ----------------------------------------------------------------------------------

----------------------------------------------------------------------------------------------------

Q93084 (Uniprot)	versus
NM_174958#(r:17_A4827807807107) (Splooce)

For more details about the Alternative Splicing Event -> Link to Splooce page

Peptides that support the ASE (Splooce-specific):
YLISSNVGEVVW (MAXQUANT)

Alignment:
Uniprot       MEAAHLLPAADVLRHFSVTAEGGLSPAQVTGARERYGPNELPSEEGKSLWELVLEQFEDLLVRILLLAALVSFVLAWFEEGEETTTAFVEPLVIMLILVA
Splooce       MEAAHLLPAADVLRHFSVTAEGGLSPAQVTGARERYGPNELPSEEGKSLWELVLEQFEDLLVRILLLAALVSFVLAWFEEGEETTTAFVEPLVIMLILVA

Uniprot       NAIVGVWQERNAESAIEALKEYEPEMGKVIRSDRKGVQRIRARDIVPGDIVEVAVGDKVPADLRLIEIKSTTLRVDQSILTGESVSVTKHTEAIPDPRAV
Splooce       NAIVGVWQERNAESAIEALKEYEPEMGKVIRSDRKGVQRIRARDIVPGDIVEVAVGDKVPADLRLIEIKSTTLRVDQSILTGESVSVTKHTEAIPDPRAV

Uniprot       NQDKKNMLFSGTNITSGKAVGVAVATGLHTELGKIRSQMAAVEPERTPLQRKLDEFGRQLSHAISVICVAVWVINIGHFADPAHGGSWLRGAVYYFKIAV
Splooce       NQDKKNMLFSGTNITSGKAVGVAVATGLHTELGKIRSQMAAVEPERTPLQRKLDEFGRQLSHAISVICVAVWVINIGHFADPAHGGSWLRGAVYYFKIAV

Uniprot       ALAVAAIPEGLPAVITTCLALGTRRMARKNAIVRSLPSVETLGCTSVICSDKTGTLTTNQMSVCRMFVVAEADAGSCLLHEFTISGTTYTPEGEVRQGDQ
Splooce       ALAVAAIPEGLPAVITTCLALGTRRMARKNAIVRSLPSVETLGCTSVICSDKTGTLTTNQMSVCRMFVVAEADAGSCLLHEFTISGTTYTPEGEVRQGDQ

Uniprot       PVRCGQFDGLVELATICALCNDSALDYNEAKGVYEKVGEATETALTCLVEKMNVFDTDLQALSRVERAGACNTVIKQLMRKEFTLEFSRDRKSMSVYCTP
Splooce       PVRCGQFDGLVELATICALCNDSALDYNEAKGVYEKVGEATETALTCLVEKMNVFDTDLQALSRVERAGACNTVIKQLMRKEFTLEFSRDRKSMSVYCTP

Uniprot       TRPHPTGQGSKMFVKGAPESVIERCSSVRVGSRTAPLTPTSREQILAKIRDWGSGSDTLRCLALATRDAPPRKEDMELDDCSKFVQYETDLTFVGCVGML
Splooce       TRPHPTGQGSKMFVKGAPESVIERCSSVRVGSRTAPLTPTSREQILAKIRDWGSGSDTLRCLALATRDAPPRKEDMELDDCSKFVQYETDLTFVGCVGML

Uniprot       DPPRPEVAACITRCYQAGIRVVMITGDNKGTAVAICRRLGIFGDTEDVAGKAYTGREFDDLSPEQQRQACRTARCFARVEPAHKSRIVENLQSFNEITAM
Splooce       DPPRPEVAACITRCYQAGIRVVMITGDNKGTAVAICRRLGIFGDTEDVAGKAYTGREFDDLSPEQQRQACRTARCFARVEPAHKSRIVENLQSFNEITAM

Uniprot       TGDGVNDAPALKKAEIGIAMGSGTAVAKSAAEMVLSDDNFASIVAAVEEGRAIYSNMKQFIRYLISSNVGEVVCIFLTAILGLPEALIPVQLLWVNLVTD
Splooce       TGDGVNDAPALKKAEIGIAMGSGTAVAKSAAEMVLSDDNFASIVAAVEEGRAIYSNMKQFIRYLISSNVGEVVW--------------------------

Uniprot       GLPATALGFNPPDLDIMEKLPRSPREALISGWLFFRYLAIGVYVGLATVAAATWWFVYDAEGPHINFYQLRNFLKCSEDNPLFAGIDCEVFESRFPTTMA
Splooce       ----------------------------------------------------------------------------------------------------

Uniprot       LSVLVTIEMCNALNSVSENQSLLRMPPWMNPWLLVAVAMSMALHFLILLVPPLPLIFQVTPLSGRQWVVVLQISLPVILLDEALKYLSRNHMHACLYPGL
Splooce       ----------------------------------------------------------------------------------------------------

Uniprot       LRTVSQAWSRQPLTTSWTPDHTGLASLKK
Splooce       -----------------------------

----------------------------------------------------------------------------------------------------

Q13505 (Uniprot)	versus
NM_002455#(-s-s-:1_M3915287073341) (Splooce)

For more details about the Alternative Splicing Event -> Link to Splooce page

Peptides that support the ASE (Splooce-specific):
LLPVLPCLLGRLR (MAXQUANT)

Alignment:
Uniprot       MLLGGPPRSPRSGTSPKGPWSSTGHVQFGKSPQTWPRRTRPRSPEPAAPSGVRGSTWTRRRDTPRRAGPTALSRYVGHLWMGRRPPSPEARGPVPRSSAA
Splooce       MLLGGPPRSPRSGTSPKGPWSSTGHVQFGKSPQTWPRRTRPRSPEPAAPSGVRGSTWTRRRDTPRRAGPTALSRYVGHLWMGRRPPSPEARGPVPRSSAA

Uniprot       SRARRSLASPGISPGPLTATIGGAVAGGGPRQGRAEAHKEVFPGQRVGKMAAPMELFCWSGGWGLPSVDLDSLAVLTYARFTGAPLKVHKISNPWQSPSG
Splooce       SRARRSLASPGISPGPLTATIGGAVAGGGPRQGRAEAHKEVFPGQRVGKMAAPMELFCWSGGWGLPSVDLDSLAVLTYARFTGAPLKVHKISNPWQSPSG

Uniprot       TLPALRTSHGEVISVPHKIITHLRKEKYNADYDLSARQGADTLAFMSLLEEKLLPVLVHTFWIDTKNYVEVTRKWYAEAMPFPLNFFLPGRMQRQYMERL
Splooce       TLPALRTSHGEVISVPHKIITHLRKEKYNADYDLSARQGADTLAFMSLLEEKLLPVLP----------------------------CLLGRLR------L

Uniprot       QLLTGEHRPEDEEELEKELYREARECLTLLSQRLGSQKFFFGDAPASLDAFVFSYLALLLQAKLPSGKLQVHLRGLHNLCAYCTHILSLYFPWDGAEVPP
Splooce       QLLG----------------------------------------PAAAG------KAAQWEAAGPP--------------AWAAQPLCLLYPHSQSLLP-

Uniprot       QRQTPAGPETEEEPYRRRNQILSVLAGLAAMVGYALLSGIVSIQRATPARAPGTRTLGMAEEDEEE
Splooce       -------------------------------LGWS-------------------------------

----------------------------------------------------------------------------------------------------

K7ESE3 (Uniprot)	versus
NM_005053#(-tF-:19_R2012497008569) (Splooce)

For more details about the Alternative Splicing Event -> Link to Splooce page

Peptides that support the ASE (Splooce-specific):
AVEYLLTGIPGSPEPEHGSVQESQVSEQPATEAGENPLEFLR (MAXQUANT)

Alignment:
Uniprot       MAVTITLKTLQQQTFKIRMEPDETVKVLKEKIEAEKGRDAFPVAGQKLIYAGKILSDDVPIRDYRIDEKNFVVVMVTKTKAGQGTSAPPEASPTAAPESS
Splooce       MAVTITLKTLQQQTFKIRMEPDETVKVLKEKIEAEKGRDAFPVAGQKLIYAGKILSDDVPIRDYRIDEKNFVVVMVTKTKAGQGTSAPPEASPTAAPESS

Uniprot       TSFPPAPTSGMSHPPPAAREDKSPSEESAPTTSPESVSGSVPSSGSSGREEDAASTLVTGSEYETMLTEIMSMGYERERVVAALRASYNNPHRAVEYLLT
Splooce       TSFPPAPTSGMSHPPPAAREDKSPSEESAPTTSPESVSGSVPSSGSSGREEDAASTLVTGSEYETMLTEIMSMGYERERVVAALRASYNNPHRAVEYLLT

Uniprot       GIPGSPEPEHGSVQESQVSEQPATEAAGENPLEFLRDQPQFQNMRQVIQQNPALLPALLQQLGQENPQLLQQISRHQEQFIQMLNEPPGELADISDVEGE
Splooce       GIPGSPEPEHGSVQESQVSEQPATEAG-ENPLEFLRDQPQFQNMRQVIQQNPALLPALLQQLGQENPQLLQVKS-------------AGTRSSSSRC---

Uniprot       VGAIGEEAPQMNYIQVTPQEKEAIERLKALGFPESLVIQAYFACEKNENLAANFLLSQNFDDE
Splooce       ---------------------------------------------------------------

----------------------------------------------------------------------------------------------------

P52789 (Uniprot)	versus
NM_000189#(-s-s-s-s-s-s-:2_H4797741904914) (Splooce)

For more details about the Alternative Splicing Event -> Link to Splooce page

Peptides that support the ASE (Splooce-specific):
DISDIEGHGQQCLLHGGDAQR (MAXQUANT)

Alignment:
Uniprot       MIASHLLAYFFTELNHDQVQKVDQYLYHMRLSDETLLEISKRFRKEMEKGLGATTHPTAAVKMLPTFVRSTPDGTEHGEFLALDLGGTNFRVLWVKVTDN
Splooce       MIASHLLAYFFTELNHDQVQKVDQYLYHMRLSDETLLEISKRFRKEMEKGLGATTHPTAAVKMLPTFVRSTPDGTEHGEFLALDLGGTNFRVLWVKVTDN

Uniprot       GLQKVEMENQIYAIPEDIMRGSGTQLFDHIAECLANFMDKLQIKDKKLPLGFTFSFPCHQTKLDESFLVSWTKGFKSSGVEGRDVVALIRKAIQRRGDFD
Splooce       GLQKVEMENQIYAIPEDIMRGSGTQLFDHIAECLANFMDKLQIKDKKLPLGFTFSFPCHQTKLDESFLVSWTKGFKSSGVEGRDVVALIRKAIQRRGDFD

Uniprot       IDIVAVVNDTVGTMMTCGYDDHNCEIGLIVGTGSNACYMEEMRHIDMVEGDEGRMCINMEWGAFGDDGSLNDIRTEFDQEIDMGSLNPGKQLFEKMISGM
Splooce       IDIVAVVNDTVGTMMTCGYDDHNCEIGLIVGTGSNACYMEEMRHIDMVEGDEGRMCINMEWGAFGDDGSLNDIRTEFDQEIDMGSLNPGKQLFEKMISGM

Uniprot       YMGELVRLILVKMAKEELLFGGKLSPELLNTGRFETKDISDIEGEKDGIRKAREVLMRLGLDPTQEDCVATHRICQIVSTRSASLCAATLAAVLQRIKEN
Splooce       YMGELVRLILVKMAKEELLFGGKLSPELLNTGRFETKDISDIEG------HGQQCLL-------------------------------------------

Uniprot       KGEERLRSTIGVDGSVYKKHPHFAKRLHKTVRRLVPGCDVRFLRSEDGSGKGAAMVTAVAYRLADQHRARQKTLEHLQLSHDQLLEVKRRMKVEMERGLS
Splooce       HGGDAQRGTGGRR----------------------RGADV----CEHGMG-----------GLRGQWMPR------------------------------

Uniprot       KETHASAPVKMLPTYVCATPDGTEKGDFLALDLGGTNFRVLLVRVRNGKWGGVEMHNKIYAIPQEVMHGTGDELFDHIVQCIADFLEYMGMKGVSLPLGF
Splooce       ----------------------------------------------------------------------------------------------------

Uniprot       TFSFPCQQNSLDESILLKWTKGFKASGCEGEDVVTLLKEAIHRREEFDLDVVAVVNDTVGTMMTCGFEDPHCEVGLIVGTGSNACYMEEMRNVELVEGEE
Splooce       ----------------------------------------------------------------------------------------------------

Uniprot       GRMCVNMEWGAFGDNGCLDDFRTEFDVAVDELSLNPGKQRFEKMISGMYLGEIVRNILIDFTKRGLLFRGRISERLKTRGIFETKFLSQIESDCLALLQV
Splooce       ----------------------------------------------------------------------------------------------------

Uniprot       RAILQHLGLESTCDDSIIVKEVCTVVARRAAQLCGAGMAAVVDRIRENRGLDALKVTVGVDGTLYKLHPHFAKVMHETVKDLAPKCDVSFLQSEDGSGKG
Splooce       ----------------------------------------------------------------------------------------------------

Uniprot       AALITAVACRIREAGQR
Splooce       -----------------

----------------------------------------------------------------------------------------------------

P53621 (Uniprot)	versus
NM_004371#(r:1_C2313924906730) (Splooce)

For more details about the Alternative Splicing Event -> Link to Splooce page

Peptides that support the ASE (Splooce-specific):
KLGEALK (PEAKS)

Alignment:
Uniprot       MLTKFETKSARVKGLSFHPKRPWILTSLHNGVIQLWDYRMCTLIDKFDEHDGPVRGIDFHKQQPLFVSGGDDYKIKVWNYKLRRCLFTLLGHLDYIRTTF
Splooce       MLTKFETKSARVKGLSFHPKRPWILTSLHNGVIQLWDYRMCTLIDKFDEHDGPVRGIDFHKQQPLFVSGGDDYKIKVWNYKLRRCLFTLLGHLDYIRTTF

Uniprot       FHHEYPWILSASDDQTIRVWNWQSRTCVCVLTGHNHYVMCAQFHPTEDLVVSASLDQTVRVWDISGLRKKNLSPGAVESDVRGITGVDLFGTTDAVVKHV
Splooce       FHHEYPWILSASDDQTIRVWNWQSRTCVCVLTGHNHYVMCAQFHPTEDLVVSASLDQTVRVWDISGLRKKNLSPGAVESDVRGITGVDLFGTTDAVVKHV

Uniprot       LEGHDRGVNWAAFHPTMPLIVSGADDRQVKIWRMNESKAWEVDTCRGHYNNVSCAVFHPRQELILSNSEDKSIRVWDMSKRTGVQTFRRDHDRFWVLAAH
Splooce       LEGHDRGVNWAAFHPTMPLIVSGADDRQVKIWRMNESKAWEVDTCRGHYNNVSCAVFHPRQELILSNSEDKSIRVWDMSKRTGVQTFRRDHDRFWVLAAH

Uniprot       PNLNLFAAGHDGGMIVFKLERERPAYAVHGNMLHYVKDRFLRQLDFNSSKDVAVMQLRSGSKFPVFNMSYNPAENAVLLCTRASNLENSTYDLYTIPKDA
Splooce       PNLNLFAAGHDGGMIVFKLERERPAYAVHGNMLHYVKDRFLRQLDFNSSKDVAVMQLRSGSKFPVFNMSYNPAENAVLLCTRASNLENSTYDLYTIPKDA

Uniprot       DSQNPDAPEGKRSSGLTAVWVARNRFAVLDRMHSLLIKNLKNEITKKVQVPNCDEIFYAGTGNLLLRDADSITLFDVQQKRTLASVKISKVKYVIWSADM
Splooce       DSQNPDAPEGKRSSGLTAVWVARNRFAVLDRMHSLLIKNLKNEITKKVQVPNCDEIFYAGTGNLLLRDADSITLFDVQQKRTLASVKISKVKYVIWSADM

Uniprot       SHVALLAKHAIVICNRKLDALCNIHENIRVKSGAWDESGVFIYTTSNHIKYAVTTGDHGIIRTLDLPIYVTRVKGNNVYCLDRECRPRVLTIDPTEFKFK
Splooce       SHVALLAKHAIVICNRKLDALCNIHENIRVKSGAWDESGVFIYTTSNHIKYAVTTGDHGIIRTLDLPIYVTRVKGNNVYCLDRECRPRVLTIDPTEFKFK

Uniprot       LALINRKYDEVLHMVRNAKLVGQSIIAYLQKKGYPEVALHFVKDEKTRFSLALECGNIEIALEAAKALDDKNCWEKLGEVALLQGNHQIVEMCYQRTKNF
Splooce       LALINRKYDEVLHMVRNAKLVGQSIIAYLQKKGYPEVALHFVKDEKTRFSLALECGNIEIALEAAKALDDKNCWEKLGEVALLQGNHQIVEMCYQRTKNF

Uniprot       DKLSFLYLITGNLEKLRKMMKIAEIRKDMSGHYQNALYLGDVSERVRILKNCGQKSLAYLTAATHGLDEEAESLKETFDPEKETIPDIDPNAKLLQPPAP
Splooce       DKLSFLYLITGNLEKLRKMMKIGEALKLRMGR-------GVLGGRRKVN---------------------------------------------------

Uniprot       IMPLDTNWPLLTVSKGFFEGTIASKGKGGALAADIDIDTVGTEGWGEDAELQLDEDGFVEATEGLGDDALGKGQEEGGGWDVEEDLELPPELDISPGAAG
Splooce       ----------------------------------------------------------------------------------------------------

Uniprot       GAEDGFFVPPTKGTSPTQIWCNNSQLPVDHILAGSFETAMRLLHDQVGVIQFGPYKQLFLQTYARGRTTYQALPCLPSMYGYPNRNWKDAGLKNGVPAVG
Splooce       ----------------------------------------------------------------------------------------------------

Uniprot       LKLNDLIQRLQLCYQLTTVGKFEEAVEKFRSILLSVPLLVVDNKQEIAEAQQLITICREYIVGLSVETERKKLPKETLEQQKRICEMAAYFTHSNLQPVH
Splooce       ----------------------------------------------------------------------------------------------------

Uniprot       MILVLRTALNLFFKLKNFKTAATFARRLLELGPKPEVAQQTRKILSACEKNPTDAYQLNYDMHNPFDICAASYRPIYRGKPVEKCPLSGACYSPEFKGQI
Splooce       ----------------------------------------------------------------------------------------------------

Uniprot       CRVTTVTEIGKDVIGLRISPLQFR
Splooce       ------------------------

----------------------------------------------------------------------------------------------------

P35221 (Uniprot)	versus
NM_001903#(-s-s-s-s-:5_C2627541305673) (Splooce)

For more details about the Alternative Splicing Event -> Link to Splooce page

Peptides that support the ASE (Splooce-specific):
LLVQLKVLRK (MAXQUANT)

Alignment:
Uniprot       MTAVHAGNINFKWDPKSLEIRTLAVERLLEPLVTQVTTLVNTNSKGPSNKKRGRSKKAHVLAASVEQATENFLEKGDKIAKESQFLKEELVAAVEDVRKQ
Splooce       MTAVHAGNINFKWDPKSLEIRTLAVERLLEPLVTQVTTLVNTNSKGPSNKKRGRSKKAHVLAASVEQATENFLEKGDKIAKESQFLKEELVAAVEDVRKQ

Uniprot       GDLMKAAAGEFADDPCSSVKRGNMVRAARALLSAVTRLLILADMADVYKLLVQLKVVEDGILKLRNAGNEQDLGIQYKALKPEVDKLNIMAAKRQQELKD
Splooce       GDLMKAAAGEFADDPCSSVKRGNMVRAARALLSAVTRLLILADMADVYKLLVQLKV--------------------------------------------

Uniprot       VGHRDQMAAARGILQKNVPILYTASQACLQHPDVAAYKANRDLIYKQLQQAVTGISNAAQATASDDASQHQGGGGGELAYALNNFDKQIIVDPLSFSEER
Splooce       ----------------------------------------------------------------------------------------------------

Uniprot       FRPSLEERLESIISGAALMADSSCTRDDRRERIVAECNAVRQALQDLLSEYMGNAGRKERSDALNSAIDKMTKKTRDLRRQLRKAVMDHVSDSFLETNVP
Splooce       ---------------------------------------------------------------------------------LRKAVMDHVSDSFLETNVP

Uniprot       LLVLIEAAKNGNEKEVKEYAQVFREHANKLIEVANLACSISNNEEGVKLVRMSASQLEALCPQVINAALALAAKPQSKLAQENMDLFKEQWEKQVRVLTD
Splooce       LLVLIEAAKNGNEKEVKEYAQVFREHANKLIEVANLACSISNNEEGVKLVRMSASQLEALCPQVINAALALAAKPQSKLAQENMDLFKEQWEKQVRVLTD

Uniprot       AVDDITSIDDFLAVSENHILEDVNKCVIALQEKDVDGLDRTAGAIRGRAARVIHVVTSEMDNYEPGVYTEKVLEATKLLSNTVMPRFTEQVEAAVEALSS
Splooce       AVDDITSIDDFLAVSENHILEDVNKCVIALQEKDVDGLDRTAGAIRGRAARVIHVVTSEMDNYEPGVYTEKVLEATKLLSNTVMPRFTEQVEAAVEALSS

Uniprot       DPAQPMDENEFIDASRLVYDGIRDIRKAVLMIRTPEELDDSDFETEDFDVRSRTSVQTEDDQLIAGQSARAIMAQLPQEQKAKIAEQVASFQEEKSKLDA
Splooce       DPAQPMDENEFIDASRLVYDGIRDIRKAVLMIRTPEELDDSDFETEDFDVRSRTSVQTEDDQLIAGQSARAIMAQLPQEQKAKIAEQVASFQEEKSKLDA

Uniprot       EVSKWDDSGNDIIVLAKQMCMIMMEMTDFTRGKGPLKNTSDVISAAKKIAEAGSRMDKLGRTIADHCPDSACKQDLLAYLQRIALYCHQLNICSKVKAEV
Splooce       EVSKWDDSGNDIIVLAKQMCMIMMEMTDFTRGKGPLKNTSDVISAAKKIAEAGSRMDKLGRTIADHCPDSACKQDLLAYLQRIALYCHQLNICSKVKAEV

Uniprot       QNLGGELVVSGVDSAMSLIQAAKNLMNAVVQTVKASYVASTKYQKSQGMASLNLPAVSWKMKAPEKKPLVKREKQDETQTKIKRASQKKHVNPVQALSEF
Splooce       QNLGGELVVSGVDSAMSLIQAAKNLMNAVVQTVKASYVASTKYQKSQGMASLNLPAVSWKMKAPEKKPLVKREKQDETQTKIKRASQKKHVNPVQALSEF

Uniprot       KAMDSI
Splooce       KAMDSI

----------------------------------------------------------------------------------------------------

P42167 (Uniprot)	versus
NM_001032283#(-s-:12_T1262619394867) (Splooce)

For more details about the Alternative Splicing Event -> Link to Splooce page

Peptides that support the ASE (Splooce-specific):
SYSQAGITETEWTSGSSK (MAXQUANT)

Alignment:
Uniprot       MPEFLEDPSVLTKDKLKSELVANNVTLPAGEQRKDVYVQLYLQHLTARNRPPLPAGTNSKGPPDFSSDEEREPTPVLGSGAAAAGRSRAAVGRKATKKTD
Splooce       ----------------------------------------------------------------------------------------------------

Uniprot       KPRQEDKDDLDVTELTNEDLLDQLVKYGVNPGPIVGTTRKLYEKKLLKLREQGTESRSSTPLPTISSSAENTRQNGSNDSDRYSDNEEDSKIELKLEKRE
Splooce       ----------------------------------------------------------------------------------------------------

Uniprot       PLKGRAKTPVTLKQRRVEHNQSYSQAGITETEWTSGSSKGGPLQALTRESTRGSRRTPRKRVETSEHFRIDGPVISESTPIAETIMASSNESLVVNRVTG
Splooce       -------MEVMILTDTVTMKKSYSQAGITETEWTSGSSKGGPLQALTRESTRGSRRTPRKRVETSEHFRIDGPVISESTPIAETIMASSNESLVVNRVTG

Uniprot       NFKHASPILPITEFSDIPRRAPKKPLTRAEVGEKTEERRVERDILKEMFPYEASTPTGISASCRRPIKGAAGRPLELSDFRMEESFSSKYVPKYVPLADV
Splooce       NFKHASPILPITEFSDIPRRAPKKPLTRAEVGEKTEERRVERDILKEMFPYEASTPTGISASCRRPIKGAAGRPLELSDFRMEESFSSKYVPKYVPLADV

Uniprot       KSEKTKKGRSIPVWIKILLFVVVAVFLFLVYQAMETNQVNPFSNFLHVDPRKSN
Splooce       KSEKTKKGRSIPVWIKILLFVVVAVFLFLVYQAMETNQVNPFSNFLHVDPRKSN

----------------------------------------------------------------------------------------------------

Q96S82 (Uniprot)	versus
NM_201265#(-s-s-:15_U6195782861165) (Splooce)

For more details about the Alternative Splicing Event -> Link to Splooce page

Peptides that support the ASE (Splooce-specific):
PYSMPFR (MAXQUANT)

Alignment:
Uniprot       MSLSDWHLAVKLADQPLTPKSILRLPETELGEYSLGGYSISFLKQLIAGKLQESVPDPELIDLIYCGRKLKDDQTLDFYGIQPGSTVHVLRKSWPEPDQK
Splooce       MSLSDWHLAVKLADQPLTPKSILRLPETELGEYSLGGYSISFLKQLIAGKLQESVPDPELIDLIYCGRKLKDDQTLDFYGIQPGSTVHVLRKSWPEPDQK

Uniprot       PEPVDKVAAMREFRVLHTALHSSSSYREAVFKMLSNKESLDQIIVATPGLSSDPIALGVLQDKDLFSVFADPNMLDTLVPAHPALVNAIVLVLHSVAGSA
Splooce       PEPVDKVAAMREFRVLHTALHSSSSYREAVFKMLSNKESLDQIIVATPGLSSDPIALGVLQDKDLFSVFADPNMLDTTPGPHPLAV-------------L

Uniprot       PMPGTDSSSRSMPSSSYRDMPGGFLFEGLSDDEDDFHPNTRSTPSSSTPSSRPASLGYSGAAGPRPITQSELATALALASTPESSSHTPTPGTQGHSSGT
Splooce       PAP-------AQPPWGTVELLG----PGPS-------PRVSWPPPWPWPALRRAALTHR---------------LLAPRVIPQGPHQCPLVSSQGRPS--

Uniprot       SPMSSGVQSGTPITNDLFSQALQHALQASGQPSLQSQWQPQLQQLRDMGIQDDELSLRALQATGGDIQAALELIFAGGAP
Splooce       -PMIS---SAKPYS-MPFRPLGSPAFRASGSPSCSSYVT---WASRTMS-------------------------------

----------------------------------------------------------------------------------------------------

UNIPROT? (Uniprot)	versus
NM_203457#(-t:1_P2892656478406) (Splooce)

For more details about the Alternative Splicing Event -> Link to Splooce page

Peptides that support the ASE (Splooce-specific):
AETQEIGNK (MAXQUANT)

Alignment:
Uniprot       -------
Splooce       MNESELFGRTIRVNLAKPMRIKEGSSRPVWSDDDWLKKFSGKTLEENKEEEGSEPPKAETQE-------------------IGNKPAGRIQMLLRSDVVP

Uniprot       -------
Splooce       MTAENFRCLCTHEKGFGFKGSSFHRIIPQFMCQGGDFTNHNGTGGKSIYGKKFDDENFILKHTGPGLLSMANSGPNTNGSQFFLTCDKTDWLDGKHVVFG

Uniprot       -------
Splooce       EVTEGLDVLRQIEAQGSKDGKPKQKVIIADCGEYV

----------------------------------------------------------------------------------------------------

P63261 (Uniprot)	versus
NM_001614#(f-:17_A2552063006919) (Splooce)

For more details about the Alternative Splicing Event -> Link to Splooce page

Peptides that support the ASE (Splooce-specific):
GIIAPPER (MAXQUANT)
GIIAPPERK (MAXQUANT)

Alignment:
Uniprot       MEEEIAALVIDNGSGMCKAGFAGDDAPRAVFPSIVGRPRHQGVMVGMGQKDSYVGDEAQSKRGILTLKYPIEHGIVTNWDDMEKIWHHTFYNELRVAPEE
Splooce       MEEEIAALVIDNGSGMCKAGFAGDDAPRAVFPSIVGRPRHQGVMVGMGQKDSYVGDEAQSKRGILTLKYPIEHGIVTNWDDMEKIWHHTFYNELRVAPEE

Uniprot       HPVLLTEAPLNPKANREKMTQIMFETFNTPAMYVAIQAVLSLYASGRTTGIVMDSGDGVTHTVPIYEGYALPHAILRLDLAGRDLTDYLMKILTERGYSF
Splooce       HPVLLTEAPLNPKANREKMTQIMFETFNTPAMYVAIQAVLSLYASGRTTGIVMDSGDGVTHTVPIYEGYALPHAILRLDLAGRDLTDYLMKILTERGYSF

Uniprot       TTTAEREIVRDIKEKLCYVALDFEQEMATAASSSSLEKSYELPDGQVITIGNERFRCPEALFQPSFLGMESCGIHETTFNSIMKCDVDIRKDLYANTVLS
Splooce       TTTAEREIVRDIKEKLCYVALDFEQEMATAASSSSLEKSYELPDGQVITIGNERFRCPEALFQPSFLGMESCGIHETTFNSIMKCDVDIRKDLYANTVLS

Uniprot       GGTTMYPGIADRMQKEITALAPSTMKIK====IIAPPERKYSVWIGGSILASLSTFQQMWISKQEYDESGPSIVHRKCF
Splooce       GGTTMYPGIADRMQKEITALAPSTMKIKVSRGIIAPPERKYSVWIGGSILASLSTFQQMWISKQEYDESGPSIVHRKCF

----------------------------------------------------------------------------------------------------

Q9NYU2 (Uniprot)	versus
NM_020120#(-s-:2_U7505651436877) (Splooce)

For more details about the Alternative Splicing Event -> Link to Splooce page

Peptides that support the ASE (Splooce-specific):
KDDSFLR (PEAKS)

Alignment:
Uniprot       MGCKGDASGACAAGALPVTGVCYKMGVLVVLTVLWLFSSVKADSKAITTSLTTKWFSTPLLLEASEFLAEDSQEKFWNFVEASQNIGSSDHDGTDYSYYH
Splooce       MGCKGDASGACAAGALPVTGVCYKMGVLVVLTVLWLFSSVKADSKAITTSLTTKWFSTPLLLEASEFLAEDSQEKFWNFVEASQNIGSSDHDGTDYSYYH

Uniprot       AILEAAFQFLSPLQQNLFKFCLSLRSYSATIQAFQQIAADEPPPEGCNSFFSVHGKKTCESDTLEALLLTASERPKPLLFKGDHRYPSSNPESPVVIFYS
Splooce       AILEAAFQFLSPLQQNLFKFCLSLRSYSATIQAFQQIAADEPPPEGCNSFFSVHGKKTCESDTLEALLLTASERPKPLLFKGDHRYPSSNPESPVVIFYS

Uniprot       EIGSEEFSNFHRQLISKSNAGKINYVFRHYIFNPRKEPVYLSGYGVELAIKSTEYKAKDDTQVKGTEVNTTVIGENDPIDEVQGFLFGKLRDLHPDLEGQ
Splooce       EIGSEEFSNFHRQLISKSNAGKINYVFRHYIFNPRKEPVYLSGYGVELAIKSTEYKAKDDTQVKGTEVNTTVIGENDPIDEVQGFLFGKLRDLHPDLEGQ

Uniprot       LKELRKHLVESTNEMAPLKVWQLQDLSFQTAARILASPVELALVVMKDLSQNFPTKARAITKTAVSSELRTEVEENQKYFKGTLGLQPGDSALFINGLHM
Splooce       LKELRKHLVESTNEMAPLKVWQLQDLSFQTAARILASPVELALVVMKDLSQNFPTKARAITKTAVSSELRTEVEENQKYFKGTLGLQPGDSALFINGLHM

Uniprot       DLDTQDIFSLFDVLRNEARVMEGLHRLGIEGLSLHNVLKLNIQPSEADYAVDIRSPAISWVNNLEVDSRYNSWPSSLQELLRPTFPGVIRQIRKNLHNMV
Splooce       DLDTQDIFSLFDVLRNEARVMEGLHRLGIEGLSLHNVLKLNIQPSEADYAVDIRSPAISWVNNLEVDSRYNSWPSSLQELLRPTFPGVIRQIRKNLHNMV

Uniprot       FIVDPAHETTAELMNTAEMFLSNHIPLRIGFIFVVNDSEDVDGMQDAGVAVLRAYNYVAQEVDDYHAFQTLTHIYNKVRTGEKVKVEHVVSVLEKKYPYV
Splooce       FIVDPAHETTAELMNTAEMFLSNHIPLRIGFIFVVNDSEDVDGMQDAGVAVLRAYNYVAQEVDDYHAFQTLTHIYNKVRTGEKVKVEHVVSVLEKKYPYV

Uniprot       EVNSILGIDSAYDRNRKEARGYYEQTGVGPLPVVLFNGMPFEREQLDPDELETITMHKILETTTFFQRAVYLGELPHDQDVVEYIMNQPNVVPRINSRIL
Splooce       EVNSILGIDSAYDRNRKEARGYYEQTGVGPLPVVLFNGMPFEREQLDPDELETITMHKILETTTFFQRAVYLGELPHDQDVVEYIMNQPNVVPRINSRIL

Uniprot       TAERDYLDLTASNNFFVDDYARFTILDSQGKTAAVANSMNYLTKKGMSSKEIYDDSFIRPVTFWIVGDFDSPSGRQLLYDAIKHQKSSNNVRISMINNPA
Splooce       TAERDYLDLTASNNFFVDDYARFTILDSQGKTAAVANSMNYLTKK========DDSFIRPVTFWIVGDFDSPSGRQLLYDAIKHQKSSNNVRISMINNPA

Uniprot       KEISYENTQISRAIWAALQTQTSNAAKNFITKMAKEGAAEALAAGADIAEFSVGGMDFSLFKEVFESSKMDFILSHAVYCRDVLKLKKGQRAVISNGRII
Splooce       KEISYENTQISRAIWAALQTQTSNAAKNFITKMAKEGAAEALAAGADIAEFSVGGMDFSLFKEVFESSKMDFILSHAVYCRDVLKLKKGQRAVISNGRII

Uniprot       GPLEDSELFNQDDFHLLENIILKTSGQKIKSHIQQLRVEEDVASDLVMKVDALLSAQPKGDPRIEYQFFEDRHSAIKLRPKEGETYFDVVAVVDPVTREA
Splooce       GPLEDSELFNQDDFHLLENIILKTSGQKIKSHIQQLRVEEDVASDLVMKVDALLSAQPKGDPRIEYQFFEDRHSAIKLRPKEGETYFDVVAVVDPVTREA

Uniprot       QRLAPLLLVLAQLINMNLRVFMNCQSKLSDMPLKSFYRYVLEPEISFTSDNSFAKGPIAKFLDMPQSPLFTLNLNTPESWMVESVRTPYDLDNIYLEEVD
Splooce       QRLAPLLLVLAQLINMNLRVFMNCQSKLSDMPLKSFYRYVLEPEISFTSDNSFAKGPIAKFLDMPQSPLFTLNLNTPESWMVESVRTPYDLDNIYLEEVD

Uniprot       SVVAAEYELEYLLLEGHCYDITTGQPPRGLQFTLGTSANPVIVDTIVMANLGYFQLKANPGAWILRLRKGRSEDIYRIYSHDGTDSPPDADEVVIVLNNF
Splooce       SVVAAEYELEYLLLEGHCYDITTGQPPRGLQFTLGTSANPVIVDTIVMANLGYFQLKANPGAWILRLRKGRSEDIYRIYSHDGTDSPPDADEVVIVLNNF

Uniprot       KSKIIKVKVQKKADMVNEDLLSDGTSENESGFWDSFKWGFTGQKTEEVKQDKDDIINIFSVASGHLYERFLRIMMLSVLKNTKTPVKFWFLKNYLSPTFK
Splooce       KSKIIKVKVQKKADMVNEDLLSDGTSENESGFWDSFKWGFTGQKTEEVKQDKDDIINIFSVASGHLYERFLRIMMLSVLKNTKTPVKFWFLKNYLSPTFK

Uniprot       EFIPYMANEYNFQYELVQYKWPRWLHQQTEKQRIIWGYKILFLDVLFPLVVDKFLFVDADQIVRTDLKELRDFNLDGAPYGYTPFCDSRREMDGYRFWKS
Splooce       EFIPYMANEYNFQYELVQYKWPRWLHQQTEKQRIIWGYKILFLDVLFPLVVDKFLFVDADQIVRTDLKELRDFNLDGAPYGYTPFCDSRREMDGYRFWKS

Uniprot       GYWASHLAGRKYHISALYVVDLKKFRKIAAGDRLRGQYQGLSQDPNSLSNLDQDLPNNMIHQVPIKSLPQEWLWCETWCDDASKKRAKTIDLCNNPMTKE
Splooce       GYWASHLAGRKYHISALYVVDLKKFRKIAAGDRLRGQYQGLSQDPNSLSNLDQDLPNNMIHQVPIKSLPQEWLWCETWCDDASKKRAKTIDLCNNPMTKE

Uniprot       PKLEAAVRIVPEWQDYDQEIKQLQIRFQKEKETGALYKEKTKEPSREGPQKREEL
Splooce       PKLEAAVRIVPEWQDYDQEIKQLQIRFQKEKETGALYKEKTKEPSREGPQKREEL

----------------------------------------------------------------------------------------------------

Q9Y320 (Uniprot)	versus
NM_015959#(-t:11_T1316950952235) (Splooce)

For more details about the Alternative Splicing Event -> Link to Splooce page

Peptides that support the ASE (Splooce-specific):
TIDELER (MAXQUANT)

Alignment:
Uniprot       MAVLAPLIALVYSVPRLSRWLAQPYYLLSALLSAAFLLVRKLPPLCHGLPTQREDGNPCDFDWREVEILMFLSAIVMMKNRRSITVEQHIGNIFMFSKVA
Splooce       MAVLAPLIALVYSVPRLSRWLAQPYYLLSALLSAAFLLVRKLPPLCHGLPTQREDGNPCDFDWREVEILMFLSAIVMMKNRRSITVEQHIGNIFMFSKVA

Uniprot       NTILFFRLDIRMGLLYITLCIVFLMTCKPPLYMGPEYIKYFNDKTIDEELERDKRVTWIVEFFANWSNDCQSFAPIYADLSLKYNCTGLNFGKVDVGRYT
Splooce       NTILFFRLDIRMGLLYITLCIVFLMTCKPPLYMGPEYIKYFNDKTID-ELERDKRVTWIVEFFANWSNDCQSFAPIYADLSLKYNCTGLNFGKVDVGRYT

Uniprot       DVSTRYKVSTSPLTKQLPTLILFQGGKEAMRRPQIDKKGRAVSWTFSEENVIREFNLNELYQRAKKLSKAGDNIPEEQPVASTPTTVSDGENKKDK
Splooce       DVSTRYKVSTSPLTKQLPTLILFQGGKEAMRRPQIDKKGRAVSWTFSEENVIREFNLNELYQRAKKLSKAGDNIPEEQPVASTPTTVSDGENKKDK

----------------------------------------------------------------------------------------------------

P04075 (Uniprot)	versus
NM_184043#(-s-s-:16_A1380365413111) (Splooce)

For more details about the Alternative Splicing Event -> Link to Splooce page

Peptides that support the ASE (Splooce-specific):
NGIVPIVEPEILPDGDHDLK (MAXQUANT)
NGIVPIVEPEILPDGDHDLKR (MAXQUANT)

Alignment:
Uniprot       MPYQYPALTPEQKKELSDIAHRIVAPGKGILAADESTGSIAKRLQSIGTENTEENRRFYRQLLLTADDRVNPCIGGVILFHETLYQKADDGRPFPQVIKS
Splooce       MPYQYPALTPEQKKELSDIAHRIVAPGKGILAADESTGSIAKRLQSIGTENTEENRRFYRQLLLTADDRVNPCIGGVILFHETLYQKADDGRPFPQVIKS

Uniprot       KGGVVGIKVDKGVVPLAGTNGETTTQGLDGLSERCAQYKKDGADFAKWRCVLKIGEHTPSALAIMENANVLARYASICQQNGIVPIVEPEILPDGDHDLK
Splooce       KGGVVGIK------------------------------------------------------------------------NGIVPIVEPEILPDGDHDLK

Uniprot       RCQYVTEKVLAAVYKALSDHHIYLEGTLLKPNMVTPGHACTQKFSHEEIAMATVTALRRTVPPAVTGITFLSGGQSEEEASINLNAINKCPLLKPWALTF
Splooce       RCQYVTEKVLAAVYKALSDHHIYLEGTLLKPNMVTPGHACTQKFSHEEIAMATVTALRRTVPPAVTGITFLSGGQSEEEASINLNAINKCPLLKPWALTF

Uniprot       SYGRALQASALKAWGGKKENLKAAQEEYVKRALANSLACQGKYTPSGQAGAAASESLFVSNHAY
Splooce       SYGRALQASALKAWGGKKENLKAAQEEYVKRALANSLACQGKYTPSGQAGAAASESLFVSNHAY

----------------------------------------------------------------------------------------------------

P28070 (Uniprot)	versus
NM_002796#(-s-:1_P3566367547044) (Splooce)

For more details about the Alternative Splicing Event -> Link to Splooce page

Peptides that support the ASE (Splooce-specific):
QVLGQMVFLGYVDMLGVAYEAPSLATGYGAYLAQPLLR (MAXQUANT)

Alignment:
Uniprot       MEAFLGSRSGLWAGGPAPGQFYRIPSTPDSFMDPASALYRGPITRTQNPMVTGTSVLGVKFEGGVVIAADMLGSYGSLARFRNISRIMRVNNSTMLGASG
Splooce       MEAFLGSRSGLWAGGPAPGQFYRIPSTPDSFMDPASALYRGPITRTQNPMVTGTSVLGVKFEGGVVIAADMLGSYGSLARFRNISRIMRVNNSTMLGASG

Uniprot       DYADFQYLKQVLGQMVIDEELLGDGHSYSPRAIHSWLTRAMYSRRSKMNPLWNTMVIGGYADGESFLGYVDMLGVAYEAPSLATGYGAYLAQPLLREVLE
Splooce       DYADFQYLKQVLGQMV-------------------------------------------------FLGYVDMLGVAYEAPSLATGYGAYLAQPLLREVLE

Uniprot       KQPVLSQTEARDLVERCMRVLYYRDARSYNRFQIATVTEKGVEIEGPLSTETNWDIAHMISGFE
Splooce       KQPVLSQTEARDLVERCMRVLYYRDARSYNRFQIATVTEKGVEIEGPLSTETNWDIAHMISGFE

----------------------------------------------------------------------------------------------------

P46379 (Uniprot)	versus
NM_080703#(-s-:6_B9836219013769) (Splooce)

For more details about the Alternative Splicing Event -> Link to Splooce page

Peptides that support the ASE (Splooce-specific):
SLVQVQPGVDIIR (MAXQUANT)

Alignment:
Uniprot       MEPNDSTSTAVEEPDSLEVLVKTLDSQTRTFIVGAQMNVKEFKEHIAASVSIPSEKQRLIYQGRVLQDDKKLQEYNVGGKVIHLVERAPPQTHLPSGASS
Splooce       MEPNDSTSTAVEEPDSLEVLVKTLDSQTRTFIVGAQMNVKEFKEHIAASVSIPSEKQRLIYQGRVLQDDKKLQEYNVGGKVIHLVERAPPQTHLPSGASS

Uniprot       GTGSASATHGGGSPPGTRGPGASVHDRNANSYVMVGTFNLPSDGSAVDVHINMEQAPIQSEPRVRLVMAQHMIRDIQTLLSRMECRGGPQPQHSQPPPQP
Splooce       GTGSASATHGGGSPPGTRGPGASVHDRNANSYVMVGTFNLPSDGSAVDVHINMEQAPIQSEPRVRLVMAQHMIRDIQTLLSRMECRGGPQPQHSQPPPQP

Uniprot       PAVTPEPVALSSQTSEPVESEAPPREPMEAEEVEERAPAQNPELTPGPAPAGPTPAPETNAPNHPSPAEYVEVLQELQRLESRLQPFLQRYYEVLGAAAT
Splooce       PAVTPEPVALSSQTSEPVESEAPPREPMEAEEVEERAPAQNPELTPGPAPAGPTPAPETNAPNHPSPAEYVEVLQELQRLESRLQPFLQRYYEVLGAAAT

Uniprot       TDYNNNHEGREEDQRLINLVGESLRLLGNTFVALSDLRCNLACTPPRHLHVVRPMSHYTTPMVLQQAAIPIQINVGTTVTMTGNGTRPPPTPNAEAPPPG
Splooce       TDYNNNHEGREEDQRLINLVGESLRLLGNTFVALSDLRCNLACTPPRHLHVVRPMSHYTTPMVLQQAAIPIQINVGTTVTMTGNGTRPPPTPNAEAPPPG

Uniprot       PGQASSVAPSSTNVESSAEGAPPPGPAPPPATSHPRVIRISHQSVEPVVMMHMNIQDSGTQPGGVPSAPTGPLGPPGHGQTLGQQVPGFPTAPTRVVIAR
Splooce       PGQASSVAPSSTNVESSAEGAPPPGPAPPPATSHPRVIRISHQSVEPVVMMHMNIQDSGTQPGGVPSAPTGPLGPPGHGQTLGQQVPGFPTAPTRVVIAR

Uniprot       PTPPQARPSHPGGPPVSGTLQGAGLGTNASLAQMVSGLVGQLLMQPVLVAQGTPGMAPPPAPATASASAGTTNTATTAGPAPGGPAQPPPTPQPSMADLQ
Splooce       PTPPQARPSHPGGPPVSGTLQGAGLGTNASLAQMVSGLVGQLLMQPVLVAQGTPGMAPPPAPATASASAGTTNTATTAGPAPGGPAQPPPTPQPSMADLQ

Uniprot       FSQLLGNLLGPAGPGAGGSGVASPTITVAMPGVPAFLQGMTDFLQATQTAPPPPPPPPPPPPAPEQQTMPPPGSPSGGAGSPGGLGLESLSPEFFTSVVQ
Splooce       FSQLLGNLLGPAGPGAGGSGVASPTITVAMPGVPAFLQGMTDFLQATQTAPPPPPPPPPPPPAPEQQTMPPPGSPSGGAGSPGGLGLESLSPEFFTSVVQ

Uniprot       GVLSSLLGSLGARAGSSESIAAFIQRLSGSSNIFEPGADGALGFFGALLSLLCQNFSMVDVVMLLHGHFQPLQRLQPQLRSFFHQHYLGGQEPTPSNIRM
Splooce       GVLSSLLGSLGARAGSSESIAAFIQRLSGSSNIFEPGADGALGFFGALLSLLCQNFSMVDVVMLLHGHFQPLQRLQPQLRSFFHQHYLGGQEPTPSNIR-

Uniprot       ATHTLITGLEEYVRESFSLVQVQPGVDIIRTNLEFLQEQFNSIAAHVLHCTDSGFGARLLELCNQGLFECLALNLHCLGGQQMELAAVINGRIRRMSRGV
Splooce       -----------------SLVQVQPGVDIIRTNLEFLQEQFNSIAAHVLHCTDSGFGARLLELCNQGLFECLALNLHCLGGQQMELAAVINGRIRRMSRGV

Uniprot       NPSLVSWLTTMMGLRLQVVLEHMPVGPDAILRYVRRVGDPPQPLPEEPMEVQGAERASPEPQRENASPAPGTTAEEAMSRGPPPAPEGGSRDEQDGASAE
Splooce       NPSLVSWLTTMMGLRLQVVLEHMPVGPDAILRYVRRVGDPPQPLPEEPMEVQGAERASPEPQRENASPAPGTTAEEAMSRGPPPAPEGGSRDEQDGASAE

Uniprot       TEPWAAAVPPEWVPIIQQDIQSQRKVKPQPPLSDAYLSGMPAKRRKTMQGEGPQLLLSEAVSRAAKAAGARPLTSPESLSRDLEAPEVQESYRQQLRSDI
Splooce       TEPWAAAVPPEWVPIIQQDIQSQRKVKPQPPLSDAYLSGMPAKRRKTMQGEGPQLLLSEAVSRAAKAAGARPLTSPESLSRDLEAPEVQESYRQQLRSDI

Uniprot       QKRLQEDPNYSPQRFPNAQRAFADDP
Splooce       QKRLQEDPNYSPQRFPNAQRAFADDP

----------------------------------------------------------------------------------------------------

Q02809 (Uniprot)	versus
NM_000302#(-s-s-s-s-s-:1_P2548238883685) (Splooce)

For more details about the Alternative Splicing Event -> Link to Splooce page

Peptides that support the ASE (Splooce-specific):
LFIHNHDWK (MAXQUANT)

Alignment:
Uniprot       MRPLLLLALLGWLLLAEAKGDAKPEDNLLVLTVATKETEGFRRFKRSAQFFNYKIQALGLGEDWNVEKGTSAGGGQKVRLLKKALEKHADKEDLVILFAD
Splooce       MRPLLLLALLGWLLLAEAKGDAKPEDNLLVLTVATKETEGFRRFKRSAQFFNYKIQALGLGEDWNVEKGTSAGGGQKVRLLKKALEKHADKEDLVILFAD

Uniprot       SYDVLFASGPRELLKKFRQARSQVVFSAEELIYPDRRLETKYPVVSDGKRFLGSGGFIGYAPNLSKLVAEWEGQDSDSDQLFYTKIFLDPEKREQINITL
Splooce       SYDVLFASGPRELLKKFRQARSQVVFSAEELIYPDRRLETKYPVVSDGKRFLGSGGFIGYAPNLSKLVAEWEGQDSDSDQLFYTKIFLDPEKREQINITL

Uniprot       DHRCRIFQNLDGALDEVVLKFEMGHVRARNLAYDTLPVLIHGNGPTKLQLNYLGNYIPRFWTFETGCTVCDEGLRSLKGIGDEALPTVLVGVFIEQPTPF
Splooce       DHRCRIFQNLDGALDEVVLKFEMGHVRARNLAYDTLPVLIHGNGPTKLQLNYLGNYIPRFWTFETGCTVCDEGLRSLKGIGDEALPTVLVGVFIEQPTPF

Uniprot       VSLFFQRLLRLHYPQKHMRLFIHNHEQHHKAQVEEFLAQHGSEYQSVKLVGPEVRMANADARNMGADLCRQDRSCTYYFSVDADVALTEPNSLRLLIQQN
Splooce       VSLFFQRLLRLHYPQKHMRLFIHNH---------------------------------------------------------------------------

Uniprot       KNVIAPLMTRHGRLWSNFWGALSADGYYARSEDYVDIVQGRRVGVWNVPYISNIYLIKGSALRGELQSSDLFHHSKLDPDMAFCANIRQQDVFMFLTNRH
Splooce       ----------------------------------------------------------------------------------------------------

Uniprot       TLGHLLSLDSYRTTHLHNDLWEVFSNPEDWKEKYIHQNYTKALAGKLVETPCPDVYWFPIFTEVACDELVEEMEHFGQWSLGNNKDNRIQGGYENVPTID
Splooce       ----------------------------DWKEKYIHQNYTKALAGKLVETPCPDVYWFPIFTEVACDELVEEMEHFGQWSLGNNKDNRIQGGYENVPTID

Uniprot       IHMNQIGFEREWHKFLLEYIAPMTEKLYPGYYTRAQFDLAFVVRYKPDEQPSLMPHHDASTFTINIALNRVGVDYEGGGCRFLRYNCSIRAPRKGWTLMH
Splooce       IHMNQIGFEREWHKFLLEYIAPMTEKLYPGYYTRAQFDLAFVVRYKPDEQPSLMPHHDASTFTINIALNRVGVDYEGGGCRFLRYNCSIRAPRKGWTLMH

Uniprot       PGRLTHYHEGLPTTRGTRYIAVSFVDP
Splooce       PGRLTHYHEGLPTTRGTRYIAVSFVDP

----------------------------------------------------------------------------------------------------

P63241 (Uniprot)	versus
NM_001970#(-s-:17_E2425347078113) (Splooce)

For more details about the Alternative Splicing Event -> Link to Splooce page

Peptides that support the ASE (Splooce-specific):
LIGIQDGYLSLLQDSGEVR (MAXQUANT)

Alignment:
Uniprot       MADDLDFETGDAGASATFPMQCSALRKNGFVVLKGRPCKIVEMSTSKTGKHGHAKVHLVGIDIFTGKKYEDICPSTHNMDVPNIKRNDFQLIGIQDGYLS
Splooce       MADDLDFETGDAGASATFPMQCSALRKNGFVVLKGRPCKIVEMSTSKTGKHGHAK-----------------------------------LIGIQDGYLS

Uniprot       LLQDSGEVREDLRLPEGDLGKEIEQKYDCGEEILITVLSAMTEEAAVAIKAMAK
Splooce       LLQDSGEVREDLRLPEGDLGKEIEQKYDCGEEILITVLSAMTEEAAVAIKAMAK

----------------------------------------------------------------------------------------------------

Q15021 (Uniprot)	versus
NM_014865#(-s-s-s-s-s-s-s-:12_N9392325028908) (Splooce)

For more details about the Alternative Splicing Event -> Link to Splooce page

Peptides that support the ASE (Splooce-specific):
VTWNSNSVSLVK (MAXQUANT)

Alignment:
Uniprot       MAPQMYEFHLPLSPEELLKSGGVNQYVVQEVLSIKHLPPQLRAFQAAFRAQGPLAMLQHFDTIYSILHHFRSIDPGLKEDTLQFLIKVVSRHSQELPAIL
Splooce       MAPQMYEFHLPLSPEELLKSGGVNQYVVQEVLSIKHLPPQLRAFQAAFRAQGPLAMLQHFDTIYSILHHFRSIDPGLKEDTLQFLIKVVSRHSQELPAIL

Uniprot       DDTTLSGSDRNAHLNALKMNCYALIRLLESFETMASQTNLVDLDLGGKGKKARTKAAHGFDWEEERQPILQLLTQLLQLDIRHLWNHSIIEEEFVSLVTG
Splooce       DDTTLSGSDRNAHLNALKMNCYALIRLLESFETMASQTNLVDLDLGGKGKKARTKAAHGFDWEEERQPILQLLTQLLQLDIRHLWNHSIIEEEFVSLVTG

Uniprot       CCYRLLENPTINHQKNRPTREAITHLLGVALTRYNHMLSATVKIIQMLQHFEHLAPVLVAAVSLWATDYGMKSIVGEIVREIGQKCPQELSRDPSGTKGF
Splooce       CCYRLLENPTINHQKNRPTREAITHLLGVALTRYNHMLSATVKIIQMLQHFEHLAPVLVAAVSLWATDYGMKSIVGEIVREIGQKCPQELSRDPSGTKGF

Uniprot       AAFLTELAERVPAILMSSMCILLDHLDGENYMMRNAVLAAMAEMVLQVLSGDQLEAAARDTRDQFLDTLQAHGHDVNSFVRSRVLQLFTRIVQQKALPLT
Splooce       AAFLTELAERVPAILMSSMCILLDHLDGENYMMRNAVLAAMAEMVLQVLSGDQLEAAARDTRDQFLDTLQAHGHDVNSFVRSRVLQLFTRIVQQKALPLT

Uniprot       RFQAVVALAVGRLADKSVLVCKNAIQLLASFLANNPFSCKLSDADLAGPLQKETQKLQEMRAQRRTAAASAVLDPEEEWEAMLPELKSTLQQLLQLPQGE
Splooce       RFQAVVALAVGRLADKSVLVCKNAIQLLASFLANNPFSCKLSDADLAGPLQKETQKLQEMRAQRRTAAASAVLDPEEEWEAMLPELKSTLQQLLQLPQGE

Uniprot       EEIPEQIANTETTEDVKGRIYQLLAKASYKKAIILTREATGHFQESEPFSHIDPEESEETRLLNILGLIFKGPAASTQEKNPRESTGNMVTGQTVCKNKP
Splooce       EEIPEQIANTETTEDVKGRIYQLLAKASYKKAIILTREATGHFQESEPFSHIDPEESEETRLLNILGLIFKGPAASTQEKNPRESTGNMVTGQTVCKNKP

Uniprot       NMSDPEESRGNDELVKQEMLVQYLQDAYSFSRKITEAIGIISKMMYENTTTVVQEVIEFFVMVFQFGVPQALFGVRRMLPLIWSKEPGVREAVLNAYRQL
Splooce       NMSDPEESRGNDELVKQEMLVQYLQDAYSFSRKITEAIGIISKMMYENTTTVVQEVIEFFVMVFQFGVPQALFGVRRMLPLIWSKEPGVREAVLNAYRQL

Uniprot       YLNPKGDSARAKAQALIQNLSLLLVDASVGTIQCLEEILCEFVQKDELKPAVTQLLWERATEKVACCPLERCSSVMLLGMMARGKPEIVGSNLDTLVSIG
Splooce       YLNPKGDSARAKAQALIQNLSLLLVDASVGTIQCLEEILCEFVQKDELKPAVTQLLWERATEKVACCPLERCSSVMLLGMMARGKPEIVGSNLDTLVSIG

Uniprot       LDEKFPQDYRLAQQVCHAIANISDRRKPSLGKRHPPFRLPQEHRLFERLRETVTKGFVHPDPLWIPFKEVAVTLIYQLAEGPEVICAQILQGCAKQALEK
Splooce       LDEKFPQDYRLAQQVCHAIANISDRRKPSLGKRHPPFRLPQEHRLFERLRETVTKGFVHPDPLWIPFKEVAVTLIYQLAEGPEVICAQILQGCAKQALEK

Uniprot       LEEKRTSQEDPKESPAMLPTFLLMNLLSLAGDVALQQLVHLEQAVSGELCRRRVLREEQEHKTKDPKEKNTSSETTMEEELGLVGATADDTEAELIRGIC
Splooce       LEEKRTSQEDPKESPAMLPTFLLMNLLSLAGDVALQQLVHLEQAVSGELCRRRVLREEQEHKTKDPKEKNTSSETTMEEELGLVGATADDTEAELIRGIC

Uniprot       EMELLDGKQTLAAFVPLLLKVCNNPGLYSNPDLSAAASLALGKFCMISATFCDSQLRLLFTMLEKSPLPIVRSNLMVATGDLAIRFPNLVDPWTPHLYAR
Splooce       EMELLDGKQTLAAFVPLLLKVCNNPGLYSNPDLSAAASLALGKFCMIRSFSRD---------------------------------------------DR

Uniprot       LRDPAQQVRKTAGLVMTHLILKDMVKVKGQVSEMAVLLIDPEPQIAALAKNFFNELSHKGNAIYNLLPDIISRLSDPELGVEEEPFHTIMKQLLSYITKD
Splooce       RRDTQENNSHSQSIGSQAQILG------------------------------------------SLFLSSLCRVS-------------------CRVTWN

Uniprot       KQTESLVEKLCQRFRTSRTERQQRDLAYCVSQLPLTERGLRKMLDNFDCFGDKLSDESIFSAFLSVVGKLRRGAKPEGKAIIDEFEQKLRACHTRGLDGI
Splooce       SNSVSLVKYLSVSFF-------------------------------------------------------------------------------------

Uniprot       KELEIGQAGSQRAPSAKKPSTGSRYQPLASTASDNDFVTPEPRRTTRRHPNTQQRASKKKPKVVFSSDESSEEDLSAEMTEDETPKKTTPILRASARRHR
Splooce       ----------------------------------------------------------------------------------------------------

Uniprot       S
Splooce       -

----------------------------------------------------------------------------------------------------

P10909 (Uniprot)	versus
NM_001831#(-s-:8_C4257872128985) (Splooce)

For more details about the Alternative Splicing Event -> Link to Splooce page

Peptides that support the ASE (Splooce-specific):
VTTGGVR (MAXQUANT)

Alignment:
Uniprot       MQVCSQPQRGCVREQSAINTAPPSAHNAASPGGARGHRVPLTEACKDSRIGGMMKTLLLFVGLLLTWESGQVLGDQTVSDNELQEMSNQGSKYVNKEIQN
Splooce       MQVCSQPQRGCVREQSAINTAPPSAHNAASPGGARGHRVPLTEACKDSRIGGMMKTLLLFVGLLLTWESGQVLGDQTVSDNELQEMSNQGSKYVNKEIQN

Uniprot       AVNGVKQIKTLIEKTNEERKTLLSNLEEAKKKKEDALNETRESETKLKELPGVCNETMMALWEECKPCLKQTCMKFYARVCRSGSGLVGRQLEEFLNQSS
Splooce       AVNGVKQIKTLIEKTNEERKTLLSNLEEAKKKKEDALNETRESETKLKELPGVCNETMMALWEECKPCLKQTCMKFYARVCRSGSGLVGRQLEEFLNQSS

Uniprot       PFYFWMNGDRIDSLLENDRQQTHMLDVMQDHFSRASSIIDELFQDRFFTREPQDTYHYLPFSLPHRRPHFFFPKSRIVRSLMPFSPYEPLNFHAMFQPFL
Splooce       PFYFWMNGDRIDSLLENDRQQTHMLDVMQDHFSRASSIIDELFQDRFFTREPQDTYHYLPFSLPHRRPHFFFPKSRIVRSLMPFSPYEPLNFHAMFQPFL

Uniprot       EMIHEAQQAMDIHFHSPAFQHPPTEFIREGDDDRTVCREIRHNSTGCLRMKDQCDKCREILSVDCSTNNPSQAKLRRELDESLQVAERLTRKYNELLKSY
Splooce       EMIHEAQQAMDIHFHSPAFQHPPTEFIREGDDDRTVCREIRHNSTGCLRMKDQCDKCREILSVDCSTNNPSQAKLRRELDESLQVAERLTRKYNELLKSY

Uniprot       QWKMLNTSSLLEQLNEQFNWVSRLANLTQGEDQYYLRVTTVASHTSDSDVPSGVTEVVVKLFDSDPITVTVPVEVSRKNPKFMETVAEKALQEYRKKHRE
Splooce       QWKMLNTSSLLEQLNEQFNWVSRLANLTQGEDQYYLRVTTGGVRCGCCFCTYGGI---------------------------------------------

Uniprot       E
Splooce       -

----------------------------------------------------------------------------------------------------

Q92879 (Uniprot)	versus
NM_006560#(-s-s-s-s-s-:11_C8191225721337) (Splooce)

For more details about the Alternative Splicing Event -> Link to Splooce page

Peptides that support the ASE (Splooce-specific):
LFIVVPK (MAXQUANT)

Alignment:
Uniprot       MNGTLDHPDQPDLDAIKMFVGQVPRTWSEKDLRELFEQYGAVYEINVLRDRSQNPPQSKGCCFVTFYTRKAALEAQNALHNMKVLPGMHHPIQMKPADSE
Splooce       MNGTLDHPDQPDLDAIKMFVGQVPRTWSEKDLRELFEQYGAVYEINVLRDRSQNPPQSKGCCFVTFYTRKAALEAQNALHNMKVLPGMHHPIQMKPADSE

Uniprot       KNNAVEDRKLFIGMISKKCTENDIRVMFSSFGQIEECRILRGPDGLSRGCAFVTFTTRAMAQTAIKAMHQAQTMEGCSSPMVVKFADTQKDKEQKRMAQQ
Splooce       KNNAVEDRKLFIGMISKKCTENDIRVMFSSFGQIEECRILRGPDGLSRGCAFVTFTTRAMAQTAIKAMHQAQTMEGCSSPMVVKFADTQKDKEQKRMAQQ

Uniprot       LQQQMQQISAASVWGNLAGLNTLGPQYLALLQQTASSGNLNTLSSLHPMGGLNAMQLQNLAALAAAASAAQNTPSGTNALTTSSSPLSVLTSSGSSPSSS
Splooce       LQQQMQQISAASVWGNLAGLNTLGPQYLALLQQTASSGNLNTLSSLHPMGDSCRLFIVVPKACRCGVKYIVQIPVSSASLMLELSPLTLC----------

Uniprot       SSNSVNPIASLGALQTLAGATAGLNVGSLAGMAALNGGLGSSGLSNGTGSTMEALTQAYSGIQQYAAAALPTLYNQNLLTQQSIGAAGSQKEGPEGANLF
Splooce       ----------------------------------------------------------------------------------------------------

Uniprot       IYHLPQEFGDQDLLQMFMPFGNVVSAKVFIDKQTNLSKCFGFVSYDNPVSAQAAIQSMNGFQIGMKRLKVQLKRSKNDSKPY
Splooce       ----------------------------------------------------------------------------------

----------------------------------------------------------------------------------------------------

P17936 (Uniprot)	versus
NM_001013398#(r:7_I6127448174333) (Splooce)

For more details about the Alternative Splicing Event -> Link to Splooce page

Peptides that support the ASE (Splooce-specific):
ARPTLWAAALTLLVLLRGLR (MAXQUANT)

Alignment:
Uniprot       MQRARPTLWAAALTLLVLLRGPPVARAGASSAGLGPVVRCEPCDARALAQCAPPPAVCAELVREPGCGCCLTCALSEGQPCGIYTERCGSGLRCQPSPDE
Splooce       MQRARPTLWAAALTLLVLLRG----------------------------------------------------------------------LRCQPSPDE

Uniprot       ARPLQALLDGRGLCVNASAVSRLRAYLLPAPPAPGEPPAPGNASESEEDRSAGSVESPSVSSTHRVSDPKFHPLHSKIIIIKKGHAKDSQRYKVDYESQS
Splooce       ARPLQALLDGRGLCVNASAVSRLRAYLLPAPPAPGEPPAPGNASESEEDRSAGSVESPSVSSTHRVSDPKFHPLHSKIIIIKKGHAKDSQRYKVDYESQS

Uniprot       TDTQNFSSESKRETEYGPCRREMEDTLNHLKFLNVLSPRGVHIPNCDKKGFYKKKQCRPSKGRKRGFCWCVDKYGQPLPGYTTKGKEDVHCYSMQSK
Splooce       TDTQNFSSESKRETEYGPCRREMEDTLNHLKFLNVLSPRGVHIPNCDKKGFYKKKQCRPSKGRKRGFCWCVDKYGQPLPGYTTKGKEDVHCYSMQSK

----------------------------------------------------------------------------------------------------

Q9Y4F5 (Uniprot)	versus
NM_015005#(-s-:14_K8903107423460) (Splooce)

For more details about the Alternative Splicing Event -> Link to Splooce page

Peptides that support the ASE (Splooce-specific):
LNDVIRFGYDHEKYTSQLQVSVK (MAXQUANT)

Alignment:
Uniprot       MRIPDQKYVTLKLNDVIRFGYDSNMYVLERVQHRVPEEALKHEKYTSQLQVSVKGLAPKRSEALPEHTPYCEASNPRPEKGDRRPGTEAASYRTPLYGQP
Splooce       MRIPDQKYVTLKLNDVIRFGYD-------------------HEKYTSQLQVSVKGLAPKRSEALPEHTPYCEASNPRPEKGDRRPGTEAASYRTPLYGQP

Uniprot       SWWGEDDGSTLPDAQRQGEPYPERPKGPVQQDGELHGFRAPAEPQGCSFRREPSYFEIPTKETPQPSQPPEVPAHEMPTKDAEAGGGGAAPVVQSHASFT
Splooce       SWWGEDDGSTLPDAQRQGEPYPERPKGPVQQDGELHGFRAPAEPQGCSFRREPSYFEIPTKETPQPSQPPEVPAHEMPTKDAEAGGGGAAPVVQSHASFT

Uniprot       IEFDDCSPGKMKIKDHITKFSLRQRRPPGKEATPGEMVSAETKVADWLVQNDPSLLHRVGPGDDRHSTKSDLPVHTRTLKGHKHEDGTQSDSEDPLAKAA
Splooce       IEFDDCSPGKMKIKDHITKFSLRQRRPPGKEATPGEMVSAETKVADWLVQNDPSLLHRVGPGDDRHSTKSDLPVHTRTLKGHKHEDGTQSDSEDPLAKAA

Uniprot       SAAGVPLEASGEQVRLQRQIKRDPQELLHNQQAFVIEFFDEDTPRKKRSQSFTHSPSGDPKADKRRGPTPADRDRPSVPAPVQAGGRSSGPQRAGSLKRE
Splooce       SAAGVPLEASGEQVRLQRQIKRDPQELLHNQQAFVIEFFDEDTPRKKRSQSFTHSPSGDPKADKRRGPTPADRDRPSVPAPVQAGGRSSGPQRAGSLKRE

Uniprot       KTEERLGSPSPASRTPARPFGSVGRRSRLAQDFMAQCLRESSPAARPSPEKVPPVLPAPLTPHGTSPVGPPTPPPAPTDPQLTKARKQEEDDSLSDAGTY
Splooce       KTEERLGSPSPASRTPARPFGSVGRRSRLAQDFMAQCLRESSPAARPSPEKVPPVLPAPLTPHGTSPVGPPTPPPAPTDPQLTKARKQEEDDSLSDAGTY

Uniprot       TIETEAQDTEVEEARKMIDQVFGVLESPELSRASSATFRPVIRGDRDESDDGGVAQRMALLQEFASRPLGAAPQAEHQGLPVPGSPGGQKWVSRWASLAD
Splooce       TIETEAQDTEVEEARKMIDQVFGVLESPELSRASSATFRPVIRGDRDESDDGGVAQRMALLQEFASRPLGAAPQAEHQGLPVPGSPGGQKWVSRWASLAD

Uniprot       SYSDPGLTEDGLGRRGGEPEGSLPVRMRRRLPQLPSERADSPAGPESSRRSGPGPPELDSEQPSRLFGQEELDPDSLSDASGSDGGRGPEPGVEPQDSRR
Splooce       SYSDPGLTEDGLGRRGGEPEGSLPVRMRRRLPQLPSERADSPAGPESSRRSGPGPPELDSEQPSRLFGQEELDPDSLSDASGSDGGRGPEPGVEPQDSRR

Uniprot       RSPQEGPTWSRGRRSPRAPGEPTPASFFIGDQNGDAVLSRKPLAAPGDGEGLGQTAQPSPPARDGVYVSANGRMVIQLRPGRSPEPDGPAPAFLRQESFT
Splooce       RSPQEGPTWSRGRRSPRAPGEPTPASFFIGDQNGDAVLSRKPLAAPGDGEGLGQTAQPSPPARDGVYVSANGRMVIQLRPGRSPEPDGPAPAFLRQESFT

Uniprot       KEPASGPPAPGKPPHISSHPLLQDLAATRAARMDFHSQDTHLILKETETALAALEARLLSNSVDAECEGGSTPRPPEDALSGDSDVDTASTVSLRSGKSG
Splooce       KEPASGPPAPGKPPHISSHPLLQDLAATRAARMDFHSQDTHLILKETETALAALEARLLSNSVDAECEGGSTPRPPEDALSGDSDVDTASTVSLRSGKSG

Uniprot       PSPTTPQPLRAQKEMSPSPPAAQDPGGTALVSAREQSSERQHHPLGPTDMGRGEPVRRSAIRRGHRPRGSLDWPSEERGPVLAHLPSSDVMASNHETPEA
Splooce       PSPTTPQPLRAQKEMSPSPPAAQDPGGTALVSAREQSSERQHHPLGPTDMGRGEPVRRSAIRRGHRPRGSLDWPSEERGPVLAHLPSSDVMASNHETPEA

Uniprot       TGAGRLGSRRKPAAPPPSPAAREEQSRSSASSQKGPQALTRSNSLSTPRPTRASRLRRARLGDASDTEAADGERGSLGNPEPVGRPAAEQAKKLSRLDIL
Splooce       TGAGRLGSRRKPAAPPPSPAAREEQSRSSASSQKGPQALTRSNSLSTPRPTRASRLRRARLGDASDTEAADGERGSLGNPEPVGRPAAEQAKKLSRLDIL

Uniprot       AMPRKRAGSFTGTSDPEAAPARTSFSGRSVELCCASRKPTMAEARAVSRKAANTATTTGPRQPFSRARSGSARYTSTTQTPRAGSSSRARSRAPGPRDTD
Splooce       AMPRKRAGSFTGTSDPEAAPARTSFSGRSVELCCASRKPTMAEARAVSRKAANTATTTGPRQPFSRARSGSARYTSTTQTPRAGSSSRARSRAPGPRDTD

Uniprot       DDEEEPDPYGFIVQTAEIAEIARLSQTLVKDVAILAQEIHDVAGDGDTLGSSEPAHSASLSNMPSTPASTISAREELVQRIPEASLNFQKVPPGSLNSRD
Splooce       DDEEEPDPYGFIVQTAEIAEIARLSQTLVKDVAILAQEIHDVAGDGDTLGSSEPAHSASLSNMPSTPASTISAREELVQRIPEASLNFQKVPPGSLNSRD

Uniprot       FDQNMNDSCEDALANKTRPRNREEVIFDNLMLNPVSQLSQAIRENTEHLAEKMKILFQNTGRAWEDLEARINAENEVPILKTSNKEISSILKELRRVQKQ
Splooce       FDQNMNDSCEDALANKTRPRNREEVIFDNLMLNPVSQLSQAIRENTEHLAEKMKILFQNTGRAWEDLEARINAENEVPILKTSNKEISSILKELRRVQKQ

Uniprot       LEVINAIVDPSGSLDLLTGNRSLASSAQPGLGKGRVAAQSPPSPASAEALLPALPLRNFPQRASCGPPSLPDPTFLPDAERFLI
Splooce       LEVINAIVDPSGSLDLLTGNRSLASSAQPGLGKGRVAAQSPPSPASAEALLPALPLRNFPQRASCGPPSLPDPTFLPDAERFLI

----------------------------------------------------------------------------------------------------

P12110 (Uniprot)	versus
NM_001849#(-s-s-s-s-s-s-s-s-s-s-:21_C6890372651011) (Splooce)

For more details about the Alternative Splicing Event -> Link to Splooce page

Peptides that support the ASE (Splooce-specific):
GDQGGKGEPGPPGDPGLTECDVMTYVRETCGCCDCEK (MAXQUANT)

Alignment:
Uniprot       MLQGTCSVLLLWGILGAIQAQQQEVISPDTTERNNNCPEKTDCPIHVYFVLDTSESVTMQSPTDILLFHMKQFVPQFISQLQNEFYLDQVALSWRYGGLH
Splooce       MLQGTCSVLLLWGILGAIQAQQQEVISPDTTERNNNCPEKTDCPIHVYFVLDTSESVTMQSPTDILLFHMKQFVPQFISQLQNEFYLDQVALSWRYGGLH

Uniprot       FSDQVEVFSPPGSDRASFIKNLQGISSFRRGTFTDCALANMTEQIRQDRSKGTVHFAVVITDGHVTGSPCGGIKLQAERAREEGIRLFAVAPNQNLKEQG
Splooce       FSDQVEVFSPPGSDRASFIKNLQGISSFRRGTFTDCALANMTEQIRQDRSKGTVHFAVVITDGHVTGSPCGGIKLQAERAREEGIRLFAVAPNQNLKEQG

Uniprot       LRDIASTPHELYRNDYATMLPDSTEIDQDTINRIIKVMKHEAYGECYKVSCLEIPGPSGPKGYRGQKGAKGNMGEPGEPGQKGRQGDPGIEGPIGFPGPK
Splooce       LRDIASTPHELYRNDYATMLPDSTEIDQDTINRIIKVMKHEAYGECYKVSCLEIPGPSGPKGYRGQKGAKGNMGEPGEPGQKGRQGDPGIEGPIGFPGPK

Uniprot       GVPGFKGEKGEFGADGRKGAPGLAGKNGTDGQKGKLGRIGPPGCKGDPGNRGPDGYPGEAGSPGERGDQGGKGDPGRPGRRGPPGEIGAKGSKGYQGNSG
Splooce       GVPGFKGEKGEFGADGRKGAPGLAGKNGTDGQKGKLGRIGPPGCKGDPGNRGPDGYPGEAGSPGERGDQGGKG---------------------------

Uniprot       APGSPGVKGAKGGPGPRGPKGEPGRRGDPGTKGSPGSDGPKGEKGDPGPEGPRGLAGEVGNKGAKGDRGLPGPRGPQGALGEPGKQGSRGDPGDAGPRGD
Splooce       ----------------------------------------------------------------------------------------------------

Uniprot       SGQPGPKGDPGRPGFSYPGPRGAPGEKGEPGPRGPEGGRGDFGLKGEPGRKGEKGEPADPGPPGEPGPRGPRGVPGPEGEPGPPGDPGLTECDVMTYVRE
Splooce       -------------------------------------------------------------------------------EPGPPGDPGLTECDVMTYVRE

Uniprot       TCGCCDCEKRCGALDVVFVIDSSESIGYTNFTLEKNFVINVVNRLGAIAKDPKSETGTRVGVVQYSHEGTFEAIQLDDERIDSLSSFKEAVKNLEWIAGG
Splooce       TCGCCDCEKRCGALDVVFVIDSSESIGYTNFTLEKNFVINVVNRLGAIAKDPKSETGTRVGVVQYSHEGTFEAIQLDDERIDSLSSFKEAVKNLEWIAGG

Uniprot       TWTPSALKFAYDRLIKESRRQKTRVFAVVITDGRHDPRDDDLNLRALCDRDVTVTAIGIGDMFHEKHESENLYSIACDKPQQVRNMTLFSDLVAEKFIDD
Splooce       TWTPSALKFAYDRLIKESRRQKTRVFAVVITDGRHDPRDDDLNLRALCDRDVTVTAIGIGDMFHEKHESENLYSIACDKPQQVRNMTLFSDLVAEKFIDD

Uniprot       MEDVLCPDPQIVCPDLPCQTELSVAQCTQRPVDIVFLLDGSERLGEQNFHKARRFVEQVARRLTLARRDDDPLNARVALLQFGGPGEQQVAFPLSHNLTA
Splooce       MEDVLCPDPQIVCPDLPCQTELSVAQCTQRPVDIVFLLDGSERLGEQNFHKARRFVEQVARRLTLARRDDDPLNARVALLQFGGPGEQQVAFPLSHNLTA

Uniprot       IHEALETTQYLNSFSHVGAGVVHAINAIVRSPRGGARRHAELSFVFLTDGVTGNDSLHESAHSMRKQNVVPTVLALGSDVDMDVLTTLSLGDRAAVFHEK
Splooce       IHEALETTQYLNSFSHVGAGVVHAINAIVRSPRGGARRHAELSFVFLTDGVTGNDSLHESAHSMRKQNVVPTVLALGSDVDMDVLTTLSLGDRAAVFHEK

Uniprot       DYDSLAQPGFFDRFIRWIC
Splooce       DYDSLAQPGFFDRFIRWIC

----------------------------------------------------------------------------------------------------

O43747 (Uniprot)	versus
NM_001030007#(-s-s-s-s-s-s-s-s-s-s-:16_A2274194244238) (Splooce)

For more details about the Alternative Splicing Event -> Link to Splooce page

Peptides that support the ASE (Splooce-specific):
NDDDSSEAMNDILAQANDLLDLLGGNDITPVIPTAPTSK (MAXQUANT)

Alignment:
Uniprot       MPAPIRLRELIRTIRTARTQAEEREMIQKECAAIRSSFREEDNTYRCRNVAKLLYMHMLGYPAHFGQLECLKLIASQKFTDKRIGYLGAMLLLDERQDVH
Splooce       MPAPIRLRELIRTIRTARTQAEEREMIQKECAAIRSSFREEDNTYRCRNVAKLLYMHMLGYPAHFGQLECLKLIASQKFTDKRIGYLGAMLLLDERQDVH

Uniprot       LLMTNCIKNDLNHSTQFVQGLALCTLGCMGSSEMCRDLAGEVEKLLKTSNSYLRKKAALCAVHVIRKVPELMEMFLPATKNLLNEKNHGVLHTSVVLLTE
Splooce       LLMTNCIKNDLNHSTQFVQGLALCTLGCMGSSEMCRDLAGEVEKLLKTSNSYLRKKAALCAVHVIRKVPELMEMFLPATKNLLNEKNHGVLHTSVVLLTE

Uniprot       MCERSPDMLAHFRKLVPQLVRILKNLIMSGYSPEHDVSGISDPFLQVRILRLLRILGRNDDDSSEAMNDILAQVATNTETSKNVGNAILYETVLTIMDIK
Splooce       MCERSPDMLAHFRKLVPQLVRILKNLIMSGYSPEHDVSGISDPFLQVRILRLLRILGRNDDDSSEAMNDILA----------------------------

Uniprot       SESGLRVLAINILGRFLLNNDKNIRYVALTSLLKTVQTDHNAVQRHRSTIVDCLKDLDVSIKRRAMELSFALVNGNNIRGMMKELLYFLDSCEPEFKADC
Splooce       ----------------------------------------------------------------------------------------------------

Uniprot       ASGIFLAAEKYAPSKRWHIDTIMRVLTTAGSYVRDDAVPNLIQLITNSVEMHAYTVQRLYKAILGDYSQQPLVQVAAWCIGEYGDLLVSGQCEEEEPIQV
Splooce       ----------------------------------------------------------------------------------------------------

Uniprot       TEDEVLDILESVLISNMSTSVTRGYALTAIMKLSTRFTCTVNRIKKVVSIYGSSIDVELQQRAVEYNALFKKYDHMRSALLERMPVMEKVTTNGPTEIVQ
Splooce       ----------------------------------------------------------------------------------------------------

Uniprot       TNGETEPAPLETKPPPSGPQPTSQANDLLDLLGGNDITPVIPTAPTSKPSSAGGELLDLLGDINLTGAPAAAPAPASVPQISQPPFLLDGLSSQPLFNDI
Splooce       -----------------------QANDLLDLLGGNDITPVIPTAPTSKPSSAGGELLDLLGDINLTGAPAAAPAPASVPQISQPPFLLDGLSSQPLFNDI

Uniprot       AAGIPSITAYSKNGLKIEFTFERSNTNPSVTVITIQASNSTELDMTDFVFQAAVPKTFQLQLLSPSSSIVPAFNTGTITQVIKVLNPQKQQLRMRIKLTY
Splooce       AAGIPSITAYSKNGLKIEFTFERSNTNPSVTVITIQASNSTELDMTDFVFQAAVPKTFQLQLLSPSSSIVPAFNTGTITQVIKVLNPQKQQLRMRIKLTY

Uniprot       NHKGSAMQDLAEVNNFPPQSWQ
Splooce       NHKGSAMQDLAEVNNFPPQSWQ

----------------------------------------------------------------------------------------------------

P61978 (Uniprot)	versus
NM_002140#(-s-s-s-:9_H8023218622095) (Splooce)

For more details about the Alternative Splicing Event -> Link to Splooce page

Peptides that support the ASE (Splooce-specific):
GGWLDLLLAK (MAXQUANT)

Alignment:
Uniprot       METEQPEETFPNTETNGEFGKRPAEDMEEEQAFKRSRNTDEMVELRILLQSKNAGAVIGKGGKNIKALRTDYNASVSVPDSSGPERILSISADIETIGEI
Splooce       METEQPEETFPNTETNGEFGKRPAEDMEEEQAFKRSRNTDEMVELRILLQSKNAGAVIGKGGKNIKALRTDYNASVSVPDSSGPERILSISADIETIGEI

Uniprot       LKKIIPTLEEGLQLPSPTATSQLPLESDAVECLNYQHYKGSDFDCELRLLIHQSLAGGIIGVKGAKIKELRENTQTTIKLFQECCPHSTDRVVLIGGKPD
Splooce       LKKIIPTLEEGLQLPSPTATSQLPLESDAVECLNYQHYKGSDFDCELRLLIHQSLAGGIIGVKGAKIKELRENTQTTIKLFQECCPHSTDRVVLIGGKPD

Uniprot       RVVECIKIILDLISESPIKGRAQPYDPNFYDETYDYGGFTMMFDDRRGRPVGFPMRGRGGFDRMPPGRGGRPMPPSRRDYDDMSPRRGPPPPPPGRGGRG
Splooce       RVVECIKIILDLISESPIKGRAQPYDPNFYDETYDYGGFTMMFDDRRGRPVGFPMRGRGGFDRMPPGRGGRPMPPSRRDYDDMSPRRGPPPPPPGRGGRG

Uniprot       GSRARNLPLPPPPPPRGGDLMAYDRRGRPGDRYDGMVGFSADETWDSAIDTWSPSEWQMAYEPQVEYHSYYSYAGGRGSYGDLGGPIITTQVTIPKDLAG
Splooce       GSRARNLPLPPPPPPRGG---------------------WLDLLLAKVVSGLNKS--VMSRELRSKLMSL------------------------------

Uniprot       SIIGKGGQRIKQIRHESGASIKIDEPLEGSEDRIITITGTQDQIQNAQYLLQNSVKQYADVEGF
Splooce       ----------------------------------------------------------------

----------------------------------------------------------------------------------------------------

E7EUT4 (Uniprot)	versus
NM_002046#(-t:12_G2869033650173) (Splooce)

For more details about the Alternative Splicing Event -> Link to Splooce page

Peptides that support the ASE (Splooce-specific):
WGDAGAEYVVESTGVFTTMEKAGKYDNSLK (MAXQUANT)

Alignment:
Uniprot       MGKVKVGVNGFGRIGRLVTRAAFNSGKVDIVAINDPFIDLNYMVYMFQYDSTHGKFHGTVKAENGKLVINGNPITIFQERDPSKIKWGDAGAEYVVESTG
Splooce       MGKVKVGVNGFGRIGRLVTRAAFNSGKVDIVAINDPFIDLNYMVYMFQYDSTHGKFHGTVKAENGKLVINGNPITIFQERDPSKIKWGDAGAEYVVESTG

Uniprot       VFTTMEKAGAHLQGGAKRVIISAPSADAPMFVMGVNHEKYDNSLKIISNASCTTNCLAPLAKVIHDNFGIVEGLMTTVHAITATQKTVDGPSGKLWRDGR
Splooce       VFTTMEKAG-----------------------------KYDNSLKIISNASCTTNCLAPLAKVIHDNFGIVEGLMTTVHAITATQKTVDGPSGKLWRDGR

Uniprot       GALQNIIPASTGAAKAVGKVIPELNGKLTGMAFRVPTANVSVVDLTCRLEKPAKYDDIKKVVKQASEGPLKGILGYTEHQVVSSDFNSDTHSSTFDAGAG
Splooce       GALQNIIPASTGAAKAVGKVIPELNGKLTGMAFRVPTANVSVVDLTCRLEKPAKYDDIKKVVKQASEGPLKGILGYTEHQVVSSDFNSDTHSSTFDAGAG

Uniprot       IALNDHFVKLISWYDNEFGYSNRVVDLMAHMASKE
Splooce       IALNDHFVKLISWYDNEFGYSNRVVDLMAHMASKE

----------------------------------------------------------------------------------------------------

Q70J99 (Uniprot)	versus
NM_199242#(-s-:17_U3162446018335) (Splooce)

For more details about the Alternative Splicing Event -> Link to Splooce page

Peptides that support the ASE (Splooce-specific):
FLEVELCYMNTNLVQENFSR (MAXQUANT)

Alignment:
Uniprot       MATLLSHPQQRPPFLRQAIKIRRRRVRDLQDPPPQMAPEIQPPSHHFSPEQRALLYEDALYTVLHRLGHPEPNHVTEASELLRYLQEAFHVEPEEHQQTL
Splooce       MATLLSHPQQRPPFLRQAIKIRRRRVRDLQDPPPQMAPEIQPPSHHFSPEQRALLYEDALYTVLHRLGHPEPNHVTEASELLRYLQEAFHVEPEEHQQTL

Uniprot       QRVRELEKPIFCLKATVKQAKGILGKDVSGFSDPYCLLGIEQGVGVPGGSPGSRHRQKAVVRHTIPEEETHRTQVITQTLNPVWDETFILEFEDITNASF
Splooce       QRVRELEKPIFCLKATVKQAKGILGKDVSGFSDPYCLLGIEQGVGVPGGSPGSRHRQKAVVRHTIPEEETHRTQVITQTLNPVWDETFILEFEDITNASF

Uniprot       HLDMWDLDTVESVRQKLGELTDLHGLRRIFKEARKDKGQDDFLGNVVLRLQDLRCREDQWYPLEPRTETYPDRGQCHLQFQLIHKRRATSASRSQPSYTV
Splooce       HLDMWDLDTVESVRQKLGELTDLHGLRRIFKEARKDKGQDDFLGNVVLRLQDLRCREDQWYPLEPRTETYPDRGQCHLQFQLIHKRRATSASRSQPSYTV

Uniprot       HLHLLQQLVSHEVTQHEAGSTSWDGSLSPQAATVLFLHATQKDLSDFHQSMAQWLAYSRLYQSLEFPSSCLLHPITSIEYQWIQGRLKAEQQEELAASFS
Splooce       HLHLLQQLVSHEVTQHEAGSTSWDGSLSPQAATVLFLHATQKDLSDFHQSMAQWLAYSRLYQSLEFPSSCLLHPITSIEYQWIQGRLKAEQQEELAASFS

Uniprot       SLLTYGLSLIRRFRSVFPLSVSDSPARLQSLLRVLVQMCKMKAFGELCPNTAPLPQLVTEALQTGTTEWFHLKQQHHQPMVQGIPEAGKALLGLVQDVIG
Splooce       SLLTYGLSLIRRFRSVFPLSVSDSPARLQSLLRVLVQMCKMKAFGELCPNTAPLPQLVTEALQTGTTEWFHLKQQHHQPMVQGIPEAGKALLGLVQDVIG

Uniprot       DLHQCQRTWDKIFHNTLKIHLFSMAFRELQWLVAKRVQDHTTVVGDVVSPEMGESLFQLYISLKELCQLRMSSSERDGVLALDNFHRWFQPAIPSWLQKT
Splooce       DLHQCQRTWDKIFHNTLKIHLFSMAFRELQWLVAKRVQDHTTVVGDVVSPEMGESLFQLYISLKELCQLRMSSSERDGVLALDNFHRWFQPAIPSWLQKT

Uniprot       YNEALARVQRAVQMDELVPLGELTKHSTSAVDLSTCFAQISHTARQLDWPDPEEAFMITVKFVEDTCRLALVYCSLIKARARELSSGQKDQGQAANMLCV
Splooce       YNEALARVQRAVQMDELVPLGELTKHSTSAVDLSTCFAQISHTARQLDWPDPEEAFMITVKFVEDTCRLALVYCSLIKARARELSSGQKDQGQAANMLCV

Uniprot       VVNDMEQLRLVIGKLPAQLAWEALEQRVGAVLEQGQLQNTLHAQLQSALAGLGHEIRTGVRTLAEQLEVGIAKHIQKLVGVRESVLPEDAILPLMKFLEV
Splooce       VVNDMEQLRLVIGKLPAQLAWEALEQRVGAVLEQGQLQNTLHAQLQSALAGLGHEIRTGVRTLAEQLEVGIAKHIQKLVGVRESVLPEDAILPLMKFLEV

Uniprot       ELCYMNTNLVQENFSSLLTLLWTHTLTVLVEAAASQRSSSLASNRLKIALQNLEICFHAEGCGLPPKALHTATFQALQRDLELQAASSRELIRKYFCSRI
Splooce       ELCYMNTNLVQENFS------------------RTWRSASTLR----------AVACHPRPCTLPPSRLCRGTWSCRR----PPAGNS---------SGS

Uniprot       QQQAETTSEELGAVTVKASYRASEQKLRVELLSASSLLPLDSNGSSDPFVQLTLEPRHEFPELAARETQKHKKDLHPLFDETFEFLVPAEPCRKAGACLL
Splooce       TSAAESSSRQK--------------------------PPLRSWGL-------------------------------------------------------

Uniprot       LTVLDYDTLGADDLEGEAFLPLREVPGLSGSEEPGEVPQTRLPLTYPAPNGDPILQLLEGRKGDREAQVFVRLRRHRAKQASQHALRPAP
Splooce       ------------------------------------------------------------------------------------------

----------------------------------------------------------------------------------------------------

P08572 (Uniprot)	versus
NM_001846#(-s-s-s-s-s-:13_C1454782858395) (Splooce)

For more details about the Alternative Splicing Event -> Link to Splooce page

Peptides that support the ASE (Splooce-specific):
VLTSTETQASQALLGK (MAXQUANT)

Alignment:
Uniprot       MGRDQRAVAGPALRRWLLLGTVTVGFLAQSVLAGVKKFDVPCGGRDCSGGCQCYPEKGGRGQPGPVGPQGYNGPPGLQGFPGLQGRKGDKGERGAPGVTG
Splooce       MGRDQRAVAGPALRRWLLLGTVTVGFLAQSVLAGVKKFDVPCGGRDCSGGCQCYPEKGGRGQPGPVGPQGYNGPPGLQGFPGLQGRKGDKGERGAPGVTG

Uniprot       PKGDVGARGVSGFPGADGIPGHPGQGGPRGRPGYDGCNGTQGDSGPQGPPGSEGFTGPPGPQGPKGQKGEPYALPKEERDRYRGEPGEPGLVGFQGPPGR
Splooce       PKGDVGARGVSGFPGADGIPGHPGQGGPRGRPGYDGCNGTQGDSGPQGPPGSEGFTGPPGPQGPKGQKGEPYALPKEERDRYRGEPGEPGLVGFQGPPGR

Uniprot       PGHVGQMGPVGAPGRPGPPGPPGPKGQQGNRGLGFYGVKGEKGDVGQPGPNGIPSDTLHPIIAPTGVTFHPDQYKGEKGSEGEPGIRGISLKGEEGIMGF
Splooce       PGHVGQMGPVGAPGRPGPPGPPGPKGQQGNRGLGFYGVKGEKGDVGQPGPNGIPSDTLHPIIAPTGVTFHPDQYKGEKGSEGEPGIRGISLKGEEGIMGF

Uniprot       PGLRGYPGLSGEKGSPGQKGSRGLDGYQGPDGPRGPKGEAGDPGPPGLPAYSPHPSLAKGARGDPGFPGAQGEPGSQGEPGDPGLPGPPGLSIGDGDQRR
Splooce       PGLRGYPGLSGEKGSPGQKGSRGLDGYQGPDGPRGPKGEAGDPGPPGLPAYSPHPSLAKGARGDPGFPGAQGEPGSQGEPGDPGLPGPPGLSIGDGDQRR

Uniprot       GLPGEMGPKGFIGDPGIPALYGGPPGPDGKRGPPGPPGLPGPPGPDGFLFGLKGAKGRAGFPGLPGSPGARGPKGWKGDAGECRCTEGDEAIKGLPGLPG
Splooce       GLPGEMGPKGFIGDPGIPALYGGPPGPDGKRGPPGPPGLPGPPGPDGFLFGLKGAKGRAGFPGLPGSPGARGPKGWKGDAGECRCTEGDEAIKGLPGLPG

Uniprot       PKGFAGINGEPGRKGDRGDPGQHGLPGFPGLKGVPGNIGAPGPKGAKGDSRTITTKGERGQPGVPGVPGMKGDDGSPGRDGLDGFPGLPGPPGDGIKGPP
Splooce       PKGFAGINGEPGRKGDRGDPGQHGLPGFPGLKGVPGNIGAPGPKGAKGDSRTITTKGERGQPGVPGVPGMKGDDGSPGRDGLDGFPGLPGPPGDGIKGPP

Uniprot       GDPGYPGIPGTKGTPGEMGPPGLGLPGLKGQRGFPGDAGLPGPPGFLGPPGPAGTPGQIDCDTDVKRAVGGDRQEAIQPGCIGGPKGLPGLPGPPGPTGA
Splooce       GDPGYPGIPGTKGTPGEMGPPGLGLPGLKGQRGFPGDAGLPGPPGFLGPPGPAGTPGQIDCDTDVKRAVGGDRQEAIQPGCIGGPKGLPGLPGPPGPTGA

Uniprot       KGLRGIPGFAGADGGPGPRGLPGDAGREGFPGPPGFIGPRGSKGAVGLPGPDGSPGPIGLPGPDGPPGERGLPGEVLGAQPGPRGDAGVPGQPGLKGLPG
Splooce       KGLRGIPGFAGADGGPGPRGLPGDAGREGFPGPPGFIGPRGSKGAVGLPGPDGSPGPIGLPGPDGPPGERGLPGEVLGAQPGPRGDAGVPGQPGLKGLPG

Uniprot       DRGPPGFRGSQGMPGMPGLKGQPGLPGPSGQPGLYGPPGLHGFPGAPGQEGPLGLPGIPGREGLPGDRGDPGDTGAPGPVGMKGLSGDRGDAGFTGEQGH
Splooce       DRGPPGFRGSQGMPGMPGLKGQPGLPGPSGQPGLYGPPGLHGFPGAPGQEGPLGLPGIPGREGLPGDRGDPGDTGAPGPVGMKGLSGDRGDAGFTGEQGH

Uniprot       PGSPGFKGIDGMPGTPGLKGDRGSPGMDGFQGMPGLKGRPGFPGSKGEAGFFGIPGLKGLAGEPGFKGSRGDPGPPGPPPVILPGMKDIKGEKGDEGPMG
Splooce       PGSPGFKGIDGMPGTPGLKGDRGSPGMDGFQGMPGLKGRPGFPGSKGEAGFFGIPGLKGLAGEPGFKGSRGDPGPPGPPPVILPGMKDIKGEKGDEGPMG

Uniprot       LKGYLGAKGIQGMPGIPGLSGIPGLPGRPGHIKGVKGDIGVPGIPGLPGFPGVAGPPGITGFPGFIGSRGDKGAPGRAGLYGEIGATGDFGDIGDTINLP
Splooce       LKGYLGAKGIQGMPGIPGLSGIPGLPGRPGHIKGVKGDIGVPGIPGLPGFPGVAGPPGITGFPGFIGSR-------------------------------

Uniprot       GRPGLKGERGTTGIPGLKGFFGEKGTEGDIGFPGITGVTGVQGPPGLKGQTGFPGLTGPPGSQGELGRIGLPGGKGDDGWPGAPGLPGFPGLRGIRGLHG
Splooce       -------VLTSTETQASQALLGKEVTQ--------------------ERPTPFQALWESQDRKE---------------------------TKELQGNEA

Uniprot       LPGTKGFPGSPGSDIHGDPGFPGPPGERGDPGEANTLPGPVGVPGQKGDQGAPGERGPPGSPGLQGFPGITPPSNISGAPGDKGAPGIFGLKGYRGPPGP
Splooce       HLGAQDFRGSLVS---------------------HPLPTSLGHLVTKGRQGYLA----------------------------------------------

Uniprot       PGSAALPGSKGDTGNPGAPGTPGTKGWAGDSGPQGRPGVFGLPGEKGPRGEQGFMGNTGPTGAVGDRGPKGPKGDPGFPGAPGTVGAPGIAGIPQKIAVQ
Splooce       ----------------------------------------------------------------------------------------------------

Uniprot       PGTVGPQGRRGPPGAPGEMGPQGPPGEPGFRGAPGKAGPQGRGGVSAVPGFRGDEGPIGHQGPIGQEGAPGRPGSPGLPGMPGRSVSIGYLLVKHSQTDQ
Splooce       ----------------------------------------------------------------------------------------------------

Uniprot       EPMCPVGMNKLWSGYSLLYFEGQEKAHNQDLGLAGSCLARFSTMPFLYCNPGDVCYYASRNDKSYWLSTTAPLPMMPVAEDEIKPYISRCSVCEAPAIAI
Splooce       ----------------------------------------------------------------------------------------------------

Uniprot       AVHSQDVSIPHCPAGWRSLWIGYSFLMHTAAGDEGGGQSLVSPGSCLEDFRATPFIECNGGRGTCHYYANKYSFWLTTIPEQSFQGSPSADTLKAGLIRT
Splooce       ----------------------------------------------------------------------------------------------------

Uniprot       HISRCQVCMKNL
Splooce       ------------

----------------------------------------------------------------------------------------------------

Q15369 (Uniprot)	versus
NM_005648#(f-:8_T8552562532298) (Splooce)

For more details about the Alternative Splicing Event -> Link to Splooce page

Peptides that support the ASE (Splooce-specific):
KAMLSGR (PEAKS)

Alignment:
Uniprot       MDGEEKTYGGCEGPDAMYVKLISSDGHEFIVKREHALTSGTIKAMLSGPGQFAENETNEVNFREIPSHVLSKVCMYFTYKVRYTNSSTEIPEFPIAPEIA
Splooce       MDGEEKTYGGCEGPDAMYVKLISSDGHEFIVKREHALTSGTIKAMLSG-RQFAENETNEVNFREIPSHVLSKVCMYFTYKVRYTNSSTEIPEFPIAPEIA

Uniprot       LELLMAANFLDC
Splooce       LELLMAANFLDC

----------------------------------------------------------------------------------------------------

P62701 (Uniprot)	versus
NM_001007#(-t:X_R6936211718679) (Splooce)

For more details about the Alternative Splicing Event -> Link to Splooce page

Peptides that support the ASE (Splooce-specific):
TDITYPAGFMALTRRER (MAXQUANT)

Alignment:
Uniprot       MARGPKKHLKRVAAPKHWMLDKLTGVFAPRPSTGPHKLRECLPLIIFLRNRLKYALTGDEVKKICMQRFIKIDGKVRTDITYPAGFMDVISIDKTGENFR
Splooce       MARGPKKHLKRVAAPKHWMLDKLTGVFAPRPSTGPHKLRECLPLIIFLRNRLKYALTGDEVKKICMQRFIKIDGKVRTDITYPAGFMALT----------

Uniprot       LIYDTKGRFAVHRITPEEAKYKLCKVRKIFVGTKGIPHLVTHDARTIRYPDPLIKVNDTIQIDLETGKITDFIKFDTGNLCMVTGGANLGRIGVITNRER
Splooce       ----RRERISV-----------------------------------------------------------------------------------------

Uniprot       HPGSFDVVHVKDANGNSFATRLSNIFVIGKGNKPWISLPRGKGIRLTIAEERDKRLAAKQSSG
Splooce       ---------------------------------------------------------------

----------------------------------------------------------------------------------------------------

Q9HCE1 (Uniprot)	versus
NM_020963#(f-T:1_M2379879444122) (Splooce)

For more details about the Alternative Splicing Event -> Link to Splooce page

Peptides that support the ASE (Splooce-specific):
RRGGGDR (MAXQUANT)

Alignment:
Uniprot       MPSKFSCRQLREAGQCFES=FLVVRGLDMETDRERL------------------------------------RTIYN------RDFKISFGTPAPGFSSM
Splooce       MRRGGGDRTKKPKGKLRAANFQLQRRLSVSFPRTLLPGPQPPPRCPVSSAAGSSGRRASVSRVSWSFGDWTWRQIASGCGPFITATSSSFGTPAPGFSSM

Uniprot       LYGMKIANLAYVTKTRVRFFRLDRWADVRFPEKRRMKLGSDISKHHKSLLAKIFYDRAEYLHGKHGVDVEVQGPHEARDGQLLIRLDLNRKEVLTLRLRN
Splooce       LYGMKIANLAYVTKTRVRFFRLDRWADVRFPEKRRMKLGSDISKHHKSLLAKIFYDRAEYLHGKHGVDVEVQGPHEARDGQLLIRLDLNRKEVLTLRLRN

Uniprot       GGTQSVTLTHLFPLCRTPQFAFYNEDQELPCPLGPGECYELHVHCKTSFVGYFPATVLWELLGPGESGSEGAGTFYIARFLAAVAHSPLAAQLKPMTPFK
Splooce       GGTQSVTLTHLFPLCRTPQFAFYNEDQELPCPLGPGECYELHVHCKTSFVGYFPATVLWELLGPGESGSEGAGTFYIARFLAAVAHSPLAAQLKPMTPFK

Uniprot       RTRITGNPVVTNRIEEGERPDRAKGYDLELSMALGTYYPPPRLRQLLPMLLQGTSIFTAPKEIAEIKAQLETALKWRNYEVKLRLLLHLEELQMEHDIRH
Splooce       RTRITGNPVVTNRIEEGERPDRAKGYDLELSMALGTYYPPPRLRQLLPMLLQGTSIFTAPKEIAEIKAQLETALKWRNYEVKLRLLLHLEELQMEHDIRH

Uniprot       YDLESVPMTWDPVDQNPRLLTLEVPGVTESRPSVLRGDHLFALLSSETHQEDPITYKGFVHKVELDRVKLSFSMSLLSRFVDGLTFKVNFTFNRQPLRVQ
Splooce       YDLESVPMTWDPVDQNPRLLTLEVPGVTESRPSVLRGDHLFALLSSETHQEDPITYKGFVHKVELDRVKLSFSMSLLSRFVDGLTFKVNFTFNRQPLRVQ

Uniprot       HRALELTGRWLLWPMLFPVAPRDVPLLPSDVKLKLYDRSLESNPEQLQAMRHIVTGTTRPAPYIIFGPPGTGKTVTLVEAIKQVVKHLPKAHILACAPSN
Splooce       HRALELTGRWLLWPMLFPVAPRDVPLLPSDVKLKLYDRSLESNPEQLQAMRHIVTGTTRPAPYIIFGPPGTGKTVTLVEAIKQVVKHLPKAHILACAPSN

Uniprot       SGADLLCQRLRVHLPSSIYRLLAPSRDIRMVPEDIKPCCNWDAKKGEYVFPAKKKLQEYRVLITTLITAGRLVSAQFPIDHFTHIFIDEAGHCMEPESLV
Splooce       SGADLLCQRLRVHLPSSIYRLLAPSRDIRMVPEDIKPCCNWDAKKGEYVFPAKKKLQEYRVLITTLITAGRLVSAQFPIDHFTHIFIDEAGHCMEPESLV

Uniprot       AIAGLMEVKETGDPGGQLVLAGDPRQLGPVLRSPLTQKHGLGYSLLERLLTYNSLYKKGPDGYDPQFITKLLRNYRSHPTILDIPNQLYYEGELQACADV
Splooce       AIAGLMEVKETGDPGGQLVLAGDPRQLGPVLRSPLTQKHGLGYSLLERLLTYNSLYKKGPDGYDPQFITKLLRNYRSHPTILDIPNQLYYEGELQACADV

Uniprot       VDRERFCRWAGLPRQGFPIIFHGVMGKDEREGNSPSFFNPEEAATVTSYLKLLLAPSSKKGKARLSPRSVGVISPYRKQVEKIRYCITKLDRELRGLDDI
Splooce       VDRERFCRWAGLPRQGFPIIFHGVMGKDEREGNSPSFFNPEEAATVTSYLKLLLAPSSKKGKARLSPRSVGVISPYRKQVEKIRYCITKLDRELRGLDDI

Uniprot       KDLKVGSVEEFQGQERSVILISTVRSSQSFVQLDLDFNLGFLKNPKRFNVAVTRAKALLIIVGNPLLLGHDPDWKVFLEFCKENGGYTGCPFPAKLDLQQ
Splooce       KDLKVGSVEEFQGQERSVILISTVRSSQSFVQLDLDFNLGFLKNPKRFNVAVTRAKALLIIVGNPLLLGHDPDWKVFLEFCKENGGYTGCPFPAKLDLQQ

Uniprot       GQNLLQGLSKLSPSTSGPHSHDYLPQEREGEGGLSLQVEPEWRNEL
Splooce       GQNLLQGLSKLSPSTSGPHSHDYLPQEREGEGGLSLQVEPEWRNEL

----------------------------------------------------------------------------------------------------

Q9UK76 (Uniprot)	versus
NM_016185#(-t:17_H9382567923623) (Splooce)

For more details about the Alternative Splicing Event -> Link to Splooce page

Peptides that support the ASE (Splooce-specific):
GEGDIHECGHR (MAXQUANT)

Alignment:
Uniprot       MTTTTTFKGVDPNSRNSSRVLRPPGGGSNFSLGFDEPTEQPVRKNKMASNIFGTPEENQASWAKSAGAKSSGGREDLESSGLQRRNSSEASSGDFLDLKG
Splooce       MTTTTTFKGVDPNSRNSSRVLRPPGGGSNFSLGFDEPTEQPVRKNKMASNIFGTPEENQASWAKSAGAKSSGGREDLESSGLQRRNSSEASSGDFLDLKG

Uniprot       EGDIHENVDTDLPGSLGQSEEKPVPAAPVPSPVAPAPVPSRRNPPGGKSSLVLG
Splooce       EGDIHE---------CGHR-------------------LARQ--PGAE------

----------------------------------------------------------------------------------------------------

P62937 (Uniprot)	versus
NM_021130#(f-t:7_P716254529325) (Splooce)

For more details about the Alternative Splicing Event -> Link to Splooce page

Peptides that support the ASE (Splooce-specific):
FEDENFILKHTGPGILSMAN (MAXQUANT)
HTGPGILSMAN (MAXQUANT)

Alignment:
Uniprot       MVNPTVFFDIAVDGEPLGRVSFELFADKVPKTAENFRALSTGEKGFGYKGSCFHRIIPGFMCQGGDFTRHNGTGGKSIYGEKFEDENFILKHTGPGILSM
Splooce       MVNPTVFFDIAVDGEPLGRVSFELFADKVPKTAENFRALSTGEKGFGYKGSCFHRIIPGFMCQGGDFTRHNGTGGKSIYGEKFEDENFILKHTGPGILSM

Uniprot       ANAGPNTNGSQFFICTAKTEWLDGKHVVFGKVKEGMNIVEAMERFGSRNGKTSKKITIADCGQLE
Splooce       AN---------------------------------------------------------------

----------------------------------------------------------------------------------------------------

P02792 (Uniprot)	versus
NM_000146#(-s-:19_F4257270421792) (Splooce)

For more details about the Alternative Splicing Event -> Link to Splooce page

Peptides that support the ASE (Splooce-specific):
LCDFLETHFLDEEVK (MAXQUANT)

Alignment:
Uniprot       MSSQIRQNYSTDVEAAVNSLVNLYLQASYTYLSLGFYFDRDDVALEGVSHFFRELAEEKREGYERLLKMQNQRGGRALFQDIKKPAEDEWGKTPDAMKAA
Splooce       MSSQIRQNYSTDVEAAVNSLVNLYLQASYTYLSLGFYFDRDDVALEGVSHFFRELAEEKREGYERLLKMQNQRGGRALFQDIK-----------------

Uniprot       MALEKKLNQALLDLHALGSARTDPHLCDFLETHFLDEEVKLIKKMGDHLTNLHRLGGPEAGLGEYLFERLTLKHD
Splooce       -------------------------LCDFLETHFLDEEVKLIKKMGDHLTNLHRLGGPEAGLGEYLFERLTLKHD

----------------------------------------------------------------------------------------------------

P60981 (Uniprot)	versus
NM_001011546#(-t:20_D2477389546106) (Splooce)

For more details about the Alternative Splicing Event -> Link to Splooce page

Peptides that support the ASE (Splooce-specific):
KEELMFFL (MAXQUANT)

Alignment:
Uniprot       MKVRKCSTPEEIKKRKKAVIFCLSADKKCIIVEEGKEILVGDVGVTITDPFKHFVGMLPEKDCRYALYDASFETKESRKEELMFFLWAPELAPLKSKMIY
Splooce       MKVRKCSTPEEIKKRKKAVIFCLSADKKCIIVEEGKEILVGDVGVTITDPFKHFVGMLPEKDCRYALYDASFETKESRKEELMFFL--------------

Uniprot       ASSKDAIKKKFQGIKHECQANGPEDLNRACIAEKLGGSLIVAFEGCPV
Splooce       ------------------------------------------------

----------------------------------------------------------------------------------------------------

Q8NC60 (Uniprot)	versus
NM_032313#(-s-:4_C7021294714403) (Splooce)

For more details about the Alternative Splicing Event -> Link to Splooce page

Peptides that support the ASE (Splooce-specific):
IDFLQIPMGGK (MAXQUANT)

Alignment:
Uniprot       MLPARLPFRLLSLFLRGSAPTAARHGLREPLLERRCAAASSFQHSSSLGRELPYDPVDTEGFGEGGDMQERFLFPEYILDPEPQPTREKQLQELQQQQEE
Splooce       MLPARLPFRLLSLFLRGSAPTAARHGLREPLLERRCAAASSFQHSSSLGRELPYDPVDTEGFGEGGDMQERFLFPEYILDPEPQPTREKQLQELQQQQEE

Uniprot       EERQRQQRREERRQQNLRARSREHPVVGHPDPALPPSGVNCSGCGAELHCQDAGVPGYLPREKFLRTAEADGGLARTVCQRCWLLSHHRRALRLQVSREQ
Splooce       EERQRQQRREERRQQNLRARSREHPVVGHPDPALPPSGVNCSGCGAELHCQDAGVPGYLPREKFLRTAEADGGLARTVCQRCWLLSHHRRALRLQVSREQ

Uniprot       YLELVSAALRRPGPSLVLYMVDLLDLPDALLPDLPALVGPKQLIVLGNKVDLLPQDAPGYRQRLRERLWEDCARAGLLLAPGHQGPQRPVKDEPQDGENP
Splooce       YLELVSAALRRPGPSLVLYMVDLLDLPDALLPDLPALVGPKQLIVLGNKVDLLPQDAPGYRQRLRERLWEDCARAGLLLAPGHQGPQRPVKDEPQDGENP

Uniprot       NPPNWSRTVVRDVRLISAKTGYGVEELISALQRSWRYRGDVYLVGATNAGKSTLFNTLLESDYCTAKGSEAIDRATISPWPGTTLNLLKFPICNPTPYRM
Splooce       NPPNWSRTVVRDVRLISAKTGYGVEELISALQRSWRYRGDVYLVGATNAGKSTLFNTLLESDYCTAKGSEAIDRATISPWPGTTLNLLKFPICNPTPYRM

Uniprot       FKRHQRLKKDSTQAEEDLSEQEQNQLNVLKKHGYVVGRVGRTFLYSEEQKDNIPFEFDADSLAFDMENDPVMGTHKSTKQVELTAQDVKDAHWFYDTPGI
Splooce       FKRHQRLKKDSTQAEEDLSEQEQNQLNVLKKHGYVVGRVGRTFLYSEEQKDNIPFEFDADSLAFDMENDPVMGTHKSTKQVELTAQDVKDAHWFYDTPGI

Uniprot       TKENCILNLLTEKEVNIVLPTQSIVPRTFVLKPGMVLFLGAIGRIDFLQGNQSAWFTVVASNILPVHITSLDRADALYQKHAGHTLLQIPMGGKERMAGF
Splooce       TKENCILNLLTEKEVNIVLPTQSIVPRTFVLKPGMVLFLGAIGRIDFLQ---------------------------------------IPMGGKERMAGF

Uniprot       PPLVAEDIMLKEGLGASEAVADIKFSSAGWVSVTPNFKDRLHLRGYTPEGTVLTVRPPLLPYIVNIKGQRIKKSVAYKTKKPPSLMYNVRKKKGKINV
Splooce       PPLVAEDIMLKEGLGASEAVADIKFSSAGWVSVTPNFKDRLHLRGYTPEGTVLTVRPPLLPYIVNIKGQRIKKSVAYKTKKPPSLMYNVRKKKGKINV

----------------------------------------------------------------------------------------------------

P10809 (Uniprot)	versus
NM_199440#(-s-s-s-s-s-s-s-s-s-s-:2_H6756246207470) (Splooce)

For more details about the Alternative Splicing Event -> Link to Splooce page

Peptides that support the ASE (Splooce-specific):
HLYIPDTGYK (MAXQUANT)

Alignment:
Uniprot       MLRLPTVFRQMRPVSRVLAPHLTRAYAKDVKFGADARALMLQGVDLLADAVAVTMGPKGRTVIIEQSWGSPKVTKDGVTVAKSIDLKDKYKNIGAKLVQD
Splooce       ----------------------------------------------------------------------------------------------------

Uniprot       VANNTNEEAGDGTTTATVLARSIAKEGFEKISKGANPVEIRRGVMLAVDAVIAELKKQSKPVTTPEEIAQVATISANGDKEIGNIISDAMKKVGRKGVIT
Splooce       ----------------------------------------------------------------------------------------------------

Uniprot       VKDGKTLNDELEIIEGMKFDRGYISPYFINTSKGQKCEFQDAYVLLSEKKISSIQSIVPALEIANAHRKPLVIIAEDVDGEALSTLVLNRLKVGLQVVAV
Splooce       ------------------------MVYCCHCP----CLQIIYFVFLNKKHL-----YIP----DTGYKSHVPVYCFQLK--SLRHFYYYSVKIRILVLAT

Uniprot       KAPGFGDNRKNQLKDMAIATGGAVFGEEGLTLNLEDVQPHDLGKVGEVIVTKDDAMLLKGKGDKAQIEKRIQEIIEQLDVTTSEYEKEKLNERLAKLSDG
Splooce       TR--------------------------------------------------------------------------------------------------

Uniprot       VAVLKVGGTSDVEVNEKKDRVTDALNATRAAVEEGIVLGGGCALLRCIPALDSLTPANEDQKIGIEIIKRTLKIPAMTIAKNAGVEGSLIVEKIMQSSSE
Splooce       ----------------------------------------------------------------------------------------------------

Uniprot       VGYDAMAGDFVNMVEKGIIDPTKVVRTALLDAAGVASLLTTAEVVVTEIPKEEKDPGMGAMGGMGGGMGGGMF
Splooce       -------------------------------------------------------------------------

----------------------------------------------------------------------------------------------------

Q9UHD8 (Uniprot)	versus
NM_006640#(-s-s-s-s-:17_S6444259730375) (Splooce)

For more details about the Alternative Splicing Event -> Link to Splooce page

Peptides that support the ASE (Splooce-specific):
SQEASGPWTSSL (MAXQUANT)

Alignment:
Uniprot       MERDRISALKRSFEVEEVETPNSTPPRRVQTPLLRATVASSTQKFQDLGVKNSEPSARHVDSLSQRSPKASLRRVELSGPKAAEPVSRRTELSIDISSKQ
Splooce       MERDRISALKRSFEVEEVETPNSTPPRRVQTPLLRATVASSTQKFQDLGVKNSEPSARHVDSLSQRSPKASLRRVELSGPKAAEPVSRRTELSIDISSKQ

Uniprot       VENAGAIGPSRFGLKRAEVLGHKTPEPAPRRTEITIVKPQESAHRRMEPPASKVPEVPTAPATDAAPKRVEIQMPKPAEAPTAPSPAQTLENSEPAPVSQ
Splooce       VENAGAIGPSRFGLKRAEVLGHKTPEPAPRRTEITIVKPQESAHRRMEPPASKVPEVPTAPATDAAPKRVEIQMPKPAEAPTAPSPAQTLENSEPAPVSQ

Uniprot       LQSRLEPKPQPPVAEATPRSQEATEAAPSCVGDMADTPRDAGLKQAPASRNEKAPVDFGYVGIDSILEQMRRKAMKQGFEFNIMVVGQSGLGKSTLINTL
Splooce       LQSRLEPKPQPPVAEATPRSQEASGPWTSSL---------------------------------------------------------------------

Uniprot       FKSKISRKSVQPTSEERIPKTIEIKSITHDIEEKGVRMKLTVIDTPGFGDHINNENCWQPIMKFINDQYEKYLQEEVNINRKKRIPDTRVHCCLYFIPAT
Splooce       ----------------------------------------------------------------------------------------------------

Uniprot       GHSLRPLDIEFMKRLSKVVNIVPVIAKADTLTLEERVHFKQRITADLLSNGIDVYPQKEFDEDSEDRLVNEKFREMIPFAVVGSDHEYQVNGKRILGRKT
Splooce       ----------------------------------------------------------------------------------------------------

Uniprot       KWGTIEVENTTHCEFAYLRDLLIRTHMQNIKDITSSIHFEAYRVKRLNEGSSAMANGMEEKEPEAPEM
Splooce       --------------------------------------------------------------------

----------------------------------------------------------------------------------------------------

Q8TBC4 (Uniprot)	versus
NM_198195#(-s-s-s-s-s-:3_U3139928788072) (Splooce)

For more details about the Alternative Splicing Event -> Link to Splooce page

Peptides that support the ASE (Splooce-specific):
NHSCSSFHKCSHCSCVCH (MAXQUANT)

Alignment:
Uniprot       MADGEEPMAVDGGCGDTGDWEGRWNHVKKFLERSGPFTHPDFEPSTESLQFLLDTCKVLVIGAGGLGCELLKNLALSGFRQIHVIDMDTIDVSNLNRQFL
Splooce       MADGEEPMAVDGGCGDTGDWEGRWNHVKKFLERSGPFTHPDFEPSTESLQFLLDTCKVLVIGAGGLGCELLKNLALSGFRQIHVIDMDTIDVSNLNRQFL

Uniprot       FRPKDIGRPKAEVAAEFLNDRVPNCNVVPHFNKIQDFNDTFYRQFHIIVCGLDSIIARRWINGMLISLLNYEDGVLDPSSIVPLIDGGTEGFKGNARVIL
Splooce       FRPKDIGRPKAEVAAEFLNDRVPNCNVVPGSKKNHSCSSFHKCSHCSCVCH-------------------------------------------------

Uniprot       PGMTACIECTLELYPPQVNFPMCTIASMPRLPEHCIEYVRMLQWPKEQPFGEGVPLDGDDPEHIQWIFQKSLERASQYNIRGVTYRLTQGVVKRIIPAVA
Splooce       ----------------------------------------------------------------------------------------------------

Uniprot       STNAVIAAVCATEVFKIATSAYIPLNNYLVFNDVDGLYTYTFEAERKENCPACSQLPQNIQFSPSAKLQEVLDYLTNSASLQMKSPAITATLEGKNRTLY
Splooce       ----------------------------------------------------------------------------------------------------

Uniprot       LQSVTSIEERTRPNLSKTLKELGLVDGQELAVADVTTPQTVLFKLHFTS
Splooce       -------------------------------------------------

----------------------------------------------------------------------------------------------------

P49368 (Uniprot)	versus
NM_001008800#(-t:1_C7339192033172) (Splooce)

For more details about the Alternative Splicing Event -> Link to Splooce page

Peptides that support the ASE (Splooce-specific):
EILSNATSR (MAXQUANT)

Alignment:
Uniprot       MMGHRPVLVLSQNTKRESGRKVQSGNINAAKIQVQHPAAKSMIEISRTQDEEVGDGTTSVIILAGEMLSVAEHFLEQQMHPTVVISAYRKALDDMISTLK
Splooce       MMGHRPVLVLSQNTKRESGRKVQSGNINAAKIQVQHPAAKSMIEISRTQDEEVGDGTTSVIILAGEMLSVAEHFLEQQMHPTVVISAYRKALDDMISTLK

Uniprot       KISIPVDISDSDMMLNIINSSITTKAISRWSSLACNIALDAVKMVQFEENGRKEIDIKKYARVEKIPGGIIEDSCVLRGVMINKDVTHPRMRRYIKNPRI
Splooce       KISIPVDISDSDMMLNIINSSITTKAISRWSSLACNIALDAVKMVQFEENGRKEIDIKKYARVEKIPGGIIEDSCVLRGVMINKDVTHPRMRRYIKNPRI

Uniprot       VLLDSSLEYKKGESQTDIEITREEDFTRILQMEEEYIQQLCEDIIQLKPDVVITEKGISDLAQHYLMRANITAIRRVRKTDNNRIARACGARIVSRPEEL
Splooce       VLLDSSLEYKKGESQTDIEITREEDFTRILQMEEEYIQQLCEDIIQLKPDVVITEKGISDLAQHYLMRANITAIRRVRKTDNNRIARACGARIVSRPEEL

Uniprot       REDDVGTGAGLLEIKKIGDEYFTFITDCKDPKACTILLRGASKEILSEVERNLQDAMQVCRNVLLDPQLVPGGGASEMAVAHALTEKSKAMTGVEQWPYR
Splooce       REDDVGTGAGLLEIKKIGDEYFTFITDCKDPKACTILLRGASKEILSNATS------------------------------------------------R

Uniprot       AVAQALEVIPRTLIQNCGASTIRLLTSLRAKHTQENCETWGVNGETGTLVDMKELGIWEPLAVKLQTYKTAVETAVLLLRIDDIVSGHKKKGDDQSRQGG
Splooce       MPCKCVAMFSWTLSWCQGVGPPRWLWPMP-----------------------------------------------------------------------

Uniprot       APDAGQE
Splooce       -------

----------------------------------------------------------------------------------------------------

P12429 (Uniprot)	versus
NM_005139#(f-T:4_A2469356082138) (Splooce)

For more details about the Alternative Splicing Event -> Link to Splooce page

Peptides that support the ASE (Splooce-specific):
ALLTLADVR (MAXQUANT)

Alignment:
Uniprot       MASIWVGHRGTVRDYPDFSPSVDAEAIQKAIRGIGTDEKMLISILTERSNAQRQLIVKEYQAAYGKELKDDLKGDLSGHFEHLMVALVTPPAVFDAKQLK
Splooce       MASIWVGHRGTVRDYPDFSPSVDAEAIQKAIRGIGTDEKMLISILTERSNAQRQLIVKEYQAAYGKELKDDLKGDLSGHFEHLMVALVTPPAVFDAKQLK

Uniprot       KSMKGAGTNEDALIEILTTRTSRQMKDISQAYYTVYKKSLGDDISSETSGDFRKALLTLADGRRDESLKVDEHLAKQDAQILYKAGENRWGTDEDKFTEI
Splooce       KSMKGAGTNEDALIEILTTRTSRQMKDISQAYYTVYKKSLGDDISSETSGDFRKALLTLADVRRDESLKVDEHLAKQDAQILYKAGENRWGTDEDKFTEI

Uniprot       LCLRSFPQLKLTFDEYRNISQKDIVDSIKGELSGHFEDLLLAIVNCVRNTPAFLAERLHRALKGIGTDEFTLNRIMVSRSEIDLLDIRTEFKKHYGYSLY
Splooce       LCLRSFPQLKLTFDEYRNISQKDIVDSIKGELSGHFEDLLLAIVNCVRNTPAFLAERLHRALKGIGTDEFTLNRIMVSRSEIDLLDIRTEFKKHYGYSLY

Uniprot       SAIKSDTSGDYEITLLKICGGDD
Splooce       SAIKSDTSGDYEITLLKICGGDD

----------------------------------------------------------------------------------------------------

Q9UFC0 (Uniprot)	versus
NM_152892#(-s-s-s-s-:7_L3295083087941) (Splooce)

For more details about the Alternative Splicing Event -> Link to Splooce page

Peptides that support the ASE (Splooce-specific):
APGEPPR (MAXQUANT)

Alignment:
Uniprot       MGPLSARLLMQRGRPKSDRLGKIRSLDLSGLELLSEHLDPKLLCRLTQLQELDLSNNHLETLPDNLGLSHLRVLRCANNQLGDVTALCQFPKLEELSLEG
Splooce       MGPLSARLLMQRGRPKSDRLGKIRSLDLSGLELLSEHLDPKLLCRLTQLQELDLSNNHLETLPDNLGLSHLRVLRCANNQLGDVTALCQFPKLEELSLEG

Uniprot       NPFLTVNDNLKVSFLLPTLRKVNGKDASSTYSQVENLNRELTSRVTAHWEKFMATLGPEEEAEKAQADFVKSAVRDVRYGPESLSEFTQWRVRMISEELV
Splooce       NPFLTVNDNLKVSFLLPTLRKVNGKDASSTYSQVENLNRELTSRVTAHWEKFMATLGPEEEAEKAQADFVKSAVRDVRYGPESLSEFTQWRVRMISEELV

Uniprot       AASRTQVQKANSPEKPPEAGAAHKPRARLAALKRPDDVPLSLSPSKRACASPSAQVEGSPVAGSDGSQPAVKLEPLHFLQCHSKNNSPQDLETQLWACAF
Splooce       AASRTQVQKANSPEKPPEAGAAHKPRARLAALKRPDDVPLSLSPSKRACASPSAQVEGSPVAGSDGSQPAVKLEPLHFLQCHSKNNSPQDLETQLWACAF

Uniprot       EPAWEEGATSQTVATCGGEAVCVIDCQTGIVLHKYKAPGEEFFSVAWTALMVVTQAGHKKRWSVLAAAGLRGLVRLLHVRAGFCCGVIRAHKKAIATLCF
Splooce       EPAWEEGATSQTVATCGGEAVCVIDCQTGIVLHKYKAPGEPPRGAAWAP-------------SACGAGGRRG----------------------------

Uniprot       SPAHETHLFTASYDKRIILWDIGVPNQDYEFQASQLLTLDTTSIPLRLCPVASCPDARLLAGCEGGCCCWDVRLDQPQKRRVCEVEFVFSEGSEASGRRV
Splooce       -------------------------------------------------------------------------------------------GAGAASPRW

Uniprot       DGLAFVNEDIVASKGSGLGTICLWSWRQTWGGRGSQSTVAVVVLARLQWSSTELAYFSLSACPDKGIVLCGDEEGNVWLYDVSNILKQPPLLPAALQAPT
Splooce       Q---------------------WWSWRGCNG--------------------------------------------------------RPPSWPTSRSAP-

Uniprot       QILKWPQPWALGQVVTKTMVNTVVANASFTYLTALTDSNIVAIWGRM
Splooce       -------------ALIRGLCSVGMRRATCGSTTSATS----------

----------------------------------------------------------------------------------------------------

P41252 (Uniprot)	versus
NM_002161#(-s-s-s-s-s-s-s-s-s-:9_I5112559152607) (Splooce)

For more details about the Alternative Splicing Event -> Link to Splooce page

Peptides that support the ASE (Splooce-specific):
WILSFMQSLIGFFETEMAVEGI (MAXQUANT)

Alignment:
Uniprot       MLQQVPENINFPAEEEKILEFWTEFNCFQECLKQSKHKPKFTFYDGPPFATGLPHYGHILAGTIKDIVTRYAHQSGFHVDRRFGWDCHGLPVEYEIDKTL
Splooce       MLQQVPENINFPAEEEKILEFWTEFNCFQECLKQSKHKPKFTFYDGPPFATGLPHYGHILAGTIKDIVTRYAHQSGFHVDRRFGWDCHGLPVEYEIDKTL

Uniprot       GIRGPEDVAKMGITEYNNQCRAIVMRYSAEWKSTVSRLGRWIDFDNDYKTLYPQFMESVWWVFKQLYDKGLVYRGVKVMPFSTACNTPLSNFESHQNYKD
Splooce       GIRGPEDVAKMGITEYNNQCRAIVMRYSAEWKSTVSRLGRWIDFDNDYKTLYPQFMESVWWVFKQLYDKGLVYRGVKVMPFSTACNTPLSNFESHQNYKD

Uniprot       VQDPSVFVTFPLEEDETVSLVAWTTTPWTLPSNLAVCVNPEMQYVKIKDVARGRLLILMEARLSALYKLESDYEILERFPGAYLKGKKYRPLFDYFLKCK
Splooce       VQDPSVFVTFPLEEDETVSLVAWTTTPWTLPSNLAVCVNPEMQYVKIKDVARGRLLILMEARLSALYKLESDYEILERFPGAYLKGKKYRPLFDYFLKCK

Uniprot       ENGAFTVLVDNYVKEEEGTGVVHQAPYFGAEDYRVCMDFNIIRKDSLPVCPVDASGCFTTEVTDFAGQYVKDADKSIIRTLKEQGRLLVATTFTHSYPFC
Splooce       ENGAFTVLVDNYVKEEEGTGVVHQAPYFGAEDYRVCMDFNIIRKDSLPVCPVDASGCFTTEVTDFAGQYVKDADKSIIRTLKEQGRLLVATTFTHSYPFC

Uniprot       WRSDTPLIYKAVPSWFVRVENMVDQLLRNNDLCYWVPELVREKRFGNWLKDARDWTISRNRYWGTPIPLWVSDDFEEVVCIGSVAELEELSGAKISDLHR
Splooce       WRSDTPLIYKAVPSWFVRVENMVDQLLRNNDLCYWVPELVREKRFGNWLKDARDWTISRNRYWGTPIPLWVSDDFEEVVCIGSVAELEELSGAKISDLHR

Uniprot       ESVDHLTIPSRCGKGSLHRISEVFDCWFESGSMPYAQVHYPFENKREFEDAFPADFIAEGIDQTRGWFYTLLVLATALFGQPPFKNVIVNGLVLASDGQK
Splooce       ESVDHLTIPSRCGKGSLHRISEVFDCWFESGSMPYAQVHYPFENKREFEDAFPADFIAEGIDQTRGWFYTLLVLATALFGQPPFKNVIVNGLVLASDGQK

Uniprot       MSKRKKNYPDPVSIIQKYGADALRLYLINSPVVRAENLRFKEEGVRDVLKDVLLPWYNAYRFLIQNVLRLQKEEEIEFLYNENTVRESPNITDRWILSFM
Splooce       MSKRKKNYPDPVSIIQKYGADALRLYLINSPVVRAENLRFKEEGVRDVLKDVLLPWYNAYRFLIQNVLRLQKEEEIEFLYNENTVRESPNITDRWILSFM

Uniprot       QSLIGFFETEMAAYRLYTVVPRLVKFVDILTNWYVRMNRRRLKGENGMEDCVMALETLFSVLLSLCRLMAPYTPFLTELMYQNLKVLIDPVSVQDKDTLS
Splooce       QSLIGFFETEMAVEGI------------------------------------------------------------------------------------

Uniprot       IHYLMLPRVREELIDKKTESAVSQMQSVIELGRVIRDRKTIPIKYPLKEIVVIHQDPEALKDIKSLEKYIIEELNVRKVTLSTDKNKYGIRLRAEPDHMV
Splooce       ----------------------------------------------------------------------------------------------------

Uniprot       LGKRLKGAFKAVMTSIKQLSSEELEQFQKTGTIVVEGHELHDEDIRLMYTFDQATGGTAQFEAHSDAQALVLLDVTPDQSMVDEGMAREVINRIQKLRKK
Splooce       ----------------------------------------------------------------------------------------------------

Uniprot       CNLVPTDEITVYYKAKSEGTYLNSVIESHTEFIFTTIKAPLKPYPVSPSDKVLIQEKTQLKGSELEITLTRGSSLPGPACAYVNLNICANGSEQGGVLLL
Splooce       ----------------------------------------------------------------------------------------------------

Uniprot       ENPKGDNRLDLLKLKSVVTSIFGVKNTELAVFHDETEIQNQTDLLSLSGKTLCVTAGSAPSLINSSSTLLCQYINLQLLNAKPQECLMGTVGTLLLENPL
Splooce       ----------------------------------------------------------------------------------------------------

Uniprot       GQNGLTHQGLLYEAAKVFGLRSRKLKLFLNETQTQEITEDIPVKTLNMKTVYVSVLPTTADF
Splooce       --------------------------------------------------------------

----------------------------------------------------------------------------------------------------

O75533 (Uniprot)	versus
NM_012433#(-s-s-s-s-:2_S1102831635814) (Splooce)

For more details about the Alternative Splicing Event -> Link to Splooce page

Peptides that support the ASE (Splooce-specific):
EEVLPPPAGYVPIR (MAXQUANT)

Alignment:
Uniprot       MAKIAKTHEDIEAQIREIQGKKAALDEAQGVGLDSTGYYDQEIYGGSDSRFAGYVTSIAATELEDDDDDYSSSTSLLGQKKPGYHAPVALLNDIPQSTEQ
Splooce       MAKIAKTHEDIEAQIREIQGKKAALDEAQGVGLDSTGYYDQEIYGGSDSRFAGYVTSIAATELEDDDDDYSSSTSLLGQKKPGYHAPVALLNDIPQSTEQ

Uniprot       YDPFAEHRPPKIADREDEYKKHRRTMIISPERLDPFADGGKTPDPKMNARTYMDVMREQHLTKEEREIRQQLAEKAKAGELKVVNGAAASQPPSKRKRRW
Splooce       YDPFAEHRPPKIADREDEYKKHRRTMIISPERLDPFADGGKTPDPKMNARTYMDVMREQHLTKEE-----------------------------------

Uniprot       DQTADQTPGATPKKLSSWDQAETPGHTPSLRWDETPGRAKGSETPGATPGSKIWDPTPSHTPAGAATPGRGDTPGHATPGHGGATSSARKNRWDETPKTE
Splooce       ----------------------------------------------------------------------------------------------------

Uniprot       RDTPGHGSGWAETPRTDRGGDSIGETPTPGASKRKSRWDETPASQMGGSTPVLTPGKTPIGTPAMNMATPTPGHIMSMTPEQLQAWRWEREIDERNRPLS
Splooce       ----------------------------------------------------------------------------------------------------

Uniprot       DEELDAMFPEGYKVLPPPAGYVPIRTPARKLTATPTPLGGMTGFHMQTEDRTMKSVNDQPSGNLPFLKPDDIQYFDKLLVDVDESTLSPEEQKERKIMKL
Splooce       -------------VLPPPAGYVPIRTPARKLTATPTPLGGMTGFHMQTEDRTMKSVNDQPSGNLPFLKPDDIQYFDKLLVDVDESTLSPEEQKERKIMKL

Uniprot       LLKIKNGTPPMRKAALRQITDKAREFGAGPLFNQILPLLMSPTLEDQERHLLVKVIDRILYKLDDLVRPYVHKILVVIEPLLIDEDYYARVEGREIISNL
Splooce       LLKIKNGTPPMRKAALRQITDKAREFGAGPLFNQILPLLMSPTLEDQERHLLVKVIDRILYKLDDLVRPYVHKILVVIEPLLIDEDYYARVEGREIISNL

Uniprot       AKAAGLATMISTMRPDIDNMDEYVRNTTARAFAVVASALGIPSLLPFLKAVCKSKKSWQARHTGIKIVQQIAILMGCAILPHLRSLVEIIEHGLVDEQQK
Splooce       AKAAGLATMISTMRPDIDNMDEYVRNTTARAFAVVASALGIPSLLPFLKAVCKSKKSWQARHTGIKIVQQIAILMGCAILPHLRSLVEIIEHGLVDEQQK

Uniprot       VRTISALAIAALAEAATPYGIESFDSVLKPLWKGIRQHRGKGLAAFLKAIGYLIPLMDAEYANYYTREVMLILIREFQSPDEEMKKIVLKVVKQCCGTDG
Splooce       VRTISALAIAALAEAATPYGIESFDSVLKPLWKGIRQHRGKGLAAFLKAIGYLIPLMDAEYANYYTREVMLILIREFQSPDEEMKKIVLKVVKQCCGTDG

Uniprot       VEANYIKTEILPPFFKHFWQHRMALDRRNYRQLVDTTVELANKVGAAEIISRIVDDLKDEAEQYRKMVMETIEKIMGNLGAADIDHKLEEQLIDGILYAF
Splooce       VEANYIKTEILPPFFKHFWQHRMALDRRNYRQLVDTTVELANKVGAAEIISRIVDDLKDEAEQYRKMVMETIEKIMGNLGAADIDHKLEEQLIDGILYAF

Uniprot       QEQTTEDSVMLNGFGTVVNALGKRVKPYLPQICGTVLWRLNNKSAKVRQQAADLISRTAVVMKTCQEEKLMGHLGVVLYEYLGEEYPEVLGSILGALKAI
Splooce       QEQTTEDSVMLNGFGTVVNALGKRVKPYLPQICGTVLWRLNNKSAKVRQQAADLISRTAVVMKTCQEEKLMGHLGVVLYEYLGEEYPEVLGSILGALKAI

Uniprot       VNVIGMHKMTPPIKDLLPRLTPILKNRHEKVQENCIDLVGRIADRGAEYVSAREWMRICFELLELLKAHKKAIRRATVNTFGYIAKAIGPHDVLATLLNN
Splooce       VNVIGMHKMTPPIKDLLPRLTPILKNRHEKVQENCIDLVGRIADRGAEYVSAREWMRICFELLELLKAHKKAIRRATVNTFGYIAKAIGPHDVLATLLNN

Uniprot       LKVQERQNRVCTTVAIAIVAETCSPFTVLPALMNEYRVPELNVQNGVLKSLSFLFEYIGEMGKDYIYAVTPLLEDALMDRDLVHRQTASAVVQHMSLGVY
Splooce       LKVQERQNRVCTTVAIAIVAETCSPFTVLPALMNEYRVPELNVQNGVLKSLSFLFEYIGEMGKDYIYAVTPLLEDALMDRDLVHRQTASAVVQHMSLGVY

Uniprot       GFGCEDSLNHLLNYVWPNVFETSPHVIQAVMGALEGLRVAIGPCRMLQYCLQGLFHPARKVRDVYWKIYNSIYIGSQDALIAHYPRIYNDDKNTYIRYEL
Splooce       GFGCEDSLNHLLNYVWPNVFETSPHVIQAVMGALEGLRVAIGPCRMLQYCLQGLFHPARKVRDVYWKIYNSIYIGSQDALIAHYPRIYNDDKNTYIRYEL

Uniprot       DYIL
Splooce       DYIL

----------------------------------------------------------------------------------------------------

Q5T9A4 (Uniprot)	versus
NM_031921#(-s-s-:1_A94012696409) (Splooce)

For more details about the Alternative Splicing Event -> Link to Splooce page

Peptides that support the ASE (Splooce-specific):
RGFMLVLASNLPEQFDCAINSR (MAXQUANT)

Alignment:
Uniprot       MSWLFGVNKGPKGEGAGPPPPLPPAQPGAEGGGDRGLGDRPAPKDKWSNFDPTGLERAAKAARELEHSRYAKEALNLAQMQEQTLQLEQQSKLKEYEAAV
Splooce       MSWLFGVNKGPKGEGAGPPPPLPPAQPGAEGGGDRGLGDRPAPKDKWSNFDPTGLERAAKAARELEHSRYAKEALNLAQMQEQTLQLEQQSKLKEYEAAV

Uniprot       EQLKSEQIRAQAEERRKTLSEETRQHQARAQYQDKLARQRYEDQLKQQQLLNEENLRKQEESVQKQEAMRRATVEREMELRHKNEMLRVETEARARAKAE
Splooce       EQLKSEQIRAQAEERRKTLSEETRQHQARAQYQDKLARQRYEDQLKQQQLLNEENLRKQEESVQKQEAMRRATVEREMELRHKNEMLRVETEARARAKAE

Uniprot       RENADIIREQIRLKASEHRQTVLESIRTAGTLFGEGFRAFVTDRDKVTATVAGLTLLAVGVYSAKNATAVTGRFIEARLGKPSLVRETSRITVLEALRHP
Splooce       RENADIIREQIRLKASEHRQTVLESIRTAGTLFGEGFRAFVTDRDKVTATVAGLTLLAVGVYSAKNATAVTGRFIEARLGKPSLVRETSRITVLEALRHP

Uniprot       IQVSRRLLSRPQDVLEGVVLSPSLEARVRDIAIATRNTKKNRGLYRHILLYGPPGTGKTLFAKKLALHSGMDYAIMTGGDVAPMGREGVTAMHKLFDWAN
Splooce       IQVSRRLLSRPQDVLEGVVLSPSLEARVRDIAIATRNTKKNRGLYRHILLYGPPGTGKTLFAKKLALHSGMDYAIMTGGDVAPMGREGVTAMHKLFDWAN

Uniprot       TSRRGLLLFMDEADAFLRKRATEEISKDLRATLNAFLYHMGQHSNKFMLVLASNLPEQFDCAINSRIDVMVHFDLPQQEERERLVRLHFDNCVLKPATEG
Splooce       TSRRG-----------------------------------------FMLVLASNLPEQFDCAINSRIDVMVHFDLPQQEERERLVRLHFDNCVLKPATEG

Uniprot       KRRLKLAQFDYGRKCSEVARLTEGMSGREIAQLAVSWQATAYASKDGVLTEAMMDACVQDAVQQYRQKMRWLKAEGPGRGVEHPLSGVQGETLTSWSLAT
Splooce       KRRLKLAQFDYGRKCSEVARLTEGMSGREIAQLAVSWQATAYASKDGVLTEAMMDACVQDAVQQYRQKMRWLKAEGPGRGVEHPLSGVQGETLTSWSLAT

Uniprot       DPSYPCLAGPCTFRICSWMGTGLCPGPLSPRMSCGGGRPFCPPGHPLL
Splooce       DPSYPCLAGPCTFRICSWMGTGLCPGPLSPRMSCGGGRPFCPPGHPLL

----------------------------------------------------------------------------------------------------

P04075 (Uniprot)	versus
NM_184043#(-s-:16_A8696064448678) (Splooce)

For more details about the Alternative Splicing Event -> Link to Splooce page

Peptides that support the ASE (Splooce-specific):
NGIVPIVEPEILPDGDHDLKR (MAXQUANT)
NGIVPIVEPEILPDGDHDLK (MAXQUANT)

Alignment:
Uniprot       MPYQYPALTPEQKKELSDIAHRIVAPGKGILAADESTGSIAKRLQSIGTENTEENRRFYRQLLLTADDRVNPCIGGVILFHETLYQKADDGRPFPQVIKS
Splooce       ----------------------------------------------------------------------------------------------------

Uniprot       KGGVVGIKVDKGVVPLAGTNGETTTQGLDGLSERCAQYKKDGADFAKWRCVLKIGEHTPSALAIMENANVLARYASICQQNGIVPIVEPEILPDGDHDLK
Splooce       ----------------------------------------------------------------------MARLP---PKNGIVPIVEPEILPDGDHDLK

Uniprot       RCQYVTEKVLAAVYKALSDHHIYLEGTLLKPNMVTPGHACTQKFSHEEIAMATVTALRRTVPPAVTGITFLSGGQSEEEASINLNAINKCPLLKPWALTF
Splooce       RCQYVTEKVLAAVYKALSDHHIYLEGTLLKPNMVTPGHACTQKFSHEEIAMATVTALRRTVPPAVTGITFLSGGQSEEEASINLNAINKCPLLKPWALTF

Uniprot       SYGRALQASALKAWGGKKENLKAAQEEYVKRALANSLACQGKYTPSGQAGAAASESLFVSNHAY
Splooce       SYGRALQASALKAWGGKKENLKAAQEEYVKRALANSLACQGKYTPSGQAGAAASESLFVSNHAY

----------------------------------------------------------------------------------------------------

P19338 (Uniprot)	versus
NM_005381#(-s-s-:2_N5866450872295) (Splooce)

For more details about the Alternative Splicing Event -> Link to Splooce page

Peptides that support the ASE (Splooce-specific):
IAYIEFK (MAXQUANT)
LAYLEFK (PEAKS)

Alignment:
Uniprot       MVKLAKAGKNQGDPKKMAPPPKEVEEDSEDEEMSEDEEDDSSGEEVVIPQKKGKKAAATSAKKVVVSPTKKVAVATPAKKAAVTPGKKAAATPAKKTVTP
Splooce       MVKLAKAGKNQGDPKKMAPPPKEVEEDSEDEEMSEDEEDDSSGEEVVIPQKKGKKAAATSAKKVVVSPTKKVAVATPAKKAAVTPGKKAAATPAKKTVTP

Uniprot       AKAVTTPGKKGATPGKALVATPGKKGAAIPAKGAKNGKNAKKEDSDEEEDDDSEEDEEDDEDEDEDEDEIEPAAMKAAAAAPASEDEDDEDDEDDEDDDD
Splooce       AKAVTTPGKKGATPGKALVATPGKKGAAIPAKGAKNGKNAKKEDSDEEEDDDSEEDEEDDEDEDEDEDEIEPAAMKAAAAAPASEDEDDEDDEDDEDDDD

Uniprot       DEEDDSEEEAMETTPAKGKKAAKVVPVKAKNVAEDEDEEEDDEDEDDDDDEDDEDDDDEDDEEEEEEEEEEPVKEAPGKRKKEMAKQKAAPEAKKQKVEG
Splooce       DEEDDSEEEAMETTPAKGKKAAKVVPVKAKNVAEDEDEEEDDEDEDDDDDEDDEDDDDEDDEEEEEEEEEEPVKEAPGKRKKEMAKQKAAPEAKKQKVEG

Uniprot       TEPTTAFNLFVGNLNFNKSAPELKTGISDVFAKNDLAVVDVRIGMTRKFGYVDFESAEDLEKALELTGLKVFGNEIKLEKPKGKDSKKERDARTLLAKNL
Splooce       TEPTTAFNLFVGNLNFNKSAPELKTGISDVFAKNDLAVVDVRIGMTR-----------------------------------------------------

Uniprot       PYKVTQDELKEVFEDAAEIRLVSKDGKSKGIAYIEFKTEADAEKTFEEKQGTEIDGRSISLYYTGEKGQNQDYRGGKNSTWSGESKTLVLSNLSYSATEE
Splooce       ------------------------------IAYIEFKTEADAEKTFEEKQGTEIDGRSISLYYTGEKGQNQDYRGGKNSTWSGESKTLVLSNLSYSATEE

Uniprot       TLQEVFEKATFIKVPQNQNGKSKGYAFIEFASFEDAKEALNSCNKREIEGRAIRLELQGPRGSPNARSQPSKTLFVKGLSEDTTEETLKESFDGSVRARI
Splooce       TLQEVFEKATFIKVPQNQNGKSKGYAFIEFASFEDAKEALNSCNKREIEGRAIRLELQGPRGSPNARSQPSKTLFVKGLSEDTTEETLKESFDGSVRARI

Uniprot       VTDRETGSSKGFGFVDFNSEEDAKAAKEAMEDGEIDGNKVTLDWAKPKGEGGFGGRGGGRGGFGGRGGGRGGRGGFGGRGRGGFGGRGGFRGGRGGGGDH
Splooce       VTDRETGSSKGFGFVDFNSEEDAKAAKEAMEDGEIDGNKVTLDWAKPKGEGGFGGRGGGRGGFGGRGGGRGGRGGFGGRGRGGFGGRGGFRGGRGGGGDH

Uniprot       KPQGKKTKFE
Splooce       KPQGKKTKFE

----------------------------------------------------------------------------------------------------

E9PAL8 (Uniprot)	versus
NM_001042535#(-t:7_A9414999463092) (Splooce)

For more details about the Alternative Splicing Event -> Link to Splooce page

Peptides that support the ASE (Splooce-specific):
RPPSSPLQR (MAXQUANT)

Alignment:
Uniprot       MNFQAGGGQSPQQQQSLAAPGGGGAAAQQLVCGGQFGGAGPGAGGGGGPSQQLAGGPPQQFALSNSAAIRAEIQRFESVHPNIYAIYDLIERIEDLALQN
Splooce       MNFQAGGGQSPQQQQSLAAPGGGGAAAQQLVCGGQFGGAGPGAGGGGGPSQQLAGGPPQQFALSNSAAIRAEIQRFESVHPNIYAIYDLIERIEDLALQN

Uniprot       QIREHVISIEDSFVNSQEWTLSRSVPELKVGIVGNLSSGKSALVHRYLTGTYVQEESPEGGRFKKEIVVDGQSYLLLIRDEGGPPELQFAAWVDAVVFVF
Splooce       QIREHVISIEDSFVNSQEWTLSRSVPELKVGIVGNLSSGKSALVHRYLTGTYVQEESPEGGRFKKEIVVDGQSYLLLIRDEGGPPELQFAAWVDAVVFVF

Uniprot       SLEDEISFQTVYNYFLRLCSFRNASEVPMVLVGTQDAISAANPRVIDDSRARKLSTDLKRCTYYETCATYGLNVERVFQDV-------------------
Splooce       SLEDEISFQTVYNYFLRLCSFRNASEVPMVLVGTQDAISAANPRVIDDSRARKLSTDLKRCTYYETCATYGLNVERVFQDVGIIDSAVPCSGPEGSGLAK

Uniprot       --AQKVVALRKKQQLA--IGPCKSLPNSPSHSAVSAASIPAVH-------INQATNGGGSAFSDYSSSVP--STPSISQRELRIET=========IAASS
Splooce       EAATGHRALQVTAQLAQPLGRVRRLHPGRAHQPGHEWRRQRLQRLLVLSPLHPQHQPAGAAHRDHRCLLHPHTHPKAVQAALQHLHDMCHCFQLFINKKA

Uniprot       TPTPIRKQSKRRSNIFTICATVSN---------FSSTKRP-----------FQLLPN----
Splooce       FPTPSKLEDQLVTHSTSGHAPCAGPEAGRALGLLSDEKRPPSSPLQRPTGTFVTNPTPREG

----------------------------------------------------------------------------------------------------

Q9UG63 (Uniprot)	versus
NM_007189#(-s-s-s-s-:7_A9482011122752) (Splooce)

For more details about the Alternative Splicing Event -> Link to Splooce page

Peptides that support the ASE (Splooce-specific):
SLHLSLWCK (MAXQUANT)

Alignment:
Uniprot       MPSDLAKKKAAKKKEAAKARQRPRKGHEENGDVVTEPQVAEKNEANGRETTEVDLLTKELEDFEMKKAAARAVTGVLASHPNSTDVHIINLSLTFHGQEL
Splooce       MPSDLAKKKAAKKKEAAKARQRPRKGHEENGDVVTEPQVAEKNEANGRETTEVDLLTKELEDFEMKKAAARAVTGVLASHPNSTDVHIINLSLTFHGQEL

Uniprot       LSDTKLELNSGRRYGLIGLNGIGKSMLLSAIGKREVPIPEHIDIYHLTREMPPSDKTPLHCVMEVDTERAMLEKEAERLAHEDAECEKLMELYERLEELD
Splooce       LSDTKLELNSGRRYGLIGLNGIGKSMLLSAIGKREVPIPEHIDIYHLTREMPPSDKTPLHCVMEVDTERAMLEKEAERLAHEDAECEKLMELYERLEELD

Uniprot       ADKAEMRASRILHGLGFTPAMQRKKLKDFSGGWRMRVALARALFIRPFMLLLDEPTNHLDLDACVWLEEELKTFKRILVLVSHSQDFLNGVCTNIIHMHN
Splooce       ADKAEMRASRILHGLGFTPAMQRKKLKDFSGGWRMRVALAR-------------------------------------------------------HCH-

Uniprot       KKLKYYTGNYDQYVKTRLELEENQMKRFHWEQDQIAHMKNYIARFGHGSAKLARQAQSKEKTLQKMMASGLTERVVSDKTLSFYFPPCGKIPPPVIMVQN
Splooce       ------------------------------------FISHHVAR-------------------------------------SLHLSLWCKM---------

Uniprot       VSFKYTKDGPCIYNNLEFGIDLDTRVALVGPNGAGKSTLLKLLTGELLPTDGMIRKHSHVKIGRYHQHLQEQLDLDLSPLEYMMKCYPEIKEKEEMRKII
Splooce       ----------------------------------------------------------------------------------------------------

Uniprot       GRYGLTGKQQVSPIRNLSDGQKCRVCLAWLAWQNPHMLFLDEPTNHLDIETIDALADAINEFEGGMMLVSHDFRLIQQVAQEIWVCEKQTITKWPGDILA
Splooce       ----------------------------------------------------------------------------------------------------

Uniprot       YKEHLKSKLVDEEPQLTKRTHNV
Splooce       -----------------------

----------------------------------------------------------------------------------------------------

P06576 (Uniprot)	versus
NM_001686#(-s-s-:12_A7426188989484) (Splooce)

For more details about the Alternative Splicing Event -> Link to Splooce page

Peptides that support the ASE (Splooce-specific):
AIYVPADDLTDPAPATTFAHLDATTVLSR (MAXQUANT)

Alignment:
Uniprot       MLGFVGRVAAAPASGALRRLTPSASLPPAQLLLRAAPTAVHPVRDYAAQTSPSPKAGAATGRIVAVIGAVVDVQFDEGLPPILNALEVQGRETRLVLEVA
Splooce       MLGFVGRVAAAPASGALRRLTPSASLPPAQLLLRAAPTAVHPVRDYAAQTSPSPKAGAATGRIVAVIGAVVDVQFDEGLPPILNALEVQGRETRLVLEVA

Uniprot       QHLGESTVRTIAMDGTEGLVRGQKVLDSGAPIKIPVGPETLGRIMNVIGEPIDERGPIKTKQFAPIHAEAPEFMEMSVEQEILVTGIKVVDLLAPYAKGG
Splooce       QHLGESTVRTIAMDGTEGLVRGQKVLDSGAPIKIPVGPETLGRIMNVIGEPIDERGPIKTKQFAPIHAEAPEFMEMSVEQEILVTGIKVVDLLAPYAKGG

Uniprot       KIGLFGGAGVGKTVLIMELINNVAKAHGGYSVFAGVGERTREGNDLYHEMIESGVINLKDATSKVALVYGQMNEPPGARARVALTGLTVAEYFRDQEGQD
Splooce       KIGLFGGAGVGKTVLIMELINNVAKAHGGYSVFAGVGERTREGNDLYHEMIESGVINLKDATS-------------------------------------

Uniprot       VLLFIDNIFRFTQAGSEVSALLGRIPSAVGYQPTLATDMGTMQERITTTKKGSITSVQAIYVPADDLTDPAPATTFAHLDATTVLSRAIAELGIYPAVDP
Splooce       ---------------------------------------------------------KAIYVPADDLTDPAPATTFAHLDATTVLSRAIAELGIYPAVDP

Uniprot       LDSTSRIMDPNIVGSEHYDVARGVQKILQDYKSLQDIIAILGMDELSEEDKLTVSRARKIQRFLSQPFQVAEVFTGHMGKLVPLKETIKGFQQILAGEYD
Splooce       LDSTSRIMDPNIVGSEHYDVARGVQKILQDYKSLQDIIAILGMDELSEEDKLTVSRARKIQRFLSQPFQVAEVFTGHMGKLVPLKETIKGFQQILAGEYD

Uniprot       HLPEQAFYMVGPIEEAVAKADKLAEEHSS
Splooce       HLPEQAFYMVGPIEEAVAKADKLAEEHSS

----------------------------------------------------------------------------------------------------

Q9Y3I0 (Uniprot)	versus
NM_014306#(-s-s-s-s-s-:22_C9991786641477) (Splooce)

For more details about the Alternative Splicing Event -> Link to Splooce page

Peptides that support the ASE (Splooce-specific):
GLPQAPESYK (MAXQUANT)

Alignment:
Uniprot       MSRSYNDELQFLEKINKNCWRIKKGFVPNMQVEGVFYVNDALEKLMFEELRNACRGGGVGGFLPAMKQIGNVAALPGIVHRSIGLPDVHSGYGFAIGNMA
Splooce       MSRSYNDELQFLEKINKNCWRIKKGFVPNMQVEGVFYVNDALEKLMFEELRNACRGGGVGGFLPAMKQIGNVAALPGIVHRSIGLPDVHSGYGFAIGNMA

Uniprot       AFDMNDPEAVVSPGGVGFDINCGVRLLRTNLDESDVQPVKEQLAQAMFDHIPVGVGSKGVIPMNAKDLEEALEMGVDWSLREGYAWAEDKEHCEEYGRML
Splooce       AFDMNDPEAVVSPGGVGFDINCGVRLLRTNLDESDVQPVKEQLAQAMFDHIPVGVGSKGVIPMNAKDLEEALEMGVDWSLREGYAWAEDKEHCEEYGRML

Uniprot       QADPNKVSARAKKRGLPQLGTLGAGNHYAEIQVVDEIFNEYAAKKMGIDHKGQVCVMIHSGSRGLGHQVATDALVAMEKAMKRDKIIVNDRQLACARIAS
Splooce       QADPNKVSARAKKRGLP-----------------------------------------------------------------------------------

Uniprot       PEGQDYLKGMAAAGNYAWVNRSSMTFLTRQAFAKVFNTTPDDLDLHVIYDVSHNIAKVEQHVVDGKERTLLVHRKGSTRAFPPHHPLIAVDYQLTGQPVL
Splooce       ----------------------------------------------------------------------------------------------------

Uniprot       IGGTMGTCSYVLTGTEQGMTETFGTTCHGAGRALSRAKSRRNLDFQDVLDKLADMGIAIRVASPKLVMEEAPESYKNVTDVVNTCHDAGISKKAIKLRPI
Splooce       ---------------------------------------------------------------------QAPESYKNVTDVVNTCHDAGISKKAIKLRPI

Uniprot       AVIKG
Splooce       AVIKG

----------------------------------------------------------------------------------------------------

A1L0T0 (Uniprot)	versus
NM_006844#(-s-:19_I845322466525) (Splooce)

For more details about the Alternative Splicing Event -> Link to Splooce page

Peptides that support the ASE (Splooce-specific):
NWASVWWTHAMR (MAXQUANT)

Alignment:
Uniprot       METPAAAAPAGSLFPSFLLLACGTLVAALLGAAHRLGLFYQLLHKVDKASVRHGGENVAAVLRAHGVRFIFTLVGGHISPLLVACEKLGIRVVDTRHEVT
Splooce       -------------------MVCGS------SSRWSVGTFPRCWWPVR---------NWASVWWTH-----------------------------------

Uniprot       AVFAADAMARLSGTVGVAAVTAGPGLTNTVTAVKNAQMAQSPILLLGGAASTLLQNRGALQAVDQLSLFRPLCKFCVSVRRVRDIVPTLRAAMAAAQSGT
Splooce       ------------------AMRSRP----------------SLLLMLWPACP----NRGALQAVDQLSLFRPLCKFCVSVRRVRDIVPTLRAAMAAAQSGT

Uniprot       PGPVFVELPVDVLYPYFMVQKEMVPAKPPKGLVGRVVSWYLENYLANLFAGAWEPQPEGPLPLDIPQASPQQVQRCVEILSRAKRPLMVLGSQALLTPTS
Splooce       PGPVFVELPVDVLYPYFMVQKEMVPAKPPKGLVGRVVSWYLENYLANLFAGAWEPQPEGPLPLDIPQASPQQVQRCVEILSRAKRPLMVLGSQALLTPTS

Uniprot       ADKLRAAVETLGVPCFLGGMARGLLGRNHPLHIRENRSAALKKADVIVLAGTVCDFRLSYGRVLSHSSKIIIVNRNREEMLLNSDIFWKPQEAVQGDVGS
Splooce       ADKLRAAVETLGVPCFLGGMARGLLGRNHPLHIRENRSAALKKADVIVLAGTVCDFRLSYGRVLSHSSKIIIVNRNREEMLLNSDIFWKPQEAVQGDVGS

Uniprot       FVLKLVEGLQGQTWAPDWVEELREADRQKEQTFREKAAMPVAQHLNPVQVLQLVEETLPDNSILVVDGGDFVGTAAHLVQPRGPLRWLDPGAFGTLGVGA
Splooce       FVLKLVEGLQGQTWAPDWVEELREADRQKEQTFREKAAMPVAQHLNPVQVLQLVEETLPDNSILVVDGGDFVGTAAHLVQPRGPLRWLDPGAFGTLGVGA

Uniprot       GFALGAKLCRPDAEVWCLFGDGAFGYSLIEFDTFVRHKIPVMALVGNDAGWTQISREQVPSLGSNVACGLAYTDYHKAAMGLGARGLLLSRENEDQVVKV
Splooce       GFALGAKLCRPDAEVWCLFGDGAFGYSLIEFDTFVRHKIPVMALVGNDAGWTQISREQVPSLGSNVACGLAYTDYHKAAMGLGARGLLLSRENEDQVVKV

Uniprot       LHDAQQQCRDGHPVVVNILIGRTDFRDGSIAV
Splooce       LHDAQQQCRDGHPVVVNILIGRTDFRDGSIAV

----------------------------------------------------------------------------------------------------

P52907 (Uniprot)	versus
NM_006135#(-t:1_C3080408519345) (Splooce)

For more details about the Alternative Splicing Event -> Link to Splooce page

Peptides that support the ASE (Splooce-specific):
IQMAMFSWLVIK (MAXQUANT)

Alignment:
Uniprot       MADFDDRVSDEEKVRIAAKFITHAPPGEFNEVFNDVRLLLNNDNLLREGAAHAFAQYNMDQFTPVKIEGYEDQVLITEHGDLGNSRFLDPRNKISFKFDH
Splooce       MADFDDRVSDEEKVRIAAKFITHAPPGEFNEVFNDVRLLLNNDNLLREGAAHAFAQYNMDQFTPVKIEGYEDQVLITEHGDLGNSRFLDPRNKISFKFDH

Uniprot       LRKEASDPQPEEADGGLKSWRESCDSALRAYVKDHYSNGFCTVYAKTIDGQQTIIACIESHQFQPKNFWNGRWRSEWKFTITPPTAQVVGVLKIQVHYYE
Splooce       LRKEASDPQPEEADGGLKSWRESCDSALRAYVKDHYSNGFCTVYAKTIDGQQTIIACIESHQFQPKNFWNGRWRSEWKFTITPPTAQVVGVLKIQMAMFS

Uniprot       DGNVQLVSHKDVQDSLTVSNEAQTAKEFIKIIENAENEYQTAISENYQTMSDTTFKALRRQLPVTRTKIDWNKILSYKIGKEMQNA
Splooce       ----------------------------------------------------------------------WLVIKMYRIH------

----------------------------------------------------------------------------------------------------

Q9BQE6 (Uniprot)	versus
NM_024099#(-s-:11_C8219727156569) (Splooce)

For more details about the Alternative Splicing Event -> Link to Splooce page

Peptides that support the ASE (Splooce-specific):
TAMEVEAPSK (MAXQUANT + PEAKS)
APQDVEMK (MAXQUANT + PEAKS)
TAMEVEAPSKPAR (MAXQUANT + PEAKS)
TSEPQLKR (MAXQUANT)
APQDVEMKDLEDES (MAXQUANT)

Alignment:
Uniprot       -MALVPGRSKEDGLWTRNSPGSSQHPESPRLPNPLWDRGKIGKVEGHQHIQDFSQKSHLPSIVVESSEVNEESGDLHLPHEELLLLTDGEEEDAEAFFQD
Splooce       MKKMLVGFLRTQHVWKPPTTVPSGTQQAP--------------------------------VFVEVALWN-------IPISCLLIALR---ELKKKLFKR

Uniprot       QSEEPGWAWSPQDPRSPLRTFNAGLSWGQDQDEEDACWILEDTACLEATNHCPFWDSTGSRVCRSGFVEYSHLLPPNSFEGAEEEAVQTPAGVESGAASE
Splooce       RRVLN------RERRLRHRVVGAVIDQG=================LITRHHLKKRASSARANITLSGKKRRKLLQQIRLAQKEKTAMEVEAPSKPARTSE

Uniprot       APGGRGCDRPRADHAAPPQEAGVQCTCQHYTVREEAQKTPPADPACPEREDSHGSGSPFKASQD
Splooce       P-----------------------------QLKRQKKTKAPQDVEMKDLEDES-----------

----------------------------------------------------------------------------------------------------

P35579 (Uniprot)	versus
NM_002473#(-s-s-s-s-s-:22_M6744507701174) (Splooce)

For more details about the Alternative Splicing Event -> Link to Splooce page

Peptides that support the ASE (Splooce-specific):
LTEMETLQSQVK (MAXQUANT)

Alignment:
Uniprot       MAQQAADKYLYVDKNFINNPLAQADWAAKKLVWVPSDKSGFEPASLKEEVGEEAIVELVENGKKVKVNKDDIQKMNPPKFSKVEDMAELTCLNEASVLHN
Splooce       MAQQAADKYLYVDKNFINNPLAQADWAAKKLVWVPSDKSGFEPASLKEEVGEEAIVELVENGKKVKVNKDDIQKMNPPKFSKVEDMAELTCLNEASVLHN

Uniprot       LKERYYSGLIYTYSGLFCVVINPYKNLPIYSEEIVEMYKGKKRHEMPPHIYAITDTAYRSMMQDREDQSILCTGESGAGKTENTKKVIQYLAYVASSHKS
Splooce       LKERYYSGLIYTYSGLFCVVINPYKNLPIYSEEIVEMYKGKKRHEMPPHIYAITDTAYRSMMQDREDQSILCTGESGAGKTENTKKVIQYLAYVASSHKS

Uniprot       KKDQGELERQLLQANPILEAFGNAKTVKNDNSSRFGKFIRINFDVNGYIVGANIETYLLEKSRAIRQAKEERTFHIFYYLLSGAGEHLKTDLLLEPYNKY
Splooce       KKDQGELERQLLQANPILEAFGNAKTVKNDNSSRFGKFIRINFDVNGYIVGANIETYLLEKSRAIRQAKEERTFHIFYYLLSGAGEHLKTDLLLEPYNKY

Uniprot       RFLSNGHVTIPGQQDKDMFQETMEAMRIMGIPEEEQMGLLRVISGVLQLGNIVFKKERNTDQASMPDNTAAQKVSHLLGINVTDFTRGILTPRIKVGRDY
Splooce       RFLSNGHVTIPGQQDKDMFQETMEAMRIMGIPEEEQMGLLRVISGVLQLGNIVFKKERNTDQASMPDNTAAQKVSHLLGINVTDFTRGILTPRIKVGRDY

Uniprot       VQKAQTKEQADFAIEALAKATYERMFRWLVLRINKALDKTKRQGASFIGILDIAGFEIFDLNSFEQLCINYTNEKLQQLFNHTMFILEQEEYQREGIEWN
Splooce       VQKAQTKEQADFAIEALAKATYERMFRWLVLRINKALDKTKRQGASFIGILDIAGFEIFDLNSFEQLCINYTNEKLQQLFNHTMFILEQEEYQREGIEWN

Uniprot       FIDFGLDLQPCIDLIEKPAGPPGILALLDEECWFPKATDKSFVEKVMQEQGTHPKFQKPKQLKDKADFCIIHYAGKVDYKADEWLMKNMDPLNDNIATLL
Splooce       FIDFGLDLQPCIDLIEKPAGPPGILALLDEECWFPKATDKSFVEKVMQEQGTHPKFQKPKQLKDKADFCIIHYAGKVDYKADEWLMKNMDPLNDNIATLL

Uniprot       HQSSDKFVSELWKDVDRIIGLDQVAGMSETALPGAFKTRKGMFRTVGQLYKEQLAKLMATLRNTNPNFVRCIIPNHEKKAGKLDPHLVLDQLRCNGVLEG
Splooce       HQSSDKFVSELWKDVDRIIGLDQVAGMSETALPGAFKTRKGMFRTVGQLYKEQLAKLMATLRNTNPNFVRCIIPNHEKKAGKLDPHLVLDQLRCNGVLEG

Uniprot       IRICRQGFPNRVVFQEFRQRYEILTPNSIPKGFMDGKQACVLMIKALELDSNLYRIGQSKVFFRAGVLAHLEEERDLKITDVIIGFQACCRGYLARKAFA
Splooce       IRICRQGFPNRVVFQEFRQRYEILTPNSIPKGFMDGKQACVLMIKALELDSNLYRIGQSKVFFRAGVLAHLEEERDLKITDVIIGFQACCRGYLARKAFA

Uniprot       KRQQQLTAMKVLQRNCAAYLKLRNWQWWRLFTKVKPLLQVSRQEEEMMAKEEELVKVREKQLAAENRLTEMETLQSQLMAEKLQLQEQLQAETELCAEAE
Splooce       KRQQQLTAMKVLQRNCAAYLKLRNWQWWRLFTKVKPLLQVSRQEEEMMAKEEELVKVREKQLAAENRLTEMETLQSQVKT--------------------

Uniprot       ELRARLTAKKQELEEICHDLEARVEEEEERCQHLQAEKKKMQQNIQELEEQLEEEESARQKLQLEKVTTEAKLKKLEEEQIILEDQNCKLAKEKKLLEDR
Splooce       ----------------------------------------------------------------------------------------------------

Uniprot       IAEFTTNLTEEEEKSKSLAKLKNKHEAMITDLEERLRREEKQRQELEKTRRKLEGDSTDLSDQIAELQAQIAELKMQLAKKEEELQAALARVEEEAAQKN
Splooce       ----------------------------------------------------------------------------------------------------

Uniprot       MALKKIRELESQISELQEDLESERASRNKAEKQKRDLGEELEALKTELEDTLDSTAAQQELRSKREQEVNILKKTLEEEAKTHEAQIQEMRQKHSQAVEE
Splooce       ----------------------------------------------------------------------------------------------------

Uniprot       LAEQLEQTKRVKANLEKAKQTLENERGELANEVKVLLQGKGDSEHKRKKVEAQLQELQVKFNEGERVRTELADKVTKLQVELDNVTGLLSQSDSKSSKLT
Splooce       ----------------------------------------------------------------------------------------------------

Uniprot       KDFSALESQLQDTQELLQEENRQKLSLSTKLKQVEDEKNSFREQLEEEEEAKHNLEKQIATLHAQVADMKKKMEDSVGCLETAEEVKRKLQKDLEGLSQR
Splooce       ----------------------------------------------------------------------------------------------------

Uniprot       HEEKVAAYDKLEKTKTRLQQELDDLLVDLDHQRQSACNLEKKQKKFDQLLAEEKTISAKYAEERDRAEAEAREKETKALSLARALEEAMEQKAELERLNK
Splooce       ----------------------------------------------------------------------------------------------------

Uniprot       QFRTEMEDLMSSKDDVGKSVHELEKSKRALEQQVEEMKTQLEELEDELQATEDAKLRLEVNLQAMKAQFERDLQGRDEQSEEKKKQLVRQVREMEAELED
Splooce       ----------------------------------------------------------------------------------------------------

Uniprot       ERKQRSMAVAARKKLEMDLKDLEAHIDSANKNRDEAIKQLRKLQAQMKDCMRELDDTRASREEILAQAKENEKKLKSMEAEMIQLQEELAAAERAKRQAQ
Splooce       ----------------------------------------------------------------------------------------------------

Uniprot       QERDELADEIANSSGKGALALEEKRRLEARIAQLEEELEEEQGNTELINDRLKKANLQIDQINTDLNLERSHAQKNENARQQLERQNKELKVKLQEMEGT
Splooce       ----------------------------------------------------------------------------------------------------

Uniprot       VKSKYKASITALEAKIAQLEEQLDNETKERQAACKQVRRTEKKLKDVLLQVDDERRNAEQYKDQADKASTRLKQLKRQLEEAEEEAQRANASRRKLQREL
Splooce       ----------------------------------------------------------------------------------------------------

Uniprot       EDATETADAMNREVSSLKNKLRRGDLPFVVPRRMARKGAGDGSDEEVDGKADGAEAKPAE
Splooce       ------------------------------------------------------------

----------------------------------------------------------------------------------------------------

O95163 (Uniprot)	versus
NM_003640#(-s-s-s-s-:9_I7427383452908) (Splooce)

For more details about the Alternative Splicing Event -> Link to Splooce page

Peptides that support the ASE (Splooce-specific):
IVTVVPQDTKLVLQILPIHTYISCKEDNPR (MAXQUANT)

Alignment:
Uniprot       MRNLKLFRTLEFRDIQGPGNPQCFSLRTEQGTVLIGSEHGLIEVDPVSREVKNEVSLVAEGFLPEDGSGRIVGVQDLLDQESVCVATASGDVILCSLSTQ
Splooce       MRNLKLFRTLEFRDIQGPGNPQCFSLRTEQGTVLIGSEHGLIEVDPVSREVKNEVSLVAEGFLPEDGSGRIVGVQDLLDQESVCVATASGDVILCSLSTQ

Uniprot       QLECVGSVASGISVMSWSPDQELVLLATGQQTLIMMTKDFEPILEQQIHQDDFGESKFITVGWGRKETQFHGSEGRQAAFQMQMHESALPWDDHRPQVTW
Splooce       QLECVGSVASGISVMSWSPDQELVLLATGQQTLIMMTKDFEPILEQQIHQDDFGESKFITVGWGRKETQFHGSEGRQAAFQMQMHESALPWDDHRPQVTW

Uniprot       RGDGQFFAVSVVCPETGARKVRVWNREFALQSTSEPVAGLGPALAWKPSGSLIASTQDKPNQQDIVFFEKNGLLHGHFTLPFLKDEVKVNDLLWNADSSV
Splooce       RGDGQFFAVSVVCPETGARKVRVWNREFALQSTSEPVAGLGPALAWKPSGSLIASTQDKPNQQDIVFFEKNGLLHGHFTLPFLKDEVKVNDLLWNADSSV

Uniprot       LAVWLEDLQREESSIPKTCVQLWTVGNYHWYLKQSLSFSTCGKSKIVSLMWDPVTPYRLHVLCQGWHYLAYDWHWTTDRSVGDNSSDLSNVAVIDGNRVL
Splooce       LAVWLEDLQREESSIPKTCVQLWTVGNYHWYLKQSLSFSTCGKSKIVSLMWDPVTPYRLHVLCQGWHYLAYDWHWTTDRSVGDNSSDLSNVAVIDGNRVL

Uniprot       VTVFRQTVVPPPMCTYQLLFPHPVNQVTFLAHPQKSNDLAVLDASNQISVYKCGDCPSADPTVKLGAVGGSGFKVCLRTPHLEKRYKIQFENNEDQDVNP
Splooce       VTVFRQTVVPPPMCTYQLLFPHPVNQVTFLAHPQKSNDLAVLDASNQISVYKCGDCPSADPTVKLGAVGGSGFKVCLRTPHLEKRYKIQFENNEDQDVNP

Uniprot       LKLGLLTWIEEDVFLAVSHSEFSPRSVIHHLTAASSEMDEEHGQLNVSSSAAVDGVIISLCCNSKTKSVVLQLADGQIFKYLWESPSLAIKPWKNSGGFP
Splooce       LKLGLLTWIEEDVFLAVSHSEFSPRSVIHHLTAASSEMDEEHGQLNVSSSAAVDGVIISLCCNSKTKSVVLQLADGQIFKYLWESPSLAIKPWKNSGGFP

Uniprot       VRFPYPCTQTELAMIGEEECVLGLTDRCRFFINDIEVASNITSFAVYDEFLLLTTHSHTCQCFCLRDASFKTLQAGLSSNHVSHGEVLRKVERGSRIVTV
Splooce       VRFPYPCTQTELAMIGEEECVLGLTDRCRFFINDIEVASNITSFAVYDEFLLLTTHSHTCQCFCLRDASFKTLQAGLSSNHVSHGEVLRKVERGSRIVTV

Uniprot       VPQDTKLVLQMPRGNLEVVHHRALVLAQIRKWLDKLMFKEAFECMRKLRINLNLIYDHNPKVFLGNVETFIKQIDSVNHINLFFTELKEEDVTKTMYPAP
Splooce       VPQDTKLVLQILP---------------------------------------------------------------------------------------

Uniprot       VTSSVYLSRDPDGNKIDLVCDAMRAVMESINPHKYCLSILTSHVKKTTPELEIVLQKVHELQGNAPSDPDAVSAEEALKYLLHLVDVNELYDHSLGTYDF
Splooce       --IHTYISCKED------------------NPR----------------------------TGNCTAKSTRASRKCSL----------------------

Uniprot       DLVLMVAEKSQKDPKEYLPFLNTLKKMETNYQRFTIDKYLKRYEKAIGHLSKCGPEYFPECLNLIKDKNLYNEALKLYSPSSQQYQDISIAYGEHLMQEH
Splooce       ----------------------------------------------------------------------------------------------------

Uniprot       MYEPAGLMFARCGAHEKALSAFLTCGNWKQALCVAAQLNFTKDQLVGLGRTLAGKLVEQRKHIDAAMVLEECAQDYEEAVLLLLEGAAWEEALRLVYKYN
Splooce       ----------------------------------------------------------------------------------------------------

Uniprot       RLDIIETNVKPSILEAQKNYMAFLDSQTATFSRHKKRLLVVRELKEQAQQAGLDDEVPHGQESDLFSETSSVVSGSEMSGKYSHSNSRISARSSKNRRKA
Splooce       ----------------------------------------------------------------------------------------------------

Uniprot       ERKKHSLKEGSPLEDLALLEALSEVVQNTENLKDEVYHILKVLFLFEFDEQGRELQKAFEDTLQLMERSLPEIWTLTYQQNSATPVLGPNSTANSIMASY
Splooce       ----------------------------------------------------------------------------------------------------

Uniprot       QQQKTSVPVLDAELFIPPKINRRTQWKLSLLD
Splooce       --------------------------------

----------------------------------------------------------------------------------------------------

Q15052 (Uniprot)	versus
NM_004840#(-s-s-:X_A4747816646842) (Splooce)

For more details about the Alternative Splicing Event -> Link to Splooce page

Peptides that support the ASE (Splooce-specific):
EVQLLWK (MAXQUANT)

Alignment:
Uniprot       MNPEEQIVTWLISLGVLESPKKTICDPEEFLKSSLKNGVVLCKLINRLMPGSVEKFCLDPQTEADCINNINDFLKGCATLQVEIFDPDDLYSGVNFSKVL
Splooce       MNPEEQIVTWLISLGVLESPKKTICDPEEFLKSSLKNGVVLCKLINRLMPGSVEKFCLDPQTEADCINNINDFLKGCATLQVEIFDPDDLYSGVNFSKVL

Uniprot       STLLAVNKATEDQLSERPCGRSSSLSAANTSQTNPQGAVSSTVSGLQRQSKTVEMTENGSHQLIVKARFNFKQTNEDELSVCKGDIIYVTRVEEGGWWEG
Splooce       STLLAVNKATEDQLSERPCGRSSSLSAANTSQTNPQGAVSSTVSGLQRQSKTVEMTENGSHQLIVKARFNFKQTNEDELSVCKGDIIYVTRVEEGGWWEG

Uniprot       TLNGRTGWFPSNYVREIKSSERPLSPKAVKGFETAPLTKNYYTVVLQNILDTEKEYAKELQSLLVTYLRPLQSNNNLSTVEVTSLLGNFEEVCTFQQTLC
Splooce       TLNGRTGWFPSNYVREIKSSERPLSPKAVKGFETAPLTKNYYTVVLQNILDTEKEYAKELQSLLVTYLRPLQSNNNLSTVEVTSLLGNFEEVCTFQQTLC

Uniprot       QALEECSKFPENQHKVGGCLLSLMPHFKSMYLAYCANHPSAVNVLTQHSDELEQFMENQGASSPGILILTTNLSKPFMRLEKYVTLLQELERHMEDTHPD
Splooce       QALEECSKFPENQHKVGGCLLSLMPHFKSMYLAYCANHPSAVNVLTQHSDELEQFMENQGASSPGILILTTNLSKPFMRLEKYVTLLQELERHMEDTHPD

Uniprot       HQDILKAIVAFKTLMGQCQDLRKRKQLELQILSEPIQAWEGEDIKNLGNVIFMSQVMVQYGACEEKEERYLMLFSNVLIMLSASPRMSGFIYQGKIPIAG
Splooce       HQDILKAIVAFKTLMGQCQDLRKRKQLELQILSEPIQAWEGEDIKNLGNVIFMSQVMVQYGACEEKEERYLMLFSNVLIMLSASPRMSGFIYQGKIPIAG

Uniprot       TVVTRLDEIEGNDCTFEITGNTVERIVVHCNNNQDFQEWLEQLNRLIRGPASCSSLSKTSSSSCSAHSSFSSTGQPRGPLEPPQIIKPWSLSCLRPAPPL
Splooce       TVVTRLDEIEGNDCTFEITGNTVERIVVHCNNNQDFQEWLEQLNRLIRGPASCSSLSKTSSSSCSAHSSFSSTGQPRGPLEPPQIIKPWSLSCLRPAPPL

Uniprot       RPSAALGYKERMSYILKESSKSPKTMKKFLHKRKTERKPSEEEYVIRKSTAALEEDAQILKVIEAYCTSANFQQGHGSSTRKDSIPQVLLPEEEKLIIEE
Splooce       RPSAALGYKE-VQLLWKRMLKSLK----------------------------------------------------------------------------

Uniprot       TRSNGQTIMEEKSLVDTVYALKDEVRELKQENKRMKQCLEEELKSRRDLEKLVRRLLKQTDECIRGESSSKTSILP
Splooce       ----------------------------------------------------------------------------

----------------------------------------------------------------------------------------------------

Q8N5Z5 (Uniprot)	versus
NM_024681#(-s-s-:22_K8038875478766) (Splooce)

For more details about the Alternative Splicing Event -> Link to Splooce page

Peptides that support the ASE (Splooce-specific):
LVLDKDMAEEAGEHR (MAXQUANT)

Alignment:
Uniprot       MQTPRPAMRMEAGEAAPPAGAGGRAAGGWGKWVRLNVGGTVFLTTRQTLCREQKSFLSRLCQGEELQSDRDETGAYLIDRDPTYFGPILNFLRHGKLVLD
Splooce       MQTPRPAMRMEAGEAAPPAGAGGRAAGGWGKWVRLNVGGTVFLTTRQTLCREQKSFLSRLCQGEELQSDRDETGAYLIDRDPTYFGPILNFLRHGKLVLD

Uniprot       KDMAEEGVLEEAEFYNIGPLIRIIKDRMEEKDYTVTQVPPKHVYRVLQCQEEELTQMVSTMSDGWRFEQLVNIGSSYNYGSEDQAEFLCVVSKELHSTPN
Splooce       KDMAEE----------------------AGEHRLLLQLRQRGPGRVPVCG--------------------------------------------VQGAP-

Uniprot       GLSSESSRKTKSTEEQLEEQQQQEEEVEEVEVEQVQVEADAQEKGSRPHPLRPEAELAVRASPRPLARPQSCHPCCYKPEAPGCEAPDHLQGLGVPI
Splooce       -------QHPKRAELRVQPQNQ----------EHGGAAGGAAAAGG--------------GGGGGGGGTGAGGGRCTGERFP--SAPSQT-------

----------------------------------------------------------------------------------------------------

P06241 (Uniprot)	versus
NM_153047#(-s-s-:6_F8926494640721) (Splooce)

For more details about the Alternative Splicing Event -> Link to Splooce page

Peptides that support the ASE (Splooce-specific):
KLGQGCFAEVWLGGCR (MAXQUANT)

Alignment:
Uniprot       MGCVQCKDKEATKLTEERDGSLNQSSGYRYGTDPTPQHYPSFGVTSIPNYNNFHAAGGQGLTVFGGVNSSSHTGTLRTRGGTGVTLFVALYDYEARTEDD
Splooce       MGCVQCKDKEATKLTEERDGSLNQSSGYRYGTDPTPQHYPSFGVTSIPNYNNFHAAGGQGLTVFGGVNSSSHTGTLRTRGGTGVTLFVALYDYEARTEDD

Uniprot       LSFHKGEKFQILNSSEGDWWEARSLTTGETGYIPSNYVAPVDSIQAEEWYFGKLGRKDAERQLLSFGNPRGTFLIRESETTKGAYSLSIRDWDDMKGDHV
Splooce       LSFHKGEKFQILNSSEGDWWEARSLTTGETGYIPSNYVAPVDSIQAEEWYFGKLGRKDAERQLLSFGNPRGTFLIRESETTKGAYSLSIRDWDDMKGDHV

Uniprot       KHYKIRKLDNGGYYITTRAQFETLQQLVQHYSEKADGLCFNLTVIASSCTPQTSGLAKDAWEVARRSLCLEKKLGQGCFAEVWLGTWNGNTKVAIKTLKP
Splooce       KHYKIRKLDNGGYYITTRAQFETLQQLVQHYSEKADGLCFNLTVIASSCTPQTSGLAKDAWEVARRSLCLEKKLGQGCFAEVWLGGCRNG----------

Uniprot       GTMSPESFLEEAQIMKKLKHDKLVQLYAVVSEEPIYIVTEYMNKGSLLDFLKDGEGRALKLPNLVDMAAQVAAGMAYIERMNYIHRDLRSANILVGNGLI
Splooce       -------------------LHRAHELYP------------------------------------------------------------------------

Uniprot       CKIADFGLARLIEDNEYTARQGAKFPIKWTAPEAALYGRFTIKSDVWSFGILLTELVTKGRVPYPGMNNREVLEQVERGYRMPCPQDCPISLHELMIHCW
Splooce       ----------------------------------------------------------------------------------------------------

Uniprot       KKDPEERPTFEYLQSFLEDYFTATEPQYQPGENL
Splooce       ----------------------------------

----------------------------------------------------------------------------------------------------

B5ME19 (Uniprot)	versus
NM_001099661#(-t:16_E9892675039781) (Splooce)

For more details about the Alternative Splicing Event -> Link to Splooce page

Peptides that support the ASE (Splooce-specific):
KMDEDEDSEDSEDDEDWDTGSTSSDSDSEEEEGK (MAXQUANT)

Alignment:
Uniprot       MSRFFTTGSDSESESSLSGEELVTKPVGGNYGKQPLLLSEDEEDTKRVVRSAKDKRFEELTNLIRTIRNAMKIRDVTKCLEEFELLGKAYGKAKSIVDKE
Splooce       MSRFFTTGSDSESESSLSGEELVTKPVGGNYGKQPLLLSEDEEDTKRVVRSAKDKRFEELTNLIRTIRNAMKIRDVTKCLEEFELLGKAYGKAKSIVDKE

Uniprot       GVPRFYIRILADLEDYLNELWEDKEGKKKMNKNNAKALSTLRQKIRKYNRDFESHITSYKQNPEQSADEDAEKNEEDSEGSSDEDEDEDGVSAATFLKKK
Splooce       GVPRFYIRILADLEDYLNELWEDKEGKKKMNKNNAKALSTLRQKIRKYNRDFESHITSYKQNPEQSADEDAEKNEEDSEGSSDEDEDEDGVSAATFLKKK

Uniprot       SEAPSGESRKFLKKMDDEDEDSEDSEDDEDWDTGSTSSDSDSEEEEGKQTALASRFLKKAPTTDEDKKAAEKKREDKAKKKHDRKSKRLDEEEEEDNEGG
Splooce       SEAPSGESRKFLKKMD-EDEDSEDSEDDEDWDTGSTSSDSDSEEEEGKQTALASRFLKKAPTTDEDKKAAEKKREDKAKKKHDRKSKRLDEEEEEDNEGG

Uniprot       EWERVRGGVPLVKEKPKMFAKGTEITHAVVIKKLNEILQARGKKGTDRAAQIELLQLLVQIAAENNLGEGVIVKIKFNIIASLYDYNPNLATYMKPEMWG
Splooce       EWERVRGGVPLVKEKPKMFAKGTEITHAVVIKKLNEILQARGKKGTDRAAQIELLQLLVQIAAENNLGEGVIVKIKFNIIASLYDYNPNLATYMKPEMWG

Uniprot       KCLDCINELMDILFANPNIFVGENILEESENLHNADQPLRVRGCILTLVERMDEEFTKIMQNTDPHSQEYVEHLKDEAQVCAIIERVQRYLEEKGTTEEV
Splooce       KCLDCINELMDILFANPNIFVGENILEESENLHNADQPLRVRGCILTLVERMDEEFTKIMQNTDPHSQEYVEHLKDEAQVCAIIERVQRYLEEKGTTEEV

Uniprot       CRIYLLRILHTYYKFDYKAHQRQLTPPEGSSKSEQDQAENEGEDSAVLMERLCKYIYAKDRTDRIRTCAILCHIYHHALHSRWYQARDLMLMSHLQDNIQ
Splooce       CRIYLLRILHTYYKFDYKAHQRQLTPPEGSSKSEQDQAENEGEDSAVLMERLCKYIYAKDRTDRIRTCAILCHIYHHALHSRWYQARDLMLMSHLQDNIQ

Uniprot       HADPPVQILYNRTMVQLGICAFRQGLTKDAHNALLDIQSSGRAKELLGQGLLLRSLQERNQEQEKVERRRQVPFHLHINLELLECVYLVSAMLLEIPYMA
Splooce       HADPPVQILYNRTMVQLGICAFRQGLTKDAHNALLDIQSSGRAKELLGQGLLLRSLQERNQEQEKVERRRQVPFHLHINLELLECVYLVSAMLLEIPYMA

Uniprot       AHESDARRRMISKQFHHQLRVGERQPLLGPPESMREHVVAASKAMKMGDWKTCHSFIINEKMNGKVWDLFPEADKVRTMLVRKIQEESLRTYLFTYSSVY
Splooce       AHESDARRRMISKQFHHQLRVGERQPLLGPPESMREHVVAASKAMKMGDWKTCHSFIINEKMNGKVWDLFPEADKVRTMLVRKIQEESLRTYLFTYSSVY

Uniprot       DSISMETLSDMFELDLPTVHSIISKMIINEELMASLDQPTQTVVMHRTEPTAQQNLALQLAEKLGSLVENNERVFDHKQGTYGGYFRDQKDGYRKNEGYM
Splooce       DSISMETLSDMFELDLPTVHSIISKMIINEELMASLDQPTQTVVMHRTEPTAQQNLALQLAEKLGSLVENNERVFDHKQGTYGGYFRDQKDGYRKNEGYM

Uniprot       RRGGYRQQQSQTAY
Splooce       RRGGYRQQQSQTAY

----------------------------------------------------------------------------------------------------

P07951 (Uniprot)	versus
NM_213674#(f-:9_T266179780449) (Splooce)

For more details about the Alternative Splicing Event -> Link to Splooce page

Peptides that support the ASE (Splooce-specific):
KLVILEGELDR (MAXQUANT)
LVILEGELDR (MAXQUANT)

Alignment:
Uniprot       MDAIKKKMQMLKLDKENAIDRAEQAEADKKQAEDRCKQLEEEQQALQKKLKGTEDEVEKYSESVKEAQEKLEQAEKKATDAEADVASLNRRIQLVEEELD
Splooce       MDAIKKKMQMLKLDKENAIDRAEQAEADKKQAEDRCKQLEEEQQALQKKLKGTEDEVEKYSESVKEAQEKLEQAEKKATDAEADVASLNRRIQLVEEELD

Uniprot       RAQERLATALQKLEEAEKAADESERGMKVIENRAMKDEEKMELQEMQLKEAKHIAEDSDRKYEEVARKLVILEGELERSEERAEVAESRARQLEEELRTM
Splooce       RAQERLATALQKLEEAEKAADESERGMKVIENRAMKDEEKMELQEMQLKEAKHIAEDSDRKYEEVARKLVILEGELDR-----------ARQLEEELRTM

Uniprot       DQALKSLMASEEEYSTKEDKYEEEIKLLEEKLKEAETRAEFAERSVAKLEKTIDDLEETLASAKEENVEIHQTLDQTLLELNNL
Splooce       DQALKSLMASEEEYSTKEDKYEEEIKLLEEKLKEAETRAEFAERSVAKLEKTIDDLEETLASAKEENVEIHQTLDQTLLELNNL

----------------------------------------------------------------------------------------------------

UNIPROT? (Uniprot)	versus
NM_001130065#(-t:19_M6908661243054) (Splooce)

For more details about the Alternative Splicing Event -> Link to Splooce page

Peptides that support the ASE (Splooce-specific):
LGPDQWLR (MAXQUANT)

Alignment:
Uniprot       -------
Splooce       MSVKEAGSSGRREQAAYHLHIYPQLSTTESQASCRVTATKDSTTSDVIKDAIASLRLDGTKCYVLVEVKESGGEEWVLDANDSPVHRVLLWPRRAQDEHP

Uniprot       -------
Splooce       QEDGYYFLLQERNADGTIKYVHMQLVAQATATRRLVERGLLPRQQADFDDLCNLPELTEGNLLKNLKHRFLQQKIYTYAGSILVAINPFKFLPIYNPKYV

Uniprot       -------
Splooce       KMYENQQLGKLEPHVFALADVAYYTMLRKRVNQCIVISGESGSGKTQSTNFLIHCLTALSQKGYASGVERTILGAGPVLEAFGNAKTAHNNNSSRFGKFI

Uniprot       -------
Splooce       QVSYLESGIVRGAVVEKYLLEKSRLVSQEKDERNYHVFYYLLLGVSEEERQEFQLKQPEDYFYLNQHNLKIEDGEDLKHDFERLKQAMEMVGFLPATKKQ

Uniprot       -------
Splooce       IFAVLSAILYLGNVTYKKRATGREEGLEVGPPEVLDTLSQLLKVKREILVEVLTKRKTVTVNDKLILPYSLSEAITARDSMAKSLYSALFDWIVLRINHA

Uniprot       -------
Splooce       LLNKKDVEEAVSCLSIGVLDIFGFEDFERNSFEQFCINYANEQLQYYFNQHIFKLEQEEYQGEGITWHNIGYTDNVGCIHLISKKPTGLFYLLDEESNFP

Uniprot       -------
Splooce       HATSQTLLAKFKQQHEDNKYFLGTPVMEPAFIIQHFAGKVKYQIKDFREKNMDYMRPDIVALLRGSDSSYVRELIGMDPVAVFRWAVLRAAIRAMAVLRE

Uniprot       -------
Splooce       AGRLRAERAEKAAGMSSPGAQSHPEELPRGASTPSEKLYRDLHNQMIKSIKGLPWQGEDPRSLLQSLSRLQKPRAFILKSKGIKQKQIIPKNLLDSKSLK

Uniprot       -------
Splooce       LIISMTLHDRTTKSLLHLHKKKKPPSISAQFQTSLNKLLEALGKAEPFFIRCIRSNAEKKELCFDDELVLQQLRYTGMLETVRIRRSGYSAKYTFQDFTE

Uniprot       -------
Splooce       QFQVLLPKDAQPCREVISTLLEKMKIDKRNYQIGKTKVFLKETERQALQETLHREVVRKILLLQSWFRMVLERRHFLQMKRAAVTIQACWRSYRVRRALE

Uniprot       -------
Splooce       RTQAAVYLQASWRGYWQRKLYRHQKQSIIRLQSLCRGHLQRKSFSQMISEKQKAEEKEREALEAARAGAEEGGQGQAAGGQQVAEQGPEPAEDGGHLASE

Uniprot       -------
Splooce       PEVQPSDRSPLEHSSPEKEAPSPEKTLPPQKTVAAESHEKVPSSREKRESRRQRGLEHVKFQNKHIQSCKEESALREPSRRVTQEQGVSLLEDKKESRED

Uniprot       -------
Splooce       ETLLVVETEAENTSQKQPTEQPQAMAVGKVSEETEKTLPSGSPRPGQLERPTSLALDSRVSPPAPGSAPETPEDKSKPCGSPRVQEKPDSPGGSTQIQRY

Uniprot       -------
Splooce       LDAERLASAVELWRGKKLVAAASPSAMLSQSLDLSDRHRATGAALTPTEERRTSFSTSDVSKLLPSLAKAQPAAETTDGERSAKKPAVQKKKPGDASSLP

Uniprot       -------
Splooce       DAGLSPGSQVDSKSTFKRLFLHKTKDKKYSLEGAEELENAVSGHVVLEATTMKKGLEAPSGQQHRHAAGEKRTKEPGGKGKKNRNVKIGKITVSEKWRES

Uniprot       -------
Splooce       VFRQITNANELKYLDEFLLNKINDLRSQKTPIESLFIEATEKFRSNIKTMYSVPNGKIHVGYKDLMENYQIVVSNLATERGQKDTNLVLNLFQSLLDEFT

Uniprot       -------
Splooce       RGYTKNDFEPVKQSKAQKKKRKQERAVQEHNGHVFASYQVSIPQSCEQCLSYIWLMDKALLCSVCKMTCHKKCVHKIQSHCSYTYGRKGEPGVEPGHFGV

Uniprot       -------
Splooce       CVDSLTSDKASVPIVLEKLLEHVEMHGLYTEGLYRKSGAANRTRELRQALQTDPAAVKLENFPIHAITGVLKQWLRELPEPLMTFAQYGDFLRAVELPEK

Uniprot       -------
Splooce       QEQLAAIYAVLEHLPEANHNSLERLIFHLVKVALLEDVNRMSPGALAIIFAPCLLRCPDNSDPLTSMKDVLKITTCVEMLIKEQMRKYKVKMEEISQLEA

Uniprot       -------
Splooce       AESIAFRRLSLLRQNAPWPLKLGFSSPYEGVLNKSPKTRDIQEEELEVLLEEEAAGGDEDREKEILIERIQSIKEEKQGGHHLPAAGAGPKGLGRGEPGL

Uniprot       -------
Splooce       GDVGQHREPAGGAGRAGGLGRSVLRAPCACSPLPRRAHPEPPPHRGRPSTTKAVVLRNGQSEDPPADPHHAHGQHQAPTRPALPPASLGTGCPGGGCPSA

Uniprot       -------
Splooce       APGATCPPPGPDTFRVHHARGRPASAGRPGAPRRGWPATWGQAEVLGSPNVLPAPRLGPDQWLRATADKVCMSEDGPCTGAGRQSCRASVRPSEKDPESK

Uniprot       -------
Splooce       AQE

----------------------------------------------------------------------------------------------------

O43491 (Uniprot)	versus
NM_001431#(-s-:6_E3006322888493) (Splooce)

For more details about the Alternative Splicing Event -> Link to Splooce page

Peptides that support the ASE (Splooce-specific):
IVITGDGDIDHDQK (MAXQUANT)

Alignment:
Uniprot       MTTEVGSVSEVKKDSSQLGTDATKEKPKEVAENQQNQSSDPEEEKGSQPPPAAESQSSLRRQKREKETSESRGISRFIPPWLKKQKSYTLVVAKDGGDKK
Splooce       MTTEVGSVSEVKKDSSQLGTDATKEKPKEVAENQQNQSSDPEEEKGSQPPPAAESQSSLRRQKREKETSESRGISRFIPPWLKKQKSYTLVVAKDGGDKK

Uniprot       EPTQAVVEEQVLDKEEPLPEEQRQAKGDAEEMAQKKQEIKVEVKEEKPSVSKEEKPSVSKVEMQPTELVSKEREEKVKETQEDKLEGGAAKRETKEVQTN
Splooce       EPTQAVVEEQVLDKEEPLPEEQRQAKGDAEEMAQKKQEIKVEVKEEKPSVSKEEKPSVSKVEMQPTELVSKEREEKVKETQEDKLEGGAAKRETKEVQTN

Uniprot       ELKAEKASQKVTKKTKTVQCKVTLLDGTEYSCDLEKHAKGQVLFDKVCEHLNLLEKDYFGLLFQESPEQKNWLDPAKEIKRQLRNLPWLFTFNVKFYPPD
Splooce       ELKAEKASQKVTKKTKTVQCKVTLLDGTEYSCDLEKHAKGQVLFDKVCEHLNLLEKDYFGLLFQESPEQKNWLDPAKEIKRQLRNLPWLFTFNVKFYPPD

Uniprot       PSQLTEDITRYFLCLQLRQDIASGRLPCSFVTHALLGSYTLQAELGDYDPEEHGSIDLSEFQFAPTQTKELEEKVAELHKTHRGLSPAQADSQFLENAKR
Splooce       PSQLTEDITRYFLCLQLRQDIASGRLPCSFVTHALLGSYTLQAELGDYDPEEHGSIDLSEFQFAPTQTKELEEKVAELHKTHRGLSPAQADSQFLENAKR

Uniprot       LSMYGVDLHHAKDSEGVDIKLGVCANGLLIYKDRLRINRFAWPKILKISYKRSNFYIKVRPAELEQFESTIGFKLPNHRAAKRLWKVCVEHHTFYRLVSP
Splooce       LSMYGVDLHHAKDSEGVDIKLGVCANGLLIYKDRLRINRFAWPKILKISYKRSNFYIKVRPAELEQFESTIGFKLPNHRAAKRLWKVCVEHHTFYRLVSP

Uniprot       EQPPKAKFLTLGSKFRYSGRTQAQTRQASTLIDRPAPHFERTSSKRVSRSLDGAPIGVMDQSLMKDFPGAAGEISAYGPGLVSIAVVQDGDGRREVRSPT
Splooce       EQPPKAKFLTLGSKFRYSGRTQAQTRQASTLIDRPAPHFERTSSKRVSRSLDGAPIGVMDQSLMKDFPGAAGEISAYGPGLVSIAVVQDGDGRREVRSPT

Uniprot       KAPHLQLIEGKKNSLRVEGDNIYVRHSNLMLEELDKAQEDILKHQASISELKRNFMESTPEPRPNEWEKRRITPLSLQTQGSSHETLNIVEEKKRAEVGK
Splooce       KAPHLQLIEGKKNSLRVEGDNIYVRHSNLMLEELDKAQEDILKHQASISELKRNFMESTPEPRPNEWEKRRITPLSLQTQGSSHETLNIVEEKKRAEVGK

Uniprot       DERVITEEMNGKEISPGSGPGEIRKVEPVTQKDSTSLSSESSSSSSESEEEDVGEYRPHHRVTEGTIREEQEYEEEVEEEPRPAAKVVEREEAVPEASPV
Splooce       DERVITEEMNGKEISPGSGPGEIRKVEPVTQKDSTSLSSESSSSSSESEEEDVGEYRPHHRVTEGTIREEQEYEEEVEEEPRPAAKVVEREEAVPEASPV

Uniprot       TQAGASVITVETVIQENVGAQKIPGEKSVHEGALKQDMGEEAEEEPQKVNGEVSHVDIDVLPQIICCSEPPVVKTEMVTISDASQRTEISTKEVPIVQTE
Splooce       TQAGASVITVETVIQENVGAQKIPGEKSVHEGALKQDMGEEAEEEPQKVNGEVSHVDIDVLPQIICCSEPPVVKTEMVTISDASQRTEISTKEVPIVQTE

Uniprot       TKTITYESPQIDGGAGGDSGTLLTAQTITSESVSTTTTTHITKTVKGGISETRIEKRIVITGDGDIDHDQALAQAIREAREQHPDMSVTRVVVHKETELA
Splooce       TKTITYESPQIDGGAGGDSGTLLTAQTITSESVSTTTTTHITKTVKGGISETRIEKRIVITGDGDIDHDQKVIF--------------------------

Uniprot       EEGED
Splooce       -----

----------------------------------------------------------------------------------------------------

P52735 (Uniprot)	versus
NM_001134398#(-s-s-s-s-s-s-s-s-:9_V7823623859425) (Splooce)

For more details about the Alternative Splicing Event -> Link to Splooce page

Peptides that support the ASE (Splooce-specific):
LAGFLQR (MAXQUANT)

Alignment:
Uniprot       MGMTEDDKRNCCLLEIQETEAKYYRTLEDIEKNYMSPLRLVLSPADMAAVFINLEDLIKVHHSFLRAIDVSVMVGGSTLAKVFLDFKERLLIYGEYCSHM
Splooce       MGMTEDDKRNCCLLEIQETEAKYYRTLEDIEKNYMSPLRLVLSPADMAAVFINLEDLIKVHHSFLRAIDVSVMVGGSTLAKVFLDFKERLLIYGEYCSHM

Uniprot       EHAQNTLNQLLASREDFRQKVEECTLKVQDGKFKLQDLLVVPMQRVLKYHLLLKELLSHSAERPERQQLKEALEAMQDLAMYINEVKRDKETLRKISEFQ
Splooce       EHAQNTLNQLLASREDFRQKVEECTLKVQDGKFKLQDLLVVPMQRVLKYHLLLKELLSHSAERPERQQLKEALEAMQDLAMYINEVKRDKETLRKISEFQ

Uniprot       SSIENLQVKLEEFGRPKIDGELKVRSIVNHTKQDRYLFLFDKVVIVCKRKGYSYELKEIIELLFHKMTDDPMNNKDVKKPLSPLQWSYGFYLIHLQGKQG
Splooce       SSIENLQVKLEEFGRPKIDGELKVRSIVNHTKQDRYLFLFDKVVIVCKRKGYSYELKEIIELLFHKMTDDPMNNKDVKKPLSPLQWSYGFYLIHLQGKQG

Uniprot       FQFFCKTEDMKRKWMEQFEMAMSNIKPDKANANHHSFQMYTFDKTTNCKACKMFLRGTFYQGYMCTKCGVGAHKECLEVIPPCKFILVLTGRLRSGTRSQ
Splooce       FQFFCKTEDMKRKWMEQFEMAMSNIKPDKANANHHSFQMYTFDKTTNCKACKMFLRGTFYQGYMCTKCGVGAHKECLEVIPPCKFILVLTGRLRSGTR--

Uniprot       DGGHAELPWQPSPSREACADLPDGRRA
Splooce       LAGFLQRT-------------------

----------------------------------------------------------------------------------------------------

Q92506 (Uniprot)	versus
NM_014234#(-s-:6_H9097051078792) (Splooce)

For more details about the Alternative Splicing Event -> Link to Splooce page

Peptides that support the ASE (Splooce-specific):
SLEVFSCNCLK (MAXQUANT)

Alignment:
Uniprot       MASQLQNRLRSALALVTGAGSGIGRAVSVRLAGEGATVAACDLDRAAAQETVRLLGGPGSKEGPPRGNHAAFQADVSEARAARCLLEQVQACFSRPPSVV
Splooce       MASQLQNRLRSALALVTGAGSGIGRAVSVRLAGEGATVAACDLDRAAAQETVRLLGGPGSKEGPPRGNHAAFQADVSEARAARCLLEQVQACFSRPPSVV

Uniprot       VSCAGITQDEFLLHMSEDDWDKVIAVNLKGTFLVTQAAAQALVSNGCRGSIINISSIVGKVGNVGQTNYAASKAGVIGLTQTAARELGRHGIRCNSVLPG
Splooce       VSCAGITQDEFLLHMSEDDWDKVIAVNLKGTFLVTQAAAQALVSNGCRGSIINISSIVGKVGNVGQTNYAASKAGVIGLTQTAARELGRHGIRCNSVLPG

Uniprot       FIATPMTQKVPQKVVDKITEMIP-----------------------MGHLGDP---EDVADVVAFLASEDS---GYITGTSVEVTGGLFM-----
Splooce       FIATPMTQKVPQKVVDKMWQMWSHSWHLKIVDTSQGPQWKSLEVFSCNCLKDPGLCSPPHHSAWPPADEDSKFPGYKRGGSVWFRNAEYGKQGCL

----------------------------------------------------------------------------------------------------

O95163 (Uniprot)	versus
NM_003640#(-s-s-s-s-s-:9_I1910966887017) (Splooce)

For more details about the Alternative Splicing Event -> Link to Splooce page

Peptides that support the ASE (Splooce-specific):
FFINDIERR (MAXQUANT)

Alignment:
Uniprot       MRNLKLFRTLEFRDIQGPGNPQCFSLRTEQGTVLIGSEHGLIEVDPVSREVKNEVSLVAEGFLPEDGSGRIVGVQDLLDQESVCVATASGDVILCSLSTQ
Splooce       MRNLKLFRTLEFRDIQGPGNPQCFSLRTEQGTVLIGSEHGLIEVDPVSREVKNEVSLVAEGFLPEDGSGRIVGVQDLLDQESVCVATASGDVILCSLSTQ

Uniprot       QLECVGSVASGISVMSWSPDQELVLLATGQQTLIMMTKDFEPILEQQIHQDDFGESKFITVGWGRKETQFHGSEGRQAAFQMQMHESALPWDDHRPQVTW
Splooce       QLECVGSVASGISVMSWSPDQELVLLATGQQTLIMMTKDFEPILEQQIHQDDFGESKFITVGWGRKETQFHGSEGRQAAFQMQMHESALPWDDHRPQVTW

Uniprot       RGDGQFFAVSVVCPETGARKVRVWNREFALQSTSEPVAGLGPALAWKPSGSLIASTQDKPNQQDIVFFEKNGLLHGHFTLPFLKDEVKVNDLLWNADSSV
Splooce       RGDGQFFAVSVVCPETGARKVRVWNREFALQSTSEPVAGLGPALAWKPSGSLIASTQDKPNQQDIVFFEKNGLLHGHFTLPFLKDEVKVNDLLWNADSSV

Uniprot       LAVWLEDLQREESSIPKTCVQLWTVGNYHWYLKQSLSFSTCGKSKIVSLMWDPVTPYRLHVLCQGWHYLAYDWHWTTDRSVGDNSSDLSNVAVIDGNRVL
Splooce       LAVWLEDLQREESSIPKTCVQLWTVGNYHWYLKQSLSFSTCGKSKIVSLMWDPVTPYRLHVLCQGWHYLAYDWHWTTDRSVGDNSSDLSNVAVIDGNRVL

Uniprot       VTVFRQTVVPPPMCTYQLLFPHPVNQVTFLAHPQKSNDLAVLDASNQISVYKCGDCPSADPTVKLGAVGGSGFKVCLRTPHLEKRYKIQFENNEDQDVNP
Splooce       VTVFRQTVVPPPMCTYQLLFPHPVNQVTFLAHPQKSNDLAVLDASNQISVYKCGDCPSADPTVKLGAVGGSGFKVCLRTPHLEKRYKIQFENNEDQDVNP

Uniprot       LKLGLLTWIEEDVFLAVSHSEFSPRSVIHHLTAASSEMDEEHGQLNVSSSAAVDGVIISLCCNSKTKSVVLQLADGQIFKYLWESPSLAIKPWKNSGGFP
Splooce       LKLGLLTWIEEDVFLAVSHSEFSPRSVIHHLTAASSEMDEEHGQLNVSSSAAVDGVIISLCCNSKTKSVVLQLADGQIFKYLWESPSLAIKPWKNSGGFP

Uniprot       VRFPYPCTQTELAMIGEEECVLGLTDRCRFFINDIEVASNITSFAVYDEFLLLTTHSHTCQCFCLRDASFKTLQAGLSSNHVSHGEVLRKVERGSRIVTV
Splooce       VRFPYPCTQTELAMIGEEECVLGLTDRCRFFINDIERRR------CHEDHVPCTSY---QQCLPVQGS--------------------------------

Uniprot       VPQDTKLVLQMPRGNLEVVHHRALVLAQIRKWLDKLMFKEAFECMRKLRINLNLIYDHNPKVFLGNVETFIKQIDSVNHINLFFTELKEEDVTKTMYPAP
Splooce       ----------------------------------------------------------------------------------------------------

Uniprot       VTSSVYLSRDPDGNKIDLVCDAMRAVMESINPHKYCLSILTSHVKKTTPELEIVLQKVHELQGNAPSDPDAVSAEEALKYLLHLVDVNELYDHSLGTYDF
Splooce       ----------------------------------------------------------------------------------------------------

Uniprot       DLVLMVAEKSQKDPKEYLPFLNTLKKMETNYQRFTIDKYLKRYEKAIGHLSKCGPEYFPECLNLIKDKNLYNEALKLYSPSSQQYQDISIAYGEHLMQEH
Splooce       ----------------------------------------------------------------------------------------------------

Uniprot       MYEPAGLMFARCGAHEKALSAFLTCGNWKQALCVAAQLNFTKDQLVGLGRTLAGKLVEQRKHIDAAMVLEECAQDYEEAVLLLLEGAAWEEALRLVYKYN
Splooce       ----------------------------------------------------------------------------------------------------

Uniprot       RLDIIETNVKPSILEAQKNYMAFLDSQTATFSRHKKRLLVVRELKEQAQQAGLDDEVPHGQESDLFSETSSVVSGSEMSGKYSHSNSRISARSSKNRRKA
Splooce       ----------------------------------------------------------------------------------------------------

Uniprot       ERKKHSLKEGSPLEDLALLEALSEVVQNTENLKDEVYHILKVLFLFEFDEQGRELQKAFEDTLQLMERSLPEIWTLTYQQNSATPVLGPNSTANSIMASY
Splooce       ----------------------------------------------------------------------------------------------------

Uniprot       QQQKTSVPVLDAELFIPPKINRRTQWKLSLLD
Splooce       --------------------------------

----------------------------------------------------------------------------------------------------

B7Z840 (Uniprot)	versus
NM_005051#(-s-s-s-s-s-s-s-:3_Q8416589614166) (Splooce)

For more details about the Alternative Splicing Event -> Link to Splooce page

Peptides that support the ASE (Splooce-specific):
HHYTWWMQH (MAXQUANT)

Alignment:
Uniprot       MAALDSLSLFTSLGLSEQKARETLKNSALSAQLREAATQAQQTLGSTIDKATGILLYGLASRLRDTRRLSFLVSYIASKKIHTEPQLSAALEYVRSHPLD
Splooce       MAALDSLSLFTSLGLSEQKARETLKNSALSAQLREAATQAQQTLGSTIDKATGILLYGLASRLRDTRRLSFLVSYIASKKIHTEPQLSAALEYVRSHPLD

Uniprot       PIDTVDFERECGVGVIVTPEQIEEAVEAAINRHRPQLLVERYHFNMGLLMGEARAVLKWADGKMIKNEVDMQVLHLLGPKLEADLEKKFKVAKARLEETD
Splooce       PIDTVDFERECGVGVIVTPEQIEEAVEAAINRHRPQLLVERYHFNMGLLMGEARAVLKWADGKMIKNEVDMQVLHLLGPKLEADLEKKFKVAKARLEETD

Uniprot       RRTAKDVVENGETADQTLSLMEQLRGEALKFHKPGENYKTPGYVVTPHTMNLLKQHLEITGGQVRTRFPPEPNGILHIGHAKAINFNFGYAKANNGICFL
Splooce       RRTAKDVVENGETADQTLSLMEQLRGEALKFHKPGENYKTPGYVVTPHTMNLLKQHLEITGGQVRTRFPPEPNGILHIGHAKAINFNFGYAKANNGICFL

Uniprot       RFDDTNPEKEEAKFFTAICDMVAWLGYTPYKVTYASDYFDQLYAWAVELIRRGLAYVCHQRGEELKGHNTLPSPWRDRPMEESLLLFEAMRKGKFSEGEA
Splooce       RFDDTNPEKEEAKFFTAICDMVAWLGYTPYKVTYASDYFDQLYAWAVELIRRGLAYVCHQRGEELKGHNTLPSPWRDRPMEESLLLFEAMRKGKFSEGEA

Uniprot       TLRMKLVMEDGKMDPVAYRVKYTPHHRTGDKWCIYPTYDYTHCLCDSIEHITHSLCTKEFQARRSSYFWLCNALDVYCPVQWEYGRLNLHYAVVSKRKIL
Splooce       TLRMKLVMEDGKMDPVAYRVKYTPHHRTGDKWCIYPTYDYTHCLCDSIEHITHSLCTKEFQARHHYTWWMQH----------------------------

Uniprot       QLVATGAVRDWDDPRLFTLTALRRRGFPPEAINNFCARVGVTVAQTTMEPHLLEACVRDVLNDTAPRAMAVLESLRVIITNFPAAKSLDIQVPNFPADET
Splooce       ----------------------------------------------------------------------------------------------------

Uniprot       KGFHQVPFAPIVFIERTDFKEEPEPGFKRLAWGQPVGLRHTGYVIELQHVVKGPSGCVESLEVTCRRADAGEKPKAFIHWVSQPLMCEVRLYERLFQHKN
Splooce       ----------------------------------------------------------------------------------------------------

Uniprot       PEDPTEVPGGFLSDLNLASLHVVDAALVDCSVALAKPFDKFQFERLGYFSVDPDSHQGKLVFNRTVTLKEDPGKV
Splooce       ---------------------------------------------------------------------------

----------------------------------------------------------------------------------------------------

P43121 (Uniprot)	versus
NM_006500#(-s-s-s-:11_M8339356052917) (Splooce)

For more details about the Alternative Splicing Event -> Link to Splooce page

Peptides that support the ASE (Splooce-specific):
VLSTLNVLVTPELLETGVECTASNDLGKNTSILFLELGR (MAXQUANT)

Alignment:
Uniprot       MGLPRLVCAFLLAACCCCPRVAGVPGEAEQPAPELVEVEVGSTALLKCGLSQSQGNLSHVDWFSVHKEKRTLIFRVRQGQGQSEPGEYEQRLSLQDRGAT
Splooce       MGLPRLVCAFLLAACCCCPRVAGVPGEAEQPAPELVEVEVGSTALLKCGLSQSQGNLSHVDWFSVHKEKRTLIFRVRQGQGQSEPGEYEQRLSLQDRGAT

Uniprot       LALTQVTPQDERIFLCQGKRPRSQEYRIQLRVYKAPEEPNIQVNPLGIPVNSKEPEEVATCVGRNGYPIPQVIWYKNGRPLKEEKNRVHIQSSQTVESSG
Splooce       LALTQVTPQDERIFLCQGKRPRSQEYRIQLRVYKAPEEPNIQVNPLGIPVNSKEPEEVATCVGRNGYPIPQVIWYKNGRPLKEEKNRVHIQSSQTVESSG

Uniprot       LYTLQSILKAQLVKEDKDAQFYCELNYRLPSGNHMKESREVTVPVFYPTEKVWLEVEPVGMLKEGDRVEIRCLADGNPPPHFSISKQNPSTREAEEETTN
Splooce       LYTLQSILKAQLVKEDKDAQFYCELNYRLPSGNHMKESREVTVPVFYPTEKVWLEVEPVGMLKEGDRVEIRCLADGNPPPHFSISKQNPSTREAEEETTN

Uniprot       DNGVLVLEPARKEHSGRYECQGLDLDTMISLLSEPQELLVNYVSDVRVSPAAPERQEGSSLTLTCEAESSQDLEFQWLREETGQVLERGPVLQLHDLKRE
Splooce       DNGVLVLEPARKEHSGRYECQGLDLDTMISLLSEPQELLVNYVSDVRVSPAAPERQEGSSLTLTCEAESSQDLEFQWLREETGQVLERGPVLQLHDLKRE

Uniprot       AGGGYRCVASVPSIPGLNRTQLVNVAIFGPPWMAFKERKVWVKENMVLNLSCEASGHPRPTISWNVNGTASEQDQDPQRVLSTLNVLVTPELLETGVECT
Splooce       AGGGYRCVASVPSIPGLNRTQLVNVAIFGPPWMAFKERKVWVKENMVLNLSCEASGHPRPTISWNVNGTASEQDQDPQRVLSTLNVLVTPELLETGVECT

Uniprot       ASNDLGKNTSILFLELVNLTTLTPDSNTTTGLSTSTASPHTRANSTSTERKLPEPESRGVVIVAVIVCILVLAVLGAVLYFLYKKGKLPCRRSGKQEITL
Splooce       ASNDLGKNTSILFLELG------REIHRSEALAPNHFS------------------------------------------------SLPC----------

Uniprot       PPSRKSELVVEVKSDKLPEEMGLLQGSSGDKRAPGDQGEKYIDLRH
Splooce       -----------LDHSQLPAHSSLSQSLQRD----------------

----------------------------------------------------------------------------------------------------

Q9UQB8 (Uniprot)	versus
NM_006340#(-s-s-s-s-s-:17_B1931242279839) (Splooce)

For more details about the Alternative Splicing Event -> Link to Splooce page

Peptides that support the ASE (Splooce-specific):
IPLPTSS (MAXQUANT)

Alignment:
Uniprot       MSLSRSEEMHRLTENVYKTIMEQFNPSLRNFIAMGKNYEKALAGVTYAAKGYFDALVKMGELASESQGSKELGDVLFQMAEVHRQIQNQLEEMLKSFHNE
Splooce       MSLSRSEEMHRLTENVYKTIMEQFNPSLRNFIAMGKNYEKALAGVTYAAKGYFDALVKMGELASESQGSKELGDVLFQMAEVHRQIQNQLEEMLKSFHNE

Uniprot       LLTQLEQKVELDSRYLSAALKKYQTEQRSKGDALDKCQAELKKLRKKSQGSKNPQKYSDKELQYIDAISNKQGELENYVSDGYKTALTEERRRFCFLVEK
Splooce       LLTQLEQKVELDSRYLSAALKKYQTEQRSKGDALDKCQAELKKLRKKSQGSKNPQKYSDKELQYIDAISNKQGELENYVSDGYKTALTEERRRFCFLVEK

Uniprot       QCAVAKNSAAYHSKGKELLAQKLPLWQQACADPSKIPERAVQLMQQVASNGATLPSALSASKSNLVISDPIPGAKPLPVPPELAPFVGRMSAQESTPIMN
Splooce       QCAVAKNSAAYHSKGKELLAQKLPLWQQACADPSKIPERAVQLMQQVASNGATLPSALSASKSNLVISDPIPGAKPLPVPPELAPFVG============

Uniprot       GVTGPDGEDYSPWADRKAAQPKSLSPPQSQSKLSDSYSNTLPVRKSVTPKNSYATTENKTLPRSSSMAAGLERNGRMRVKAIFSHAAGDNSTLLSFKEGD
Splooce       ===============RRCGSGQILSR---------------------------LTRVRIPLPTSS-----------------------------------

Uniprot       LITLLVPEARDGWHYGESEKTKMRGWFPFSYTRVLDSDGSDRLHMSLQQGKSSSTGNLLDKDDLAIPPPDYGAASRAFPAQTASGFKQRPYSVAVPAFSQ
Splooce       ----------------------------------------------------------------------------------------------------

Uniprot       GLDDYGARSMSSADVEVARF
Splooce       --------------------

----------------------------------------------------------------------------------------------------

P31749 (Uniprot)	versus
NM_005163#(f-:14_A1328526230363) (Splooce)

For more details about the Alternative Splicing Event -> Link to Splooce page

Peptides that support the ASE (Splooce-specific):
TMNEFEYLKLLGK (MAXQUANT)

Alignment:
Uniprot       MSDVAIVKEGWLHKRGEYIKTWRPRYFLLKNDGTFIGYKERPQDVDQREAPLNNFSVAQCQLMKTERPRPNTFIIRCLQWTTVIERTFHVETPEEREEWT
Splooce       --------------------MWR----LLRSG--------------------------------------------------------------------

Uniprot       TAIQTVADGLKKQEEEEMDFRSGSPSDNSGAEEMEVSLAKPKHRVTMNEFEYLKLLGKGTFGKVILVKEKATGRYYAMKILKKEVIVAKDEVAHTLTENR
Splooce       --------------------RSGQPPSR-----------------TMNEFEYLKLLGKGTFGKVILVKEKATGRYYAMKILKKEVIVAKDEVAHTLTENR

Uniprot       VLQNSRHPFLTALKYSFQTHDRLCFVMEYANGGELFFHLSRERVFSEDRARFYGAEIVSALDYLHSEKNVVYRDLKLENLMLDKDGHIKITDFGLCKEGI
Splooce       VLQNSRHPFLTALKYSFQTHDRLCFVMEYANGGELFFHLSRERVFSEDRARFYGAEIVSALDYLHSEKNVVYRDLKLENLMLDKDGHIKITDFGLCKEGI

Uniprot       KDGATMKTFCGTPEYLAPEVLEDNDYGRAVDWWGLGVVMYEMMCGRLPFYNQDHEKLFELILMEEIRFPRTLGPEAKSLLSGLLKKDPKQRLGGGSEDAK
Splooce       KDGATMKTFCGTPEYLAPEVLEDNDYGRAVDWWGLGVVMYEMMCGRLPFYNQDHEKLFELILMEEIRFPRTLGPEAKSLLSGLLKKDPKQRLGGGSEDAK

Uniprot       EIMQHRFFAGIVWQHVYEKKLSPPFKPQVTSETDTRYFDEEFTAQMITITPPDQDDSMECVDSERRPHFPQFSYSASGTA
Splooce       EIMQHRFFAGIVWQHVYEKKLSPPFKPQVTSETDTRYFDEEFTAQMITITPPDQDDSMECVDSERRPHFPQFSYSASGTA

----------------------------------------------------------------------------------------------------

Q96HE7 (Uniprot)	versus
NM_014584#(f-:14_E8179471848009) (Splooce)

For more details about the Alternative Splicing Event -> Link to Splooce page

Peptides that support the ASE (Splooce-specific):
RPLNPLASGQENTFYSWLEGLCVEK (MAXQUANT)

Alignment:
Uniprot       MGRGWGFLFGLLGAVWLLSSGHGEEQPPETAAQRCFCQVSGYLDDCTCDVETIDRFNNYRLFPRLQKLLESDYFRYYKVNLKRPCPFWNDISQCGRRDCA
Splooce       MGRGWGFLFGLLGAVWLLSSGHGEEQPPETAAQRCFCQVSGYLDDCTCDVETIDRFNNYRLFPRLQKLLESDYFRYYKVNLKRPCPFWNDISQCGRRDCA

Uniprot       VKPCQSDEVPDGIKSASYKYSEEANNLIEECEQAERLGAVDESLSEETQKAVLQWTKHDDSSDNFCEADDIQSPEAEYVDLLLNPERYTGYKGPDAWKIW
Splooce       VKPCQSDEVPDGIKSASYKYSEEANNLIEECEQAERLGAVDESLSEETQKAVLQWTKHDDSSDNFCEADDIQSPEAEYVDLLLNPERYTGYKGPDAWKIW

Uniprot       NVIYEENCFKPQTIKRPLNPLASGQGKLLIEHF--LQLARRSLCRKKS---------------ILQTYIWPTCKH-------------------------
Splooce       NVIYEENCFKPQTIKRPLNPLASGQENTFYSWLEGLCVEKRAFYRLISGLHASINVHLSARYLLQETWLEKKWGHNITEFQQRFDGILTEGEGPRRLKNL

Uniprot       ----------------------------------------------------------------------------------------------------
Splooce       YFLYLIELRALSKVLPFFERPDFQLFTGNKIQDEENKMLLLEILHEIKSFPLHFDENSFFAGDKKEAHKLKEDFRLHFRNISRIMDCVGCFKCRLWGKLQ

Uniprot       ----------------------------------------------------------------
Splooce       TQGLGTALKILFSEKLIANMPESGPSYEFHLTRQEIVSLFNAFGRISTSVKELENFRNLLQNIH

----------------------------------------------------------------------------------------------------

Q86UP2 (Uniprot)	versus
NM_001079522#(-s-s-:14_K9167787760973) (Splooce)

For more details about the Alternative Splicing Event -> Link to Splooce page

Peptides that support the ASE (Splooce-specific):
NNSYGEWLHGFEK (MAXQUANT)

Alignment:
Uniprot       MEFYESAYFIVLIPSIVITVIFLFFWLFMKETLYDEVLAKQKREQKLIPTKTDKKKAEKKKNKKKEIQNGNLHESDSESVPRDFKLSDALAVEDDQVAPV
Splooce       MEFYESAYFIVLIPSIVITVIFLFFWLFMKETLYDEVLAKQKREQKLIPTKTDKKKAEKKKNKKKEIQNGNLHESDSESVPRDFKLSDALAVEDDQVAPV

Uniprot       PLNVVETSSSVRERKKKEKKQKPVLEEQVIKESDASKIPGKKVEPVPVTKQPTPPSEAAASKKKPGQKKSKNGSDDQDKKVETLMVPSKRQEALPLHQET
Splooce       PLNVVETSSSVRERKKKEKKQKPVLEEQVIKESDASKIPGKKVEPVPVTKQPTPPSEAAASKKKPGQKKSKNGSDDQDKKVETLMVPSKRQEALPLHQET

Uniprot       KQESGSGKKKASSKKQKTENVFVDEPLIHATTYIPLMDNADSSPVVDKREVIDLLKPDQVEGIQKSGTKKLKTETDKENAEVKFKDFLLSLKTMMFSEDE
Splooce       KQESGSGKKKASSKKQKTENVFVDEPLIHATTYIPLMDNADSSPVVDKREVIDLLKPDQVEGIQKSGTKKLKTETDKENAEVKFKDFLLSLKTMMFSEDE

Uniprot       ALCVVDLLKEKSGVIQDALKKSSKGELTTLIHQLQEKDKLLAAVKEDAAATKDRCKQLTQEMMTEKERSNVVITRMKDRIGTLEKEHNVFQNKIHVSYQE
Splooce       ALCVVDLLKEKSGVIQDALKKSSKGELTTLIHQLQEKDKLLAAVKEDAAATKDRCKQLTQEMMTEKERSNVVITRMKDRIGTLEKEHNVFQNKIHVSYQE

Uniprot       TQQMQMKFQQVREQMEAEIAHLKQENGILRDAVSNTTNQLESKQSAELNKLRQDYARLVNELTEKTGKLQQEEVQKKNAEQAATQLKVQLQEAERRWEEV
Splooce       TQQMQMKFQQVREQMEAEIAHLKQENGILRDAVSNTTNQLESKQSAELNKLRQDYARLVNELTEKTGKLQQEEVQKKNAEQAATQLKVQLQEAERRWEEV

Uniprot       QSYIRKRTAEHEAAQQDLQSKFVAKENEVQSLHSKLTDTLVSKQQLEQRLMQLMESEQKRVNKEESLQMQVQDILEQNEALKAQIQQFHSQIAAQTSASV
Splooce       QSYIRKRTAEHEAAQQDLQSKFVAKENEVQSLHSKLTDTLVSKQQLEQRLMQLMESEQKRVNKEESLQMQVQDILEQNEALKAQIQQFHSQIAAQTSASV

Uniprot       LAEELHKVIAEKDKQIKQTEDSLASERDRLTSKEEELKDIQNMNFLLKAEVQKLQALANEQAAAAHELEKMQQSVYVKDDKIRLLEEQLQHEISNKMEEF
Splooce       LAEELHKVIAEKDKQIKQTEDSLASERDRLTSKEEELKDIQNMNFLLKAEVQKLQALANEQAAAAHELEKMQQSVYVKDDKIRLLEEQLQHEISNKMEEF

Uniprot       KILNDQNKALKSEVQKLQTLVSEQPNKDVVEQMEKCIQEKDEKLKTVEELLETGLIQVATKEEELNAIRTENSSLTKEVQDLKAKQNDQVSFASLVEELK
Splooce       KILNDQNKALKSEVQKLQTLVSEQPNKDVVEQMEKCIQEKDEKLKTVEELLETGLIQVATKEEELNAIRTENSSLTKEVQDLKAKQNDQVSFASLVEELK

Uniprot       KVIHEKDGKIKSVEELLEAELLKVANKEKTVQLSITSKVQELQNLLKGKEEQMNTMKAVLEEKEKDLANTGKWLQDLQEENESLKAHVQEVAQHNLKEAS
Splooce       KVIHEKDGKIKSVEELLEAELLKVANKEKTVQLSITSKVQELQNLLKGKEEQMNTMKAVLEEKEKDLANTGKWLQDLQEENESLKAHVQEVAQHNLKEAS

Uniprot       SASQFEELEIVLKEKENELKRLEAMLKERESDLSSKTQLLQDVQDENKLFKSQIEQLKQQNYQQASSFPPHEELLKVISEREKEISGLWNELDSLKDAVE
Splooce       SASQFEELEIVLKEKENELKRLEAMLKERESDLSSKTQLLQDVQDENKLFKSQIEQLKQQNYQQASSFPPHEELLKVISEREKEISGLWNELDSLKDAVE

Uniprot       HQRKKNNDLREKNWEAMEALASTEKMLQDKVNKTSKERQQQVEAVELEAKEVLKKLFPKVSVPSNLSYGEWLHGFEKKAKECMAGTSGSEEVKVLEHKLK
Splooce       HQRKKNN-----------------------------------------------------------SYGEWLHGFEKKAKECMAGTSGSEEVKVLEHKLK

Uniprot       EADEMHTLLQLECEKYKSVLAETEGILQKLQRSVEQEENKWKVKVDESHKTIKQMQSSFTSSEQELERLRSENKDIENLRREREHLEMELEKAEMERSTY
Splooce       EADEMHTLLQLECEKYKSVLAETEGILQKLQRSVEQEENKWKVKVDESHKTIKQMQSSFTSSEQELERLRSENKDIENLRREREHLEMELEKAEMERSTY

Uniprot       VTEVRELKAQLNETLTKLRTEQNERQKVAGDLHKAQQSLELIQSKIVKAAGDTTVIENSDVSPETESSEKETMSVSLNQTVTQLQQLLQAVNQQLTKEKE
Splooce       VTEVRELKAQLNETLTKLRTEQNERQKVAGDLHKAQQSLELIQSKIVKAAGDTTVIENSDVSPETESSEKETMSVSLNQTVTQLQQLLQAVNQQLTKEKE

Uniprot       HYQVLE
Splooce       HYQVLE

----------------------------------------------------------------------------------------------------

P0CG48 (Uniprot)	versus
NM_021009#(rr:12_U5754582962429) (Splooce)

For more details about the Alternative Splicing Event -> Link to Splooce page

Peptides that support the ASE (Splooce-specific):
TLTGKTITLEVEPSDTIENNVK (MAXQUANT)
TITLEVEPSDTIENNVKAK (MAXQUANT)

Alignment:
Uniprot       MQIFVKTLTGKTITLEVEPSDTIENVKAKIQDKEGIPPDQQRLIFAGKQLEDGRTLSDYNIQKESTLHLVLRLRGGMQIFVKTLTGKTITLEVEPSDTIE
Splooce       ----------------------------------------------------------------------------MQIFVKTLTGKTITLEVEPSDTIE

Uniprot       NVKAKIQDKEGIPPDQQRLIFAGKQLEDGRTLSDYNIQKESTLHLVLRLRGGMQIFVKTLTGKTITLEVEPSDTIENVKAKIQDKEGIPSDQQRLIFAGK
Splooce       NVKAKIQDKEGIPPDQQRLIFAGKQLEDGRTLSDYNIQKESTLHLVLRLRGGMQIFVKTLTGKTITLEVEPSDTIEN-----------------------

Uniprot       QLEDGRTLSDYNIQKESTLHLVLRLRGGMQIFVKTLTGKTITLEVEPSDTIENVKAKIQDKEGIPPDQQRLIFAGKQLEDGRTLSDYNIQKESTLHLVLR
Splooce       ----------------------------------------------------NVKAKIQDKEGIPPDQQRLIFAGKQLEDGRTLSDYNIQKESTLHLVLR

Uniprot       LRGGMQIFVKTLTGKTITLEVEPSDTIENVKAKIQDKEGIPPDQQRLIFAGKQLEDGRTLSDYNIQKESTLHLVLRLRGGMQIFVKTLTGKTITLEVEPS
Splooce       LRGGMQIFVKTLTGKTITLEVEPSDTIENVKAKIQDKEGIPPDQQRLIFAGKQLEDGRTLSDYNIQKESTLHLVLRLRGGMQIFVKTLTGKTITLEVEPS

Uniprot       DTIENVKAKIQDKEGIPPDQQRLIFAGKQLEDGRTLSDYNIQKESTLHLVLRLRGGMQIFVKTLTGKTITLEVEPSDTIENVKAKIQDKEGIPPDQQRLI
Splooce       DTIENVKAKIQDKEGIPPDQQRLIFAGKQLEDGRTLSDYNIQKESTLHLVLRLRGGMQIFVKTLTGKTITLEVEPSDTIENVKAKIQDKEGIPPDQQRLI

Uniprot       FAGKQLEDGRTLSDYNIQKESTLHLVLRLRGGMQIFVKTLTGKTITLEVEPSDTIENVKAKIQDKEGIPPDQQRLIFAGKQLEDGRTLSDYNIQKESTLH
Splooce       FAGKQLEDGRTLSDYNIQKESTLHLVLRLRGGMQIFVKTLTGKTITLEVEPSDTIENVKAKIQDKEGIPPDQQRLIFAGKQLEDGRTLSDYNIQKESTLH

Uniprot       LVLRLRGGMQIFVKTLTGKTITLEVEPSDTIENVKAKIQDKEGIPPDQQRLIFAGKQLEDGRTLSDYNIQKESTLHLVLRLRGGV
Splooce       LVLRLRGGMQIFVKTLTGKTITLEVEPSDTIENVKAKIQDKEGIPPDQQRLIFAGKQLEDGRTLSDYNIQKESTLHLVLRLRGGV

----------------------------------------------------------------------------------------------------

E7EUT4 (Uniprot)	versus
NM_002046#(f-:12_G1887555240001) (Splooce)

For more details about the Alternative Splicing Event -> Link to Splooce page

Peptides that support the ASE (Splooce-specific):
AHLQGGAK (MAXQUANT + PEAKS)

Alignment:
Uniprot       MGKVKVGVNGFGRIGRLVTRAAFNSGKVDIVAINDPFIDLNYMVYMFQYDSTHGKFHGTVKAENGKLVINGNPITIFQERDPSKIKWGDAGAEYVVESTG
Splooce       MGKVKVGVNGFGRIGRLVTRAAFNSGKVDIVAINDPFIDLNYMVYMFQYDSTHGKFHGTVKAENGKLVINGNPITIFQERDPSKIKWGDAGAEYVVESTG

Uniprot       VFTTMEKAGAHLQGGAKRVIISAPSADAPMFVMGVNHEKYDNSLKIISNASCTTNCLAPLAKVIHDNFGIVEGLMTTVHAITATQKTVDGPSGKLWRDGR
Splooce       VFTTMEKA--HLQGGAKRVIISAPSADAPMFVMGVNHEKYDNSLKIISNASCTTNCLAPLAKVIHDNFGIVEGLMTTVHAITATQKTVDGPSGKLWRDGR

Uniprot       GALQNIIPASTGAAKAVGKVIPELNGKLTGMAFRVPTANVSVVDLTCRLEKPAKYDDIKKVVKQASEGPLKGILGYTEHQVVSSDFNSDTHSSTFDAGAG
Splooce       GALQNIIPASTGAAKAVGKVIPELNGKLTGMAFRVPTANVSVVDLTCRLEKPAKYDDIKKVVKQASEGPLKGILGYTEHQVVSSDFNSDTHSSTFDAGAG

Uniprot       IALNDHFVKLISWYDNEFGYSNRVVDLMAHMASKE
Splooce       IALNDHFVKLISWYDNEFGYSNRVVDLMAHMASKE

----------------------------------------------------------------------------------------------------

Q9ULW0 (Uniprot)	versus
NM_012112#(-t:20_T3413801531271) (Splooce)

For more details about the Alternative Splicing Event -> Link to Splooce page

Peptides that support the ASE (Splooce-specific):
SVAVQEPFQLATEKR (MAXQUANT)

Alignment:
Uniprot       MSQVKSSYSYDAPSDFINFSSLDDEGDTQNIDSWFEEKANLENKLLGKNGTGGLFQGKTPLRKANLQQAIVTPLKPVDNTYYKEAEKENLVEQSIPSNAC
Splooce       MSQVKSSYSYDAPSDFINFSSLDDEGDTQNIDSWFEEKANLENKLLGKNGTGGLFQGKTPLRKANLQQAIVTPLKPVDNTYYKEAEKENLVEQSIPSNAC

Uniprot       SSLEVEAAISRKTPAQPQRRSLRLSAQKDLEQKEKHHVKMKAKRCATPVIIDEILPSKKMKVSNNKKKPEEEGSAHQDTAEKNASSPEKAKGRHTVPCMP
Splooce       SSLEVEAAISRKTPAQPQRRSLRLSAQKDLEQKEKHHVKMKAKRCATPVIIDEILPSKKMKVSNNKKKPEEEGSAHQDTAEKNASSPEKAKGRHTVPCMP

Uniprot       PAKQKFLKSTEEQELEKSMKMQQEVVEMRKKNEEFKKLALAGIGQPVKKSVSQVTKSVDFHFRTDERIKQHPKNQEEYKEVNFTSELRKHPSSPARVTKG
Splooce       PAKQKFLKSTEEQELEKSMKMQQEVVEMRKKNEEFKKLALAGIGQPVKKSVSQVTKSVDFHFRTDERIKQHPKNQEEYKEVNFTSELRKHPSSPARVTKG

Uniprot       CTIVKPFNLSQGKKRTFDETVSTYVPLAQQVEDFHKRTPNRYHLRSKKDDINLLPSKSSVTKICRDPQTPVLQTKHRARAVTCKSTAELEAEELEKLQQY
Splooce       CTIVKPFNLSQGKKRTFDETVSTYVPLAQQVEDFHKRTPNRYHLRSKKDDINLLPSKSSVTKICRDPQTPVLQTKHRARAVTCKSTAELEAEELEKLQQY

Uniprot       KFKARELDPRILEGGPILPKKPPVKPPTEPIGFDLEIEKRIQERESKKKTEDEHFEFHSRPCPTKILEDVVGVPEKKVLPITVPKSPAFALKNRIRMPTK
Splooce       KFKARELDPRILEGGPILPKKPPVKPPTEPIGFDLEIEKRIQERESKKKTEDEHFEFHSRPCPTKILEDVVGVPEKKVLPITVPKSPAFALKNRIRMPTK

Uniprot       EDEEEDEPVVIKAQPVPHYGVPFKPQIPEARTVEICPFSFDSRDKERQLQKEKKIKELQKGEVPKFKALPLPHFDTINLPEKKVKNVTQIEPFCLETDRR
Splooce       EDEEEDEPVVIKAQPVPHYGVPFKPQIPEARTVEICPFSFDSRDKERQLQKEKKIKELQKGEVPKFKALPLPHFDTINLPEKKVKNVTQIEPFCLETDRR

Uniprot       GALKAQTWKHQLEEELRQQKEAACFKARPNTVISQEPFVPKKEKKSVAEGLSGSLVQEPFQLATEKRAKERQELEKRMAEVEAQKAQQLEEARLQEEEQK
Splooce       GALKAQTWKHQLEEELRQQKEAACFKARPNTVISQEPFVPKKEKKSVA-------VQEPFQLATEKRAKERQELEKRMAEVEAQKAQQLEEARLQEEEQK

Uniprot       KEELARLRRELVHKANPIRKYQGLEIKSSDQPLTVPVSPKFSTRFHC
Splooce       KEELARLRRELVHKANPIRKYQGLEIKSSDQPLTVPVSPKFSTRFHC

----------------------------------------------------------------------------------------------------

P51991 (Uniprot)	versus
NM_194247#(-s-:2_H2862992890305) (Splooce)

For more details about the Alternative Splicing Event -> Link to Splooce page

Peptides that support the ASE (Splooce-specific):
GGYGGGGPGYGNQGGGYGGGGGYDGYNEGGNFGGGGYGSGGGSGGYGSRR (MAXQUANT)

Alignment:
Uniprot       MEVKPPPGRPQPDSGRRRRRRGEEGHDPKEPEQLRKLFIGGLSFETTDDSLREHFEKWGTLTDCVVMRDPQTKRSRGFGFVTYSCVEEVDAAMCARPHKV
Splooce       MEVKPPPGRPQPDSGRRRRRRGEEGHDPKEPEQLRKLFIGGLSFETTDDSLREHFEKWGTLTDCVVMRDPQTKRSRGFGFVTYSCVEEVDAAMCARPHKV

Uniprot       DGRVVEPKRAVSREDSVKPGAHLTVKKIFVGGIKEDTEEYNLRDYFEKYGKIETIEVMEDRQSGKKRGFAFVTFDDHDTVDKIVVQKYHTINGHNCEVKK
Splooce       DGRVVEPKRAVSREDSVKPGAHLTVKKIFVGGIKEDTEEYNLRDYFEKYGKIETIEVMEDRQSGKKRGFAFVTFDDHDTVDKIVVQKYHTINGHNCEVKK

Uniprot       ALSKQEMQSAGSQRGRGGGSGNFMGRGGNFGGGGGNFGRGGNFGGRGGYGGGGGGSRGSYGGGDGGYNGFGGDGGNYGGGPGYSSRGGYGGGGPGYGNQG
Splooce       ALSKQEMQSAGSQRGRGGGSGNFMGRGGNFGGGGGNFGRGGNFGGRGGYGGGGGGSRGSYGGGDGGYNGFGGDGGNYGGGPGYSSRGGYGGGGPGYGNQG

Uniprot       GGYGGGGGYDGYNEGGNFGGGNYGGGGNYNDFGNYSGQQQSNYGPMKGGSFGGRSSGSPYGGGYGSGGGSGGYGSRRF
Splooce       GGYGGGGGYDGYNEGGNFGGG-----------------------------------------GYGSGGGSGGYGSRRF

----------------------------------------------------------------------------------------------------

F5H667 (Uniprot)	versus
NM_004318#(-s-s-:8_A2200811710940) (Splooce)

For more details about the Alternative Splicing Event -> Link to Splooce page

Peptides that support the ASE (Splooce-specific):
LHHDTDDVTYQVYEEQAVYEPLENEGIEITETNR (MAXQUANT)

Alignment:
Uniprot       MAQRKNAKSSGNSSSSGSGSGSTSAGSSSPGARRETKHGGHKNGRKGGLSGTSFFTWFMVIALLGVWTSVAVVWFDLVDYEEVLGKLGIYDADGDGDFDV
Splooce       MAQRKNAKSSGNSSSSGSGSGSTSAGSSSPGARRETKHGGHKNGRKGGLSGTSFFTWFMVIALLGVWTSVAVVWFDLVDYEEVLGKLGIYDADGDGDFDV

Uniprot       DDAKVLLGLKERSTSEPAVPPEEAEPHTEPEEQVPVEAEPQNIEDEAKEQIQSLLHEMVHAEHVEGEDLQQEDGPTGEPQQEDDEFLMATDVDDRFETLE
Splooce       DDAKVLLGLKERSTSEPAVPPEEAEPHTEPEEQVPVEAEPQNIEDEAKEQIQSLLHEMVHAEHVEGEDLQQEDGPTGEPQQEDDEFLMATDVDDRFETLE

Uniprot       PEVSHEETEHSYHVEETVSQDCNQDMEEMMSEQENPDSSEPVVEDERLHHDTDDVTYQVYEEQAVYEPLENEGIEITEVTAPPEDNPVEDSQVIVEEVSI
Splooce       PEVSHEETEHSYHVEETVSQDCNQDMEEMMSEQENPDSSEPVVEDERLHHDTDDVTYQVYEEQAVYEPLENEGIEIT-----------------------

Uniprot       FPVEEQQEVPPETNRKTDDPEQKAKVKKKKPKLLNKFDKTIKAELDAAEKLRKRGKIEEAVNAFKELVRKYPQSPRARYGKAQCEDDLAEKRRSNEVLRG
Splooce       -----------ETNRKTDDPEQKAKVKKKKPKLLNKFDKTIKAELDAAEKLRKRGKIEEAVNAFKELVRKYPQSPRARYGKAQCEDDLAEKRRSNEVLRG

Uniprot       AIETYQEVASLPDVPADLLKLSLKRRSDRQQFLGHMRGSLLTLQRLVQLFPNDTSLKNDLGVGYLLIGDNDNAKKVYEEVLSVTPNDGFAKVHYGFILKA
Splooce       AIETYQEVASLPDVPADLLKLSLKRRSDRQQFLGHMRGSLLTLQRLVQLFPNDTSLKNDLGVGYLLIGDNDNAKKVYEEVLSVTPNDGFAKVHYGFILKA

Uniprot       QNKIAESIPYLKEGIESGDPGTDDGRFYFHLGDAMQRVGNKEAYKWYELGHKRGHFASVWQRSLYNVNGLKAQPWWTPKETGYTELVKSLERNWKLIRDE
Splooce       QNKIAESIPYLKEGIESGDPGTDDGRFYFHLGDAMQRVGNKEAYKWYELGHKRGHFASVWQRSLYNVNGLKAQPWWTPKETGYTELVKSLERNWKLIRDE

Uniprot       GLAVMDKAKGLFLPEDENLREKGDWSQFTLWQQGRRNENACKGAPKTCTLLEKFPETTGCRRGQIKYSIMHPGTHVWPHTGPTNCRLRMHLGLVIPKEGC
Splooce       GLAVMDKAKGLFLPEDENLREKGDWSQFTLWQQGRRNENACKGAPKTCTLLEKFPETTGCRRGQIKYSIMHPGTHVWPHTGPTNCRLRMHLGLVIPKEGC

Uniprot       KIRCANETKTWEEGKVLIFDDSFEHEVWQDASSFRLIFIVDVWHPELTPQQRRSLPAI
Splooce       KIRCANETKTWEEGKVLIFDDSFEHEVWQDASSFRLIFIVDVWHPELTPQQRRSLPAI

----------------------------------------------------------------------------------------------------

E7EWW0 (Uniprot)	versus
NM_020314#(-s-:16_C732265999783) (Splooce)

For more details about the Alternative Splicing Event -> Link to Splooce page

Peptides that support the ASE (Splooce-specific):
PITDSSR (MAXQUANT)

Alignment:
Uniprot       MAVFPWHSRNRNYKAEFASCRLEAVPLEFGDYHPLKPITVTESKTKKVNRKGSTSSTSSSSSSSVVDPLSSVLDGTDPLSMFAATADPAALAAAMDSSRR
Splooce       MAVFPWHSRNRNYKAEFASCRLEAVPLEFGDYHPLKPIT--------------------------------------------------------DSSRR

Uniprot       KRDRDDNSVVGSDFEPWTNKRGEILARYTTTEKLSINLFMGSEKGKAGTATLAMSEKVRTRLEELDDFEEGSQKELLNLTQQDYVNRIEELNQSLKDAWA
Splooce       KRDRDDNSVVGSDFEPWTNKRGEILARYTTTEKLSINLFMGSEKGKAGTATLAMSEKVRTRLEELDDFEEGSQKELLNLTQQDYVNRIEELNQSLKDAWA

Uniprot       SDQKVKALKIVIQCSKLLSDTSVIQFYPSKFVLITDILDTFGKLVYERIFSMCVDSRSVLPDHFSPENANDTAKETCLNWFFKIASIRELIPRFYVEASI
Splooce       SDQKVKALKIVIQCSKLLSDTSVIQFYPSKFVLITDILDTFGKLVYERIFSMCVDSRSVLPDHFSPENANDTAKETCLNWFFKIASIRELIPRFYVEASI

Uniprot       LKCNKFLSKTGISECLPRLTCMIRGIGDPLVSVYARAYLCRVGMEVAPHLKETLNKNFFDFLLTFKQIHGDTVQNQLVVQGVELPSYLPLYPPAMDWIFQ
Splooce       LKCNKFLSKTGISECLPRLTCMIRGIGDPLVSVYARAYLCRVGMEVAPHLKETLNKNFFDFLLTFKQIHGDTVQNQLVVQGVELPSYLPLYPPAMDWIFQ

Uniprot       CISYHAPEALLTEMMERCKKLGNNALLLNSVMSAFRAEFIATRSMDFIGMIKECDESGFPKHLLFRSLGLNLALADPPESDRLQILNEAWKVITKLKNPQ
Splooce       CISYHAPEALLTEMMERCKKLGNNALLLNSVMSAFRAEFIATRSMDFIGMIKECDESGFPKHLLFRSLGLNLALADPPESDRLQILNEAWKVITKLKNPQ

Uniprot       DYINCAEVWVEYTCKHFTKREVNTVLADVIKHMTPDRAFEDSYPQLQLIIKKVIAHFHDFSVLFSVEKFLPFLDMFQKESVRVEVCKCIMDAFIKHQQEP
Splooce       DYINCAEVWVEYTCKHFTKREVNTVLADVIKHMTPDRAFEDSYPQLQLIIKKVIAHFHDFSVLFSVEKFLPFLDMFQKESVRVEVCKCIMDAFIKHQQEP

Uniprot       TKDPVILNALLHVCKTMHDSVNALTLEDEKRMLSYLINGFIKMVSFGRDFEQQLSFYVESRSMFCNLEPVLVQLIHSVNRLAMETRKVMKGNHSRKTAAF
Splooce       TKDPVILNALLHVCKTMHDSVNALTLEDEKRMLSYLINGFIKMVSFGRDFEQQLSFYVESRSMFCNLEPVLVQLIHSVNRLAMETRKVMKGNHSRKTAAF

Uniprot       VRACVAYCFITIPSLAGIFTRLNLYLHSGQVALANQCLSQADAFFKAAISLVPEVPKMINIDGKMRPSESFLLEFLCNFFSTLLIVPDHPEHGVLFLVRE
Splooce       VRACVAYCFITIPSLAGIFTRLNLYLHSGQVALANQCLSQADAFFKAAISLVPEVPKMINIDGKMRPSESFLLEFLCNFFSTLLIVPDHPEHGVLFLVRE

Uniprot       LLNVIQDYTWEDNSDEKIRIYTCVLHLLSAMSQETYLYHIDKVDSNDSLYGGDSKFLAENNKLCETVMAQILEHLKTLAKDEALKRQSSLGLSFFNSILA
Splooce       LLNVIQDYTWEDNSDEKIRIYTCVLHLLSAMSQETYLYHIDKVDSNDSLYGGDSKFLAENNKLCETVMAQILEHLKTLAKDEALKRQSSLGLSFFNSILA

Uniprot       HGDLRNNKLNQLSVNLWHLAQRHGCADTRTMVKTLEYIKKQSKQPDMTHLTELALRLPLQTRT
Splooce       HGDLRNNKLNQLSVNLWHLAQRHGCADTRTMVKTLEYIKKQSKQPDMTHLTELALRLPLQTRT

----------------------------------------------------------------------------------------------------

P50213 (Uniprot)	versus
NM_005530#(f-:15_I5612548113334) (Splooce)

For more details about the Alternative Splicing Event -> Link to Splooce page

Peptides that support the ASE (Splooce-specific):
QVQTVTLIPGDGIGPEISAAVMK (MAXQUANT)

Alignment:
Uniprot       MAGPAWISKVSRLLGAFHNPKQVTRGFTGGVQTVTLIPGDGIGPEISAAVMKIFDAAKAPIQWEERNVTAIQGPGGKWMIPSEAKESMDKNKMGLKGPLK
Splooce       MAGPAWISKVSRLLGAFHNPKQ--------VQTVTLIPGDGIGPEISAAVMKIFDAAKAPIQWEERNVTAIQGPGGKWMIPSEAKESMDKNKMGLKGPLK

Uniprot       TPIAAGHPSMNLLLRKTFDLYANVRPCVSIEGYKTPYTDVNIVTIRENTEGEYSGIEHVIVDGVVQSIKLITEGASKRIAEFAFEYARNNHRSNVTAVHK
Splooce       TPIAAGHPSMNLLLRKTFDLYANVRPCVSIEGYKTPYTDVNIVTIRENTEGEYSGIEHVIVDGVVQSIKLITEGASKRIAEFAFEYARNNHRSNVTAVHK

Uniprot       ANIMRMSDGLFLQKCREVAESCKDIKFNEMYLDTVCLNMVQDPSQFDVLVMPNLYGDILSDLCAGLIGGLGVTPSGNIGANGVAIFESVHGTAPDIAGKD
Splooce       ANIMRMSDGLFLQKCREVAESCKDIKFNEMYLDTVCLNMVQDPSQFDVLVMPNLYGDILSDLCAGLIGGLGVTPSGNIGANGVAIFESVHGTAPDIAGKD

Uniprot       MANPTALLLSAVMMLRHMGLFDHAARIEAACFATIKDGKSLTKDLGGNAKCSDFTEEICRRVKDLD
Splooce       MANPTALLLSAVMMLRHMGLFDHAARIEAACFATIKDGKSLTKDLGGNAKCSDFTEEICRRVKDLD

----------------------------------------------------------------------------------------------------

Q53EL6 (Uniprot)	versus
NM_014456#(-s-:10_P5140585263401) (Splooce)

For more details about the Alternative Splicing Event -> Link to Splooce page

Peptides that support the ASE (Splooce-specific):
ELEVPHFHHELVYEGYER (MAXQUANT)

Alignment:
Uniprot       MDVENEQILNVNPADPDNLSDSLFSGDEENAGTEEIKNEINGNWISASSINEARINAKAKRRLRKNSSRDSGRGDSVSDSGSDALRSGLTVPTSPKGRLL
Splooce       MDVENEQILNVNPADPDNLSDSLFSGDEENAGTEEIKNEINGNWISASSINEARINAKAKRRLRKNSSRDSGRGDSVSDSGSDALRSGLTVPTSPKGRLL

Uniprot       DRRSRSGKGRGLPKKGGAGGKGVWGTPGQVYDVEEVDVKDPNYDDDQENCVYETVVLPLDERAFEKTLTPIIQEYFEHGDTNEVAEMLRDLNLGEMKSGV
Splooce       DRRSRSGKGRGLPKKGGAGGKGVWGTPGQVYDVEEVDVKDPNYDDDQENCVYETVVLPLDERAFEKTLTPIIQEYFEHGDTNEVAEMLRDLNLGEMKSGV

Uniprot       PVLAVSLALEGKASHREMTSKLLSDLCGTVMSTTDVEKSFDKLLKDLPELALDTPRAPQLVGQFIARAVGDGILCNTYIDSYKGTVDCVQARAALDKATV
Splooce       PVLAVSLALEGKASHREMTSKLLSDLCGTVMSTTDVEKSFDKLLKDLPELALDTPRAPQLVGQFIARAVGDGILCNTYIDSYKGTVDCVQARAALDKATV

Uniprot       LLSMSKGGKRKDSVWGSGGGQQSVNHLVKEIDMLLKEYLLSGDISEAEHCLKELEVPHFHHELVYEAIIMVLESTGESTFKMILDLLKSLWKSSTITVDQ
Splooce       LLSMSKGGKRKDSVWGSGGGQQSVNHLVKEIDMLLKEYLLSGDISEAEHCLKELEVPHFHHELVYE----------------------------------

Uniprot       MKRGYERIYNEIPDINLDVPHSYSVLERFVEECFQAGIISKQLRDLCPSRGRKRFVSEGDGGRLKPESY
Splooce       ---GYERIYNEIPDINLDVPHSYSVLERFVEECFQAGIISKQLRDLCPSRGRKRFVSEGDGGRLKPESY

----------------------------------------------------------------------------------------------------

P0CG48 (Uniprot)	versus
NM_021009#(r:12_U8381091092268) (Splooce)

For more details about the Alternative Splicing Event -> Link to Splooce page

Peptides that support the ASE (Splooce-specific):
STLHLVLR (PEAKS)

Alignment:
Uniprot       MQIFVKTLTGKTITLEVEPSDTIENVKAKIQDKEGIPPDQQRLIFAGKQLEDGRTLSDYNIQKESTLHLVLRLRGGMQIFVKTLTGKTITLEVEPSDTIE
Splooce       ----------------------------------------------------------------------------------------------------

Uniprot       NVKAKIQDKEGIPPDQQRLIFAGKQLEDGRTLSDYNIQKESTLHLVLRLRGGMQIFVKTLTGKTITLEVEPSDTIENVKAKIQDKEGIPSDQQRLIFAGK
Splooce       ----------------------------------------------------------------------------------------------------

Uniprot       QLEDGRTLSDYNIQKESTLHLVLRLRGGMQIFVKTLTGKTITLEVEPSDTIENVKAKIQDKEGIPPDQQRLIFAGKQLEDGRTLSDYNIQKESTLHLVLR
Splooce       ----------------------------------------------------------------------------------------------------

Uniprot       LRGGMQIFVKTLTGKTITLEVEPSDTIENVKAKIQDKEGIPPDQQRLIFAGKQLEDGRTLSDYNIQKESTLHLVLRLRGGMQIFVKTLTGKTITLEVEPS
Splooce       -------------------------------------------------------MGAPCLTTTSRKKSTLHLVLRLRGGMQIFVKTLTGKTITLEVEPS

Uniprot       DTIENVKAKIQDKEGIPPDQQRLIFAGKQLEDGRTLSDYNIQKESTLHLVLRLRGGMQIFVKTLTGKTITLEVEPSDTIENVKAKIQDKEGIPPDQQRLI
Splooce       DTIENVKAKIQDKEGIPPDQQRLIFAGKQLEDGRTLSDYNIQKESTLHLVLRLRGGMQIFVKTLTGKTITLEVEPSDTIENVKAKIQDKEGIPPDQQRLI

Uniprot       FAGKQLEDGRTLSDYNIQKESTLHLVLRLRGGMQIFVKTLTGKTITLEVEPSDTIENVKAKIQDKEGIPPDQQRLIFAGKQLEDGRTLSDYNIQKESTLH
Splooce       FAGKQLEDGRTLSDYNIQKESTLHLVLRLRGGMQIFVKTLTGKTITLEVEPSDTIENVKAKIQDKEGIPPDQQRLIFAGKQLEDGRTLSDYNIQKESTLH

Uniprot       LVLRLRGGMQIFVKTLTGKTITLEVEPSDTIENVKAKIQDKEGIPPDQQRLIFAGKQLEDGRTLSDYNIQKESTLHLVLRLRGGV
Splooce       LVLRLRGGMQIFVKTLTGKTITLEVEPSDTIENVKAKIQDKEGIPPDQQRLIFAGKQLEDGRTLSDYNIQKESTLHLVLRLRGGV

----------------------------------------------------------------------------------------------------

Q7L5D6 (Uniprot)	versus
NM_015949#(r:7_C742198453071) (Splooce)

For more details about the Alternative Splicing Event -> Link to Splooce page

Peptides that support the ASE (Splooce-specific):
RDPMYNEVR (MAXQUANT)

Alignment:
Uniprot       MAAAAAMAEQESARNGGRNRGGVQRVEGKLRASVEKGDYYEAHQMYRTLFFRYMSQSKHTEARELMYSGALLFFSHGQQNSAADLSMLVLESLEKAEVEV
Splooce       MAAAAAMAEQESARNGGRNRGGVQRVEGKLRASVEKGDYYEAHQMYRTLFFRYMSQSKHTEARELMYSGALLFFSHGQQNSAADLSMLVLESLEKAEVEV

Uniprot       ADELLENLAKVFSLMDPNSPERVTFVSRALKWSSGGSGKLGHPRLHQLLALTLWKEQNYCESRYHFLHSADGEGCANMLVEYSTSRGFRSEVDMFVAQAV
Splooce       ADELLENLAKVFSLMDPNSPERVTFVSRALKWSSGGSGKLGHPRLHQLLALTLWKEQNYCESRYHFLHSADGEGCANMLVEYSTSRGFRSEVDMFVAQAV

Uniprot       LQFLCLKNKSSASVVFTTYTQKHPSIEDGPPFVEPLLNFIWFLLLAVDGGKLTVFTVLCEQYQPSLRRDPMYNEYLDRIGQLFFGVPPKQTSSYGGLLGN
Splooce       LQFLCLKNKSSASVVFTTYTQKHPSIEDGPPFVEPLLNFIWFLLLAVDGGKLTVFTVLCEQYQPSLRRDPMYNEVR--------------AWGWGGRRG-

Uniprot       LLTSLMGSSEQEDGEESPSDGSPIELD
Splooce       --PHGLGPPPLSPLPVPCSTSTA----

----------------------------------------------------------------------------------------------------

Q8WVP7 (Uniprot)	versus
NM_022458#(-t:7_L2914245370753) (Splooce)

For more details about the Alternative Splicing Event -> Link to Splooce page

Peptides that support the ASE (Splooce-specific):
RLNASSVEYNIMELEQELENVK (MAXQUANT)

Alignment:
Uniprot       MEGQDEVSAREQHFHSQVRESTICFLLFAILYVVSYFIITRYKRKSDEQEDEDAIVNRISLFLSTFTLAVSAGAVLLLPFSIISNEILLSFPQNYYIQWL
Splooce       MEGQDEVSAREQHFHSQVRESTICFLLFAILYVVSYFIITRYKRKSDEQEDEDAIVNRISLFLSTFTLAVSAGAVLLLPFSIISNEILLSFPQNYYIQWL

Uniprot       NGSLIHGLWNLASLFSNLCLFVLMPFAFFFLESEGFAGLKKGIRARILETLVMLLLLALLILGIVWVASALIDNDAASMESLYDLWEFYLPYLYSCISLM
Splooce       NGSLIHGLWNLASLFSNLCLFVLMPFAFFFLESEGFAGLKKGIRARILETLVMLLLLALLILGIVWVASALIDNDAASMESLYDLWEFYLPYLYSCISLM

Uniprot       GCLLLLLCTPVGLSRMFTVMGQLLVKPTILEDLDEQIYIITLEEEALQRRLNGLSSSVEYNIMELEQELENVKTLKTKLERRKKASAWERNLVYPAVMVL
Splooce       GCLLLLLCTPVGLSRMFTVMGQLLVKPTILEDLDEQIYIITLEEEALQRRLN--ASSVEYNIMELEQELENVKTLKTKLERRKKASAWERNLVYPAVMVL

Uniprot       LLIETSISVLLVACNILCLLVDETAMPKGTRGPGIGNASLSTFGFVGAALEIILIFYLMVSSVVGFYSLRFFGNFTPKKDDTTMTKIIGNCVSILVLSSA
Splooce       LLIETSISVLLVACNILCLLVDETAMPKGTRGPGIGNASLSTFGFVGAALEIILIFYLMVSSVVGFYSLRFFGNFTPKKDDTTMTKIIGNCVSILVLSSA

Uniprot       LPVMSRTLGITRFDLLGDFGRFNWLGNFYIVLSYNLLFAIVTTLCLVRKFTSAVREELFKALGLHKLHLPNTSRDSETAKPSVNGHQKAL
Splooce       LPVMSRTLGITRFDLLGDFGRFNWLGNFYIVLSYNLLFAIVTTLCLVRKFTSAVREELFKALGLHKLHLPNTSRDSETAKPSVNGHQKAL

----------------------------------------------------------------------------------------------------

P49189 (Uniprot)	versus
NM_000696#(f-s-:1_A7804977911027) (Splooce)

For more details about the Alternative Splicing Event -> Link to Splooce page

Peptides that support the ASE (Splooce-specific):
VCEHIQLPGGSFGYTR (MAXQUANT)

Alignment:
Uniprot       MFLRAGLAALSPLLRSLRPSPVAAMSTGTFVVSQPLNYRGGARVEPADASGTEKAFEPATGRVIATFTCSGEKEVNLAVQNAKAAFKIWSQKSGMERCRI
Splooce       MFLRAGLAALSPLLRSLRPSPVAAMSTGTFVVSQPLNYRGGARVEPADASGTEKAFEPATGRVIATFTCSGEKEVNLAVQNAKAAFKIWSQKSGMERCRI

Uniprot       LLEAARIIREREDEIATMECINNGKSIFEARLDIDISWQCLEYYAGLAASMAGEHIQLPGGSFGYTRREPLGVCVGIGAWNYPFQIASWKSAPALACGNA
Splooce       LLEAARIIR------------------------------------------VCEHIQLPGGSFGYTRREPLGVCVGIGAWNYPFQIASWKSAPALACGNA

Uniprot       MVFKPSPFTPVSALLLAEIYSEAGVPPGLFNVVQGGAATGQFLCQHPDVAKVSFTGSVPTGMKIMEMSAKGIKPVTLELGGKSPLIIFSDCDMNNAVKGA
Splooce       MVFKPSPFTPVSALLLAEIYSEAGVPPGLFNVVQGGAATGQFLCQHPDVAKVSFTGSVPTGMKIMEMSAKGIKPVTLELGGKSPLIIFSDCDMNNAVKGA

Uniprot       LMANFLTQGQVCCNGTRVFVQKEILDKFTEEVVKQTQRIKIGDPLLEDTRMGPLINRPHLERVLGFVKVAKEQGAKVLCGGDIYVPEDPKLKDGYYMRPC
Splooce       LMANFLTQGQVCCNGTRVFVQKEILDKFTEEVVKQTQRIKIGDPLLEDTRMGPLINRPHLERVLGFVKVAKEQGAKVLCGGDIYVPEDPKLKDGYYMRPC

Uniprot       VLTNCRDDMTCVKEEIFGPVMSILSFDTEAEVLERANDTTFGLAAGVFTRDIQRAHRVVAELQAGTCFINNYNVSPVELPFGGYKKSGFGRENGRVTIEY
Splooce       VLTNCRDDMTCVKEEIFGPVMSILSFDTEAEVLERANDTTFGLAAGVFTRDIQRAHRVVAELQAGTCFINNYNVSPVELPFGGYKKSGFGRENGRVTIEY

Uniprot       YSQLKTVCVEMGDVESAF
Splooce       YSQLKTVCVEMGDVESAF

----------------------------------------------------------------------------------------------------

P98173 (Uniprot)	versus
NM_021806#(-s-s-s-:X_F8247422908188) (Splooce)

For more details about the Alternative Splicing Event -> Link to Splooce page

Peptides that support the ASE (Splooce-specific):
IVVLVVSVGVTWIVVSILLGGPGSGFPRIQQLFTR (MAXQUANT)

Alignment:
Uniprot       MRLAGPLRIVVLVVSVGVTWIVVSILLGGPGSGFPRIQQLFTSPESSVTAAPRARKYKCGLPQPCPEEHLAFRVVSGAANVIGPKICLEDKMLMSSVKDN
Splooce       MRLAGPLRIVVLVVSVGVTWIVVSILLGGPGSGFPRIQQLFTR---------------------------------------------------------

Uniprot       VGRGLNIALVNGVSGELIEARAFDMWAGDVNDLLKFIRPLHEGTLVFVASYDDPATKMNEETRKLFSELGSRNAKELAFRDSWVFVGAKGVQNKSPFEQH
Splooce       ------------VSGELIEARAFDMWAGDVNDLLKFIRPLHEGTLVFVASYDDPATKMNEETRKLFSELGSRNAKELAFRDSWVFVGAKGVQNKSPFEQH

Uniprot       VKNSKHSNKYEGWPEALEMEGCIPRRSTAS
Splooce       VKNSKHSNKYEGWPEALEMEGCIPRRSTAS

----------------------------------------------------------------------------------------------------

O15382 (Uniprot)	versus
NM_001190#(-s-s-s-s-:19_B9996699405531) (Splooce)

For more details about the Alternative Splicing Event -> Link to Splooce page

Peptides that support the ASE (Splooce-specific):
LGGTESEPTSGCSR (MAXQUANT)

Alignment:
Uniprot       MAAAALGQIWARKLLSVPWLLCGPRRYASSSFKAADLQLEMTQKPHKKPGPGEPLVFGKTFTDHMLMVEWNDKGWGQPRIQPFQNLTLHPASSSLHYSLQ
Splooce       MAAAALGQIWARKLLSVPWLLCGPRRYASSSFKAADLQLEMTQKPHKKPGPGEPLVFGKTFTDHMLMVEWNDKGWGQPRIQPFQNLTLHPASSSLHYSLQ

Uniprot       LFEGMKAFKGKDQQVRLFRPWLNMDRMLRSAMRLCLPSFDKLELLECIRRLIEVDKDWVPDAAGTSLYVRPVLIGNEPSLGVSQPTRALLFVILCPVGAY
Splooce       LFEGMKAFKGKDQQVRLFRPWLNMDRMLRSAMRLCLPSFDKLELLECIRRLIEVDKDWVPDAAGTSLYVRPVLIGNEPSLGVSQPTRALLFVILCPVGAY

Uniprot       FPGGSVTPVSLLADPAFIRAWVGGVGNYKLGGNYGPTVLVQQEALKRGCEQVLWLYGPDHQLTEVGTMNIFVYWTHEDGVLELVTPPLNGVILPGVVRQS
Splooce       FPGGSVTPVSLLADPAFIRAWVGGVGNYKLGG--------TESEPTSGCS--------------------------------------------------

Uniprot       LLDMAQTWGEFRVVERTITMKQLLRALEEGRVREVFGSGTACQVCPVHRILYKDRNLHIPTMENGPELILRFQKELKEIQYGIRAHEWMFPV
Splooce       ------------------------RCEAAG-----------CAPDPPTRSISSRQ--HSPPYQ-----------------------------

----------------------------------------------------------------------------------------------------

P50851 (Uniprot)	versus
NM_006726#(f-:4_L4835817596158) (Splooce)

For more details about the Alternative Splicing Event -> Link to Splooce page

Peptides that support the ASE (Splooce-specific):
IILAYTEGLHGK (MAXQUANT)

Alignment:
Uniprot       MASEDNRVPSPPPTGDDGGGGGREETPTEGGALSLKPGLPIRGIRMKFAVLTGLVEVGEVSNRDIVETVFNLLVGGQFDLEMNFIIQEGESINCMVDLLE
Splooce       MASEDNRVPSPPPTGDDGGGGGREETPTEGGALSLKPGLPIRGIRMKFAVLTGLVEVGEVSNRDIVETVFNLLVGGQFDLEMNFIIQEGESINCMVDLLE

Uniprot       KCDITCQAEVWSMFTAILKKSIRNLQVCTEVGLVEKVLGKIEKVDNMIADLLVDMLGVLASYNLTVRELKLFFSKLQGDKGRWPPHAGKLLSVLKHMPQK
Splooce       KCDITCQAEVWSMFTAILKKSIRNLQVCTEVGLVEKVLGKIEKVDNMIADLLVDMLGVLASYNLTVRELKLFFSKLQGDKGRWPPHAGKLLSVLKHMPQK

Uniprot       YGPDAFFNFPGKSAAAIALPPIAKWPYQNGFTFHTWLRMDPVNNINVDKDKPYLYCFRTSKGLGYSAHFVGGCLIVTSIKSKGKGFQHCVKFDFKPQKWY
Splooce       YGPDAFFNFPGKSAAAIALPPIAKWPYQNGFTFHTWLRMDPVNNINVDKDKPYLYCFRTSKGLGYSAHFVGGCLIVTSIKSKGKGFQHCVKFDFKPQKWY

Uniprot       MVTIVHIYNRWKNSELRCYVNGELASYGEITWFVNTSDTFDKCFLGSSETADANRVFCGQMTAVYLFSEALNAAQIFAIYQLGLGYKGTFKFKAESDLFL
Splooce       MVTIVHIYNRWKNSELRCYVNGELASYGEITWFVNTSDTFDKCFLGSSETADANRVFCGQMTAVYLFSEALNAAQIFAIYQLGLGYKGTFKFKAESDLFL

Uniprot       AEHHKLLLYDGKLSSAIAFTYNPRATDAQLCLESSPKDNPSIFVHSPHALMLQDVKAVLTHSIQSAMHSIGGVQVLFPLFAQLDYRQYLSDEIDLTICST
Splooce       AEHHKLLLYDGKLSSAIAFTYNPRATDAQLCLESSPKDNPSIFVHSPHALMLQDVKAVLTHSIQSAMHSIGGVQVLFPLFAQLDYRQYLSDEIDLTICST

Uniprot       LLAFIMELLKNSIAMQEQMLACKGFLVIGYSLEKSSKSHVSRAVLELCLAFSKYLSNLQNGMPLLKQLCDHVLLNPAIWIHTPAKVQLMLYTYLSTEFIG
Splooce       LLAFIMELLKNSIAMQEQMLACKGFLVIGYSLEKSSKSHVSRAVLELCLAFSKYLSNLQNGMPLLKQLCDHVLLNPAIWIHTPAKVQLMLYTYLSTEFIG

Uniprot       TVNIYNTIRRVGTVLLIMHTLKYYYWAVNPQDRSGITPKGLDGPRPNQKEMLSLRAFLLMFIKQLVMKDSGVKEDELQAILNYLLTMHEDDNLMDVLQLL
Splooce       TVNIYNTIRRVGTVLLIMHTLKYYYWAVNPQDRSGITPKGLDGPRPNQKEMLSLRAFLLMFIKQLVMKDSGVKEDELQAILNYLLTMHEDDNLMDVLQLL

Uniprot       VALMSEHPNSMIPAFDQRNGLRVIYKLLASKSEGIRVQALKAMGYFLKHLAPKRKAEVMLGHGLFSLLAERLMLQTNLITMTTYNVLFEILIEQIGTQVI
Splooce       VALMSEHPNSMIPAFDQRNGLRVIYKLLASKSEGIRVQALKAMGYFLKHLAPKRKAEVMLGHGLFSLLAERLMLQTNLITMTTYNVLFEILIEQIGTQVI

Uniprot       HKQHPDPDSSVKIQNPQILKVIATLLRNSPQCPESMEVRRAFLSDMIKLFNNSRENRRSLLQCSVWQEWMLSLCYFNPKNSDEQKITEMVYAIFRILLYH
Splooce       HKQHPDPDSSVKIQNPQILKVIATLLRNSPQCPESMEVRRAFLSDMIKLFNNSRENRRSLLQCSVWQEWMLSLCYFNPKNSDEQKITEMVYAIFRILLYH

Uniprot       AVKYEWGGWRVWVDTLSITHSKVTFEIHKENLANIFREQQGKVDEEIGLCSSTSVQAASGIRRDINVSVGSQQPDTKDSPVCPHFTTNGNENSSIEKTSS
Splooce       AVKYEWGGWRVWVDTLSITHSKVTFEIHKENLANIFREQQGKVDEEIGLCSSTSVQAASGIRRDINVSVGSQQPDTKDSPVCPHFTTNGNENSSIEKTSS

Uniprot       LESASNIELQTTNTSYEEMKAEQENQELPDEGTLEETLTNETRNADDLEVSSDIIEAVAISSNSFITTGKDSMTVSEVTASISSPSEEDASEMPEFLDKS
Splooce       LESASNIELQTTNTSYEEMKAEQENQELPDEGTLEETLTNETRNADDLEVSSDIIEAVAISSNSFITTGKDSMTVSEVTASISSPSEEDASEMPEFLDKS

Uniprot       IVEEEEDDDYVELKVEGSPTEEANLPTELQDNSLSPAASEAGEKLDMFGNDDKLIFQEGKPVTEKQTDTETQDSKDSGIQTMTASGSSAMSPETTVSQIA
Splooce       IVEEEEDDDYVELKVEGSPTEEANLPTELQDNSLSPAASEAGEKLDMFGNDDKLIFQEGKPVTEKQTDTETQDSKDSGIQTMTASGSSAMSPETTVSQIA

Uniprot       VESDLGQMLEEGKKATNLTRETKLINDCHGSVSEASSEQKIAKLDVSNVATDTERLELKASPNVEAPQPHRHVLEISRQHEQPGQGIAPDAVNGQRRDSR
Splooce       VESDLGQMLEEGKKATNLTRETKLINDCHGSVSEASSEQKIAKLDVSNVATDTERLELKASPNVEAPQPHRHVLEISRQHEQPGQGIAPDAVNGQRRDSR

Uniprot       STVFRIPEFNWSQMHQRLLTDLLFSIETDIQMWRSHSTKTVMDFVNSSDNVIFVHNTIHLISQVMDNMVMACGGILPLLSAATSATHELENIEPTQGLSI
Splooce       STVFRIPEFNWSQMHQRLLTDLLFSIETDIQMWRSHSTKTVMDFVNSSDNVIFVHNTIHLISQVMDNMVMACGGILPLLSAATSATHELENIEPTQGLSI

Uniprot       EASVTFLQRLISLVDVLIFASSLGFTEIEAEKSMSSGGILRQCLRLVCAVAVRNCLECQQHSQLKTRGDKALKPMHSLIPLGKSAAKSPVDIVTGGISPV
Splooce       EASVTFLQRLISLVDVLIFASSLGFTEIEAEKSMSSGGILRQCLRLVCAVAVRNCLECQQHSQLKTRGDKALKPMHSLIPLGKSAAKSPVDIVTGGISPV

Uniprot       RDLDRLLQDMDINRLRAVVFRDIEDSKQAQFLALAVVYFISVLMVSKYRDILEPQNERHSQSCTETGSENENVSLSEITPAAFSTLTTASVEESESTSSA
Splooce       RDLDRLLQDMDINRLRAVVFRDIEDSKQAQFLALAVVYFISVLMVSKYRDILEPQNERHSQSCTETGSENENVSLSEITPAAFSTLTTASVEESESTSSA

Uniprot       RRRDSGIGEETATGLGSHVEVTPHTAPPGVSAGPDAISEVLSTLSLEVNKSPETKNDRGNDLDTKATPSVSVSKNVNVKDILRSLVNIPADGVTVDPALL
Splooce       RRRDSGIGEETATGLGSHVEVTPHTAPPGVSAGPDAISEVLSTLSLEVNKSPETKNDRGNDLDTKATPSVSVSKNVNVKDILRSLVNIPADGVTVDPALL

Uniprot       PPACLGALGDLSVEQPVQFRSFDRSVIVAAKKSAVSPSTFNTSIPTNAVSVVSSVDSAQASDMGGESPGSRSSNAKLPSVPTVDSVSQDPVSNMSITERL
Splooce       PPACLGALGDLSVEQPVQFRSFDRSVIVAAKKSAVSPSTFNTSIPTNAVSVVSSVDSAQASDMGGESPGSRSSNAKLPSVPTVDSVSQDPVSNMSITERL

Uniprot       EHALEKAAPLLREIFVDFAPFLSRTLLGSHGQELLIEGTSLVCMKSSSSVVELVMLLCSQEWQNSIQKNAGLAFIELVNEGRLLSQTMKDHLVRVANEAE
Splooce       EHALEKAAPLLREIFVDFAPFLSRTLLGSHGQELLIEGTSLVCMKSSSSVVELVMLLCSQEWQNSIQKNAGLAFIELVNEGRLLSQTMKDHLVRVANEAE

Uniprot       FILSRQRAEDIHRHAEFESLCAQYSADKREDEKMCDHLIRAAKYRDHVTATQLIQKIINILTDKHGAWGNSAVSRPLEFWRLDYWEDDLRRRRRFVRNPL
Splooce       FILSRQRAEDIHRHAEFESLCAQYSADKREDEKMCDHLIRAAKYRDHVTATQLIQKIINILTDKHGAWGNSAVSRPLEFWRLDYWEDDLRRRRRFVRNPL

Uniprot       GSTHPEATLKTAVEHVCIFKLRENSKATDEDILAKGKQSIRSQALGNQNSENEILLEGDDDTLSSVDEKDLENLAGPVSLSTPAQLVAPSVVVKGTLSVT
Splooce       GSTHPEATLKTAVEHVCIFKLRENSKATDEDILAKGKQSIRSQALGNQNSENEILLEGDDDTLSSVDEKDLENLAGPVSLSTPAQLVAPSVVVKGTLSVT

Uniprot       SSELYFEVDEEDPNFKKIDPKILAYTEGLHGKWLFTEIRSIFSRRYLLQNTALEIFMANRVAVMFNFPDPATVKKVVNYLPRVGVGTSFGLPQTRRISLA
Splooce       SSELYFEVDEEDPNFKKI---ILAYTEGLHGKWLFTEIRSIFSRRYLLQNTALEIFMANRVAVMFNFPDPATVKKVVNYLPRVGVGTSFGLPQTRRISLA

Uniprot       SPRQLFKASNMTQRWQHREISNFEYLMFLNTIAGRSYNDLNQYPVFPWVITNYESEELDLTLPTNFRDLSKPIGALNPKRAAFFAERYESWEDDQVPKFH
Splooce       SPRQLFKASNMTQRWQHREISNFEYLMFLNTIAGRSYNDLNQYPVFPWVITNYESEELDLTLPTNFRDLSKPIGALNPKRAAFFAERYESWEDDQVPKFH

Uniprot       YGTHYSTASFVLAWLLRIEPFTTYFLNLQGGKFDHADRTFSSISRAWRNSQRDTSDIKELIPEFYYLPEMFVNFNNYNLGVMDDGTVVSDVELPPWAKTS
Splooce       YGTHYSTASFVLAWLLRIEPFTTYFLNLQGGKFDHADRTFSSISRAWRNSQRDTSDIKELIPEFYYLPEMFVNFNNYNLGVMDDGTVVSDVELPPWAKTS

Uniprot       EEFVHINRLALESEFVSCQLHQWIDLIFGYKQQGPEAVRALNVFYYLTYEGAVNLNSITDPVLREAVEAQIRSFGQTPSQLLIEPHPPRGSAMQVSPLMF
Splooce       EEFVHINRLALESEFVSCQLHQWIDLIFGYKQQGPEAVRALNVFYYLTYEGAVNLNSITDPVLREAVEAQIRSFGQTPSQLLIEPHPPRGSAMQVSPLMF

Uniprot       TDKAQQDVIMVLKFPSNSPVTHVAANTQPGLATPAVITVTANRLFAVNKWHNLPAHQGAVQDQPYQLPVEIDPLIASNTGMHRRQITDLLDQSIQVHSQC
Splooce       TDKAQQDVIMVLKFPSNSPVTHVAANTQPGLATPAVITVTANRLFAVNKWHNLPAHQGAVQDQPYQLPVEIDPLIASNTGMHRRQITDLLDQSIQVHSQC

Uniprot       FVITSDNRYILVCGFWDKSFRVYSTDTGRLIQVVFGHWDVVTCLARSESYIGGNCYILSGSRDATLLLWYWNGKCSGIGDNPGSETAAPRAILTGHDYEV
Splooce       FVITSDNRYILVCGFWDKSFRVYSTDTGRLIQVVFGHWDVVTCLARSESYIGGNCYILSGSRDATLLLWYWNGKCSGIGDNPGSETAAPRAILTGHDYEV

Uniprot       TCAAVCAELGLVLSGSQEGPCLIHSMNGDLLRTLEGPENCLKPKLIQASREGHCVIFYENGLFCTFSVNGKLQATMETDDNIRAIQLSRDGQYLLTGGDR
Splooce       TCAAVCAELGLVLSGSQEGPCLIHSMNGDLLRTLEGPENCLKPKLIQASREGHCVIFYENGLFCTFSVNGKLQATMETDDNIRAIQLSRDGQYLLTGGDR

Uniprot       GVVVVRQVSDLKQLFAYPGCDAGIRAMALSYDQRCIISGMASGSIVLFYNDFNRWHHEYQTRY
Splooce       GVVVVRQVSDLKQLFAYPGCDAGIRAMALSYDQRCIISGMASGSIVLFYNDFNRWHHEYQTRY

----------------------------------------------------------------------------------------------------

P27449 (Uniprot)	versus
NM_001694#(-s-:16_A9899533815955) (Splooce)

For more details about the Alternative Splicing Event -> Link to Splooce page

Peptides that support the ASE (Splooce-specific):
PAAPTLR (PEAKS)

Alignment:
Uniprot       MSESKSGPEYASFFAVMGASAAMVFSALGAAYGTAKSGTGIAAMSVMRPEQIMKSIIPVVMAGIIAIYGLVVAVLIANSLNDDISLYKSFLQLGAGLSVG
Splooce       --MLRFSPSWAPRPPWS--SAELPPAGRRPERGPERPGSRLCHR--HRGGRWRAGHRPAAPT====IRG------------HDP---DSHLRRGARP---

Uniprot       LSGLAAGFAIGIVGDAGVRGTAQQPRLFVGMILILIFAEVLGLYGLIVALILSTK
Splooce       LRSHRRPHPLHKVDP--LR--AHQPQNIM--------------------------

----------------------------------------------------------------------------------------------------

Q8N201 (Uniprot)	versus
NM_001080453#(-s-s-s-s-s-s-s-s-:7_I8828218907790) (Splooce)

For more details about the Alternative Splicing Event -> Link to Splooce page

Peptides that support the ASE (Splooce-specific):
MLMEMVMTNRR (MAXQUANT)

Alignment:
Uniprot       MNRAKPTTVRRPSAAAKPSGHPPPGDFIALGSKGQANESKTASTLLKPAPSGLPSERKRDAAAALSSASALTGLTKRPKLSSTPPLSALGRLAEAAVAEK
Splooce       MNRAKPTTVRRPSAAAKPSGHPPPGDFIALGSKGQANESKTASTLLKPAPSGLPSERKRDAAAALSSASALTGLTKRPKLSSTPPLSALGRLAEAAVAEK

Uniprot       RAISPSIKEPSVVPIEVLPTVLLDEIEAAELEGNDDRIEGVLCGAVKQLKVTRAKPDSTLYLSLMYLAKIKPNIFATEGVIEALCSLLRRDASINFKAKG
Splooce       RAISPSIKEPSVVPIEVLPTVLLDEIEAAELEGNDDRIEGVLCGAVKQLKVTRAKPDSTLYLSLMYLAKIKPNIFATEGVIEALCSLLRRDASINFKAKG

Uniprot       NSLVSVLACNLLMAAYEEDENWPEIFVKVYIEDSLGERIWVDSPHCKTFVDNIQTAFNTRMPPRSVLLQGEAGRVAGDLGAGSSPHPSLTEEEDSQTELL
Splooce       NSLVSVLACNLLMAAYEEDENWPEIFVKVYIEDSLGERIWVDSPHCKTFVDNIQTAFNTRMPPRSVLLQGEAGRVAGDLGAGSSPHPSLTEEEDSQTELL

Uniprot       IAEEKLSPEQEGQLMPRYEELAESVEEYVLDMLRDQLNRRQPIDNVSRNLLRLLTSTCGYKEVRLLAVQKLEMWLQNPKLTRPAQDLLMSVCMNCNTHGS
Splooce       IAEEKLSPEQEGQLMPRYEELAESVEEYVLDMLRDQLNRRQPIDNVSRNLLRLLTSTCGYKEVRLLAVQKLEMWLQNPKLTRPAQDLLMSVCMNCNTHGS

Uniprot       EDMDVISHLIKIRLKPKVLLNHFMLCIRELLSAHKDNLGTTIKLVIFNELSSARNPNNMQVLYTALQHSSELAPKFLAMVFQDLLTNKDDYLRASRALLR
Splooce       EDMDVISHLIKIRLKPKVLLNHFMLCIRELLSAHKDNLGTTIKLVIFNELSSARNPNNMQVLYTALQHSSELAPKFLAMVFQDLLTNKDDYLRASRALLR

Uniprot       EIIKQTKHEINFQAFCLGLMQERKEPQYLEMEFKERFVVHITDVLAVSMMLGITAQVKEAGIAWDKGEKRNLEVLRSFQNQIAAIQRDAVWWLHTVVPSI
Splooce       EIIKQTKHEINFQAFCLGLMQERKEPQYLEMEFKERFVVHITDVLAVSMMLGITAQVKEAGIAWDKGEKRNLEVLRSFQNQIAAIQRDAVWWLHTVVPSI

Uniprot       SKLAPKDYVHCLHKVLFTEQPETYYKWDNWPPESDRNFFLRLCSEVPILEDTLMRILVIGLSRELPLGPADAMELADHLVKRAAAVQADDVEVLKVGRTQ
Splooce       SKLAPKDYVHCLHKVLFTEQPETYYKWDNWPPESDRNFFLRLCSEVPILEDTLMRILVIGLSRELPLGPADAMELADHLVKRAAAVQADDVEVLKVGRTQ

Uniprot       LIDAVLNLCTYHHPENIQLPPGYQPPNLAISTLYWKAWPLLLVVAAFNPENIGLAAWEEYPTLKMLMEMVMTNNYSYPPCTLTDEETRTEMLNRELQTAQ
Splooce       LIDAVLNLCTYHHPENIQLPPGYQPPNLAISTLYWKAWPLLLVVAAFNPENIGLAAWEEYPTLKMLMEMVMTN---------------------------

Uniprot       REKQEILAFEGHLAAASTKQTITESSSLLLSQLTSLDPQGPPRRPPPHILDQVKSLNQSLRLGHLLCRSRNPDFLLHIIQRQASSQSMPWLADLVQSSEG
Splooce       -RRQRVPGAAGHLVSG--------------GEATAHRLPGG-----------------------------------HIGGGAAAS---------------

Uniprot       SLDVLPVQCLCEFLLHDAVDDAASGEEDDEGESKEQKAKKRQRQQKQRQLLGRLQDLLLGPKADEQTTCEVLDYFLRRLGSSQVASRVLAMKGLSLVLSE
Splooce       ----------------------------------------------------------------------------------------------------

Uniprot       GSLRDGEEKEPPMEEDVGDTDVLQGYQWLLRDLPRLPLFDSVRSTTALALQQAIHMETDPQTISAYLIYLSQHTPVEEQAQHSDLALDVARLVVERSTIM
Splooce       ----------------------------------------------------------------------------------------------------

Uniprot       SHLFSKLSPSAASDAVLSALLSIFSRYVRRMRQSKEGEEVYSWSESQDQVFLRWSSGETATMHILVVHAMVILLTLGPPRADDSEFQALLDIWFPEEKPL
Splooce       ----------------------------------------------------------------------------------------------------

Uniprot       PTAFLVDTSEEALLLPDWLKLRMIRSEVLRLVDAALQDLEPQQLLLFVQSFGIPVSSMSKLLQFLDQAVAHDPQTLEQNIMDKNYMAHLVEVQHERGASG
Splooce       ----------------------------------------------------------------------------------------------------

Uniprot       GQTFHSLLTASLPPRRDSTEAPKPKSSPEQPIGQGRIRVGTQLRVLGPEDDLAGMFLQIFPLSPDPRWQSSSPRPVALALQQALGQELARVVQGSPEVPG
Splooce       ----------------------------------------------------------------------------------------------------

Uniprot       ITVRVLQALATLLSSPHGGALVMSMHRSHFLACPLLRQLCQYQRCVPQDTGFSSLFLKVLLQMLQWLDSPGVEGGPLRAQLRMLASQASAGRRLSDVRGG
Splooce       ----------------------------------------------------------------------------------------------------

Uniprot       LLRLAEALAFRQDLEVVSSTVRAVIATLRSGEQCSVEPDLISKVLQGLIEVRSPHLEELLTAFFSATADAASPFPACKPVVVVSSLLLQEEEPLAGGKPG
Splooce       ----------------------------------------------------------------------------------------------------

Uniprot       ADGGSLEAVRLGPSSGLLVDWLEMLDPEVVSSCPDLQLRLLFSRRKGKGQAQVPSFRPYLLTLFTHQSSWPTLHQCIRVLLGKSREQRFDPSASLDFLWA
Splooce       ----------------------------------------------------------------------------------------------------

Uniprot       CIHVPRIWQGRDQRTPQAAAGGAGAAGPGPGAHQPGGADPGRGGDAEPGRGHSRLQPHPGPAAPAAQLLLWGR
Splooce       -------------------------------------------------------------------------

----------------------------------------------------------------------------------------------------

Q9H4G0 (Uniprot)	versus
NM_012156#(-s-s-:20_E5683279496695) (Splooce)

For more details about the Alternative Splicing Event -> Link to Splooce page

Peptides that support the ASE (Splooce-specific):
SLDGVLR (MAXQUANT)

Alignment:
Uniprot       MTTETGPDSEVKKAQEEAPQQPEAAAAVTTPVTPAGHGHPEANSNEKHPSQQDTRPAEQSLDMEEKDYSEADGLSERTTPSKAQKSPQKIAKKYKSAICR
Splooce       MTTETGPDSEVKKAQEEAPQQPEAAAAVTTPVTPAGHGHPEANSNEKHPSQQDTRPAEQSLDMEEKDYSEADGLSERTTPSKAQKSPQKIAKKYKSAICR

Uniprot       VTLLDASEYECEVEKHGRGQVLFDLVCEHLNLLEKDYFGLTFCDADSQKNWLDPSKEIKKQIRSSPWNFAFTVKFYPPDPAQLTEDITRYYLCLQLRADI
Splooce       VTLLDASEYECEVEKHGRGQVLFDLVCEHLNLLEKDYFGLTFCDADSQKNWLDPSKEIKKQIRSSPWNFAFTVKFYPPDPAQLTEDITRYYLCLQLRADI

Uniprot       ITGRLPCSFVTHALLGSYAVQAELGDYDAEEHVGNYVSELRFAPNQTRELEERIMELHKTYRGMTPGEAEIHFLENAKKLSMYGVDLHHAKDSEGIDIML
Splooce       ITGRLPCSFVTHALLGSYAVQAELGDYDAEEHVGNYVSELRFAPNQTRELEERIMELHKTYRGMTPGEAEIHFLENAKKLSMYGVDLHHAKDSEGIDIML

Uniprot       GVCANGLLIYRDRLRINRFAWPKILKISYKRSNFYIKIRPGEYEQFESTIGFKLPNHRSAKRLWKVCIEHHTFFRLVSPEPPPKGFLVMGSKFRYSGRTQ
Splooce       GVCANGLLIYRDRLRINRFAWPKILKISYKRSNFYIKIRPGEYEQFESTIGFKLPNHRSAKRLWKVCIEHHTFFRLVSPEPPPKGFLVMGSKFRYSGRTQ

Uniprot       AQTRQASALIDRPAPFFERSSSKRYTMSRSLDGAEFSRPASVSENHDAGPDGDKRDEDGESGGQRSEAEEGEVRTPTKIKELKPEQETTPRHKQEFLDKP
Splooce       AQTRQASALIDRPAPFFERSSSKRYTMSRSLD--------------------------------------GVLRQARRCLAEAPGQHQ------------

Uniprot       EDVLLKHQASINELKRTLKEPNSKLIHRDRDWERERRLPSSPASPSPKGTPEKANERAGLREGSEEKVKPPRPRAPESDTGDEDQDQERDTVFLKDNHLA
Splooce       ----------------------------------------------------------------------------------------------------

Uniprot       IERKCSSITVSSTSSLEAEVDFTVIGDYHGSAFEDFSRSLPELDRDKSDSDTEGLLFSRDLNKGAPSQDDESGGIEDSPDRGACSTPDMPQFEPVKTETM
Splooce       ----------------------------------------------------------------------------------------------------

Uniprot       TVSSLAIRKKIEPEAVLQTRVSAMDNTQQVDGSASVGREFIATTPSITTETISTTMENSLKSGKGAAAMIPGPQTVATEIRSLSPIIGKDVLTSTYGATA
Splooce       ----------------------------------------------------------------------------------------------------

Uniprot       ETLSTSTTTHVTKTVKGGFSETRIEKRIIITGDEDVDQDQALALAIKEAKLQHPDMLVTKAVVYRETDPSPEERDKKPQES
Splooce       ---------------------------------------------------------------------------------

----------------------------------------------------------------------------------------------------

Q01780 (Uniprot)	versus
NM_001001998#(-s-s-s-:1_E9767247913955) (Splooce)

For more details about the Alternative Splicing Event -> Link to Splooce page

Peptides that support the ASE (Splooce-specific):
PIEETPCHFISSLDELVELNEKLLNCQEFAVDLEK (MAXQUANT)

Alignment:
Uniprot       MAPPSTREPRVLSATSATKSDGEMVLPGFPDADSFVKFALGSVVAVTKASGGLPQFGDEYDFYRSFPGFQAFCETQGDRLLQCMSRVMQYHGCRSNIKDR
Splooce       MAPPSTREPRVLSATSATKSDGEMVLPGFPDADSFVKFALGSVVAVTKASGGLPQFGDEYDFYRSFPGFQAFCETQGDRLLQCMSRVMQYHGCRSNIKDR

Uniprot       SKVTELEDKFDLLVDANDVILERVGILLDEASGVNKNQQPVLPAGLQVPKTVVSSWNRKAAEYGKKAKSETFRLLHAKNIIRPQLKFREKIDNSNTPFLP
Splooce       SKVTELEDKFDLLVDANDVILERVGILLDEASGVNKNQQPVLPAGLQVPKTVVSSWNRKAAEYGKKAKSETFRLLHAKNIIRPQLKFREKIDNSNTPFLP

Uniprot       KIFIKPNAQKPLPQALSKERRERPQDRPEDLDVPPALADFIHQQRTQQVEQDMFAHPYQYELNHFTPADAVLQKPQPQLYRPIEETPCHFISSLDELVEL
Splooce       KIFIKPNAQKPLPQALSKERRERPQDRPEDLDVPPALADFIHQQRTQQVEQDMFAHPYQYELNHFTPADAVLQKPQPQLYRPIEETPCHFISSLDELVEL

Uniprot       NEKLLNCQEFAVDLEHHSYRSFLGLTCLMQISTRTEDFIIDTLELRSDMYILNESLTDPAIVKVFHGADSDIEWLQKDFGLYVVNMFDTHQAARLLNLGR
Splooce       NEKLLNCQEFAVDLE-------------------------------------------------------------------------------------

Uniprot       HSLDHLLKLYCNVDSNKQYQLADWRIRPLPEEMLSYARDDTHYLLYIYDKMRLEMWERGNGQPVQLQVVWQRSRDICLKKFIKPIFTDESYLELYRKQKK
Splooce       -------------------------------------------------------------------------------KFIKPIFTDESYLELYRKQKK

Uniprot       HLNTQQLTAFQLLFAWRDKTARREDESYGYVLPNHMMLKIAEELPKEPQGIIACCNPVPPLVRQQINEMHLLIQQAREMPLLKSEVAAGVKKSGPLPSAE
Splooce       HLNTQQLTAFQLLFAWRDKTARREDESYGYVLPNHMMLKIAEELPKEPQGIIACCNPVPPLVRQQINEMHLLIQQAREMPLLKSEVAAGVKKSGPLPSAE

Uniprot       RLENVLFGPHDCSHAPPDGYPIIPTSGSVPVQKQASLFPDEKEDNLLGTTCLIATAVITLFNEPSAEDSKKGPLTVAQKKAQNIMESFENPFRMFLPSLG
Splooce       RLENVLFGPHDCSHAPPDGYPIIPTSGSVPVQKQASLFPDEKEDNLLGTTCLIATAVITLFNEPSAEDSKKGPLTVAQKKAQNIMESFENPFRMFLPSLG

Uniprot       HRAPVSQAAKFDPSTKIYEISNRWKLAQVQVQKDSKEAVKKKAAEQTAAREQAKEACKAAAEQAISVRQQVVLENAAKKRERATSDPRTTEQKQEKKRLK
Splooce       HRAPVSQAAKFDPSTKIYEISNRWKLAQVQVQKDSKEAVKKKAAEQTAAREQAKEACKAAAEQAISVRQQVVLENAAKKRERATSDPRTTEQKQEKKRLK

Uniprot       ISKKPKDPEPPEKEFTPYDYSQSDFKAFAGNSKSKVSSQFDPNKQTPSGKKCIAAKKIKQSVGNKSMSFPTGKSDRGFRYNWPQR
Splooce       ISKKPKDPEPPEKEFTPYDYSQSDFKAFAGNSKSKVSSQFDPNKQTPSGKKCIAAKKIKQSVGNKSMSFPTGKSDRGFRYNWPQR

----------------------------------------------------------------------------------------------------

P21399 (Uniprot)	versus
NM_002197#(-t:9_A6439648373952) (Splooce)

For more details about the Alternative Splicing Event -> Link to Splooce page

Peptides that support the ASE (Splooce-specific):
SLQKNQDLEFER (MAXQUANT)

Alignment:
Uniprot       MSNPFAHLAEPLDPVQPGKKFFNLNKLEDSRYGRLPFSIRVLLEAAIRNCDEFLVKKQDIENILHWNVTQHKNIEVPFKPARVILQDFTGVPAVVDFAAM
Splooce       MSNPFAHLAEPLDPVQPGKKFFNLNKLEDSRYGRLPFSIRVLLEAAIRNCDEFLVKKQDIENILHWNVTQHKNIEVPFKPARVILQDFTGVPAVVDFAAM

Uniprot       RDAVKKLGGDPEKINPVCPADLVIDHSIQVDFNRRADSLQKNQDLEFERNRERFEFLKWGSQAFHNMRIIPPGSGIIHQVNLEYLARVVFDQDGYYYPDS
Splooce       RDAVKKLGGDPEKINPVCPADLVIDHSIQVDFNR---SLQKNQDLEFERNRERFEFLKWGSQAFHNMRIIPPGSGIIHQVNLEYLARVVFDQDGYYYPDS

Uniprot       LVGTDSHTTMIDGLGILGWGVGGIEAEAVMLGQPISMVLPQVIGYRLMGKPHPLVTSTDIVLTITKHLRQVGVVGKFVEFFGPGVAQLSIADRATIANMC
Splooce       LVGTDSHTTMIDGLGILGWGVGGIEAEAVMLGQPISMVLPQVIGYRLMGKPHPLVTSTDIVLTITKHLRQVGVVGKFVEFFGPGVAQLSIADRATIANMC

Uniprot       PEYGATAAFFPVDEVSITYLVQTGRDEEKLKYIKKYLQAVGMFRDFNDPSQDPDFTQVVELDLKTVVPCCSGPKRPQDKVAVSDMKKDFESCLGAKQGFK
Splooce       PEYGATAAFFPVDEVSITYLVQTGRDEEKLKYIKKYLQAVGMFRDFNDPSQDPDFTQVVELDLKTVVPCCSGPKRPQDKVAVSDMKKDFESCLGAKQGFK

Uniprot       GFQVAPEHHNDHKTFIYDNTEFTLAHGSVVIAAITSCTNTSNPSVMLGAGLLAKKAVDAGLNVMPYIKTSLSPGSGVVTYYLQESGVMPYLSQLGFDVVG
Splooce       GFQVAPEHHNDHKTFIYDNTEFTLAHGSVVIAAITSCTNTSNPSVMLGAGLLAKKAVDAGLNVMPYIKTSLSPGSGVVTYYLQESGVMPYLSQLGFDVVG

Uniprot       YGCMTCIGNSGPLPEPVVEAITQGDLVAVGVLSGNRNFEGRVHPNTRANYLASPPLVIAYAIAGTIRIDFEKEPLGVNAKGQQVFLKDIWPTRDEIQAVE
Splooce       YGCMTCIGNSGPLPEPVVEAITQGDLVAVGVLSGNRNFEGRVHPNTRANYLASPPLVIAYAIAGTIRIDFEKEPLGVNAKGQQVFLKDIWPTRDEIQAVE

Uniprot       RQYVIPGMFKEVYQKIETVNESWNALATPSDKLFFWNSKSTYIKSPPFFENLTLDLQPPKSIVDAYVLLNLGDSVTTDHISPAGNIARNSPAARYLTNRG
Splooce       RQYVIPGMFKEVYQKIETVNESWNALATPSDKLFFWNSKSTYIKSPPFFENLTLDLQPPKSIVDAYVLLNLGDSVTTDHISPAGNIARNSPAARYLTNRG

Uniprot       LTPREFNSYGSRRGNDAVMARGTFANIRLLNRFLNKQAPQTIHLPSGEILDVFDAAERYQQAGLPLIVLAGKEYGAGSSRDWAAKGPFLLGIKAVLAESY
Splooce       LTPREFNSYGSRRGNDAVMARGTFANIRLLNRFLNKQAPQTIHLPSGEILDVFDAAERYQQAGLPLIVLAGKEYGAGSSRDWAAKGPFLLGIKAVLAESY

Uniprot       ERIHRSNLVGMGVIPLEYLPGENADALGLTGQERYTIIIPENLKPQMKVQVKLDTGKTFQAVMRFDTDVELTYFLNGGILNYMIRKMAK
Splooce       ERIHRSNLVGMGVIPLEYLPGENADALGLTGQERYTIIIPENLKPQMKVQVKLDTGKTFQAVMRFDTDVELTYFLNGGILNYMIRKMAK

----------------------------------------------------------------------------------------------------

P62753 (Uniprot)	versus
NM_001010#(f-T:9_R1409832922374) (Splooce)

For more details about the Alternative Splicing Event -> Link to Splooce page

Peptides that support the ASE (Splooce-specific):
DADFPLCELLLLSLNPVR (MAXQUANT)

Alignment:
Uniprot       MKLNISFPATGCQKLIEVDDERKLRTFYEKRMATEVAADALGEEWKGYVVRISGGNDKQGFPMKQGVLTHGRVRLLLSKGHSCYRPRRTGERKRKSVRGC
Splooce       MKLNISFPATGCQKLIEVDDERKLRTFYEKRMATEVAADALGEEWKGYVVRISGGNDKQGFPMKQGVLTHGRVRLLLSKGHSCYRPRRTGERKRKSVRGC

Uniprot       IVDANLSVLNLVIVKKGEKDIPGLTDTTVPRRLGPKRASRIRKLFNLSKEDDVRQYVVRKPLNKEGKKPRTKAPKIQRLVTPRVLQHKRRRIALKKQRTK
Splooce       IVDANLSVLNLVIVKKGEKDIPGLTDTTVPRRLGPKRASRIRKLFNLSKEDDVRQYVVRKPLNKEGKKPRTKAPKIQRLVTPRVLQHKRRRIALKKQRTK

Uniprot       KNKEEAAEYAKLLAKRMKEAKEKRQEQIAKRRR---LSSLRASTSKSESSQK-
Splooce       KNKEEAAEYAKLLAKRMKVSLRRSARNKLRRDADFPLCELLLLSLNPVRNKIF

----------------------------------------------------------------------------------------------------

Q01780 (Uniprot)	versus
NM_001001998#(-s-s-:1_E3061257518385) (Splooce)

For more details about the Alternative Splicing Event -> Link to Splooce page

Peptides that support the ASE (Splooce-specific):
LYRPIEETPCHFISSLDELVELNEK (MAXQUANT)

Alignment:
Uniprot       MAPPSTREPRVLSATSATKSDGEMVLPGFPDADSFVKFALGSVVAVTKASGGLPQFGDEYDFYRSFPGFQAFCETQGDRLLQCMSRVMQYHGCRSNIKDR
Splooce       ----------------------------------------------------------------------------------------------------

Uniprot       SKVTELEDKFDLLVDANDVILERVGILLDEASGVNKNQQPVLPAGLQVPKTVVSSWNRKAAEYGKKAKSETFRLLHAKNIIRPQLKFREKIDNSNTPFLP
Splooce       ----------------------------------------------------------------------------------------------------

Uniprot       KIFIKPNAQKPLPQALSKERRERPQDRPEDLDVPPALADFIHQQRTQQVEQDMFAHPYQYELNHFTPADAVLQKPQPQLYRPIEETPCHFISSLDELVEL
Splooce       ------------------------MAKKQNLKLSGCFMQKISSDLSSSFERRLTIPTHHFFLK--SSSNPMLRNLSLKLYRPIEETPCHFISSLDELVEL

Uniprot       NEKLLNCQEFAVDLEHHSYRSFLGLTCLMQISTRTEDFIIDTLELRSDMYILNESLTDPAIVKVFHGADSDIEWLQKDFGLYVVNMFDTHQAARLLNLGR
Splooce       NEKLLNCQEFAVDLEHHSYRSFLGLTCLMQISTRTEDFIIDTLELRSDMYILNESLTDPAIVKVFHGADSDIEWLQKDFGLYVVNMFDTHQAARLLNLGR

Uniprot       HSLDHLLKLYCNVDSNKQYQLADWRIRPLPEEMLSYARDDTHYLLYIYDKMRLEMWERGNGQPVQLQVVWQRSRDICLKKFIKPIFTDESYLELYRKQKK
Splooce       HSLDHLLKLYCNVDSNKQYQLADWRIRPLPEEMLSYARDDTHYLLYIYDKMRLEMWERGNGQPVQLQVVWQRSRDICLKKFIKPIFTDESYLELYRKQKK

Uniprot       HLNTQQLTAFQLLFAWRDKTARREDESYGYVLPNHMMLKIAEELPKEPQGIIACCNPVPPLVRQQINEMHLLIQQAREMPLLKSEVAAGVKKSGPLPSAE
Splooce       HLNTQQLTAFQLLFAWRDKTARREDESYGYVLPNHMMLKIAEELPKEPQGIIACCNPVPPLVRQQINEMHLLIQQAREMPLLKSEVAAGVKKSGPLPSAE

Uniprot       RLENVLFGPHDCSHAPPDGYPIIPTSGSVPVQKQASLFPDEKEDNLLGTTCLIATAVITLFNEPSAEDSKKGPLTVAQKKAQNIMESFENPFRMFLPSLG
Splooce       RLENVLFGPHDCSHAPPDGYPIIPTSGSVPVQKQASLFPDEKEDNLLGTTCLIATAVITLFNEPSAEDSKKGPLTVAQKKAQNIMESFENPFRMFLPSLG

Uniprot       HRAPVSQAAKFDPSTKIYEISNRWKLAQVQVQKDSKEAVKKKAAEQTAAREQAKEACKAAAEQAISVRQQVVLENAAKKRERATSDPRTTEQKQEKKRLK
Splooce       HRAPVSQAAKFDPSTKIYEISNRWKLAQVQVQKDSKEAVKKKAAEQTAAREQAKEACKAAAEQAISVRQQVVLENAAKKRERATSDPRTTEQKQEKKRLK

Uniprot       ISKKPKDPEPPEKEFTPYDYSQSDFKAFAGNSKSKVSSQFDPNKQTPSGKKCIAAKKIKQSVGNKSMSFPTGKSDRGFRYNWPQR
Splooce       ISKKPKDPEPPEKEFTPYDYSQSDFKAFAGNSKSKVSSQFDPNKQTPSGKKCIAAKKIKQSVGNKSMSFPTGKSDRGFRYNWPQR

----------------------------------------------------------------------------------------------------

O95163 (Uniprot)	versus
NM_003640#(-s-s-:9_I8234845137928) (Splooce)

For more details about the Alternative Splicing Event -> Link to Splooce page

Peptides that support the ASE (Splooce-specific):
YLSTFGVASNITSFAVYDEFLLLTTHSHTCQCFCLR (MAXQUANT)

Alignment:
Uniprot       MRNLKLFRTLEFRDIQGPGNPQCFSLRTEQGTVLIGSEHGLIEVDPVSREVKNEVSLVAEGFLPEDGSGRIVGVQDLLDQESVCVATASGDVILCSLSTQ
Splooce       ----------------------------------------------------------------------------------------------------

Uniprot       QLECVGSVASGISVMSWSPDQELVLLATGQQTLIMMTKDFEPILEQQIHQDDFGESKFITVGWGRKETQFHGSEGRQAAFQMQMHESALPWDDHRPQVTW
Splooce       ----------------------------------------------------------------------------------------------------

Uniprot       RGDGQFFAVSVVCPETGARKVRVWNREFALQSTSEPVAGLGPALAWKPSGSLIASTQDKPNQQDIVFFEKNGLLHGHFTLPFLKDEVKVNDLLWNADSSV
Splooce       ----------------------------------------------------------------------------------------------------

Uniprot       LAVWLEDLQREESSIPKTCVQLWTVGNYHWYLKQSLSFSTCGKSKIVSLMWDPVTPYRLHVLCQGWHYLAYDWHWTTDRSVGDNSSDLSNVAVIDGNRVL
Splooce       ----------------------------------------------------------------------------------------------------

Uniprot       VTVFRQTVVPPPMCTYQLLFPHPVNQVTFLAHPQKSNDLAVLDASNQISVYKCGDCPSADPTVKLGAVGGSGFKVCLRTPHLEKRYKIQFENNEDQDVNP
Splooce       ----------------------------------------------------------------------------------------------------

Uniprot       LKLGLLTWIEEDVFLAVSHSEFSPRSVIHHLTAASSEMDEEHGQLNVSSSAAVDGVIISLCCNSKTKSVVLQLADGQIFKYLWESPSLAIKPWKNSGGFP
Splooce       ----------------------------------------------------------------------------------------------------

Uniprot       VRFPYPCTQTELAMIGEEECVLGLTDRCRFFINDIEVASNITSFAVYDEFLLLTTHSHTCQCFCLRDASFKTLQAGLSSNHVSHGEVLRKVERGSRIVTV
Splooce       ---------------------------MARYLSTFGVASNITSFAVYDEFLLLTTHSHTCQCFCLRDASFKTLQAGLSSNHVSHGEVLRKVERGSRIVTV

Uniprot       VPQDTKLVLQMPRGNLEVVHHRALVLAQIRKWLDKLMFKEAFECMRKLRINLNLIYDHNPKVFLGNVETFIKQIDSVNHINLFFTELKEEDVTKTMYPAP
Splooce       VPQDTKLVLQMPRGNLEVVHHRALVLAQIRKWLDKLMFKEAFECMRKLRINLNLIYDHNPKVFLGNVETFIKQIDSVNHINLFFTELKEEDVTKTMYPAP

Uniprot       VTSSVYLSRDPDGNKIDLVCDAMRAVMESINPHKYCLSILTSHVKKTTPELEIVLQKVHELQGNAPSDPDAVSAEEALKYLLHLVDVNELYDHSLGTYDF
Splooce       VTSSVYLSRDPDGNKIDLVCDAMRAVMESINPHKYCLSILTSHVKKTTPELEIVLQKVHELQGNAPSDPDAVSAEEALKYLLHLVDVNELYDHSLGTYDF

Uniprot       DLVLMVAEKSQKDPKEYLPFLNTLKKMETNYQRFTIDKYLKRYEKAIGHLSKCGPEYFPECLNLIKDKNLYNEALKLYSPSSQQYQDISIAYGEHLMQEH
Splooce       DLVLMVAEKSQKDPKEYLPFLNTLKKMETNYQRFTIDKYLKRYEKAIGHLSKCGPEYFPECLNLIKDKNLYNEALKLYSPSSQQYQDISIAYGEHLMQEH

Uniprot       MYEPAGLMFARCGAHEKALSAFLTCGNWKQALCVAAQLNFTKDQLVGLGRTLAGKLVEQRKHIDAAMVLEECAQDYEEAVLLLLEGAAWEEALRLVYKYN
Splooce       MYEPAGLMFARCGAHEKALSAFLTCGNWKQALCVAAQLNFTKDQLVGLGRTLAGKLVEQRKHIDAAMVLEECAQDYEEAVLLLLEGAAWEEALRLVYKYN

Uniprot       RLDIIETNVKPSILEAQKNYMAFLDSQTATFSRHKKRLLVVRELKEQAQQAGLDDEVPHGQESDLFSETSSVVSGSEMSGKYSHSNSRISARSSKNRRKA
Splooce       RLDIIETNVKPSILEAQKNYMAFLDSQTATFSRHKKRLLVVRELKEQAQQAGLDDEVPHGQESDLFSETSSVVSGSEMSGKYSHSNSRISARSSKNRRKA

Uniprot       ERKKHSLKEGSPLEDLALLEALSEVVQNTENLKDEVYHILKVLFLFEFDEQGRELQKAFEDTLQLMERSLPEIWTLTYQQNSATPVLGPNSTANSIMASY
Splooce       ERKKHSLKEGSPLEDLALLEALSEVVQNTENLKDEVYHILKVLFLFEFDEQGRELQKAFEDTLQLMERSLPEIWTLTYQQNSATPVLGPNSTANSIMASY

Uniprot       QQQKTSVPVLDAELFIPPKINRRTQWKLSLLD
Splooce       QQQKTSVPVLDAELFIPPKINRRTQWKLSLLD

----------------------------------------------------------------------------------------------------

Q96CN5 (Uniprot)	versus
NM_144999#(-s-s-s-s-:17_L6248799376559) (Splooce)

For more details about the Alternative Splicing Event -> Link to Splooce page

Peptides that support the ASE (Splooce-specific):
FLDLKFLDLMETIDK (MAXQUANT)

Alignment:
Uniprot       MEEFRRSYSRLCRESGAEPQEAVLQQLHQLPRGRLDLATQSLTVETCRALGKLLPRETLCTELVLSDCMLSEEGATLLLRGLCANTVLRFLDLKGNNLRA
Splooce       MEEFRRSYSRLCRESGAEPQEAVLQQLHQLPRGRLDLATQSLTVETCRALGKLLPRETLCTELVLSDCMLSEEGATLLLRGLCANTVLRFLDLK------

Uniprot       AGAEALGKLLQQNKSIQSLTLEWNSLGTWDDAFATFCGGLAANGALQRLDLRNNQISHKGAEELALALKGNTTLQQLDLRWNNVGLLGGRALMNCLPSNR
Splooce       ----------------------------------------------------------------------------------------------------

Uniprot       TLWRLDLAGNNIPGDVLRAVEQAMGHSQDRLTTFQENQARTHVLSKEVQHLREEKSKQFLDLMETIDKQREEMAKSSRASAARVGQLQEALNERHSIINA
Splooce       ----------------------------------------------------------FLDLMETIDKQREEMAKSSRASAARVGQLQEALNERHSIINA

Uniprot       LKAKLQMTEAALALSEQKAQDLGELLATAEQEQLSLSQRQAKELKLEQQEAAERESKLLRDLSAANEKNLLLQNQVDELERKFRCQQEQLFQTRQEMTSM
Splooce       LKAKLQMTEAALALSEQKAQDLGELLATAEQEQLSLSQRQAKELKLEQQEAAERESKLLRDLSAANEKNLLLQNQVDELERKFRCQQEQLFQTRQEMTSM

Uniprot       SAELKMRAIQAEERLDMEKRRCRQSLEDSESLRIKEVEHMTRHLEESEKAMQERVQRLEAARLSLEEELSRVKAAALSERGQAEEELIKAKSQARLEEQQ
Splooce       SAELKMRAIQAEERLDMEKRRCRQSLEDSESLRIKEVEHMTRHLEESEKAMQERVQRLEAARLSLEEELSRVKAAALSERGQAEEELIKAKSQARLEEQQ

Uniprot       RLAHLEDKLRLLAQARDEAQGACLQQKQVVAEAQTRVSQLGLQVEGLRRRLEELQQELSLKDQERVAEVSRVRVELQEQNGRLQAELAAQEALREKAAAL
Splooce       RLAHLEDKLRLLAQARDEAQGACLQQKQVVAEAQTRVSQLGLQVEGLRRRLEELQQELSLKDQERVAEVSRVRVELQEQNGRLQAELAAQEALREKAAAL

Uniprot       ERQLKVMASDHREALLDRESENASLREKLRLREAEIARIRDEEAQRASFLQNAVLAYVQASPVRTLSPPK
Splooce       ERQLKVMASDHREALLDRESENASLREKLRLREAEIARIRDEEAQRASFLQNAVLAYVQASPVRTLSPPK

----------------------------------------------------------------------------------------------------

Q8N3D4 (Uniprot)	versus
NM_001099409#(-s-s-:11_E902788088512) (Splooce)

For more details about the Alternative Splicing Event -> Link to Splooce page

Peptides that support the ASE (Splooce-specific):
SLMESDWQK (MAXQUANT)

Alignment:
Uniprot       MTSVWKRLQRVGKRAAKFQFVACYHELVLECTKKWQPDKLVVVWTRRNRRICSKAHSWQPGIQNPYRGTVVWMVPENVDISVTLYRDPHVDQYEAKEWTF
Splooce       MTSVWKRLQRVGKRAAKFQFVACYHELVLECTKKWQPDKLVVVWTRRNRRICSKAHSWQPGIQNPYRGTVVWMVPENVDISVTLYRDPHVDQYEAKEWTF

Uniprot       IIENESKGQRKVLATAEVDLARHAGPVPVQVPVRLRLKPKSVKVVQAELSLTLSGVLLREGRATDDDMQSLASLMSVKPSDVGNLDDFAESDEDEAHGPG
Splooce       IIENESKGQRKVLATAEVDLARHAGPVPVQVPVRLRLKPKSVKVVQAELSLTLSGVLLREGRATDDDMQSLASLMSVKPSDVGNLDDFAESDEDEAHGPG

Uniprot       APEARARVPQPDPSRELKTLCEEEEEGQGRPQQAVASPSNAEDTSPAPVSAPAPPARTSRGQGSERANEAGGQVGPEAPRPPETSPEMRSSRQPAQDTAP
Splooce       APEARARVPQPDPSRELKTLCEEEEEGQGRPQQAVASPSNAEDTSPAPVSAPAPPARTSRGQGSERANEAGGQVGPEAPRPPETSPEMRSSRQPAQDTAP

Uniprot       TPAPRLRKGSDALRPPVPQGEDEVPKASGAPPAGLGSARETQAQACPQEGTEAHGARLGPSIEDKGSGDPFGRQRLKAEEMDTEDRPEASGVDTEPRSGG
Splooce       TPAPRLRKGSDALRPPVPQGEDEVPKASGAPPAGLGSARETQAQACPQEGTEAHGARLGPSIEDKGSGDPFGRQRLKAEEMDTEDRPEASGVDTEPRSGG

Uniprot       REANTKRSGVRAGEAEESSAVCQVDAEQRSKVRHVDTKGPEATGVMPEARCRGTPEAPPRGSQGRLGVRTRDEAPSGLSLPPAEPAGHSGQLGDLEGARA
Splooce       REANTKRSGVRAGEAEESSAVCQVDAEQRSKVRHVDTKGPEATGVMPEARCRGTPEAPPRGSQGRLGVRTRDEAPSGLSLPPAEPAGHSGQLGDLEGARA

Uniprot       AAGQEREGAEVRGGAPGIEGTGLEQGPSVGAISTRPQVSSWQGALLSTAQGAISRGLGGWEAEAGGSGDLETETEVVGLEVLGTQEKEVEGSGFPETRTL
Splooce       AAGQEREGAEVRGGAPGIEGTGLEQGPSVGAISTRPQVSSWQGALLSTAQGAISRGLGGWEAEAGGSGDLETETEVVGLEVLGTQEKEVEGSGFPETRTL

Uniprot       EIEILGALEKEAARSRVLESEVAGTAQCEGLETQETEVGVIETPGTETEVLGTQKTEAGGSGVLQTRTTIAETEVLVTQEISGDLGPLKIEDTIQSEMLG
Splooce       EIEILGALEKEAARSRVLESEVAGTAQCEGLETQETEVGVIETPGTETEVLGTQKTEAGGSGVLQTRTTIAETEVLVTQEISGDLGPLKIEDTIQSEMLG

Uniprot       TQETEVEASRVPESEAEGTEAKILGTQEITARDSGVREIEAEIAESDILVAQEIEVGLLGVLGIETGAAEGAILGTQEIASRDSGVPGLEADTTGIQVKE
Splooce       TQETEVEASRVPESEAEGTEAKILGTQEITARDSGVREIEAEIAESDILVAQEIEVGLLGVLGIETGAAEGAILGTQEIASRDSGVPGLEADTTGIQVKE

Uniprot       VGGSEVPEIATGTAETEILGTQEIASRSSGVPGLESEVAGAQETEVGGSGISGPEAGMAEARVLMTRKTEIIVPEAEKEEAQTSGVQEAETRVGSALKYE
Splooce       VGGSEVPEIATGTAETEILGTQEIASRSSGVPGLESEVAGAQETEVGGSGISGPEAGMAEARVLMTRKTEIIVPEAEKEEAQTSGVQEAETRVGSALKYE

Uniprot       ALRAPVTQPRVLGSQEAKAEISGVQGSETQVLRVQEAEAGVWGMSEGKSGAWGAQEAEMKVLESPENKSGTFKAQEAEAGVLGNEKGKEAEGSLTEASLP
Splooce       ALRAPVTQPRVLGSQEAKAEISGVQGSETQVLRVQEAEAGVWGMSEGKSGAWGAQEAEMKVLESPENKSGTFKAQEAEAGVLGNEKGKEAEGSLTEASLP

Uniprot       EAQVASGAGAGAPRASSPEKAEEDRRLPGSQAPPALVSSSQSLLEWCQEVTTGYRGVRITNFTTSWRNGLAFCAILHRFYPDKIDYASLDPLNIKQNNKQ
Splooce       EAQVASGAGAGAPRASSPEKAEEDRRLPGSQAPPALVSSSQSLLEWCQEVTTGYRGVRITNFTTSWRNGLAFCAILHRFYPDKIDYASLDPLNIKQNNKQ

Uniprot       AFDGFAALGVSRLLEPADMVLLSVPDKLIVMTYLCQIRAFCTGQELQLVQLEGGGGAGTYRVGSAQPSPPDDLDAGGLAQRLRGHGAEGPQEPKEAADRA
Splooce       AFDGFAALGVSRLLEPADMVLLSVPDKLIVMTYLCQIRAFCTGQELQLVQLEGGGGAGTYRVGSAQPSPPDDLDAGGLAQRLRGHGAEGPQEPKEAADRA

Uniprot       DGAAPGVASRNAVAGRASKDGGAEAPRESRPAEVPAEGLVNGAGAPGGGGVRLRRPSVNGEPGSVPPPRAHGSFSHVRDADLLKKRRSRLRNSSSFSMDD
Splooce       DGAAPGVASRNAVAGRASKDGGAEAPRESRPAEVPAEGLVNGAGAPGGGGVRLRRPSVNGEPGSVPPPRAHGSFSHVRDADLLKKRRSRLRNSSSFSMDD

Uniprot       PDAGAMGAAAAEGQAPDPSPAPGPPTAADSQQPPGGSSPSEEPPPSPGEEAGLQRFQDTSQYVCAELQALEQEQRQIDGRAAEVEMQLRSLMESGANKLQ
Splooce       PDAGAMGAAAAEGQAPDPSPAPGPPTAADSQQPPGGSSPSEEPPPSPGEEAGLQRFQDTSQYVCAELQALEQEQRQIDGRAAEVEMQLRSLMES------

Uniprot       EEVLIQEWFTLVNKKNALIRRQDQLQLLMEEQDLERRFELLSRELRAMLAIEDWQKTSAQQHREQLLLEELVSLVNQRDELVRDLDHKERIALEEDERLE
Splooce       ----------------------------------------------------DWQKTSAQQHREQLLLEELVSLVNQRDELVRDLDHKERIALEEDERLE

Uniprot       RGLEQRRRKLSRQLSRRERCVLS
Splooce       RGLEQRRRKLSRQLSRRERCVLS

----------------------------------------------------------------------------------------------------

Q8N163 (Uniprot)	versus
NM_199205#(-t:8_K5324873092948) (Splooce)

For more details about the Alternative Splicing Event -> Link to Splooce page

Peptides that support the ASE (Splooce-specific):
DDGEEEFGAK (MAXQUANT)

Alignment:
Uniprot       MSQFKRQRINPLPGGRNFSGTASTSLLGPPPGLLTPPVATELSQNARHLQGGEKQRVFTGIVTSLHDYFGVVDEEVFFQLSVVKGRLPQLGEKVLVKAAY
Splooce       MSQFKRQRINPLPGGRNFSGTASTSLLGPPPGLLTPPVATELSQNARHLQGGEKQRVFTGIVTSLHDYFGVVDEEVFFQLSVVKGRLPQLGEKVLVKAAY

Uniprot       NPGQAVPWNAVKVQTLSNQPLLKSPAPPLLHVAALGQKQGILGAQPQLIFQPHRIPPLFPQKPLSLFQTSHTLHLSHLNRFPARGPHGRLDQGRSDDYDS
Splooce       NPGQAVPWNAVKVQTLSNQPLLKSPAPPLLHVAALGQKQGILGAQPQLIFQPHRIPPLFPQKPLSLFQTSHTLHLSHLNRFPARGPHGRLDQGRSDDYDS

Uniprot       KKRKQRAGGEPWGAKKPRHDLPPYRVHLTPYTVDSPICDFLELQRRYRSLLVPSDFLSVHLSWLSAFPLSQPFSLHHPSRIQVSSEKEAAPDAGAEPITA
Splooce       KKRKQRAGGEPWGAKKPRHDLPPYRVHLTPYTVDSPICDFLELQRRYRSLLVPSDFLSVHLSWLSAFPLSQPFSLHHPSRIQVSSEKEAAPDAGAEPITA

Uniprot       DSDPAYSSKVLLLSSPGLEELYRCCMLFVDDMAEPRETPEHPLKQIKFLLGRKEEEAVLVGGEWSPSLDGLDPQADPQVLVRTAIRCAQAQTGIDLSGCT
Splooce       DSDPAYSSKVLLLSSPGLEELYRCCMLFVDDMAEPRETPEHPLKQIKFLLGRKEEEAVLVGGEWSPSLDGLDPQADPQVLVRTAIRCAQAQTGIDLSGCT

Uniprot       KWWRFAEFQYLQPGPPRRLQTVVVYLPDVWTIMPTLEEWEALCQQKAAEAAPPTQEAQGETEPTEQAPDALEQAADTSRRNAETPEATTQQETDTDLPEA
Splooce       KWWRFAEFQYLQPGPPRRLQTVVVYLPDVWTIMPTLEEWEALCQQKAAEAAPPTQEAQGETEPTEQAPDALEQAADTSRRNAETPEATTQQETDTDLPEA

Uniprot       PPPPLEPAVIARPGCVNLSLHGIVEDRRPKERISFEVMVLAELFLEMLQRDFGYRVYKMLLSLPEKVVSPPEPEKEEAAKEEATKEEEAIKEEVVKEPKD
Splooce       PPPPLEPAVIARPGCVNLSLHGIVEDRRPKERISFEVMVLAELFLEMLQRDFGYRVYKMLLSLPEKVVSPPEPEKEEAAKEEATKEEEAIKEEVVKEPKD

Uniprot       EAQNEGPATESEAPLKEDGLLPKPLSSGGEEEEKPRGEASEDLCEMALDPELLLLRDDGEEEFAGAKLEDSEVRSVASNQSEMEFSSLQDMPKELDPSAV
Splooce       EAQNEGPATESEAPLKEDGLLPKPLSSGGEEEEKPRGEASEDLCEMALDPELLLLRDDGEEEFG-AKLEDSEVRSVASNQSEMEFSSLQDMPKELDPSAV

Uniprot       LPLDCLLAFVFFDANWCGYLHRRDLERILLTLGIRLSAEQAKQLVSRVVTQNICQYRSLQYSRQEGLDGGLPEEVLFGNLDLLPPPGKSTKPGAAPTEHK
Splooce       LPLDCLLAFVFFDANWCGYLHRRDLERILLTLGIRLSAEQAKQLVSRVVTQNICQYRSLQYSRQEGLDGGLPEEVLFGNLDLLPPPGKSTKPGAAPTEHK

Uniprot       ALVSHNGSLINVGSLLQRAEQQDSGRLYLENKIHTLELKLEESHNRFSATEVTNKTLAAEMQELRVRLAEAEETARTAERQKSQLQRLLQELRRRLTPLQ
Splooce       ALVSHNGSLINVGSLLQRAEQQDSGRLYLENKIHTLELKLEESHNRFSATEVTNKTLAAEMQELRVRLAEAEETARTAERQKSQLQRLLQELRRRLTPLQ

Uniprot       LEIQRVVEKADSWVEKEEPAPSN
Splooce       LEIQRVVEKADSWVEKEEPAPSN

----------------------------------------------------------------------------------------------------

Q86SQ9 (Uniprot)	versus
NM_205861#(-s-:1_D1657540305187) (Splooce)

For more details about the Alternative Splicing Event -> Link to Splooce page

Peptides that support the ASE (Splooce-specific):
LSDFLLWQK (MAXQUANT)

Alignment:
Uniprot       MSWIKEGELSLWERFCANIIKAGPMPKHIAFIMDGNRRYAKKCQVERQEGHSQGFNKLAETLRWCLNLGILEVTVYAFSIENFKRSKSEVDGLMDLARQK
Splooce       MSWIKEGELSLWERFCANIIKAGPMPKHIAFIMDGNRRYAKKCQVERQEGHSQGFNKLAETLRWCLNLGILEVTVYAFSIENFKRSKSEVDGLMDLARQK

Uniprot       FSRLMEEKEKLQKHGVCIRVLGDLHLLPLDLQELIAQAVQATKNYNKCFLNVCFAYTSRHEISNAVREMAWGVEQGLLDPSDISESLLDKCLYTNRSPHP
Splooce       FSRLMEEKEKLQKHGVCIRVLGDLHLLPLDLQELIAQAVQATKNYNKCFLNVCFAYTSRHEISNAVREMAWGVEQGLLDPSDISESLLDKCLYTNRSPHP

Uniprot       DILIRTSGEVRLSDFLLWQTSHSCLVFQPVLWPEYTFWNLFEAILQFQMNHSVLQKARDMYAEERKRQQLERDQATVTEQLLREGLQASGDAQLRRTRLH
Splooce       DILIRTSGEVRLSDFLLWQ------------------------------------KARDMYAEERKRQQLERDQATVTEQLLREGLQASGDAQLRRTRLH

Uniprot       KLSARREERVQGFLQALELKRADWLARLGTASA
Splooce       KLSARREERVQGFLQALELKRADWLARLGTASA

----------------------------------------------------------------------------------------------------

O43707 (Uniprot)	versus
NM_004924#(-s-s-s-s-:19_A1890062583411) (Splooce)

For more details about the Alternative Splicing Event -> Link to Splooce page

Peptides that support the ASE (Splooce-specific):
SGASPLR (MAXQUANT)

Alignment:
Uniprot       MVDYHAANQSYQYGPSSAGNGAGGGGSMGDYMAQEDDWDRDLLLDPAWEKQQRKTFTAWCNSHLRKAGTQIENIDEDFRDGLKLMLLLEVISGERLPKPE
Splooce       MVDYHAANQSYQYGPSSAGNGAGGGGSMGDYMAQEDDWDRDLLLDPAWEKQQRKTFTAWCNSHLRKAGTQIENIDEDFRDGLKLMLLLEVISGERLPKPE

Uniprot       RGKMRVHKINNVNKALDFIASKGVKLVSIGAEEIVDGNAKMTLGMIWTIILRFAIQDISVEETSAKEGLLLWCQRKTAPYKNVNVQNFHISWKDGLAFNA
Splooce       RGKMRVHKINNVNKALDFIASKGVKLVSIGAEEIVDGNAKMTLGMIWTIILRFAIQDISVEETSAKEGLLLWCQRKTAPYKNVNVQNFHISWKDGLAFNA

Uniprot       LIHRHRPELIEYDKLRKDDPVTNLNNAFEVAEKYLDIPKMLDAEDIVNTARPDEKAIMTYVSSFYHAFSGAQKAETAANRICKVLAVNQENEHLMEDYEK
Splooce       LIHRHRPELIEYDKLRKDDPVTNLNNAFEVAEKYLDIPKMLDAEDIVNTARPDEKAIMTYVSSFYHAFSGAQKAETAANRICKVLAVNQENEHLMEDYEK

Uniprot       LASDLLEWIRRTIPWLEDRVPQKTIQEMQQKLEDFRDYRRVHKPPKVQEKCQLEINFNTLQTKLRLSNRPAFMPSEGKMVSDINNGWQHLEQAEKGYEEW
Splooce       LASDLLEWIRRTIPWLEDRVPQKTIQEMQQKLEDFRDYRRVHKPPKVQEKCQLEINFNTLQTKLRLSNRPAFMPSEGKMVSDINNGWQHLEQAEKGYEEW

Uniprot       LLNEIRRLERLDHLAEKFRQKASIHEAWTDGKEAMLKHRDYETATLSDIKALIRKHEAFESDLAAHQDRVEQIAAIAQELNELDYYDSHNVNTRCQKICD
Splooce       LLNEIRRLERLDHLAEKFRQKASIHEAWTDGKEAMLKHRDYETATLSDIKALIRKHEAFESDLAAHQDRVEQIAAIAQEL--------------------

Uniprot       QWDALGSLTHSRREALEKTEKQLEAIDQLHLEYAKRAAPFNNWMESAMEDLQDMFIVHTIEEIEGLISAHDQFKSTLPDADREREAILAIHKEAQRIAES
Splooce       ----------------------------------------------------------------------------------------------------

Uniprot       NHIKLSGSNPYTTVTPQIINSKWEKVQQLVPKRDHALLEEQSKQQSNEHLRRQFASQANVVGPWIQTKMEEIGRISIEMNGTLEDQLSHLKQYERSIVDY
Splooce       ---KRSGASPLR----------------------------------------------------------------------------------------

Uniprot       KPNLDLLEQQHQLIQEALIFDNKHTNYTMEHIRVGWEQLLTTIARTINEVENQILTRDAKGISQEQMQEFRASFNHFDKDHGGALGPEEFKACLISLGYD
Splooce       ----------------------------------------------------------------------------------------------------

Uniprot       VENDRQGEAEFNRIMSLVDPNHSGLVTFQAFIDFMSRETTDTDTADQVIASFKVLAGDKNFITAEELRRELPPDQAEYCIARMAPYQGPDAVPGALDYKS
Splooce       ----------------------------------------------------------------------------------------------------

Uniprot       FSTALYGESDL
Splooce       -----------

----------------------------------------------------------------------------------------------------

Q14320 (Uniprot)	versus
NM_004699#(-s-s-s-s-:X_F9413055759032) (Splooce)

For more details about the Alternative Splicing Event -> Link to Splooce page

Peptides that support the ASE (Splooce-specific):
PLFNFDVHDDVR (MAXQUANT)

Alignment:
Uniprot       MAQYKGAASEAGRAMHLMKKREKQREQMEQMKQRIAEENIMKSNIDKKFSAHYDAVEAELKSSTVGLVTLNDMKAKQEALVKEREKQLAKKEQSKELQMK
Splooce       MAQYKGAASEAGRAMHLMKKREKQREQMEQMKQRIAEENIMKSNIDKKFSAHYDAVEAELKSSTVGLVTLNDMKAKQEALVKEREKQLAKKEQSKELQMK

Uniprot       LEKLREKERKKEAKRKISSLSFTLEEEEEGGEEEEEAAMYEEEMEREEITTKKRKLGKNPDVDTSFLPDRDREEEENRLREELRQEWEAKQEKIKSEEIE
Splooce       LEKLREKERKKEAKRKISSLSFTLEEEEEGGEEEEEAAMYEEEMEREEITTKKRKLGKNPDVDTSFLPDRDREEEENRLREELRQEWEAKQEKIKR----

Uniprot       ITFSYWDGSGHRRTVKMRKGNTMQQFLQKALEILRKDFSELRSAGVEQLMYIKEDLIIPHHHSFYDFIVTKARGKSGPLFNFDVHDDVRLLSDATVEKDE
Splooce       -----------------------------------------------------------------------------PLFNFDVHDDVRLLSDATVEKDE

Uniprot       SHAGKVVLRSWYEKNKHIFPASRWEPYDPEKKWDKYTIR
Splooce       SHAGKVVLRSWYEKNKHIFPASRWEPYDPEKKWDKYTIR

----------------------------------------------------------------------------------------------------

P18124 (Uniprot)	versus
NM_000971#(-t:8_R2656822341903) (Splooce)

For more details about the Alternative Splicing Event -> Link to Splooce page

Peptides that support the ASE (Splooce-specific):
IVEPYIAWGQNQ (MAXQUANT)

Alignment:
Uniprot       MEGVEEKKKEVPAVPETLKKKRRNFAELKIKRLRKKFAQKMLRKARRKLIYEKAKHYHKEYRQMYRTEIRMARMARKAGNFYVPAEPKLAFVIRIRGING
Splooce       MEGVEEKKKEVPAVPETLKKKRRNFAELKIKRLRKKFAQKMLRKARRKLIYEKAKHYHKEYRQMYRTEIRMARMARKAGNFYVPAEPKLAFVIRIRGING

Uniprot       VSPKVRKVLQLLRLRQIFNGTFVKLNKASINMLRIVEPYIAWGYPNLKSVNELIYKRGYGKINKKRIALTDNALIARSLGKYGIICMEDLIHEIYTVGKR
Splooce       VSPKVRKVLQLLRLRQIFNGTFVKLNKASINMLRIVEPYIAWGQ------NQ------------------------------------------------

Uniprot       FKEANNFLWPFKLSSPRGGMKKKTTHFVEGGDAGNREDQINRLIRRMN
Splooce       ------------------------------------------------

----------------------------------------------------------------------------------------------------

P39023 (Uniprot)	versus
NM_001033853#(-s-:22_R3279967131510) (Splooce)

For more details about the Alternative Splicing Event -> Link to Splooce page

Peptides that support the ASE (Splooce-specific):
VIAHTQVSPK (MAXQUANT)

Alignment:
Uniprot       ----------------------------------------------------------------------------------------------------
Splooce       MSHRKFSAPRHGSLGFLPRKRSSRHRGKVKSFPKDDPSKPVHLTAFLGYKAGMTHIVREVDRPGSKVNKKEVVEAVTIVETPPMVVVGIVGYVETPRGLR

Uniprot       ---------------------------------------------------------------------------MEIQVNGGTVAEKLDWARERLEQQV
Splooce       TFKTVFAEHISDECKRRFYKNWHKSKKKAFTKYCKKWQDEDGKKQLEKDFSSMKKYCQVIRVIAHTQVSPKKAHLMEIQVNGGTVAEKLDWARERLEQQV

Uniprot       PVNQVFGQDEMIDVIGVTKGKGYKGVTSRWHTKKLPRKTHRGLRKVACIGAWHPARVAFSVARAGQKGYHHRTEINKKIYKIGQGYLIKDGKLIKNNAST
Splooce       PVNQVFGQDEMIDVIGVTKGKGYKGVTSRWHTKKLPRKTHRGLRKVACIGAWHPARVAFSVARAGQKGYHHRTEINKKIYKIGQGYLIKDGKLIKNNAST

Uniprot       DYDLSDKSINPLGGFVHYGEVTNDFVMLKGCVVGTKKRVLTLRKSLLVQTKRRALEKIDLKFIDTTSKFGHGRFQTMEEKKAFMGPLKKDRIAKEEGA
Splooce       DYDLSDKSINPLGGFVHYGEVTNDFVMLKGCVVGTKKRVLTLRKSLLVQTKRRALEKIDLKFIDTTSKFGHGRFQTMEEKKAFMGPLKKDRIAKEEGA

----------------------------------------------------------------------------------------------------

Q9NU22 (Uniprot)	versus
NM_014611#(-s-s-s-s-:6_M3020782122858) (Splooce)

For more details about the Alternative Splicing Event -> Link to Splooce page

Peptides that support the ASE (Splooce-specific):
ESTIHTAHQFLMDTIFQFWR (MAXQUANT)

Alignment:
Uniprot       MEHFLLEVAAAPLRLIAAKNEKSRSELGRFLAKQVWTPQDRQCVLSTLAQLLLDKDCTVLVGRQLRPLLLDLLERNAEAIKAGGQINHDLHERLCVSMSK
Splooce       MEHFLLEVAAAPLRLIAAKNEKSRSELGRFLAKQVWTPQDRQCVLSTLAQLLLDKDCTVLVGRQLRPLLLDLLERNAEAIKAGGQINHDLHERLCVSMSK

Uniprot       LIGNHPDVLPFALRYFKDTSPVFQRLFLESSDANPVRYGRRRMKLRDLMEAAFKFLQQEQSVFRELWDWSVCVPLLRSHDTLVRWYTANCLALVTCMNEE
Splooce       LIGNHPDVLPFALRYFKDTSPVFQRLFLESSDANPVRYGRRRMKLRDLMEAAFKFLQQEQSVFRELWDWSVCVPLLRSHDTLVRWYTANCLALVTCMNEE

Uniprot       HKLSFLKKIFNSDELIHFRLRLLEEAQLQDLEKALVLANPEVSLWRKQKELQYLQGHLVSSDLSPRVTAVCGVVLPGQLPAPGELGGNRSSSREQELALR
Splooce       HKLSFLKKIFNSDELIHFRLRLLEEAQLQDLEKALVLANPEVSLWRKQKELQYLQGHLVSSDLSPRVTAVCGVVLPGQLPAPGELGGNRSSSREQELALR

Uniprot       SYVLVESVCKSLQTLAMAVASQNAVLLEGPIGCGKTSLVEYLAAVTGRTKPPQLLKVQLGDQTDSKMLLGMYRCTDVPGEFVWQPGTLTQAATMGHWILL
Splooce       SYVLVESVCKSLQTLAMAVASQNAVLLEGPIGCGKTSLVEYLAAVTGRTKPPQLLKVQLGDQTDSKMLLGMYRCTDVPGEFVWQPGTLTQAATMGHWILL

Uniprot       EDIDYAPLDVVSVLIPLLENGELLIPGRGDCLKVAPGFQFFATRRLLSCGGNWYRPLNSHATLLDKYWTKIHLDNLDKRELNEVLQSRYPSLLAVVDHLL
Splooce       EDIDYAPLDVVSVLIPLLENGELLIPGRGDCLKVAPGFQFFATRRLLSCGGNWYRPLNSHATLLDKYWTKIHLDNLDKRELNEVLQSRYPSLLAVVDHLL

Uniprot       DIYIQLTGEKHHSWSDSSVGCEQAPEEVSEARRENKRPTLEGRELSLRDLLNWCNRIAHSFDSSSLSASLNIFQEALDCFTAMLSEHTSKLKMAEVIGSK
Splooce       DIYIQLTGEKHHSWSDSSVGCEQAPEEVSEARRENKRPTLEGRELSLRDLLNWCNRIAHSFDSSSLSASLNIFQEALDCFTAMLSEHTSKLKMAEVIGSK

Uniprot       LNISRKKAEFFCQLYKPEIVINELDLQVGRVRLLRKQSEAVHLQREKFTFAATRPSSVLIEQLAVCVSKGEPVLLVGETGTGKTSTIQYLAHITGHRLRV
Splooce       LNISRKKAEFFCQLYKPEIVINELDLQVGRVRLLRKQSEAVHLQREKFTFAATRPSSVLIEQLAVCVSKGEPVLLVGETGTGKTSTIQYLAHITGHRLRV

Uniprot       VNMNQQSDTADLLGGYKPVDHKLIWLPLREAFEELFAQTFSKKQNFTFLGHIQTCYRQKRWHDLLRLMQHVHKSAVNKDGKDSETGLLIKEKWEAFGLRL
Splooce       VNMNQQSDTADLLGGYKPVDHKLIWLPLREAFEELFAQTFSKKQNFTFLGHIQTCYRQKRWHDLLRLMQHVHKSAVNKDGKDSETGLLIKEKWEAFGLRL

Uniprot       NHAQQQMKMTENTLLFAFVEGTLAQAVKKGEWILLDEINLAAPEILECLSGLLEGSSGSLVLLDRGDTEPLVRHPDFRLFACMNPATDVGKRNLPPGIRN
Splooce       NHAQQQMKMTENTLLFAFVEGTLAQAVKKGEWILLDEINLAAPEILECLSGLLEGSSGSLVLLDRGDTEPLVRHPDFRLFACMNPATDVGKRNLPPGIRN

Uniprot       RFTELYVEELESKEDLQVLIVDYLKGLSVNKNTVQGIINFYTALRKESGTKLVDGTGHRPHYSLRTLCRALRFAASNPCGNIQRSLYEGFCLGFLTQLDR
Splooce       RFTELYVEELESKEDLQVLIVDYLKGLSVNKNTVQGIINFYTALRKESGTKLVDGTGHRPHYSLRTLCRALRFAASNPCGNIQRSLYEGFCLGFLTQLDR

Uniprot       ASHPIVQKLICQHIVPGNVKSLLKQPIPEPKGGRLIQVEGYWIAVGDKEPTIDETYILTSSVKLNLRDIVRVVSAGTYPVLIQGETSVGKTSLIQWLAAA
Splooce       ASHPIVQKLICQHIVPGNVKSLLKQPIPEPKGGRLIQVEGYWIAVGDKEPTIDETYILTSSVKLNLRDIVRVVSAGTYPVLIQGETSVGKTSLIQWLAAA

Uniprot       TGNHCVRINNHEHTDIQEYIGCYTSDSSGKLVFKEGVLIDAMRKGYWIILDELNLAPTDVLEALNRLLDDNRELLVTETQEVVKAHPRFMLFATQNPPGL
Splooce       TGNHCVRINNHEHTDIQEYIGCYTSDSSGKLVFKEGVLIDAMRKGYWIILDELNLAPTDVLEALNRLLDDNRELLVTETQEVVKAHPRFMLFATQNPPGL

Uniprot       YGGRKVLSRAFRNRFVELHFDELPSSELETILHKRCSLPPSYCSKLVKVMLDLQSYRRSSSVFAGKQGFITLRDLFRWAERYRLAEPTEKEYDWLQHLAN
Splooce       YGGRKVLSRAFRNRFVELHFDELPSSELETILHKRCSLPPSYCSKLVKVMLDLQSYRRSSSVFAGKQGFITLRDLFRWAERYRLAEPTEKEYDWLQHLAN

Uniprot       DGYMLLAGRVRKQEEIDVIQEVLEKHFKKKLCPQSLFSKENVLKLLGKLSTQISTLECNFGHIVWTEGMRRLAMLVGRALEFGEPVLLVGDTGCGKTTIC
Splooce       DGYMLLAGRVRKQEEIDVIQEVLEKHFKKKLCPQSLFSKENVLKLLGKLSTQISTLECNFGHIVWTEGMRRLAMLVGRALEFGEPVLLVGDTGCGKTTIC

Uniprot       QVFAALANQKLYSVSCHLHMETSDFLGGLRPVRQKPNDKEEIDTSRLFEWHDGPLVQAMKEDGFFLLDEISLADDSVLERLNSVLEVEKSLVLAEKGSPE
Splooce       QVFAALANQKLYSVSCHLHMETSDFLGGLRPVRQKPNDKEEIDTSRLFEWHDGPLVQAMKEDGFFLLDEISLADDSVLERLNSVLEVEKSLVLAEKGSPE

Uniprot       DKDSEIELLTAGKKFRILATMNPGGDFGKKELSPALRNRFTEIWCPQSTSREDLIQIISHNLRPGLCLGRIDPKGSDIPEVMLDFIDWLTHQEFGRKCVV
Splooce       DKDSEIELLTAGKKFRILATMNPGGDFGKKELSPALRNRFTEIWCPQSTSREDLIQIISHNLRPGLCLGRIDPKGSDIPEVMLDFIDWLTHQEFGRKCVV

Uniprot       SIRDILSWVNFMNKMGEEAALKRPEIISTVTSFVHAACLVYIDGIGSGVTSSGFGTALLARKECLKFLIKRLAKIVRLTEYQKNELKIYDRMKAKEFTGI
Splooce       SIRDILSWVNFMNKMGEEAALKRPEIISTVTSFVHAACLVYIDGIGSGVTSSGFGTALLARKECLKFLIKRLAKIVRLTEYQKNELKIYDRMKAKEFTGI

Uniprot       DNLWGIHPFFIPRGPVLHRNNIADYALSAGTTAMNAQRLLRATKLKKPILLEGSPGVGKTSLVGALAKASGNTLVRINLSEQTDITDLFGADLPVEGGKG
Splooce       DNLWGIHPFFIPRGPVLHRNNIADYALSAGTTAMNAQRLLRATKLKKPILLEGSPGVGKTSLVGALAKASGNTLVRINLSEQTDITDLFGADLPVEGGKG

Uniprot       GEFAWRDGPLLAALKAGHWVVLDELNLASQSVLEGLNACFDHRGEIYVPELGMSFQVQHEKTKIFGCQNPFRQGGGRKGLPRSFLNRFTQVFVDPLTVID
Splooce       GEFAWRDGPLLAALKAGHWVVLDELNLASQSVLEGLNACFDHRGEIYVPELGMSFQVQHEKTKIFGCQNPFRQGGGRKGLPRSFLNRFTQVFVDPLTVID

Uniprot       MEFIASTLFPAIEKNIVKKMVAFNNQIDHEVTVEKKWGQKGGPWEFNLRDLFRWCQLMLVDQSPGCYDPGQHVFLVYGERMRTEEDKKKVIAVFKDVFGS
Splooce       MEFIASTLFPAIEKNIVKKMVAFNNQIDHEVTVEKKWGQKGGPWEFNLRDLFRWCQLMLVDQSPGCYDPGQHVFLVYGERMRTEEDKKKVIAVFKDVFGS

Uniprot       NSNPYMGTRLFRITPYDVQLGYSVLSRGSCVPHPSRHPLLLLHQSFQPLESIMKCVQMSWMVILVGPASVGKTSLVQLLAHLTGHTLKIMAMNSAMDTTE
Splooce       NSNPYMGTRLFRITPYDVQLGYSVLSRGSCVPHPSRHPLLLLHQSFQPLESIMKCVQMSWMVILVGPASVGKTSLVQLLAHLTGHTLKIMAMNSAMDTTE

Uniprot       LLGGFEQVDLIRPWRRLLEKVEGTVRALLRDSLLISADDAEVVLRAWSHFLLTYKPKCLGEGGKAITMEIVNKLEAVLLLMQRLNNKINSYCKAEFAKLV
Splooce       LLGGFEQVDLIRPWRRLLEKVEGTVRALLRDSLLISADDAEVVLRAWSHFLLTYKPKCLGEGGKAITMEIVNKLEAVLLLMQRLNNKINSYCKAEFAKLV

Uniprot       EEFRSFGVKLTQLASGHSHGTFEWVDSMLVQALKSGDWLLMDNVNFCNPSVLDRLNALLEPGGVLTISERGMIDGSTPTITPNPNFRLFLSMDPVHGDIS
Splooce       EEFRSFGVKLTQLASGHSHGTFEWVDSMLVQALKSGDWLLMDNVNFCNPSVLDRLNALLEPGGVLTISERGMIDGSTPTITPNPNFRLFLSMDPVHGDIS

Uniprot       RAMRNRGLEIYISGEGDASTPDNLDLKVLLHSLGLVGNSVCDILLALHTETRSTVVGSPTSSVSTLIQTAILIVQYLQRGLSLDRAFSEACWEVYVCSQH
Splooce       RAMRNRGLEIYISGEGDASTPDNLDLKVLLHSLGLVGNSVCDILLALHTETRSTVVGSPTSSVSTLIQTAILIVQYLQRGLSLDRAFSEACWEVYVCSQH

Uniprot       SPANRKLVQALLEKHVSSLRAHETWGDSILGMGLWPDSVPSALFATEDSHLSTVRRDGQILVYCLNRMSMKTSSWTRSQPFTLQDLEKIMQSPSPENLKF
Splooce       SPANRKLVQALLEKHVSSLRAHETWGDSILGMGLWPDSVPSALFATEDSHLSTVRRDGQILVYCLNRMSMKTSSWTRSQPFTLQDLEKIMQSPSPENLKF

Uniprot       NAVEVNTYWIDEPDVLVMAVKLLIERATNQDWMLRVKWLYHLAKNIPQGLESIQIHLEASAASLRNFYSHSLSGAVSNVFKILQPNTTDEFVIPLDPRWN
Splooce       NAVEVNTYWIDEPDVLVMAVKLLIERATNQDWMLRVKWLYHLAKNIPQGLESIQIHLEASAASLRNFYSHSLSGAVSNVFKILQPNTTDEFVIPLDPRWN

Uniprot       MQALDMIRNLMDFDPQTDQPDQLFALLESAANKTIIYLDREKRVFTEANLVSVGSKKLRESVLRMSFEFHQDPESYHTLPHEIVVNLAAFFELCDALVLL
Splooce       MQALDMIRNLMDFDPQTDQPDQLFALLESAANKTIIYLDREKRVFTEANLVSVGSKKLRESVLRMSFEFHQDPESYHTLPHEIVVNLAAFFELCDALVLL

Uniprot       WVQSSQGMVSDASANEILGSLRWRDRFWTVADTVKVDAPGLALLALHWHWVLKHLVHQIPRLLMNYEDKYYKEVQTVSEHIQNCLGSQTGGFAGIKKLQK
Splooce       WVQSSQGMVSDASANEILGSLRWRDRFWTVADTVKVDAPGLALLALHWHWVLKHLVHQIPRLLMNYEDKYYKEVQTVSEHIQNCLGSQTGGFAGIKKLQK

Uniprot       FLGRPFPFKDKLVVECFSQLKVLNKVLAIREQMSALGESGWQEDINRLQVVASQWTLKKSLLQAWGLILRANILEDVSLDELKNFVHAQCLELKAKGLSL
Splooce       FLGRPFPFKDKLVVECFSQLKVLNKVLAIREQMSALGESGWQEDINRLQVVASQWTLKKSLLQAWGLILRANILEDVSLDELKNFVHAQCLELKAKGLSL

Uniprot       GFLEKKHDEASSLSHPDLTSVIHLTRSVQLWPAMEYLAMLWRYKVTADFMAQACLRRCSKNQQPQINEEISHLISFCLYHTPVTPQELRDLWSLLHHQKV
Splooce       GFLEKKHDEASSLSHPDLTSVIHLTRSVQLWPAMEYLAMLWRYKVTADFMAQACLRRCSKNQQPQINEEISHLISFCLYHTPVTPQELRDLWSLLHHQKV

Uniprot       SPEEITSLWSELFNSMFMSFWSSTVTTNPEYWLMWNPLPGMQQREAPKSVLDSTLKGPGNLNRPIFSKCCFEVLTSSWRASPWDVSGLPILSSSHVTLGE
Splooce       SPEEITSLWSELFNSMFMSFWSSTVTTNPEYWLMWNPLPGMQQREAPKSVLDSTLKGPGNLNRPIFSKCCFEVLTSSWRASPWDVSGLPILSSSHVTLGE

Uniprot       WVERTQQLQDISSMLWTNMAISSVAEFRRTDSQLQGQVLFRHLAGLAELLPESRRQEYMQNCEQLLLGSSQAFQHVGQTLGDMAGQEVLPKELLCQLLTS
Splooce       WVERTQQLQDISSMLWTNMAISSVAEFRRTDSQLQGQVLFRHLAGLAELLPESRRQEYMQNCEQLLLGSSQAFQHVGQTLGDMAGQEVLPKELLCQLLTS

Uniprot       LHHFVGEGESKRSLPEPAQRGSLWVSLGLLQIQTWLPQARFDPAVKREYKLNYVKEELHQLQCEWKTRNLSSQLQTGRDLEDEVVVSYSHPHVRLLRQRM
Splooce       LHHFVGEGESKRSLPEPAQRGSLWVSLGLLQIQTWLPQARFDPAVKREYKLNYVKEELHQLQCEWKTRNLSSQLQTGRDLEDEVVVSYSHPHVRLLRQRM

Uniprot       DRLDNLTCHLLKKQAFRPQLPAYESLVQEIHHYVTSIAKAPAVQDLLTRLLQALHIDGPRSAQVAQSLLKEEASWQQSHHQFRKRLSEEYTFYPDAVSPL
Splooce       DRLDNLTCHLLKKQAFRPQLPAYESLVQEIHHYVTSIAKAPAVQDLLTRLLQALHIDGPRSAQVAQSLLKEEASWQQSHHQFRKRLSEEYTFYPDAVSPL

Uniprot       QASILQLQHGMRLVASELHTSLHSSMVGADRLGTLATALLAFPSVGPTFPTYYAHADTLCSVKSEEVLRGLGKLILKRSGGKELEGKGQKACPTREQLLM
Splooce       QASILQLQHGMRLVASELHTSLHSSMVGADRLGTLATALLAFPSVGPTFPTYYAHADTLCSVKSEEVLRGLGKLILKRSGGKELEGKGQKACPTREQLLM

Uniprot       NALLYLRSHVLCKGELDQRALQLFRHVCQEIISEWDEQERIAQEKAEQESGLYRYRSRNSRTALSEEEEEEREFRKQFPLHEKDFADILVQPTLEENKGT
Splooce       NALLYLRSHVLCKGELDQRALQLFRHVCQEIISEWDEQERIAQEKAEQESGLYRYRSRNSRTALSEEEEEEREFRKQFPLHEKDFADILVQPTLEENKGT

Uniprot       SDGQEEEAGTNPALLSQNSMQAVMLIHQQLCLNFARSLWYQQTLPPHEAKHYLSLFLSCYQTGASLVTHFYPLMGVELNDRLLGSQLLACTLSHNTLFGE
Splooce       SDGQEEEAGTNPALLSQNSMQAVMLIHQQLCLNFARSLWYQQTLPPHEAKHYLSLFLSCYQTGASLVTHFYPLMGVELNDRLLGSQLLACTLSHNTLFGE

Uniprot       APSDLMVKPDGPYDFYQHPNVPEARQCQPVLQGFSEAVSHLLQDWPEHPALEQLLVVMDRIRSFPLSSPISKFLNGLEILLAKAQDWEENASRALSLRKH
Splooce       APSDLMVKPDGPYDFYQHPNVPEARQCQPVLQGFSEAVSHLLQDWPEHPALEQLLVVMDRIRSFPLSSPISKFLNGLEILLAKAQDWEENASRALSLRKH

Uniprot       LDLISQMIIRWRKLELNCWSMSLDNTMKRHTEKSTKHWFSIYQMLEKHMQEQTEEQEDDKQMTLMLLVSTLQAFIEGSSLGEFHVRLQMLLVFHCHVLLM
Splooce       LDLISQMIIRWRKLELNCWSMSLDNTMKRHTEKSTKHWFSIYQMLEKHMQEQTEEQEDDKQMTLMLLVSTLQAFIEGSSLGEFHVRLQMLLVFHCHVLLM

Uniprot       PQVEGKDSLCSVLWNLYHYYKQFFDRVQAKIVELRSPLEKELKEFVKISKWNDVSFWSIKQSVEKTHRTLFKFMKKFEAVLSEPCRSSLVESDKEEQPDF
Splooce       PQVEGKDSLCSVLWNLYHYYKQFFDRVQAKIVELRSPLEKELKEFVKISKWNDVSFWSIKQSVEKTHRTLFKFMKKFEAVLSEPCRSSLVESDKEEQPDF

Uniprot       LPRPTDGAASELSSIQNLNRALRETLLAQPAAGQATIPEWCQGAAPSGLEGELLRRLPKLRKRMRKMCLTFMKESPLPRLVEGLDQFTGEVISSVSELQS
Splooce       LPRPTDGAASELSSIQNLNRALRETLLAQPAAGQATIPEWCQGAAPSGLEGELLRRLPKLRKRMRKMCLTFMKESPLPRLVEGLDQFTGEVISSVSELQS

Uniprot       LKVEPSAEKEKQRSEAKHILMQKQRALSDLFKHLAKIGLSYRKGLAWARSKNPQEMLHLHPLDLQSALSIVSSTQEADSRLLTEISSSWDGCQKYFYRSL
Splooce       LKVEPSAEKEKQRSEAKHILMQKQRALSDLFKHLAKIGLSYRKGLAWARSKNPQEMLHLHPLDLQSALSIVSSTQEADSRLLTEISSSWDGCQKYFYRSL

Uniprot       ARHARLNAALATPAKEMGMGNVERCRGFSAHLMKMLVRQRRSLTTLSEQWIILRNLLSCVQEIHSRLMGPQAYPVAFPPQDGVQQWTERLQHLAMQCQIL
Splooce       ARHARLNAALATPAKEMGMGNVERCRGFSAHLMKMLVRQRRSLTTLSEQWIILRNLLSCVQEIHSRLMGPQAYPVAFPPQDGVQQWTERLQHLAMQCQIL

Uniprot       LEQLSWLLQCCPSVGPAPGHGNVQVLGQPPGPCLEGPELSKGQLCGVVLDLIPSNLSYPSPIPGSQLPSGCRMRKQDHLWQQSTTRLTEMLKTIKTVKAD
Splooce       LEQLSWLLQCCPSVGPAPGHGNVQVLGQPPGPCLEGPELSKGQLCGVVLDLIPSNLSYPSPIPGSQLPSGCRMRKQDHLWQQSTTRLTEMLKTIKTVKAD

Uniprot       VDKIRQQSCETLFHSWKDFEVCSSALSCLSQVSVHLQGLESLFILPGMEVEQRDSQMALVESLEYVRGEISKAMADFTTWKTHLLTSDSQGGNQMLDEGF
Splooce       VDKIRQQSCETLFHSWKDFEVCSSALSCLSQVSVHLQGLESLFILPGMEVEQRDSQMALVESLEYVRGEISKAMADFTTWKTHLLTSDSQGGNQMLDEGF

Uniprot       VEDFSEQMEIAIRAILCAIQNLEERKNEKAEENTDQASPQEDYAGFERLQSGHLTKLLEDDFWADVSTLHVQKIISAISELLERLKSYGEDGTAAKHLFF
Splooce       VEDFSEQMEIAIRAILCAIQNLEERKNEKAEENTDQASPQEDYAGFERLQSGHLTKLLEDDFWADVSTLHVQKIISAISELLERLKSYGEDGTAAKHLFF

Uniprot       SQSCSLLVRLVPVLSSYSDLVLFFLTMSLATHRSTAKLLSVLAQVFTELAQKGFCLPKEFMEDSAGEGATEFHDYEGGGIGEGEGMKDVSDQIGNEEQVE
Splooce       SQSCSLLVRLVPVLSSYSDLVLFFLTMSLATHRSTAKLLSVLAQVFTELAQKGFCLPKEFMEDSAGEGATEFHDYEGGGIGEGEGMKDVSDQIGNEEQVE

Uniprot       DTFQKGQEKDKEDPDSKSDIKGEDNAIEMSEDFDGKMHDGELEEQEEDDEKSDSEGGDLDKHMGDLNGEEADKLDERLWGDDDEEEDEEEEDNKTEETGP
Splooce       DTFQKGQEKDKEDPDSKSDIKGEDNAIEMSEDFDGKMHDGELEEQEEDDEKSDSEGGDLDKHMGDLNGEEADKLDERLWGDDDEEEDEEEEDNKTEETGP

Uniprot       GMDEEDSELVAKDDNLDSGNSNKDKSQQDKKEEKEEAEADDGGQGEDKINEQIDERDYDENEVDPYHGNQEKVPEPEALDLPDDLNLDSEDKNGGEDTDN
Splooce       GMDEEDSELVAKDDNLDSGNSNKDKSQQDKKEEKEEAEADDGGQGEDKINEQIDERDYDENEVDPYHGNQEKVPEPEALDLPDDLNLDSEDKNGGEDTDN

Uniprot       EEGEEENPLEIKEKPEEAGHEAEERGETETDQNESQSPQEPEEGPSEDDKAEGEEEMDTGADDQDGDAAQHPEEHSEEQQQSVEEKDKEADEEGGENGPA
Splooce       EEGEEENPLEIKEKPEEAGHEAEERGETETDQNESQSPQEPEEGPSEDDKAEGEEEMDTGADDQDGDAAQHPEEHSEEQQQSVEEKDKEADEEGGENGPA

Uniprot       DQGFQPQEEEEREDSDTEEQVPEALERKEHASCGQTGVENMQNTQAMELAGAAPEKEQGKEEHGSGAADANQAEGHESNFIAQLASQKHTRKNTQSFKRK
Splooce       DQGFQPQEEEEREDSDTEEQVPEALERKEHASCGQTGVENMQNTQAMELAGAAPEKEQGKEEHGSGAADANQAEGHESNFIAQLASQKHTRKNTQSFKRK

Uniprot       PGQADNERSMGDHNERVHKRLRTVDTDSHAEQGPAQQPQAQVEDADAFEHIKQGSDAYDAQTYDVASKEQQQSAKDSGKDQEEEEIEDTLMDTEEQEEFK
Splooce       PGQADNERSMGDHNERVHKRLRTVDTDSHAEQGPAQQPQAQVEDADAFEHIKQGSDAYDAQTYDVASKEQQQSAKDSGKDQEEEEIEDTLMDTEEQEEFK

Uniprot       AADVEQLKPEEIKSGTTAPLGFDEMEVEIQTVKTEEDQDPRTDKAHKETENEKPERSRESTIHTAHQFLMDTIFQPFLKDVNELRQELERQLEMWQPRES
Splooce       AADVEQLKPEEIKSGTTAPLGFDEMEVEIQTVKTEEDQDPRTDKAHKETENEKPERSRESTIHTAHQFLMDTIFQ-------------------------

Uniprot       GNPEEEKVAAEMWQSYLILTAPLSQRLCEELRLILEPTQAAKLKGDYRTGKRLNIRKVIPYIASQFRKDKIWLRRTKPSKRQYQICLAIDDSSSMVDNHT
Splooce       ---------------------------------------------------------------------------------FWRICKAVTPIS-------

Uniprot       KQLAFESLAVIGNALTLLEVGQIAVCSFGESVKLLHPFHEQFSDYSGSQILRLCKFQQKKTKIAQFLESVANMFAAAQQLSQNISSETAQLLLVVSDGRG
Splooce       ----------------------------------------------------------------------------------------------------

Uniprot       LFLEGKERVLAAVQAARNANIFVIFVVLDNPSSRDSILDIKVPIFKGPGEMPEIRSYMEEFPFPYYIILRDVNALPETLSDALRQWFELVTASDHP
Splooce       ------------------------------------------------------------------------------------------------

----------------------------------------------------------------------------------------------------

Q5T2N8 (Uniprot)	versus
NM_001039211#(-s-s-:1_A2180075896246) (Splooce)

For more details about the Alternative Splicing Event -> Link to Splooce page

Peptides that support the ASE (Splooce-specific):
GFMLILASCHPEQFDWAINACIDVMVHFDLPGQEER (MAXQUANT)

Alignment:
Uniprot       MSKDALNLAQMQEQTLQLEQQSKLKQLVNEDLRKQEESVQKHHQTFLESIRAAGTLFGEGFRAFVTDRDKVTATVAGLTLLAVGVYSAKNATAVTGRYIE
Splooce       MSKDALNLAQMQEQTLQLEQQSKLKQLVNEDLRKQEESVQKHHQTFLESIRAAGTLFGEGFRAFVTDRDKVTATVAGLTLLAVGVYSAKNATAVTGRYIE

Uniprot       ARLGKPSLVRETSRITVLEALRHPIQQVSRRLLSRPQDVLEGVVLSPSLEARVRDIAIMTRNIKKNRGLYRHILLYGPPGTGKTLFAKKLALHSGMDYAI
Splooce       ARLGKPSLVRETSRITVLEALRHPIQQVSRRLLSRPQDVLEGVVLSPSLEARVRDIAIMTRNIKKNRGLYRHILLYGPPGTGKTLFAKKLALHSGMDYAI

Uniprot       MTGGDVAPMGREGVTAMHKLFDWANTSRRGLLLFVDEADAFLRKRATEKISEDLRATLNAFLYRTGQHSNKFMLILASCHPEQFDWAINACIDVMVHFDL
Splooce       MTGGDVAPMGREGVTAMHKLFDWANTSRRG-----------------------------------------FMLILASCHPEQFDWAINACIDVMVHFDL

Uniprot       PGQEERARLVRMYLNEYVLKPATEGKRRLKLAQFDYGRKCLEIARLTEGMSCRKIAQLAVSWQATAYASKDGVLTEAMMDACVQDFVQQHQQMMRWLKGE
Splooce       PGQEERARLVRMYLNEYVLKPATEGKRRLKLAQFDYGRKCLEIARLTEGMSCRKIAQLAVSWQATAYASKDGVLTEAMMDACVQDFVQQHQQMMRWLKGE

Uniprot       RPGPEDEQPSS
Splooce       RPGPEDEQPSS

----------------------------------------------------------------------------------------------------

Q75MJ1 (Uniprot)	versus
NM_005692#(-s-s-:7_A8742441041111) (Splooce)

For more details about the Alternative Splicing Event -> Link to Splooce page

Peptides that support the ASE (Splooce-specific):
LLTGEVSPIRNLSDGQK (MAXQUANT)

Alignment:
Uniprot       MPSDLAKKKAAKKKEAAKARQRPRKGHEENGDVVTEPQVAEKNEANGRETTEVDLLTKELEDFEMKKAAARAVTGVLASHPNSTDVHIINLSLTFHGQEL
Splooce       MPSDLAKKKAAKKKEAAKARQRPRKGHEENGDVVTEPQVAEKNEANGRETTEVDLLTKELEDFEMKKAAARAVTGVLASHPNSTDVHIINLSLTFHGQEL

Uniprot       LSDTKLELNSGRRYGLIGLNGIGKSMLLSAIGKREVPIPEHIDIYHLTREMPPSDKTPLHCVMEVDTERAMLEKEAERLAHEDAECEKLMELYERLEELD
Splooce       LSDTKLELNSGRRYGLIGLNGIGKSMLLSAIGKREVPIPEHIDIYHLTREMPPSDKTPLHCVMEVDTERAMLEKEAERLAHEDAECEKLMELYERLEELD

Uniprot       ADKAEMRASRILHGLGFTPAMQRKKLKDFSGGWRMRVALARALFIRPFMLLLDEPTNHLDLDACVWLEEELKTFKRILVLVSHSQDFLNGVCTNIIHMHN
Splooce       ADKAEMRASRILHGLGFTPAMQRKKLKDFSGGWRMRVALARALFIRPFMLLLDEPTNHLDLDACVWLEEELKTFKRILVLVSHSQDFLNGVCTNIIHMHN

Uniprot       KKLKYYTGNYDQYVKTRLELEENQMKRFHWEQDQIAHMKNYIARFGHGSAKLARQAQSKEKTLQKMMASGLTERVVSDKTLSFYFPPCGKIPPPVIMVQN
Splooce       KKLKYYTGNYDQYVKTRLELEENQMKRFHWEQDQIAHMKNYIARFGHGSAKLARQAQSKEKTLQKMMASGLTERVVSDKTLSFYFPPCGKIPPPVIMVQN

Uniprot       VSFKYTKDGPCIYNNLEFGIDLDTRVALVGPNGAGKSTLLKLLTGELLPTDGMIRKHSHVKIGRYHQHLQEQLDLDLSPLEYMMKCYPEIKEKEEMRKII
Splooce       VSFKYTKDGPCIYNNLEFGIDLDTRVALVGPNGAGKSTLLKLLTG-------------------------------------------------------

Uniprot       GRYGLTGKQQVSPIRNLSDGQKCRVCLAWLAWQNPHMLFLDEPTNHLDIETIDALADAINEFEGGMMLVSHDFRLIQQVAQEIWVCEKQTITKWPGDILA
Splooce       ---------EVSPIRNLSDGQKCRVCLAWLAWQNPHMLFLDEPTNHLDIETIDALADAINEFEGGMMLVSHDFRLIQQVAQEIWVCEKQTITKWPGDILA

Uniprot       YKEHLKSKLVDEEPQLTKRTHNVCTLTLASLPRP
Splooce       YKEHLKSKLVDEEPQLTKRTHNVCTLTLASLPRP

----------------------------------------------------------------------------------------------------

Q9UHX1 (Uniprot)	versus
NM_014281#(-s-s-s-s-:8_P9551264263148) (Splooce)

For more details about the Alternative Splicing Event -> Link to Splooce page

Peptides that support the ASE (Splooce-specific):
WKPPQVGR (MAXQUANT)

Alignment:
Uniprot       MATATIALQVNGQQGGGSEPAAAAAVVAAGDKWKPPQGTDSIKMENGQSTAAKLGLPPLTPEQQEALQKAKKYAMEQSIKSVLVKQTIAHQQQQLTNLQM
Splooce       MATATIALQVNGQQGGGSEPAAAAAVVAAGDKWKPPQ---------------------------------------------------------------

Uniprot       AAQRQRALAIMCRVYVGSIYYELGEDTIRQAFAPFGPIKSIDMSWDSVTMKHKGFAFVEYEVPEAAQLALEQMNSVMLGGRNIKVGRPSNIGQAQPIIDQ
Splooce       ------------------------------------------------------------------------------------VGRPSNIGQAQPIIDQ

Uniprot       LAEEARAFNRIYVASVHQDLSDDDIKSVFEAFGKIKSCTLARDPTTGKHKGYGFIEYEKAQSSQDAVSSMNLFDLGGQYLRVGKAVTPPMPLLTPATPGG
Splooce       LAEEARAFNRIYVASVHQDLSDDDIKSVFEAFGKIKSCTLARDPTTGKHKGYGFIEYEKAQSSQDAVSSMNLFDLGGQYLRVGKAVTPPMPLLTPATPGG

Uniprot       LPPAAAVAAAAATAKITAQEAVAGAAVLGTLGTPGLVSPALTLAQPLGTLPQAVMAAQAPGVITGVTPARPPIPVTIPSVGVVNPILASPPTLGLLEPKK
Splooce       LPPAAAVAAAAATAKITAQEAVAGAAVLGTLGTPGLVSPALTLAQPLGTLPQAVMAAQAPGVITGVTPARPPIPVTIPSVGVVNPILASPPTLGLLEPKK

Uniprot       EKEEEELFPESERPEMLSEQEHMSISGSSARHMVMQKLLRKQESTVMVLRNMVDPKDIDDDLEGEVTEECGKFGAVNRVIIYQEKQGEEEDAEIIVKIFV
Splooce       EKEEEELFPESERPEMLSEQEHMSISGSSARHMVMQKLLRKQESTVMVLRNMVDPKDIDDDLEGEVTEECGKFGAVNRVIIYQEKQGEEEDAEIIVKIFV

Uniprot       EFSIASETHKAIQALNGRWFAGRKVVAEVYDQERFDNSDLSA
Splooce       EFSIASETHKAIQALNGRWFAGRKVVAEVYDQERFDNSDLSA

----------------------------------------------------------------------------------------------------

P14618 (Uniprot)	versus
NM_182470#(-t:15_P5814384899664) (Splooce)

For more details about the Alternative Splicing Event -> Link to Splooce page

Peptides that support the ASE (Splooce-specific):
LLTPGACSSEVPSAVPSR (MAXQUANT)

Alignment:
Uniprot       MSKPHSEAGTAFIQTQQLHAAMADTFLEHMCRLDIDSPPITARNTGIICTIGPASRSVETLKEMIKSGMNVARLNFSHGTHEYHAETIKNVRTATESFAS
Splooce       ----------------------------------------------------------------------------------------------------

Uniprot       DPILYRPVAVALDTKGPEIRTGLIKGSGTAEVELKKGATLKITLDNAYMEKCDENILWLDYKNICKVVEVGSKIYVDDGLISLQVKQKGADFLVTEVENG
Splooce       ----------------------------------------------------------------------------------------------------

Uniprot       GSLGSKKGVNLPGAAVDLPAVSEKDIQDLKFGVEQDVDMVFASFIRKASDVHEVRKVLGEKGKNIKIISKIENHEGVRRFDEILEASDGIMVARGDLGIE
Splooce       ----------------------------------------------------------------------------------------------------

Uniprot       IPAEKVFLAQKMMIGRCNRAGKPVICATQMLESMIKKPRPTRAEGSDVANAVLDGADCIMLSGETAKGDYPLEAVRMQHLIAREAEAAMFHRKLFEELVR
Splooce       -------------------------------------------------------------------------------------------------MQW

Uniprot       ASSHSTDLMEAMAMGSVEASYKCLAAALIVLTESGRSAHQVARYRPRAPIIAVTRNPQTARQAHLYRGIFPVLCKDP=VQEAWAEDVDLRVNFAMNVGKA
Splooce       SSERGERLLTP---G------ACSSEVPSAVP---------SRSGPQQP----CRSPIVKPGLPSFR---PSSCTQPWLTHSWSTCAAWTL---IHHPSQ

Uniprot       RGFFKKGDVVIVLTG=WRPGSGFTNTMRVVPVP
Splooce       PGTLASSVPLAQLPDQWRR--------------

----------------------------------------------------------------------------------------------------

P23588 (Uniprot)	versus
NM_001417#(-t:12_E5959462724319) (Splooce)

For more details about the Alternative Splicing Event -> Link to Splooce page

Peptides that support the ASE (Splooce-specific):
PEENPASFSSASK (MAXQUANT)

Alignment:
Uniprot       MAASAKKKNKKGKTISLTDFLAEDGGTGGGSTYVSKPVSWADETDDLEGDVSTTWHSNDDDVYRAPPIDRSILPTAPRAAREPNIDRSRLPKSPPYTAFL
Splooce       MAASAKKKNKKGKTISLTDFLAEDGGTGGGSTYVSKPVSWADETDDLEGDVSTTWHSNDDDVYRAPPIDRSILPTAPRAAREPNIDRSRLPKSPPYTAFL

Uniprot       GNLPYDVTEESIKEFFRGLNISAVRLPREPSNPERLKGFGYAEFEDLDSLLSALSLNEESLGNRRIRVDVADQAQDKDRDDRSFGRDRNRDSDKTDTDWR
Splooce       GNLPYDVTEESIKEFFRGLNISAVRLPREPSNPERLKGFGYAEFEDLDSLLSALSLNEESLGNRRIRVDVADQAQDKDRDDRSFGRDRNRDSDKTDTDWR

Uniprot       ARPATDSFDDYPPRRGDDSFGDKYRDRYDSDRYRDGYRDGYRDGPRRDMDRYGGRDRYDDRGSRDYDRGYDSRIGSGRRAFGSGYRRDDDYRGGGDRYED
Splooce       ARPATDSFDDYPPRRGDDSFGDKYRDRYDSDRYRDGYRDGYRDGPRRDMDRYGGRDRYDDRGSRDYDRGYDSRIGSGRRAFGSGYRRDDDYRGGGDRYED

Uniprot       RYDRRDDRSWSSRDDYSRDDYRRDDRGPPQRPKLNLKPRSTPKEDDSSASTSQSTRAASIFGGAKPVDTAAREREVEERLQKEQEKLQRQLDEPKLERRP
Splooce       RYDRRDDRSWSSRDDYSRDDYRRDDRGPPQRPKLNLKPRSTPKEDDSSASTSQSTRAASIFGGAKPVDTAAREREVEERLQKEQEKLQRQLDEPKLERRP

Uniprot       RERHPSWRSEETQERERSRTGSESSQTGTSTTSSRNARRRESEKSLENETLNKEEDCHSPTSKPPKPDQPLKVMPAPPPKENAWVKRSSNPPARSQSSDT
Splooce       RERHPSWRSEETQERERSRTGSESSQTGTSTTSSRNARRRESEKSLENETLNKEEDCHSPTSKPPKPDQPLKVMPAPPPKENAWVKRSSNPPARSQSSDT

Uniprot       EQQSPTSGGGKVAPAQPSEEGPGRKDENKVDGMNAPKGQTGNSSRGPGDGGNRDHWKESDRKDGKKDQDSRSAPEPKKPEENPASKFSSASKYAALSVDG
Splooce       EQQSPTSGGGKVAPAQPSEEGPGRKDENKVDGMNAPKGQTGNSSRGPGDGGNRDHWKESDRKDGKKDQDSRSAPEPKKPEENPAS-FSSASKYAALSVDG

Uniprot       EDENEGEDYAE
Splooce       EDENEGEDYAE

----------------------------------------------------------------------------------------------------

Q2NL68 (Uniprot)	versus
NM_001039887#(-t:19_C9208851825572) (Splooce)

For more details about the Alternative Splicing Event -> Link to Splooce page

Peptides that support the ASE (Splooce-specific):
LFPDSLEDTPPHFEGPPPPK (MAXQUANT)

Alignment:
Uniprot       MDRSLPVFSIQDSPFGDAPLGRSHYWPSQSQTWCPKTLSPSRSQRSRLPQAPKALATGPNSPELFEESWPSSSGTPSLPSTTEGQMWASPAPTLIDSGDS
Splooce       MDRSLPVFSIQDSPFGDAPLGRSHYWPSQSQTWCPKTLSPSRSQRSRLPQAPKALATGPNSPELFEESWPSSSGTPSLPSTTEGQMWASPAPTLIDSGDS

Uniprot       VVAKYINRFRQAQPTSREERQPAGPTPADFWWLQSDSPDPSSQSAAAGANKPEGRPHTAVPTAVNVTSASHAVAPLQEIKQNLHTWNSSLLDLETLSLQS
Splooce       VVAKYINRFRQAQPTSREERQPAGPTPADFWWLQSDSPDPSSQSAAAGANKPEGRPHTAVPTAVNVTSASHAVAPLQEIKQNLHTWNSSLLDLETLSLQS

Uniprot       RAARLLKRSKASISSSSSLSPSDASTSSFPTSSDGLSPFSETFIPDSSKGLGPRAPASPAPAQAQTPTPAPAPASSQAPLRPEDDILYQWRQRRKLEQAQ
Splooce       RAARLLKRSKASISSSSSLSPSDASTSSFPTSSDGLSPFSETFIPDSSKGLGPRAPASPAPAQAQTPTPAPAPASSQAPLRPEDDILYQWRQRRKLEQAQ

Uniprot       GSKGDRAWVPPLTPALRTL---------------------------------------------------------------------------------
Splooce       GSKGDRAWVPPLTPALRTLTSPAPVETLSSLGTQPNHVPLWSSVAQPGPPEAFYVERPPFPSVSSPHIFWAPSSHGFFWAPQSGPWVSLGAVPPTQQAST

Uniprot       ----------------------------------------------------------------------------------------------------
Splooce       LAHLGSTLAPPASLASTLEPPASTPAPLASTLAPPASTPAPLACTPAPPASTLASPAVPQGLPIPDPSSCAQPKSLGPKSRRSRAPRPEAAEQVPAAGQG

Uniprot       ---------------------------------------------------------------AESLKAKALPPAAGSVIRKSEATPSPGACLQPEVPLS
Splooce       PGPQLRGVLGQVVAARLFPDSLEDTPPHFEGPPPPKAGSPKVQATQPQTKVTPPPSESQCRAKAESLKAKALPPAAGSVIRKSEATPSPGACLQPEVPLS

Uniprot       PAEQATTVKASPPAFQVGSPEALAPPPPAADHAPSEALLAQGRPAAAGCRRLRRQRVPGRSRAAGAKSP
Splooce       PAEQATTVKASPPAFQVGSPEALAPPPPAADHAPSEALLAQGRPAAAGCRRLRRQRVPGRSRAAGAKSP

----------------------------------------------------------------------------------------------------

P08865 (Uniprot)	versus
NM_002295#(-s-:3_R6006634327102) (Splooce)

For more details about the Alternative Splicing Event -> Link to Splooce page

Peptides that support the ASE (Splooce-specific):
LEKEEQAAAEK (PEAKS)
IEKEEQAAAEK (MAXQUANT)

Alignment:
Uniprot       MSGALDVLQMKEEDVLKFLAAGTHLGGTNLDFQMEQYIYKRKSDGIYIINLKRTWEKLLLAARAIVAIENPADVSVISSRNTGQRAVLKFAAATGATPIA
Splooce       MSGALDVLQMKEEDVLKFLAAGTHLGGTNLDFQMEQYIYKRKSDGIYIINLKRTWEKLLLAARAIVAIENPADVSVISSRNTGQRAVLKFAAATGATPIA

Uniprot       GRFTPGTFTNQIQAAFREPRLLVVTDPRADHQPLTEASYVNLPTIALCNTDSPLRYVDIAIPCNNKGAHSVGLMWWMLAREVLRMRGTISREHPWEVMPD
Splooce       GRFTPGTFTNQIQAAFREPRLLVVTDPRADHQPLTEASYVNLPTIALCNTDSPLRYVDIAIPCNNK----------------------------------

Uniprot       LYFYRDPEEIEKEEQAAAEKAVTKEEFQGEWTAPAPEFTATQPEVADWSEGVQVPSVPIQQFPTEDWSAQPATEDWSAAPTAQATEWVGATTDWS
Splooce       ---------IEKEEQAAAEKAVTKEEFQGEWTAPAPEFTATQPEVADWSEGVQVPSVPIQQFPTEDWSAQPATEDWSAAPTAQATEWVGATTDWS

----------------------------------------------------------------------------------------------------

Q9P2E9 (Uniprot)	versus
NM_004587#(-s-s-s-s-:20_R2171955532715) (Splooce)

For more details about the Alternative Splicing Event -> Link to Splooce page

Peptides that support the ASE (Splooce-specific):
QVLQLQDLR (MAXQUANT)

Alignment:
Uniprot       MDIYDTQTLGVVVFGGFMVVSAIGIFLVSTFSMKETSYEEALANQRKEMAKTHHQKVEKKKKEKTVEKKGKTKKKEEKPNGKIPDHDPAPNVTVLLREPV
Splooce       MDIYDTQTLGVVVFGGFMVVSAIGIFLVSTFSMKETSYEEALANQRKEMAKTHHQKVEKKKKEKTVEKKGKTKKKEEKPNGKIPDHDPAPNVTVLLREPV

Uniprot       RAPAVAVAPTPVQPPIIVAPVATVPAMPQEKLASSPKDKKKKEKKVAKVEPAVSSVVNSIQVLTSKAAILETAPKEGRNTDVAQSPEAPKQEAPAKKKSG
Splooce       RAPAVAVAPTPVQPPIIVAPVATVPAMPQEKLASSPKDKKKKEKKVAKVEPAVSSVVNSIQVLTSKAAILETAPKEGRNTDVAQSPEAPKQEAPAKKKSG

Uniprot       SKKKGPPDADGPLYLPYKTLVSTVGSMVFNEGEAQRLIEILSEKAGIIQDTWHKATQKGDPVAILKRQLEEKEKLLATEQEDAAVAKSKLRELNKEMAAE
Splooce       SKKKGPPDADGPLYLPYKTLVSTVGSMVFNEGEAQRLIEILSEKAGIIQDTWHKATQKGDPVAILKRQLEEKEKLLATEQEDAAVAKSKLRELNKEMAAE

Uniprot       KAKAAAGEAKVKKQLVAREQEITAVQARMQASYREHVKEVQQLQGKIRTLQEQLENGPNTQLARLQQENSILRDALNQATSQVESKQNAELAKLRQELSK
Splooce       KAKAAAGEAKVKKQLVAREQEITAVQARMQASYREHVKEVQQLQGKIRTLQEQLENGPNTQLARLQQENSILRDALNQATSQVESKQNAELAKLRQELSK

Uniprot       VSKELVEKSEAVRQDEQQRKALEAKAAAFEKQVLQLQASHRESEEALQKRLDEVSRELCHTQSSHASLRADAEKAQEQQQQMAELHSKLQSSEAEVRSKC
Splooce       VSKELVEKSEAVRQDEQQRKALEAKAAAFEKQVLQLQ---------------------------------------------------------------

Uniprot       EELSGLHGQLQEARAENSQLTERIRSIEALLEAGQARDAQDVQASQAEADQQQTRLKELESQVSGLEKEAIELREAVEQQKVKNNDLREKNWKAMEALAT
Splooce       -------------------------------------------------------------------------------------DLREKNWKAMEALAT

Uniprot       AEQACKEKLLSLTQAKEESEKQLCLIEAQTMEALLALLPELSVLAQQNYTEWLQDLKEKGPTLLKHPPAPAEPSSDLASKLREAEETQSTLQAECDQYRS
Splooce       AEQACKEKLLSLTQAKEESEKQLCLIEAQTMEALLALLPELSVLAQQNYTEWLQDLKEKGPTLLKHPPAPAEPSSDLASKLREAEETQSTLQAECDQYRS

Uniprot       ILAETEGMLRDLQKSVEEEEQVWRAKVGAAEEELQKSRVTVKHLEEIVEKLKGELESSDQVREHTSHLEAELEKHMAAASAECQNYAKEVAGLRQLLLES
Splooce       ILAETEGMLRDLQKSVEEEEQVWRAKVGAAEEELQKSRVTVKHLEEIVEKLKGELESSDQVREHTSHLEAELEKHMAAASAECQNYAKEVAGLRQLLLES

Uniprot       QSQLDAAKSEAQKQSDELALVRQQLSEMKSHVEDGDIAGAPASSPEAPPAEQDPVQLKTQLEWTEAILEDEQTQRQKLTAEFEEAQTSACRLQEELEKLR
Splooce       QSQLDAAKSEAQKQSDELALVRQQLSEMKSHVEDGDIAGAPASSPEAPPAEQDPVQLKTQLEWTEAILEDEQTQRQKLTAEFEEAQTSACRLQEELEKLR

Uniprot       TAGPLESSETEEASQLKERLEKEKKLTSDLGRAATRLQELLKTTQEQLAREKDTVKKLQEQLEKAEDGSSSKEGTSV
Splooce       TAGPLESSETEEASQLKERLEKEKKLTSDLGRAATRLQELLKTTQEQLAREKDTVKKLQEQLEKAEDGSSSKEGTSV

----------------------------------------------------------------------------------------------------

Q8NBF6 (Uniprot)	versus
NM_015060#(f-:7_K3071628663207) (Splooce)

For more details about the Alternative Splicing Event -> Link to Splooce page

Peptides that support the ASE (Splooce-specific):
LCVEEALIQIHDPELR (MAXQUANT)

Alignment:
Uniprot       MEKARRGGDGVPRGPVLHIVVVGFHHKKGCQVEFSYPPLIPGDGHDSHTLPEEWKYLPFLALPDGAHNYQEDTVFFHLPPRNGNGATVFGISCYRQIEAK
Splooce       MEKARRGGDGVPRGPVLHIVVVGFHHKKGCQVEFSYPPLIPGDGHDSHTLPEEWKYLPFLALPDGAHNYQEDTVFFHLPPRNGNGATVFGISCYRQIEAK

Uniprot       ALKVRQADITRETVQKSVCVLSKLPLYGLLQAKLQLITHAYFEEKDFSQISILKELYEHMNSSLGGASLEGSQVYLGLSPRDLVLHFRHKVLILFKLILL
Splooce       ALKVRQADITRETVQKSVCVLSKLPLYGLLQAKLQLITHAYFEEKDFSQISILKELYEHMNSSLGGASLEGSQVYLGLSPRDLVLHFRHKVLILFKLILL

Uniprot       EKKVLFYISPVNKLVGALMTVLSLFPGMIEHGLSDCSQYRPRKSMSEDGGLQESNPCADDFVSASTADVSHTNLGTIRKVMAGNHGEDAAMKTEEPLFQV
Splooce       EKKVLFYISPVNKLVGALMTVLSLFPGMIEHGLSDCSQYRPRKSMSEDGGLQESNPCADDFVSASTADVSHTNLGTIRKVMAGNHGEDAAMKTEEPLFQV

Uniprot       EDSSKGQEPNDTNQYLKPPSRPSPDSSESDWETLDPSVLEDPNLKEREQLGSDQTNLFPKDSVPSESLPITVQPQANTGQVVLIPGLISGLEEDQYGMPL
Splooce       EDSSKGQEPNDTNQYLKPPSRPSPDSSESDWETLDPSVLEDPNLKEREQLGSDQTNLFPKDSVPSESLPITVQPQANTGQVVLIPGLISGLEEDQYGMPL

Uniprot       AIFTKGYLCLPYMALQQHHLLSDVTVRGFVAGATNILFRQQKHLSDAIVEV====EEALIQIHDPELRKLLNPTTADLRFADYLVRHVTENRDDVFLDGT
Splooce       AIFTKGYLCLPYMALQQHHLLSDVTVRGFVAGATNILFRQQKHLSDAIVEVRLCVEEALIQIHDPELRKLLNPTTADLRFADYLVRHVTENRDDVFLDGT

Uniprot       GWEGGDEWIRAQFAVYIHALLAATLQLDNEKILSDYGTTFVTAWKNTHNYRVWNSNKHPALAEINPNHPFQGQYSVSDMKLRFSHSVQNSERGKKIGNVM
Splooce       GWEGGDEWIRAQFAVYIHALLAATLQLDNEKILSDYGTTFVTAWKNTHNYRVWNSNKHPALAEINPNHPFQGQYSVSDMKLRFSHSVQNSERGKKIGNVM

Uniprot       VTTSRNVVQTGKAVGQSVGGAFSSAKTAMSSWLSTFTTSTSQSLTEPPDEKP
Splooce       VTTSRNVVQTGKAVGQSVGGAFSSAKTAMSSWLSTFTTSTSQSLTEPPDEKP

----------------------------------------------------------------------------------------------------

P14209 (Uniprot)	versus
NM_002414#(f-:X_C648068284584) (Splooce)

For more details about the Alternative Splicing Event -> Link to Splooce page

Peptides that support the ASE (Splooce-specific):
SFSDADLADGVSGGEGK (MAXQUANT + PEAKS)

Alignment:
Uniprot       MARGAALALLLFGLLGVLVAAPDGGFDLSDALPDNENKKPTAIPKKPSAGDDFDLGDAVVDGENDDPRPPNPPKPMPNPNPNHPSSSGSFSDADLADGVS
Splooce       -------MLLLM-----------------EKMTTHDHR---THPNR---------------CQIQTPTTLVPPVRVSD---------RSFSDADLADGVS

Uniprot       GGEGKGGSDGGGSHRKEGEEADAPGVIPGIVGAVVVAVAGAISSFIAYQKKKLCFKENAEQGEVDMESHRNANAEPAVQRTLLEK
Splooce       GGEGKGGSDGGGSHRKEGEEADAPGVIPGIVGAVVVAVAGAISSFIAYQKKKLCFKENAEQGEVDMESHRNANAEPAVQRTLLEK

----------------------------------------------------------------------------------------------------

UNIPROT? (Uniprot)	versus
NM_001135056#(-s-s-:3_T1318804836967) (Splooce)

For more details about the Alternative Splicing Event -> Link to Splooce page

Peptides that support the ASE (Splooce-specific):
RPGVEDK (PEAKS)

Alignment:
Uniprot       -------
Splooce       ----------------------------------MLQRRRDHG---------------CPLFPHHANTSTR-----------PGVEDKESWHGKPLPKNM

Uniprot       -------
Splooce       AEQIIQEIYSQIQSKKKILATPPQEDAPSVDIANIRMPSLPSYKVGDKIATRKAYGQALAKLGHASDRIIALDGDTKNSTFSEIFKKEHPDRFIECYIAE

Uniprot       -------
Splooce       QNMVSIAVGCATRNRTVPFCSTFAAFFTRAFDQIRMAAISESNINLCGSHCGVSIGEDGPSQMALEDLAMFRSVPTSTVFYPSDGVATEKAVELAANTKG

Uniprot       -------
Splooce       ICFIRTSRPENAIIYNNNEDFQVGQAKVVLKSKDDQVTVIGAGVTLHEALAAAELLKKEKINIRVLDPFTIKPLDRKLILDSARATKGRILTVEDHYYEG

Uniprot       -------
Splooce       GIGEAVSSAVVGEPGITVTHLAVNRVPRSGKPAELLKMFGIDRDAIAQAVRGLITKA

----------------------------------------------------------------------------------------------------

P39023 (Uniprot)	versus
NM_000967#(-s-:22_R6196352163651) (Splooce)

For more details about the Alternative Splicing Event -> Link to Splooce page

Peptides that support the ASE (Splooce-specific):
GGFVHYGEVTNDFVMLK (MAXQUANT)

Alignment:
Uniprot       MSHRKFSAPRHGSLGFLPRKRSSRHRGKVKSFPKDDPSKPVHLTAFLGYKAGMTHIVREVDRPGSKVNKKEVVEAVTIVETPPMVVVGIVGYVETPRGLR
Splooce       MSHRKFSAPRHGSLGFLPRKRSSRHRGKVKSFPKDDPSKPVHLTAFLGYKAGMTHIVREVDRPGSKVNKKEVVEAVTIVETPPMVVVGIVGYVETPRGLR

Uniprot       TFKTVFAEHISDECKRRFYKNWHKSKKKAFTKYCKKWQDEDGKKQLEKDFSSMKKYCQVIRVIAHTQMRLLPLRQKKAHLMEIQVNGGTVAEKLDWARER
Splooce       TFKTVFAEHISDECKRRFYKNWHKSKKKAFTKYCKKWQDEDGKKQLEKDFSSMKKYCQVIRVIAHTQMRLLPLRQKKAHLMEIQVNGGTVAEKLDWARER

Uniprot       LEQQVPVNQVFGQDEMIDVIGVTKGKGYKGVTSRWHTKKLPRKTHRGLRKVACIGAWHPARVAFSVARAGQKGYHHRTEINKKIYKIGQGYLIKDGKLIK
Splooce       LEQQVPVNQVFGQDEMIDVIGVTKGKGYKGVTSRWHTKKLPRKTHRGLRKVACIGAWHPARVAFSVARAGQKGYHHRTEINKK-----------------

Uniprot       NNASTDYDLSDKSINPLGGFVHYGEVTNDFVMLKGCVVGTKKRVLTLRKSLLVQTKRRALEKIDLKFIDTTSKFGHGRFQTMEEKKAFMGPLKKDRIAKE
Splooce       -----------------GGFVHYGEVTNDFVMLKGCVVGTKKRVLTLRKSLLVQTKRRALEKIDLKFIDTTSKFGHGRFQTMEEKKAFMGPLKKDRIAKE

Uniprot       EGA
Splooce       EGA

----------------------------------------------------------------------------------------------------

P34897 (Uniprot)	versus
NM_005412#(-s-s-s-s-:12_S3621803936379) (Splooce)

For more details about the Alternative Splicing Event -> Link to Splooce page

Peptides that support the ASE (Splooce-specific):
LNVVLTTTWCWWTCGPR (MAXQUANT)

Alignment:
Uniprot       MLYFSLFWAARPLQRCGQLVRMAIRAQHSNAAQTQTGEANRGWTGQESLSDSDPEMWELLQREKDRQCRGLELIASENFCSRAALEALGSCLNNKYSEGY
Splooce       MLYFSLFWAARPLQRCGQLVRMAIRAQHSNAAQTQTGEANRGWTGQESLSDSDPEMWELLQREKDRQCRGLELIASENFCSRAALEALGSCLNNKYSEGY

Uniprot       PGKRYYGGAEVVDEIELLCQRRALEAFDLDPAQWGVNVQPYSGSPANLAVYTALLQPHDRIMGLDLPDGGHLTHGYMSDVKRISATSIFFESMPYKLNPK
Splooce       PGKRYYGGAEVVDEIELLCQRRALEAFDLDPAQWGVNVQPYSGSPANLAVYTALLQPHDRIMGLDLPDGGHLTHGYMSDVKRISATSIFFESMPYKLN--

Uniprot       TGLIDYNQLALTARLFRPRLIIAGTSAYARLIDYARMREVCDEVKAHLLADMAHISGLVAAKVIPSPFKHADIVTTTTHKTLRGARSGLIFYRKGVKAVD
Splooce       ------------------------------------------------------------------------VVLTTT----------------------

Uniprot       PKTGREIPYTFEDRINFAVFPSLQGGPHNHAIAAVAVALKQACTPMFREYSLQVLKNARAMADALLERGYSLVSGGTDNHLVLVDLRPKGLDGARAERVL
Splooce       -------------------WCWWTCGPR-----------------------------------AWMELGLSGC---------------------------

Uniprot       ELVSITANKNTCPGDRSAITPGGLRLGAPALTSRQFREDDFRRVVDFIDEGVNIGLEVKSKTAKLQDFKSFLLKDSETSQRLANLRQRVEQFARAFPMPG
Splooce       ----------------------------------------------------------------------------------------------------

Uniprot       FDEH
Splooce       ----

----------------------------------------------------------------------------------------------------

Q5JWU6 (Uniprot)	versus
NM_199360#(-s-s-s-:20_T7105587983731) (Splooce)

For more details about the Alternative Splicing Event -> Link to Splooce page

Peptides that support the ASE (Splooce-specific):
VTQSDLNSATFK (MAXQUANT)

Alignment:
Uniprot       MDSAGQDINLNSPNKGLLSDSMTDVPVDTGVAARTPAVEGLTEAEEEELRAELTKVEEEIVTLRQVLAAKERHCGELKRRLGLSTLGELKQNLSRSWHDV
Splooce       MDSAGQDINLNSPNKGLLSDSMTDVPVDTGVAARTPAVEGLTEAEEEELRAELTKVEEEIVTLRQVLAAKERHCGELKRRLGLSTLGELKQNLSRSWHDV

Uniprot       QVSSAYVKTSEKLGEWNEKVTQSDLYKKTQETLSQAGQKTSAALSTVGSAISRKLGDMRAHPFSHSFSSYSIRHSISMPAMRNSATFKSFEDRVGTIKSK
Splooce       QVSSAYVKTSEKLGEWNEKVTQSDL---------------------------------------------------------NSATFKSFEDRVGTIKSK

Uniprot       VVGDRENGSDNLPSSAGSGDKPLSDPAPF
Splooce       VVGDRENGSDNLPSSAGSGDKPLSDPAPF

----------------------------------------------------------------------------------------------------

Q14194 (Uniprot)	versus
NM_001313#(-s-s-s-s-s-:4_C5713386003492) (Splooce)

For more details about the Alternative Splicing Event -> Link to Splooce page

Peptides that support the ASE (Splooce-specific):
PEEVFGLQGVSR (MAXQUANT)

Alignment:
Uniprot       MSYQGKKSIPHITSDRLLIKGGRIINDDQSLYADVYLEDGLIKQIGENLIVPGGVKTIEANGRMVIPGGIDVNTYLQKPSQGMTAADDFFQGTRAALVGG
Splooce       MSYQGKKSIPHITSDRLLIKGGRIINDDQSLYADVYLEDGLIKQIGENLIVPGGVKTIEANGRMVIPGGIDVNTYLQKPSQGMTAADDFFQGTRAALVGG

Uniprot       TTMIIDHVVPEPGSSLLTSFEKWHEAADTKSCCDYSLHVDITSWYDGVREELEVLVQDKGVNSFQVYMAYKDVYQMSDSQLYEAFTFLKGLGAVILVHAE
Splooce       TTMIIDHVVPEPGSSLLTSFEKWHEAADTKSCCDYSLHVDITSWYDGVREELEVLVQDKGVNSFQVYMAYKDVYQMSDSQLYEAFTFLKGLGAVILVHAE

Uniprot       NGDLIAQEQKRILEMGITGPEGHALSRPEELEAEAVFRAITIAGRINCPVYITKVMSKSAADIIALARKKGPLVFGEPIAASLGTDGTHYWSKNWAKAAA
Splooce       NGDLIAQEQKRILEMGITGPEGHALSRPE-----------------------------------------------------------------------

Uniprot       FVTSPPLSPDPTTPDYLTSLLACGDLQVTGSGHCPYSTAQKAVGKDNFTLIPEGVNGIEERMTVVWDKAVATGKMDENQFVAVTSTNAAKIFNLYPRKGR
Splooce       ----------------------------------------------------------------------------------------------------

Uniprot       IAVGSDADVVIWDPDKLKTITAKSHKSAVEYNIFEGMECHGSPLVVISQGKIVFEDGNINVNKGMGRFIPRKAFPEHLYQRVKIRNKVFGLQGVSRGMYD
Splooce       --------------------------------------------------------------------------------------EVFGLQGVSRGMYD

Uniprot       GPVYEVPATPKYATPAPSAKSSPSKHQPPPIRNLHQSNFSLSGAQIDDNNPRRTGHRIVAPPGGRSNITSLG
Splooce       GPVYEVPATPKYATPAPSAKSSPSKHQPPPIRNLHQSNFSLSGAQIDDNNPRRTGHRIVAPPGGRSNITSLG

----------------------------------------------------------------------------------------------------

Q8TD30 (Uniprot)	versus
NM_133443#(-s-s-s-s-:16_G3611005557097) (Splooce)

For more details about the Alternative Splicing Event -> Link to Splooce page

Peptides that support the ASE (Splooce-specific):
CIEDVIHFAWEEKLFLLADEDDYPPSSGEAENGAAEGER (MAXQUANT)

Alignment:
Uniprot       MQRAAALVRRGCGPRTPSSWGRSQSSAAAEASAVLKVRPERSRRERILTLESMNPQVKAVEYAVRGPIVLKAGEIELELQRGIKKPFTEVIRANIGDAQA
Splooce       MQRAAALVRRGCGPRTPSSWGRSQSSAAAEASAVLKVRPERSRRERILTLESMNPQVKAVEYAVRGPIVLKAGEIELELQRGIKKPFTEVIRANIGDAQA

Uniprot       MGQQPITFLRQVMALCTYPNLLDSPSFPEDAKKRARRILQACGGNSLGSYSASQGVNCIREDVAAYITRRDGGVPADPDNIYLTTGASDGISTILKILVS
Splooce       MGQQPITFLRQVMALCTYPNLLDSPSFPEDAKKRARRILQACGGNSLGSYSASQGVNCIREDVAAYITRRDGGVPADPDNIYLTTGASDGISTILKILVS

Uniprot       GGGKSRTGVMIPIPQYPLYSAVISELDAIQVNYYLDEENCWALNVNELRRAVQEAKDHCDPKVLCIINPGNPTGQVQSRKCIEDVIHFAWEEKLFLLADE
Splooce       GGGKSRTGVMIPIPQYPLYSAVISELDAIQVNYYLDEENCWALNVNELRRAVQEAKDHCDPKVLCIINPGNPTGQVQSRKCIEDVIHFAWEEKLFLLADE

Uniprot       VYQDNVYSPDCRFHSFKKVLYEMGPEYSSNVELASFHSTSKGYMGECGYRGGYMEVINLHPEIKGQLVKLLSVRLCPPVSGQAAMDIVVNPPVAGEESFE
Splooce       ---DD------------------------------------------------------------------------------------YPPSSGE----

Uniprot       QFSREKESVLGNLAKKAKLTEDLFNQVPGIHCNPLQGAMYAFPRIFIPAKAVEAAQAHQMAPDMFYCMKLLEETGICVVPGSGFGQREGTYHFRMTILPP
Splooce       ---AENGAAEG---------ERLPHQLPG------------------EVRVRTPEPQRETCPWLFLPMP-----------------------VRLNSPPP

Uniprot       VEKLKTVLQKVKDFHINFLEKYA
Splooce       -----------------------

----------------------------------------------------------------------------------------------------

O00154 (Uniprot)	versus
NM_181864#(-s-s-:1_A7622310687063) (Splooce)

For more details about the Alternative Splicing Event -> Link to Splooce page

Peptides that support the ASE (Splooce-specific):
NGDIVQPVLNPEPNTVSYSQSSLIHLVGPSDCTLHGFVHGAR (MAXQUANT)

Alignment:
Uniprot       MKLLARALRLCEFGRQASSRRLVAGQGCVGPRRGCCAPVQVVGPRADLPPCGACITGRIMRPDDANVAGNVHGGTILKMIEEAGAIISTRHCNSQNGERC
Splooce       MKLLARALRLCEFGRQASSRRLVAGQGCVGPRRGCCAPVQVVGPRADLPPCGACITGRIMRPDDANVAGNVHGGTILKMIEEAGAIISTRHCNSQNGERC

Uniprot       VAALARVERTDFLSPMCIGEVAHVSAEITYTSKHSVEVQVNVMSENILTGAKKLTNKATLWYVPLSLKNVDKVLEVPPVVYSRQEQEEEGRKRYEAQKLE
Splooce       VAALARVERTDFLSPMCIGEVAHVSAEITYTSKHSVEVQVNVMSENILTGAKKLTNKATLWYVPLSLKNVDKVLEVPPVVYSRQEQEEEGRKRYEAQKLE

Uniprot       RMETKWRNGDIVQPVLNPEPNTVSYSQSSLIHLVGPSDCTLHGFVHGGVTMKLMDEVAGIVAARHCKTNIVTASVDAINFHDKIRKGCVITISGRMTFTS
Splooce       RMETKWRNGDIVQPVLNPEPNTVSYSQSSLIHLVGPSDCTLHGFVHG---------------AR-----------------DRGRE--------------

Uniprot       NKSMEIEVLVDADPVVDSSQKRYRAASAFFTYVSLSQEGRSLPVPQLVPETEDEKKRFEEGKGRYLQMKAKRQGHAEPQP
Splooce       ------EAL-----------------------------------------------------------------------

----------------------------------------------------------------------------------------------------

E7ETM7 (Uniprot)	versus
NM_001102399#(-s-s-s-s-s-s-:1_H948980978842) (Splooce)

For more details about the Alternative Splicing Event -> Link to Splooce page

Peptides that support the ASE (Splooce-specific):
YEDYYYHPPPR (MAXQUANT + PEAKS)

Alignment:
Uniprot       MKTYRQREKQGSKVQESTKGPDEAKIKALLERTGYTLDVTTGQRKYGGPPPDSVYSGVQPGIGTEVFVGKIPRDLYEDELVPLFEKAGPIWDLRLMMDPL
Splooce       ----------------------------------------------------------------------------------------------------

Uniprot       SGQNRGYAFITFCGKEAAQEAVKLCDSYEIRPGKHLGVCISVANNRLFVGSIPKNKTKENILEEFSKVTGLTEGLVDVILYHQPDDKKKNRGFCFLEYED
Splooce       ----------------------------------------------------------------------------------------------------

Uniprot       HKSAAQARRRLMSGKVKVWGNVVTVEWADPVEEPDPEVMAKVKVLFVRNLATTVTEEILEKSFSEFGKLERVKKLKDYAFVHFEDRGAAVKAMDEMNGKE
Splooce       ----------------------------------------------------------------------------------------------------

Uniprot       IEGEEIEIVLAKPPDKKRKERQAARQASRSTAYEDYYYHPPPRMPPPIRGRGRGGGRGGYGYPPDYYGYEDYYDDYYGYDYHDYRGGYEDPYYGYDDGYA
Splooce       ---------------MKRRSR-----------YEDYYYHPPPRMPPPIRGRGRGGGRGGYGYPPDYYGYEDYYDDYYGYDYHDYRGGYEDPYYGYDDGYA

Uniprot       VRGRGGGRGGRGAPPPPRGRGAPPPRGRAGYSQRGAPLGPPRGSRGGRGGPAQQQRGRGSRGSRGNRGGNVGGKRKADGYNQPDSKRRQTNNQQNWGSQP
Splooce       VRGRGGGRGGRGAPPPPRGRGAPPPRGRAGYSQRGAPLGPPRGSRGGRGGPAQQQRGRGSRGSRGNRGGNVGGKRKADGYNQPDSKRRQTNNQQNWGSQP

Uniprot       IAQQPLQQGGDYSGNYGYNNDNQEFYQDTYGQQWK
Splooce       IAQQPLQQGGDYSGNYGYNNDNQEFYQDTYGQQWK

----------------------------------------------------------------------------------------------------

Q9BQG0 (Uniprot)	versus
NM_014520#(-s-s-s-s-s-:17_M8800510072595) (Splooce)

For more details about the Alternative Splicing Event -> Link to Splooce page

Peptides that support the ASE (Splooce-specific):
AAAFQHLLLLVGIHLLKVLDLVEVLVTK (MAXQUANT)

Alignment:
Uniprot       MESRDPAQPMSPGEATQSGARPADRYGLLKHSREFLDFFWDIAKPEQETRLAATEKLLEYLRGRPKGSEMKYALKRLITGLGVGRETARPCYSLALAQLL
Splooce       MESRDPAQPMSPGEATQSGARPADRYGLLKHSREFLDFFWDIAKPEQETRLAATEKLLEYLRGRPKGSEMKYALKRLITGLGVGRETARPCYSLALAQLL

Uniprot       QSFEDLPLCSILQQIQEKYDLHQVKKAMLRPALFANLFGVLALFQSGRLVKDQEALMKSVKLLQALAQYQNHLQEQPRKALVDILSEVSKATLQEILPEV
Splooce       QSFEDLPLCSILQQIQEKYDLHQVKKAMLRPALFANLFGVLALFQSGRLVKDQEALMKSVKLLQALAQYQNHLQEQPRKALVDILSEVSKATLQEILPEV

Uniprot       LKADLNIILSSPEQLELFLLAQQKVPSKLKKLVGSVNLFSDENVPRLVNVLKMAASSVKKDRKLPAIALDLLRLALKEDKFPRFWKEVVEQGLLKMQFWP
Splooce       LKADLNIILSSPEQLELFLLAQQKVPSKLKKLVGSVNLFSDENVPRLVNVLKMAASSVKKDRKLPAIALDLLRLALKEDKFPRFWKEVVEQGLLKMQFWP

Uniprot       ASYLCFRLLGAALPLLTKEQLHLVMQGDVIRHYGEHVCTAKLPKQFKFAPEMDDYVGTFLEGCQDDPERQLAVLVAFSSVTNQGLPVTPTFWRVVRFLSP
Splooce       ASYLCFRLLGAALPLLTKEQLHLVMQGDVIRHYGEHVCTAKLPKQFKFAPEMDDYVGTFLEGCQDDPERQLAVLVAFSSVTNQGLPVTPTFWRVVRFLSP

Uniprot       PALQGYVAWLRAMFLQPDLDSLVDFSTNNQKKAQDSSLHMPERAVFRLRKWIIFRLVSIVDSLHLEMEEALTEQVARFCLFHSFFVTKKPTSQIPETKHP
Splooce       PALQGYVAWLRAMFLQPDLDSLVDFSTNNQKKAQDSSLHMPERAVFRLRKWIIFRLVSIVDSLHLEMEEALTEQVARFCLFHSFFVTKKPTSQIPETKHP

Uniprot       FSFPLENQAREAVSSAFFSLLQTLSTQFKQAPGQTQGGQPWTYHLVQFADLLLNHSHNVTTVTPFTAQQRQAWDRMLQTLKELEAHSAEARAAAFQHLLL
Splooce       FSFPLENQAREAVSSAFFSLLQTLSTQFKQAPGQTQGGQPWTYHLVQFADLLLNHSHNVTTVTPFTAQQRQAWDRMLQTLKELEAHSAEARAAAFQHLLL

Uniprot       LVGIHLLKSPAESCDLLGDIQTCIRKSLGEKPRRSRTKTIDPQEPPWVEVLVEILLALLAQPSHLMRQVARSVFGHICSHLTPRALQLILDVLNPETSED
Splooce       LVGIHLLK--------------------------------------------------------------------------------------------

Uniprot       ENDRVVVTDDSDERRLKGAEDKSEEGEDNRSSESEEESEGEESEEEERDGDVDQGFREQLMTVLQAGKALGGEDSENEEELGDEAMMALDQSLASLFAEQ
Splooce       ----------------------------------------------------------------------------------------------------

Uniprot       KLRIQARRDEKNKLQKEKALRRDFQIRVLDLVEVLVTKQPENALVLELLEPLLSIIRRSLRSSSSKQEQDLLHKTARIFTHHLCRARRYCHDLGERAGAL
Splooce       ---------------------------VLDLVEVLVTKQPENALVLELLEPLLSIIRRSLRSSSSKQEQDLLHKTARIFTHHLCRARRYCHDLGERAGAL

Uniprot       HAQVERLVQQAGRQPDSPTALYHFNASLYLLRVLKGNTAEGCVHETQEKQKAGTDPSHMPTGPQAASCLDLNLVTRVYSTALSSFLTKRNSPLTVPMFLS
Splooce       HAQVERLVQQAGRQPDSPTALYHFNASLYLLRVLKGNTAEGCVHETQEKQKAGTDPSHMPTGPQAASCLDLNLVTRVYSTALSSFLTKRNSPLTVPMFLS

Uniprot       LFSRHPVLCQSLLPILVQHITGPVRPRHQACLLLQKTLSMREVRSCFEDPEWKQLMGQVLAKVTENLRVLGEAQTKAQHQQALSSLELLNVLFRTCKHEK
Splooce       LFSRHPVLCQSLLPILVQHITGPVRPRHQACLLLQKTLSMREVRSCFEDPEWKQLMGQVLAKVTENLRVLGEAQTKAQHQQALSSLELLNVLFRTCKHEK

Uniprot       LTLDLTVLLGVLQGQQQSLQQGAHSTGSSRLHDLYWQAMKTLGVQRPKLEKKDAKEIPSATQSPISKKRKKKGFLPETKKRKKRKSEDGTPAEDGTPAAT
Splooce       LTLDLTVLLGVLQGQQQSLQQGAHSTGSSRLHDLYWQAMKTLGVQRPKLEKKDAKEIPSATQSPISKKRKKKGFLPETKKRKKRKSEDGTPAEDGTPAAT

Uniprot       GGSQPPSMGRKKRNRTKAKVPAQANGTPTTKSPAPGAPTRSPSTPAKSPKLQKKNQKPSQVNGAPGSPTEPAGQKQHQKALPKKGVLGKSPLSALARKKA
Splooce       GGSQPPSMGRKKRNRTKAKVPAQANGTPTTKSPAPGAPTRSPSTPAKSPKLQKKNQKPSQVNGAPGSPTEPAGQKQHQKALPKKGVLGKSPLSALARKKA

Uniprot       RLSLVIRSPSLLQSGAKKKAQVRKAGKP
Splooce       RLSLVIRSPSLLQSGAKKKAQVRKAGKP

----------------------------------------------------------------------------------------------------

G3XA88 (Uniprot)	versus
NM_001017957#(-s-s-s-s-s-s-s-s-:12_O5073045909378) (Splooce)

For more details about the Alternative Splicing Event -> Link to Splooce page

Peptides that support the ASE (Splooce-specific):
HIQQYHMEDR (MAXQUANT)

Alignment:
Uniprot       MAAETLLSSLLGLLLLGLLLPASLTGGVGSLNLEELSEMRYGIEILPLPVMGGQSQSSDVVIVSSKYKQRYECRLPAGAIHFQREREEETPAYQGPGIPE
Splooce       MAAETLLSSLLGLLLLGLLLPASLTGGVGSLNLEELSEMRYGIEILPLPVMGGQSQSSDVVIVSSKYKQRYECRLPAGAIHFQREREEETPAYQGPGIPE

Uniprot       LLSPMRDAPCLLKTKDWWTYEFCYGRHIQQYHMEDSEIKGEVLYLGYYQSAFDWDDETAKASKQHRLKRYHSQTYGNGSKCDLNGRPREAEVRFLCDEGA
Splooce       LLSPMRDAPCLLKTKDWWTYEFCYGRHIQQYHMEDRERAGP-----------RWAEEGVRA-------------------------------------GS

Uniprot       GISGDYIDRVDEPLSCSYVLTIRTPRLCPHPLLRPPPSAAPQAILCHPSLQPEEYMAYVQRQADSKQYGDKIIEELQDLGPQVWSETKSGVAPQKMAGAS
Splooce       GNAGSHIH--------------------------------------------------------------------------------SQQTHQKTGGKT

Uniprot       PTKDDSKDSDFWKMLNEPEDQAPGGEEVPAEEQDPSPEAADSASGAPNDFQNNVQVKVIRSPADLIRFIEELKGGTKKGKPNIGQEQPVDDAAEVPQREP
Splooce       ESR--------------------AGEEAQEKEGCP-----QKASPITPTYREN-----------------------------------------------

Uniprot       EKERGDPERQREMEEEEDEDEDEDEDEDERQLLGEFEKELEGILLPSDRDRLRSETEKELDPDGLKKESERDRAMLALTSTLNKLIKRLEEKQSPELVKK
Splooce       ----------------------------------------------------------------------------------------------------

Uniprot       HKKKRVVPKKPPPSPQPTGKIEIKIVRPWAEGTEEGARWLTDEDTRNLKEIFFNILVPGAEEAQKERQRQKELESNYRRVWGSPGGEGTGDLDEFDF
Splooce       -------------------------------------------------------------------------------------------------

----------------------------------------------------------------------------------------------------

Q0ZGT2 (Uniprot)	versus
NM_144573#(-s-s-s-:1_N6818505555342) (Splooce)

For more details about the Alternative Splicing Event -> Link to Splooce page

Peptides that support the ASE (Splooce-specific):
RAEQVNEDEENQDTAK (MAXQUANT)
AEQVNEDEENQDTAK (MAXQUANT)

Alignment:
Uniprot       MNDISQKAEILLSSSKPVPKTYVPKLGKGDVKDKFEAMQRAREERNQRRSRDEKQRRKEQYIREREWNRRKQEIKEMLASDDEEDVSSKVEKAYVPKLTG
Splooce       MNDISQKAEILLSSSKPVPKTYVPKLGKGDVKDKFEAMQRAREERNQRRSRDEKQRRKEQYIREREWNRRKQEIKEMLASDDEEDVSSKVEKAYVPKLTG

Uniprot       TVKGRFAEMEKQRQEEQRKRTEEERKRRIEQDMLEKRKIQRELAKRAEQIEDINNTGTESASEEGDDSLLITVVPVKSYKTSGKMKKNFEDLEKEREEKE
Splooce       TVKGRFAEMEKQRQEEQRKRTEEERKRRIEQDMLEKRKIQRELAKRAEQ---------------------------------------------------

Uniprot       RIKYEEDKRIRYEEQRPSLKEAKCLSLVMDDEIESEAKKESLSPGKLKLTFEELERQRQENRKKQAEEEARKRLEEEKRAFEEARRQMVNEDEENQDTAK
Splooce       ----------------------------------------------------------------------------------------VNEDEENQDTAK

Uniprot       IFKGYRPGKLKLSFEEMERQRREDEKRKAEEEARRRIEEEKKAFAEARRNMVVDDDSPEMYKTISQEFLTPGKLEINFEELLKQKMEEEKRRTEEERKHK
Splooce       IFKGYRPGKLKLSFEEMERQRREDEKRKAEEEARRRIEEEKKAFAEARRNMVVDDDSPEMYKTISQEFLTPGKLEINFEELLKQKMEEEKRRTEEERKHK

Uniprot       LEMEKQEFEQLRQEMGEEEEENETFGLSREYEELIKLKRSGSIQAKNLKSKFEKIGQLSEKEIQKKIEEERARRRAIDLEIKEREAENFHEEDDVDVRPA
Splooce       LEMEKQEFEQLRQEMGEEEEENETFGLSREYEELIKLKRSGSIQAKNLKSKFEKIGQLSEKEIQKKIEEERARRRAIDLEIKEREAENFHEEDDVDVRPA

Uniprot       RKSEAPFTHKVNMKARFEQMAKAREEEEQRRIEEQKLLRMQFEQREIDAALQKKREEEEEEEGSIMNGSTAEDEEQTRSGAPWFKKPLKNTSVVDSEPVR
Splooce       RKSEAPFTHKVNMKARFEQMAKAREEEEQRRIEEQKLLRMQFEQREIDAALQKKREEEEEEEGSIMNGSTAEDEEQTRSGAPWFKKPLKNTSVVDSEPVR

Uniprot       FTVKVTGEPKPEITWWFEGEILQDGEDYQYIERGETYCLYLPETFPEDGGEYMCKAVNNKGSAASTCILTIESKN
Splooce       FTVKVTGEPKPEITWWFEGEILQDGEDYQYIERGETYCLYLPETFPEDGGEYMCKAVNNKGSAASTCILTIESKN

----------------------------------------------------------------------------------------------------

P35579 (Uniprot)	versus
NM_002473#(-s-s-s-s-s-s-s-s-s-s-s-:22_M7996016236293) (Splooce)

For more details about the Alternative Splicing Event -> Link to Splooce page

Peptides that support the ASE (Splooce-specific):
EQELEEQLEEEESAR (MAXQUANT)

Alignment:
Uniprot       MAQQAADKYLYVDKNFINNPLAQADWAAKKLVWVPSDKSGFEPASLKEEVGEEAIVELVENGKKVKVNKDDIQKMNPPKFSKVEDMAELTCLNEASVLHN
Splooce       MAQQAADKYLYVDKNFINNPLAQADWAAKKLVWVPSDKSGFEPASLKEEVGEEAIVELVENGKKVKVNKDDIQKMNPPKFSKVEDMAELTCLNEASVLHN

Uniprot       LKERYYSGLIYTYSGLFCVVINPYKNLPIYSEEIVEMYKGKKRHEMPPHIYAITDTAYRSMMQDREDQSILCTGESGAGKTENTKKVIQYLAYVASSHKS
Splooce       LKERYYSGLIYTYSGLFCVVINPYKNLPIYSEEIVEMYKGKKRHEMPPHIYAITDTAYRSMMQDREDQSILCTGESGAGKTENTKKVIQYLAYVASSHKS

Uniprot       KKDQGELERQLLQANPILEAFGNAKTVKNDNSSRFGKFIRINFDVNGYIVGANIETYLLEKSRAIRQAKEERTFHIFYYLLSGAGEHLKTDLLLEPYNKY
Splooce       KKDQGELERQLLQANPILEAFGNAKTVKNDNSSRFGKFIRINFDVNGYIVGANIETYLLEKSRAIRQAKEERTFHIFYYLLSGAGEHLKTDLLLEPYNKY

Uniprot       RFLSNGHVTIPGQQDKDMFQETMEAMRIMGIPEEEQMGLLRVISGVLQLGNIVFKKERNTDQASMPDNTAAQKVSHLLGINVTDFTRGILTPRIKVGRDY
Splooce       RFLSNGHVTIPGQQDKDMFQETMEAMRIMGIPEEEQMGLLRVISGVLQLGNIVFKKERNTDQASMPDNTAAQKVSHLLGINVTDFTRGILTPRIKVGRDY

Uniprot       VQKAQTKEQADFAIEALAKATYERMFRWLVLRINKALDKTKRQGASFIGILDIAGFEIFDLNSFEQLCINYTNEKLQQLFNHTMFILEQEEYQREGIEWN
Splooce       VQKAQTKE--------------------------------------------------------------------------------------------

Uniprot       FIDFGLDLQPCIDLIEKPAGPPGILALLDEECWFPKATDKSFVEKVMQEQGTHPKFQKPKQLKDKADFCIIHYAGKVDYKADEWLMKNMDPLNDNIATLL
Splooce       ----------------------------------------------------------------------------------------------------

Uniprot       HQSSDKFVSELWKDVDRIIGLDQVAGMSETALPGAFKTRKGMFRTVGQLYKEQLAKLMATLRNTNPNFVRCIIPNHEKKAGKLDPHLVLDQLRCNGVLEG
Splooce       ----------------------------------------------------------------------------------------------------

Uniprot       IRICRQGFPNRVVFQEFRQRYEILTPNSIPKGFMDGKQACVLMIKALELDSNLYRIGQSKVFFRAGVLAHLEEERDLKITDVIIGFQACCRGYLARKAFA
Splooce       ----------------------------------------------------------------------------------------------------

Uniprot       KRQQQLTAMKVLQRNCAAYLKLRNWQWWRLFTKVKPLLQVSRQEEEMMAKEEELVKVREKQLAAENRLTEMETLQSQLMAEKLQLQEQLQAETELCAEAE
Splooce       ----------------------------------------------------------------------------------------------------

Uniprot       ELRARLTAKKQELEEICHDLEARVEEEEERCQHLQAEKKKMQQNIQELEEQLEEEESARQKLQLEKVTTEAKLKKLEEEQIILEDQNCKLAKEKKLLEDR
Splooce       ---------------------------------------------QELEEQLEEEESARQKLQLEKVTTEAKLKKLEEEQIILEDQNCKLAKEKKLLEDR

Uniprot       IAEFTTNLTEEEEKSKSLAKLKNKHEAMITDLEERLRREEKQRQELEKTRRKLEGDSTDLSDQIAELQAQIAELKMQLAKKEEELQAALARVEEEAAQKN
Splooce       IAEFTTNLTEEEEKSKSLAKLKNKHEAMITDLEERLRREEKQRQELEKTRRKLEGDSTDLSDQIAELQAQIAELKMQLAKKEEELQAALARVEEEAAQKN

Uniprot       MALKKIRELESQISELQEDLESERASRNKAEKQKRDLGEELEALKTELEDTLDSTAAQQELRSKREQEVNILKKTLEEEAKTHEAQIQEMRQKHSQAVEE
Splooce       MALKKIRELESQISELQEDLESERASRNKAEKQKRDLGEELEALKTELEDTLDSTAAQQELRSKREQEVNILKKTLEEEAKTHEAQIQEMRQKHSQAVEE

Uniprot       LAEQLEQTKRVKANLEKAKQTLENERGELANEVKVLLQGKGDSEHKRKKVEAQLQELQVKFNEGERVRTELADKVTKLQVELDNVTGLLSQSDSKSSKLT
Splooce       LAEQLEQTKRVKANLEKAKQTLENERGELANEVKVLLQGKGDSEHKRKKVEAQLQELQVKFNEGERVRTELADKVTKLQVELDNVTGLLSQSDSKSSKLT

Uniprot       KDFSALESQLQDTQELLQEENRQKLSLSTKLKQVEDEKNSFREQLEEEEEAKHNLEKQIATLHAQVADMKKKMEDSVGCLETAEEVKRKLQKDLEGLSQR
Splooce       KDFSALESQLQDTQELLQEENRQKLSLSTKLKQVEDEKNSFREQLEEEEEAKHNLEKQIATLHAQVADMKKKMEDSVGCLETAEEVKRKLQKDLEGLSQR

Uniprot       HEEKVAAYDKLEKTKTRLQQELDDLLVDLDHQRQSACNLEKKQKKFDQLLAEEKTISAKYAEERDRAEAEAREKETKALSLARALEEAMEQKAELERLNK
Splooce       HEEKVAAYDKLEKTKTRLQQELDDLLVDLDHQRQSACNLEKKQKKFDQLLAEEKTISAKYAEERDRAEAEAREKETKALSLARALEEAMEQKAELERLNK

Uniprot       QFRTEMEDLMSSKDDVGKSVHELEKSKRALEQQVEEMKTQLEELEDELQATEDAKLRLEVNLQAMKAQFERDLQGRDEQSEEKKKQLVRQVREMEAELED
Splooce       QFRTEMEDLMSSKDDVGKSVHELEKSKRALEQQVEEMKTQLEELEDELQATEDAKLRLEVNLQAMKAQFERDLQGRDEQSEEKKKQLVRQVREMEAELED

Uniprot       ERKQRSMAVAARKKLEMDLKDLEAHIDSANKNRDEAIKQLRKLQAQMKDCMRELDDTRASREEILAQAKENEKKLKSMEAEMIQLQEELAAAERAKRQAQ
Splooce       ERKQRSMAVAARKKLEMDLKDLEAHIDSANKNRDEAIKQLRKLQAQMKDCMRELDDTRASREEILAQAKENEKKLKSMEAEMIQLQEELAAAERAKRQAQ

Uniprot       QERDELADEIANSSGKGALALEEKRRLEARIAQLEEELEEEQGNTELINDRLKKANLQIDQINTDLNLERSHAQKNENARQQLERQNKELKVKLQEMEGT
Splooce       QERDELADEIANSSGKGALALEEKRRLEARIAQLEEELEEEQGNTELINDRLKKANLQIDQINTDLNLERSHAQKNENARQQLERQNKELKVKLQEMEGT

Uniprot       VKSKYKASITALEAKIAQLEEQLDNETKERQAACKQVRRTEKKLKDVLLQVDDERRNAEQYKDQADKASTRLKQLKRQLEEAEEEAQRANASRRKLQREL
Splooce       VKSKYKASITALEAKIAQLEEQLDNETKERQAACKQVRRTEKKLKDVLLQVDDERRNAEQYKDQADKASTRLKQLKRQLEEAEEEAQRANASRRKLQREL

Uniprot       EDATETADAMNREVSSLKNKLRRGDLPFVVPRRMARKGAGDGSDEEVDGKADGAEAKPAE
Splooce       EDATETADAMNREVSSLKNKLRRGDLPFVVPRRMARKGAGDGSDEEVDGKADGAEAKPAE

----------------------------------------------------------------------------------------------------

Q96TA1 (Uniprot)	versus
NM_022833#(-s-:9_F8683753176316) (Splooce)

For more details about the Alternative Splicing Event -> Link to Splooce page

Peptides that support the ASE (Splooce-specific):
QHIAGQR (MAXQUANT)

Alignment:
Uniprot       MGDVLSTHLDDARRQHIA------------------------------EKTGKILTEFLQFYEDQYGVALFNSMRHEIEGTGLPQAQLLWRKVPLDERIV
Splooce       MGDVLSTHLDDARRQHIAGQRSTSLWPWTPGVFSEFLEDGGMGLWFQQEKTGKILTEFLQFYEDQYGVALFNSMRHEIEGTGLPQAQLLWRKVPLDERIV

Uniprot       FSGNLFQHQEDSKKWRNRFSLVPHNYGLVLYENKAAYERQVPPRAVINSAGYKILTSVDQYLELIGNSLPGTTAKSGSAPILKCPTQFPLILWHPYARHY
Splooce       FSGNLFQHQEDSKKWRNRFSLVPHNYGLVLYENKAAYERQVPPRAVINSAGYKILTSVDQYLELIGNSLPGTTAKSGSAPILKCPTQFPLILWHPYARHY

Uniprot       YFCMMTEAEQDKWQAVLQDCIRHCNNGIPEDSKVEGPAFTDAIRMYRQSKELYGTWEMLCGNEVQILSNLVMEELGPELKAELGPRLKGKPQERQRQWIQ
Splooce       YFCMMTEAEQDKWQAVLQDCIRHCNNGIPEDSKVEGPAFTDAIRMYRQSKELYGTWEMLCGNEVQILSNLVMEELGPELKAELGPRLKGKPQERQRQWIQ

Uniprot       ISDAVYHMVYEQAKARFEEVLSKVQQVQPAMQAVIRTDMDQIITSKEHLASKIRAFILPKAEVCVRNHVQPYIPSILEALMVPTSQGFTEVRDVFFKEVT
Splooce       ISDAVYHMVYEQAKARFEEVLSKVQQVQPAMQAVIRTDMDQIITSKEHLASKIRAFILPKAEVCVRNHVQPYIPSILEALMVPTSQGFTEVRDVFFKEVT

Uniprot       DMNLNVINEGGIDKLGEYMEKLSRLAYHPLKMQSCYEKMESLRLDGLQQRFDVSSTSVFKQRAQIHMREQMDNAVYTFETLLHQELGKGPTKEELCKSIQ
Splooce       DMNLNVINEGGIDKLGEYMEKLSRLAYHPLKMQSCYEKMESLRLDGLQQRFDVSSTSVFKQRAQIHMREQMDNAVYTFETLLHQELGKGPTKEELCKSIQ

Uniprot       RVLERVLKKYDYDSSSVRKRFFREALLQISIPFLLKKLAPTCKSELPRFQELIFEDFARFILVENTYEEVVLQTVMKDILQAVKEAAVQRKHNLYRDSMV
Splooce       RVLERVLKKYDYDSSSVRKRFFREALLQISIPFLLKKLAPTCKSELPRFQELIFEDFARFILVENTYEEVVLQTVMKDILQAVKEAAVQRKHNLYRDSMV

Uniprot       MHNSDPNLHLLAEGAPIDWGEEYSNSGGGGSPSPSTPESATLSEKRRRAKQVVSVVQDEEVGLPFEASPESPPPASPDGVTEIRGLLAQGLRPESPPPAG
Splooce       MHNSDPNLHLLAEGAPIDWGEEYSNSGGGGSPSPSTPESATLSEKRRRAKQVVSVVQDEEVGLPFEASPESPPPASPDGVTEIRGLLAQGLRPESPPPAG

Uniprot       PLLNGAPAGESPQPKAAPEASSPPASPLQHLLPGKAVDLGPPKPSDQETGEQVSSPSSHPALHTTTEDSAGVQTEF
Splooce       PLLNGAPAGESPQPKAAPEASSPPASPLQHLLPGKAVDLGPPKPSDQETGEQVSSPSSHPALHTTTEDSAGVQTEF

----------------------------------------------------------------------------------------------------

P04075 (Uniprot)	versus
NM_000034#(-s-:16_A1237256709659) (Splooce)

For more details about the Alternative Splicing Event -> Link to Splooce page

Peptides that support the ASE (Splooce-specific):
ISSEAPELATTSTMPYQYPALTPEQK (MAXQUANT)

Alignment:
Uniprot       --------------------------------------------MPYQYPALTPEQKKELSDIAHRIVAPGKGILAADESTGSIAKRLQSIGTENTEENR
Splooce       MLAAPSPPELSLAKDLFLLTTKGLRLDFQGRISSEAPELATTSTMPYQYPALTPEQKKELSDIAHRIVAPGKGILAADESTGSIAKRLQSIGTENTEENR

Uniprot       RFYRQLLLTADDRVNPCIGGVILFHETLYQKADDGRPFPQVIKSKGGVVGIKVDKGVVPLAGTNGETTTQGLDGLSERCAQYKKDGADFAKWRCVLKIGE
Splooce       RFYRQLLLTADDRVNPCIGGVILFHETLYQKADDGRPFPQVIKSKGGVVGIKVDKGVVPLAGTNGETTTQGLDGLSERCAQYKKDGADFAKWRCVLKIGE

Uniprot       HTPSALAIMENANVLARYASICQQNGIVPIVEPEILPDGDHDLKRCQYVTEKVLAAVYKALSDHHIYLEGTLLKPNMVTPGHACTQKFSHEEIAMATVTA
Splooce       HTPSALAIMENANVLARYASICQQNGIVPIVEPEILPDGDHDLKRCQYVTEKVLAAVYKALSDHHIYLEGTLLKPNMVTPGHACTQKFSHEEIAMATVTA

Uniprot       LRRTVPPAVTGITFLSGGQSEEEASINLNAINKCPLLKPWALTFSYGRALQASALKAWGGKKENLKAAQEEYVKRALANSLACQGKYTPSGQAGAAASES
Splooce       LRRTVPPAVTGITFLSGGQSEEEASINLNAINKCPLLKPWALTFSYGRALQASALKAWGGKKENLKAAQEEYVKRALANSLACQGKYTPSGQAGAAASES

Uniprot       LFVSNHAY
Splooce       LFVSNHAY

----------------------------------------------------------------------------------------------------

Q9Y285 (Uniprot)	versus
NM_004461#(-s-:19_F2073774096819) (Splooce)

For more details about the Alternative Splicing Event -> Link to Splooce page

Peptides that support the ASE (Splooce-specific):
GTSITGSWTR (MAXQUANT)

Alignment:
Uniprot       MADGQVAELLLRRLEASDGGLDSAELAAELGMEHQAVVGAVKSLQALGEVIEAELRSTKHWELTAEGEEIAREGSHEARVFRSIPPEGLAQSELMRLPSG
Splooce       MADGQVAELLLRRLEASDGGLDSAELAAELGMEHQAVVGAVKSLQALGEVIEAELRSTKHWELTAEGEEIAREGSHEARVFRSIPPEGLAQSELMRLPSG

Uniprot       KVGFSKAMSNKWIRVDKSAADGPRVFRVVDSMEDEVQRRLQLVRGGQAEKLGEKERSELRKRKLLAEVTLKTYWVSKGSAFSTSISKQETELSPEMISSG
Splooce       KVGFSKAMSNKWIRVDKSAADGPRVFRVVDSMEDEVQRRLQLVRGGQAEKLGEKERSELRKRKLLAEVTLKTYWVSKGSAFSTSISKQETELSPEMISSG

Uniprot       SWRDRPFKPYNFLAHGVLPDSGHLHPLLKVRSQFRQIFLEMGFTEMPTDNFIESSFWNFDALFQPQQHPARDQHDTFFLRDPAEALQLPMDYVQRVKRTH
Splooce       SWRDRPFKPYNFLAHGVLPDSGHLHPLLKVRSQFRQIFLEMGFTEMPTDNFIESSFWNFDALFQPQQHPARDQHDTFFLRG-----TSITGSWTRPGKTY

Uniprot       SQGGYGSQGYKYNWKLDEARKNLLRTHTTSASARALYRLAQKKPFTPVKYFSIDRVFRNETLDATHLAEFHQIEGVVADHGLTLGHLMGVLREFFTKLGI
Splooce       CEPTPHQPAPVRSTALPR-RSPSLRSSTSPSTA-----YSGMRPWTPRTWLSSTR---------------------------------------------

Uniprot       TQLRFKPAYNPYTEPSMEVFSYHQGLKKWVEVGNSGVFRPEMLLPMGLPENVSVIAWGLSLERPTMIKYGINNIRELVGHKVNLQMVYDSPLCRLDAEPR
Splooce       SRAWWRIMVSPWATS-------------WAFCGSS---------------SPSWVSRNSASSQPTTHT--------------------QSPAWRCSATTK

Uniprot       PPPTQEAA
Splooce       A-------

----------------------------------------------------------------------------------------------------

P55072 (Uniprot)	versus
NM_007126#(-s-:9_V4728223921908) (Splooce)

For more details about the Alternative Splicing Event -> Link to Splooce page

Peptides that support the ASE (Splooce-specific):
YPVEHPDK (MAXQUANT + PEAKS)

Alignment:
Uniprot       MASGADSKGDDLSTAILKQKNRPNRLIVDEAINEDNSVVSLSQPKMDELQLFRGDTVLLKGKKRREAVCIVLSDDTCSDEKIRMNRVVRNNLRVRLGDVI
Splooce       MASGADSKGDDLSTAILKQKNRPNRLIVDEAINEDNSVVSLSQPKMDELQLFRGDTVLLKGKKRREAVCIVLSDDTCSDEKIRMNRVVRNNLRVRLGDVI

Uniprot       SIQPCPDVKYGKRIHVLPIDDTVEGITGNLFEVYLKPYFLEAYRPIRKGDIFLVRGGMRAVEFKVVETDPSPYCIVAPDTVIHCEGEPIKREDEEESLNE
Splooce       SIQPCPDVKYGKRIHVLPIDDTVEGITGNLFEVYLKPYFLEAYRPIRKGDIFLVRGGMRAVEFKVVETDPSPYCIVAPDTVIHCEGEPIKREDEEESLNE

Uniprot       VGYDDIGGCRKQLAQIKEMVELPLRHPALFKAIGVKPPRGILLYGPPGTGKTLIARAVANETGAFFFLINGPEIMSKLAGESESNLRKAFEEAEKNAPAI
Splooce       VGYDDIGGCRKQLAQIKEMVELPLRHPALFKAIGVKPPRGILLYGPPGTGKTLIARAVANETGAFFFLINGPEIMSKLAGESESNLRKAFEEAEKNAPAI

Uniprot       IFIDELDAIAPKREKTHGEVERRIVSQLLTLMDGLKQRAHVIVMAATNRPNSIDPALRRFGRFDREVDIGIPDATGRLEILQIHTKNMKLADDVDLEQVA
Splooce       IFIDELDAIAPKREKTHGEVERRIVSQLLTLMDGLKQRAHVIVMAATNRPNSIDPALRRFGRFDREVDIGIPDATGRLEILQIHTKNMKLADDVDLEQVA

Uniprot       NETHGHVGADLAALCSEAALQAIRKKMDLIDLEDETIDAEVMNSLAVTMDDFRWALSQSNPSALRETVVEVPQVTWEDIGGLEDVKRELQELVQYPVEHP
Splooce       NETHGHVGADLAALCSEAALQAIRKKMDLIDLEDETIDAEVMNSLAVTMDDFR-----------------------------------------YPVEHP

Uniprot       DKFLKFGMTPSKGVLFYGPPGCGKTLLAKAIANECQANFISIKGPELLTMWFGESEANVREIFDKARQAAPCVLFFDELDSIAKARGGNIGDGGGAADRV
Splooce       DKFLKFGMTPSKGVLFYGPPGCGKTLLAKAIANECQANFISIKGPELLTMWFGESEANVREIFDKARQAAPCVLFFDELDSIAKARGGNIGDGGGAADRV

Uniprot       INQILTEMDGMSTKKNVFIIGATNRPDIIDPAILRPGRLDQLIYIPLPDEKSRVAILKANLRKSPVAKDVDLEFLAKMTNGFSGADLTEICQRACKLAIR
Splooce       INQILTEMDGMSTKKNVFIIGATNRPDIIDPAILRPGRLDQLIYIPLPDEKSRVAILKANLRKSPVAKDVDLEFLAKMTNGFSGADLTEICQRACKLAIR

Uniprot       ESIESEIRRERERQTNPSAMEVEEDDPVPEIRRDHFEEAMRFARRSVSDNDIRKYEMFAQTLQQSRGFGSFRFPSGNQGGAGPSQGSGGGTGGSVYTEDN
Splooce       ESIESEIRRERERQTNPSAMEVEEDDPVPEIRRDHFEEAMRFARRSVSDNDIRKYEMFAQTLQQSRGFGSFRFPSGNQGGAGPSQGSGGGTGGSVYTEDN

Uniprot       DDDLYG
Splooce       DDDLYG

----------------------------------------------------------------------------------------------------

Q8N201 (Uniprot)	versus
NM_001080453#(-s-s-s-:7_I7217046819881) (Splooce)

For more details about the Alternative Splicing Event -> Link to Splooce page

Peptides that support the ASE (Splooce-specific):
TLCATGHR (MAXQUANT)

Alignment:
Uniprot       MNRAKPTTVRRPSAAAKPSGHPPPGDFIALGSKGQANESKTASTLLKPAPSGLPSERKRDAAAALSSASALTGLTKRPKLSSTPPLSALGRLAEAAVAEK
Splooce       MNRAKPTTVRRPSAAAKPSGHPPPGDFIALGSKGQANESKTASTLLKPAPSGLPSERKRDAAAALSSASALTGLTKRPKLSSTPPLSALGRLAEAAVAEK

Uniprot       RAISPSIKEPSVVPIEVLPTVLLDEIEAAELEGNDDRIEGVLCGAVKQLKVTRAKPDSTLYLSLMYLAKIKPNIFATEGVIEALCSLLRRDASINFKAKG
Splooce       RAISPSIKEPSVVPIEVLPTVLLDEIEAAELEGNDDRIEGVLCGAVKQLKVTRAKPDSTLYLSLMYLAKIKPNIFATEGVIEALCSLLRRDASINFKAKG

Uniprot       NSLVSVLACNLLMAAYEEDENWPEIFVKVYIEDSLGERIWVDSPHCKTFVDNIQTAFNTRMPPRSVLLQGEAGRVAGDLGAGSSPHPSLTEEEDSQTELL
Splooce       NSLVSVLACNLLMAAYEEDENWPEIFVKVYIEDSLGERIWVDSPHCKTFVDNIQTAFNTRMPPRSVLLQGEAGRVAGDLGAGSSPHPSLTEEEDSQTELL

Uniprot       IAEEKLSPEQEGQLMPRYEELAESVEEYVLDMLRDQLNRRQPIDNVSRNLLRLLTSTCGYKEVRLLAVQKLEMWLQNPKLTRPAQDLLMSVCMNCNTHGS
Splooce       IAEEKLSPEQEGQLMPRYEELAESVEEYVLDMLRDQLNRRQPIDNVSRNLLRLLTSTCGYKEVRLLAVQKLEMWLQNPKLTRPAQDLLMSVCMNCNTHGS

Uniprot       EDMDVISHLIKIRLKPKVLLNHFMLCIRELLSAHKDNLGTTIKLVIFNELSSARNPNNMQVLYTALQHSSELAPKFLAMVFQDLLTNKDDYLRASRALLR
Splooce       EDMDVISHLIKIRLKPKVLLNHFMLCIRELLSAHKDNLGTTIKLVIFNELSSARNPNNMQVLYTALQHSSELAPKFLAMVFQDLLTNKDDYLRASRALLR

Uniprot       EIIKQTKHEINFQAFCLGLMQERKEPQYLEMEFKERFVVHITDVLAVSMMLGITAQVKEAGIAWDKGEKRNLEVLRSFQNQIAAIQRDAVWWLHTVVPSI
Splooce       EIIKQTKHEINFQAFCLGLMQERKEPQYLEMEFKERFVVHITDVLAVSMMLGITAQVKEAGIAWDKGEKRNLEVLRSFQNQIAAIQRDAVWWLHTVVPSI

Uniprot       SKLAPKDYVHCLHKVLFTEQPETYYKWDNWPPESDRNFFLRLCSEVPILEDTLMRILVIGLSRELPLGPADAMELADHLVKRAAAVQADDVEVLKVGRTQ
Splooce       SKLAPKDYVHCLHKVLFTEQPETYYKWDNWPPESDRNFFLRLCSEVPILEDTLMRILVIGLSRELPLGPADAMELADHLVKRAAAVQADDVEVLKVGRTQ

Uniprot       LIDAVLNLCTYHHPENIQLPPGYQPPNLAISTLYWKAWPLLLVVAAFNPENIGLAAWEEYPTLKMLMEMVMTNNYSYPPCTLTDEETRTEMLNRELQTAQ
Splooce       LIDAVLNLCTYHHPENIQLPPGYQPPNLAISTLYWKAWPLLLVVAAFNPENIGLAAWEEYPTLKMLMEMVMTNNYSYPPCTLTDEETRTEMLNRELQTAQ

Uniprot       REKQEILAFEGHLAAASTKQTITESSSLLLSQLTSLDPQGPPRRPPPHILDQVKSLNQSLRLGHLLCRSRNPDFLLHIIQRQASSQSMPWLADLVQSSEG
Splooce       REKQEILAFEGHLAAASTKQTITESSSLLLSQLTSLDPQGPPRRPPPHILDQVKSLNQSLRLGHLLCRSRNPDFLLHIIQRQASSQSMPWLADLVQSSEG

Uniprot       SLDVLPVQCLCEFLLHDAVDDAASGEEDDEGESKEQKAKKRQRQQKQRQLLGRLQDLLLGPKADEQTTCEVLDYFLRRLGSSQVASRVLAMKGLSLVLSE
Splooce       SLDVLPVQCLCEFLLHDAVDDAASGEEDDEGESKEQKAKKRQRQQKQRQLLGRLQDLLLGPKADEQTTCEVLDYFLRRLGSSQVASRVLAMKGLSLVLSE

Uniprot       GSLRDGEEKEPPMEEDVGDTDVLQGYQWLLRDLPRLPLFDSVRSTTALALQQAIHMETDPQTISAYLIYLSQHTPVEEQAQHSDLALDVARLVVERSTIM
Splooce       GSLRDGEEKEPPMEEDVGDTDVLQGYQWLLRDLPRLPLFDSVRSTTALALQQAIHMETDPQTISAYLIYLSQHTPVEEQAQHSDLALDVARLVVERSTIM

Uniprot       SHLFSKLSPSAASDAVLSALLSIFSRYVRRMRQSKEGEEVYSWSESQDQVFLRWSSGETATMHILVVHAMVILLTLGPPRADDSEFQALLDIWFPEEKPL
Splooce       SHLFSKLSPSAASDAVLSALLSIFSRYVRRMRQSKEGEEVYSWSESQDQVFLRWSSGETATMHILVVHAMVILLTLGPPRADDSEFQALLDIWFPEEKPL

Uniprot       PTAFLVDTSEEALLLPDWLKLRMIRSEVLRLVDAALQDLEPQQLLLFVQSFGIPVSSMSKLLQFLDQAVAHDPQTLEQNIMDKNYMAHLVEVQHERGASG
Splooce       PTAFLVDTSEEALLLPDWLKLRMIRSEVLRLVDAALQDLEPQQLLLFVQSFGIPVSSMSKLLQFLDQAVAHDPQTLEQNIMDK----TLCATGHR-----

Uniprot       GQTFHSLLTASLPPRRDSTEAPKPKSSPEQPIGQGRIRVGTQLRVLGPEDDLAGMFLQIFPLSPDPRWQSSSPRPVALALQQALGQELARVVQGSPEVPG
Splooce       -------LLLALPEG-----APADAAVAGQPWRGGRAPAGTAQDACQP------------------------------GLSRAQAQ--------------

Uniprot       ITVRVLQALATLLSSPHGGALVMSMHRSHFLACPLLRQLCQYQRCVPQDTGFSSLFLKVLLQMLQWLDSPGVEGGPLRAQLRMLASQASAGRRLSDVRGG
Splooce       ----------------------------------------------------------------------------------------------------

Uniprot       LLRLAEALAFRQDLEVVSSTVRAVIATLRSGEQCSVEPDLISKVLQGLIEVRSPHLEELLTAFFSATADAASPFPACKPVVVVSSLLLQEEEPLAGGKPG
Splooce       ----------------------------------------------------------------------------------------------------

Uniprot       ADGGSLEAVRLGPSSGLLVDWLEMLDPEVVSSCPDLQLRLLFSRRKGKGQAQVPSFRPYLLTLFTHQSSWPTLHQCIRVLLGKSREQRFDPSASLDFLWA
Splooce       ----------------------------------------------------------------------------------------------------

Uniprot       CIHVPRIWQGRDQRTPQAAAGGAGAAGPGPGAHQPGGADPGRGGDAEPGRGHSRLQPHPGPAAPAAQLLLWGR
Splooce       -------------------------------------------------------------------------

----------------------------------------------------------------------------------------------------

P22314 (Uniprot)	versus
NM_153280#(-s-s-s-s-s-:X_U5577645170028) (Splooce)

For more details about the Alternative Splicing Event -> Link to Splooce page

Peptides that support the ASE (Splooce-specific):
QLLHNFPPDQYYNQEWTLWDR (MAXQUANT)

Alignment:
Uniprot       MSSSPLSKKRRVSGPDPKPGSNCSPAQSVLSEVPSVPTNGMAKNGSEADIDEGLYSRQLYVLGHEAMKRLQTSSVLVSGLRGLGVEIAKNIILGGVKAVT
Splooce       MSSSPLSKKRRVSGPDPKPGSNCSPAQSVLSEVPSVPTNGMAKNGSEADIDEGLYSRQLYVLGHEAMKRLQTSSVLVSGLRGLGVEIAKNIILGGVKAVT

Uniprot       LHDQGTAQWADLSSQFYLREEDIGKNRAEVSQPRLAELNSYVPVTAYTGPLVEDFLSGFQVVVLTNTPLEDQLRVGEFCHNRGIKLVVADTRGLFGQLFC
Splooce       LHDQGTAQWADLSSQFYLREEDIGKNRAEVSQPRLAELNSYVPVTAYTGPLVEDFLSGFQVVVLTNTPLEDQLRVGEFCHNRGIKLVVADTRGLFGQLFC

Uniprot       DFGEEMILTDSNGEQPLSAMVSMVTKDNPGVVTCLDEARHGFESGDFVSFSEVQGMVELNGNQPMEIKVLGPYTFSICDTSNFSDYIRGGIVSQVKVPKK
Splooce       DFGEEMILTDSNGEQPLSAMVSMVTKDNPGVVTCLDEARHGFESGDFVSFSEVQGMVELNGNQPMEIKVLGPYTFSICDTSNFSDYIRGGIVSQVKVPKK

Uniprot       ISFKSLVASLAEPDFVVTDFAKFSRPAQLHIGFQALHQFCAQHGRPPRPRNEEDAAELVALAQAVNARALPAVQQNNLDEDLIRKLAYVAAGDLAPINAF
Splooce       ISFKSLVASLAEPDFVVTDFAKFSRPAQLHIGFQALHQFCAQHGRPPRPRNEEDAAELVALAQAVNARALPAVQQNNLDEDLIRKLAYVAAGDLAPINAF

Uniprot       IGGLAAQEVMKACSGKFMPIMQWLYFDALECLPEDKEVLTEDKCLQRQNRYDGQVAVFGSDLQEKLGKQKYFLVGAGAIGCELLKNFAMIGLGCGEGGEI
Splooce       IGGLAAQEVMKACSGKFMPIMQWLYFDALECLPEDKEVLTEDKCLQRQNRYDGQVAVFGSDLQEKLGKQKYFLVGAGAIGCELLKNFAMIGLGCGEGGEI

Uniprot       IVTDMDTIEKSNLNRQFLFRPWDVTKLKSDTAAAAVRQMNPHIRVTSHQNRVGPDTERIYDDDFFQNLDGVANALDNVDARMYMDRRCVYYRKPLLESGT
Splooce       IVTDMDTIEKSNLNRQFLFRPWDVTKLKSDTAAAAVRQMNPHIRVTSHQNRVGPDTERIYDDDFFQNLDGVANALDNVDARMYMDRRCVYYRKPLLESGT

Uniprot       LGTKGNVQVVIPFLTESYSSSQDPPEKSIPICTLKNFPNAIEHTLQWARDEFEGLFKQPAENVNQYLTDPKFVERTLRLAGTQPLEVLEAVQRSLVLQRP
Splooce       LGTKGNVQVVIPFLTESYSSSQDPPEKSIPICTLKNFPNAIEHTLQWARDEFEGLFKQPAENVNQYLTDPKFVERTLRLAGTQPLEVLEAVQRSLVLQRP

Uniprot       QTWADCVTWACHHWHTQYSNNIRQLLHNFPPDQLTSSGAPFWSGPKRCPHPLTFDVNNPLHLDYVMAAANLFAQTYGLTGSQDRAAVATFLQSVQVPEFT
Splooce       QTWADCVTWACHHWHTQYSNNIRQLLHNFPPD--------------------------------------------------------------------

Uniprot       PKSGVKIHVSDQELQSANASVDDSRLEELKATLPSPDKLPGFKMYPIDFEKDDDSNFHMDFIVAASNLRAENYDIPSADRHKSKLIAGKIIPAIATTTAA
Splooce       ----------------------------------------------------------------------------------------------------

Uniprot       VVGLVCLELYKVVQGHRQLDSYKNGFLNLALPFFGFSEPLAAPRHQYYNQEWTLWDRFEVQGLQPNGEEMTLKQFLDYFKTEHKLEITMLSQGVSMLYSF
Splooce       ---------------------------------------------QYYNQEWTLWDRFEVQGLQPNGEEMTLKQFLDYFKTEHKLEITMLSQGVSMLYSF

Uniprot       FMPAAKLKERLDQPMTEIVSRVSKRKLGRHVRALVLELCCNDESGEDVEVPYVRYTIR
Splooce       FMPAAKLKERLDQPMTEIVSRVSKRKLGRHVRALVLELCCNDESGEDVEVPYVRYTIR

----------------------------------------------------------------------------------------------------

B7Z840 (Uniprot)	versus
NM_005051#(-s-:3_Q2719665117827) (Splooce)

For more details about the Alternative Splicing Event -> Link to Splooce page

Peptides that support the ASE (Splooce-specific):
DVVENGENYK (MAXQUANT)

Alignment:
Uniprot       MAALDSLSLFTSLGLSEQKARETLKNSALSAQLREAATQAQQTLGSTIDKATGILLYGLASRLRDTRRLSFLVSYIASKKIHTEPQLSAALEYVRSHPLD
Splooce       MAALDSLSLFTSLGLSEQKARETLKNSALSAQLREAATQAQQTLGSTIDKATGILLYGLASRLRDTRRLSFLVSYIASKKIHTEPQLSAALEYVRSHPLD

Uniprot       PIDTVDFERECGVGVIVTPEQIEEAVEAAINRHRPQLLVERYHFNMGLLMGEARAVLKWADGKMIKNEVDMQVLHLLGPKLEADLEKKFKVAKARLEETD
Splooce       PIDTVDFERECGVGVIVTPEQIEEAVEAAINRHRPQLLVERYHFNMGLLMGEARAVLKWADGKMIKNEVDMQVLHLLGPKLEADLEKKFKVAKARLEETD

Uniprot       RRTAKDVVENGETADQTLSLMEQLRGEALKFHKPGENYKTPGYVVTPHTMNLLKQHLEITGGQVRTRFPPEPNGILHIGHAKAINFNFGYAKANNGICFL
Splooce       RRTAKDVVENG------------------------ENYKTPGYVVTPHTMNLLKQHLEITGGQVRTRFPPEPNGILHIGHAKAINFNFGYAKANNGICFL

Uniprot       RFDDTNPEKEEAKFFTAICDMVAWLGYTPYKVTYASDYFDQLYAWAVELIRRGLAYVCHQRGEELKGHNTLPSPWRDRPMEESLLLFEAMRKGKFSEGEA
Splooce       RFDDTNPEKEEAKFFTAICDMVAWLGYTPYKVTYASDYFDQLYAWAVELIRRGLAYVCHQRGEELKGHNTLPSPWRDRPMEESLLLFEAMRKGKFSEGEA

Uniprot       TLRMKLVMEDGKMDPVAYRVKYTPHHRTGDKWCIYPTYDYTHCLCDSIEHITHSLCTKEFQARRSSYFWLCNALDVYCPVQWEYGRLNLHYAVVSKRKIL
Splooce       TLRMKLVMEDGKMDPVAYRVKYTPHHRTGDKWCIYPTYDYTHCLCDSIEHITHSLCTKEFQARRSSYFWLCNALDVYCPVQWEYGRLNLHYAVVSKRKIL

Uniprot       QLVATGAVRDWDDPRLFTLTALRRRGFPPEAINNFCARVGVTVAQTTMEPHLLEACVRDVLNDTAPRAMAVLESLRVIITNFPAAKSLDIQVPNFPADET
Splooce       QLVATGAVRDWDDPRLFTLTALRRRGFPPEAINNFCARVGVTVAQTTMEPHLLEACVRDVLNDTAPRAMAVLESLRVIITNFPAAKSLDIQVPNFPADET

Uniprot       KGFHQVPFAPIVFIERTDFKEEPEPGFKRLAWGQPVGLRHTGYVIELQHVVKGPSGCVESLEVTCRRADAGEKPKAFIHWVSQPLMCEVRLYERLFQHKN
Splooce       KGFHQVPFAPIVFIERTDFKEEPEPGFKRLAWGQPVGLRHTGYVIELQHVVKGPSGCVESLEVTCRRADAGEKPKAFIHWVSQPLMCEVRLYERLFQHKN

Uniprot       PEDPTEVPGGFLSDLNLASLHVVDAALVDCSVALAKPFDKFQFERLGYFSVDPDSHQGKLVFNRTVTLKEDPGKV
Splooce       PEDPTEVPGGFLSDLNLASLHVVDAALVDCSVALAKPFDKFQFERLGYFSVDPDSHQGKLVFNRTVTLKEDPGKV

----------------------------------------------------------------------------------------------------

P10809 (Uniprot)	versus
NM_199440#(-s-s-s-s-s-s-s-:2_H3698120817513) (Splooce)

For more details about the Alternative Splicing Event -> Link to Splooce page

Peptides that support the ASE (Splooce-specific):
RGLELLK (PEAKS)

Alignment:
Uniprot       MLRLPTVFRQMRPVSRVLAPHLTRAYAKDVKFGADARALMLQGVDLLADAVAVTMGPKGRTVIIEQSWGSPKVTKDGVTVAKSIDLKDKYKNIGAKLVQD
Splooce       MLRLPTVFRQMRPVSRVLAPHLTRAYAKDVKFGADARALMLQGVDLLADAVAVTMGPKGRTVIIEQSWGSPKVTKDGVTVAKSIDLKDKYKNIGAKLVQD

Uniprot       VANNTNEEAGDGTTTATVLARSIAKEGFEKISKGANPVEIRRGVMLAVDAVIAELKKQSKPVTTPEEIAQVATISANGDKEIGNIISDAMKKVGRKGVIT
Splooce       VANNTNEEAGDGTTTATVLARSIAKEGFEKISKGANPVEIRR==========================================================

Uniprot       VKDGKTLNDELEIIEGMKFDRGYISPYFINTSKGQKCEFQDAYVLLSEKKISSIQSIVPALEIANAHRKPLVIIAEDVDGEALSTLVLNRLKVGLQVVAV
Splooce       ====================================================================================================

Uniprot       KAPGFGDNRKNQLKDMAIATGGAVFGEEGLTLNLEDVQPHDLGKVGEVIVTKDDAMLLKGKGDKAQIEKRIQEIIEQLDVTTSEYEKEKLNERLAKLSDG
Splooce       ====================================================================================================

Uniprot       VAVLKVGGTSDVEVNEKKDRVTDALNATRAAVEEGIVLGGGCALLRCIPALDSLTPANEDQKIGIEIIKRTLKIPAMTIAKNAGVEGSLIVEKIMQSSSE
Splooce       ===============================================================GIEIIKRTLKIPAMTIAKNAGVEGSLIVEKIMQSSSE

Uniprot       VGYDAMAGDFVNMVEKGIIDPTKVVRTALLDAAGVASLLTTAEVVVTEIPKEEKDPGMGAMGGMGGGMGGGMF
Splooce       VGYDAMAGDFVNMVEKGIIDPTKVVRTALLDAAGVASLLTTAEVVVTEIPKEEKDPGMGAMGGMGGGMGGGMF

----------------------------------------------------------------------------------------------------

P29401 (Uniprot)	versus
NM_001135055#(-s-s-s-s-s-s-s-s-s-s-s-:3_T3333382168575) (Splooce)

For more details about the Alternative Splicing Event -> Link to Splooce page

Peptides that support the ASE (Splooce-specific):
ISSDLDGHPVPPVQVLGKK (MAXQUANT)

Alignment:
Uniprot       MESYHKPDQQKLQALKDTANRLRISSIQATTAAGSGHPTSCCSAAEIMAVLFFHTMRYKSQDPRNPHNDRFVLSKGHAAPILYAVWAEAGFLAEAELLNL
Splooce       MESYHKPDQQKLQALKDTANRLRISSIQATTAAGSGHPTSCCSAAEIMAVLFFHTMRYKSQDPRNPHNDRFVLSKGHAAPILYAVWAEAGFLAEAELLNL

Uniprot       RKISSDLDGHPVPKQAFTDVATGSLGQGLGAACGMAYTGKYFDKASYRVYCLLGDGELSEGSVWEAMAFASIYKLDNLVAILDINRLGQSDPAPLQHQMD
Splooce       RKISSDLDGHPVP-------PVQVLGKKRGGCAREAG--------------VLGPG--------------------------------------------

Uniprot       IYQKRCEAFGWHAIIVDGHSVEELCKAFGQAKHQPTAIIAKTFKGRGITGVEDKESWHGKPLPKNMAEQIIQEIYSQIQSKKKILATPPQEDAPSVDIAN
Splooce       -------------------------------------------PGPGCP----KSPWNHLQLPS---------------TSRTIAAS-------------

Uniprot       IRMPSLPSYKVGDKIATRKAYGQALAKLGHASDRIIALDGDTKNSTFSEIFKKEHPDRFIECYIAEQNMVSIAVGCATRNRTVPFCSTFAAFFTRAFDQI
Splooce       -----------------------------HSSP------GDI----------------------------------------------------------

Uniprot       RMAAISESNINLCGSHCGVSIGEDGPSQMALEDLAMFRSVPTSTVFYPSDGVATEKAVELAANTKGICFIRTSRPENAIIYNNNEDFQVGQAKVVLKSKD
Splooce       ----------------------------------------------------------------------------------------------------

Uniprot       DQVTVIGAGVTLHEALAAAELLKKEKINIRVLDPFTIKPLDRKLILDSARATKGRILTVEDHYYEGGIGEAVSSAVVGEPGITVTHLAVNRVPRSGKPAE
Splooce       ----------------------------------------------------------------------------------------------------

Uniprot       LLKMFGIDRDAIAQAVRGLITKA
Splooce       -----------------------

----------------------------------------------------------------------------------------------------

Q9Y6D9 (Uniprot)	versus
NM_003550#(-t:7_M7111184016371) (Splooce)

For more details about the Alternative Splicing Event -> Link to Splooce page

Peptides that support the ASE (Splooce-specific):
YAGHPGVLR (MAXQUANT)

Alignment:
Uniprot       MEDLGENTMVLSTLRSLNNFISQRVEGGSGLDISTSAPGSLQMQYQQSMQLEERAEQIRSKSHLIQVEREKMQMELSHKRARVELERAASTSARNYEREV
Splooce       MEDLGENTMVLSTLRSLNNFISQRVEGGSGLDISTSAPGSLQMQYQQSMQLEERAEQIRSKSHLIQVEREKMQMELSHKRARVELERAASTSARNYEREV

Uniprot       DRNQELLTRIRQLQEREAGAEEKMQEQLERNRQCQQNLDAASKRLREKEDSLAQAGETINALKGRISELQWSVMDQEMRVKRLESEKQELQEQLDLQHKK
Splooce       DRNQELLTRIRQLQEREAGAEEKMQEQLERNRQCQQNLDAASKRLREKEDSLAQAGETINALKGRISELQWSVMDQEMRVKRLESEKQELQEQLDLQHKK

Uniprot       CQEANQKIQELQASQEARADHEQQIKDLEQKLSLQEQDAAIVKNMKSELVRLPRLERELKQLREESAHLREMRETNGLLQEELEGLQRKLGRQEKMQETL
Splooce       CQEANQKIQELQASQEARADHEQQIKDLEQKLSLQEQDAAIVKNMKSELVRLPRLERELKQLREESAHLREMRETNGLLQEELEGLQRKLGRQEKMQETL

Uniprot       VGLELENERLLAKLQSWERLDQTMGLSIRTPEDLSRFVVELQQRELALKDKNSAVTSSARGLEKARQQLQEELRQVSGQLLEERKKRETHEALARRLQKR
Splooce       VGLELENERLLAKLQSWERLDQTMGLSIRTPEDLSRFVVELQQRELALKDKNSAVTSRQR----------------------------------------

Uniprot       VLLLTKERDGMRAILGSYDSELTPAEYSPQLTRRMREAEDMVQKVHSHSAEMEAQLSQALEELGGQKQRADMLEMELKMLKSQSSSAEQSFLFSREEADT
Splooce       ------------------------------------------------PGAGEGQAAAAGGAPAGQ----------------------------------

Uniprot       LRLKVEELEGERSRLEEEKRMLEAQLERRALQGDYDQSRTKVLHMSLNPTSVARQRLREDHSQLQAECERLRGLLRAMERGGTVPADLEAAAASLPSSKE
Splooce       ---------------------------RPAVGGEEE----------------------------------------ARDPRGAGPEAPETGPAAHQG---

Uniprot       VAELKKQVESAELKNQRLKEVFQTKIQEFRKACYTLTGYQIDITTENQYRLTSLYAEHPGDCLIFKATSPSGSKMQLLETEFSHTVGELIEVHLRRQDSI
Splooce       ---------------------------------------------------AGRYAGHPG---VLRQRADPGR--VLTPADAAHAGG-------------

Uniprot       PAFLSSLTLELFSRQTVA
Splooce       ------------------

----------------------------------------------------------------------------------------------------

Q5XPI4 (Uniprot)	versus
NM_022064#(-s-s-:3_R9689711184387) (Splooce)

For more details about the Alternative Splicing Event -> Link to Splooce page

Peptides that support the ASE (Splooce-specific):
IQQAAER (MAXQUANT)

Alignment:
Uniprot       MASKGAGMSFSRKSYRLTSDAEKSRVTGIVQEKLLNDYLNRIFSSSEHAPPAATSRKPLNFQNLPEHLDQLLQVDNEEEESQGQVEGRLGPSTVVLDHTG
Splooce       MASKGAGMSFSRKSYRLTSDAEKSRVTGIVQEKLLNDYLNRIFSSSEHAPPAATSRKPLNFQNLPEHLDQLLQVDNEEEESQGQVEGRLGPSTVVLDHTG

Uniprot       GFEGLLLVDDDLLGVIGHSNFGTIRSTTCVYKGKWLYEVLISSQGLMQIGWCTISCRFNQEEGVGDTHNSYAYDGNRVRKWNVTTTNYGKAWAAGDIVSC
Splooce       GFEGLLLVDDDLLGVIGHSNFGTIRSTTCVYKGKWLYEVLISSQGLMQIGWCTISCRFNQEEGVGDTHNSYAYDGNRVRKWNVTTTNYGKAWAAGDIVSC

Uniprot       LIDLDDGTLSFCLNGVSLGTAFENLSRGLGMAYFPAISLSFKESVAFNFGSRPLRYPVAGYRPLQDPPSADLVRAQRLLGCFRAVLSVELDPVEGRLLDK
Splooce       LIDLDDGTLSFCLNGVSLGTAFENLSRGLGMAYFPAISLSFKESVAFNFGSRPLRYPVAGYRPLQDPPSADLVRAQRLLGCFRAVLSVELDPVEGRLLDK

Uniprot       ESSKWRLRGQPTVLLTLAHIFHHFAPLLRKVYLVEAVLMSFLLGIVEKGTPTQAQSVVHQVLDLLWLFMEDYEVQDCLKQLMMSLLRLYRFSPIVPDLGL
Splooce       ESSKWRLRGQPTVLLTLAHIFHHFAPLLRKVYLVEAVLMSFLLGIVEKGTPTQAQSVVHQVLDLLWLFMEDYEVQDCLKQLMMSLLRLYRFSPIVPDLGL

Uniprot       QIHYLRLTIAILRHEKSRKFLLSNVLFDVLRSVVFFYIKSPLRVEEAGLQELIPTTWWPHCSSREGKESTEMKEETAEERLRRRAYERGCQRLRKRIEVV
Splooce       QIHYLRLTIAILRHEKSRKFLLSNVLFDVLRSVVFFYIKSPLRVEEAGLQELIPTTWWPHCSSREGKESTEMKEETAEERLRRRAYERGCQRLRKRIEVV

Uniprot       EELQVQILKLLLDNKDDNGGEASRYIFLTKFRKFLQENASGRGNMPMLCPPEYMVCFLHRLISALRYYWDEYKASNPHASFSEEAYIPPQVFYNGKVDYF
Splooce       EELQVQILKLLLDNKDDNGGEASRYIFLTKFRKFLQENASGRGNMPMLCPPEYMVCFLHRLISALRYYWDEYKASNPHASFSEEAYIPPQVFYNGKVDYF

Uniprot       DLQRLGGLLSHLRKTLKDDLASKANIVIDPLELQSTAMDDLDEDEEPAPAMAQRPMQALAVGGPLPLPRPGWLSSPTLGRANRFLSTAAVSLMTPRRPLS
Splooce       DLQRLGGLLSHLRKTLKDDLASKANIVIDPLELQSTAMDDLDEDEEPAPAMAQRPMQALAVGGPLPLPRPGWLSSPTLGRANRFLSTAAVSLMTPRRPLS

Uniprot       TSEKVKVRTLSVEQRTREDIEGSHWNEGLLLGRPPEEPEQPLTENSLLEVLDGAVMMYNLSVHQQLGKMVGVSDDVNEYAMALRDTEDKLRRCPKRRKDI
Splooce       TSEKVKVRTLSVEQRTREDIEGSHWNEGLLLGRPPEEPEQPLTENSLLEVLDGAVMMYNLSVHQQLGKMVGVSDDVNEYAMALRDTEDKLRRCPKRRKDI

Uniprot       LAELTKSQKVFSEKLDHLSRRLAWVHATVYSQEKMLDIYWLLRVCLRTIEHGDRTGSLFAFMPEFYLSVAINSYSALKNYFGPVHSMEELPGYEETLTRL
Splooce       LAELTKSQKVFSEKLDHLSRRLAWVHATVYSQEKMLDIYWLLRVCLRTIEHGDRTGSLFAFMPEFYLSVAINSYSALKNYFGPVHSMEELPGYEETLTRL

Uniprot       AAILAKHFADARIVGTDIRDSLMQALASYVCYPHSLRAVERIPEEQRIAMVRNLLAPYEQRPWAQTNWILVRLWRGCGFGYRYTRLPHLLKTKLEDANLP
Splooce       AAILAKHFADARIVGTDIRDSLMQALASYVCYPHSLRAVERIPEEQRIAMVRNLLAPYEQRPWAQTNWILVRLWR-------------------------

Uniprot       SLQKPCPSTLLQQHMADLLQQGPDVAPSFLNSVLNQLNWAFSEFIGMIQEIQQAAERLERNFVDSRQLKVCATCFDLSVSLLRVLEMTITLVPEIFLDWT
Splooce       --------------------------------------------------IQQAAERLERNFVDSRQLKVCATCFDLSVSLLRVLEMTITLVPEIFLDWT

Uniprot       RPTSEMLLRRLAQLLNQVLNRVTAERNLFDRVVTLRLPGLESVDHYPILVAVTGILVQLLVRGPASEREQATSVLLADPCFQLRSICYLLGQPEPPAPGT
Splooce       RPTSEMLLRRLAQLLNQVLNRVTAERNLFDRVVTLRLPGLESVDHYPILVAVTGILVQLLVRGPASEREQATSVLLADPCFQLRSICYLLGQPEPPAPGT

Uniprot       ALPAPDRKRFSLQSYADYISADELAQVEQMLAHLTSASAQAAAASLPTSEEDLCPICYAHPISAVFQPCGHKSCKACINQHLMNNKDCFFCKTTIVSVED
Splooce       ALPAPDRKRFSLQSYADYISADELAQVEQMLAHLTSASAQAAAASLPTSEEDLCPICYAHPISAVFQPCGHKSCKACINQHLMNNKDCFFCKTTIVSVED

Uniprot       WEKGANTSTTSSAA
Splooce       WEKGANTSTTSSAA

----------------------------------------------------------------------------------------------------

Q9C0J8 (Uniprot)	versus
NM_018383#(-t:2_W5800984775029) (Splooce)

For more details about the Alternative Splicing Event -> Link to Splooce page

Peptides that support the ASE (Splooce-specific):
THVFEGAEKK (MAXQUANT)

Alignment:
Uniprot       MATEIGSPPRFFHMPRFQHQAPRQLFYKRPDFAQQQAMQQLTFDGKRMRKAVNRKTIDYNPSVIKYLENRIWQRDQRDMRAIQPDAGYYNDLVPPIGMLN
Splooce       MATEIGSPPRFFHMPRFQHQAPRQLFYKRPDFAQQQAMQQLTFDGKRMRKAVNRKTIDYNPSVIKYLENRIWQRDQRDMRAIQPDAGYYNDLVPPIGMLN

Uniprot       NPMNAVTTKFVRTSTNKVKCPVFVVRWTPEGRRLVTGASSGEFTLWNGLTFNFETILQAHDSPVRAMTWSHNDMWMLTADHGGYVKYWQSNMNNVKMFQA
Splooce       NPMNAVTTKFVRTSTNKVKCPVFVVRWTPEGRRLVTGASSGEFTLWNGLTFNFETILQAHDSPVRAMTWSHNDMWMLTADHGGYVKYWQSNMNNVKMFQA

Uniprot       HKEAIREASFSPTDNKFATCSDDGTVRIWDFLRCHEERILRGHGADVKCVDWHPTKGLVVSGSKDSQQPIKFWDPKTGQSLATLHAHKNTVMEVKLNLNG
Splooce       HKEAIREASFSPTDNKFATCSDDGTVRIWDFLRCHEERILRGHGADVKCVDWHPTKGLVVSGSKDSQQPIKFWDPKTGQSLATLHAHKNTVMEVKLNLNG

Uniprot       NWLLTASRDHLCKLFDIRNLKEELQVFRGHKKEATAVAWHPVHEGLFASGGSDGSLLFWHVGVEKEVGGMEMAHEGMIWSLAWHPLGHILCSGSNDHTSK
Splooce       NWLLTASRDHLCKLFDIRNLKEELQVFRGHKKEATAVAWHPVHEGLFASGGSDGSLLFWHVGVEKEVGGMEMAHEGMIWSLAWHPLGHILCSGSNDHTSK

Uniprot       FWTRNRPGDKMRDRYNLNLLPGMSEDGVEYDDLEPNSLAVIPGMGIPEQLKLAMEQEQMGKDESNEIEMTIPGLDWGMEEVMQKDQKKVPQKKVPYAKPI
Splooce       FWTRNRPGDKMRDRYNLNLLPGMSEDGVEYDDLEPNSLAVIPGMGIPEQLKLAMEQEQMGKDESNEIEMTIPGLDWGMEEVMQKDQKKVPQKKVPYAKPI

Uniprot       PAQFQQAWMQNKVPIPAPNEVLNDRKEDIKLEEKKKTQAEIEQEMATLQYTNPQLLEQLKIERLAQKQVEQIQPPPSSGTPLLGPQPFPGQGPMSQIPQG
Splooce       PAQFQQAWMQNKVPIPAPNEVLNDRKEDIKLEEKKKTQAEIEQEMATLQYTNPQLLEQLKIERLAQKQVEQIQPPPSSGTPLLGPQPFPGQGPMSQIPQG

Uniprot       FQQPHPSQQMPMNMAQMGPPGPQGQFRPPGPQGQMGPQGPPLHQGGGGPQGFMGPQGPQGPPQGLPRPQDMHGPQGMQRHPGPHGPLGPQGPPGPQGSSG
Splooce       FQQPHPSQQMPMNMAQMGPPGPQGQFRPPGPQGQMGPQGPPLHQGGGGPQGFMGPQGPQGPPQGLPRPQDMHGPQGMQRHPGPHGPLGPQGPPGPQGSSG

Uniprot       PQGHMGPQGPPGPQGHIGPQGPPGPQGHLGPQGPPGTQGMQGPPGPRGMQGPPHPHGIQGGPGSQGIQGPVSQGPLMGLNPRGMQGPPGPRENQGPAPQG
Splooce       PQGHMGPQGPPGPQGHIGPQGPPGPQGHLGPQGPPGTQGMQGPPGPRGMQGPPHPHGIQGGPGSQGIQGPVSQGPLMGLNPRGMQGPPGPRENQGPAPQG

Uniprot       MIMGHPPQEMRGPHPPGGLLGHGPQEMRGPQEIRGMQGPPPQGSMLGPPQELRGPPGSQSQQGPPQGSLGPPPQGGMQGPPGPQGQQNPARGPHPSQGPI
Splooce       MIMGHPPQEMRGPHPPGGLLGHGPQEMRGPQEIRGMQGPPPQGSMLGPPQELRGPPGSQSQQGPPQGSLGPPPQGGMQGPPGPQGQQNPARGPHPSQGPI

Uniprot       PFQQQKTPLLGDGPRAPFNQEGQSTGPPPLIPGLGQQGAQGRIPPLNPGQGPGPNKGDSRGPPNHHMGPMSERRHEQSGGPEHGPERGPFRGGQDCRGPP
Splooce       PFQQQKTPLLGDGPRAPFNQEGQSTGPPPLIPGLGQQGAQGRIPPLNPGQGPGPNKGNS-ARGMAGVQPEALRGHGKAAGPEMN----------------

Uniprot       DRRGPHPDFPDDFSRPDDFHPDKRFGHRLREFEGRGGPLPQEEKWRRGGPGPPFPPDHREFSEGDGRGAARGPPGAWEGRRPGDERFPRDPEDPRFRGRR
Splooce       ----VSPGIP-----------------RTHVFEGAEKKVSEEEPRR--GMRAVLPPEE---------GMVFLVLKTLVQRR-ILMLLRKRPED------E

Uniprot       EESFRRGAPPRHEGRAPPRGRDGFPGPEDFGPEENFDASEEAARGRDLRGRGRGTPRGGRKGLLPTPDEFPRFEGGRKPDSWDGNREPGPGHEHFRDTPR
Splooce       ISEVEVGVPHEEEGRVY------FP---------------LLTSSLALKEGGSQIPGMETESLGQVMNIFVILPALIIP------------LTTVIPQPA

Uniprot       PDHPPHDGHSPASRERSSSLQGMDMASLPPRKRPWHDGPGTSEHREMEAPGGPSEDRGGKGRGGPGPAQRVPKSGRSSSLDGEHHDGYHRDEPFGGPPGS
Splooce       ENAPLLSKAWTWHPYLPESAPGMMAQALLSTER-WRPQEALLKTEEAKAEGAQDLLRECPNLGVPAP---------------------------------

Uniprot       GTPSRGGRSGSNWGRGSNMNSGPPRRGASRGGGRGR
Splooce       ------------------------------------

----------------------------------------------------------------------------------------------------

P00439 (Uniprot)	versus
NM_000277#(f-:12_P6960415512699) (Splooce)

For more details about the Alternative Splicing Event -> Link to Splooce page

Peptides that support the ASE (Splooce-specific):
FSAMERNWMLTTLVLK (MAXQUANT)

Alignment:
Uniprot       MSTAVLENPGLGRKLSDFGQETSYIEDNCNQNGAISLIFSLKEEVGALAKVLRLFEENDVNLTHIESRPSRLKKDEYEFFTHLDKRSLPALTNIIKILRH
Splooce       MTLVPLS-----MSFHEIRRKT-----QCPGS-----------------------QEPFKSWTDLPIRFSAMERN-----------------WMLTTLVL

Uniprot       DIGATVHELSRDKKKDTVPWFPRTIQELDRFANQILSYGAELDADHPGFKDPVYRARRKQFADIAYNYRHGQPIPRVEYMEEEKKTWGTVFKTLKSLYKT
Splooce       KILCTVQDGS------SLLTLPTTTAS------------------------------------------NGQPIPRVEYMEEEKKTWGTVFKTLKSLYKT

Uniprot       HACYEYNHIFPLLEKYCGFHEDNIPQLEDVSQFLQTCTGFRLRPVAGLLSSRDFLGGLAFRVFHCTQYIRHGSKPMYTPEPDICHELLGHVPLFSDRSFA
Splooce       HACYEYNHIFPLLEKYCGFHEDNIPQLEDVSQFLQTCTGFRLRPVAGLLSSRDFLGGLAFRVFHCTQYIRHGSKPMYTPEPDICHELLGHVPLFSDRSFA

Uniprot       QFSQEIGLASLGAPDEYIEKLATIYWFTVEFGLCKQGDSIKAYGAGLLSSFGELQYCLSEKPKLLPLELEKTAIQNYTVTEFQPLYYVAESFNDAKEKVR
Splooce       QFSQEIGLASLGAPDEYIEKLATIYWFTVEFGLCKQGDSIKAYGAGLLSSFGELQYCLSEKPKLLPLELEKTAIQNYTVTEFQPLYYVAESFNDAKEKVR

Uniprot       NFAATIPRPFSVRYDPYTQRIEVLDNTQQLKILADSINSEIGILCSALQKIK
Splooce       NFAATIPRPFSVRYDPYTQRIEVLDNTQQLKILADSINSEIGILCSALQKIK

----------------------------------------------------------------------------------------------------

P06576 (Uniprot)	versus
NM_001686#(-s-s-s-:12_A5786049767184) (Splooce)

For more details about the Alternative Splicing Event -> Link to Splooce page

Peptides that support the ASE (Splooce-specific):
WPSIWVALVYGQMNEPPGAR (MAXQUANT)

Alignment:
Uniprot       MLGFVGRVAAAPASGALRRLTPSASLPPAQLLLRAAPTAVHPVRDYAAQTSPSPKAGAATGRIVAVIGAVVDVQFDEGLPPILNALEVQGRETRLVLEVA
Splooce       ----------------------------------------------------------------------------------------------------

Uniprot       QHLGESTVRTIAMDGTEGLVRGQKVLDSGAPIKIPVGPETLGRIMNVIGEPIDERGPIKTKQFAPIHAEAPEFMEMSVEQEILVTGIKVVDLLAPYAKGG
Splooce       --------------------------------------------------------------------------------------------MPWKCKAG

Uniprot       KIGLFGGAGVGKTVLIMELINNVAKAHGGYSVFAGVGERTREGNDLYHEMIESGVINLKDATSKVALVYGQMNEPPGARARVALTGLTVAEYFRDQEGQD
Splooce       RPDWF----------------------------------------------------WRWPSIWVALVYGQMNEPPGARARVALTGLTVAEYFRDQEGQD

Uniprot       VLLFIDNIFRFTQAGSEVSALLGRIPSAVGYQPTLATDMGTMQERITTTKKGSITSVQAIYVPADDLTDPAPATTFAHLDATTVLSRAIAELGIYPAVDP
Splooce       VLLFIDNIFRFTQAGSEVSALLGRIPSAVGYQPTLATDMGTMQERITTTKKGSITSVQAIYVPADDLTDPAPATTFAHLDATTVLSRAIAELGIYPAVDP

Uniprot       LDSTSRIMDPNIVGSEHYDVARGVQKILQDYKSLQDIIAILGMDELSEEDKLTVSRARKIQRFLSQPFQVAEVFTGHMGKLVPLKETIKGFQQILAGEYD
Splooce       LDSTSRIMDPNIVGSEHYDVARGVQKILQDYKSLQDIIAILGMDELSEEDKLTVSRARKIQRFLSQPFQVAEVFTGHMGKLVPLKETIKGFQQILAGEYD

Uniprot       HLPEQAFYMVGPIEEAVAKADKLAEEHSS
Splooce       HLPEQAFYMVGPIEEAVAKADKLAEEHSS

----------------------------------------------------------------------------------------------------

P00505 (Uniprot)	versus
NM_002080#(-s-:16_G2134707188382) (Splooce)

For more details about the Alternative Splicing Event -> Link to Splooce page

Peptides that support the ASE (Splooce-specific):
IGASFLK (MAXQUANT)

Alignment:
Uniprot       MALLHSGRVLPGIAAAFHPGLAAAASARASSWWTHVEMGPPDPILGVTEAFKRDTNSKKMNLGVGAYRDDNGKPYVLPSVRKAEAQIAAKNLDKEYLPIG
Splooce       MALLHSGRVLPGIAAAFHPGLAAAASARASSWWTHVEMGPPDPILGVTEAFKRDTNSKKMNLGVGAYRDDNGKPYVLPSVRKAEAQIAAKNLDKEYLPIG

Uniprot       GLAEFCKASAELALGENSEVLKSGRFVTVQTISGTGALRIGASFLQRFFKFSRDVFLPKPTWGNHTPIFRDAGMQLQGYRYYDPKTCGFDFTGAVEDISK
Splooce       GLAEFCKASAELALGENSEVLKSGRFVTVQTISGTGALRIGASFLK------------------------------------------------------

Uniprot       IPEQSVLLLHACAHNPTGVDPRPEQWKEIATVVKKRNLFAFFDMAYQGFASGDGDKDAWAVRHFIEQGINVCLCQSYAKNMGLYGERVGAFTMVCKDADE
Splooce       IPEQSVLLLHACAHNPTGVDPRPEQWKEIATVVKKRNLFAFFDMAYQGFASGDGDKDAWAVRHFIEQGINVCLCQSYAKNMGLYGERVGAFTMVCKDADE

Uniprot       AKRVESQLKILIRPMYSNPPLNGARIAAAILNTPDLRKQWLQEVKVMADRIIGMRTQLVSNLKKEGSTHNWQHITDQIGMFCFTGLKPEQVERLIKEFSI
Splooce       AKRVESQLKILIRPMYSNPPLNGARIAAAILNTPDLRKQWLQEVKVMADRIIGMRTQLVSNLKKEGSTHNWQHITDQIGMFCFTGLKPEQVERLIKEFSI

Uniprot       YMTKDGRISVAGVTSSNVGYLAHAIHQVTK
Splooce       YMTKDGRISVAGVTSSNVGYLAHAIHQVTK

----------------------------------------------------------------------------------------------------

O60308 (Uniprot)	versus
NM_014704#(-s-s-s-s-:1_K8200800156545) (Splooce)

For more details about the Alternative Splicing Event -> Link to Splooce page

Peptides that support the ASE (Splooce-specific):
EIQAEVQLPNR (MAXQUANT)

Alignment:
Uniprot       MPHKIGFVVVSSSGHEDGFSARELMIHAPTVSGWRSPRFCQFPQEIVLQMVERCRIRKLQLLAHQYMISSKIEFYISESLPEYFAPYQAERFRRLGYVSL
Splooce       MPHKIGFVVVSSSGHEDGFSARELMIHAPTVSGWRSPRFCQFPQEIVLQMVERCRIRKLQLLAHQYMISSKIEFYISESLPEYFAPYQAERFRRLGYVSL

Uniprot       CDNEKTGCKARELKSVYVDAVGQFLKLIFHQNHVNKYNIYNQVALVAINIIGDPADFSDESNTASREKLIDHYLGHNSEDPALEGTYARKSDYISPLDDL
Splooce       CDNEKTGCKARELKSVYVDAVGQFLKLIFHQNHVNKYNIYNQVALVAINIIGDPADFSDESNTASREKLIDHYLGHNSEDPALEGTYARKSDYISPLDDL

Uniprot       AFDMYQDPEVAQIIRKLDERKREAVQKERYDYAKKLKQAIADLQKVGERLGRYEVEKRCAVEKEDYDLAKEKKQQMEQYRAEVYEQLELHSLLDAELMRR
Splooce       AFDMYQDPEVAQIIRKLDERKREAVQKERYDYAKKLKQAIADLQKVGERLGRYEVEKRCAVEKEDYDLAKEKKQQMEQYRAEVYEQLELHSLLDAELMRR

Uniprot       PFDLPLQPLARSGSPCHQKPMPSLPQLEERGTENQFAEPFLQEKPSSYSLTISPQHSAVDPLLPATDPHPKINAESLPYDERPLPAIRKHYGEAVVEPEM
Splooce       PFDLPLQPLARSGSPCHQKPMPSLPQLEERGTENQFAEPFLQEKPSSYSLTISPQHSAVDPLLPATDPHPKINAESLPYDERPLPAIRKHYGEAVVEPEM

Uniprot       SNADISDARRGGMLGEPEPLTEKALREASSAIDVLGETLVAEAYCKTWSYREDALLALSKKLMEMPVGTPKEDLKNTLRASVFLVRRAIKDIVTSVFQAS
Splooce       SNADISDARRGGMLGEPEPLTEKALREASSAIDVLGETLVAEAYCKTWSYREDALLALSKKLMEMPVGTPKEDLKNTLRASVFLVRRAIKDIVTSVFQAS

Uniprot       LKLLKMIITQYIPKHKLSKLETAHCVERTIPVLLTRTGDSSARLRVTAANFIQEMALFKEVKSLQIIPSYLVQPLKANSSVHLAMSQMGLLARLLKDLGT
Splooce       LKLLKMIITQYIPKHKLSKLETAHCVERTIPVLLTRTGDSSARLRVTAANFIQEMALFKEVKSLQIIPSYLVQPLKANSSVHLAMSQMGLLARLLKDLGT

Uniprot       GSSGFTIDNVMKFSVSALEHRVYEVRETAVRIILDMYRQHQASILEYLPPDDSNTRRNILYKTIFEGFAKIDGRATDAEMRARRKAATEEAEKQKKEEIK
Splooce       GSSGFTIDNVMKFSVSALEHRVYEVRETAVRIILDMYRQHQASILEYLPPDDSNTRRNILYKTIFEGFAKIDGRATDAEMRARRKAATEEAEKQKKEEIK

Uniprot       ALQGQLAALKEIQAEVQEKESDAVKPKNQDIQGGKAAPAEALGIPDEHYLDNLCIFCGERSESFTEEGLDLHYWKHCLMLTRCDHCKQVVEISSLTEHLL
Splooce       ALQGQLAALKEIQAEVQ-------LPNRRSWQTG--------------------VPCVMRTSALEKR-----------------HGKLT-----------

Uniprot       TECDKKDGFGKCYRCSEAVFKEELPRHIKHKDCNPAKPEKLANRCPLCHENFSPGEEAWKAHLMGPAGCTMNLRKTHILQKAPALQPGKSSAVAASGPLG
Splooce       ----------------------------------------------------------------------------------------------------

Uniprot       SKAGSKIPTPKGGLSKSSSRTYAKR
Splooce       -------------------------

----------------------------------------------------------------------------------------------------

Q00341 (Uniprot)	versus
NM_203346#(-s-s-s-:2_H5267870912087) (Splooce)

For more details about the Alternative Splicing Event -> Link to Splooce page

Peptides that support the ASE (Splooce-specific):
LQTQANSFTVSSVAAPSWLHR (MAXQUANT)

Alignment:
Uniprot       MSSVAVLTQESFAEHRSGLVPQQIKVATLNSEEESDPPTYKDAFPPLPEKAACLESAQEPSGAWGNKIRPIKASVITQVFHVPLEERKYKDMNQFGEGEQ
Splooce       MSSVAVLTQESFAEHRSGLVPQQIKVATLNSEEESDPPTYKDAFPPLPEKAACLESAQEPSGAWGNKIRPIKASVITQVFHVPLEERKYKDMNQFGEGEQ

Uniprot       AKICLEIMQRTGAHLELSLAKDQGLSIMVSGKLDAVMKARKDIVARLQTQASATVAIPKEHHRFVIGKNGEKLQDLELKTATKIQIPRPDDPSNQIKITG
Splooce       AKICLEIMQRTGAHLELSLAKDQGLSIMVSGKLDAVMKARKDIVARLQTQA-------------------------------------------------

Uniprot       TKEGIEKARHEVLLISAEQDKRAVERLEVEKAFHPFIAGPYNRLVGEIMQETGTRINIPPPSVNRTEIVFTGEKEQLAQAVARIKKIYEEKKKKTTTIAV
Splooce       ----------------------------------------------------------------------------------------------------

Uniprot       EVKKSQHKYVIGPKGNSLQEILERTGVSVEIPPSDSISETVILRGEPEKLGQALTEVYAKANSFTVSSVAAPSWLHRFIIGKKGQNLAKITQQMPKVHIE
Splooce       -------------------------------------------------------------NSFTVSSVAAPSWLHRFIIGKKGQNLAKITQQMPKVHIE

Uniprot       FTEGEDKITLEGPTEDVNVAQEQIEGMVKDLINRMDYVEINIDHKFHRHLIGKSGANINRIKDQYKVSVRIPPDSEKSNLIRIEGDPQGVQQAKRELLEL
Splooce       FTEGEDKITLEGPTEDVNVAQEQIEGMVKDLINRMDYVEINIDHKFHRHLIGKSGANINRIKDQYKVSVRIPPDSEKSNLIRIEGDPQGVQQAKRELLEL

Uniprot       ASRMENERTKDLIIEQRFHRTIIGQKGERIREIRDKFPEVIINFPDPAQKSDIVQLRGPKNEVEKCTKYMQKMVADLVENSYSISVPIFKQFHKNIIGKG
Splooce       ASRMENERTKDLIIEQRFHRTIIGQKGERIREIRDKFPEVIINFPDPAQKSDIVQLRGPKNEVEKCTKYMQKMVADLVENSYSISVPIFKQFHKNIIGKG

Uniprot       GANIKKIREESNTKIDLPAENSNSETIIITGKRANCEAARSRILSIQKDLANIAEVEVSIPAKLHNSLIGTKGRLIRSIMEECGGVHIHFPVEGSGSDTV
Splooce       GANIKKIREESNTKIDLPAENSNSETIIITGKRANCEAARSRILSIQKDLANIAEVEVSIPAKLHNSLIGTKGRLIRSIMEECGGVHIHFPVEGSGSDTV

Uniprot       VIRGPSSDVEKAKKQLLHLAEEKQTKSFTVDIRAKPEYHKFLIGKGGGKIRKVRDSTGARVIFPAAEDKDQDLITIIGKEDAVREAQKELEALIQNLDNV
Splooce       VIRGPSSDVEKAKKQLLHLAEEKQTKSFTVDIRAKPEYHKFLIGKGGGKIRKVRDSTGARVIFPAAEDKDQDLITIIGKEDAVREAQKELEALIQNLDNV

Uniprot       VEDSMLVDPKHHRHFVIRRGQVLREIAEEYGGVMVSFPRSGTQSDKVTLKGAKDCVEAAKKRIQEIIEDLEAQVTLECAIPQKFHRSVMGPKGSRIQQIT
Splooce       VEDSMLVDPKHHRHFVIRRGQVLREIAEEYGGVMVSFPRSGTQSDKVTLKGAKDCVEAAKKRIQEIIEDLEAQVTLECAIPQKFHRSVMGPKGSRIQQIT

Uniprot       RDFSVQIKFPDREENAVHSTEPVVQENGDEAGEGREAKDCDPGSPRRCDIIIISGRKEKCEAAKEALEALVPVTIEVEVPFDLHRYVIGQKGSGIRKMMD
Splooce       RDFSVQIKFPDREENAVHSTEPVVQENGDEAGEGREAKDCDPGSPRRCDIIIISGRKEKCEAAKEALEALVPVTIEVEVPFDLHRYVIGQKGSGIRKMMD

Uniprot       EFEVNIHVPAPELQSDIIAITGLAANLDRAKAGLLERVKELQAEQEDRALRSFKLSVTVDPKYHPKIIGRKGAVITQIRLEHDVNIQFPDKDDGNQPQDQ
Splooce       EFEVNIHVPAPELQSDIIAITGLAANLDRAKAGLLERVKELQAEQEDRALRSFKLSVTVDPKYHPKIIGRKGAVITQIRLEHDVNIQFPDKDDGNQPQDQ

Uniprot       ITITGYEKNTEAARDAILRIVGELEQMVSEDVPLDHRVHARIIGARGKAIRKIMDEFKVDIRFPQSGAPDPNCVTVTGLPENVEEAIDHILNLEEEYLAD
Splooce       ITITGYEKNTEAARDAILRIVGELEQMVSEDVPLDHRVHARIIGARGKAIRKIMDEFKVDIRFPQSGAPDPNCVTVTGLPENVEEAIDHILNLEEEYLAD

Uniprot       VVDSEALQVYMKPPAHEEAKAPSRGFVVRDAPWTASSSEKAPDMSSSEEFPSFGAQVAPKTLPWGPKR
Splooce       VVDSEALQVYMKPPAHEEAKAPSRGFVVRDAPWTASSSEKAPDMSSSEEFPSFGAQVAPKTLPWGPKR

----------------------------------------------------------------------------------------------------

Q8N5M0 (Uniprot)	versus
NM_005804#(-s-s-:19_D2514269065093) (Splooce)

For more details about the Alternative Splicing Event -> Link to Splooce page

Peptides that support the ASE (Splooce-specific):
PMEVFVDDETK (PEAKS)

Alignment:
Uniprot       MAEQDVENDLLDYDEEEEPQAPQESTPAPPKKDIKGSYVSIHSSGFRDFLLKPELLRAIVDCGFEHPSEVQHECIPQAILGMDVLCQAKSGMGKTAVFVL
[truncated: 789,036 more chars]
